# Divergent Risks of Hematologic Malignancies Associated with GLP-1 Receptor Agonists and SGLT2 Inhibitors: Preliminary Findings from a Pilot Network Meta-Analysis

**DOI:** 10.3390/biom15111622

**Published:** 2025-11-19

**Authors:** Pao-Yen Lin, Bing-Yan Zeng, Chih-Wei Hsu, Mein-Woei Suen, Chao-Ming Hung, Brendon Stubbs, Yen-Wen Chen, Tien-Yu Chen, Wei-Te Lei, Jiann-Jy Chen, Bing-Syuan Zeng, Kuan-Pin Su, Chih-Sung Liang, Ping-Tao Tseng

**Affiliations:** 1 Department of Psychiatry, Kaohsiung Chang Gung Memorial Hospital and Chang Gung University College of Medicine, Kaohsiung 833, Taiwan; paoyenlin@gmail.com (P.-Y.L.); harwicacademia@gmail.com (C.-W.H.); 2 Institute for Translational Research in Biomedical Sciences, Kaohsiung Chang Gung Memorial Hospital, Kaohsiung 833, Taiwan; 3 Institute of Biomedical Sciences, National Sun Yat-sen University, Kaohsiung 804, Taiwan; holdinggreat@yahoo.com.tw; 4 Department of Internal Medicine, E-Da Dachang Hospital, I-Shou University, Kaohsiung 807, Taiwan; 5 Department of Psychology, College of Medical and Health Science, Asia University, Taichung 413, Taiwan; blake@asia.edu.tw (M.-W.S.); cobolsu@gmail.com (K.-P.S.); 6 Gender Equality Education and Research Center, Asia University, Taichung 413, Taiwan; 7 Department of Medical Research, Asia University Hospital, Asia University, Taichung 413, Taiwan; 8 Department of Medical Research, China Medical University Hospital, China Medical University, Taichung 404, Taiwan; 9 Division of General Surgery, Department of Surgery, E-Da Cancer Hospital, I-Shou University, Kaohsiung 824, Taiwan; ed100647@edah.org.tw; 10 School of Medicine, College of Medicine, I-Shou University, Kaohsiung 824, Taiwan; 11 Department of Psychological Medicine, Institute of Psychiatry, Psychology and Neuroscience, King’s College London, London WC2R 2LS, UK; brendon.stubbs@kcl.ac.uk (B.S.); cobolsu@gmail.com (K.-P.S.); 12 Department of Sport, University of Vienna, Vienna 1030, Austria; 13 Prospect Clinic for Otorhinolaryngology & Neurology, Kaohsiung 811, Taiwan; kevinachen0527@gmail.com (Y.-W.C.); jiannjy@yahoo.com.tw (J.-J.C.); 14 Department of Psychiatry, Tri-Service General Hospital, National Defense Medical University, Taipei 114, Taiwan; verducciwol@gmail.com; 15 Department of Psychiatry, College of Medicine, National Defense Medical University, Taipei 114, Taiwan; 16 Section of Immunology, Rheumatology, and Allergy Department of Pediatrics, Hsinchu Munipical MacKay Children’s Hospital, Hsinchu 300, Taiwan; lazyleisure@gmail.com; 17 Center for Molecular and Clinical Immunology, Chang Gung University, Taoyuan 333, Taiwan; 18 Department of Otorhinolaryngology, E-Da Cancer Hospital, I-Shou University, Kaohsiung 824, Taiwan; 19 Department of Internal Medicine, E-Da Cancer Hospital, I-Shou University, Kaohsiung 824, Taiwan; b95401072@ntu.edu.tw; 20 Mind-Body Interface Research Center (MBI Lab & Care), China Medical University Hospital, Taichung 404, Taiwan; 21 College of Medicine, China Medical University, Taichung 404, Taiwan; 22 An-Nan Hospital, China Medical University, Tainan 709, Taiwan; 23 Department of Psychiatry, Beitou Branch, Tri-Service General Hospital, School of Medicine, National Defense Medical University, Taipei 112, Taiwan; 24 Department of Psychiatry, National Defense Medical University, Taipei 114, Taiwan; 25 Institute of Precision Medicine, National Sun Yat-sen University, Kaohsiung 804, Taiwan

**Keywords:** network meta-analysis, GLP-1 receptor agonist, SGLT2 inhibitor, hematology, malignancy, lymphoma, leukemia, myeloma

## Abstract

Background: Although glucagon-like peptide-1 (GLP-1) receptor agonists and sodium–glucose cotransporter 2 (SGLT2) inhibitors have gained attention for their broad therapeutic effects, their influence on hematologic malignancy remains underexplored. Given the high mortality associated with hematologic cancers, clarifying the impact of these agents on such malignancies is essential. Objectives: This pilot network meta-analysis (NMA) aimed to assess the comparative risk of hematologic malignancies—including lymphoma, leukemia, and myeloma—associated with various GLP-1 receptor agonists and SGLT2 inhibitors. Methods: Following Cochrane-recommended confirmatory methods, we systematically searched multiple databases for randomized controlled trials (RCTs) published through 4 December 2024. The primary outcome was the incidence of overall hematologic malignancies. A frequentist random-effects NMA via the netmeta package was conducted, with additional validation through Bayesian NMA for solely sensitivity analyses. Results: Fifty-five RCTs (*n* = 200,606) were analyzed. Dulaglutide showed a significantly higher risk of overall hematologic malignancy [odds ratio (OR) = 2.18, 95% confidence interval (95%CI) = 1.14–4.19). In contrast, tirzepatide was linked to a significantly reduced risk (OR = 0.14, 95%CI = 0.03–0.60), especially for lymphoma. No statistically significant associations were identified for SGLT2 inhibitors (i.e., 95%CI across 1.0). Conclusions: Our preliminary findings reveal distinct and agent-specific effects of GLP-1 receptor agonists on hematologic malignancy risk. While dulaglutide may elevate the risk, tirzepatide appears protective, particularly against lymphoma. These results call for further long-term mechanistic studies to clarify causality and underlying pathways.

## 1. Introduction

Glucagon-like peptide-1 (GLP-1) receptor agonists and sodium–glucose cotransporter 2 (SGLT2) inhibitors have emerged as innovative classes of antihyperglycemic agents, acting through mechanisms distinct from traditional therapies [1]. As the clinical application of these agents continues to grow, attention has also turned to their long-term safety profiles regarding cancer risk. While the relationship between these agents and solid malignancies [2] has been moderately explored, their influence on hematologic cancers remains largely uncharted. Previous reports had demonstrated that diabetes mellitus was associated with a higher risk of hematological malignancies and is an independent risk factor of all-cause and cause-specific mortality [3,4]. To date, only a few investigations have focused specifically on hematologic malignancies in the context of GLP-1 receptor agonist [5] or SGLT2 inhibitor [6] therapy. This knowledge gap is particularly significant given the generally poor prognosis associated with hematologic malignancies [7]. Fortunately, many large-scale randomized controlled trials (RCTs) have systematically captured adverse events, including hematologic cancers, within their safety assessments [8,9,10,11].

When RCTs are limited in duration or statistical power to assess rare adverse outcomes, meta-analytic methods can serve as a valuable tool to aggregate evidence. These methods harness data from extensive clinical trials and observational studies, offering insights that may reflect real-world clinical trends [12,13]. A well-executed network meta-analysis (NMA) is particularly advantageous, as it enables indirect comparisons among multiple interventions and facilitates the identification of superior regimens or doses [14]. Such approaches enhance evidence-based decision-making in clinical pharmacology.

Given the above, we undertook a pilot NMA to examine the comparative risks of hematologic malignancies associated with various GLP-1 receptor agonists and SGLT2 inhibitors. Our analytical framework builds upon previous studies examining neurological [15], colorectal cancer [2], metastatic cancer [16], and audiovestibular outcomes [17] related to these agents. This study represents the first NMA, to our knowledge, focusing specifically on hematologic malignancy risk in this drug class.

## 2. Methods

### 2.1. Study Design and Analytical Framework

This pilot NMA employed a hypothesis-driven design to preliminarily evaluate the hematologic malignancy risks linked to GLP-1 receptor agonists and SGLT2 inhibitors. Following the Cochrane Collaboration’s guidelines for adverse event-focused synthesis [18], we concentrated on predefined outcomes rather than general safety profiles. The review was conducted in accordance with the PRISMA extension statement for network meta-analyses (PRISMA-NMA) [19] (refer to Table S1). Our study was prospectively registered on PROSPERO (CRD42024622310) and approved by the Tri-Service General Hospital’s Institutional Review Board (TSGHIRB E202516007).

### 2.2. Literature Search Strategy

A broad and systematic literature search was conducted across eight major databases—PubMed, Embase, Cochrane CENTRAL, ClinicalTrials.gov, ProQuest, ScienceDirect, Web of Science, and ClinicalKey—up to 4 December 2024 (Table S2). Two independent reviewers (PT Tseng and BY Zeng) performed the selection process, starting with screening of titles and abstracts. Full-text evaluation was used for eligibility determination. Conflicts were resolved through mutual discussion. Additionally, reference lists from eligible reviews and prior meta-analyses were manually examined to identify supplementary studies. No filters for language were applied.

### 2.3. Eligibility Criteria

Study selection was guided by the PICOS model:

Population: Individuals without a prior diagnosis of hematologic malignancy

Intervention: Any GLP-1 receptor agonist or SGLT2 inhibitor, regardless of dosage

Comparator: Placebo, standard of care, or active control group

Outcome: Hematologic malignancy incidence

Study design: Randomized controlled trials (RCTs)

RCTs were eligible if they enrolled patients free from hematologic malignancy at baseline and evaluated the use of GLP-1 receptor agonists or SGLT2 inhibitors in human subjects, with either systematic adverse event tracking or explicit reporting of hematologic outcomes. Studies were excluded if they (1) involved pre-existing hematologic malignancies, (2) lacked a direct comparison group, (3) did not report hematologic events, (4) used a non-randomized design, or (5) were based on animal models. To reduce selective outcome reporting bias, only trials with structured monitoring of adverse events were retained [20].

### 2.4. Risk of Bias Assessment

Risk of bias was independently assessed by two reviewers using the Cochrane Risk of Bias Tool version 1.0 [21]. Any disagreement was resolved by consensus with a third investigator.

### 2.5. Outcome Definitions and Dose Categorization

Hematologic malignancies included in this pilot NMA were categorized as lymphoma, leukemia, and myeloma, based on definitions from the Global Burden of Disease project [22] and the 5th edition of the WHO classification of hematolymphoid neoplasms [23]. Dropout rate from trials, representing discontinuation for any cause, was used as a proxy for overall tolerability.

Medication dosages were stratified in accordance with definitions from their respective source trials:

Canagliflozin: Low: 100 mg; High: 300 mg.

Efpeglenatide: Low: 2 mg; Medium: 4 mg; High: 6 mg.

Ertugliflozin: Low: 5 mg; High: 15 mg.

Dapagliflozin: Low: 2.5 mg; Medium: 5 mg; High: 10 mg.

Injectable Semaglutide: Low: 0.5 mg; Medium: 1.0 mg; High: 2.4 mg.

Empagliflozin: Low: 1–10 mg; High: 25–50 mg.

### 2.6. Data Extraction and Handling

Two authors (PT Tseng and BY Zeng) independently extracted data on study design, patient demographics, intervention characteristics, malignancy outcomes, and trial discontinuations. Any unclear or missing information was clarified through correspondence with study authors. The extraction process followed Cochrane Handbook standards for systematic reviews [24].

### 2.7. Statistical Methodology

Random-effects models were utilized for all treatment comparisons involving more than one active comparator [25], and analyses were conducted using MetaInsight version 4.0.2 (Complex Reviews Support Unit, National Institute for Health Research, UK), which employs the *netmeta* package in R for frequentist NMAs [26].

When encountering single-zero-event arms, we applied a continuity correction to stabilize variance estimates, whereas studies with zero events in both arms were excluded to avoid underestimation of treatment effects [27,28]. Results were presented as odds ratios (ORs) with 95% confidence intervals (CIs), displayed via forest plots [29]. A network graph was constructed to visualize direct and indirect treatment connections. To assess agreement between direct and indirect comparisons, we implemented the node-splitting approach [26,30]. Statistical significance was defined as a two-sided *p*-value < 0.05.

### 2.8. Sensitivity and Subgroup Analyses

We conducted stratified analyses based on hematologic malignancy subtype—lymphoma, leukemia, and myeloma—to examine treatment-specific patterns. Further, a Bayesian NMA framework was used to validate the frequentist findings and assess model stability. Surface under the cumulative ranking curve (SUCRA) values were calculated to determine treatment hierarchy using Litmus Rank-O-Gram and radial SUCRA plots [31]. A deviation-from-model-fit analysis was conducted to assess potential inconsistency in treatment effects [32]. Finally, we applied the GRADE framework to evaluate the certainty of evidence across network estimates [33].

### 2.9. Ethics

All procedures adhered to the ethical principles of the Declaration of Helsinki.

## 3. Results

### 3.1. Study Selection and Characteristics

**Figure 1:**
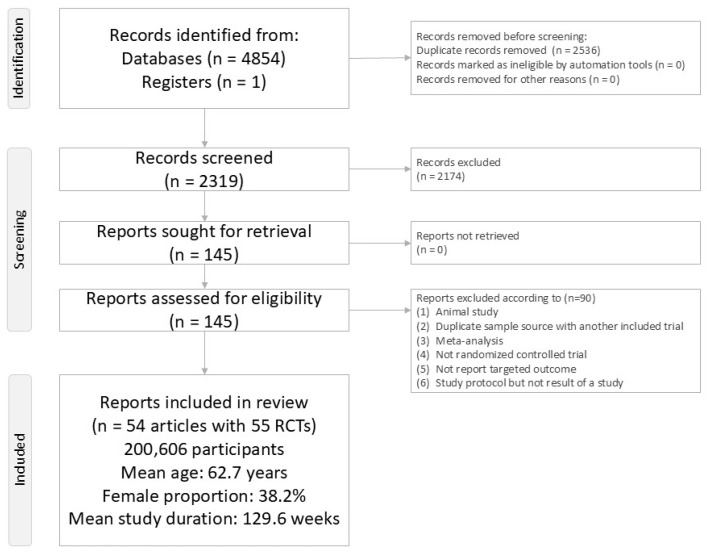
PRISMA2020 Flowchart of current network meta-analysis.

The literature review and selection process are illustrated in Figure 1. After removing 90 articles for failing to meet inclusion criteria (see Table S3), 54 publications encompassing 55 randomized controlled trials (RCTs) were included for analysis (Table S4) [8,9,10,11,34,35,36,37,38,39,40,41,42,43,44,45,46,47,48,49,50,51,52,53,54,55,56,57,58,59,60,61,62,63,64,65,66,67,68,69,70,71,72,73,74,75,76,77,78,79,80,81,82,83]. These trials enrolled a total of 200,606 participants, with a mean age of 62.7 years (range: 41.2 to 71.9 years) and an average female proportion of 38.2% (range: 20.8–79.0%). The median follow-up period was 129.6 weeks, ranging from 12 to 281 weeks.

Across the included studies, we identified 24 distinct treatment arms: one standard-of-care or placebo group, and 23 active comparator groups representing various GLP-1 receptor agonists and SGLT2 inhibitors across multiple dosing regimens. GLP-1 receptor agonists examined included tirzepatide, efpeglenatide, liraglutide, albiglutide, dulaglutide, exenatide, semaglutide, and lixisenatide. SGLT2 inhibitors included bexagliflozin, canagliflozin, empagliflozin, ertugliflozin, dapagliflozin, and sotagliflozin.

### 3.2. Primary Outcome: Incidence of Overall Hematologic Malignancies

**Figure 2:**
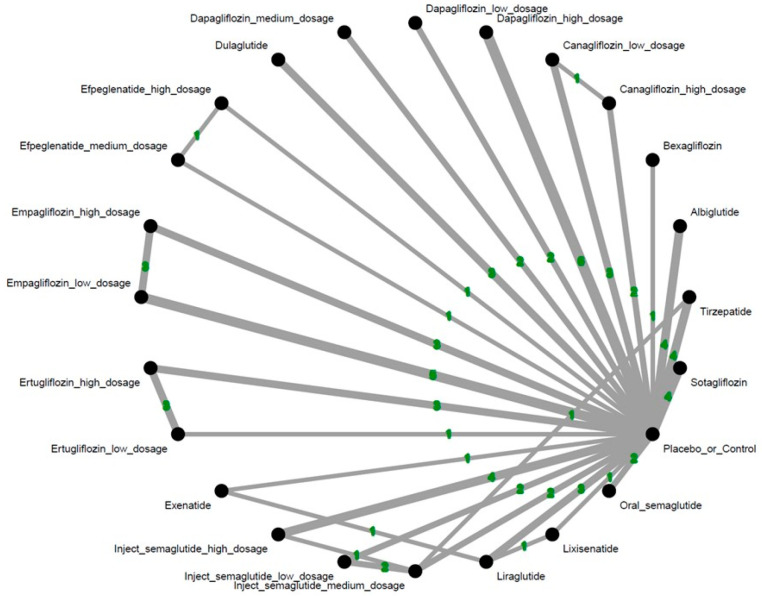
Network structure of the primary outcome: overall hematologic malignancy. The overall structure of the network meta-analysis. The lines between nodes represent direct comparisons from various trials, with the green numbers over the lines indicating the number of trials providing these comparisons for each specific treatment. The thickness of the lines corresponds to the number of trials linked to the network.

**Figure 3:**
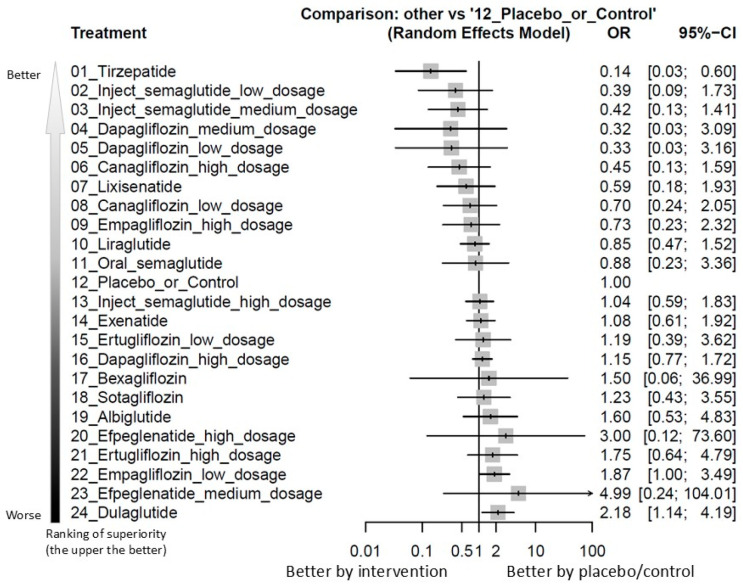
Forest plot of primary outcome: overall hematologic malignancy. When the effect size (expressed as odds ratio) is less than 1, the specified treatment is associated with fewer hematologic malignancy events compared to placebo/controls. Abbreviation: 95%CIs: 95% confidence intervals; GLP-1 agonist: glucagon-like peptide-1 agonist; NMA: network meta-analysis; OR: odds ratio; RCT: randomized controlled trial; SGLT2 inhibitor: sodium–glucose cotransporter 2 inhibitor.

In this pilot NMA preliminarily evaluating all hematologic malignancies combined, dulaglutide was associated with a significantly elevated risk compared to control (OR = 2.18; 95% CI = 1.14–4.19). In contrast, tirzepatide demonstrated a significantly protective association, with markedly lower odds of hematologic malignancy compared to the control group (OR = 0.14; 95% CI = 0.03–0.60). Among all agents assessed, tirzepatide ranked as the safest in this domain (see Figure 2 and Figure 3, Figure S3A, and Table 1).

### 3.3. Subgroup Analysis: Specific Hematologic Cancer Types

When broken down by specific cancer subtype, tirzepatide continued to demonstrate a statistically significant reduction in lymphoma risk (OR = 0.18; 95% CI = 0.03–0.93) compared to the control arm. No other agent showed a significant increase or decrease in lymphoma risk relative to control (see Figures S1A, S2A and S3B, and Table S5A).

As for the risk of leukemia and myeloma, none of the investigated interventions exhibited statistically significant associations when compared with control groups (Figures S1B,C, S2B,C and S3C,D, and Table S5B,C).

### 3.4. Safety Profile: Dropout Rate

Dropout rate was used as a surrogate marker of overall tolerability. Several agents were associated with significantly lower discontinuation rates than the control group, including liraglutide (OR = 0.50; 95% CI = 0.38–0.66), tirzepatide (OR = 0.56; 95% CI = 0.42–0.74), medium-dose injectable semaglutide (OR = 0.65; 95% CI = 0.43–0.98), high-dose injectable semaglutide (OR = 0.71; 95% CI = 0.52–0.96), and high-dose empagliflozin (OR = 0.74; 95% CI = 0.56–0.97). Among all agents, liraglutide ranked highest for treatment retention, followed by tirzepatide (Figures S1D, S2D and S3E, and Table S5D).

### 3.5. Robustness Evaluation: Bayesian-Based Sensitivity Analysis

To verify the consistency of findings, we performed parallel analyses using a Bayesian framework. The direction and magnitude of treatment effects in Bayesian NMA aligned closely with those obtained from frequentist models (Figure S4A–E). Drug rankings by Bayesian SUCRA values are displayed in Table S6A–E and Figure S5A–J. No significant deviation was detected in model fit across treatment comparisons as assessed by deviation-from-consistency modeling (Figure S6A–O).

### 3.6. Risk of Bias and Inconsistency Assessment

Across all studies, 78.7% (303 of 385 items) were judged to have a low risk of bias, 15.8% (61 items) as unclear, and 5.5% (21 items) as high risk (Figure S7A,B). Evaluation of consistency between direct and indirect evidence did not reveal significant discrepancies (Table S7A–E). The overall quality of evidence supporting the primary and secondary outcomes was rated as moderate to high using the GRADE approach (Table S8A–E).

## 4. Discussion

This pilot NMA offers preliminary views into the distinct hematologic safety profiles of GLP-1 receptor agonists and SGLT2 inhibitors. Among the GLP-1 receptor agonists, tirzepatide stood out for its association with a significantly reduced incidence of hematologic malignancy—especially lymphoma—while dulaglutide was linked to an elevated risk. In contrast, no significant associations were detected between any SGLT2 inhibitor and the risk of blood cancers. The divergent trends observed between tirzepatide and dulaglutide are particularly noteworthy, considering their shared classification within the GLP-1 receptor agonist family. These findings suggest that potential hematologic effects may differ considerably across agents, even within the same drug class.

One of the most prominent observations in this pilot study was the elevated risk of hematologic malignancy associated with dulaglutide. A possible mechanistic explanation may involve dulaglutide’s influence on apoptosis-related signaling cascades. Preclinical studies have suggested that dulaglutide can enhance anti-apoptotic activity by modulating the BCL2/BAX/c-caspase3 axis, a pathway implicated in tumor progression and cell survival [84]. Though a case linking dulaglutide to medullary thyroid carcinoma has been reported [85], evidence for its association with hematologic malignancy is still lacking in clinical settings. Another theoretical mechanism involves dulaglutide’s impact on gene methylation. One study reported that dulaglutide induces demethylation of CDH1, the gene encoding E-cadherin—a cell adhesion molecule whose dysregulation has been implicated in certain leukemias [86,87]. While these molecular signals could hypothetically explain the observed associations, no direct clinical evidence has yet confirmed causality. Therefore, the potential linkage between hematologic malignancy and such medication still remained early speculation. Thus, further clinical and mechanistic investigations are required to establish a clearer link between dulaglutide use and hematologic cancer development.

Conversely, tirzepatide was associated with a protective effect against overall hematologic malignancies, most notably in reducing lymphoma risk. In fact, the tirzepatide exerted a different reaction on GLP-1 receptor from those of traditional GLP-1 receptor agonists. Tirzepatide is a biased agonist of GLP-1 receptor with significantly weaker affinity to GLP-1 receptor than natural GLP-1 receptor agonists (such as dulaglutide) [88]. Specifically, tirzepatide could act more on enhancing cAMP expression but less on β arrestin 2 recruitment [89]. Previous reports had demonstrated the potential role of β arrestin 2 on carcinogenesis [90]. Although evidence from clinical trials on tirzepatide’s role in oncology is still sparse [91], early preclinical studies suggest it may inhibit oncogenic pathways. For example, Kong et al. demonstrated that tirzepatide exerted weight-dependent anti-tumor effects in mice, modulating metabolic and signaling pathways based on the animals’ baseline obesity status [91]. In obese mice, the compound suppressed glycolytic and ErbB signaling—two pathways linked to cancer proliferation. In leaner models, tirzepatide enhanced O-linked glycosylation and phospholipase D signaling. Overactivation of ErbB pathways is well-documented in multiple tumor types [92], and inhibition of this axis is an emerging strategy in cancer therapeutics [93]. Although these findings are promising, they remain largely theoretical and early speculated based on animal models. Human studies explicitly designed to assess tirzepatide’s potential anti-cancer effects are still lacking and urgently needed. Future large-scale clinical trials and NMAs should be warranted to support or refute the result of our study.

### Strengths and Limitations

Our analysis possesses several methodological advantages that enhance its clinical relevance. The use of NMA enabled us to perform indirect comparisons across a broad spectrum of GLP-1 receptor agonists and SGLT2 inhibitors, generating more granular insights than conventional meta-analyses. By restricting our inclusion criteria to RCTs, we ensured that the source data were of high methodological quality. Furthermore, we excluded patients with pre-existing hematologic malignancies, allowing for a more accurate assessment of drug-related causality or prophylaxis. We also carried out malignancy-type-specific subgroup analyses, providing more detailed results to inform targeted clinical strategies. Lastly, Bayesian sensitivity analysis reinforced the validity of our findings, as results remained consistent across modeling frameworks.

Nonetheless, several limitations merit discussion. First, the average trial duration of approximately 129.6 weeks (around 2.5 years) may be insufficient to fully capture the long-term risk of developing malignancy. Although the hematologic malignancy might have shorter period of latency (around 2 years) than solid tumors, the relatively shorter trial duration might still limit the property of hematologic malignancy detection [94]. Second, while our focus on RCTs enhances internal validity, it may exclude useful data from well-conducted observational studies. Although we recognized the fact that the numbers of included studies would matter a lot in clinical practice, we kept to only include RCTs due to avoidance of heterogeneity of study designation. Third, heterogeneity in diagnostic methodology across international trials could introduce variability in how hematologic cancers were identified, potentially affecting accuracy in pooled estimates. Fourth, given the small number of target events, the main results of this NMA should be interpreted with caution. Additionally, most included RCTs were not primarily designed to evaluate hematologic cancer outcomes, as they typically addressed glucose control in diabetes or weight reduction in obesity. Therefore, malignancy data may have been underreported or inconsistently monitored. Since adverse event reporting often receives less rigorous scrutiny than primary outcomes, there’s a possibility that hematologic events may have been missed or incompletely recorded. In addition, they were not initially designed to exclude the potential confounding factors, such as drug interactions, tolerance of these antidiabetics and further risk factors of these malignancies. Therefore, although the patient characteristic data of the current study fell within the hematologic malignancy range (i.e., age, gender distribution), we could not make further exploration regarding the confounding effects related to patient characteristics.

## 5. Conclusions

In summary, this pilot NMA provides preliminary evidence suggesting agent-specific differences in hematologic malignancy risk among GLP-1 receptor agonists. Specifically, dulaglutide was linked to a significantly higher incidence of overall hematologic malignancies, whereas tirzepatide demonstrated a notable risk-reducing effect—particularly for lymphoma. In contrast, no meaningful associations were identified for SGLT2 inhibitors regarding hematologic cancer risk. However, given the nature of the preliminary findings of this pilot NMA, we could only view the results as an exploratory finding but not final conclusion. Future research should prioritize large-scale RCTs directly addressing the risk of hematologic malignancy related to specific GLP-1 receptor agonists prescription. Further, to support or refute the results of the current NMA, large-scale and comprehensive NMA addressing various new generation antidiabetic agents should be warranted.

## Figures and Tables

**Table 1:** League table of the primary outcome: overall hematologic malignancy.

Tirzepatide		0.11 [0.00; 2.72]									* 0.19 [0.04; 0.90]	.	.	.	.	.	.	.	.	.	.	.	.
0.37 [0.05; 2.85]	Inject_ semaglutide_ low_dosage	0.95 [0.14; 6.52]									0.34 [0.07; 1.59]	.	.	.	.	.	.	.	.	.	.	.	.
0.33 [0.06; 1.85]	0.91 [0.16; 5.19]	Inject_ semaglutide_ medium_dosage									0.41 [0.10; 1.64]	0.20 [0.01; 4.17]	.	.	.	.	.	.	.	.	.	.	.
0.44 [0.03; 6.50]	1.21 [0.08; 18.32]	1.33 [0.10; 17.31]	Dapagliflozin_ medium_dosage								0.32 [0.03; 3.09]	.	.	.	.	.	.	.	.	.	.	.	.
0.43 [0.03; 6.35]	1.18 [0.08; 17.88]	1.30 [0.10; 16.90]	0.98 [0.04; 24.14]	Dapagliflozin_ low_dosage							0.33 [0.03; 3.16]	.	.	.	.	.	.	.	.	.	.	.	.
0.31 [0.05; 2.13]	0.86 [0.12; 6.08]	0.94 [0.17; 5.37]	0.71 [0.05; 9.50]	0.73 [0.05; 9.73]	Canagliflozin_ high_dosage		0.75 [0.17; 3.36]				0.56 [0.14; 2.17]	.	.	.	.	.	.	.	.	.	.	.	.
0.24 [0.04; 1.53]	0.65 [0.10; 4.39]	0.72 [0.13; 3.85]	0.54 [0.04; 6.94]	0.55 [0.04; 7.11]	0.76 [0.14; 4.27]	Lixisenatide			0.33 [0.01; 8.19]		0.67 [0.19; 2.36]	.	.	.	.	.	.	.	.	.	.	.	.
0.20 [0.03; 1.22]	0.55 [0.09; 3.50]	0.61 [0.12; 3.05]	0.46 [0.04; 5.63]	0.47 [0.04; 5.77]	0.64 [0.18; 2.35]	0.85 [0.17; 4.20]	Canagliflozin_ low_dosage				0.87 [0.28; 2.68]	.	.	.	.	.	.	.	.	.	.	.	.
0.19 [0.03; 1.22]	0.53 [0.08; 3.50]	0.58 [0.11; 3.07]	0.44 [0.03; 5.57]	0.45 [0.04; 5.70]	0.62 [0.11; 3.40]	0.81 [0.16; 4.23]	0.96 [0.20; 4.63]	Empagliflozin_ high_dosage			0.59 [0.15; 2.28]	.	.	.	.	.	.	.	.	.	0.37 [0.11; 1.33]	.	.
* 0.17 [0.04; 0.79]	0.45 [0.09; 2.28]	0.50 [0.13; 1.90]	0.38 [0.04; 3.92]	0.39 [0.04; 4.01]	0.53 [0.13; 2.13]	0.70 [0.19; 2.52]	0.82 [0.24; 2.81]	0.86 [0.24; 3.14]	Liraglutide		0.85 [0.47; 1.56]	.	0.33 [0.01; 8.08]	.	.	.	.	.	.	.	.	.	.
0.16 [0.02; 1.16]	0.44 [0.06; 3.30]	0.48 [0.08; 2.93]	0.36 [0.03; 5.09]	0.37 [0.03; 5.21]	0.51 [0.08; 3.24]	0.68 [0.11; 4.05]	0.80 [0.14; 4.46]	0.83 [0.14; 4.90]	0.97 [0.22; 4.19]	Oral_ semaglutide	0.88 [0.23; 3.36]	.	.	.	.	.	.	.	.	.	.	.	.
* 0.14 [0.03; 0.60]	0.39 [0.09; 1.73]	0.42 [0.13; 1.41]	0.32 [0.03; 3.09]	0.33 [0.03; 3.16]	0.45 [0.13; 1.59]	0.59 [0.18; 1.93]	0.70 [0.24; 2.05]	0.73 [0.23; 2.32]	0.85 [0.47; 1.52]	0.88 [0.23; 3.36]	Placebo_ or_Control	0.99 [0.56; 1.75]	0.95 [0.53; 1.71]	0.83 [0.25; 2.73]	0.87 [0.58; 1.30]	0.66 [0.03; 16.34]	0.81 [0.28; 2.35]	0.62 [0.21; 1.88]	0.33 [0.01; 8.20]	0.61 [0.22; 1.70]	* 0.52 [0.28; 0.99]	0.20 [0.01; 4.18]	* 0.46 [0.24; 0.88]
* 0.14 [0.03; 0.63]	0.37 [0.07; 1.83]	0.41 [0.11; 1.48]	0.31 [0.03; 3.18]	0.31 [0.03; 3.26]	0.43 [0.11; 1.72]	0.57 [0.15; 2.11]	0.67 [0.20; 2.27]	0.70 [0.19; 2.54]	0.82 [0.36; 1.84]	0.84 [0.20; 3.63]	0.96 [0.55; 1.69]	Inject_ semaglutide_ high_dosage	.	.	.	.	.	.	.	.	.	.	.
* 0.13 [0.03; 0.62]	0.36 [0.07; 1.78]	0.39 [0.10; 1.49]	0.30 [0.03; 3.07]	0.30 [0.03; 3.14]	0.42 [0.10; 1.67]	0.55 [0.15; 2.04]	0.65 [0.19; 2.20]	0.68 [0.19; 2.46]	0.79 [0.35; 1.76]	0.81 [0.19; 3.50]	0.93 [0.52; 1.65]	0.96 [0.43; 2.16]	Exenatide	.	.	.	.	.	.	.	.	.	.
* 0.12 [0.02; 0.73]	0.32 [0.05; 2.10]	0.36 [0.07; 1.83]	0.27 [0.02; 3.36]	0.28 [0.02; 3.44]	0.38 [0.07; 2.03]	0.50 [0.10; 2.53]	0.59 [0.12; 2.77]	0.61 [0.12; 3.06]	0.71 [0.20; 2.51]	0.74 [0.13; 4.22]	0.84 [0.28; 2.56]	0.87 [0.25; 3.05]	0.91 [0.26; 3.18]	Ertugliflozin_ low_dosage	.	.	.	.	.	0.71 [0.27; 1.91]	.	.	.
* 0.12 [0.03; 0.55]	0.34 [0.07; 1.59]	0.37 [0.10; 1.31]	0.28 [0.03; 2.78]	0.28 [0.03; 2.85]	0.39 [0.10; 1.47]	0.52 [0.15; 1.80]	0.61 [0.19; 1.92]	0.64 [0.19; 2.16]	0.74 [0.36; 1.50]	0.76 [0.19; 3.10]	0.87 [0.58; 1.30]	0.90 [0.45; 1.81]	0.94 [0.47; 1.90]	1.04 [0.32; 3.38]	Dapagliflozin_ high_dosage	.	.	.	.	.	.	.	.
0.09 [0.00; 3.15]	0.26 [0.01; 8.80]	0.28 [0.01; 8.63]	0.21 [0.00; 10.74]	0.22 [0.00; 11.01]	0.30 [0.01; 9.35]	0.39 [0.01; 11.97]	0.46 [0.02; 13.62]	0.49 [0.02; 14.62]	0.56 [0.02; 14.63]	0.58 [0.02; 18.78]	0.66 [0.03; 16.34]	0.69 [0.03; 17.85]	0.72 [0.03; 18.59]	0.79 [0.03; 23.46]	0.76 [0.03; 19.25]	Bexagliflozin	.	.	.	.	.	.	.
* 0.12 [0.02; 0.69]	0.31 [0.05; 1.97]	0.35 [0.07; 1.71]	0.26 [0.02; 3.18]	0.27 [0.02; 3.26]	0.37 [0.07; 1.90]	0.48 [0.10; 2.36]	0.57 [0.13; 2.58]	0.59 [0.12; 2.85]	0.69 [0.21; 2.32]	0.71 [0.13; 3.95]	0.81 [0.28; 2.35]	0.85 [0.25; 2.81]	0.88 [0.26; 2.94]	0.97 [0.21; 4.50]	0.93 [0.30; 2.91]	1.22 [0.04; 35.71]	Sotagliflozin	.	.	.	.	.	.
* 0.09 [0.01; 0.54]	0.24 [0.04; 1.55]	0.27 [0.05; 1.35]	0.20 [0.02; 2.48]	0.20 [0.02; 2.54]	0.28 [0.05; 1.50]	0.37 [0.07; 1.86]	0.44 [0.09; 2.04]	0.46 [0.09; 2.25]	0.53 [0.15; 1.85]	0.55 [0.10; 3.11]	0.62 [0.21; 1.88]	0.65 [0.19; 2.24]	0.67 [0.19; 2.34]	0.74 [0.15; 3.56]	0.72 [0.22; 2.32]	0.94 [0.03; 27.76]	0.77 [0.17; 3.55]	Albiglutide	.	.	.	.	.
0.05 [0.00; 1.58]	0.13 [0.00; 4.42]	0.14 [0.00; 4.33]	0.11 [0.00; 5.39]	0.11 [0.00; 5.52]	0.15 [0.00; 4.69]	0.20 [0.01; 6.01]	0.23 [0.01; 6.84]	0.24 [0.01; 7.34]	0.28 [0.01; 7.34]	0.29 [0.01; 9.43]	0.33 [0.01; 8.20]	0.35 [0.01; 8.96]	0.36 [0.01; 9.33]	0.40 [0.01; 11.77]	0.38 [0.02; 9.66]	0.50 [0.01; 46.50]	0.41 [0.01; 11.97]	0.53 [0.02; 15.80]	Efpeglenatide_ high_dosage	.	.	0.60 [0.08; 4.55]	.
* 0.08 [0.01; 0.47]	0.22 [0.04; 1.35]	0.24 [0.05; 1.17]	0.18 [0.02; 2.19]	0.19 [0.02; 2.24]	0.26 [0.05; 1.30]	0.34 [0.07; 1.61]	0.40 [0.09; 1.75]	0.42 [0.09; 1.94]	0.49 [0.15; 1.56]	0.50 [0.09; 2.70]	0.57 [0.21; 1.57]	0.60 [0.19; 1.89]	0.62 [0.19; 1.98]	0.68 [0.26; 1.79]	0.66 [0.22; 1.95]	0.86 [0.03; 24.76]	0.70 [0.16; 3.05]	0.92 [0.21; 4.09]	1.72 [0.06; 49.25]	Ertugliflozin_ high_dosage	.	.	.
* 0.08 [0.02; 0.36]	0.21 [0.04; 1.05]	* 0.23 [0.06; 0.88]	0.17 [0.02; 1.79]	0.17 [0.02; 1.84]	* 0.24 [0.06; 0.98]	0.32 [0.08; 1.20]	0.37 [0.11; 1.30]	0.39 [0.13; 1.19]	0.45 [0.19; 1.07]	0.47 [0.11; 2.06]	0.53 [0.29; 1.00]	0.55 [0.24; 1.29]	0.58 [0.25; 1.35]	0.64 [0.18; 2.28]	0.61 [0.29; 1.29]	0.80 [0.03; 20.98]	0.66 [0.19; 2.25]	0.86 [0.24; 3.04]	1.60 [0.06; 41.73]	0.93 [0.28; 3.05]	Empagliflozin_ low_dosage	.	.
* 0.03 [0.00; 0.82]	0.08 [0.00; 2.29]	0.09 [0.00; 2.23]	0.06 [0.00; 2.84]	0.07 [0.00; 2.90]	0.09 [0.00; 2.42]	0.12 [0.00; 3.09]	0.14 [0.01; 3.52]	0.15 [0.01; 3.78]	0.17 [0.01; 3.75]	0.18 [0.01; 4.87]	0.20 [0.01; 4.18]	0.21 [0.01; 4.58]	0.22 [0.01; 4.77]	0.24 [0.01; 6.06]	0.23 [0.01; 4.93]	0.30 [0.00; 24.90]	0.25 [0.01; 6.15]	0.32 [0.01; 8.13]	0.60 [0.08; 4.55]	0.35 [0.01; 8.59]	0.38 [0.02; 8.34]	Efpeglenatide_ medium_dosage	.
* 0.07 [0.01; 0.31]	* 0.18 [0.03; 0.91]	* 0.19 [0.05; 0.76]	0.15 [0.01; 1.55]	0.15 [0.01; 1.59]	* 0.21 [0.05; 0.85]	0.27 [0.07; 1.05]	0.32 [0.09; 1.13]	0.34 [0.09; 1.26]	* 0.39 [0.16; 0.94]	0.40 [0.09; 1.79]	* 0.46 [0.24; 0.88]	0.48 [0.20; 1.13]	0.50 [0.21; 1.18]	0.55 [0.15; 1.98]	0.53 [0.25; 1.13]	0.69 [0.03; 18.12]	0.56 [0.16; 1.96]	0.73 [0.20; 2.65]	1.37 [0.05; 36.05]	0.80 [0.24; 2.66]	0.86 [0.35; 2.12]	2.29 [0.10; 51.12]	Dulaglutide

Gray background represent the indicated medicatoin. Data present as OR [95%CIs]. Pairwise (upper-right portion) and network (lower-left portion) meta-analysis results are presented as estimate effect sizes for the outcome of overall events of hematologic malignancy. Interventions are reported in order of mean ranking of beneficially prophylactic effect on overall events of hematologic malignancy, and outcomes are expressed as odds ratio (OR) (95% confidence intervals) (95%CIs). For the pairwise meta-analyses, OR of less than 1 indicate that the treatment specified in the row got more beneficial effect than that specified in the column. For the network meta-analysis (NMA), OR of less than 1 indicate that the treatment specified in the column got more beneficial effect than that specified in the row. Bold results marked with * indicate statistical significance. Abbreviation: 95%CIs: 95% confidence intervals; GLP-1 agonist: glucagon-like peptide-1 agonist; NMA: network meta-analysis; OR: odds ratio; RCT: randomized controlled trial; SGLT2 inhibitor: sodium–glucose cotransporter 2 inhibitor.

## Data Availability

No new data were created or analyzed in this study.

## Supplementary Materials

The following supporting information can be downloaded at: https://www.mdpi.com/article/10.3390/biom15111622/s1, Figure S1: Network structure of NMA of primary outcome and safety profile; Figure S2: Forest plot of NMA of primary outcome and safety profile; Figure S3: Individual study result of primary outcome and safety profile; Figure S4: Bayesian-based forest plot of NMA of primary outcome and safety profile; Figure S5: Bayesian-based Litmus Rank-O-Gram rank plot of primary outcome; Figure S6: Bayesian-based residual deviance NMA/UME model of primary outcome; Figure S7: Overview of risk of bias; Table S1: PRISMA 2020 checklist of the current network meta-analysis; Table S2: Keyword used in each database and search results; Table S3: Excluded studies and reason; Table S4: Characteristics of the included studies; Table S5: League table of NMA of primary outcome and safety profile; Table S6: SUCRA (Surface under the cumulative ranking) of primary outcome and safety profile; Table S7: Inconsistency within the network meta-analysis of primary outcome and safety profile; Table S8: GRADE of primary outcome and safety profile.
