# Supplementary material for: Divergent Risks of Hematologic Malignancies Associated with GLP-1 Receptor Agonists and SGLT2 Inhibitors: Preliminary Findings from a Pilot Network Meta-Analysis
Source: Biomolecules. 2025 Nov 19;15(11):1622. doi: 10.3390/biom15111622 (PMC12650725; doi:10.3390/biom15111622)
Supplement: Supplementary file 1 [file biomolecules-15-01622-s001.zip › biomolecules-3897098-supplementary.pdf]

**List of content in supplement materials**

# Divergent Risks of Hematologic Malignancies Associated with GLP-1 Receptor Agonists and SGLT2 Inhibitors

## A Network Meta-Analysis of 55 Randomized Trials

*Pao-Yen Lin, et al.*


| | |
|---|---|
| Figure S1 | (A) Network structure of NMA of primary outcome: subgroup of lymphoma |
| | (B) Network structure of NMA of primary outcome: subgroup of leukemia |
| | (C) Network structure of NMA of primary outcome: subgroup of myeloma |
| | (D) Network structure of NMA of safety profile: drop-out rate |
| Figure S2 | (A) Forest plot of NMA of primary outcome: subgroup of lymphoma |
| | (B) Forest plot of NMA of primary outcome: subgroup of leukemia |
| | (C) Forest plot of NMA of primary outcome: subgroup of myeloma |
| | (D) Forest plot of NMA of safety profile: drop-out rate |
| Figure S3 | (A) Individual study result of primary outcome: overall hematologic malignancy |
| | (B) Individual study result of primary outcome: subgroup of lymphoma |
| | (C) Individual study result of primary outcome: subgroup of leukemia |
| | (D) Individual study result of primary outcome: subgroup of myeloma |
| | (E) Individual study result of safety profile: drop-out rate |
| Figure S4 | (A) Bayesian-based forest plot of NMA of primary outcome: overall hematologic malignancy |
| | (B) Bayesian-based forest plot of NMA of primary outcome: subgroup of lymphoma |
| | (C) Bayesian-based forest plot of NMA of primary outcome: subgroup of leukemia |
| | (D) Bayesian-based forest plot of NMA of primary outcome: subgroup of myeloma |
| | (E) Bayesian-based forest plot of NMA of safety profile: drop-out rate |
| Figure S5 | (A) Bayesian-based Litmus Rank-O-Gram rank plot of primary outcome: overall hematologic malignancy |
| | (B) Bayesian-based radial surface under the cumulative ranking of primary outcome: overall hematologic malignancy |
| | (C) Bayesian-based Litmus Rank-O-Gram rank plot of primary outcome: subgroup of lymphoma |
| | (D) Bayesian-based radial surface under the cumulative ranking of primary outcome: subgroup of lymphoma |
| | (E) Bayesian-based Litmus Rank-O-Gram rank plot of primary outcome: subgroup of leukemia |

| | |
|---|---|
| | (F) Bayesian-based radial surface under the cumulative ranking of primary outcome: subgroup of leukemia |
| | (G) Bayesian-based Litmus Rank-O-Gram rank plot of primary outcome: subgroup of myeloma |
| | (H) Bayesian-based radial surface under the cumulative ranking of primary outcome: subgroup of myeloma |
| | (I) Bayesian-based Litmus Rank-O-Gram rank plot of safety profile: drop-out rate |
| | (J) Bayesian-based radial surface under the cumulative ranking of safety profile: drop-out rate |
| Figure S6 | (A) Bayesian-based residual deviance NMA/UME model of primary outcome: overall hematologic malignancy |
| | (B) Bayesian-based per-arm residual deviance of primary outcome: overall hematologic malignancy |
| | (C) Bayesian-based leverage plot of primary outcome: overall hematologic malignancy |
| | (D) Bayesian-based residual deviance NMA/UME model of primary outcome: subgroup of lymphoma |
| | (E) Bayesian-based per-arm residual deviance of primary outcome: subgroup of lymphoma |
| | (F) Bayesian-based leverage plot of primary outcome: subgroup of lymphoma |
| | (G) Bayesian-based residual deviance NMA/UME model of primary outcome: subgroup of leukemia |
| | (H) Bayesian-based per-arm residual deviance of primary outcome: subgroup of leukemia |
| | (I) Bayesian-based leverage plot of primary outcome: subgroup of leukemia |
| | (J) Bayesian-based residual deviance NMA/UME model of primary outcome: subgroup of myeloma |
| | (K) Bayesian-based per-arm residual deviance of primary outcome: subgroup of myeloma |
| | (L) Bayesian-based leverage plot of primary outcome: subgroup of myeloma |
| | (M) Bayesian-based residual deviance NMA/UME model of safety profile: drop-out rate |
| | (N) Bayesian-based per-arm residual deviance of safety profile: drop-out rate |
| | (O) Bayesian-based leverage plot of safety profile: drop-out rate |
| Figure S7 | (A) Overview of risk of bias |
| | (B) Detailed risk of bias in each study |
| Table S1 | PRISMA 2020 checklist of the current network meta-analysis |
| Table S2 | Keyword used in each database and search results |
| Table S3 | Excluded studies and reason |
| Table S4 | Characteristics of the included studies |
| Table S5 | (A): League table of NMA of primary outcome: subgroup of lymphoma |
| | (B): League table of NMA of primary outcome: subgroup of leukemia |
| | (C): League table of NMA of primary outcome: subgroup of myeloma |
| | (D): League table of NMA of safety profile: drop-out rate |
| Table S6 | (A) SUCRA (Surface under the cumulative ranking) of primary outcome: overall hematologic malignancy |
| | (B) SUCRA (Surface under the cumulative ranking) of primary outcome: subgroup of lymphoma |

| | |
| --- | --- |
| | (C) SUCRA (Surface under the cumulative ranking) of primary outcome: subgroup of leukemia |
| | (D) SUCRA (Surface under the cumulative ranking) of primary outcome: subgroup of myeloma |
| | (E) SUCRA (Surface under the cumulative ranking) of safety profile: drop-out rate |
| Table S7 | (A) Inconsistency within the network meta-analysis of primary outcome: overall hematologic malignancy |
| | (B) Inconsistency within the network meta-analysis of primary outcome: subgroup of lymphoma |
| | (C) Inconsistency within the network meta-analysis of primary outcome: subgroup of leukemia |
| | (D) Inconsistency within the network meta-analysis of primary outcome: subgroup of myeloma |
| | (E) Inconsistency within the network meta-analysis of safety profile: drop-out rate |
| Table S8 | (A) GRADE of primary outcome: overall hematologic malignancy |
| | (B) GRADE of primary outcome: subgroup of lymphoma |
| | (C) GRADE of primary outcome: subgroup of leukemia |
| | (D) GRADE of primary outcome: subgroup of myeloma |
| | (E) GRADE of safety profile: drop-out rate |

**Figure S1A network structure of NMA of primary outcome: subgroup of lymphoma**

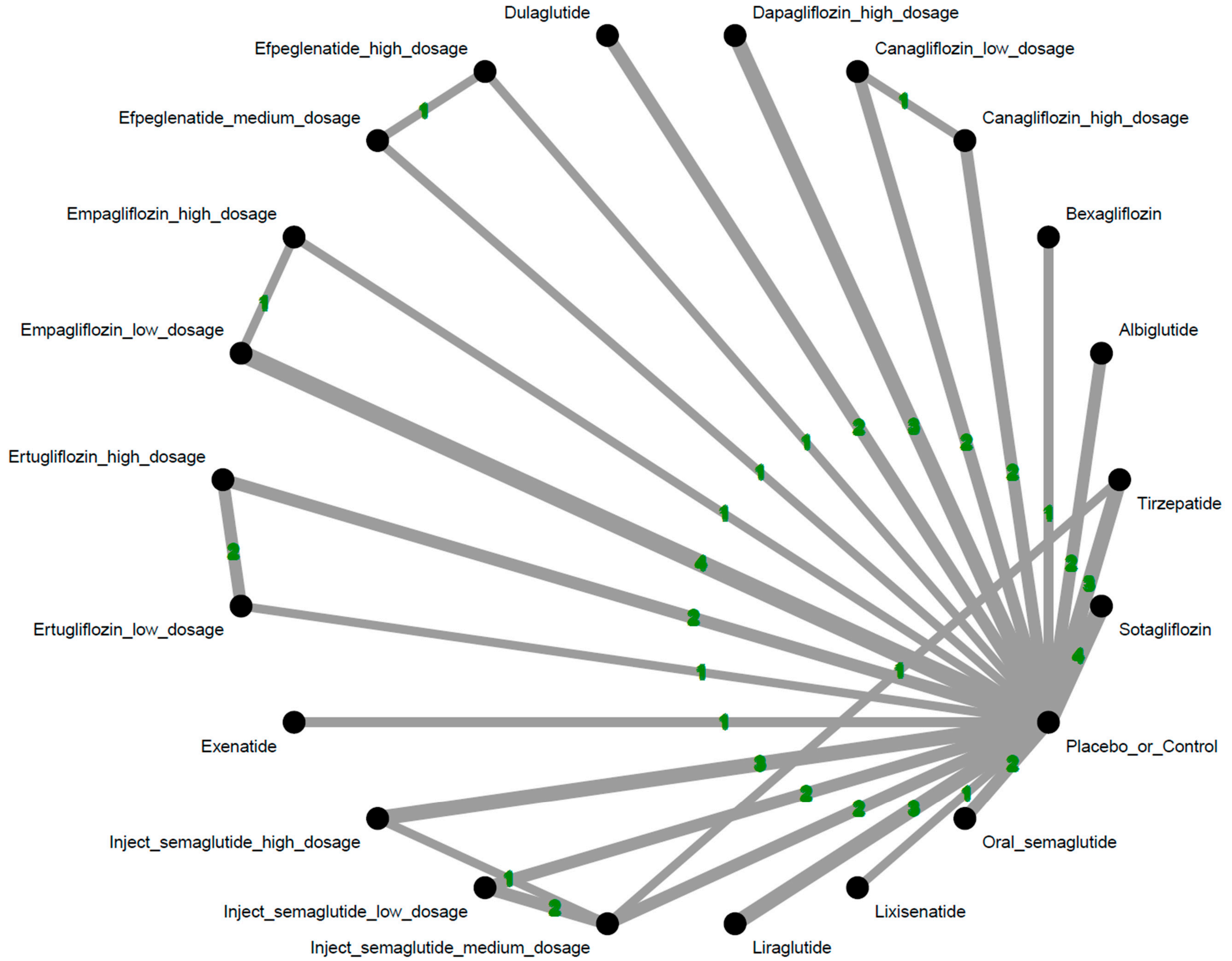

**Figure S1B network structure of NMA of primary outcome: subgroup of leukemia**

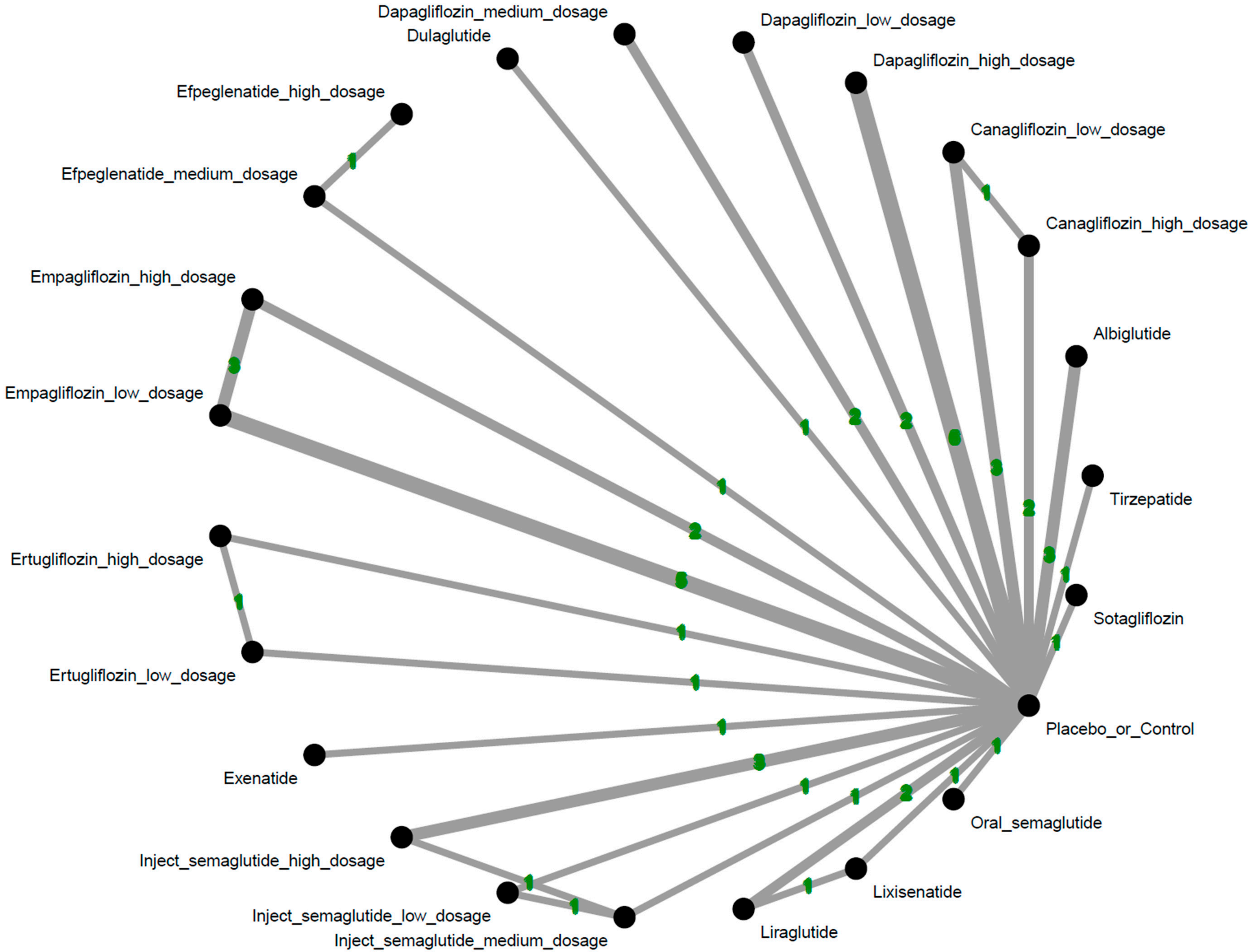

**Figure S1C network structure of NMA of primary outcome: subgroup of myeloma**

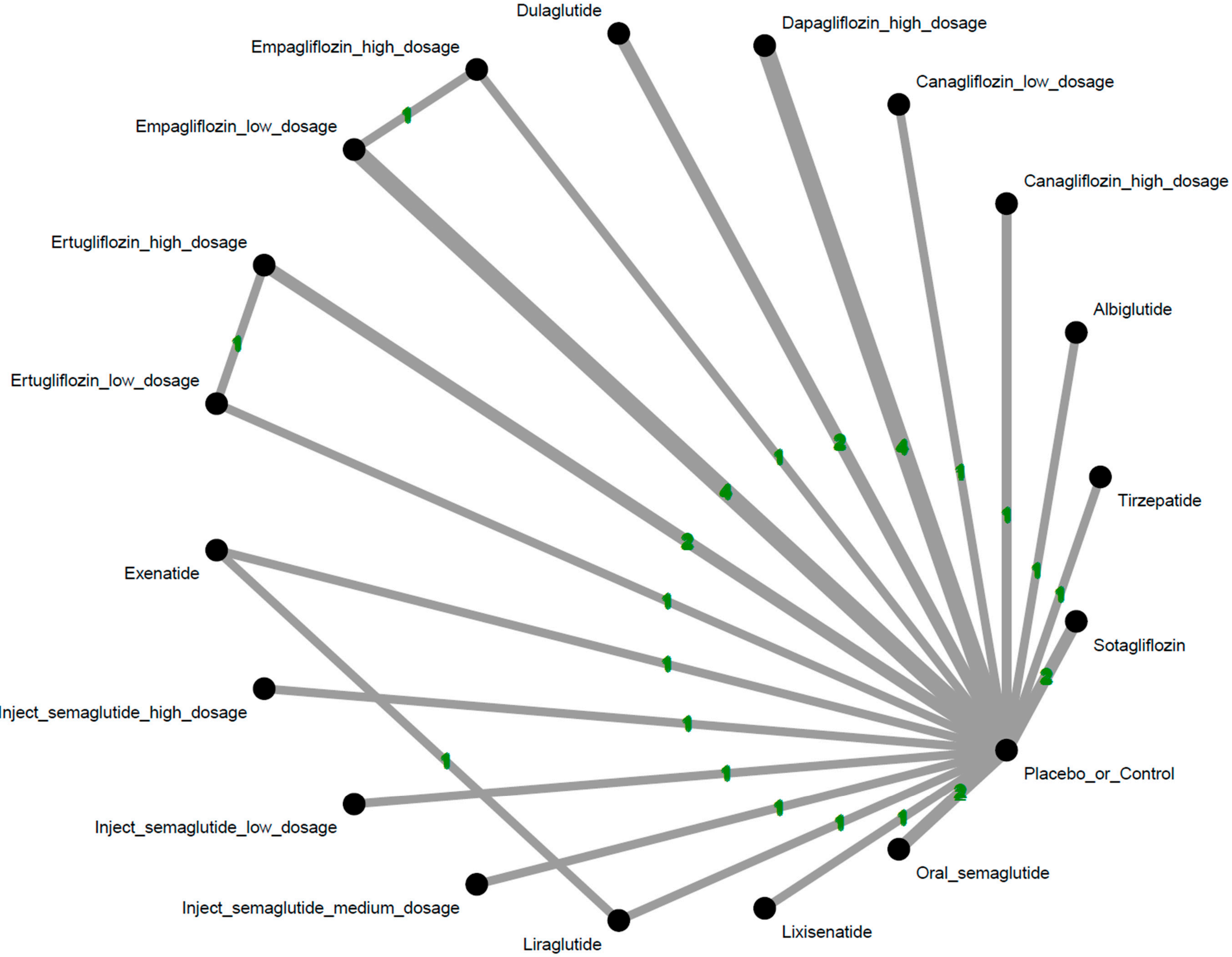

**Figure S1D network structure of NMA of safety profile: drop-out rate**

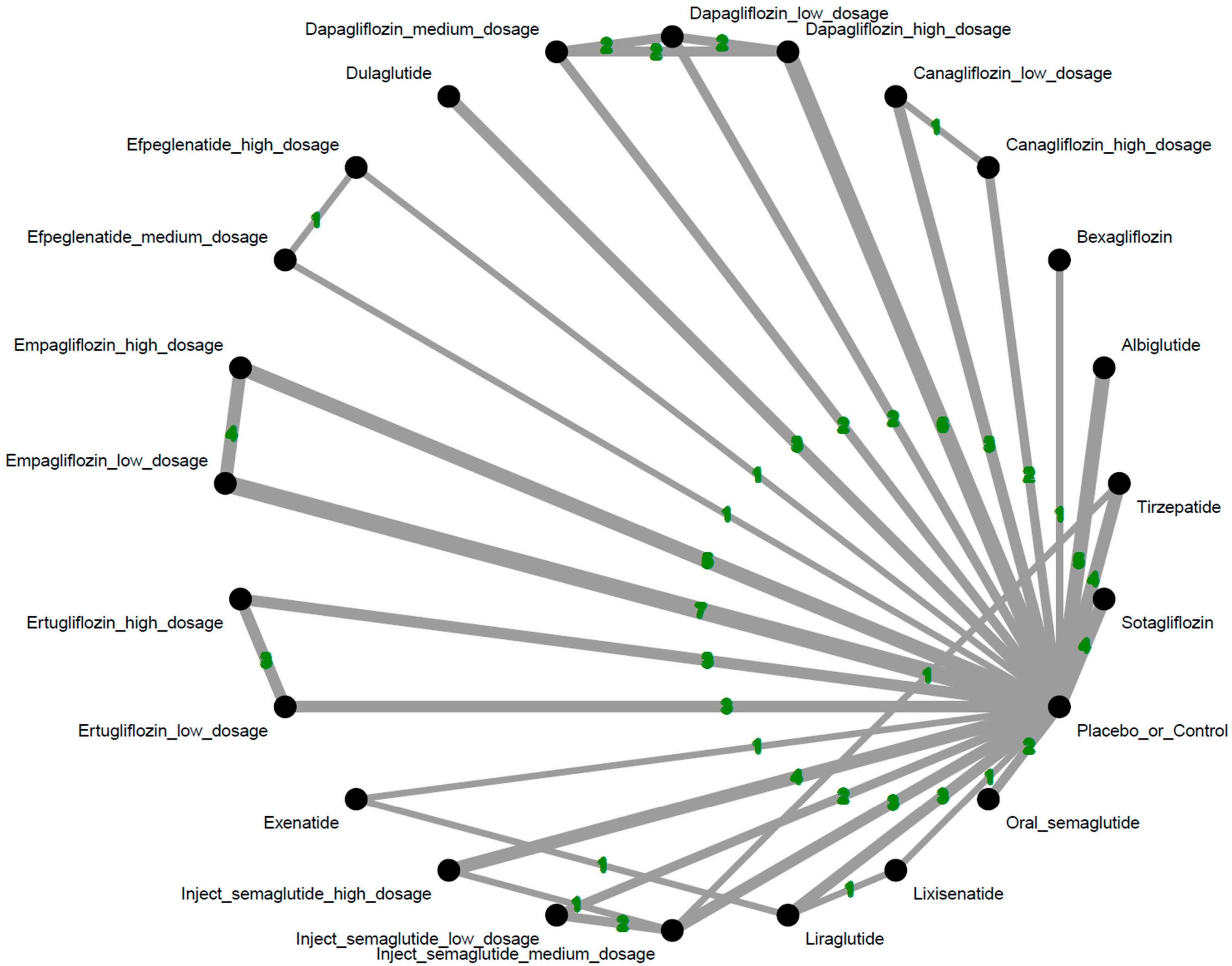

## Figure legend of Figure S1A-D

The structure of the network meta-analysis. The lines between nodes represent direct comparisons from various trials, with the numbers over the lines indicating the number of trials providing these comparisons for each specific treatment. The thickness of the lines corresponds to the number of trials linked to the network.

## *Abbreviation for Figure S1A-D:*

*95%CIs: 95% confidence intervals; GLP-1 agonist: glucagon-like peptide-1 agonist; NMA: network meta-analysis; OR: odds ratio; RCT: randomized controlled trial; SGLT2 inhibitor: sodium–glucose cotransporter 2 inhibitor*

**Figure S2A forest plot of NMA of primary outcome: subgroup of lymphoma**

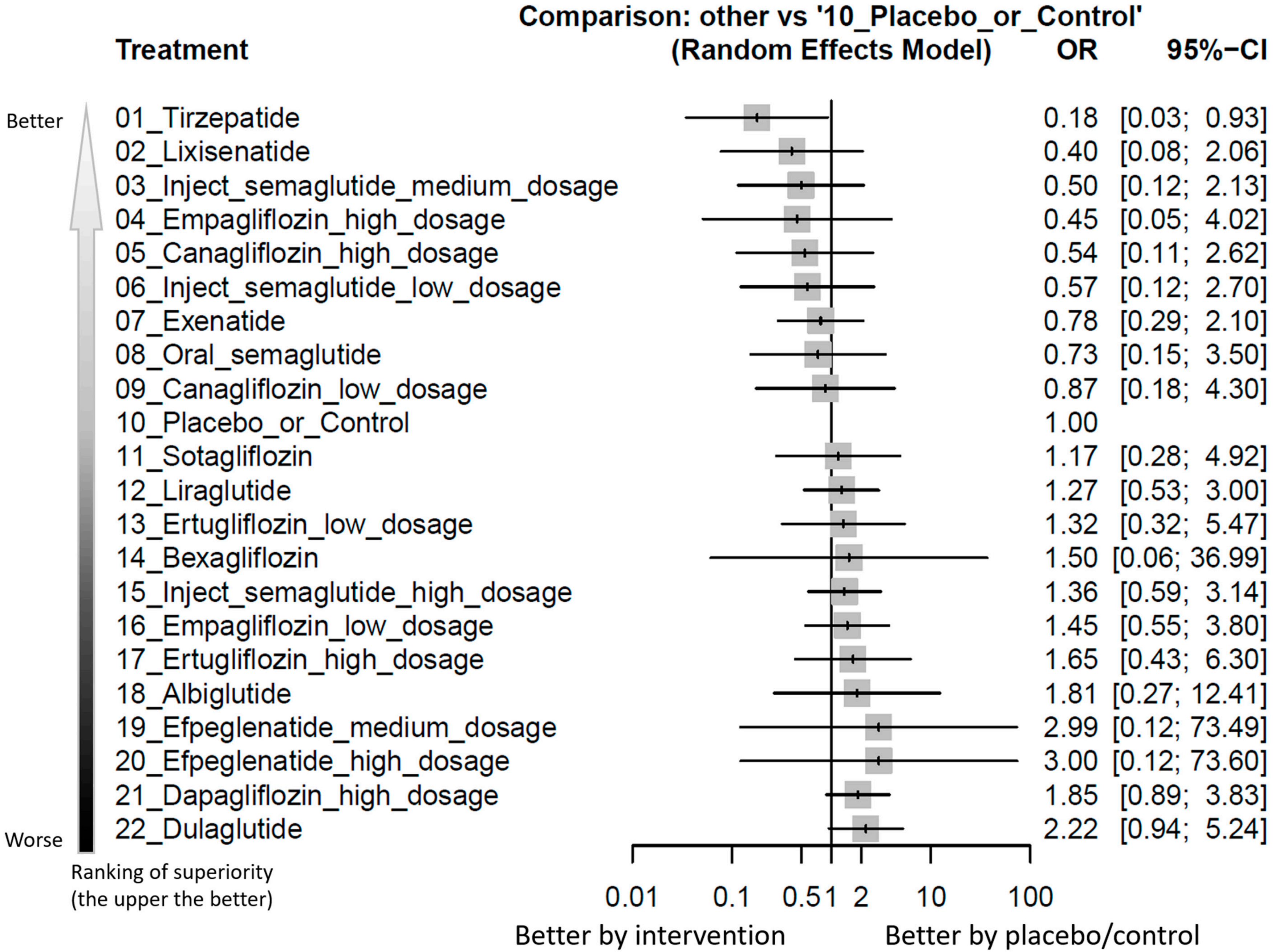

**Figure S2B forest plot of NMA of primary outcome: subgroup of leukemia**

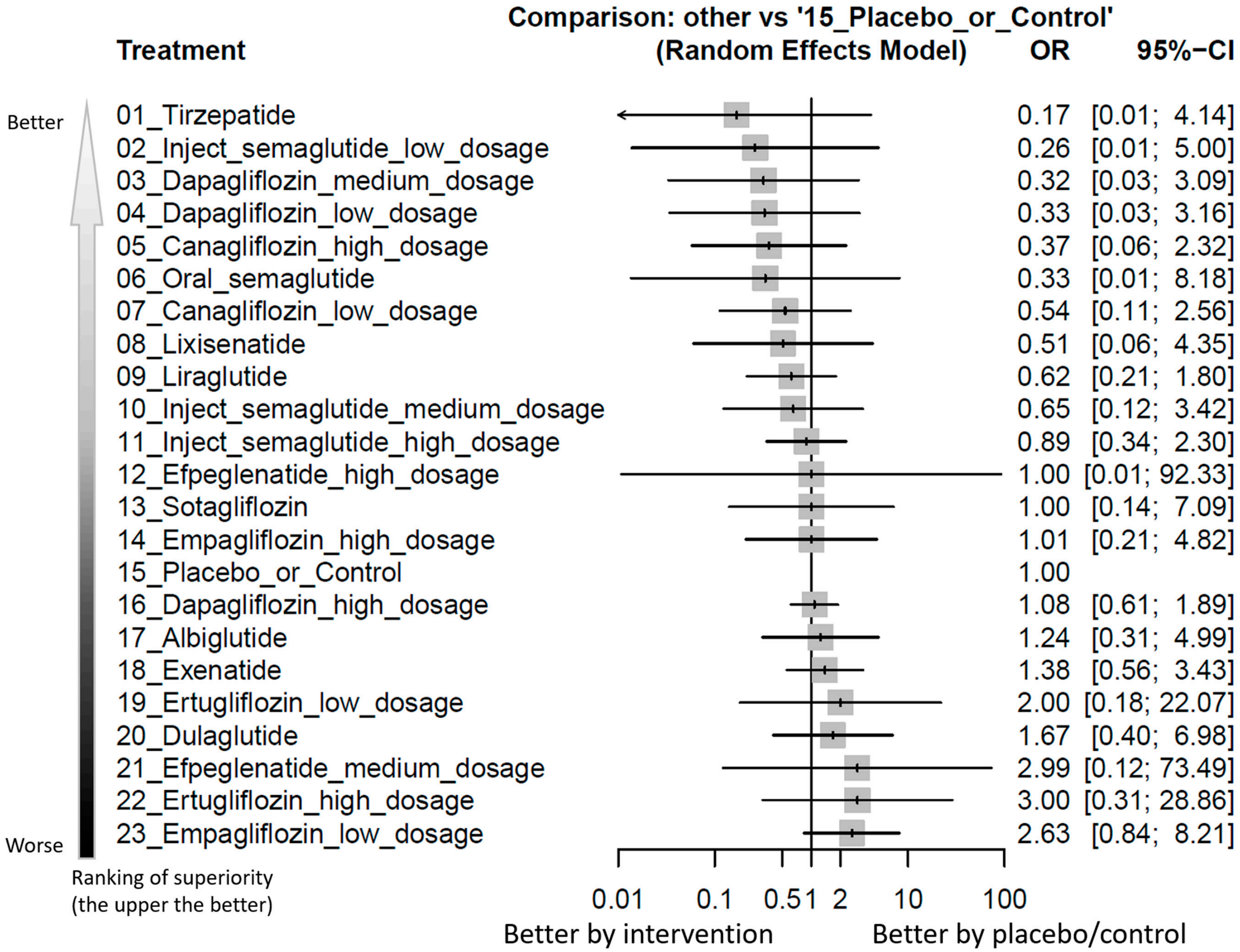

**Figure S2C forest plot of NMA of primary outcome: subgroup of myeloma**

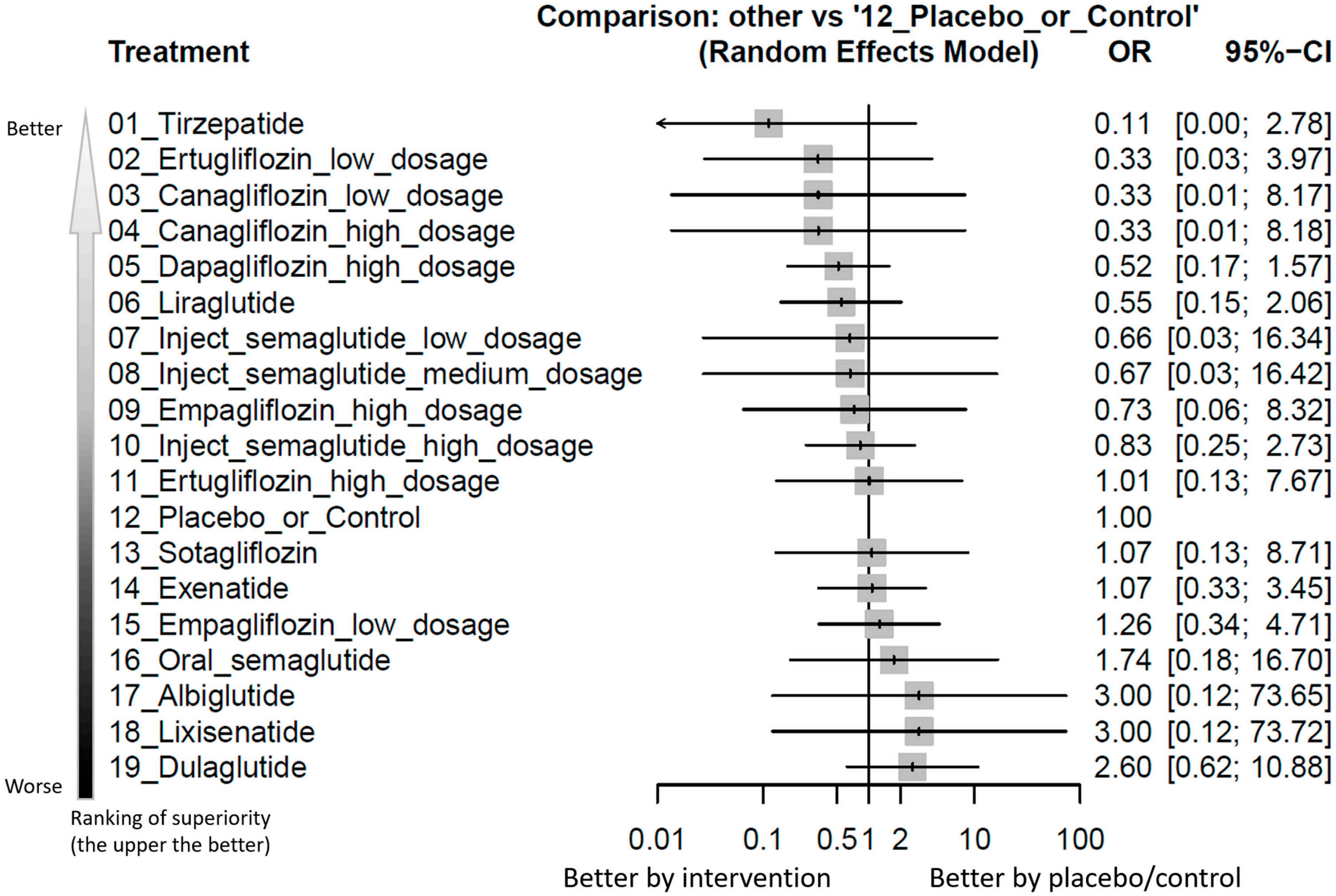

**Figure S2D forest plot of NMA of safety profile: drop-out rate**

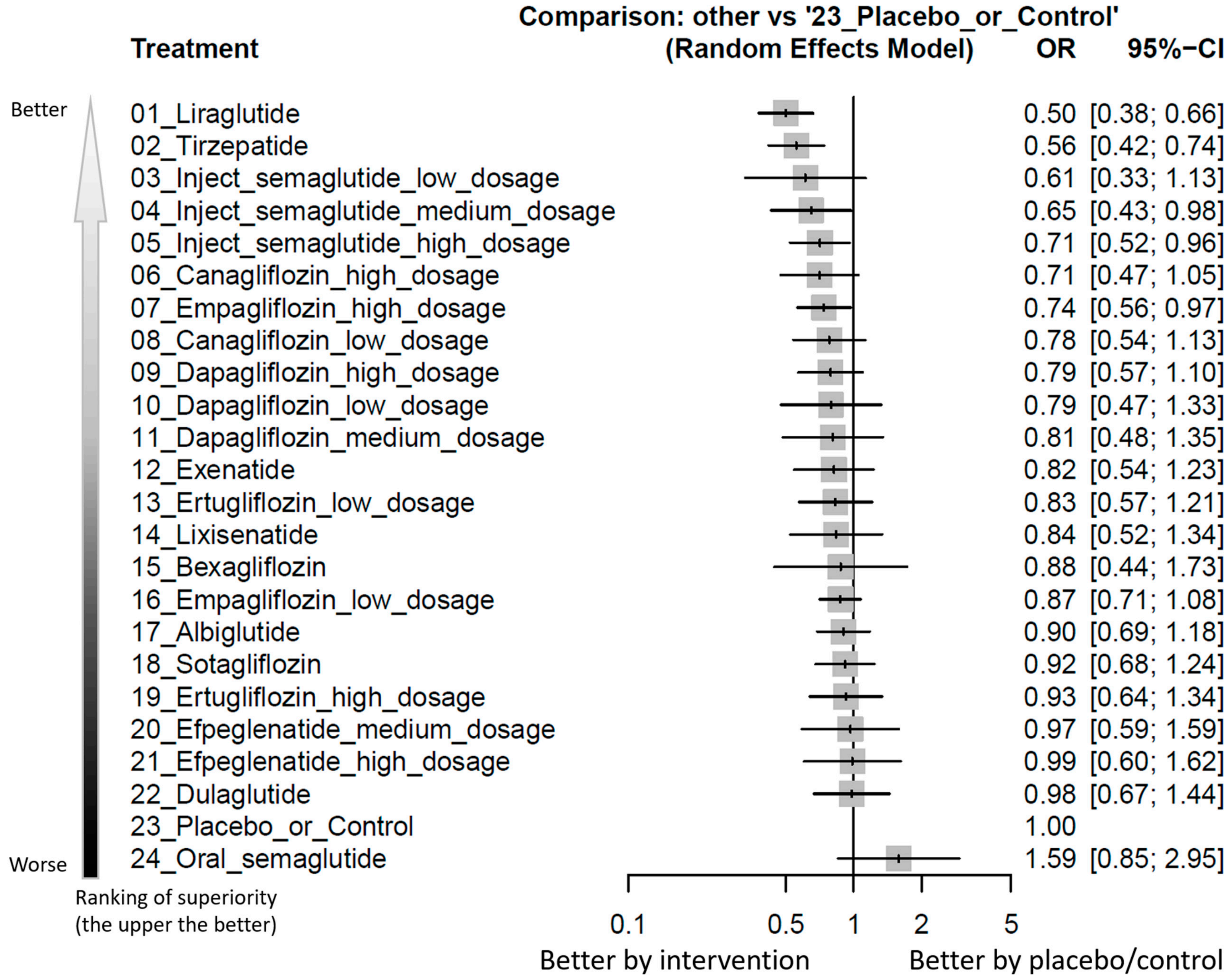

## ***Abbreviation for Figure S2A-D:***

*95%CIs: 95% confidence intervals; GLP-1 agonist: glucagon-like peptide-1 agonist; NMA: network meta-analysis; OR: odds ratio; RCT: randomized controlled trial; SGLT2 inhibitor: sodium–glucose cotransporter 2 inhibitor*

**Figure S3A Individual study result of primary outcome: overall hematologic malignancy**

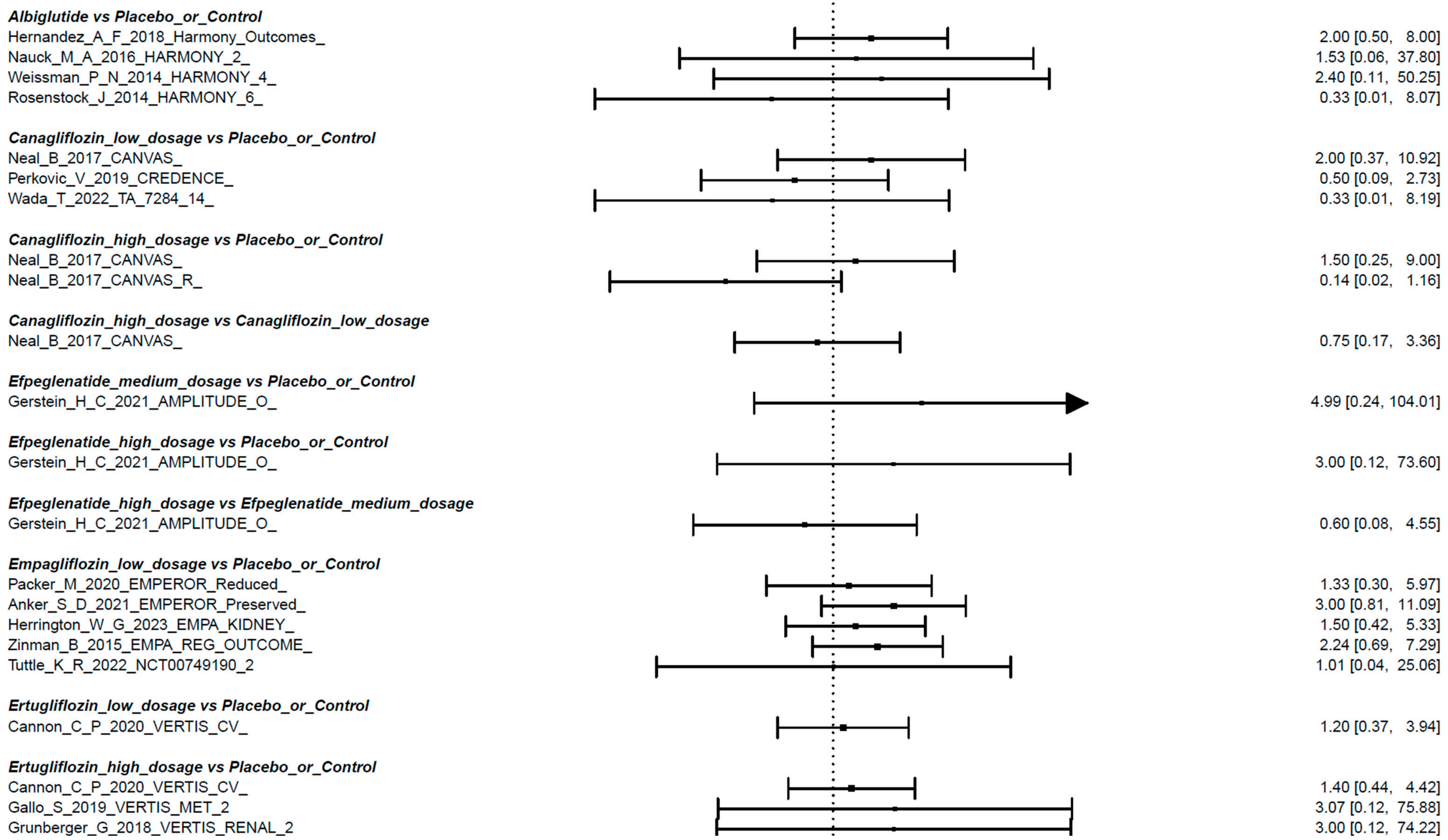

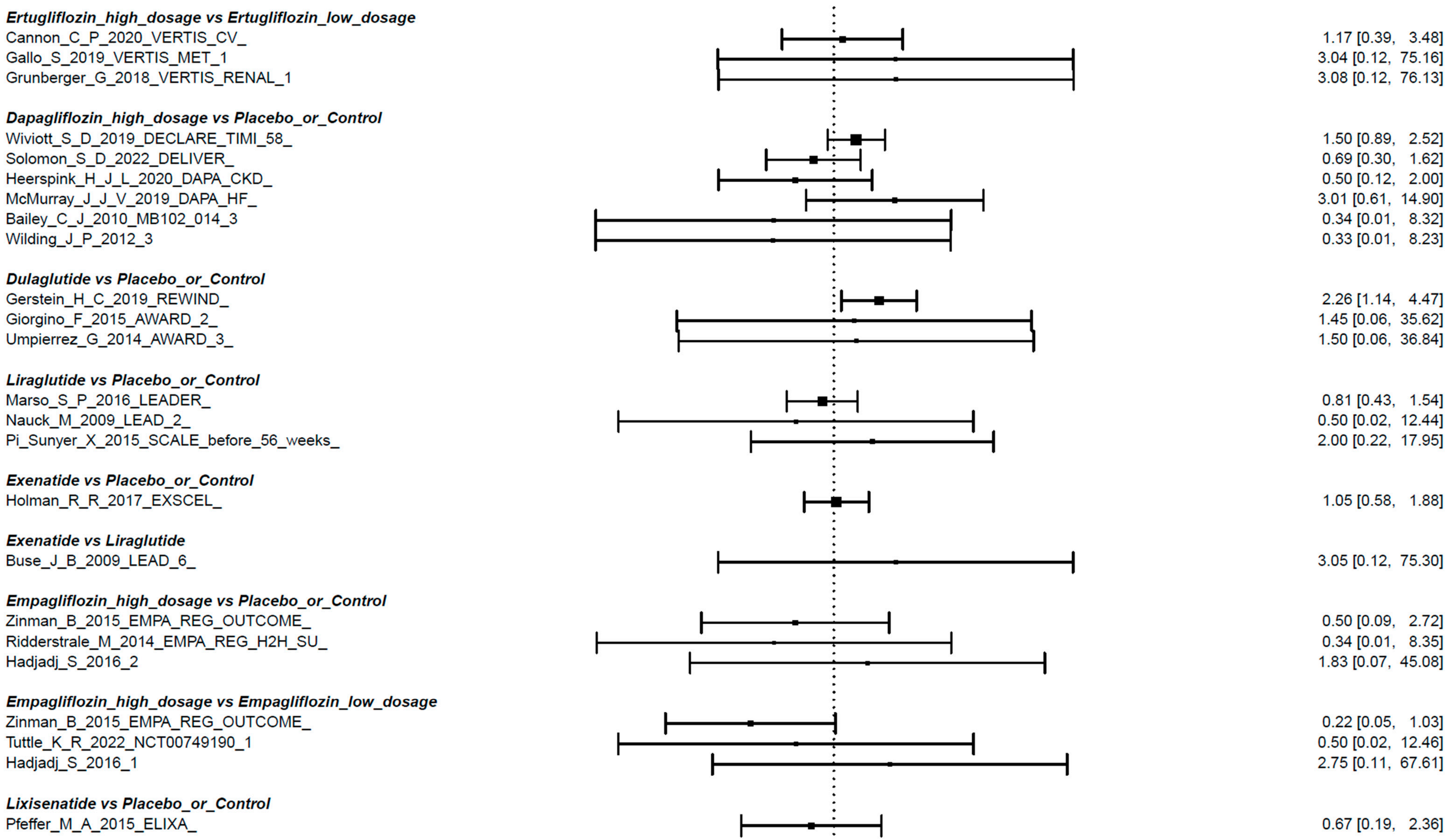
Ertugliflozin_high_dosage vs Ertugliflozin_low_dosage
Cannon_C_P_2020_VERTIS_CV_ 1.17 [0.39, 3.48]
Gallo_S_2019_VERTIS_MET_1 3.04 [0.12, 75.16]
Grunberger_G_2018_VERTIS_RENAL_1 3.08 [0.12, 76.13]
Dapagliflozin_high_dosage vs Placebo_or_Control
Wiviott_S_D_2019_DECLARE_TIMI_58_ 1.50 [0.89, 2.52]
Solomon_S_D_2022_DELIVER_ 0.69 [0.30, 1.62]
Heerspink_H_J_L_2020_DAPA_CKD_ 0.50 [0.12, 2.00]
McMurray_J_J_V_2019_DAPA_HF_ 3.01 [0.61, 14.90]
Bailey_C_J_2010_MB102_014_3 0.34 [0.01, 8.32]
Wilding_J_P_2012_3 0.33 [0.01, 8.23]
Dulaglutide vs Placebo_or_Control
Gerstein_H_C_2019_REWIND_ 2.26 [1.14, 4.47]
Giorgino_F_2015_AWARD_2_ 1.45 [0.06, 35.62]
Umpierrez_G_2014_AWARD_3_ 1.50 [0.06, 36.84]
Liraglutide vs Placebo_or_Control
Marso_S_P_2016_LEADER_ 0.81 [0.43, 1.54]
Nauck_M_2009_LEAD_2_ 0.50 [0.02, 12.44]
Pi_Sunyer_X_2015_SCALE_before_56_weeks_ 2.00 [0.22, 17.95]
Exenatide vs Placebo_or_Control
Holman_R_R_2017_EXSCEL_ 1.05 [0.58, 1.88]
Exenatide vs Liraglutide
Buse_J_B_2009_LEAD_6_ 3.05 [0.12, 75.30]
Empagliflozin_high_dosage vs Placebo_or_Control
Zinman_B_2015_EMPA_REG_OUTCOME_ 0.50 [0.09, 2.72]
Ridderstrale_M_2014_EMPA_REG_H2H_SU_ 0.34 [0.01, 8.35]
Hadjadj_S_2016_2 1.83 [0.07, 45.08]
Empagliflozin_high_dosage vs Empagliflozin_low_dosage
Zinman_B_2015_EMPA_REG_OUTCOME_ 0.22 [0.05, 1.03]
Tuttle_K_R_2022_NCT00749190_1 0.50 [0.02, 12.46]
Hadjadj_S_2016_1 2.75 [0.11, 67.61]
Lixisenatide vs Placebo_or_Control
Pfeffer_M_A_2015_ELIXA_ 0.67 [0.19, 2.36]

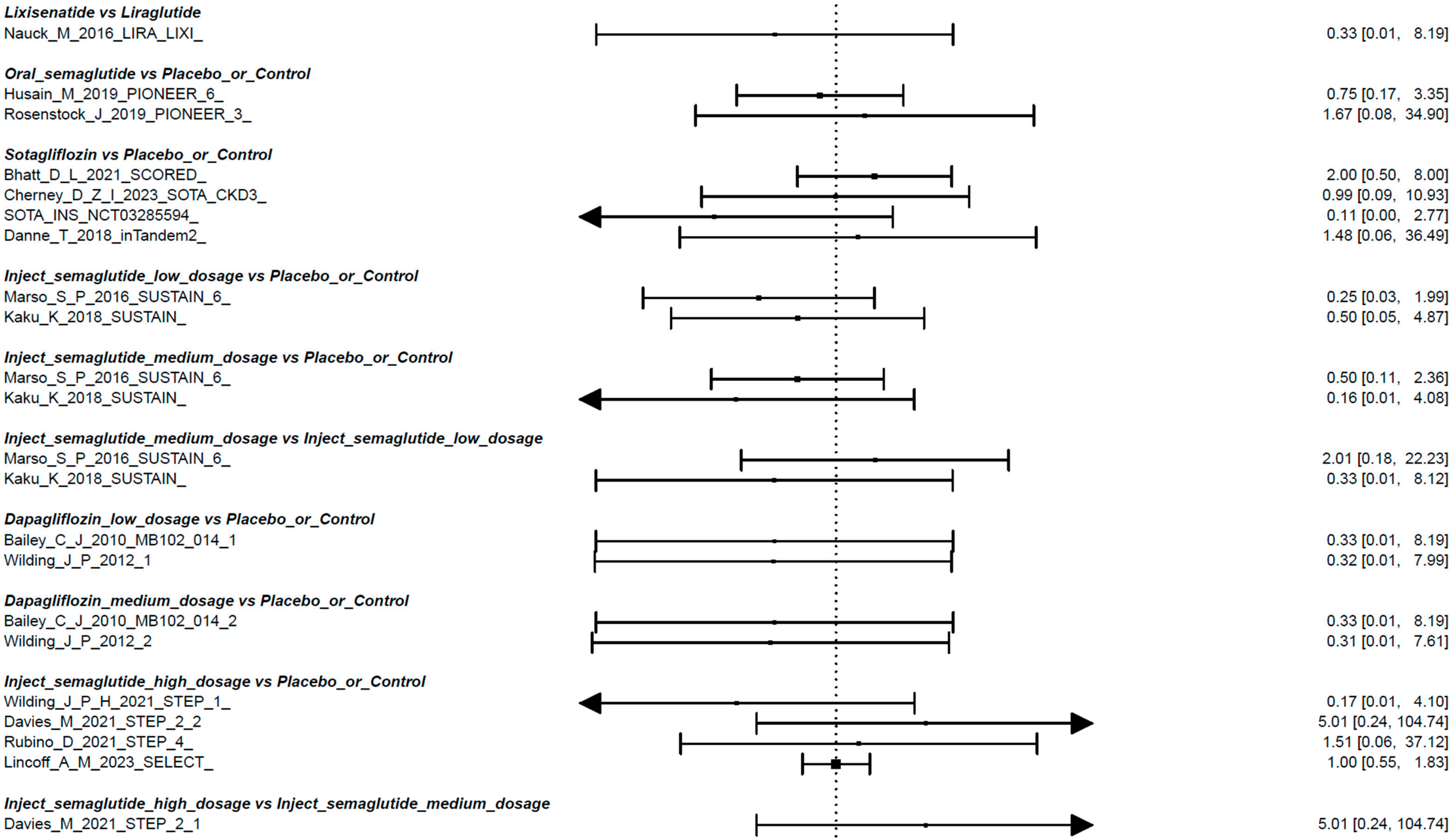
Lixisenatide vs Liraglutide
Nauck_M_2016_LIRA_LIXI_ 0.33 [0.01, 8.19]
Oral_semaglutide vs Placebo_or_Control
Husain_M_2019_PIONEER_6_ 0.75 [0.17, 3.35]
Rosenstock_J_2019_PIONEER_3_ 1.67 [0.08, 34.90]
Sotagliflozin vs Placebo_or_Control
Bhatt_D_L_2021_SCORED_ 2.00 [0.50, 8.00]
Cherney_D_Z_I_2023_SOTA_CKD3_ 0.99 [0.09, 10.93]
SOTA_INS_NCT03285594_ 0.11 [0.00, 2.77]
Danne_T_2018_inTandem2_ 1.48 [0.06, 36.49]
Inject_semaglutide_low_dosage vs Placebo_or_Control
Marso_S_P_2016_SUSTAIN_6_ 0.25 [0.03, 1.99]
Kaku_K_2018_SUSTAIN_ 0.50 [0.05, 4.87]
Inject_semaglutide_medium_dosage vs Placebo_or_Control
Marso_S_P_2016_SUSTAIN_6_ 0.50 [0.11, 2.36]
Kaku_K_2018_SUSTAIN_ 0.16 [0.01, 4.08]
Inject_semaglutide_medium_dosage vs Inject_semaglutide_low_dosage
Marso_S_P_2016_SUSTAIN_6_ 2.01 [0.18, 22.23]
Kaku_K_2018_SUSTAIN_ 0.33 [0.01, 8.12]
Dapagliflozin_low_dosage vs Placebo_or_Control
Bailey_C_J_2010_MB102_014_1 0.33 [0.01, 8.19]
Wilding_J_P_2012_1 0.32 [0.01, 7.99]
Dapagliflozin_medium_dosage vs Placebo_or_Control
Bailey_C_J_2010_MB102_014_2 0.33 [0.01, 8.19]
Wilding_J_P_2012_2 0.31 [0.01, 7.61]
Inject_semaglutide_high_dosage vs Placebo_or_Control
Wilding_J_P_H_2021_STEP_1_ 0.17 [0.01, 4.10]
Davies_M_2021_STEP_2_2 5.01 [0.24, 104.74]
Rubino_D_2021_STEP_4_ 1.51 [0.06, 37.12]
Lincoff_A_M_2023_SELECT_ 1.00 [0.55, 1.83]
Inject_semaglutide_high_dosage vs Inject_semaglutide_medium_dosage
Davies_M_2021_STEP_2_1 5.01 [0.24, 104.74]

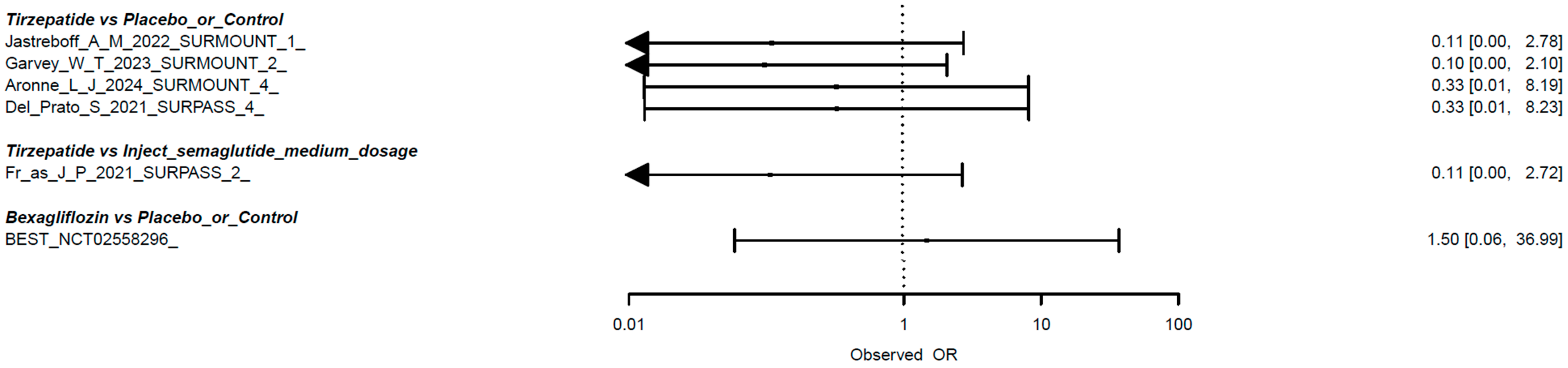
Tirzepatide vs Placebo_or_Control
Jastreboff_A_M_2022_SURMOUNT_1_ 0.11 [0.00, 2.78]
Garvey_W_T_2023_SURMOUNT_2_ 0.10 [0.00, 2.10]
Aronne_L_J_2024_SURMOUNT_4_ 0.33 [0.01, 8.19]
Del_Prato_S_2021_SURPASS_4_ 0.33 [0.01, 8.23]
Tirzepatide vs Inject_semaglutide_medium_dosage
Fr_as_J_P_2021_SURPASS_2_ 0.11 [0.00, 2.72]
Bexagliflozin vs Placebo_or_Control
BEST_NCT02558296_ 1.50 [0.06, 36.99]
0.01
1
10
100
Observed OR

## Figure S3B Individual study result of primary outcome: subgroup of lymphoma

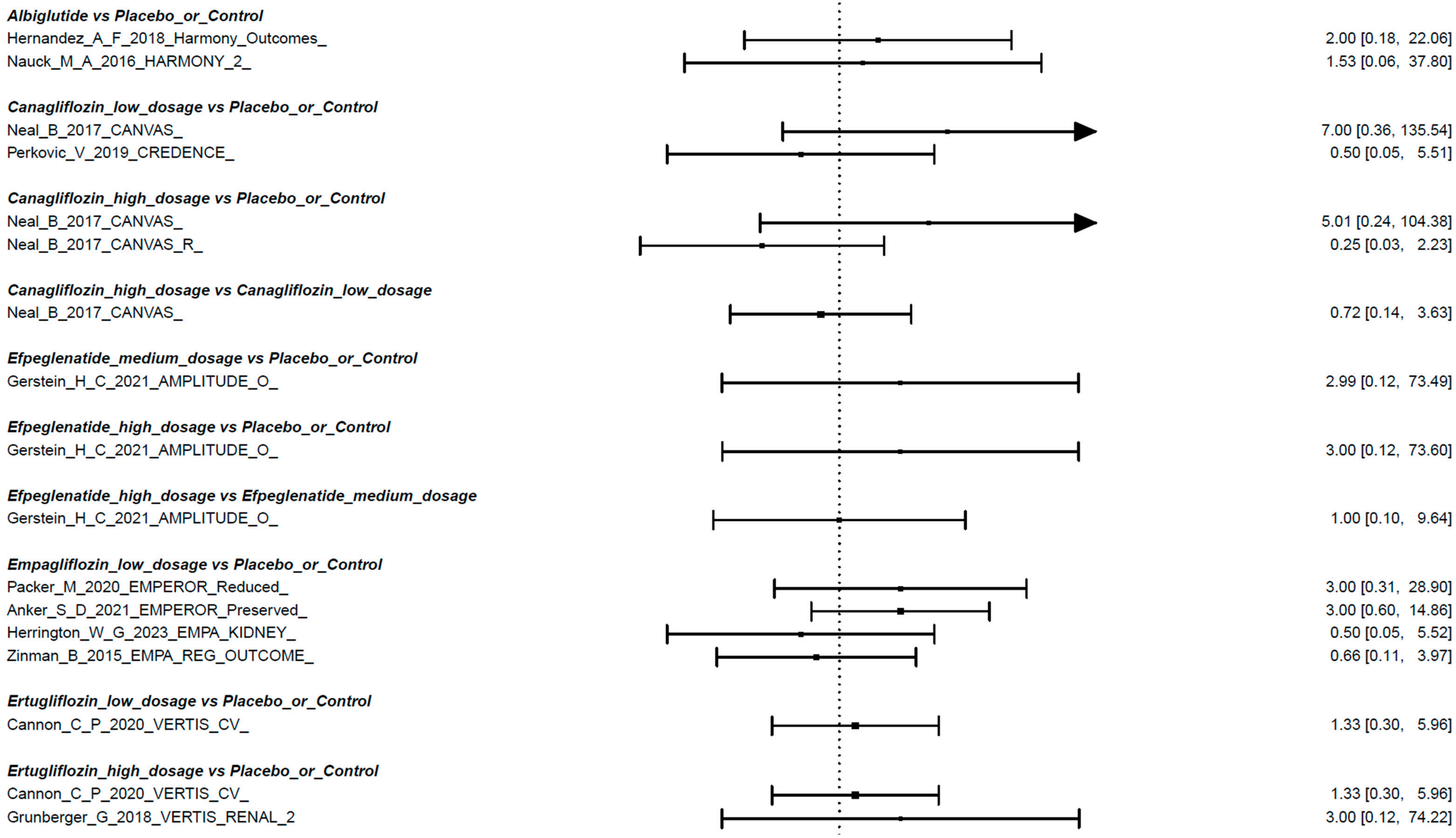

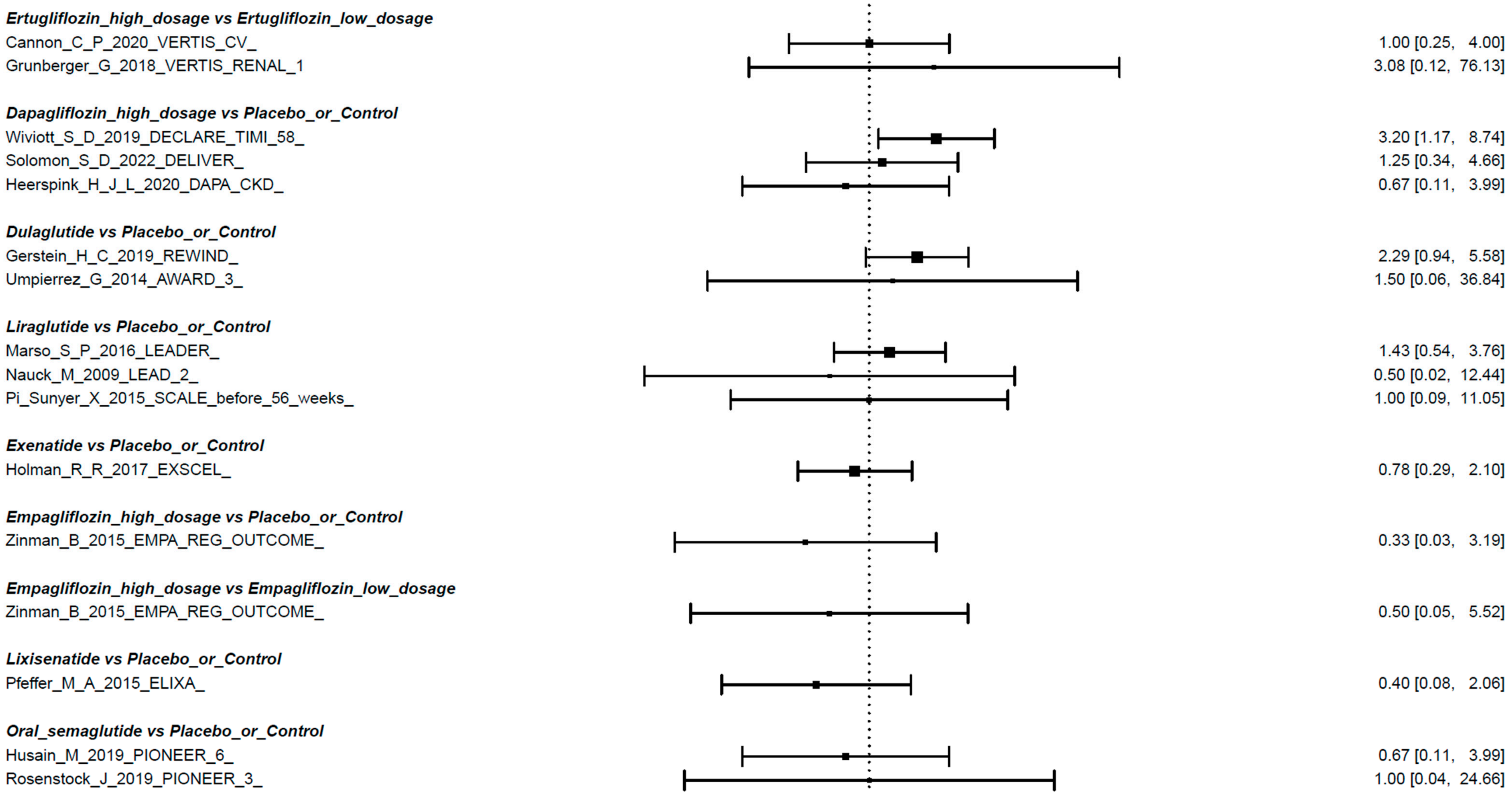
Ertugliflozin_high_dosage vs Ertugliflozin_low_dosage
Cannon_C_P_2020_VERTIS_CV_ 1.00 [0.25, 4.00]
Grunberger_G_2018_VERTIS_RENAL_1 3.08 [0.12, 76.13]
Dapagliflozin_high_dosage vs Placebo_or_Control
Wiviott_S_D_2019_DECLARE_TIMI_58_ 3.20 [1.17, 8.74]
Solomon_S_D_2022_DELIVER_ 1.25 [0.34, 4.66]
Heerspink_H_J_L_2020_DAPA_CKD_ 0.67 [0.11, 3.99]
Dulaglutide vs Placebo_or_Control
Gerstein_H_C_2019_REWIND_ 2.29 [0.94, 5.58]
Umpierrez_G_2014_AWARD_3_ 1.50 [0.06, 36.84]
Liraglutide vs Placebo_or_Control
Marso_S_P_2016_LEADER_ 1.43 [0.54, 3.76]
Nauck_M_2009_LEAD_2_ 0.50 [0.02, 12.44]
Pi_Sunyer_X_2015_SCALE_before_56_weeks_ 1.00 [0.09, 11.05]
Exenatide vs Placebo_or_Control
Holman_R_R_2017_EXSCEL_ 0.78 [0.29, 2.10]
Empagliflozin_high_dosage vs Placebo_or_Control
Zinman_B_2015_EMPA_REG_OUTCOME_ 0.33 [0.03, 3.19]
Empagliflozin_high_dosage vs Empagliflozin_low_dosage
Zinman_B_2015_EMPA_REG_OUTCOME_ 0.50 [0.05, 5.52]
Lixisenatide vs Placebo_or_Control
Pfeffer_M_A_2015_ELIXA_ 0.40 [0.08, 2.06]
Oral_semaglutide vs Placebo_or_Control
Husain_M_2019_PIONEER_6_ 0.67 [0.11, 3.99]
Rosenstock_J_2019_PIONEER_3_ 1.00 [0.04, 24.66]

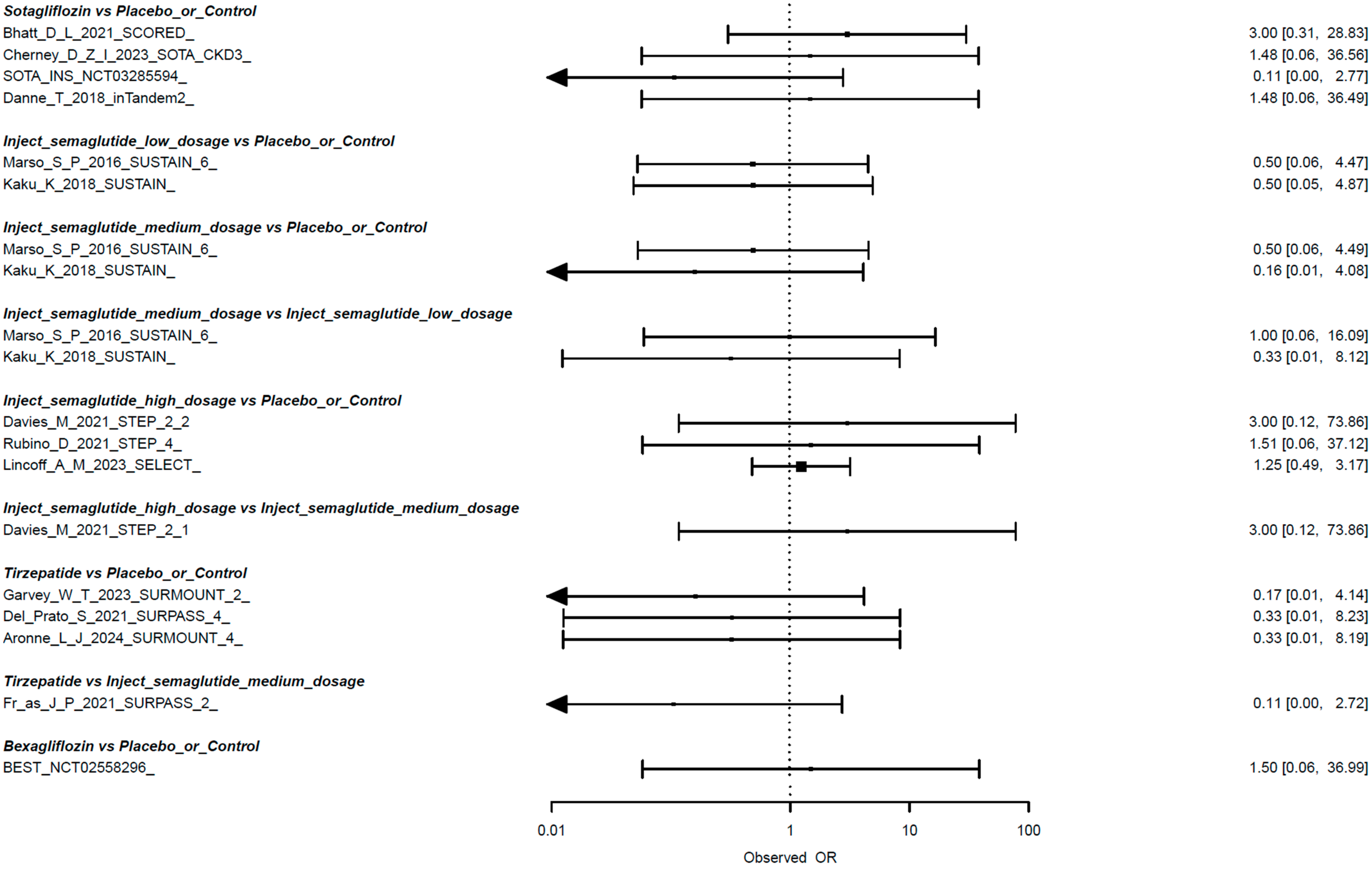
Sotagliflozin vs Placebo_or_Control
Bhatt_D_L_2021_SCORED_ 3.00 [0.31, 28.83]
Cherney_D_Z_I_2023_SOTA_CKD3_ 1.48 [0.06, 36.56]
SOTA_INS_NCT03285594_ 0.11 [0.00, 2.77]
Danne_T_2018_inTandem2_ 1.48 [0.06, 36.49]
Inject_semaglutide_low_dosage vs Placebo_or_Control
Marso_S_P_2016_SUSTAIN_6_ 0.50 [0.06, 4.47]
Kaku_K_2018_SUSTAIN_ 0.50 [0.05, 4.87]
Inject_semaglutide_medium_dosage vs Placebo_or_Control
Marso_S_P_2016_SUSTAIN_6_ 0.50 [0.06, 4.49]
Kaku_K_2018_SUSTAIN_ 0.16 [0.01, 4.08]
Inject_semaglutide_medium_dosage vs Inject_semaglutide_low_dosage
Marso_S_P_2016_SUSTAIN_6_ 1.00 [0.06, 16.09]
Kaku_K_2018_SUSTAIN_ 0.33 [0.01, 8.12]
Inject_semaglutide_high_dosage vs Placebo_or_Control
Davies_M_2021_STEP_2_2 3.00 [0.12, 73.86]
Rubino_D_2021_STEP_4_ 1.51 [0.06, 37.12]
Lincoff_A_M_2023_SELECT_ 1.25 [0.49, 3.17]
Inject_semaglutide_high_dosage vs Inject_semaglutide_medium_dosage
Davies_M_2021_STEP_2_1 3.00 [0.12, 73.86]
Tirzepatide vs Placebo_or_Control
Garvey_W_T_2023_SURMOUNT_2_ 0.17 [0.01, 4.14]
Del_Prato_S_2021_SURPASS_4_ 0.33 [0.01, 8.23]
Aronne_L_J_2024_SURMOUNT_4_ 0.33 [0.01, 8.19]
Tirzepatide vs Inject_semaglutide_medium_dosage
Fr_as_J_P_2021_SURPASS_2_ 0.11 [0.00, 2.72]
Bexagliflozin vs Placebo_or_Control
BEST_NCT02558296_ 1.50 [0.06, 36.99]
0.01
1
10
100
Observed OR

## Figure S3C Individual study result of primary outcome: subgroup of leukemia

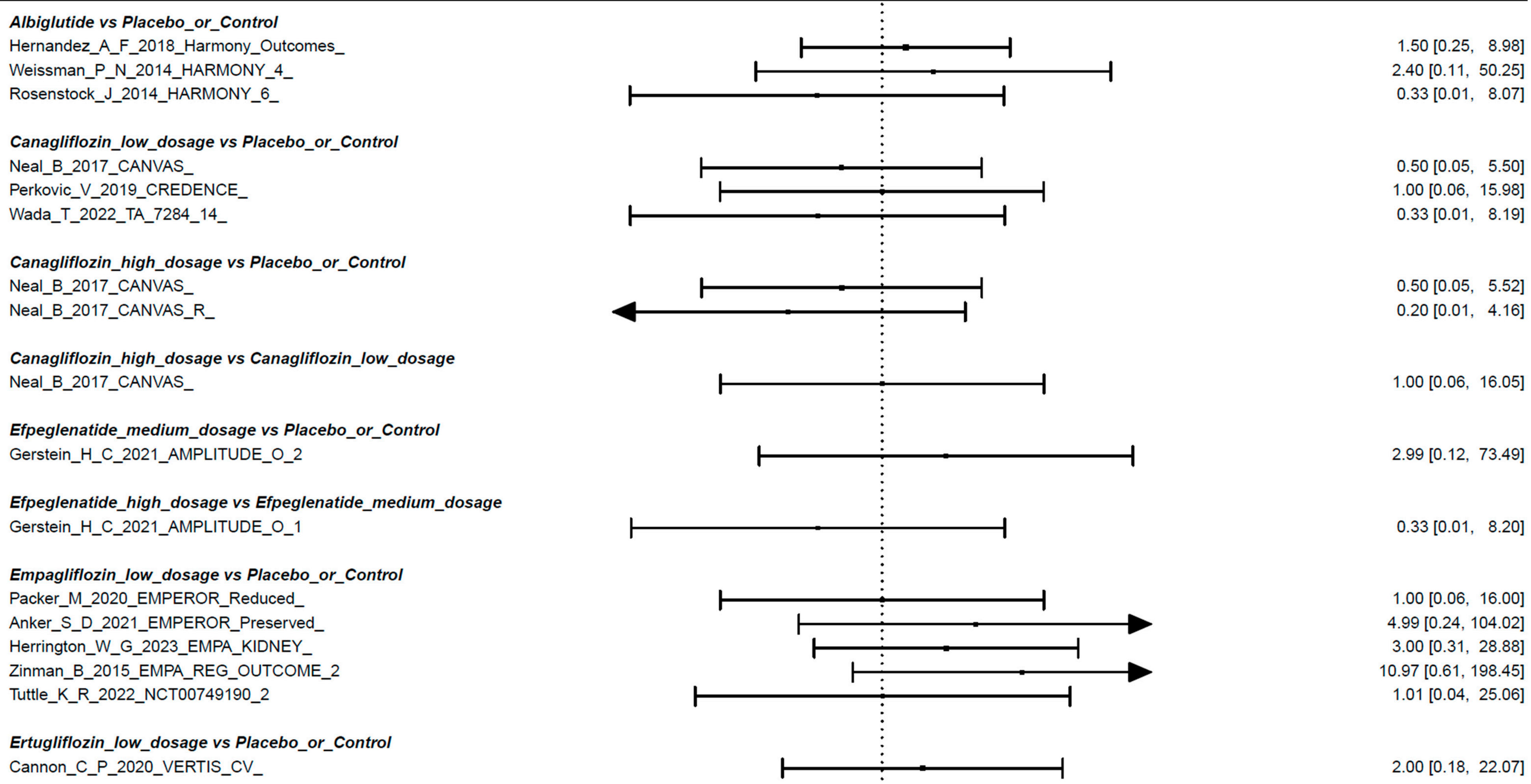

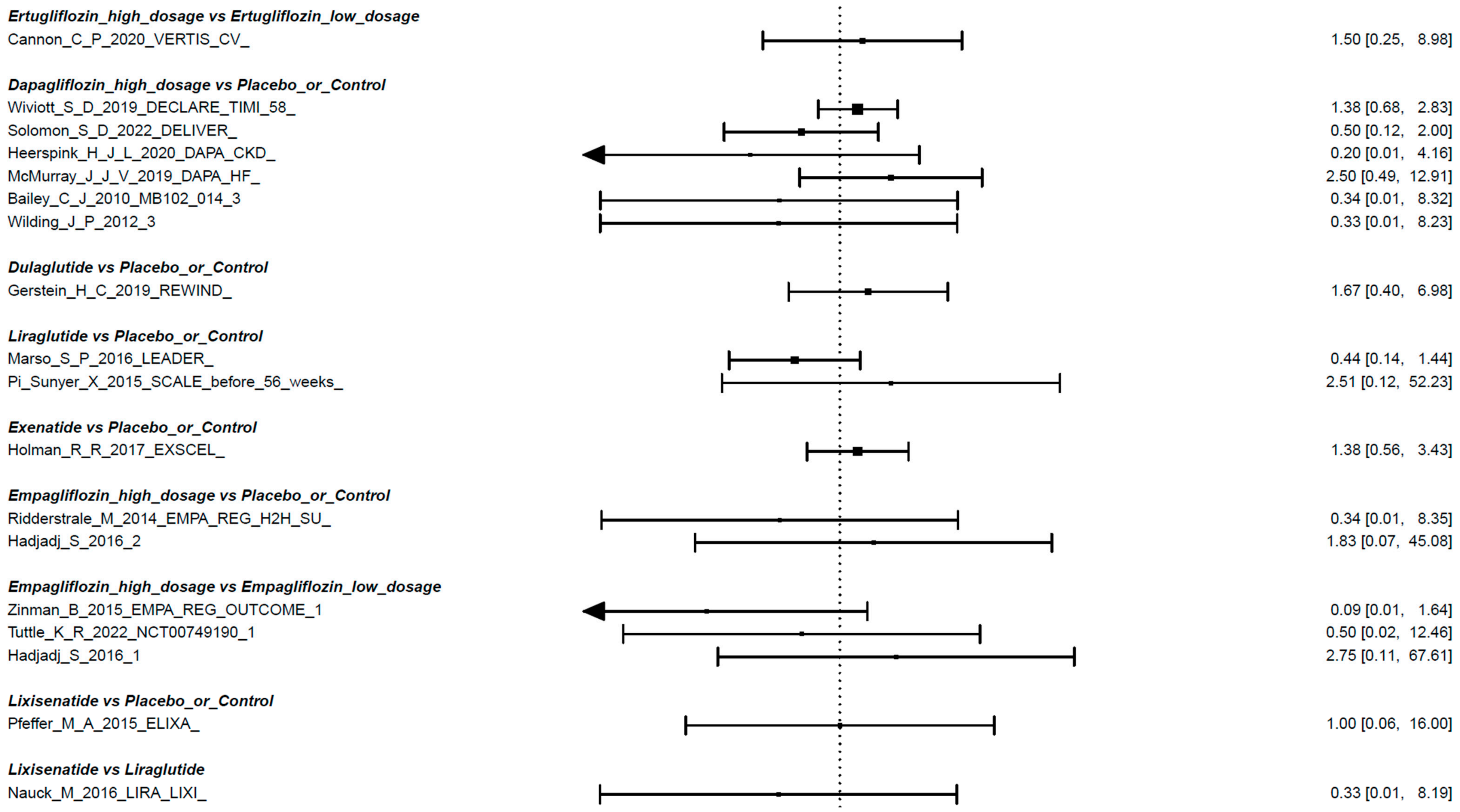
Ertugliflozin_high_dosage vs Ertugliflozin_low_dosage
Cannon_C_P_2020_VERTIS_CV_ 1.50 [0.25, 8.98]
Dapagliflozin_high_dosage vs Placebo_or_Control
Wiviott_S_D_2019_DECLARE_TIMI_58_ 1.38 [0.68, 2.83]
Solomon_S_D_2022_DELIVER_ 0.50 [0.12, 2.00]
Heerspink_H_J_L_2020_DAPA_CKD_ 0.20 [0.01, 4.16]
McMurray_J_J_V_2019_DAPA_HF_ 2.50 [0.49, 12.91]
Bailey_C_J_2010_MB102_014_3 0.34 [0.01, 8.32]
Wilding_J_P_2012_3 0.33 [0.01, 8.23]
Dulaglutide vs Placebo_or_Control
Gerstein_H_C_2019_REWIND_ 1.67 [0.40, 6.98]
Liraglutide vs Placebo_or_Control
Marso_S_P_2016_LEADER_ 0.44 [0.14, 1.44]
Pi_Sunyer_X_2015_SCALE_before_56_weeks_ 2.51 [0.12, 52.23]
Exenatide vs Placebo_or_Control
Holman_R_R_2017_EXSCEL_ 1.38 [0.56, 3.43]
Empagliflozin_high_dosage vs Placebo_or_Control
Ridderstrale_M_2014_EMPA_REG_H2H_SU_ 0.34 [0.01, 8.35]
Hadjadj_S_2016_2 1.83 [0.07, 45.08]
Empagliflozin_high_dosage vs Empagliflozin_low_dosage
Zinman_B_2015_EMPA_REG_OUTCOME_1 0.09 [0.01, 1.64]
Tuttle_K_R_2022_NCT00749190_1 0.50 [0.02, 12.46]
Hadjadj_S_2016_1 2.75 [0.11, 67.61]
Lixisenatide vs Placebo_or_Control
Pfeffer_M_A_2015_ELIXA_ 1.00 [0.06, 16.00]
Lixisenatide vs Liraglutide
Nauck_M_2016_LIRA_LIXI_ 0.33 [0.01, 8.19]

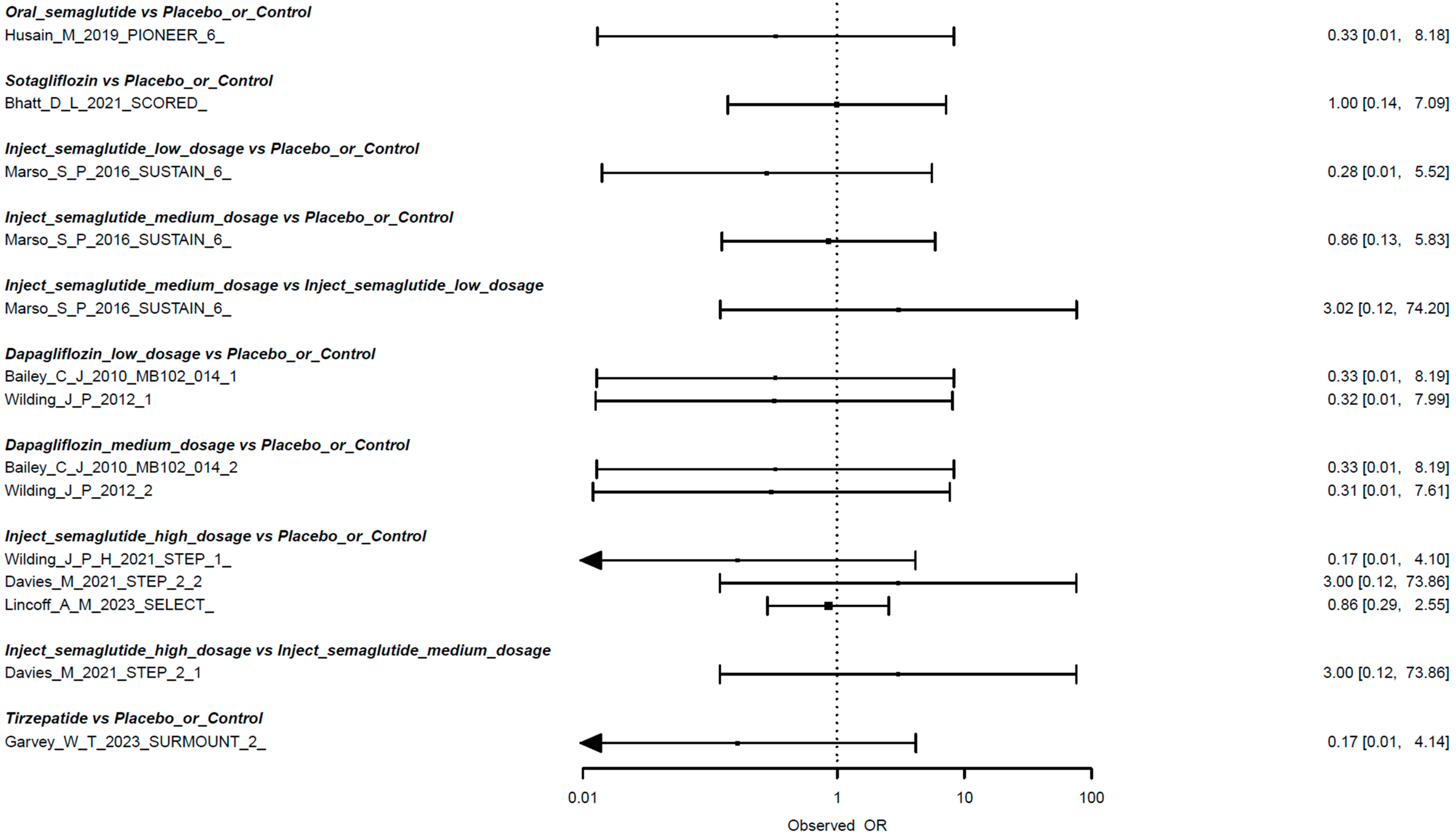
Oral_semaglutide vs Placebo_or_Control
Husain_M_2019_PIONEER_6_ 0.33 [0.01, 8.18]
Sotagliflozin vs Placebo_or_Control
Bhatt_D_L_2021_SCORED_ 1.00 [0.14, 7.09]
Inject_semaglutide_low_dosage vs Placebo_or_Control
Marso_S_P_2016_SUSTAIN_6_ 0.28 [0.01, 5.52]
Inject_semaglutide_medium_dosage vs Placebo_or_Control
Marso_S_P_2016_SUSTAIN_6_ 0.86 [0.13, 5.83]
Inject_semaglutide_medium_dosage vs Inject_semaglutide_low_dosage
Marso_S_P_2016_SUSTAIN_6_ 3.02 [0.12, 74.20]
Dapagliflozin_low_dosage vs Placebo_or_Control
Bailey_C_J_2010_MB102_014_1 0.33 [0.01, 8.19]
Wilding_J_P_2012_1 0.32 [0.01, 7.99]
Dapagliflozin_medium_dosage vs Placebo_or_Control
Bailey_C_J_2010_MB102_014_2 0.33 [0.01, 8.19]
Wilding_J_P_2012_2 0.31 [0.01, 7.61]
Inject_semaglutide_high_dosage vs Placebo_or_Control
Wilding_J_P_H_2021_STEP_1_ 0.17 [0.01, 4.10]
Davies_M_2021_STEP_2_2 3.00 [0.12, 73.86]
Lincoff_A_M_2023_SELECT_ 0.86 [0.29, 2.55]
Inject_semaglutide_high_dosage vs Inject_semaglutide_medium_dosage
Davies_M_2021_STEP_2_1 3.00 [0.12, 73.86]
Tirzepatide vs Placebo_or_Control
Garvey_W_T_2023_SURMOUNT_2_ 0.17 [0.01, 4.14]
0.01
1
10
100
Observed OR

# Figure S3D Individual study result of primary outcome: subgroup of myeloma

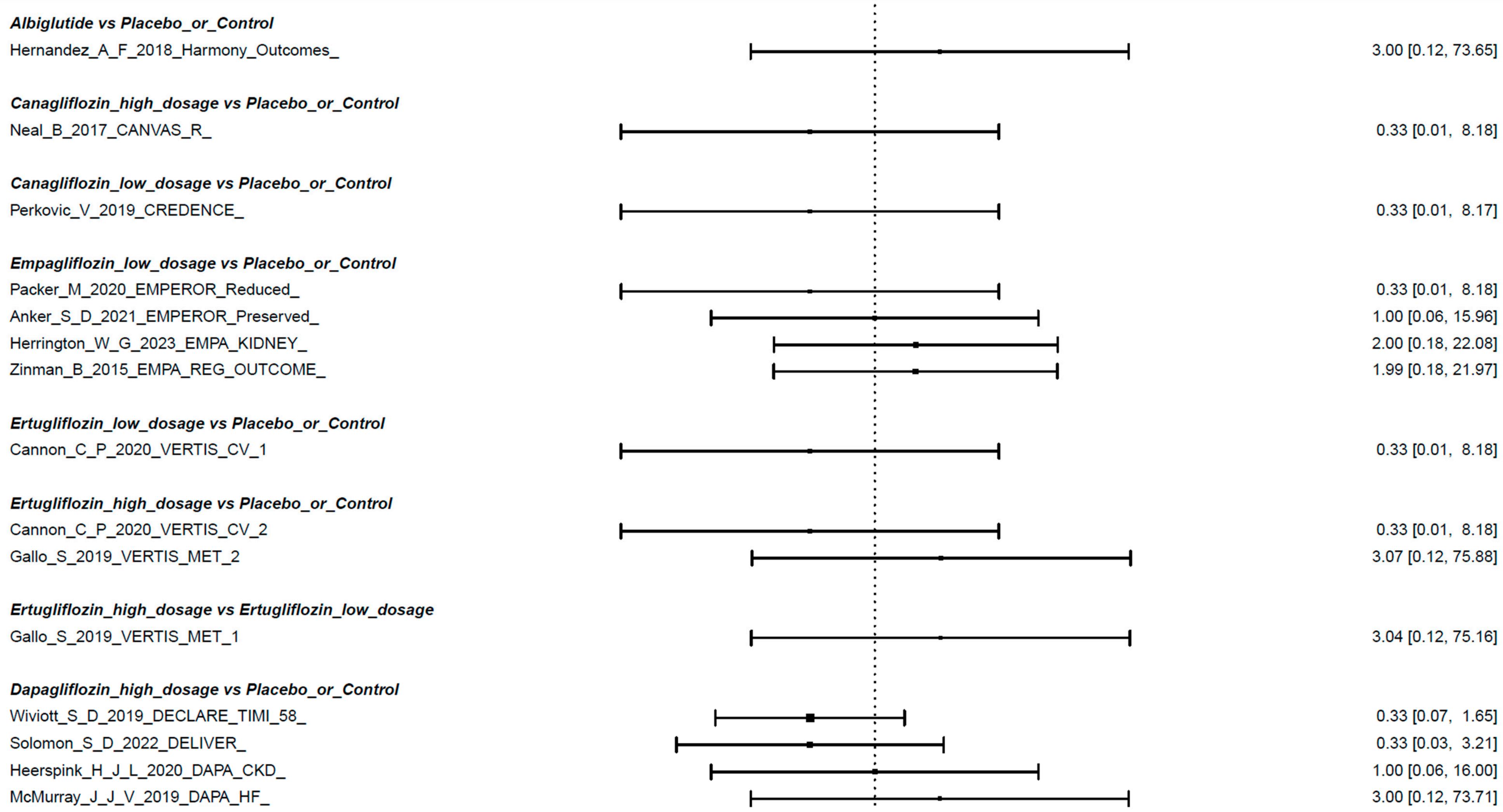

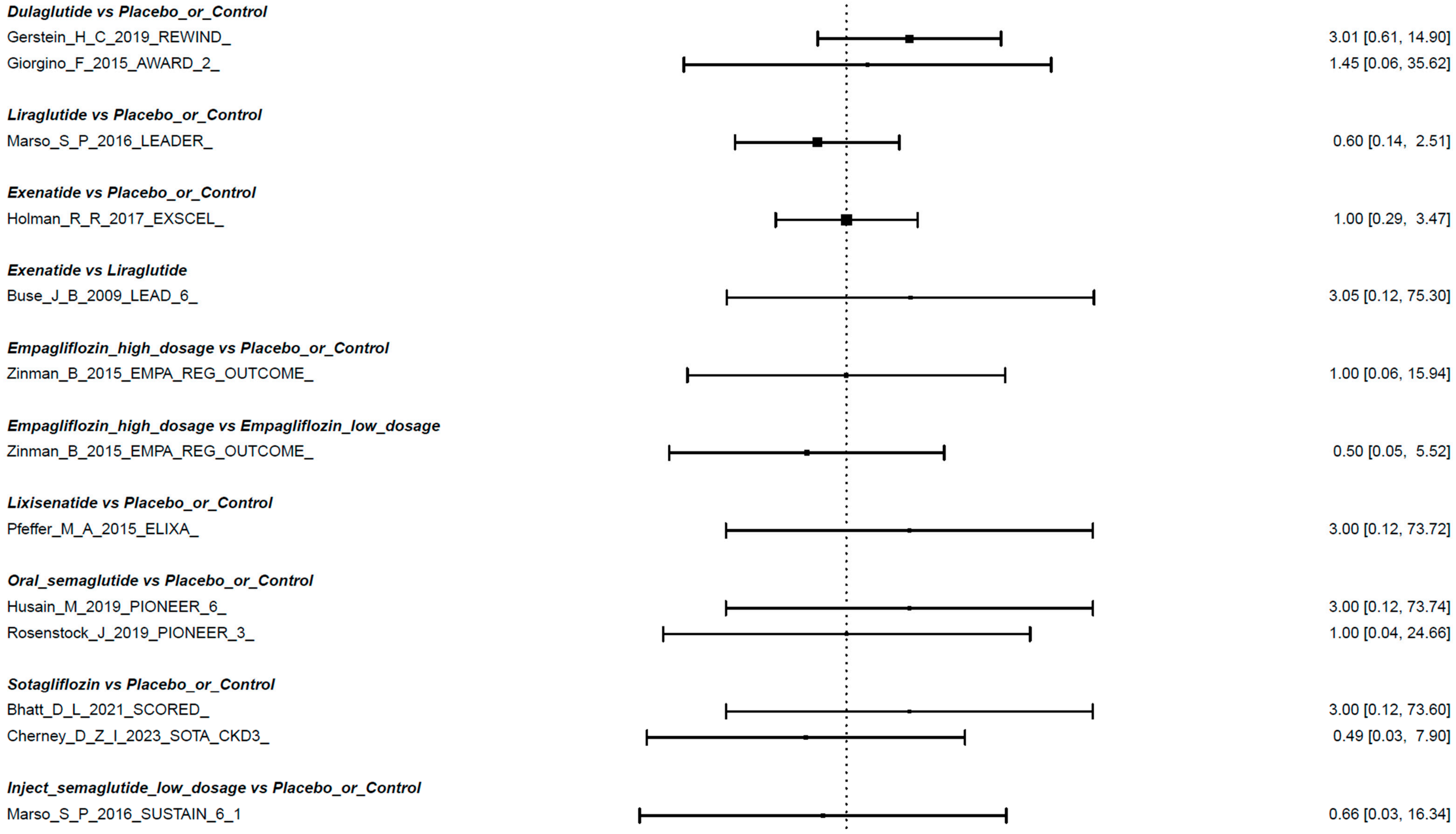

Dulaglutide vs Placebo_or_Control
Gerstein_H_C_2019_REWIND_
3.01 [0.61, 14.90]
Giorgino_F_2015_AWARD_2_
1.45 [0.06, 35.62]
Liraglutide vs Placebo_or_Control
Marso_S_P_2016_LEADER_
0.60 [0.14, 2.51]
Exenatide vs Placebo_or_Control
Holman_R_R_2017_EXSCEL_
1.00 [0.29, 3.47]
Exenatide vs Liraglutide
Buse_J_B_2009_LEAD_6_
3.05 [0.12, 75.30]
Empagliflozin_high_dosage vs Placebo_or_Control
Zinman_B_2015_EMPA_REG_OUTCOME_
1.00 [0.06, 15.94]
Empagliflozin_high_dosage vs Empagliflozin_low_dosage
Zinman_B_2015_EMPA_REG_OUTCOME_
0.50 [0.05, 5.52]
Lixisenatide vs Placebo_or_Control
Pfeffer_M_A_2015_ELIXA_
3.00 [0.12, 73.72]
Oral_semaglutide vs Placebo_or_Control
Husain_M_2019_PIONEER_6_
3.00 [0.12, 73.74]
Rosenstock_J_2019_PIONEER_3_
1.00 [0.04, 24.66]
Sotagliflozin vs Placebo_or_Control
Bhatt_D_L_2021_SCORED_
3.00 [0.12, 73.60]
Cherney_D_Z_I_2023_SOTA_CKD3_
0.49 [0.03, 7.90]
Inject_semaglutide_low_dosage vs Placebo_or_Control
Marso_S_P_2016_SUSTAIN_6_1
0.66 [0.03, 16.34]

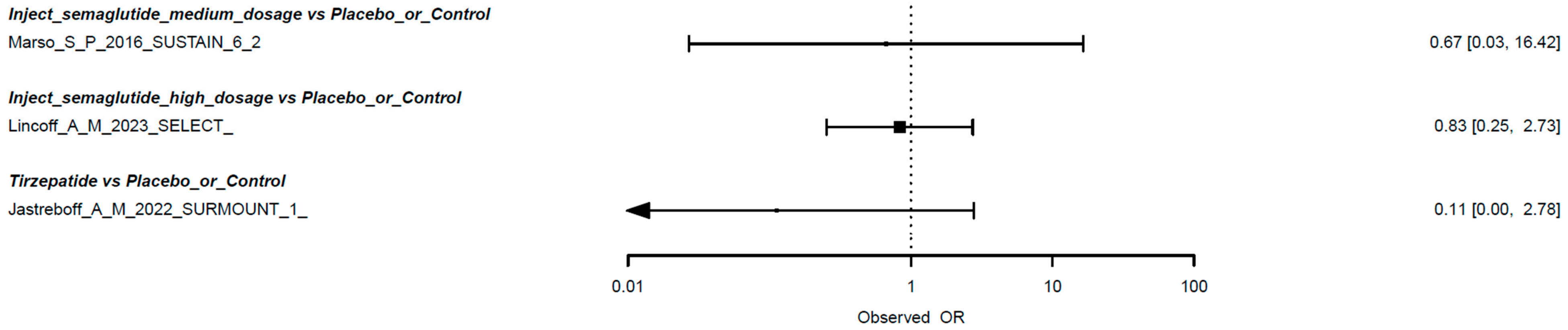
Inject_semaglutide_medium_dosage vs Placebo_or_Control
Marso_S_P_2016_SUSTAIN_6_2
0.67 [0.03, 16.42]
Inject_semaglutide_high_dosage vs Placebo_or_Control
Lincoff_A_M_2023_SELECT_
0.83 [0.25, 2.73]
Tirzepatide vs Placebo_or_Control
Jastreboff_A_M_2022_SURMOUNT_1_
0.11 [0.00, 2.78]
0.01
1
10
100
Observed OR

## Figure S3E Individual study result of safety profile: drop-out rate

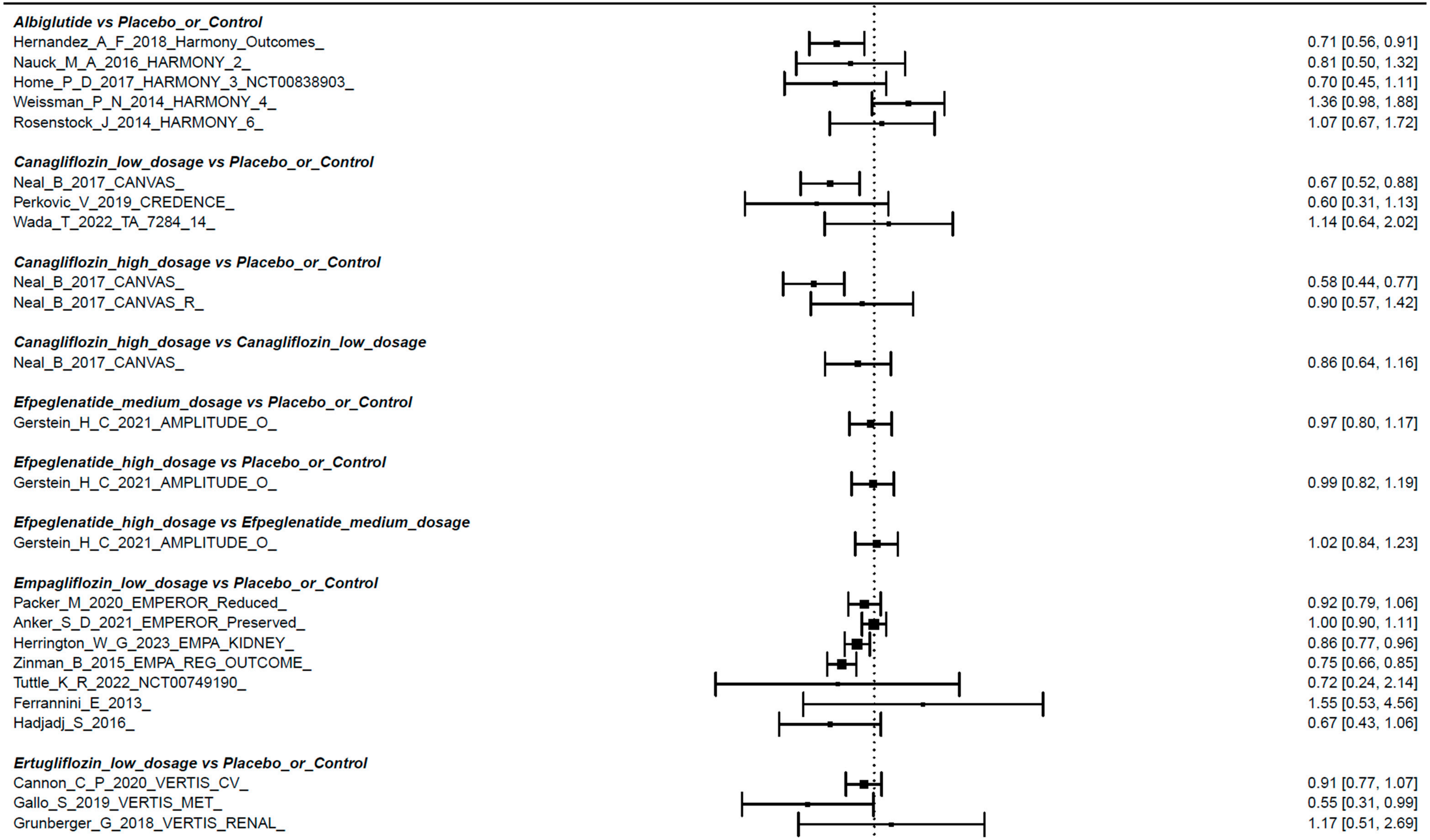

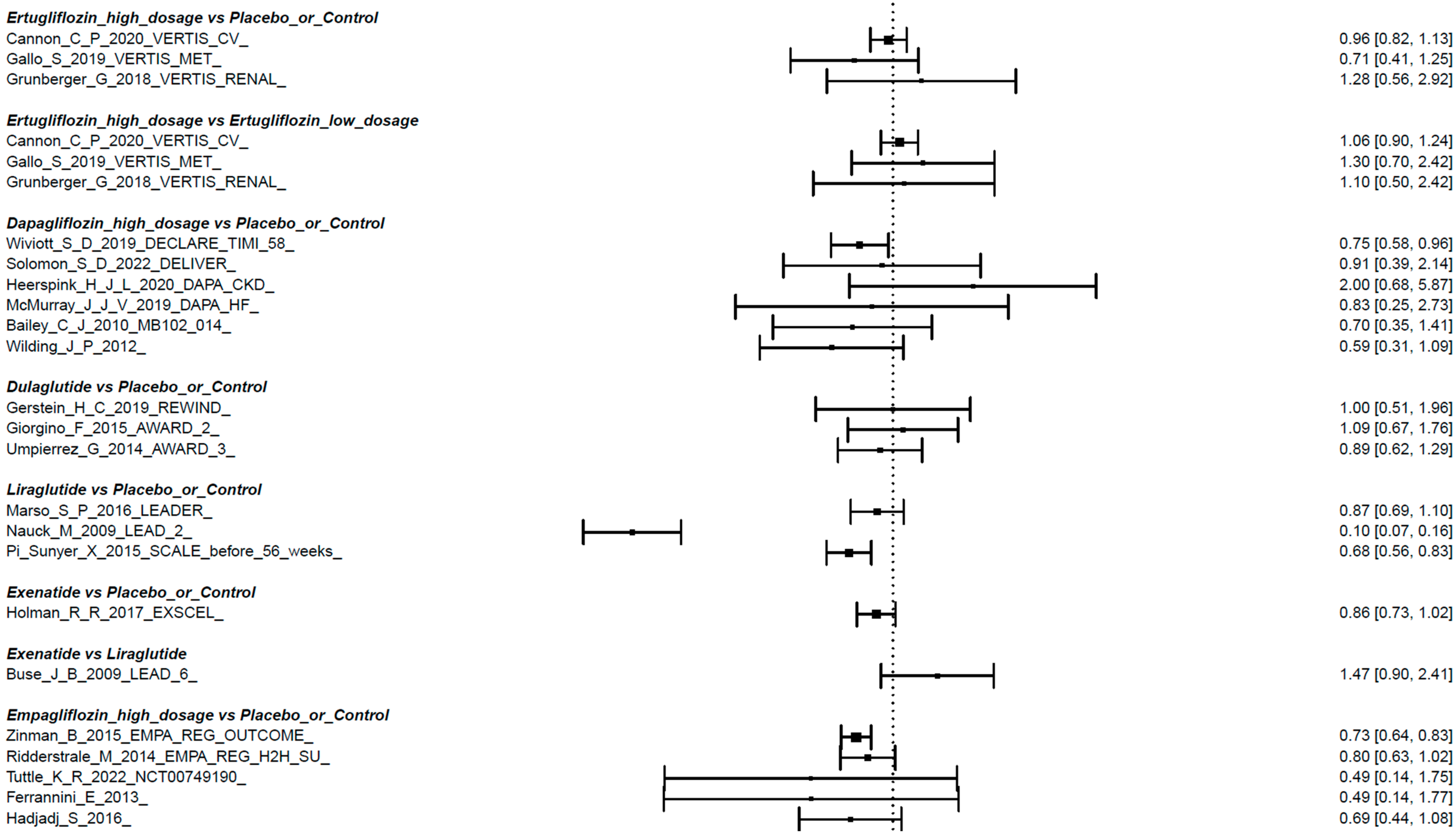

Ertugliflozin_high_dosage vs Placebo_or_Control
Cannon_C_P_2020_VERTIS_CV_ 0.96 [0.82, 1.13]
Gallo_S_2019_VERTIS_MET_ 0.71 [0.41, 1.25]
Grunberger_G_2018_VERTIS_RENAL_ 1.28 [0.56, 2.92]
Ertugliflozin_high_dosage vs Ertugliflozin_low_dosage
Cannon_C_P_2020_VERTIS_CV_ 1.06 [0.90, 1.24]
Gallo_S_2019_VERTIS_MET_ 1.30 [0.70, 2.42]
Grunberger_G_2018_VERTIS_RENAL_ 1.10 [0.50, 2.42]
Dapagliflozin_high_dosage vs Placebo_or_Control
Wiviott_S_D_2019_DECLARE_TIMI_58_ 0.75 [0.58, 0.96]
Solomon_S_D_2022_DELIVER_ 0.91 [0.39, 2.14]
Heerspink_H_J_L_2020_DAPA_CKD_ 2.00 [0.68, 5.87]
McMurray_J_J_V_2019_DAPA_HF_ 0.83 [0.25, 2.73]
Bailey_C_J_2010_MB102_014_ 0.70 [0.35, 1.41]
Wilding_J_P_2012_ 0.59 [0.31, 1.09]
Dulaglutide vs Placebo_or_Control
Gerstein_H_C_2019_REWIND_ 1.00 [0.51, 1.96]
Giorgino_F_2015_AWARD_2_ 1.09 [0.67, 1.76]
Umpierrez_G_2014_AWARD_3_ 0.89 [0.62, 1.29]
Liraglutide vs Placebo_or_Control
Marso_S_P_2016_LEADER_ 0.87 [0.69, 1.10]
Nauck_M_2009_LEAD_2_ 0.10 [0.07, 0.16]
Pi_Sunyer_X_2015_SCALE_before_56_weeks_ 0.68 [0.56, 0.83]
Exenatide vs Placebo_or_Control
Holman_R_R_2017_EXSCEL_ 0.86 [0.73, 1.02]
Exenatide vs Liraglutide
Buse_J_B_2009_LEAD_6_ 1.47 [0.90, 2.41]
Empagliflozin_high_dosage vs Placebo_or_Control
Zinman_B_2015_EMPA_REG_OUTCOME_ 0.73 [0.64, 0.83]
Ridderstrale_M_2014_EMPA_REG_H2H_SU_ 0.80 [0.63, 1.02]
Tuttle_K_R_2022_NCT00749190_ 0.49 [0.14, 1.75]
Ferrannini_E_2013_ 0.49 [0.14, 1.77]
Hadjadj_S_2016_ 0.69 [0.44, 1.08]

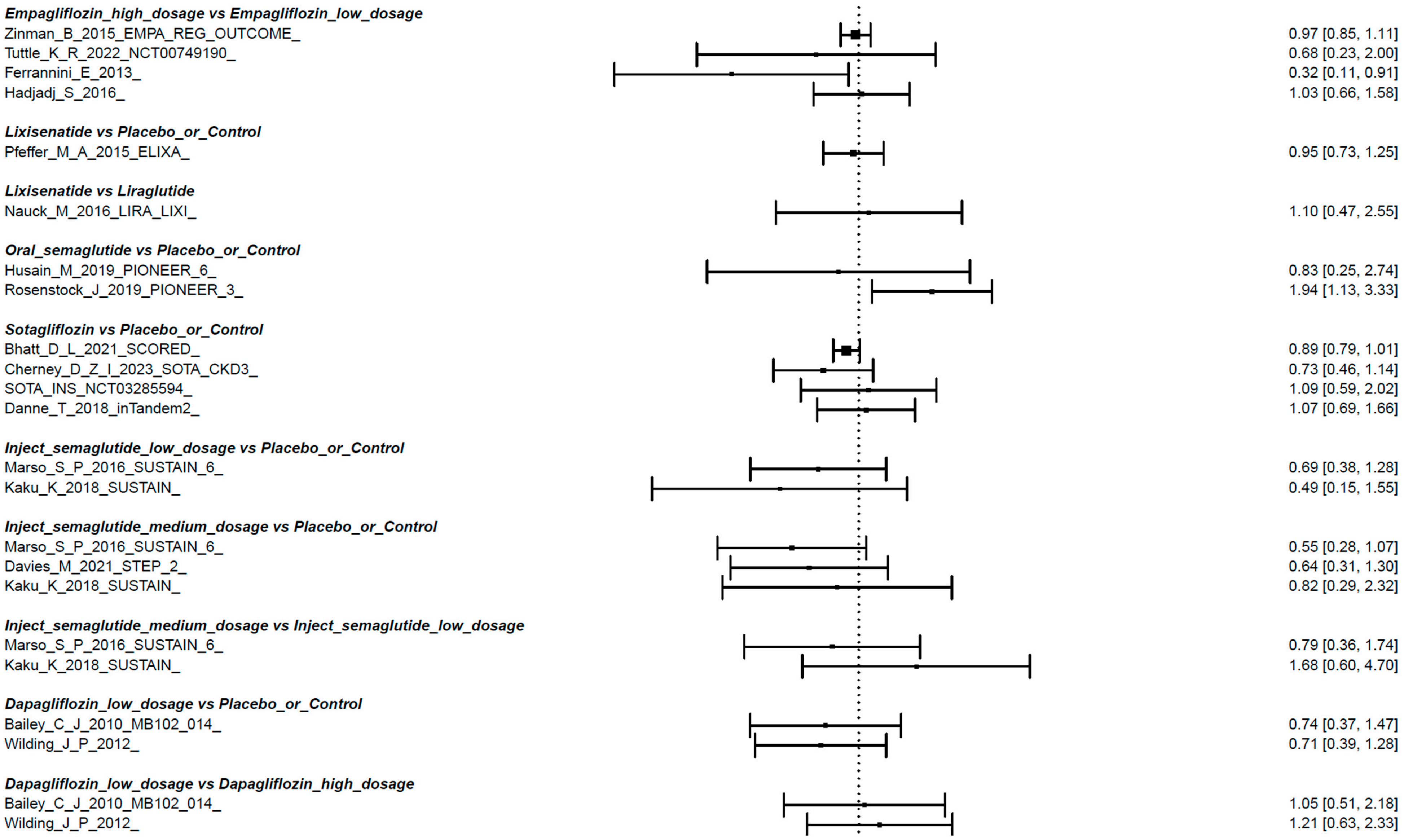
Empagliflozin_high_dosage vs Empagliflozin_low_dosage
Zinman_B_2015_EMPA_REG_OUTCOME_ 0.97 [0.85, 1.11]
Tuttle_K_R_2022_NCT00749190_ 0.68 [0.23, 2.00]
Ferrannini_E_2013_ 0.32 [0.11, 0.91]
Hadjadj_S_2016_ 1.03 [0.66, 1.58]
Lixisenatide vs Placebo_or_Control
Pfeffer_M_A_2015_ELIXA_ 0.95 [0.73, 1.25]
Lixisenatide vs Liraglutide
Nauck_M_2016_LIRA_LIXI_ 1.10 [0.47, 2.55]
Oral_semaglutide vs Placebo_or_Control
Husain_M_2019_PIONEER_6_ 0.83 [0.25, 2.74]
Rosenstock_J_2019_PIONEER_3_ 1.94 [1.13, 3.33]
Sotagliflozin vs Placebo_or_Control
Bhatt_D_L_2021_SCORED_ 0.89 [0.79, 1.01]
Cherney_D_Z_I_2023_SOTA_CKD3_ 0.73 [0.46, 1.14]
SOTA_INS_NCT03285594_ 1.09 [0.59, 2.02]
Danne_T_2018_inTandem2_ 1.07 [0.69, 1.66]
Inject_semaglutide_low_dosage vs Placebo_or_Control
Marso_S_P_2016_SUSTAIN_6_ 0.69 [0.38, 1.28]
Kaku_K_2018_SUSTAIN_ 0.49 [0.15, 1.55]
Inject_semaglutide_medium_dosage vs Placebo_or_Control
Marso_S_P_2016_SUSTAIN_6_ 0.55 [0.28, 1.07]
Davies_M_2021_STEP_2_ 0.64 [0.31, 1.30]
Kaku_K_2018_SUSTAIN_ 0.82 [0.29, 2.32]
Inject_semaglutide_medium_dosage vs Inject_semaglutide_low_dosage
Marso_S_P_2016_SUSTAIN_6_ 0.79 [0.36, 1.74]
Kaku_K_2018_SUSTAIN_ 1.68 [0.60, 4.70]
Dapagliflozin_low_dosage vs Placebo_or_Control
Bailey_C_J_2010_MB102_014_ 0.74 [0.37, 1.47]
Wilding_J_P_2012_ 0.71 [0.39, 1.28]
Dapagliflozin_low_dosage vs Dapagliflozin_high_dosage
Bailey_C_J_2010_MB102_014_ 1.05 [0.51, 2.18]
Wilding_J_P_2012_ 1.21 [0.63, 2.33]

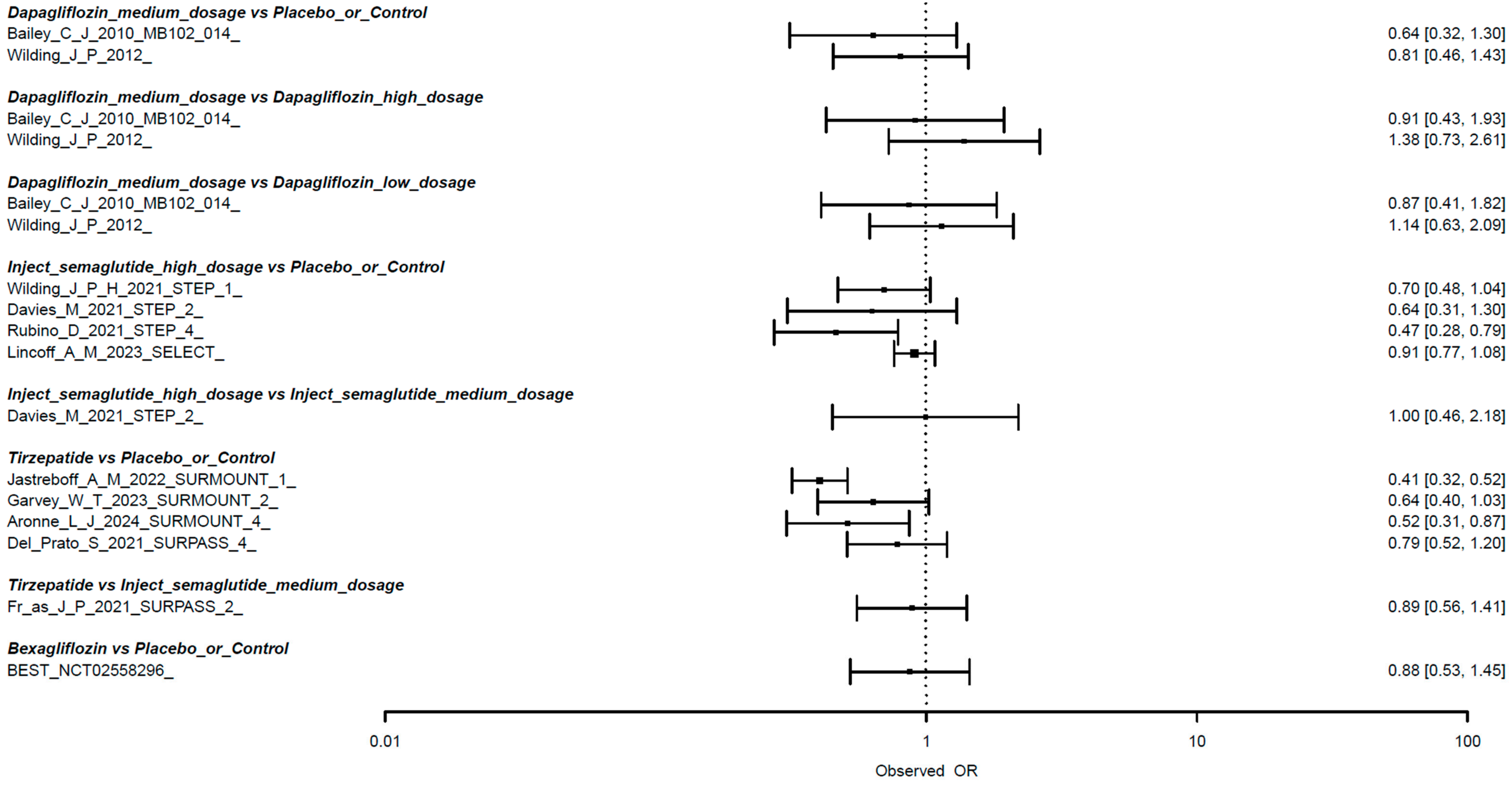

Dapagliflozin_medium_dosage vs Placebo_or_Control
Bailey_C_J_2010_MB102_014_
0.64 [0.32, 1.30]
Wilding_J_P_2012_
0.81 [0.46, 1.43]
Dapagliflozin_medium_dosage vs Dapagliflozin_high_dosage
Bailey_C_J_2010_MB102_014_
0.91 [0.43, 1.93]
Wilding_J_P_2012_
1.38 [0.73, 2.61]
Dapagliflozin_medium_dosage vs Dapagliflozin_low_dosage
Bailey_C_J_2010_MB102_014_
0.87 [0.41, 1.82]
Wilding_J_P_2012_
1.14 [0.63, 2.09]
Inject_semaglutide_high_dosage vs Placebo_or_Control
Wilding_J_P_H_2021_STEP_1_
0.70 [0.48, 1.04]
Davies_M_2021_STEP_2_
0.64 [0.31, 1.30]
Rubino_D_2021_STEP_4_
0.47 [0.28, 0.79]
Lincoff_A_M_2023_SELECT_
0.91 [0.77, 1.08]
Inject_semaglutide_high_dosage vs Inject_semaglutide_medium_dosage
Davies_M_2021_STEP_2_
1.00 [0.46, 2.18]
Tirzepatide vs Placebo_or_Control
Jastreboff_A_M_2022_SURMOUNT_1_
0.41 [0.32, 0.52]
Garvey_W_T_2023_SURMOUNT_2_
0.64 [0.40, 1.03]
Aronne_L_J_2024_SURMOUNT_4_
0.52 [0.31, 0.87]
Del_Prato_S_2021_SURPASS_4_
0.79 [0.52, 1.20]
Tirzepatide vs Inject_semaglutide_medium_dosage
Fr_as_J_P_2021_SURPASS_2_
0.89 [0.56, 1.41]
Bexagliflozin vs Placebo_or_Control
BEST_NCT02558296_
0.88 [0.53, 1.45]
0.01
1
10
100
Observed OR

***Abbreviation for Figure S3A-E:***

*95%CIs: 95% confidence intervals; GLP-1 agonist: glucagon-like peptide-1 agonist; NMA: network meta-analysis; OR: odds ratio; RCT: randomized controlled trial; SGLT2 inhibitor: sodium–glucose cotransporter 2 inhibitor*

**Figure S4A Bayesian-based forest plot of NMA of primary outcome: overall hematologic malignancy**

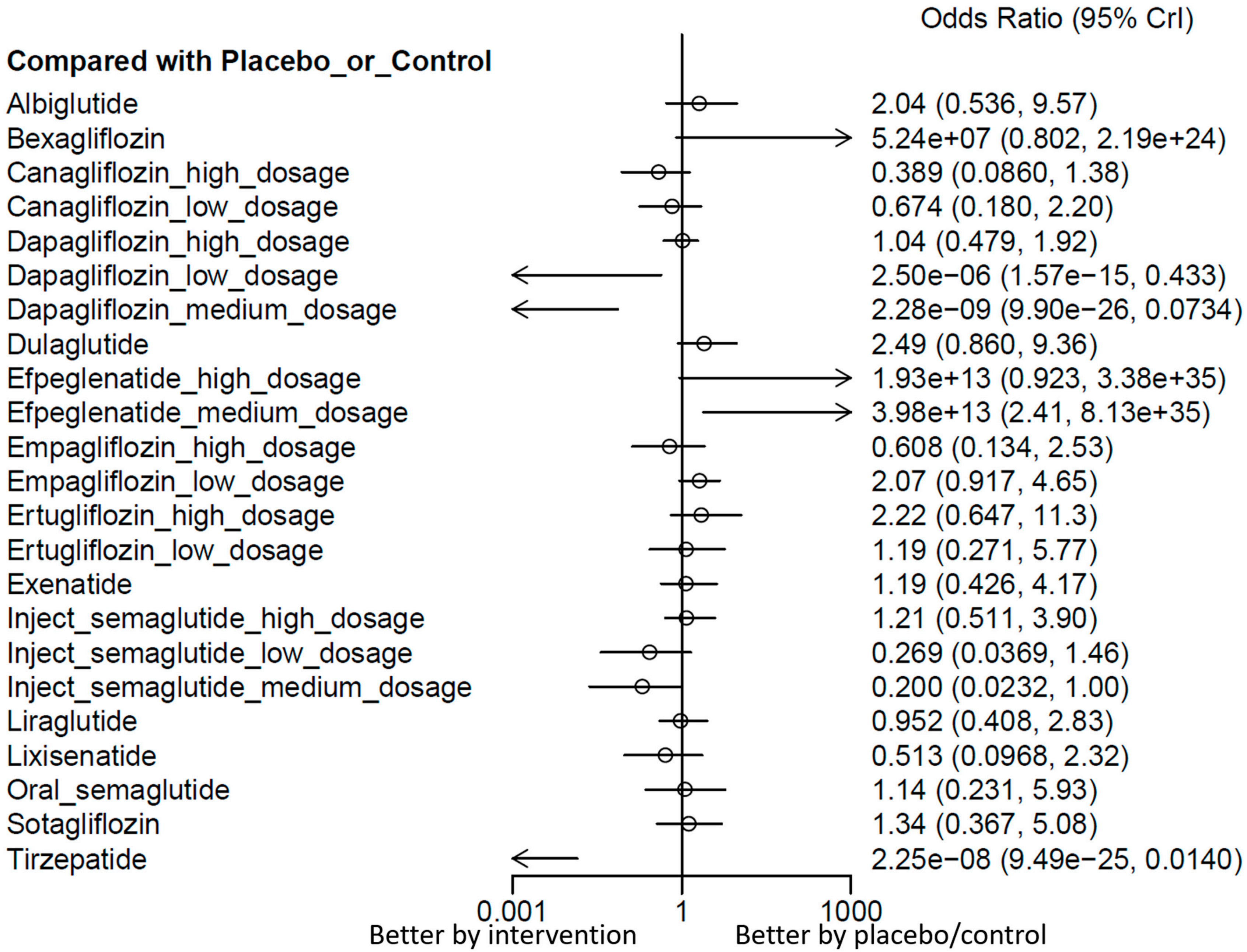

**Figure S4B Bayesian-based forest plot of NMA of primary outcome: subgroup of lymphoma**

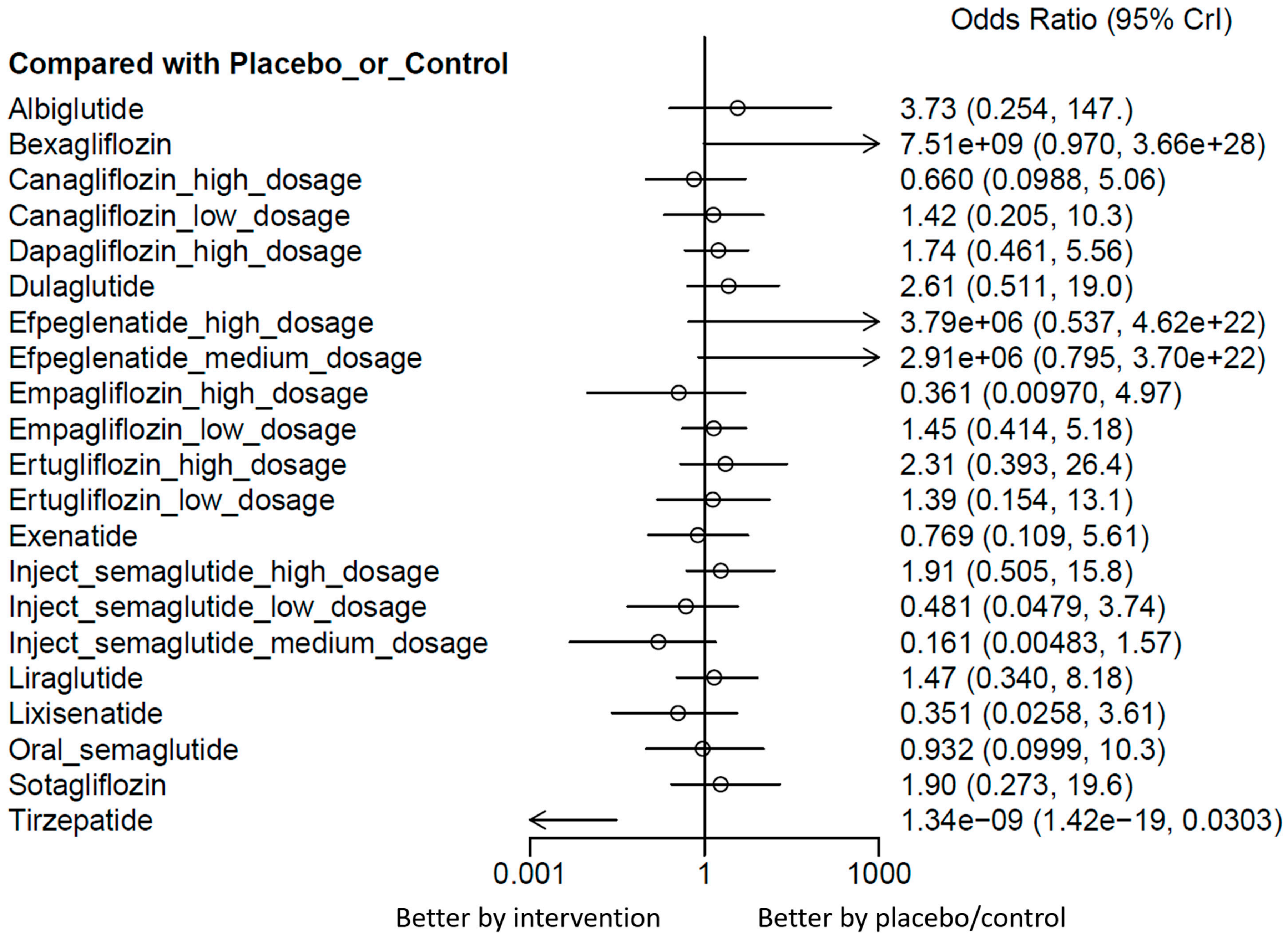

**Figure S4C Bayesian-based forest plot of NMA of primary outcome: subgroup of leukemia**

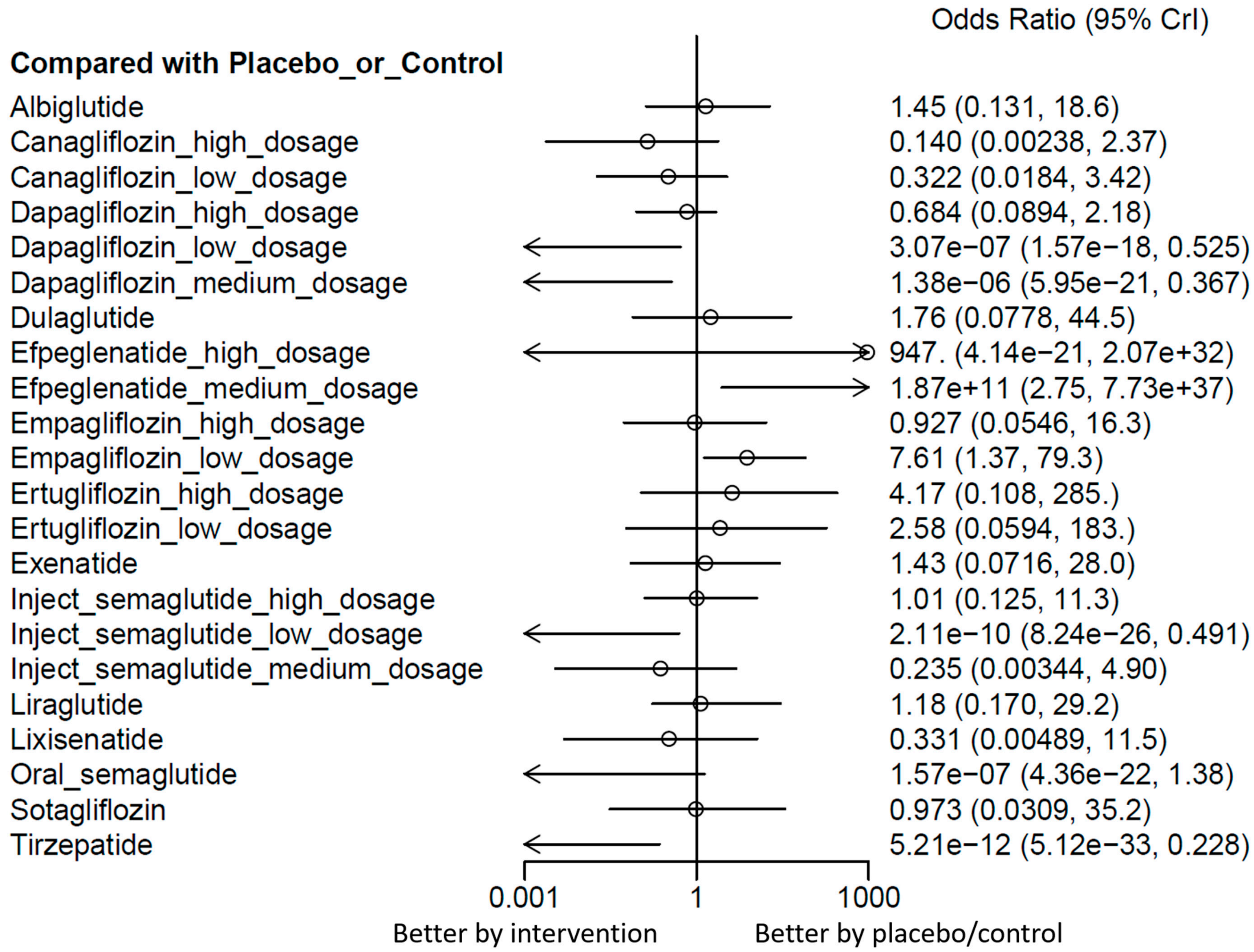

**Figure S4D Bayesian-based forest plot of NMA of primary outcome: subgroup of myeloma**

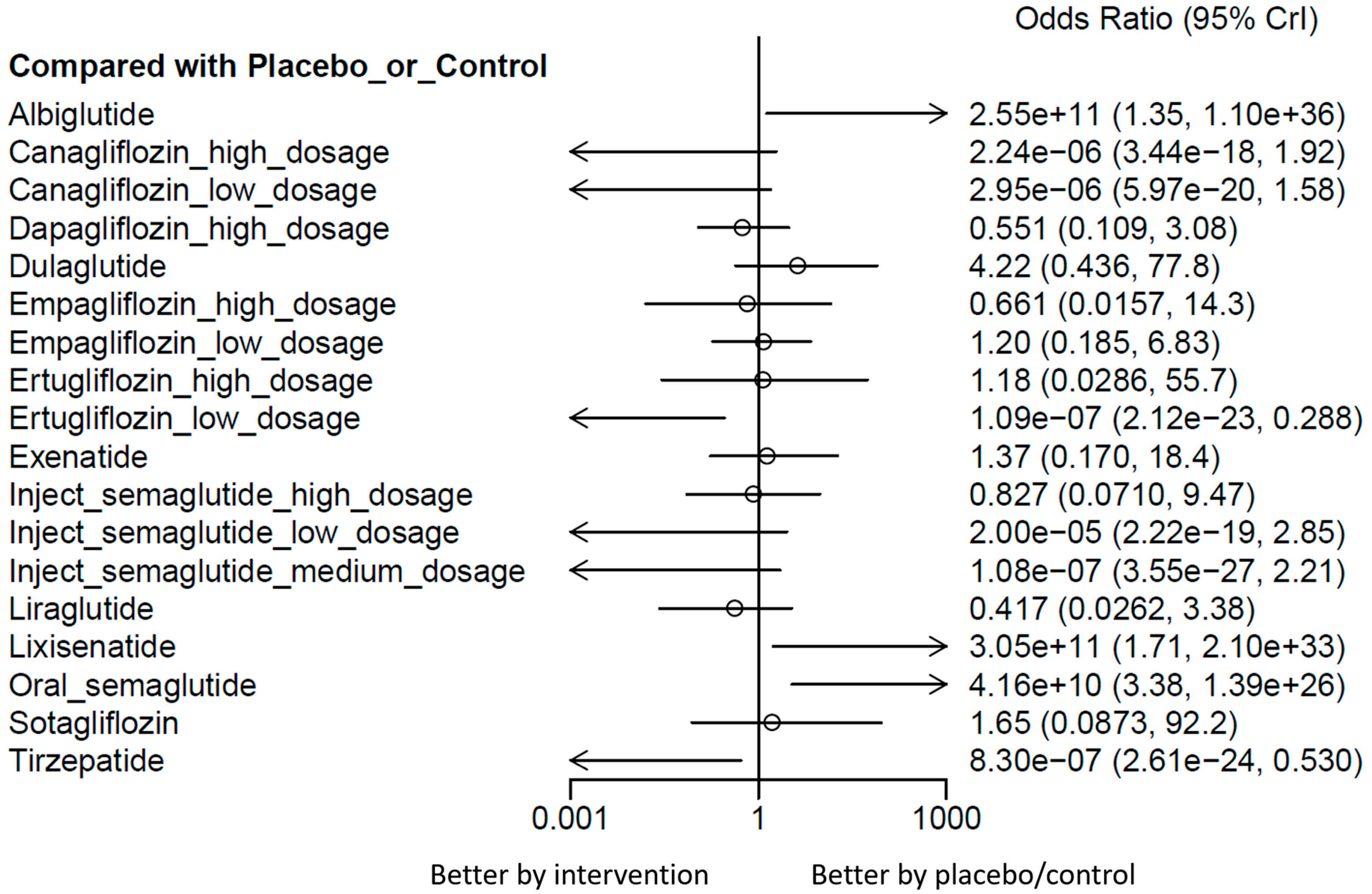

**Figure S4E Bayesian-based forest plot of NMA of safety profile: drop-out rate**

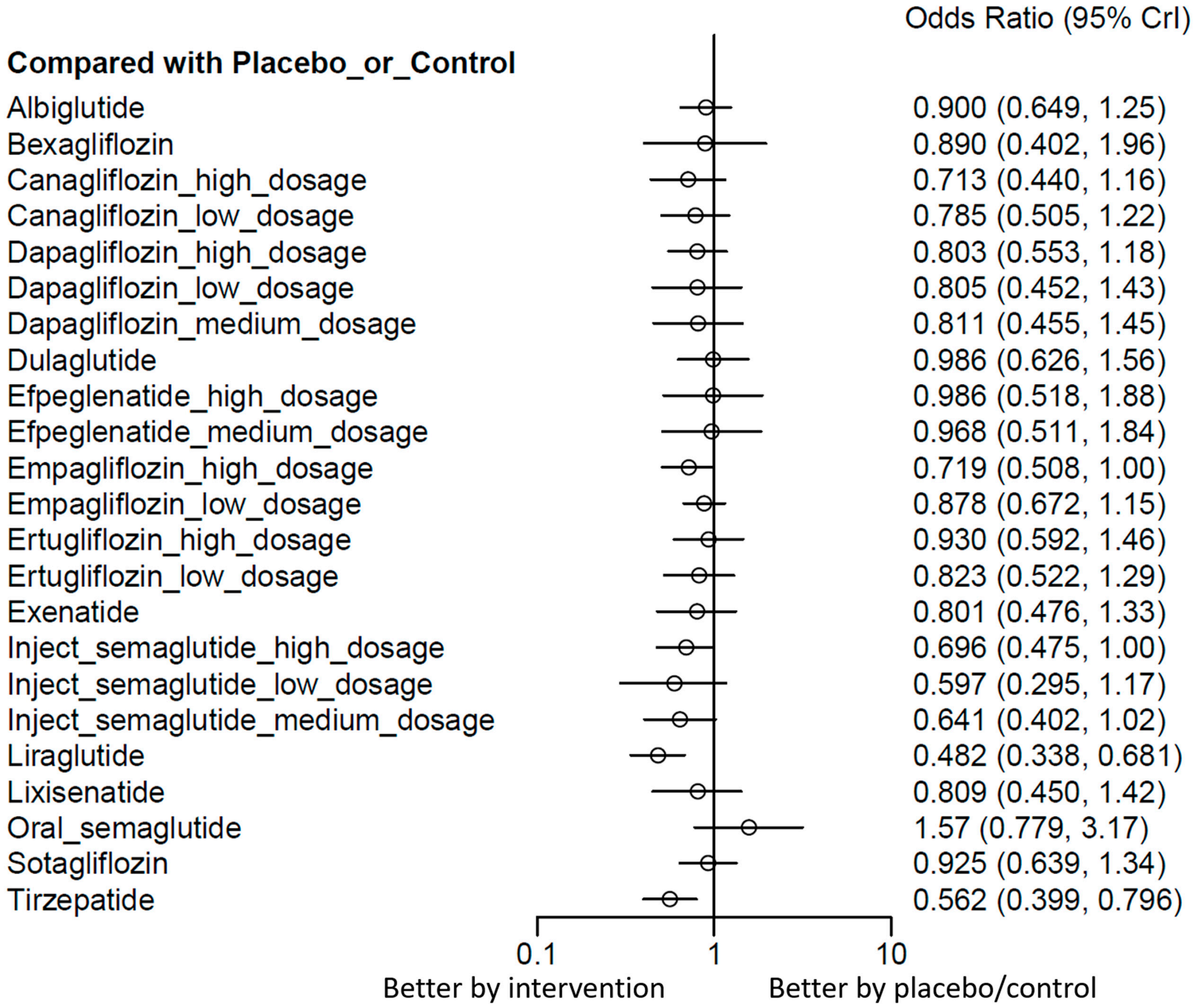

***Abbreviation for Figure S4A-E:***

*95%CIs: 95% confidence intervals; GLP-1 agonist: glucagon-like peptide-1 agonist; NMA: network meta-analysis; OR: odds ratio; RCT: randomized controlled trial; SGLT2 inhibitor: sodium–glucose cotransporter 2 inhibitor*

**Figure S5A Bayesian-based Litmus Rank-O-Gram rank plot of primary outcome: overall hematologic malignancy**

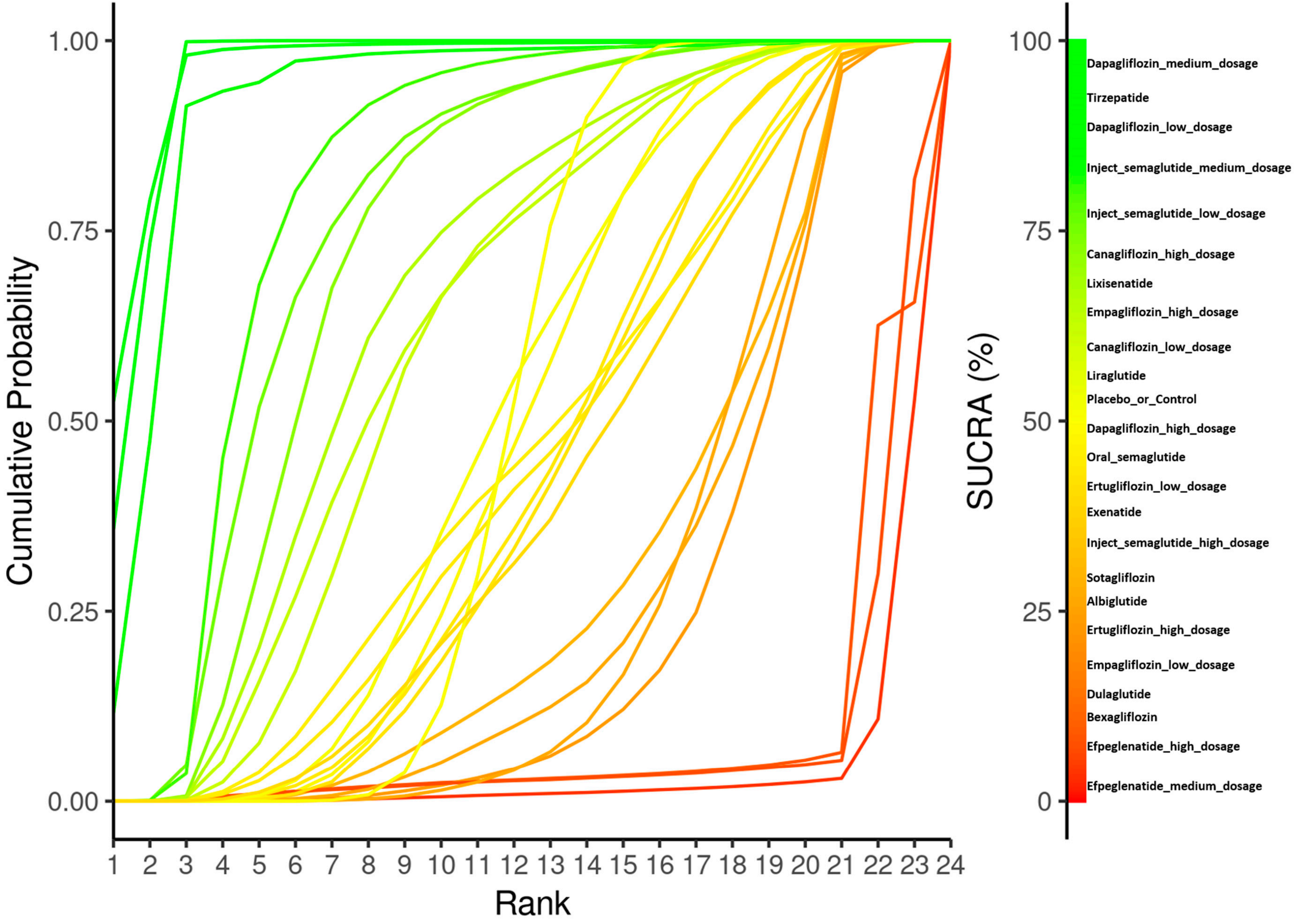

**Figure S5B Bayesian-based radial surface under the cumulative ranking of primary outcome: overall hematologic malignancy**

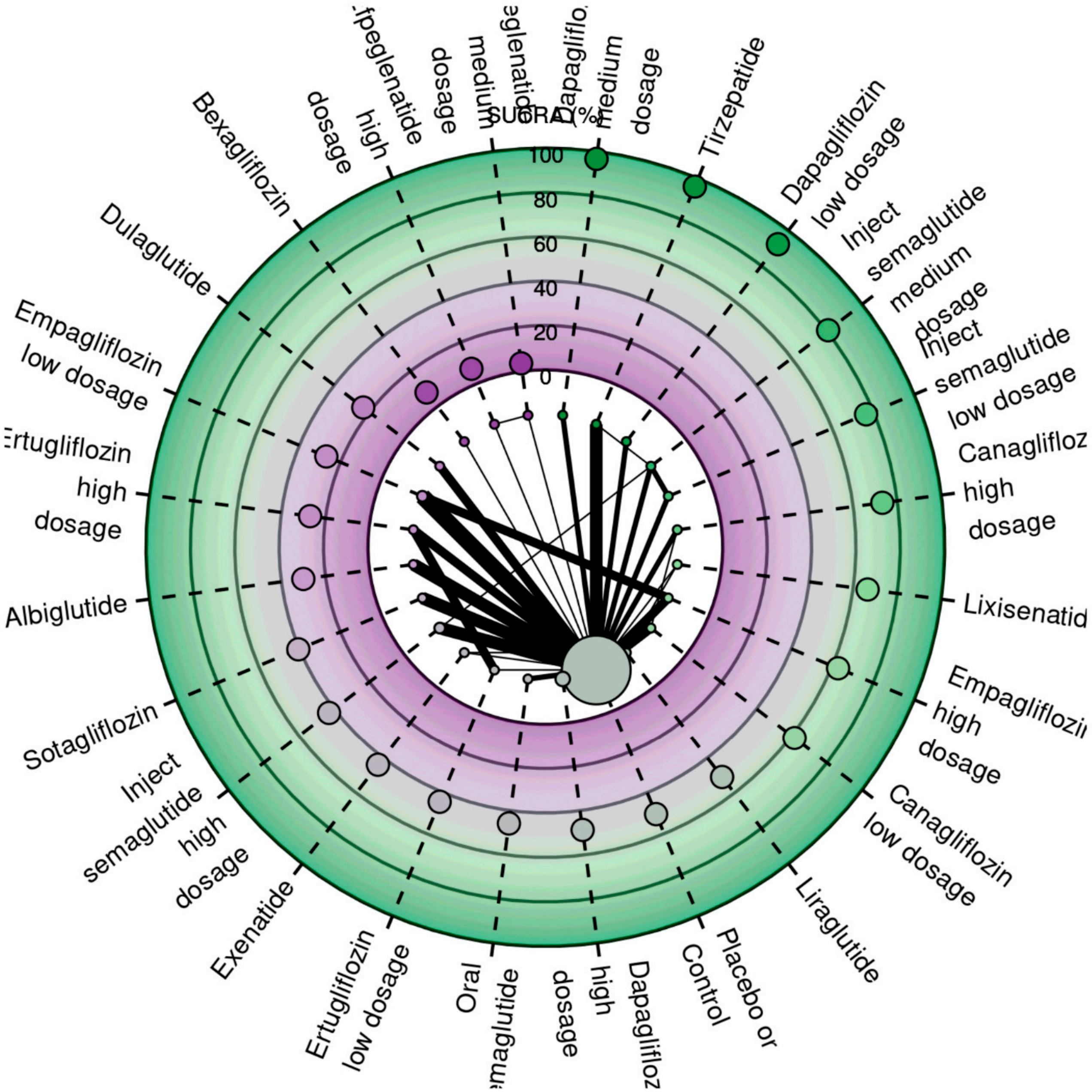

**Figure S5C Bayesian-based Litmus Rank-O-Gram rank plot of primary outcome: subgroup of lymphoma**

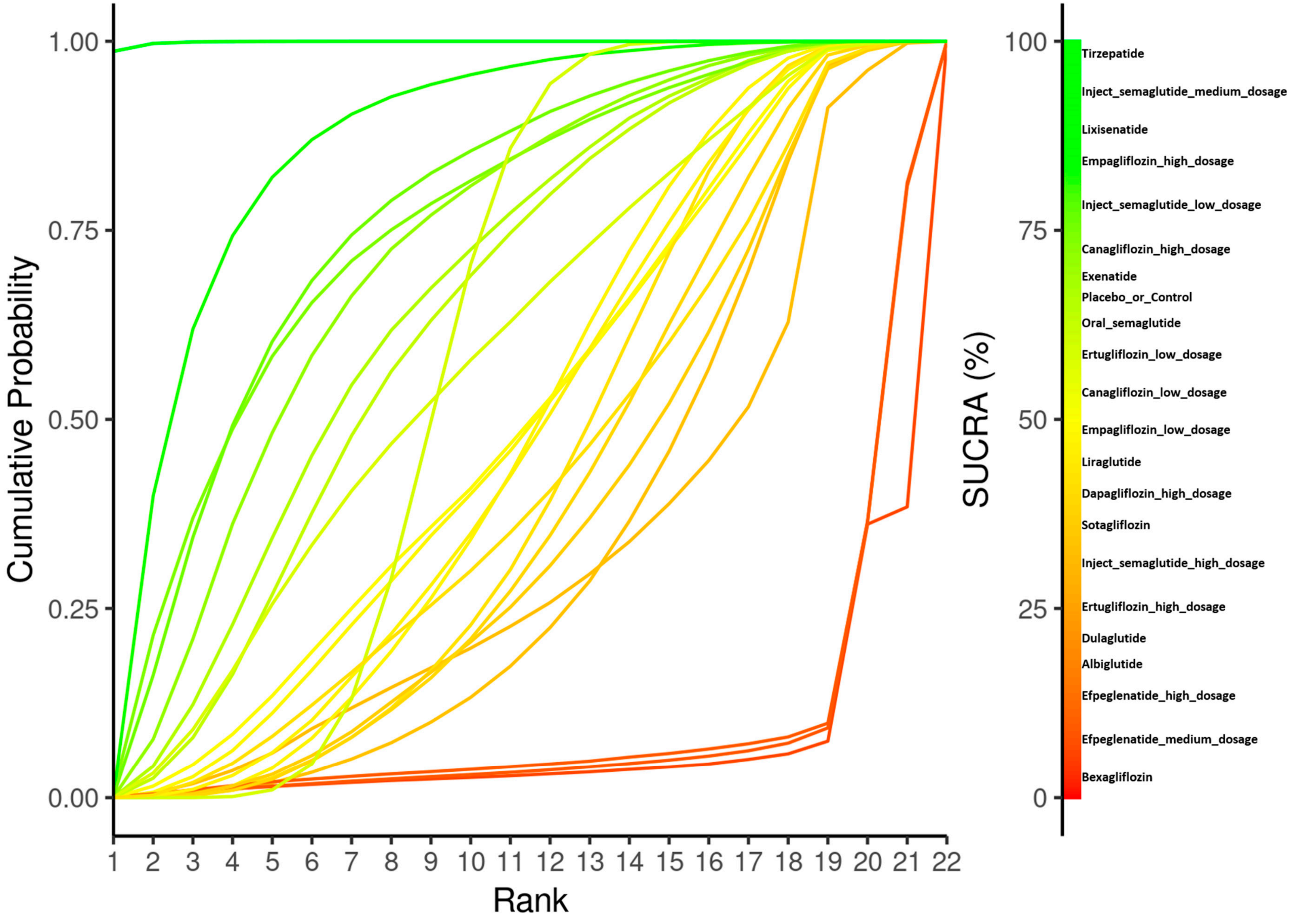

**Figure S5D Bayesian-based radial surface under the cumulative ranking of primary outcome: subgroup of lymphoma**

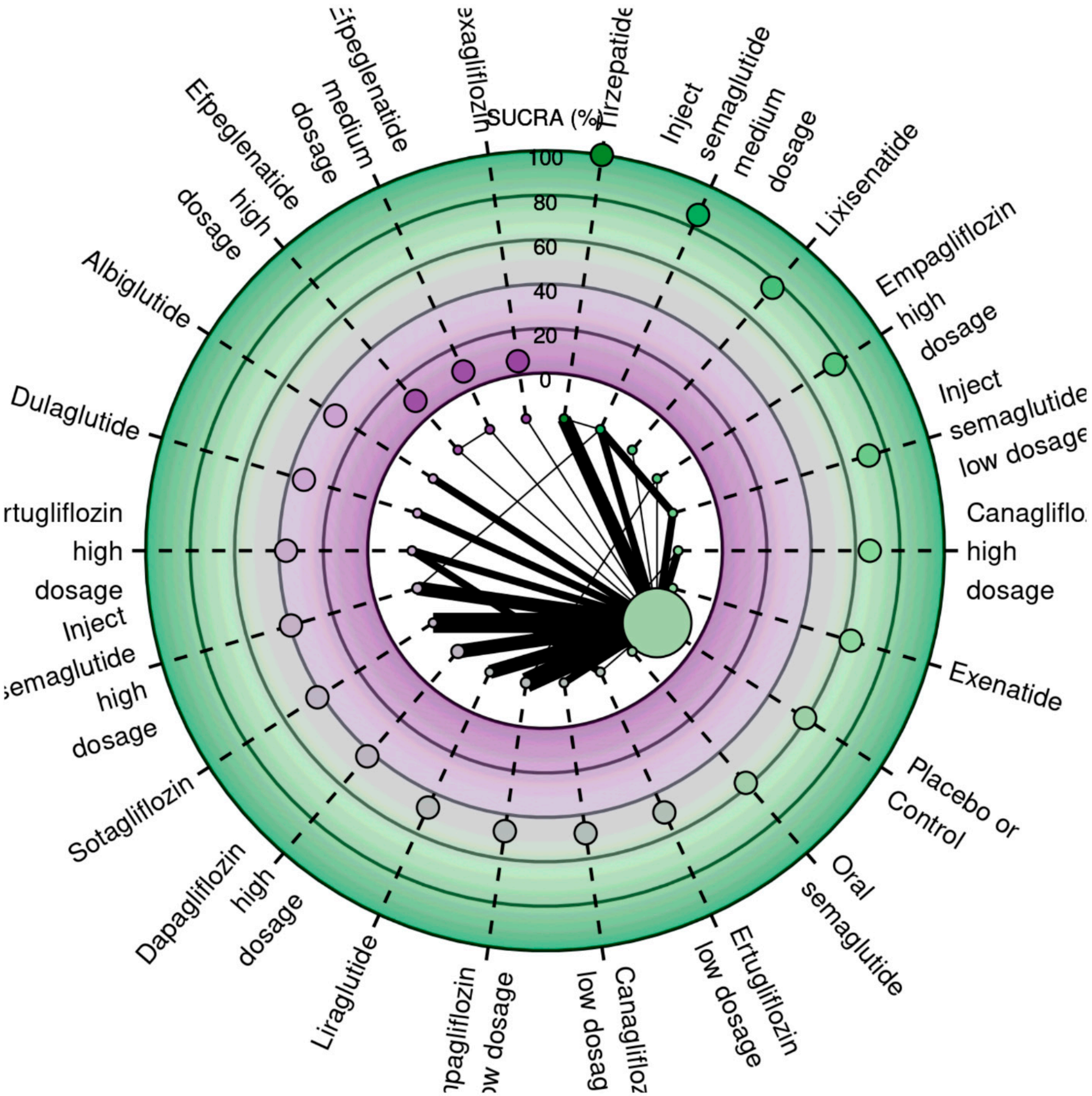

**Figure S5E Bayesian-based Litmus Rank-O-Gram rank plot of primary outcome: subgroup of leukemia**

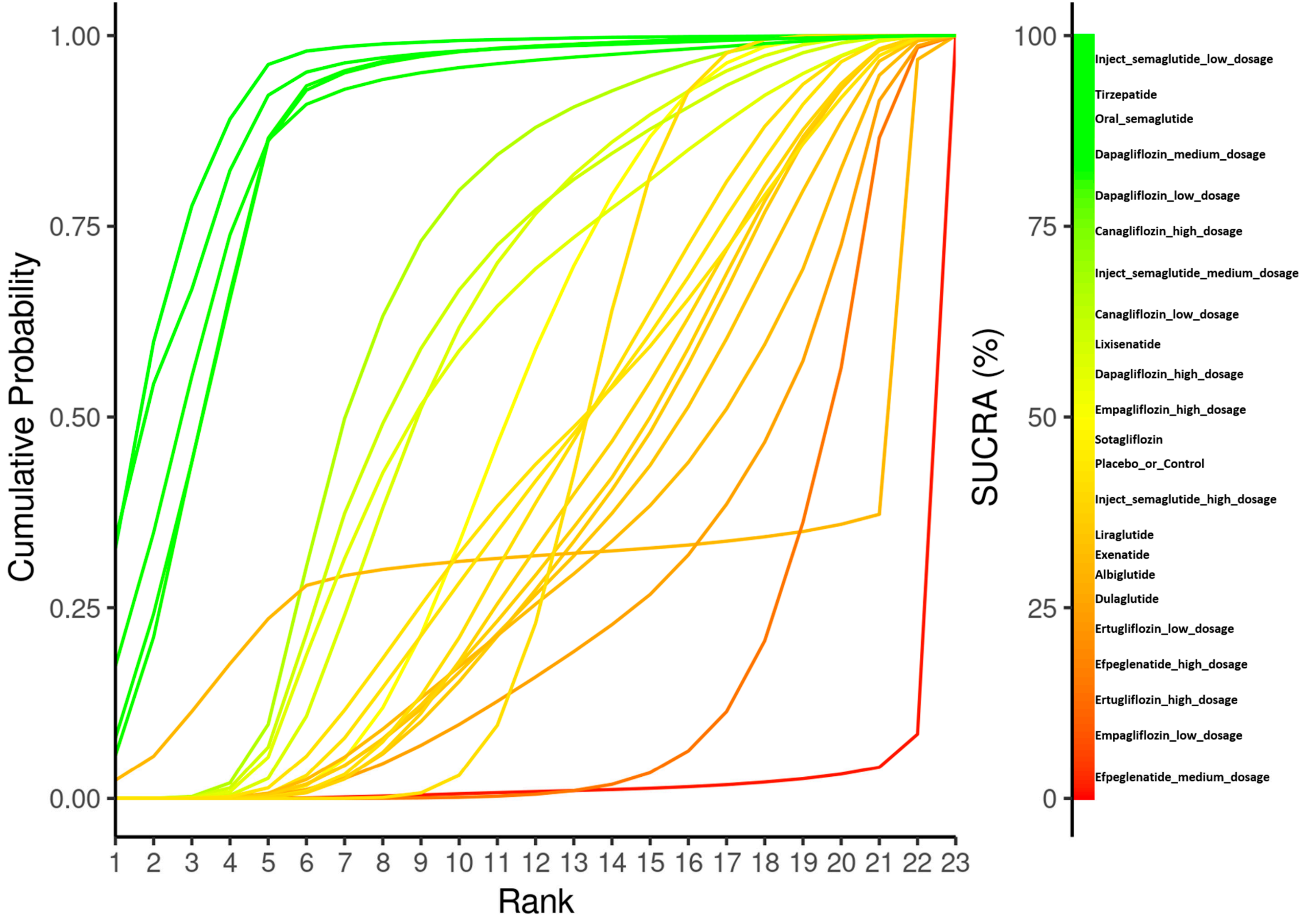

**Figure S5F Bayesian-based radial surface under the cumulative ranking of primary outcome: subgroup of leukemia**

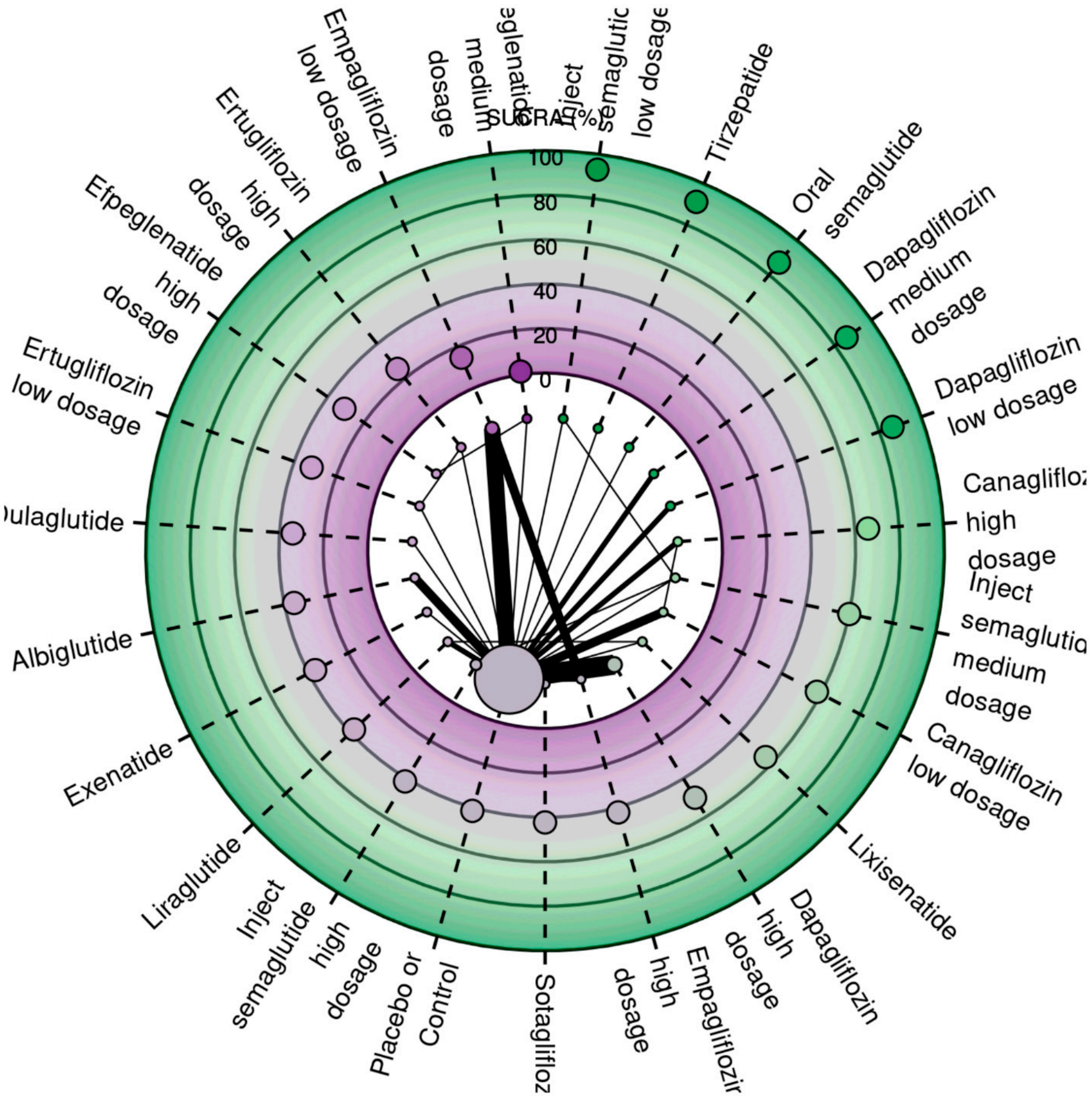

**Figure S5G Bayesian-based Litmus Rank-O-Gram rank plot of primary outcome: subgroup of myeloma**

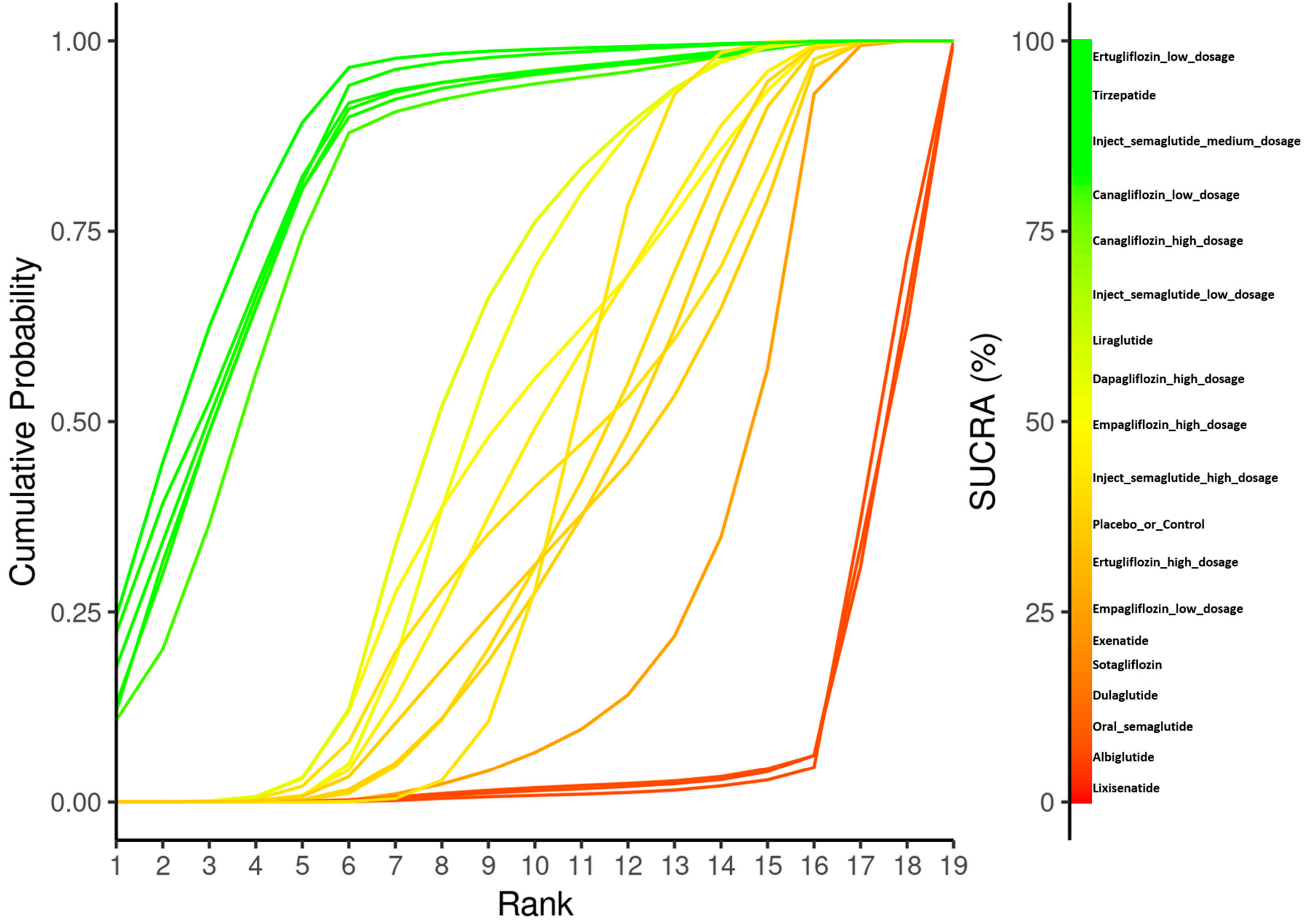

**Figure S5H Bayesian-based radial surface under the cumulative ranking of primary outcome: subgroup of myeloma**

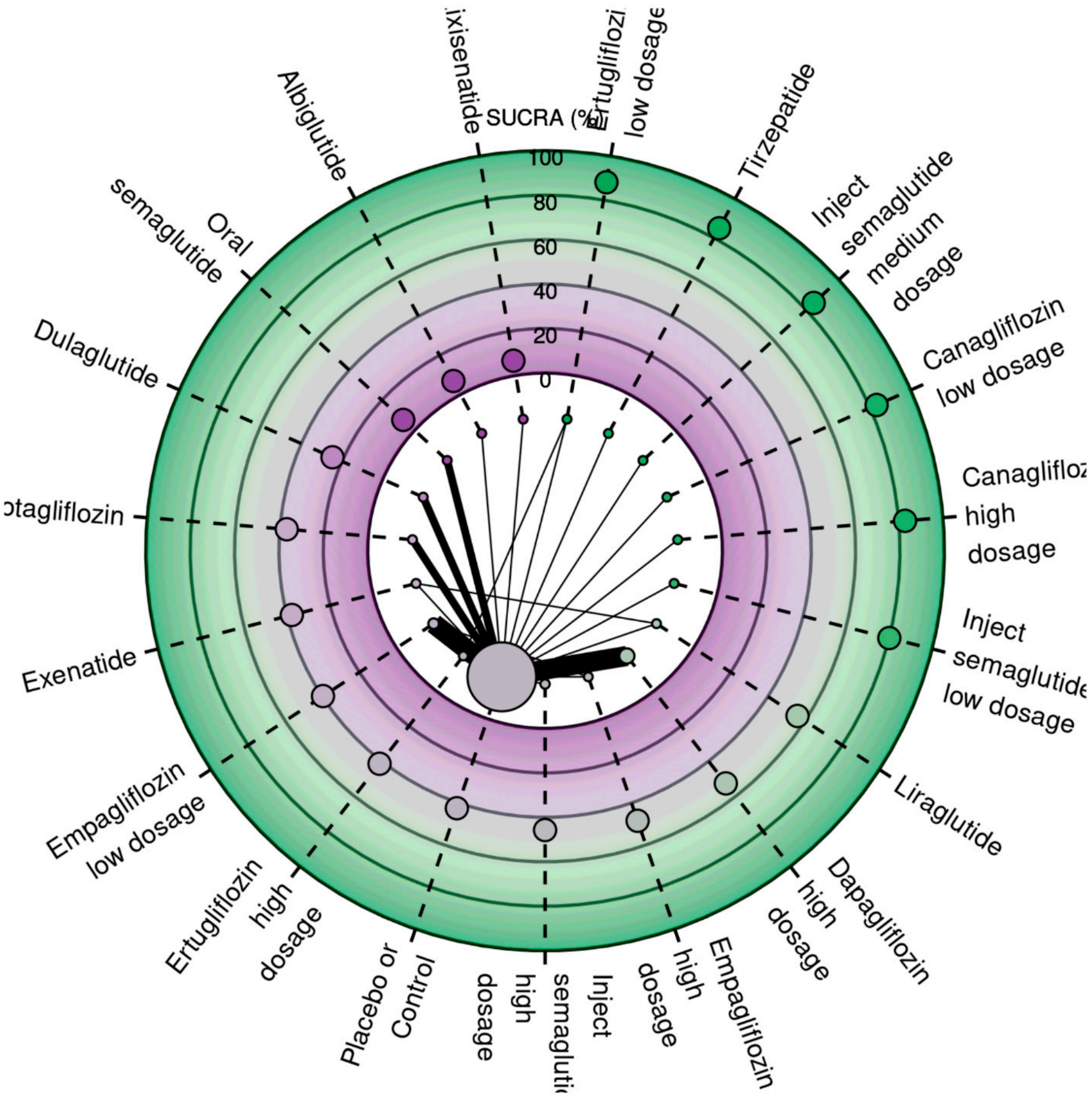

**Figure S5I Bayesian-based Litmus Rank-O-Gram rank plot of safety profile: drop-out rate**

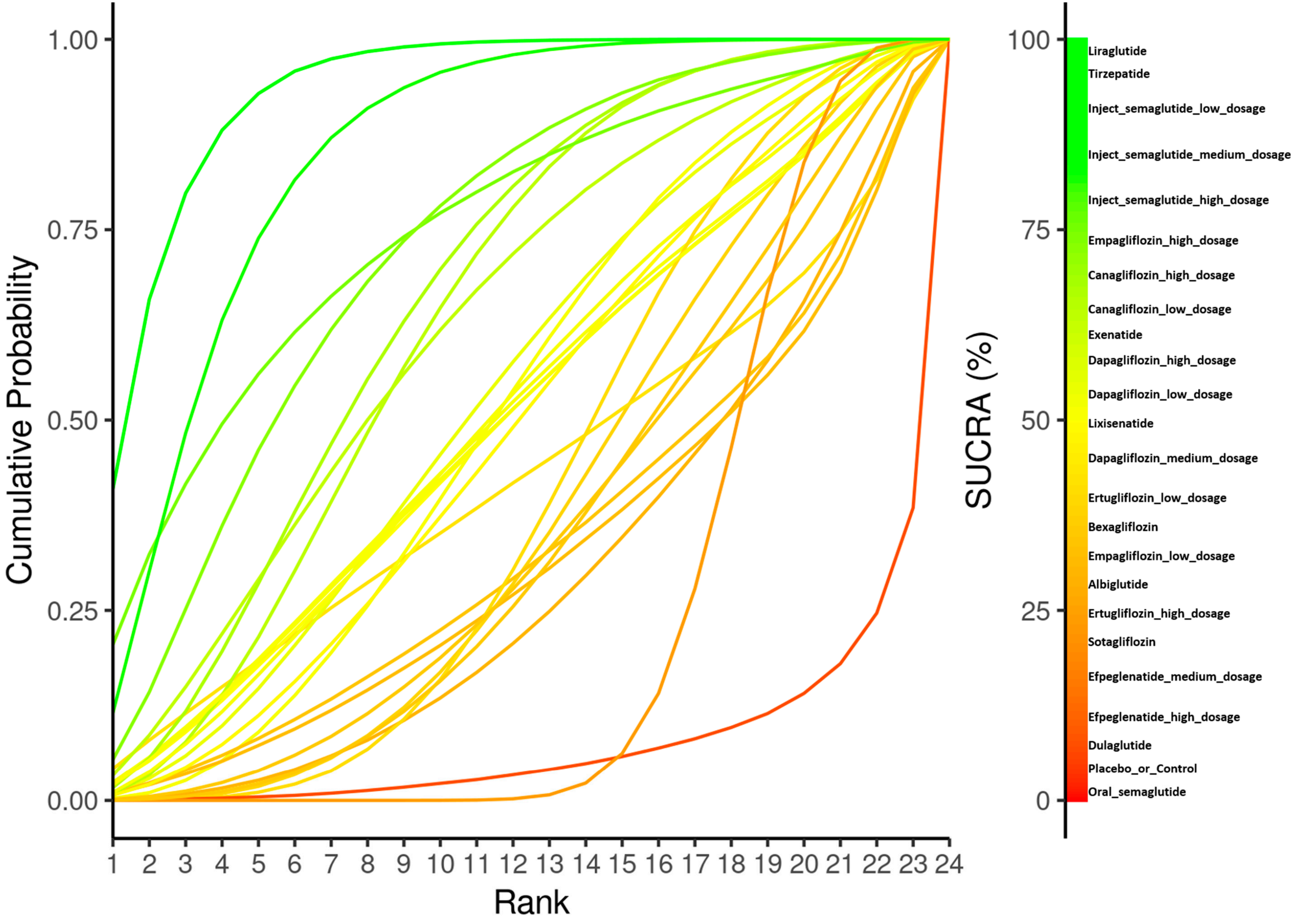

**Figure S5J Bayesian-based radial surface under the cumulative ranking of safety profile: drop-out rate**

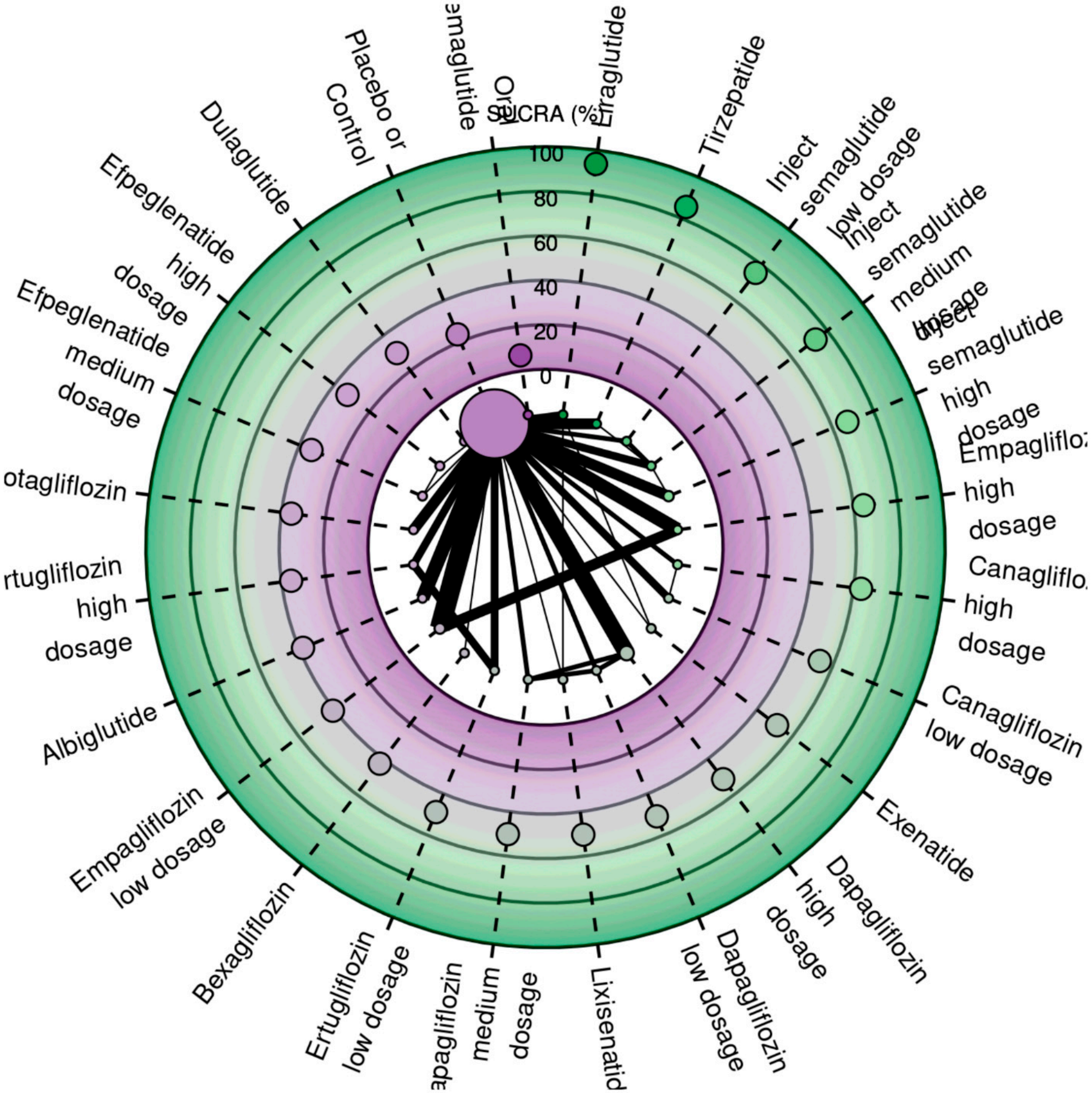

***Abbreviation for Figure S5A-J:***

*95%CIs: 95% confidence intervals; GLP-1 agonist: glucagon-like peptide-1 agonist; NMA: network meta-analysis; OR: odds ratio; RCT: randomized controlled trial; SGLT2 inhibitor: sodium–glucose cotransporter 2 inhibitor*

**Figure S6A Bayesian-based residual deviance NMA/UME model of primary outcome: overall hematologic malignancy**

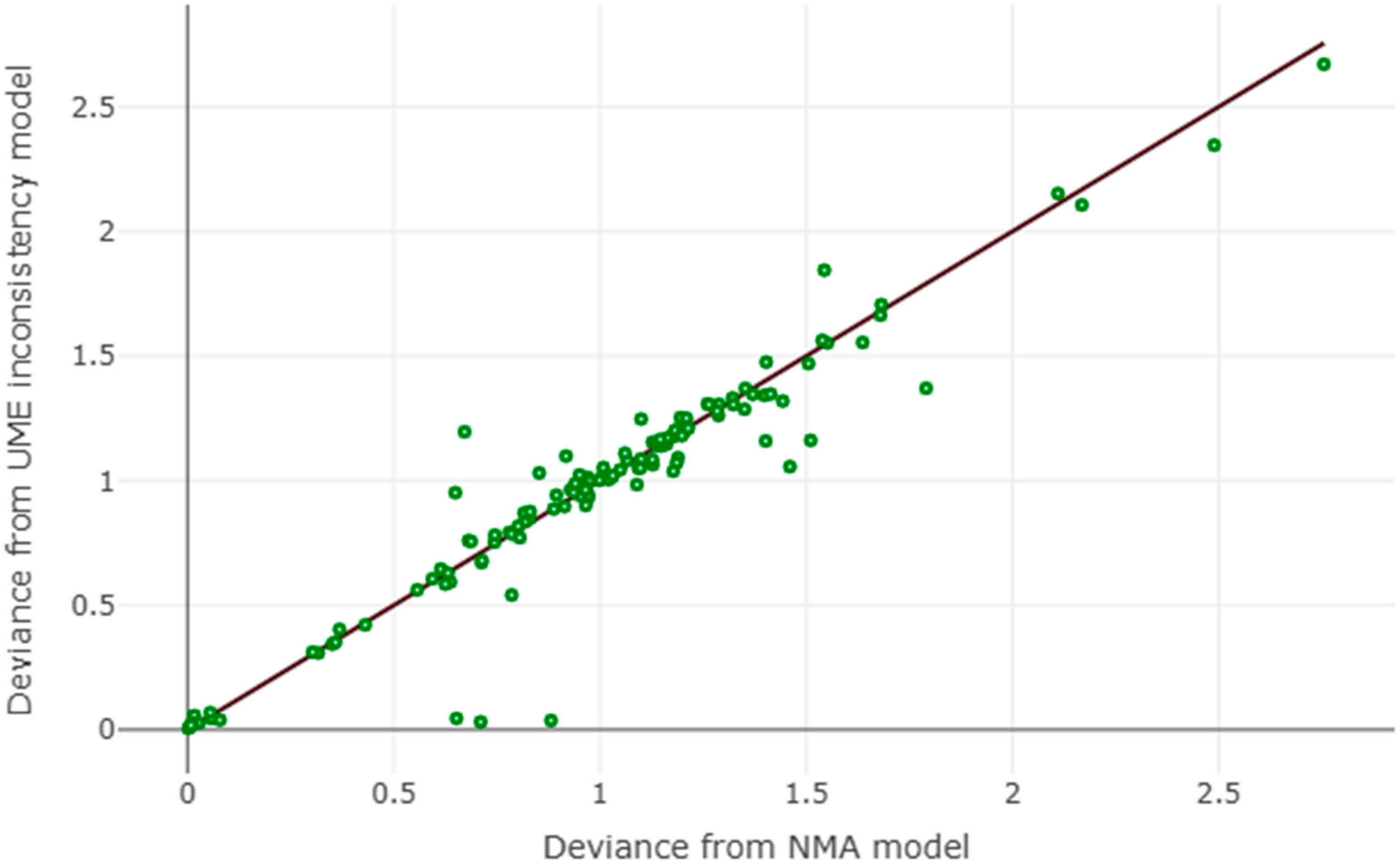

**Figure S6B Bayesian-based per-arm residual deviance of primary outcome: overall hematologic malignancy**

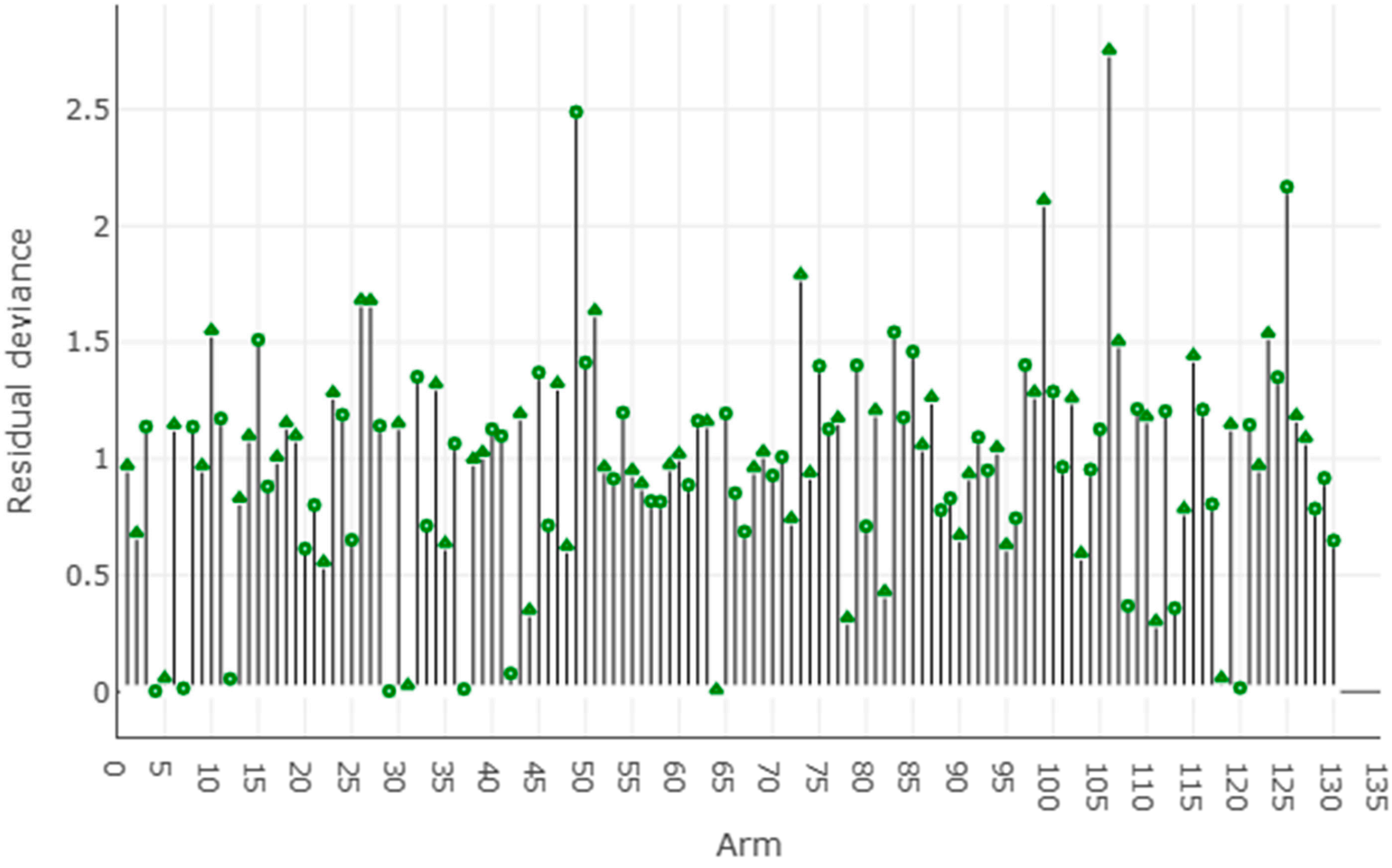

**Figure S6C Bayesian-based leverage plot of primary outcome: overall hematologic malignancy**

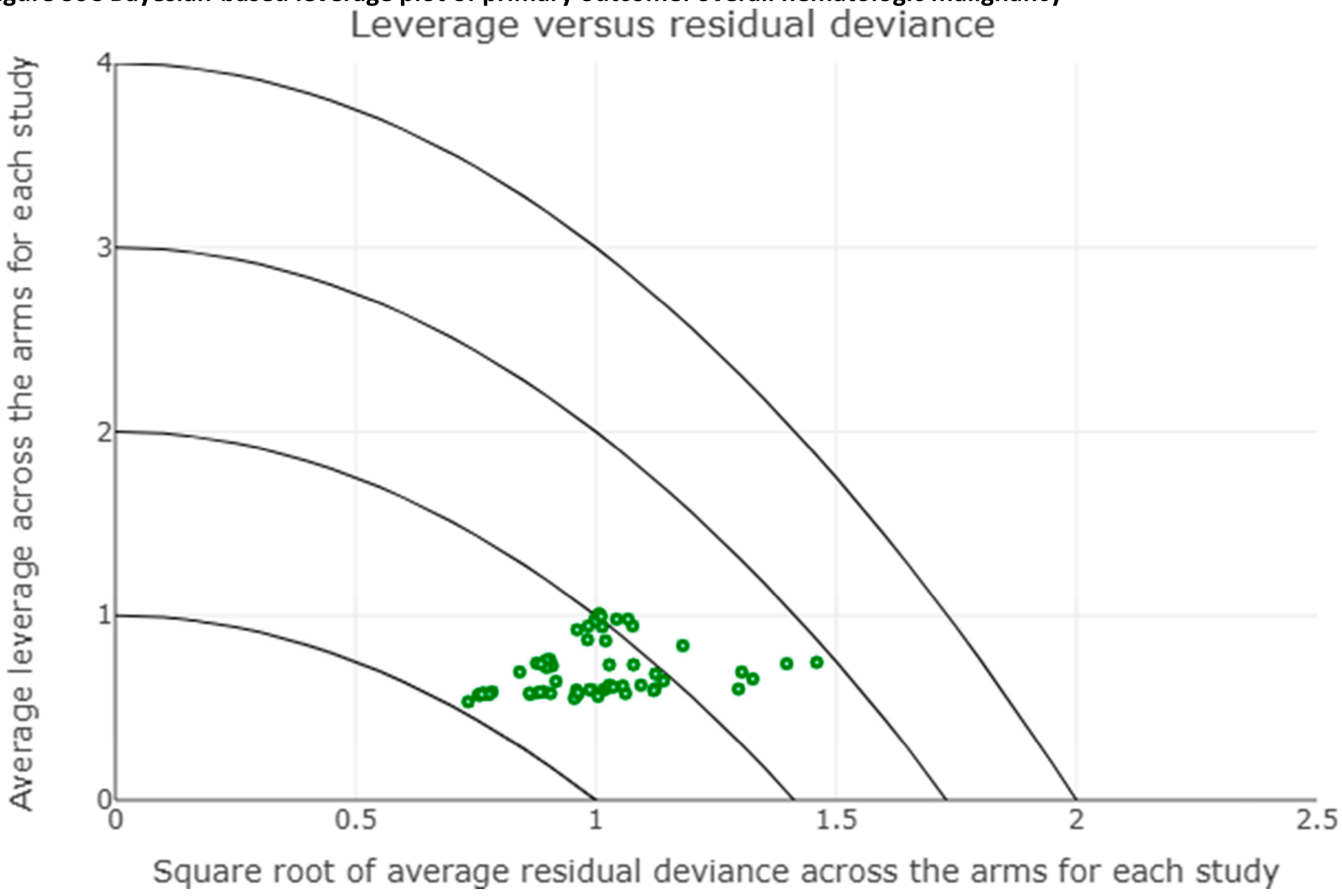

**Figure S6D Bayesian-based residual deviance NMA/UME model of primary outcome: subgroup of lymphoma**

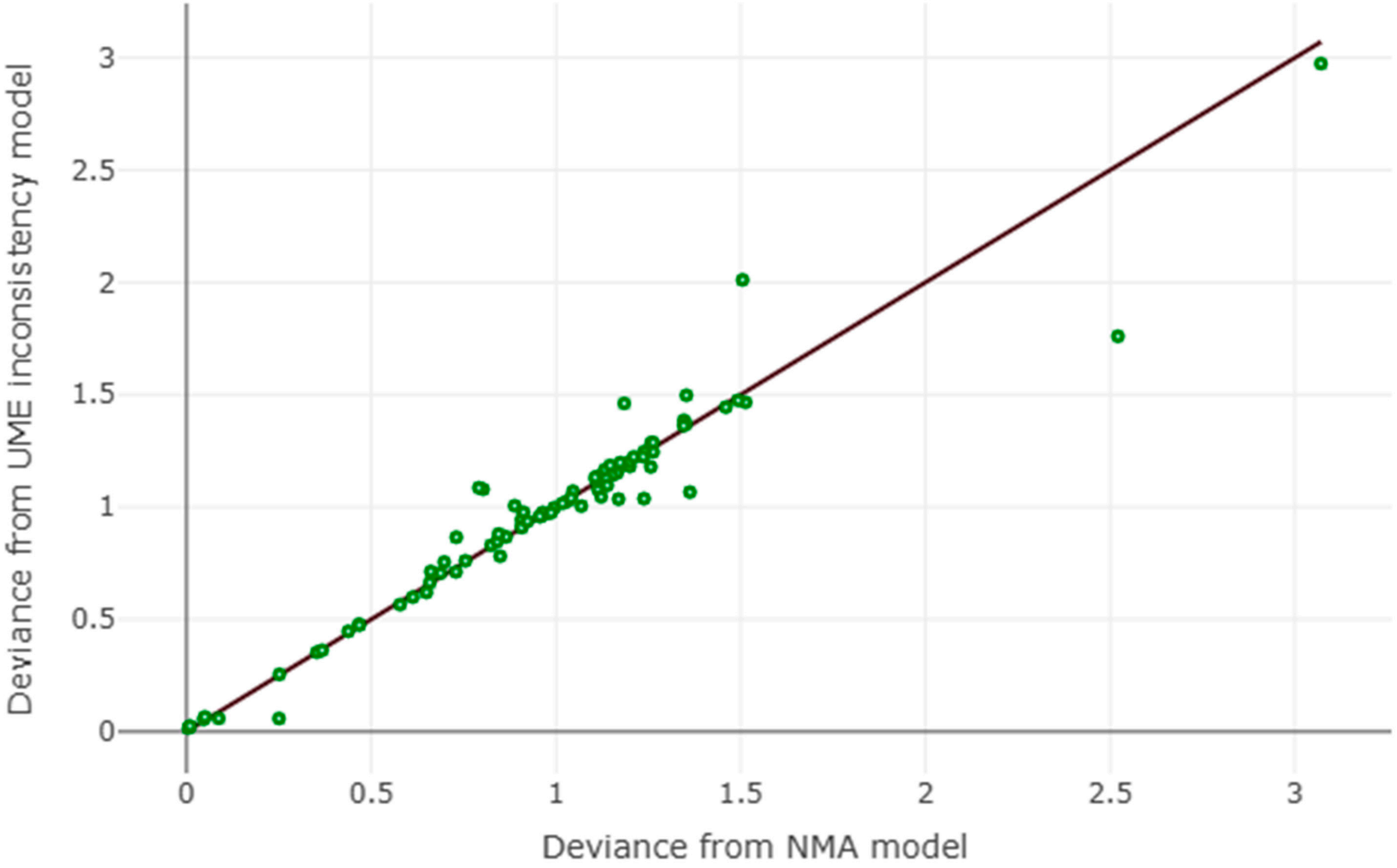

**Figure S6E Bayesian-based per-arm residual deviance of primary outcome: subgroup of lymphoma**

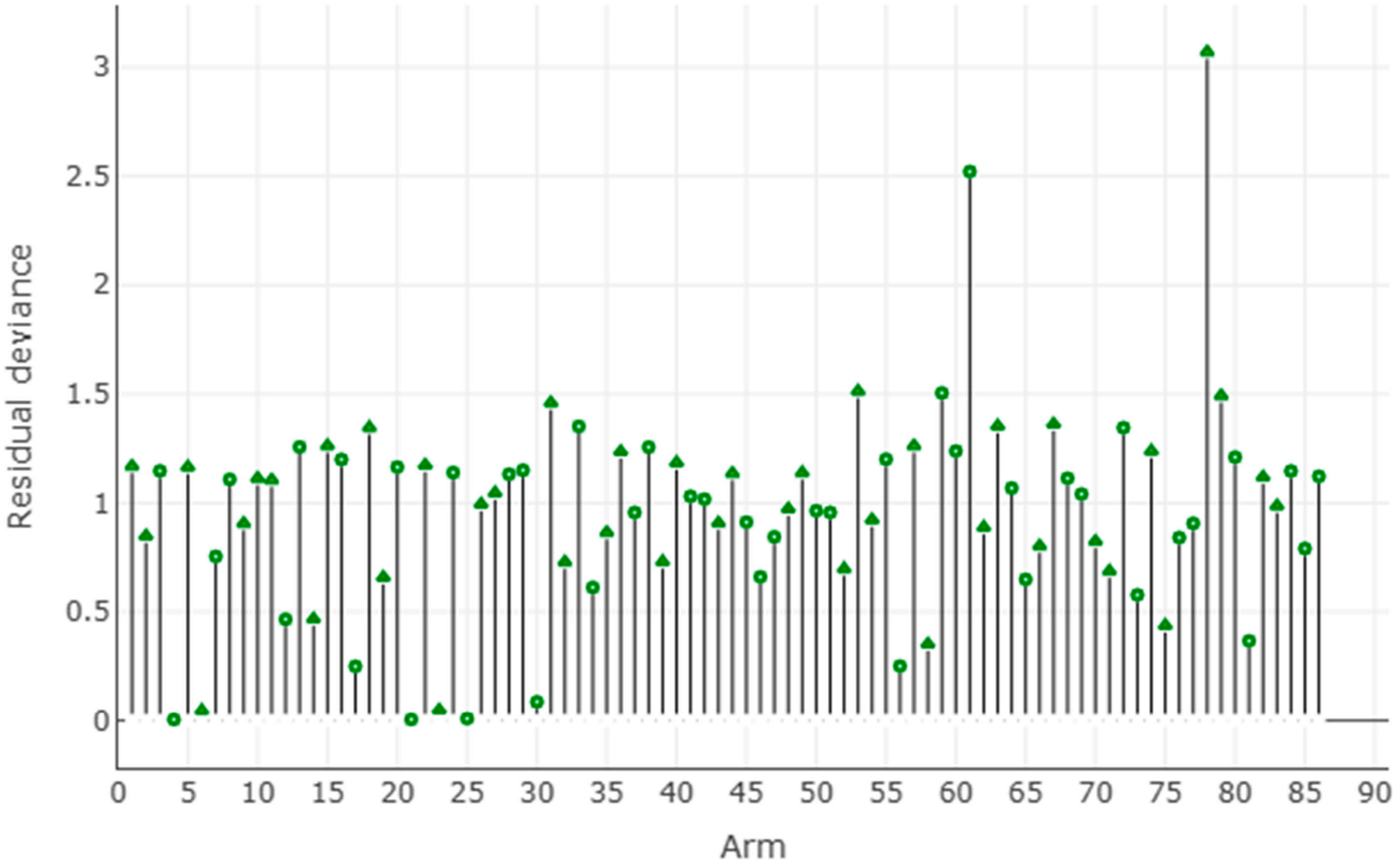

**Figure S6F Bayesian-based leverage plot of primary outcome: subgroup of lymphoma**

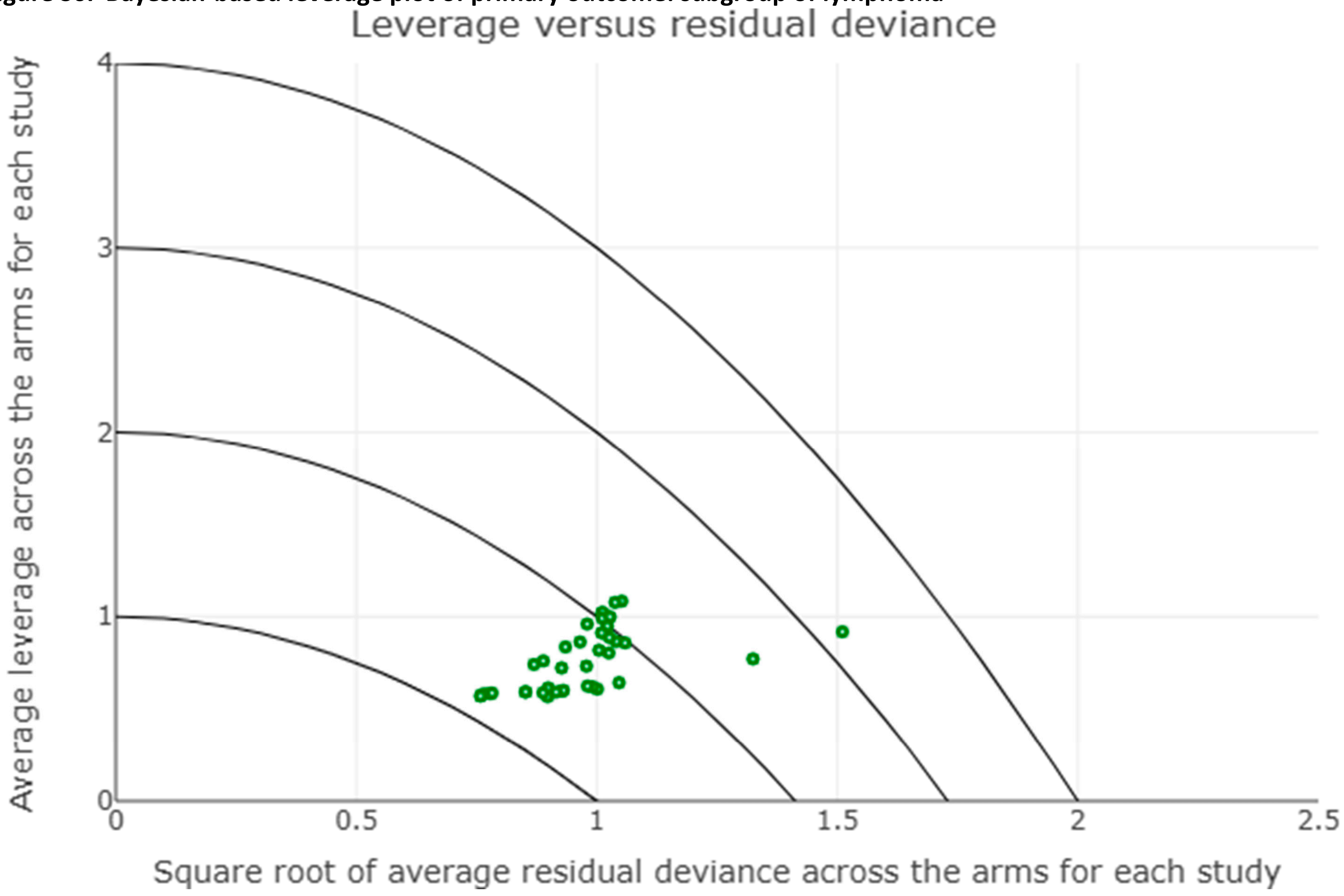

**Figure S6G Bayesian-based residual deviance NMA/UME model of primary outcome: subgroup of leukemia**

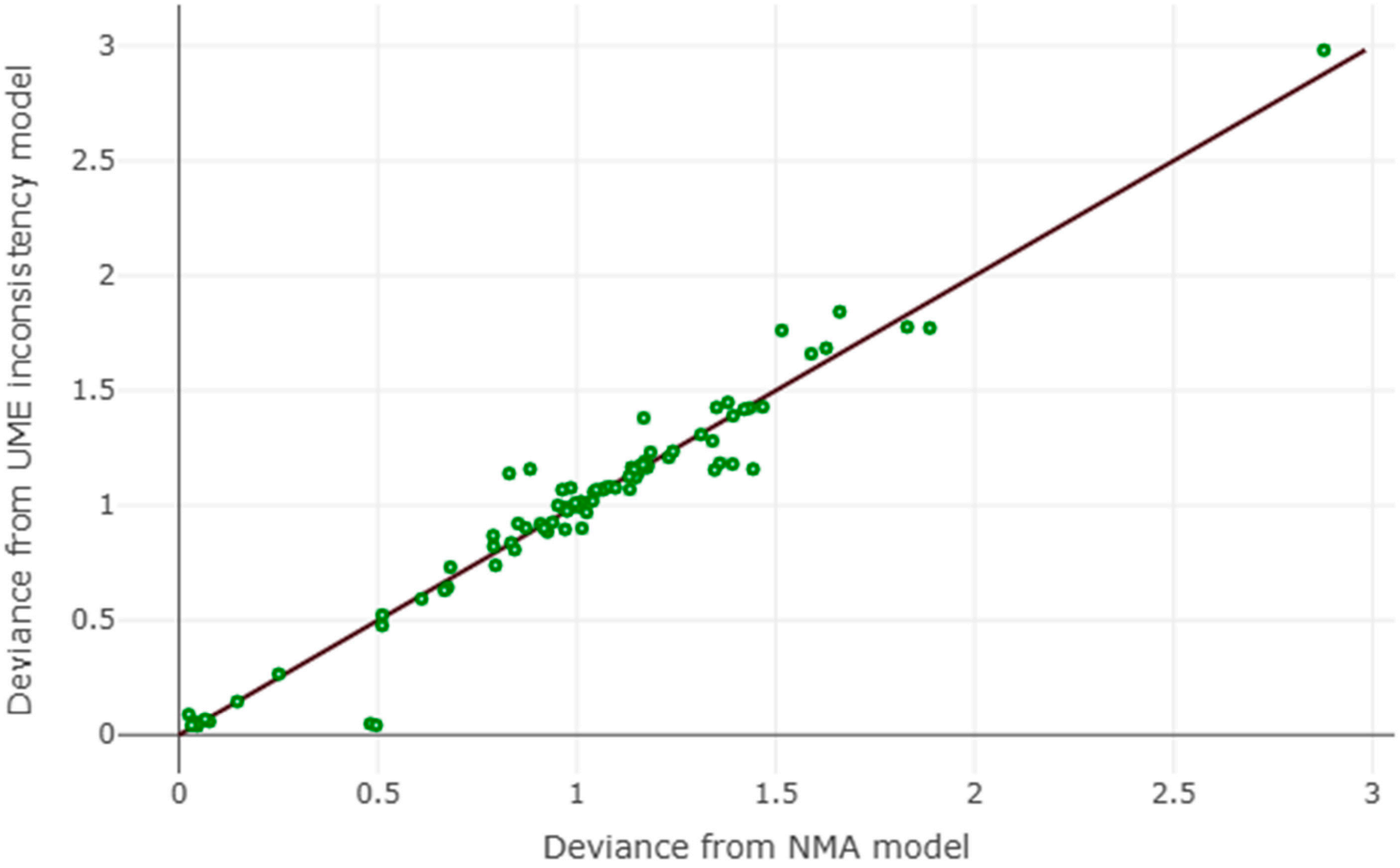

**Figure S6H Bayesian-based per-arm residual deviance of primary outcome: subgroup of leukemia**

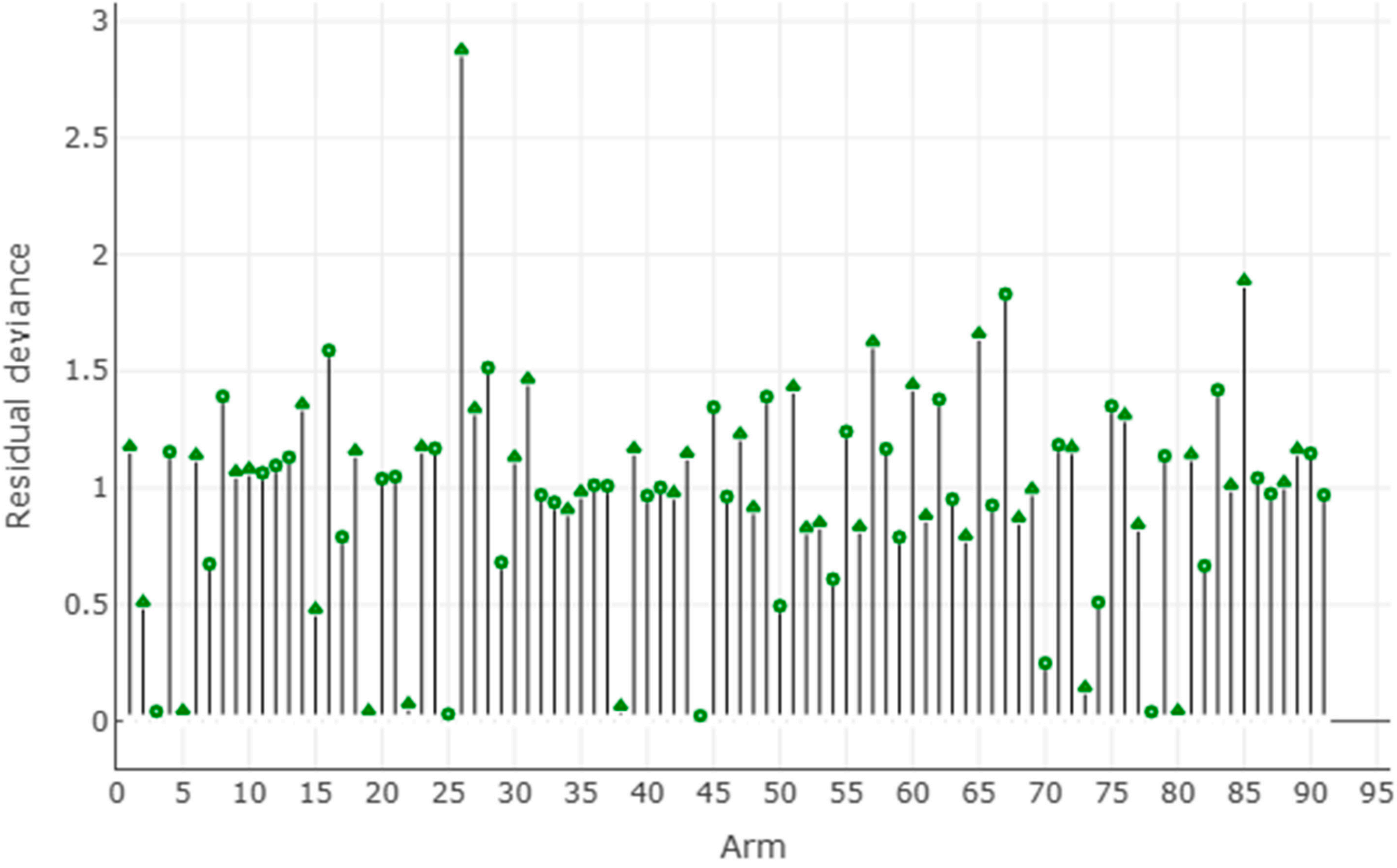

**Figure S6I Bayesian-based leverage plot of primary outcome: subgroup of leukemia**

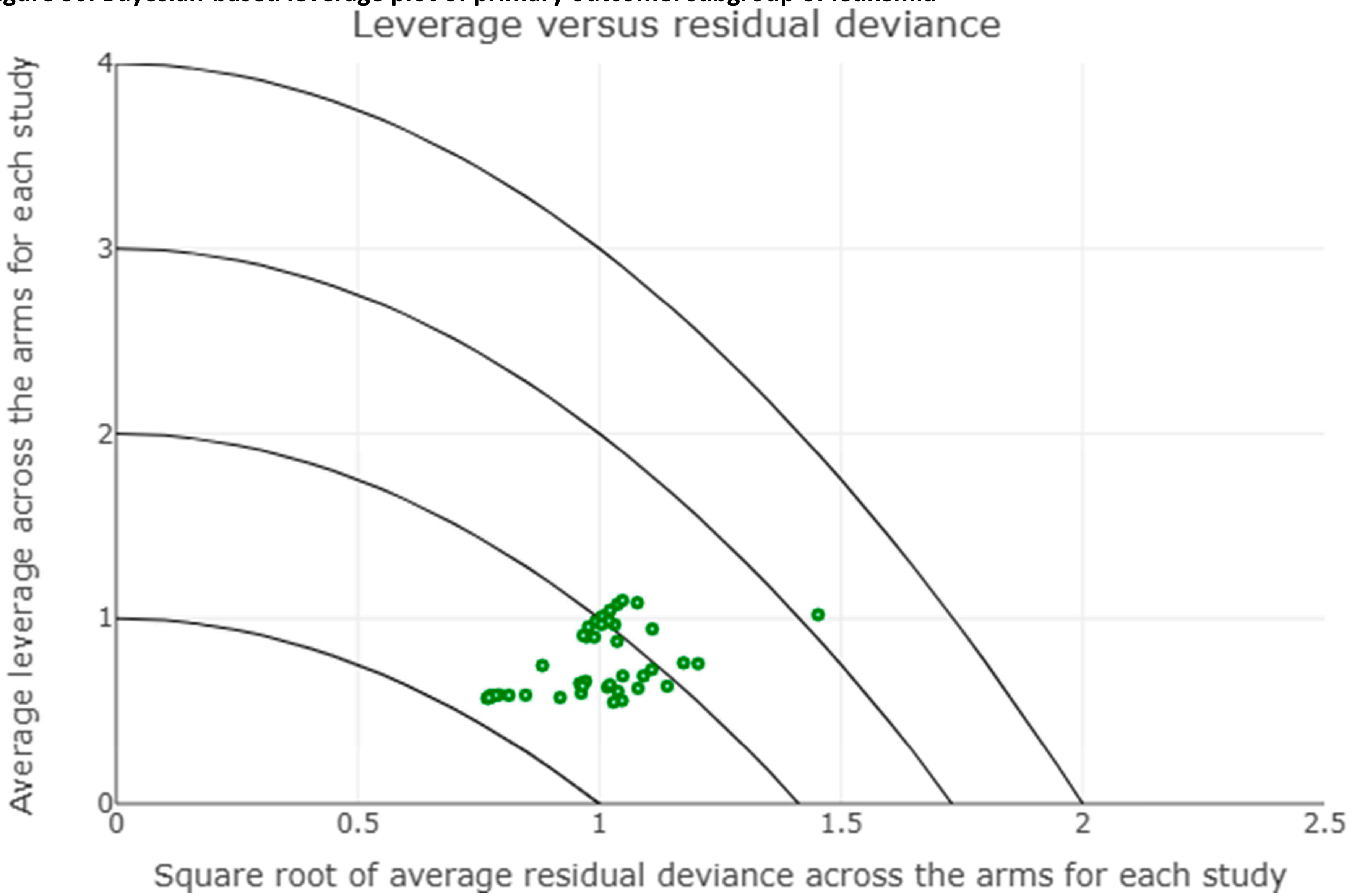

**Figure S6J Bayesian-based residual deviance NMA/UME model of primary outcome: subgroup of myeloma**

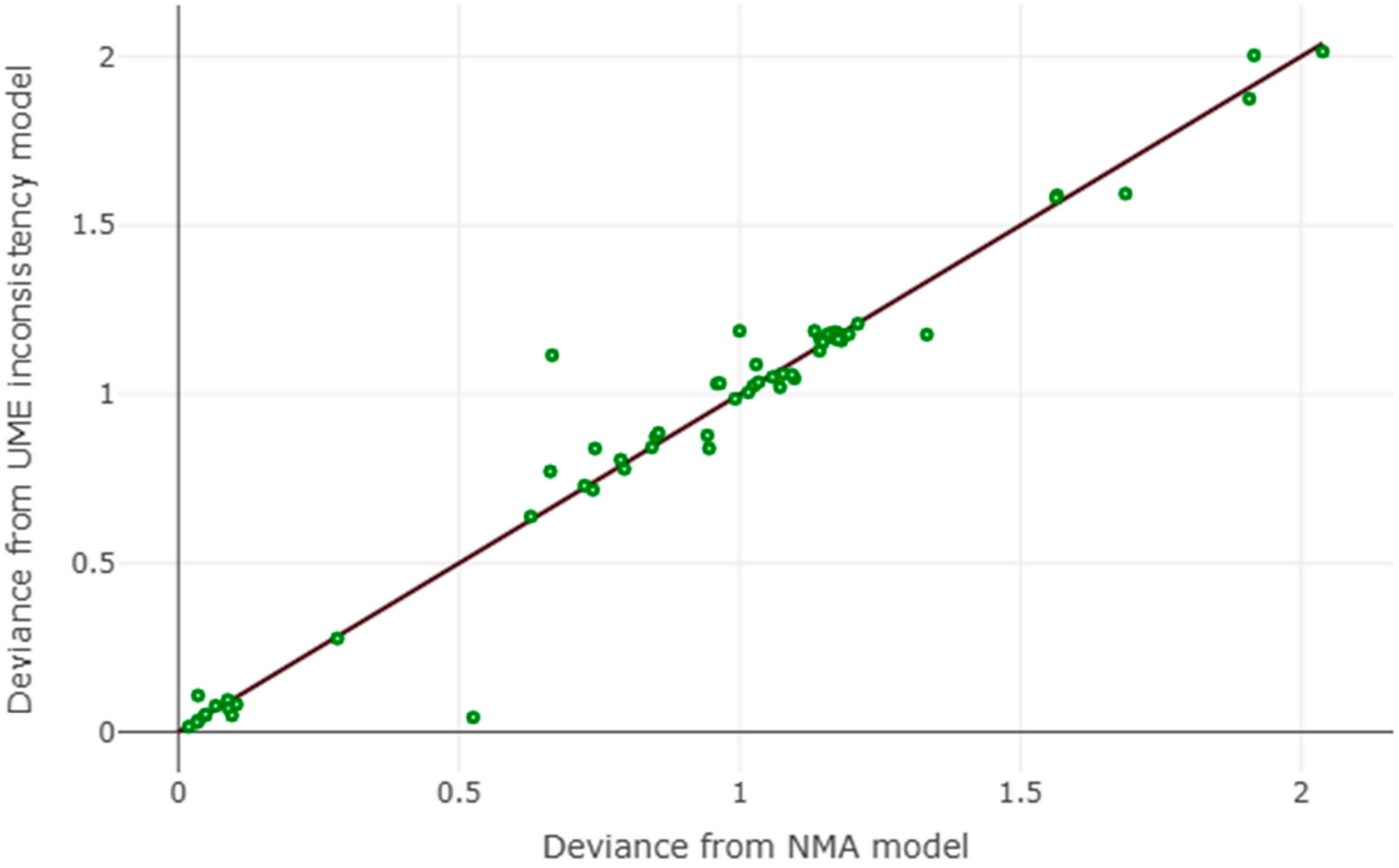

**Figure S6K Bayesian-based per-arm residual deviance of primary outcome: subgroup of myeloma**

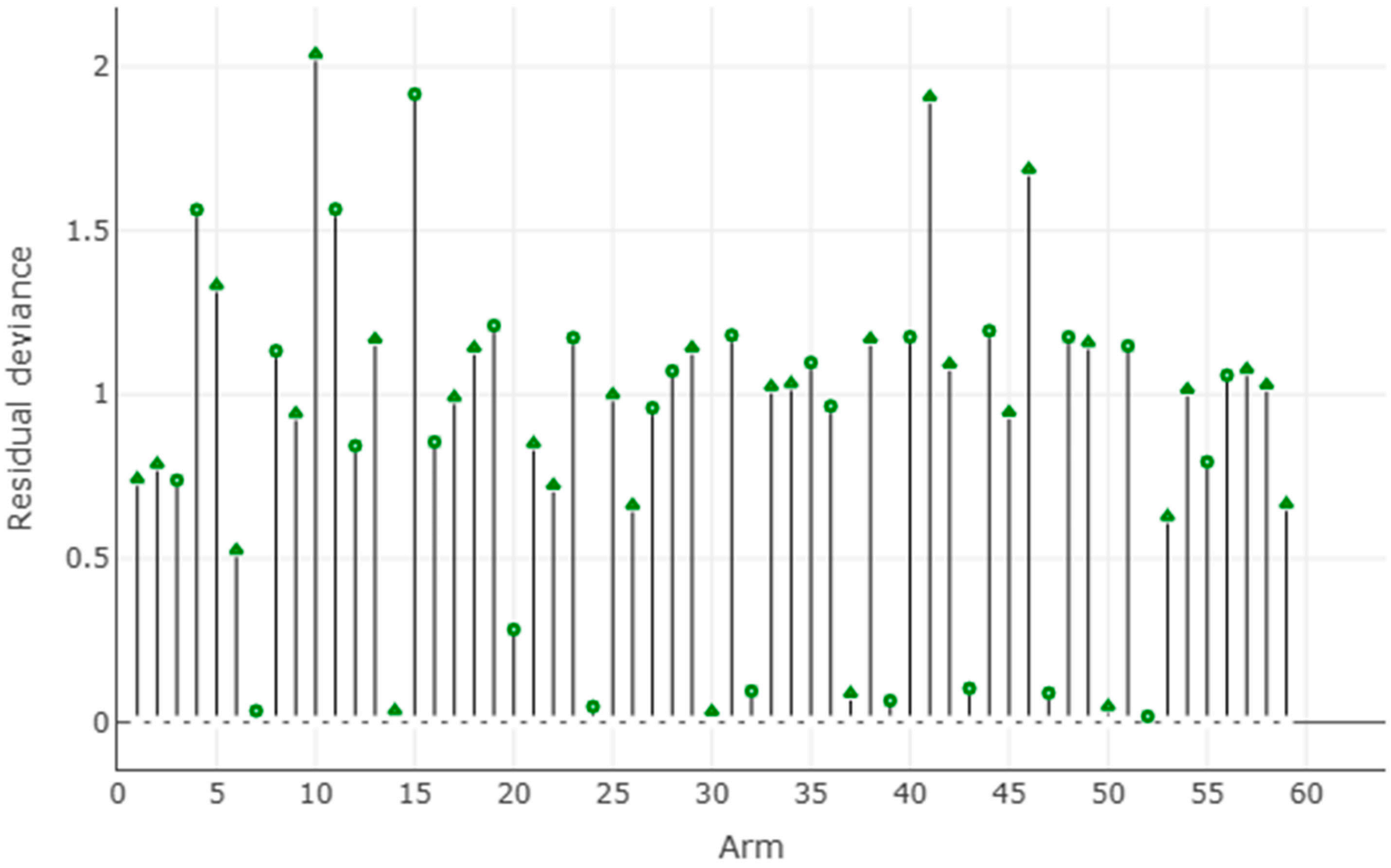

**Figure S6L Bayesian-based leverage plot of primary outcome: subgroup of myeloma**

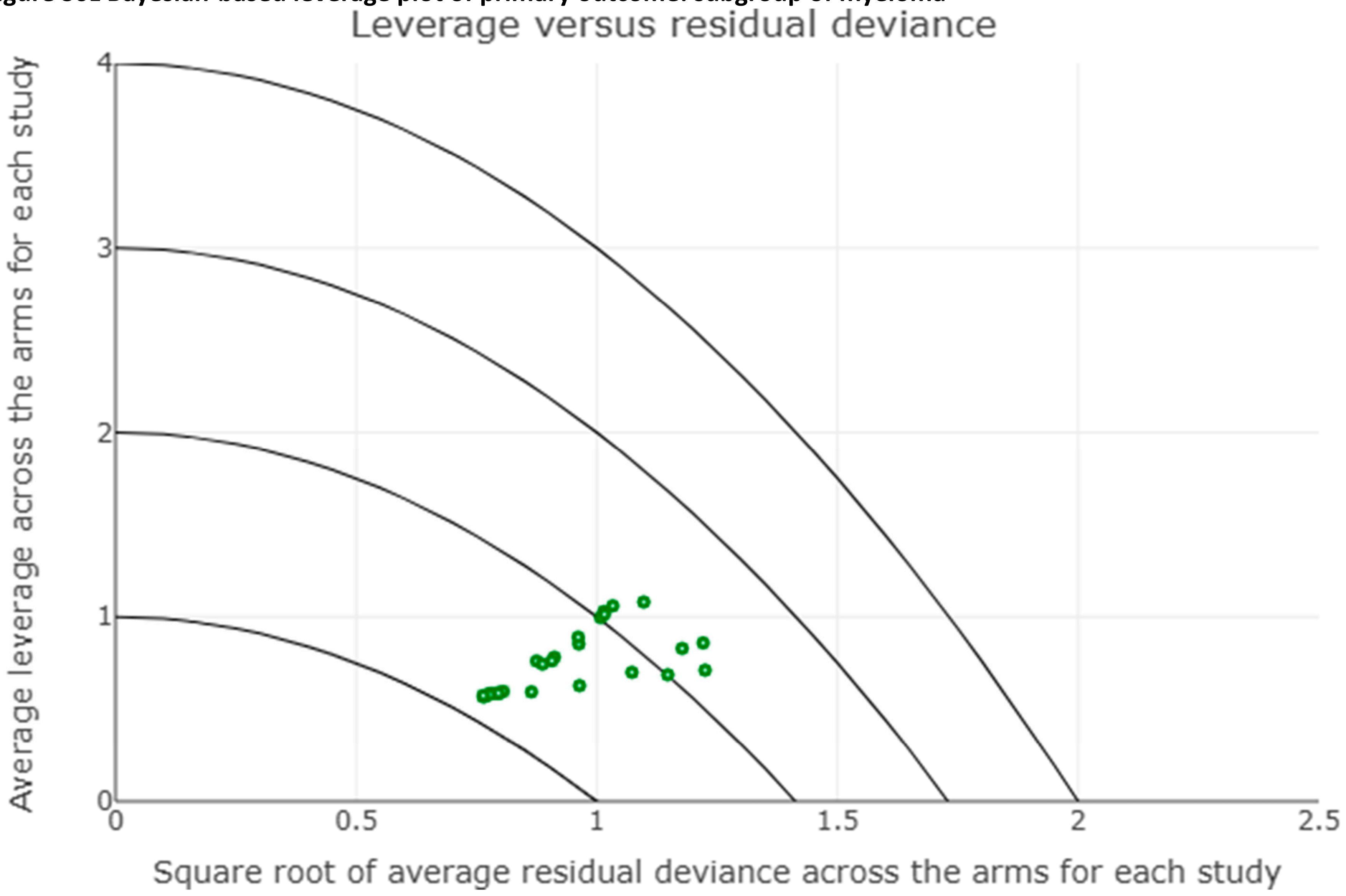

**Figure S6M Bayesian-based residual deviance NMA/UME model of safety profile: drop-out rate**

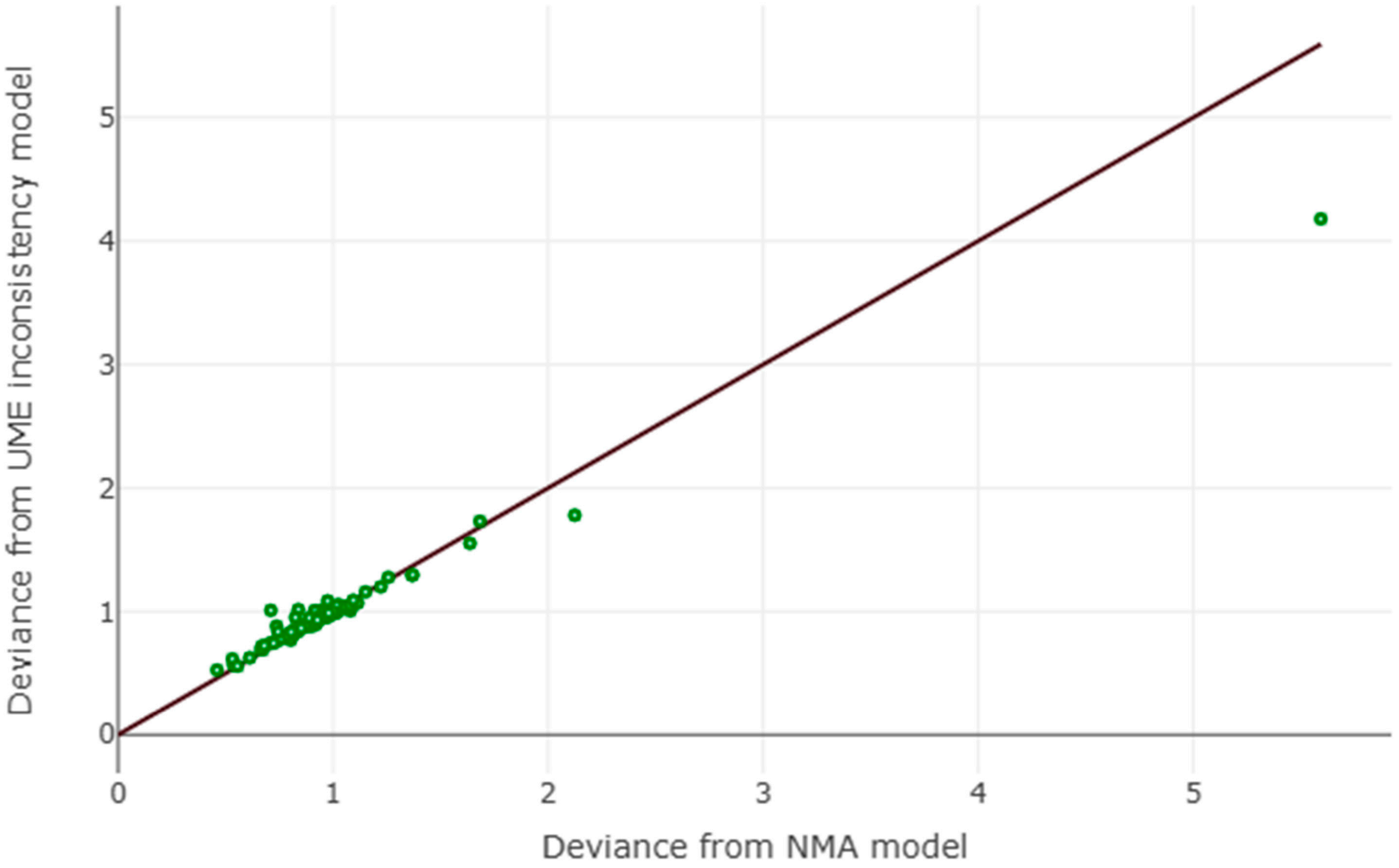

**Figure S6N Bayesian-based per-arm residual deviance of safety profile: drop-out rate**

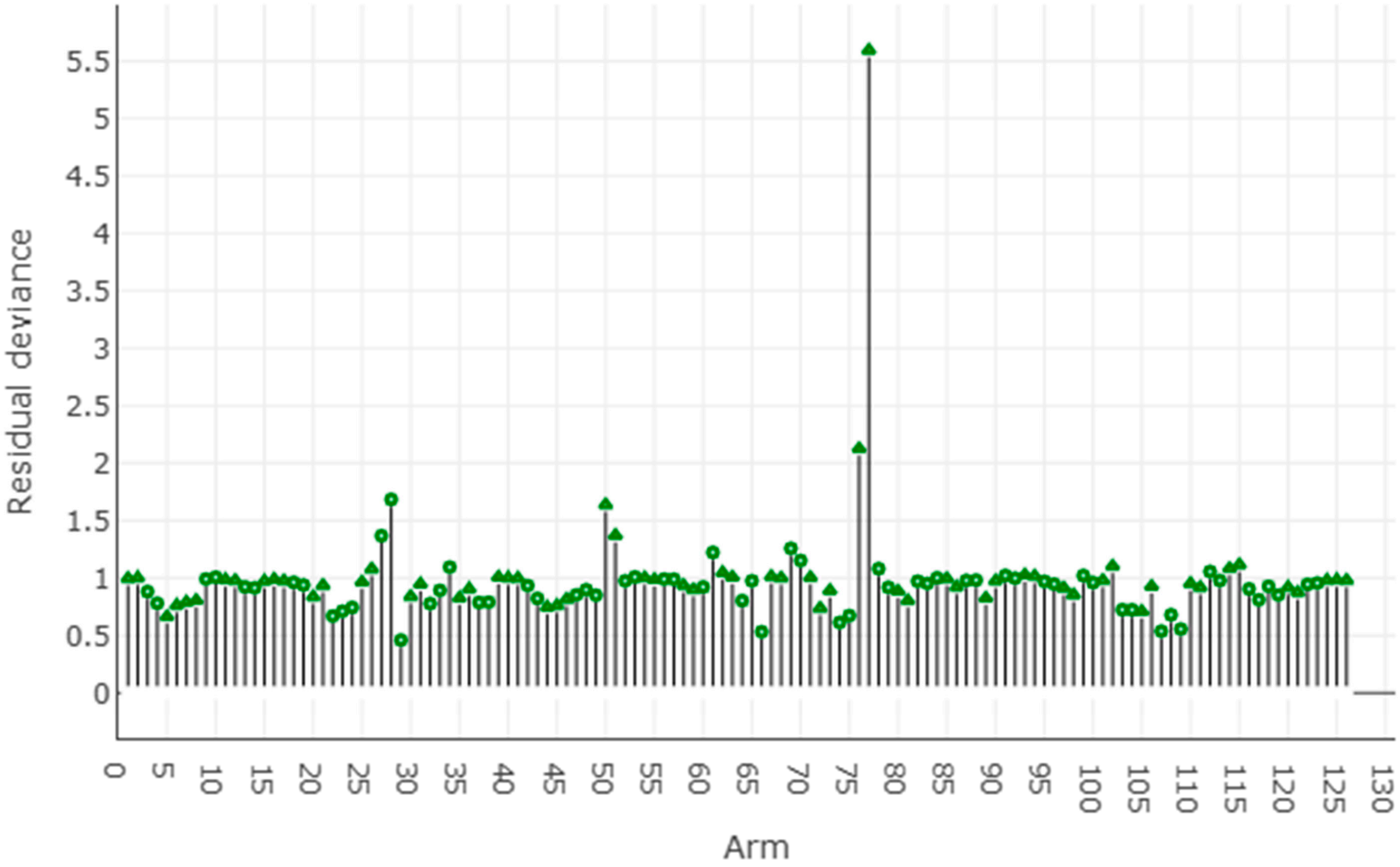

**Figure S6O Bayesian-based leverage plot of safety profile: drop-out rate**

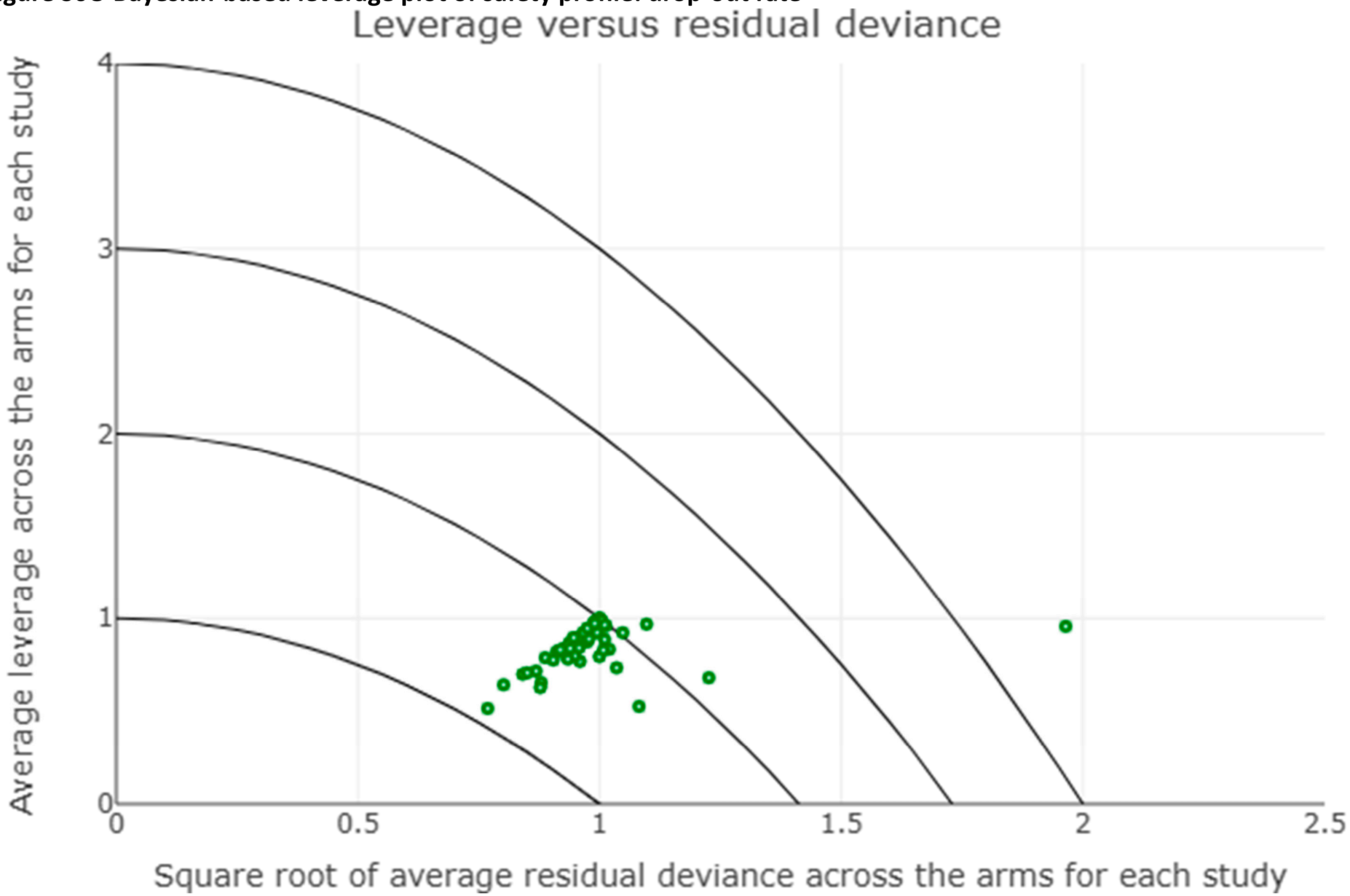

***Abbreviation for Figure S6A-O:***

*95%CIs: 95% confidence intervals; GLP-1 agonist: glucagon-like peptide-1 agonist; NMA: network meta-analysis; OR: odds ratio; RCT: randomized controlled trial; SGLT2 inhibitor: sodium–glucose cotransporter 2 inhibitor*

**Figure S7A overview of risk of bias**

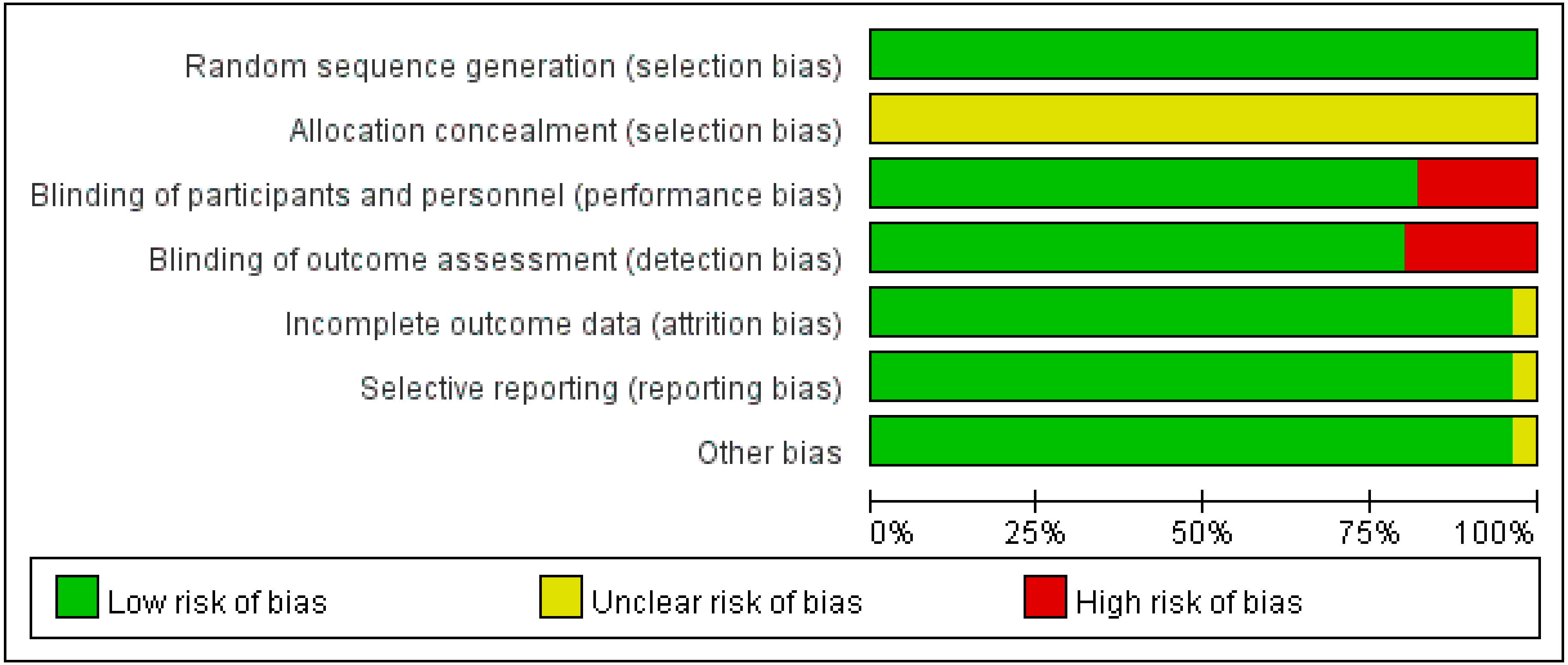

## Figure S7B detailed risk of bias in each study

| | Random sequence generation (selection bias) | Allocation concealment (selection bias) | Blinding of participants and personnel (performance bias) | Blinding of outcome assessment (detection bias) | Incomplete outcome data (attrition bias) | Selective reporting (reporting bias) | Other bias |
|---|---|---|---|---|---|---|---|
| Anker, S.D. (2021) (EMPEROR-Preserved) | + | ? | + | + | + | + | + |
| Aronne, L.J. (2024) (SURMOUNT-4) | + | ? | − | − | + | + | + |
| Bailey, C.J. (2010) (MB102-014) | + | ? | + | + | + | + | + |
| Bhatt, D.L. (2021) (SCORED) | + | ? | + | + | + | + | + |
| Buse, J.B. (2009) (LEAD-6) | + | ? | − | − | + | + | + |
| Cannon, C.P. (2020) (VERTIS CV) | + | ? | + | + | + | + | + |
| Cherney, D.Z.I.(2023) (SOTA-CKD3) | + | ? | + | + | + | + | + |
| Danne, T. (2018) (inTandem2) | + | ? | + | + | + | + | + |
| Davies, M. (2021) (STEP 2) | + | ? | + | + | + | + | + |
| Del Prato, S. (2021) (SURPASS-4) | + | ? | − | − | + | + | + |
| Ferrannini, E. (2013) monotherapy | + | ? | − | − | + | + | + |
| Frías, J.P. (2021) (SURPASS-2) | + | ? | − | − | + | + | + |
| Gallo, S. (2019) (VERTIS MET) | + | ? | + | + | + | + | + |
| Garvey, W.T. (2023) (SURMOUNT-2) | + | ? | + | + | + | + | + |
| Gerstein, H.C. (2019) (REWIND) | + | ? | + | + | + | + | + |
| Gerstein, H.C. (2021) (AMPLITUDE-O) | + | ? | + | + | + | + | + |
| Giorgino, F. (2015) (AWARD-2) | + | ? | − | − | + | + | + |
| Grunberger, G. (2018) (VERTIS RENAL) | + | ? | + | + | + | + | + |

| | | | | | | | |
|---|---|---|---|---|---|---|---|
| Hadjadj, S. (2016) | + | ? | + | + | + | + | + |
| Heerspink, H.J.L. (2020) (DAPA-CKD) | + | ? | + | + | + | + | + |
| Hernandez, A.F. (2018) (Harmony Outcomes) | + | ? | + | + | + | + | + |
| Herrington, W.G. (2023) (EMPA-KIDNEY) | + | ? | + | + | + | + | + |
| Holman, R.R. (2017) (EXSCEL) | + | ? | + | + | + | + | + |
| Home, P.D. (2017) (HARMONY 3-NCT00838903) | + | ? | + | + | + | + | + |
| Husain, M. (2019) (PIONEER 6) | + | ? | + | + | + | + | + |
| Jastreboff, A.M. (2022) (SURMOUNT-1) | + | ? | + | + | + | + | + |
| Kaku, K. (2018) (SUSTAIN) | + | ? | - | - | + | + | + |
| Lincoff, A.M. (2023) (SELECT) | + | ? | + | + | + | + | + |
| Lock, J.P. (2021) (BEST) (NCT02558296) | + | ? | + | + | ? | ? | ? |
| Marso, S.P. (2016) (LEADER) | + | ? | + | + | + | + | + |
| Marso, S.P. (2016) (SUSTAIN-6) | + | ? | + | + | + | + | + |
| McMurray, J.J.V. (2019) (DAPA-HF) | + | ? | + | + | + | + | + |
| Nauck, M. (2009) (LEAD-2) | + | ? | + | + | + | + | + |
| Nauck, M. (2016) (LIRA-LIXI) | + | ? | - | - | + | + | + |
| Nauck, M.A. (2016) (HARMONY 2) | + | ? | + | + | + | + | + |
| Neal, B. (2017) (CANVAS) | + | ? | + | + | + | + | + |
| Neal, B. (2017) (CANVAS-R) | + | ? | + | + | + | + | + |
| Packer, M. (2020) (EMPEROR-Reduced) | + | ? | + | + | + | + | + |
| Perkovic, V. (2019) (CREDENCE) | + | ? | + | - | + | + | + |
| Pfeffer, M.A. (2015) (ELIXA) | + | ? | + | + | + | + | + |
| Pi-Sunyer, X. (2015) (SCALE) (before 56 weeks) | + | ? | + | + | + | + | + |
| Ridderstrale, M. (2014) (EMPA-REG H2H-SU) | + | ? | + | + | + | + | + |
| Rosenstock, J. (2014) (HARMONY 6) | + | ? | - | - | + | + | + |
| Rosenstock, J. (2019) (PIONEER 3) | + | ? | + | + | + | + | + |
| Rubino, D. (2021) (STEP 4) | + | ? | + | + | + | + | + |
| Solomon, S.D. (2022) (DELIVER) | + | ? | + | + | + | + | + |
| Tuttle, K.R. (2022) (NCT00749190) | + | ? | + | + | + | + | + |
| Umpierrez, G. (2014) (AWARD-3) | + | ? | + | + | + | + | + |
| Wada, T. (2022) (TA-7284-14) | + | ? | + | + | + | + | + |
| Wason, S. (2021) (SOTA-INS) (NCT03285594) | + | ? | + | + | ? | ? | ? |
| Weissman, P.N. (2014) (HARMONY 4) | + | ? | - | - | + | + | + |
| Wilding, J.P. (2012) | + | ? | + | + | + | + | + |
| Wilding, J.P.H. (2021) (STEP 1) | + | ? | + | + | + | + | + |
| Wiviott, S.D. (2019) (DECLARE-TIMI 58) | + | ? | + | + | + | + | + |
| Zinman, B. (2015) (EMPA-REG OUTCOME) | + | ? | + | + | + | + | + |

**Table S1:** PRISMA 2020 checklist of the current network meta-analysis

| Section and Topic | Item # | Checklist item | Page where item is reported |
|---|---|---|---|
| **TITLE** | | | |
| Title | 1 | Identify the report as a systematic review. | 1 |
| **ABSTRACT** | | | |
| Abstract | 2 | See the PRISMA 2020 for Abstracts checklist. | 7-8 |
| **INTRODUCTION** | | | |
| Rationale | 3 | Describe the rationale for the review in the context of existing knowledge. | 9-10 |
| Objectives | 4 | Provide an explicit statement of the objective(s) or question(s) the review addresses. | 9-10 |
| **METHODS** | | | |
| Eligibility criteria | 5 | Specify the inclusion and exclusion criteria for the review and how studies were grouped for the syntheses. | 11-12 |
| Information sources | 6 | Specify all databases, registers, websites, organisations, reference lists and other sources searched or consulted to identify studies. Specify the date when each source was last searched or consulted. | 11-12 |
| Search strategy | 7 | Present the full search strategies for all databases, registers and websites, including any filters and limits used. | 11-12 |
| Selection process | 8 | Specify the methods used to decide whether a study met the inclusion criteria of the review, including how many reviewers screened each record and each report retrieved, whether they worked independently, and if applicable, details of automation tools used in the process. | 11-12 |
| Data collection process | 9 | Specify the methods used to collect data from reports, including how many reviewers collected data from each report, whether they worked independently, any processes for obtaining or confirming data from study investigators, and if applicable, details of automation tools used in the process. | 11-12 |
| Data items | 10a | List and define all outcomes for which data were sought. Specify whether all results that were compatible with each outcome domain in each study were sought (e.g. for all measures, time points, analyses), and if not, the methods used to decide which results to collect. | 12-13 |
| | 10b | List and define all other variables for which data were sought (e.g. participant and intervention characteristics, funding sources). Describe any assumptions made about any missing or unclear information. | 12-13 |
| Study risk of bias assessment | 11 | Specify the methods used to assess risk of bias in the included studies, including details of the tool(s) used, how many reviewers assessed each study and whether they worked independently, and if applicable, details of automation tools used in the process. | 12-13 |
| Effect measures | 12 | Specify for each outcome the effect measure(s) (e.g. risk ratio, mean difference) used in the synthesis or presentation of results. | 12-13 |
| Synthesis methods | 13a | Describe the processes used to decide which studies were eligible for each synthesis (e.g. tabulating the study intervention characteristics and comparing against the planned groups for each synthesis (item #5)). | 12-13 |
| | 13b | Describe any methods required to prepare the data for presentation or synthesis, such as handling of missing summary statistics, or data conversions. | 13-15 |
| | 13c | Describe any methods used to tabulate or visually display results of individual studies and syntheses. | 13-15 |
| | 13d | Describe any methods used to synthesize results and provide a rationale for the choice(s). If meta-analysis was performed, describe the model(s), method(s) to identify the presence and extent of statistical heterogeneity, and software package(s) used. | 13-15 |
| | 13e | Describe any methods used to explore possible causes of heterogeneity among study results (e.g. subgroup analysis, meta-regression). | 13-15 |
| | 13f | Describe any sensitivity analyses conducted to assess robustness of the synthesized results. | 13-15 |

| Section and Topic | Item # | Checklist item | Page where item is reported |
|---|---|---|---|
| Reporting bias assessment | 14 | Describe any methods used to assess risk of bias due to missing results in a synthesis (arising from reporting biases). | 13-15 |
| Certainty assessment | 15 | Describe any methods used to assess certainty (or confidence) in the body of evidence for an outcome. | 13-15 |
| **RESULTS** | | | |
| Study selection | 16a | Describe the results of the search and selection process, from the number of records identified in the search to the number of studies included in the review, ideally using a flow diagram. | 16-17, Fig 1, eTab 2 |
| | 16b | Cite studies that might appear to meet the inclusion criteria, but which were excluded, and explain why they were excluded. | 16-17, eTab 3 |
| Study characteristics | 17 | Cite each included study and present its characteristics. | 16-17, eTab 4 |
| Risk of bias in studies | 18 | Present assessments of risk of bias for each included study. | 16-17, eFig 7 |
| Results of individual studies | 19 | For all outcomes, present, for each study: (a) summary statistics for each group (where appropriate) and (b) an effect estimate and its precision (e.g. confidence/credible interval), ideally using structured tables or plots. | 16-17, eFig 3 |
| Results of syntheses | 20a | For each synthesis, briefly summarise the characteristics and risk of bias among contributing studies. | 17-18, Fig 2 |
| | 20b | Present results of all statistical syntheses conducted. If meta-analysis was done, present for each the summary estimate and its precision (e.g. confidence/credible interval) and measures of statistical heterogeneity. If comparing groups, describe the direction of the effect. | 17-18, Fig 3 |
| | 20c | Present results of all investigations of possible causes of heterogeneity among study results. | 17-18, eTab 7 |
| | 20d | Present results of all sensitivity analyses conducted to assess the robustness of the synthesized results. | 17-18 |
| Reporting biases | 21 | Present assessments of risk of bias due to missing results (arising from reporting biases) for each synthesis assessed. | 17-18, eFig 7 |
| Certainty of evidence | 22 | Present assessments of certainty (or confidence) in the body of evidence for each outcome assessed. | 17-18 |
| **DISCUSSION** | | | |
| Discussion | 23a | Provide a general interpretation of the results in the context of other evidence. | 19-21 |
| | 23b | Discuss any limitations of the evidence included in the review. | 21-22 |
| | 23c | Discuss any limitations of the review processes used. | 21-22 |
| | 23d | Discuss implications of the results for practice, policy, and future research. | 23 |
| **OTHER INFORMATION** | | | |
| Registration and protocol | 24a | Provide registration information for the review, including register name and registration number, or state that the review was not registered. | 8 |
| | 24b | Indicate where the review protocol can be accessed, or state that a protocol was not prepared. | 8 |
| | 24c | Describe and explain any amendments to information provided at registration or in the protocol. | 8 |
| Support | 25 | Describe sources of financial or non-financial support for the review, and the role of the funders or sponsors in the review. | 24 |
| Competing interests | 26 | Declare any competing interests of review authors. | 24 |
| Availability of data, | 27 | Report which of the following are publicly available and where they can be found: template data collection forms; data extracted from included | 24 |

| Section and Topic | Item # | Checklist item | Page where item is reported |
|---|---|---|---|
| code and other materials | | studies; data used for all analyses; analytic code; any other materials used in the review. | |

The current checklist followed the latest PRISMA 2020 guideline [1].

**Table S2: Keyword used in each database and search results**

| Database | Keyword | Filter | Date | Result |
|---|---|---|---|---|
| PubMed | (leukemia OR leukaemia OR lymphoma OR myeloma) AND (glucagon-like peptide-1 receptor agonist OR Sodium Glucose Cotransporter 2 Inhibitor OR lixisenatide OR orforglipron OR exenatide OR semaglutide OR liraglutide OR albiglutide OR dulaglutide OR tirzepatide OR bexagliflozin OR canagliflozin OR dapagliflozin OR empagliflozin OR ertugliflozin OR ipragliflozin OR luseogliflozin OR remogliflozin OR sergliflozin OR sotagliflozin OR tofogliflozin OR henagliflozin OR janagliflozin OR mizagliflozin OR velagliflozin OR enavogliflozin OR licogliflozin OR rongliflozin) AND (random OR randomized OR randomised) | N/A | 2024/12/04 | 7 |
| ClinicalKey | (leukemia OR leukaemia OR lymphoma OR myeloma) AND (glucagon-like peptide-1 receptor agonist OR Sodium Glucose Cotransporter 2 Inhibitor OR lixisenatide OR orforglipron OR exenatide OR semaglutide OR liraglutide OR albiglutide OR dulaglutide OR tirzepatide OR bexagliflozin OR canagliflozin OR dapagliflozin OR empagliflozin OR ertugliflozin OR ipragliflozin OR luseogliflozin OR remogliflozin OR sergliflozin OR sotagliflozin OR tofogliflozin OR henagliflozin OR janagliflozin OR mizagliflozin OR velagliflozin OR enavogliflozin OR licogliflozin OR rongliflozin) AND (random OR randomized OR randomised) | N/A | 2024/12/04 | 822 |
| Cochrane CENTRAL | (leukemia OR leukaemia OR lymphoma OR myeloma) AND (glucagon-like peptide-1 receptor agonist OR Sodium Glucose Cotransporter 2 Inhibitor OR lixisenatide OR | N/A | 2024/12/04 | 6 |

| | | | | |
|---|---|---|---|---|
| | orforglipron OR exenatide OR semaglutide OR liraglutide OR albiglutide OR dulaglutide OR tirzepatide OR bexagliflozin OR canagliflozin OR dapagliflozin OR empagliflozin OR ertugliflozin OR ipragliflozin OR luseogliflozin OR remogliflozin OR sergliflozin OR sotagliflozin OR tofogliflozin OR henagliflozin OR janagliflozin OR mizagliflozin OR velagliflozin OR enavogliflozin OR licogliflozin OR rongliflozin) AND (random OR randomized OR randomised) | | | |
| Embase | (leukemia OR leukaemia OR lymphoma OR myeloma) AND (glucagon-like peptide-1 receptor agonist OR Sodium Glucose Cotransporter 2 Inhibitor) AND (random OR randomized OR randomised) | N/A | 2024/12/04 | 61 |
| ProQuest | (leukemia OR leukaemia OR lymphoma OR myeloma) AND (glucagon-like peptide-1 receptor agonist OR Sodium Glucose Cotransporter 2 Inhibitor) AND (random OR randomized OR randomised) | N/A | 2024/12/04 | 2028 |
| ScienceDirect | (leukemia OR leukaemia OR lymphoma OR myeloma) AND (glucagon-like peptide-1 receptor agonist OR Sodium Glucose Cotransporter 2 Inhibitor) AND (random OR randomized OR randomised) | N/A | 2024/12/04 | 1927 |
| Web of Science | (leukemia OR leukaemia OR lymphoma OR myeloma) AND (glucagon-like peptide-1 receptor agonist OR Sodium Glucose Cotransporter 2 Inhibitor) AND (random OR randomized OR randomised) | N/A | 2024/12/04 | 3 |
| ClinicalTrials.gov | (leukemia OR leukaemia OR lymphoma OR myeloma) AND (glucagon-like peptide-1 receptor agonist OR Sodium Glucose Cotransporter 2 Inhibitor OR lixisenatide OR orforglipron OR exenatide OR semaglutide OR liraglutide OR albiglutide OR dulaglutide OR tirzepatide OR bexagliflozin OR canagliflozin OR dapagliflozin OR empagliflozin OR | N/A | 2024/12/04 | 1 |

| | |
|---|---|
| | ertugliflozin OR ipragliflozin OR luseogliflozin OR remogliflozin OR sergliflozin OR sotagliflozin OR tofogliflozin OR henagliflozin OR janagliflozin OR mizagliflozin OR velagliflozin OR enavogliflozin OR licogliflozin OR rongliflozin) |

Abbreviation: N/A: not applied

**Table S3: Excluded studies and reason**

| Reason | Numbers | References |
|---|---|---|
| Animal study | 1 | [2] |
| Duplicate sample source with another included trial | 1 | [3] |
| Meta-analysis | 1 | [4] |
| Not randomized controlled trial | 2 | [5,6] |
| Not report targeted outcome | 84 | [7-90] |
| Study protocol but not result of a study | 1 | [91] |

## Table S4: Characteristics of the included studies

| Study name | Baseline illness | Comparison | Subjects | Mean age (year) | Female (%) | Treatment duration | Category | ClinicalTrials.gov | Country |
|---|---|---|---|---|---|---|---|---|---|
| Aronne, L.J. (2024) (SURMOUNT-4)[92] | patients with obesity | Tirzepatide 10-15mg<br>Placebo | 335<br>335 | 49.0±13.0<br>48.0±12.0 | 70.4<br>70.7 | 36 weeks | GLP-1 agonist | NCT04660643 | Multiple countries |
| Cherney, D.Z.I.(2023) (SOTA-CKD3)[93] | patients with type 2 diabetes mellitus | Sotagliflozin<br>Placebo | 527<br>260 | 69.5±7.9<br>69.3±8.1 | 44.0<br>42.7 | 26 weeks | SGLT2 inhibitor | NCT03242252 | Multiple countries |
| Garvey, W.T. (2023) (SURMOUNT-2)[94] | patients with obesity | Tirzepatide 10mg<br>Tirzepatide 15mg<br>Placebo | 312<br>311<br>315 | 54.3±10.7<br>53.6±10.6<br>54.7±10.5 | 50.6<br>51.1<br>50.5 | 72 weeks | GLP-1 agonist | NCT04657003 | Multiple countries |
| Herrington, W.G. (2023) (EMPA-KIDNEY)[95] | patients with renal failure | Empagliflozin 10mg<br>Placebo | 3304<br>3305 | 63.9±13.9<br>63.8±13.9 | 33.2<br>33.1 | 104 weeks | SGLT2 inhibitor | NCT03594110 | Multiple countries |
| Lincoff, A.M. (2023) (SELECT)[96] | patients with obesity | Inject Semaglutide 2.4mg<br>Placebo | 8803<br>8801 | 61.6±8.9<br>61.6±8.8 | 27.8<br>27.5 | 104 weeks | GLP-1 agonist | NCT03574597 | Multiple countries |
| Jastreboff, A.M. (2022) (SURMOUNT-1)[97] | patients with obesity | Tirzepatide 5mg<br>Tirzepatide 10mg<br>Tirzepatide 15mg<br>Placebo | 630<br>636<br>630<br>643 | 45.6±12.7<br>44.7±12.4<br>44.9±12.3<br>44.4±12.5 | 67.6<br>67.1<br>67.5<br>67.8 | 72 weeks | GLP-1 agonist | NCT04184622 | Multiple countries |
| Solomon, S.D. (2022) (DELIVER)[98] | patients with stabilized heart failure | Dapagliflozin 10mg<br>Placebo | 3131<br>3132 | 71.8±9.6<br>71.5±9.5 | 43.6<br>44.2 | 120 weeks | SGLT2 inhibitor | NCT03619213 | Multiple countries |
| Tuttle, K.R. (2022) (NCT00749190)[73] | patients with type 2 diabetes mellitus | Empagliflozin 1-10mg<br>Empagliflozin 25-50mg<br>Placebo | 213<br>140<br>71 | 58.7±8.5<br>57.3±8.9<br>59.7±8.5 | 51.7<br>45.7<br>53.5 | 12 weeks | SGLT2 inhibitor | NCT00749190 | Multiple countries |
| Wada, T. (2022) (TA-7284-14)[99] | patients with type 2 diabetes mellitus and chronic kidney disease | Canagliflozin 100 mg<br>Placebo | 154<br>154 | 62.5±10.5<br>62.4±11.1 | 25.3<br>16.2 | 104 weeks | SGLT2 inhibitor | NCT03436693 | Japan |
| Anker, S.D. (2021) (EMPEROR-Preserved)[100] | patients with heart failure with preserved ejection fraction | Empagliflozin 10mg<br>Placebo | 2997<br>2991 | 71.8±9.3<br>71.9±9.6 | 44.6<br>44.7 | 156 weeks | SGLT2 inhibitor | NCT03057951 | Multiple countries |

| | | | | | | | | | |
|---|---|---|---|---|---|---|---|---|---|
| Bhatt, D.L. (2021) (SCORED)[101] | patients with type 2 diabetes mellitus and chronic kidney disease | Sotagliflozin 200-400mg<br>Placebo | 5292<br>5292 | 68.4±8.4<br>68.2±8.4 | 44.3<br>45.5 | 116 weeks | SGLT2 inhibitor | NCT03315143 | Multiple countries |
| Davies, M. (2021) (STEP 2)[102] | patients with type 2 diabetes mellitus and obesity | Inject semaglutide 1.0 mg<br>Inject Semaglutide 2.4 mg<br>Placebo | 403<br>404<br>403 | 56.0±10.0<br>55.0±11.0<br>55.0±11.0 | 50.4<br>55.2<br>47.1 | 68 weeks | GLP-1 agonist | NCT03552757 | Multiple countries |
| Del Prato, S. (2021) (SURPASS-4)[103] | patients with type 2 diabetes mellitus | Tirzepatide 5mg<br>Tirzepatide 10mg<br>Tirzepatide 15mg<br>Controls with insulin glargine | 329<br>328<br>338<br>1000 | 62.9±8.6<br>63.7±8.7<br>63.7±8.6<br>63.8±8.5 | 39.8<br>36.3<br>39.9<br>36.4 | 108 weeks | GLP-1 agonist | NCT03730662 | Multiple countries |
| Frías, J.P. (2021) (SURPASS-2)[104] | patients with type 2 diabetes mellitus | Tirzepatide 5mg<br>Tirzepatide 10mg<br>Tirzepatide 15mg<br>Inject semaglutide 1.0 mg | 470<br>469<br>470<br>469 | 56.3±10.0<br>57.2±10.5<br>55.9±10.4<br>56.9±10.8 | 56.4<br>49.3<br>54.5<br>52.0 | 40 weeks | GLP-1 agonist | NCT03987919 | Multiple countries |
| Gerstein, H.C. (2021) (AMPLITUDE-O)[105] | patients with type 2 diabetes mellitus | Efpeglenatide 4 mg<br>Efpeglenatide 6 mg<br>Placebo | 1359<br>1358<br>1359 | 64.6±8.2<br>64.7±8.2<br>64.4±8.3 | 32.5<br>35.6<br>30.8 | 104 weeks | GLP-1 agonist | NCT03496298 | Multiple countries |
| Lock, J.P. (2021) (BEST) (NCT02558296)[106] | patients with type 2 diabetes mellitus | Bexagliflozin 20mg<br>Placebo | 1132<br>567 | 64.4±7.9<br>64.6±8.0 | 30.1<br>31.2 | 52 weeks | SGLT2 inhibitor | NCT02558296 | Multiple countries |
| Rubino, D. (2021) (STEP 4)[107] | patients with overweight or obesity | Inject semaglutide 2.4 mg<br>Placebo | 535<br>268 | 47.0±12.0<br>46.0±12.0 | 80.2<br>76.5 | 68 weeks | GLP-1 agonist | NCT03548987 | Multiple countries |
| Wason, S. (2021) (SOTA-INS) (NCT03285594)[108] | patients with type 2 diabetes mellitus | Sotagliflozin 200-400mg<br>Placebo | 427<br>144 | 62.5±9.5<br>62.2±8.9 | 46.2<br>40.3 | 52 weeks | SGLT2 inhibitor | NCT03285594 | Multiple countries |
| Wilding, J.P.H. (2021) (STEP 1)[109] | patients with obesity | Inject semaglutide 2.4 mg<br>Placebo | 1306<br>655 | 46.0±13.0<br>47.0±12.0 | 73.1<br>76.0 | 68 weeks | GLP-1 agonist | NCT03548935 | Multiple countries |

| | | | | | | | | | |
|---|---|---|---|---|---|---|---|---|---|
| Cannon, C.P. (2020) (VERTIS CV)[110] | patients with type 2 diabetes mellitus | Ertugliflozin 5 mg<br>Ertugliflozin 15 mg<br>Placebo | 2752<br>2747<br>2747 | 64.3±8.2<br>64.4±8.0<br>64.4±8.0 | 29.1<br>30.3<br>30.7 | 182 weeks | SGLT2 inhibitor | NCT01986881 | Multiple countries |
| Heerspink, H.J.L. (2020) (DAPA-CKD)[111] | patients with renal failure | Dapagliflozin 10mg<br>Placebo | 2152<br>2152 | 61.8±12.1<br>61.9±12.1 | 32.9<br>33.3 | 125 weeks | SGLT2 inhibitor | NCT03036150 | Multiple countries |
| Packer, M. (2020) (EMPEROR-Reduced)[112] | patients with chronic heart failure | Empagliflozin 10mg<br>Placebo | 1863<br>1867 | 67.2±10.8<br>66.5±11.2 | 23.5<br>24.4 | 64 weeks | SGLT2 inhibitor | NCT03057977 | Multiple countries |
| Gallo, S. (2019) (VERTIS MET)[113] | patients with type 2 diabetes mellitus | Ertugliflozin 5 mg<br>Ertugliflozin 15 mg<br>Placebo with glimepiride | 207<br>205<br>209 | 56.6±8.2<br>56.9±9.4<br>56.5±8.7 | 53.1<br>54.6<br>53.1 | 104 weeks | SGLT2 inhibitor | NCT02033889 | Multiple countries |
| Gerstein, H.C. (2019) (REWIND)[114] | patients with type 2 diabetes mellitus | Dulaglutide 1.5 mg<br>Placebo | 4949<br>4952 | 66.2±6.5<br>66.2±6.5 | 46.6<br>46.1 | 281 weeks | GLP-1 agonist | NCT01394952 | Multiple countries |
| Husain, M. (2019) (PIONEER 6)[115] | patients with cardiovascular disease or chronic kidney disease | Semaglutide 14mg<br>Placebo | 1591<br>1592 | 66.0±7.0<br>66.0±7.0 | 31.9<br>31.4 | 64 weeks | GLP-1 agonist | NCT02692716 | Multiple countries |
| McMurray, J.J.V. (2019) (DAPA-HF)[116] | patients with stabilized heart failure | Dapagliflozin 10mg<br>Placebo | 2373<br>2371 | 66.2±11.0<br>66.5±10.8 | 23.8<br>23.0 | 73 weeks | SGLT2 inhibitor | NCT03036124 | Multiple countries |
| Perkovic, V. (2019) (CREDENCE)[117] | patients with type 2 diabetes mellitus | Canagliflozin 100 mg<br>Placebo | 2202<br>2199 | 62.9±9.2<br>63.2±9.2 | 34.6<br>33.3 | 130 weeks | SGLT2 inhibitor | NCT02065791 | Multiple countries |
| Rosenstock, J. (2019) (PIONEER 3)[118] | patients with type 2 diabetes mellitus | Semaglutide 3-14 mg<br>Control with sitagliptin | 1396<br>466 | 57.7±10.0<br>58.0±10.0 | 46.6<br>49.0 | 78 weeks | GLP-1 agonist | NCT02607865 | Multiple countries |
| Wiviott, S.D. (2019) (DECLARE-TIMI 58)[119] | patients with atherosclerotic vascular disease | Dapagliflozin 10mg<br>Placebo | 8582<br>8578 | 63.9±6.8<br>64.0±6.8 | 36.9<br>37.9 | 206 weeks | SGLT2 inhibitor | NCT01730534 | Multiple countries |
| Danne, T. (2018) (inTandem2)[120] | patients with type 1 diabetes mellitus | Sotagliflozin 200-400mg<br>Placebo | 524<br>258 | 42.0±13.4<br>39.7±13.4 | 48.1<br>48.1 | 52 weeks | SGLT2 inhibitor | NCT02421510 | Multiple countries |
| Grunberger, G. (2018) (VERTIS RENAL)[121] | patients with type 2 diabetes mellitus with chronic kidney disease | Ertugliflozin 5 mg<br>Ertugliflozin 15 mg<br>Placebo | 158<br>155<br>154 | 66.7±8.3<br>67.5±8.5<br>67.5±8.9 | 46.8<br>51.6<br>53.2 | 54 weeks | SGLT2 inhibitor | NCT01986855 | Multiple countries |
| Hernandez, A.F. (2018) (Harmony Outcomes)[122] | patients with type 2 diabetes mellitus | Albiglutide 30-50 mg<br>Placebo | 4731<br>4732 | 64.1±8.7<br>64.2±8.7 | 30.2<br>31.0 | 86 weeks | GLP-1 agonist | NCT02465515 | Multiple countries |

| | | | | | | | | | |
|---|---|---|---|---|---|---|---|---|---|
| Kaku, K. (2018) (SUSTAIN)[123] | patients with type 2 diabetes mellitus | Inject semaglutide 0.5 mg<br>Inject semaglutide 1.0 mg<br>Control with standard care | 239<br>241<br>121 | 58.0±10.6<br>58.7±10.2<br>59.2±10.1 | 30.5<br>27.8<br>25.8 | 61 weeks | GLP-1 agonist | NCT02207374 | Multiple countries |
| Holman, R.R. (2017) (EXSCEL)[124] | patients with type 2 diabetes mellitus | Exenatide 2mg<br>Placebo | 7356<br>7396 | 61.8±9.4<br>61.9±9.4 | 38.0<br>38.0 | 166 weeks | GLP-1 agonist | NCT01144338 | Multiple countries |
| Home, P.D. (2017) (HARMONY 3-NCT00838903)[27] | patients with type 2 diabetes mellitus | Albiglutide 30 mg<br>Placebo | 302<br>101 | 54.3±10.1<br>56.1±10.0 | 55.3<br>50.5 | 104 weeks | GLP-1 agonist | NCT00838903 | Multiple countries |
| Neal, B. (2017) (CANVAS)[125] | patients with type 2 diabetes mellitus | Canagliflozin 100 mg<br>Canagliflozin 300 mg<br>Placebo | 1445<br>1443<br>1442 | 62.2±8.0<br>62.8±8.1<br>62.3±7.9 | 33.5<br>34.6<br>33.7 | 126 weeks | SGLT2 inhibitor | NCT01032629 | Multiple countries |
| Neal, B. (2017) (CANVAS-R)[125] | patients with type 2 diabetes mellitus | Canagliflozin 300 mg<br>Placebo | 2907<br>2905 | 63.9±8.4<br>64.0±8.3 | 36.2<br>38.2 | 126 weeks | SGLT2 inhibitor | NCT01989754 | Multiple countries |
| Hadjadj, S. (2016)[126] | patients with type 2 diabetes mellitus | Empagliflozin 10mg<br>Empagliflozin 25mg<br>Placebo | 497<br>551<br>332 | 52.5±11.2<br>52.4±10.7<br>52.5±10.9 | 41.1<br>42.5<br>46.4 | 24 weeks | SGLT2 inhibitor | NCT01719003 | Multiple countries |
| Marso, S.P. (2016) (LEADER)[127] | patients with type 2 diabetes mellitus | Liraglutide 1.8mg<br>Placebo | 4668<br>4672 | 64.2±7.2<br>64.4±7.2 | 35.5<br>36.0 | 198 weeks | GLP-1 agonist | NCT01179048 | Multiple countries |
| Marso, S.P. (2016) (SUSTAIN-6)[128] | patients with type 2 diabetes mellitus | Inject semaglutide 0.5 mg<br>Inject semaglutide 1.0 mg<br>Placebo | 826<br>822<br>1649 | NA | 40.1<br>37.0<br>40.0 | 109 weeks | GLP-1 agonist | NCT01720446 | Multiple countries |
| Nauck, M. (2016) (LIRA-LIXI)[129] | patients with type 2 diabetes mellitus | Liraglutide 1.8mg<br>Lixisenatide 20ug | 202<br>202 | 56.3±10.6<br>56.1±10.0 | 35.0<br>45.0 | 26 weeks | GLP-1 agonist | NCT01973231 | Multiple countries |
| Nauck, M.A. (2016) (HARMONY 2)[130] | patients with type 2 diabetes mellitus | Albiglutide 30-50 mg<br>Placebo | 200<br>101 | 52.8±11.4<br>53.1±11.7 | 46.0<br>42.6 | 52 weeks | GLP-1 agonist | NCT00849017 | Multiple countries |
| Giorgino, F. (2015) (AWARD-2)[131] | patients with type 2 diabetes mellitus | Dulaglutide 0.75-1.5 mg<br>Control with insulin glargine | 545<br>262 | 56.5±9.5<br>57.0±9.0 | 48.6<br>48.9 | 78 weeks | GLP-1 agonist | NCT01075282 | Multiple countries |

| | | | | | | | | | |
|---|---|---|---|---|---|---|---|---|---|
| Pfeffer, M.A. (2015) (ELIXA)[132] | patients with type 2 diabetes mellitus and recent acute coronary syndrome | Lixisenatide 20ug<br>Placebo | 3034<br>3034 | 59.9±9.7<br>60.6±9.6 | 30.4<br>30.9 | 100 weeks | GLP-1 agonist | NCT01147250 | Multiple countries |
| Pi-Sunyer, X. (2015) (SCALE) (before 56 weeks)[133] | patients with obesity | Liraglutide 3.0mg<br>Placebo | 2487<br>1244 | 45.2±12.1<br>45.0±12.0 | 78.7<br>78.1 | 56 weeks | GLP-1 agonist | NCT01272219 | Multiple countries |
| Zinman, B. (2015) (EMPA-REG OUTCOME)[134] | patients with type 2 diabetes mellitus | Empagliflozin 10mg<br>Empagliflozin 25mg<br>Placebo | 2345<br>2342<br>2333 | 63.0±8.6<br>63.2±8.6<br>63.2±8.8 | 29.5<br>28.1<br>28.0 | 135 weeks | SGLT2 inhibitor | NCT01131676 | Multiple countries |
| Ridderstrale, M. (2014) (EMPA-REG H2H-SU)[135] | patients with type 2 diabetes mellitus and moderate-to-severe chronic kidney disease | Empagliflozin 25mg<br>Control with glimepiride | 765<br>780 | 56.2±10.3<br>55.7±10.4 | 43.5<br>46.0 | 104 weeks | SGLT2 inhibitor | NCT01167881 | Multiple countries |
| Rosenstock, J. (2014) (HARMONY 6)[136] | patients with type 2 diabetes mellitus | Albiglutide 30-50 mg<br>Control with Insulin glargine and lispro | 285<br>281 | 54.8±9.1<br>56.3±8.9 | 53.7<br>51.6 | 52 weeks | GLP-1 agonist | NCT00976391 | Multiple countries |
| Umpierrez, G. (2014) (AWARD-3)[137] | patients with type 2 diabetes mellitus | Dulaglutide 0.75-1.5 mg<br>Placebo plus metformin | 539<br>268 | 56.0±10.5<br>55.0±10.0 | 56.9<br>54.9 | 52 weeks | GLP-1 agonist | NCT01126580 | Multiple countries |
| Weissman, P.N. (2014) (HARMONY 4)[138] | patients with type 2 diabetes mellitus | Albiglutide 30 mg<br>Control with insulin glargine | 504<br>241 | 55.8±9.3<br>54.7±9.8 | 43.3<br>45.2 | 52 weeks | GLP-1 agonist | NCT00838916 | Multiple countries |
| Ferrannini, E. (2013) monotherapy[139] | patients with type 2 diabetes mellitus | Empagliflozin 10mg<br>Empagliflozin 25mg<br>Placebo | 106<br>109<br>56 | 59.0<br>59.0<br>58.0 | 53.8<br>47.7<br>50.0 | 78 weeks | SGLT2 inhibitor | NCT00881530 | Multiple countries |
| Wilding, J.P. (2012)[140] | patients with type 2 diabetes mellitus and moderate-to-severe chronic kidney disease | Dapagliflozin 2.5 mg with insulin<br>Dapagliflozin 5 mg with insulin<br>Dapagliflozin 10 mg with insulin<br>Placebo with insulin | 202<br>211<br>194<br>193 | 59.8±7.6<br>59.3±7.9<br>59.3±8.8<br>58.8±8.6 | 50.5<br>52.6<br>55.2<br>50.8 | 104 weeks | SGLT2 inhibitor | NCT00673231 | Multiple countries |
| Bailey, C.J. (2010) (MB102-014)[141] | patients with type 2 diabetes mellitus | Dapagliflozin 2.5 mg<br>Dapagliflozin 5 mg | 137<br>137 | 55.0±9.3<br>54.3±9.4 | 48.9<br>49.6 | 24 weeks | SGLT2 inhibitor | NCT00528879 | Multiple countries |

| | | | | | | | | | |
|---|---|---|---|---|---|---|---|---|---|
| | | Dapagliflozin 10mg<br>Placebo | 135<br>137 | 52.7±9.9<br>53.7±10.3 | 43.0<br>44.5 | | | | |
| Buse, J.B. (2009) (LEAD-6)[142] | patients with type 2 diabetes mellitus and moderate-to-severe chronic kidney disease | Liraglutide<br>Exenatide | 233<br>231 | 56.3±9.8<br>57.1±10.8 | 51.1<br>45.0 | 26 weeks | GLP-1 agonist | NCT00518882 | Multiple countries |
| Nauck, M. (2009) (LEAD-2)[143] | patients with type 2 diabetes mellitus | Liraglutide<br>Placebo | 724<br>121 | 56.7±9.7<br>56.0±9.0 | 41.7<br>40.0 | 26 weeks | GLP-1 agonist | NCT00318461 | Multiple countries |

*Abbreviations: GLP-1 agonist: glucagon-like peptide-1 agonist; NA: not available; SGLT2 inhibitor: sodium–glucose cotransporter 2 inhibitor*

## Table S5A: League table of the primary outcome: subgroup of lymphoma

| | | | | | | | | | | | | | | | | | | | | | |
|---|---|---|---|---|---|---|---|---|---|---|---|---|---|---|---|---|---|---|---|---|---|
| Tirzepatide | . | 0.11 [0.00; 2.72] | . | . | . | . | . | . | 0.27 [0.04; 1.69] | . | . | . | . | . | . | . | . | . | . | . | . |
| 0.45 [0.04; 4.56] | Lixisenatide | . | . | . | . | . | . | . | 0.40 [0.08; 2.06] | . | . | . | . | . | . | . | . | . | . | . | . |
| 0.36 [0.05; 2.51] | 0.81 [0.09; 7.25] | Inject_ semaglutide_ medium_dosage | . | . | 0.62 [0.08; 5.08] | . | . | . | 0.35 [0.06; 2.15] | . | . | . | . | 0.33 [0.01; 8.21] | . | . | . | . | . | . | . |
| 0.40 [0.03; 6.06] | 0.88 [0.06; 13.53] | 1.09 [0.08; 15.11] | Empagliflozin_ high_dosage | . | . | . | . | . | 0.33 [0.03; 3.19] | . | . | . | . | . | 0.50 [0.05; 5.52] | . | . | . | . | . | . |
| 0.33 [0.03; 3.23] | 0.74 [0.08; 7.20] | 0.92 [0.11; 7.86] | 0.84 [0.06; 12.38] | Canagliflozin_ high_dosage | . | . | . | 0.72 [0.14; 3.63] | 0.70 [0.12; 4.12] | . | . | . | . | . | . | . | . | . | . | . | . |
| 0.31 [0.03; 2.82] | 0.70 [0.07; 6.63] | 0.86 [0.13; 5.69] | 0.79 [0.05; 11.45] | 0.94 [0.10; 8.58] | Inject_ semaglutide_ low_dosage | . | . | . | 0.50 [0.10; 2.42] | . | . | . | . | . | . | . | . | . | . | . | . |
| 0.23 [0.03; 1.56] | 0.51 [0.08; 3.48] | 0.64 [0.11; 3.70] | 0.58 [0.05; 6.37] | 0.69 [0.11; 4.46] | 0.74 [0.12; 4.61] | Exenatide | . | . | 0.78 [0.29; 2.10] | . | . | . | . | . | . | . | . | . | . | . | . |
| 0.24 [0.03; 2.35] | 0.54 [0.06; 5.25] | 0.68 [0.08; 5.73] | 0.62 [0.04; 9.04] | 0.74 [0.08; 6.79] | 0.78 [0.09; 7.05] | 1.06 [0.17; 6.75] | Oral_ semaglutide | . | 0.73 [0.15; 3.50] | . | . | . | . | . | . | . | . | . | . | . | . |
| 0.21 [0.02; 2.03] | 0.46 [0.05; 4.53] | 0.57 [0.07; 4.96] | 0.52 [0.03; 7.78] | 0.62 [0.15; 2.65] | 0.66 [0.07; 6.10] | 0.90 [0.14; 5.87] | 0.84 [0.09; 7.88] | Canagliflozin_ low_dosage | 1.42 [0.22; 9.18] | . | . | . | . | . | . | . | . | . | . | . | . |
| ***0.18 [0.03; 0.93]** | 0.40 [0.08; 2.06] | 0.50 [0.12; 2.13] | 0.45 [0.05; 4.02] | 0.54 [0.11; 2.62] | 0.57 [0.12; 2.70] | 0.78 [0.29; 2.10] | 0.73 [0.15; 3.50] | 0.87 [0.18; 4.30] | Placebo_ or_Control | 0.85 [0.20; 3.57] | 0.79 [0.33; 1.87] | 0.75 [0.17; 3.35] | 0.66 [0.03; 16.34] | 0.74 [0.31; 1.75] | 0.69 [0.26; 1.82] | 0.65 [0.17; 2.52] | 0.55 [0.08; 3.77] | 0.33 [0.01; 8.21] | 0.33 [0.01; 8.20] | 0.54 [0.26; 1.12] | 0.45 [0.19; 1.06] |
| 0.15 [0.02; 1.35] | 0.34 [0.04; 3.01] | 0.42 [0.05; 3.26] | 0.39 [0.03; 5.25] | 0.46 [0.05; 3.88] | 0.49 [0.06; 4.03] | 0.66 [0.12; 3.79] | 0.63 [0.08; 5.21] | 0.74 [0.09; 6.34] | 0.85 [0.20; 3.57] | Sotagliflozin | . | . | . | . | . | . | . | . | . | . | . |
| ***0.14 [0.02; 0.90]** | 0.32 [0.05; 2.02] | 0.39 [0.07; 2.13] | 0.36 [0.03; 3.74] | 0.43 [0.07; 2.58] | 0.45 [0.08; 2.67] | 0.62 [0.17; 2.29] | 0.58 [0.10; 3.46] | 0.69 [0.11; 4.22] | 0.79 [0.33; 1.87] | 0.93 [0.17; 4.94] | Liraglutide | . | . | . | . | . | . | . | . | . | . |
| 0.14 [0.02; 1.19] | 0.30 [0.03; 2.65] | 0.38 [0.05; 2.88] | 0.34 [0.03; 4.64] | 0.41 [0.05; 3.42] | 0.44 [0.05; 3.55] | 0.59 [0.10; 3.34] | 0.56 [0.07; 4.60] | 0.66 [0.08; 5.59] | 0.76 [0.18; 3.14] | 0.89 [0.12; 6.69] | 0.96 [0.18; 5.06] | Ertugliflozin_ low_dosage | . | . | . | 0.84 [0.23; 2.99] | . | . | . | . | . |
| 0.12 [0.00; 4.35] | 0.27 [0.01; 9.70] | 0.33 [0.01; 11.12] | 0.30 [0.01; 14.51] | 0.36 [0.01; 12.76] | 0.38 [0.01; 13.37] | 0.52 [0.02; 14.80] | 0.49 [0.01; 17.22] | 0.58 [0.02; 20.72] | 0.66 [0.03; 16.34] | 0.78 [0.02; 26.05] | 0.84 [0.03; 23.20] | 0.88 [0.03; 29.16] | Bexagliflozin | . | . | . | . | . | . | . | . |
| ***0.13 [0.02; 0.82]** | 0.29 [0.05; 1.86] | 0.36 [0.07; 1.80] | 0.33 [0.03; 3.45] | 0.40 [0.07; 2.37] | 0.42 [0.07; 2.41] | 0.57 [0.16; 2.10] | 0.54 [0.09; 3.18] | 0.64 [0.11; 3.89] | 0.74 [0.32; 1.70] | 0.86 [0.16; 4.54] | 0.93 [0.28; 3.10] | 0.97 [0.19; 5.06] | 1.11 [0.04; 30.32] | Inject_ semaglutide_ high_dosage | . | . | . | . | . | . | . |
| ***0.12 [0.02; 0.83]** | 0.28 [0.04; 1.86] | 0.34 [0.06; 1.98] | 0.31 [0.03; 2.90] | 0.37 [0.06; 2.38] | 0.40 [0.06; 2.46] | 0.54 [0.14; 2.15] | 0.51 [0.08; 3.19] | 0.60 [0.09; 3.90] | 0.69 [0.26; 1.82] | 0.81 [0.14; 4.57] | 0.88 [0.24; 3.20] | 0.91 [0.16; 5.10] | 1.04 [0.04; 29.53] | 0.94 [0.26; 3.38] | Empagliflozin_ low_dosage | . | . | . | . | . | . |
| ***0.11 [0.01; 0.90]** | 0.24 [0.03; 2.01] | 0.30 [0.04; 2.17] | 0.27 [0.02; 3.55] | 0.33 [0.04; 2.59] | 0.35 [0.04; 2.69] | 0.47 [0.09; 2.49] | 0.44 [0.06; 3.48] | 0.53 [0.07; 4.23] | 0.61 [0.16; 2.31] | 0.71 [0.10; 5.05] | 0.77 [0.16; 3.77] | 0.80 [0.23; 2.80] | 0.91 [0.03; 29.30] | 0.82 [0.17; 3.99] | 0.88 [0.17; 4.56] | Ertugliflozin_ high_dosage | . | . | . | . | . |
| 0.10 [0.01; 1.24] | 0.22 [0.02; 2.76] | 0.27 [0.02; 3.05] | 0.25 [0.01; 4.57] | 0.30 [0.02; 3.58] | 0.32 [0.03; 3.73] | 0.43 [0.05; 3.74] | 0.40 [0.03; 4.82] | 0.48 [0.04; 5.84] | 0.55 [0.08; 3.77] | 0.65 [0.06; 7.11] | 0.70 [0.08; 5.74] | 0.73 [0.07; 7.95] | 0.83 [0.02; 34.73] | 0.75 [0.09; 6.09] | 0.80 [0.09; 6.85] | 0.91 [0.09; 9.47] | Albiglutide | . | . | . | . |
| 0.06 [0.00; 2.19] | 0.13 [0.00; 4.88] | 0.17 [0.00; 5.59] | 0.15 [0.00; 7.30] | 0.18 [0.01; 6.42] | 0.19 [0.01; 6.72] | 0.26 [0.01; 7.44] | 0.25 [0.01; 8.66] | 0.29 [0.01; 10.42] | 0.33 [0.01; 8.21] | 0.39 [0.01; 13.10] | 0.42 [0.02; 11.66] | 0.44 [0.01; 14.66] | 0.50 [0.01; 46.57] | 0.45 [0.02; 12.42] | 0.48 [0.02; 13.69] | 0.55 [0.02; 17.74] | 0.61 [0.01; 25.40] | Efpeglenatide_ medium_dosage | 1.00 [0.10; 9.61] | . | . |
| 0.06 [0.00; 2.18] | 0.13 [0.00; 4.87] | 0.17 [0.00; 5.58] | 0.15 [0.00; 7.28] | 0.18 [0.01; 6.41] | 0.19 [0.01; 6.71] | 0.26 [0.01; 7.43] | 0.25 [0.01; 8.64] | 0.29 [0.01; 10.40] | 0.33 [0.01; 8.20] | 0.39 [0.01; 13.08] | 0.42 [0.02; 11.65] | 0.44 [0.01; 14.64] | 0.50 [0.01; 46.50] | 0.45 [0.02; 12.41] | 0.48 [0.02; 13.67] | 0.55 [0.02; 17.71] | 0.61 [0.01; 25.36] | 1.00 [0.10; 9.61] | Efpeglenatide_ high_dosage | . | . |
| ***0.10 [0.02; 0.58]** | 0.22 [0.04; 1.30] | 0.27 [0.05; 1.37] | 0.25 [0.02; 2.45] | 0.29 [0.05; 1.66] | 0.31 [0.06; 1.72] | 0.42 [0.12; 1.44] | 0.40 [0.07; 2.23] | 0.47 [0.08; 2.72] | 0.54 [0.26; 1.12] | 0.64 [0.13; 3.17] | 0.69 [0.22; 2.12] | 0.71 [0.14; 3.53] | 0.81 [0.03; 21.72] | 0.73 [0.24; 2.23] | 0.78 [0.23; 2.62] | 0.89 [0.19; 4.10] | 0.98 [0.13; 7.67] | 1.62 [0.06; 43.14] | 1.62 [0.06; 43.21] | Dapagliflozin_ high_dosage | . |
| ***0.08 [0.01; 0.51]** | 0.18 [0.03; 1.14] | 0.22 [0.04; 1.21] | 0.20 [0.02; 2.12] | 0.24 [0.04; 1.46] | 0.26 [0.04; 1.51] | 0.35 [0.09; 1.30] | 0.33 [0.06; 1.96] | 0.39 [0.06; 2.40] | 0.45 [0.19; 1.06] | 0.53 [0.10; 2.80] | 0.57 [0.17; 1.92] | 0.59 [0.11; 3.12] | 0.68 [0.02; 18.62] | 0.61 [0.18; 2.02] | 0.65 [0.18; 2.36] | 0.74 [0.15; 3.64] | 0.82 [0.10; 6.70] | 1.35 [0.05; 36.99] | 1.35 [0.05; 37.04] | 0.83 [0.27; 2.56] | Dulaglutide |

Data present as OR [95%CIs]. Pairwise (upper-right portion) and network (lower-left portion) meta-analysis results are presented as estimate effect sizes for the outcome of events of lymphoma. Interventions are reported in order of mean ranking of beneficially prophylactic effect on

events of lymphoma, and outcomes are expressed as odds ratio (OR) (95% confidence intervals) (95%CIs). For the pairwise meta-analyses, OR of less than 1 indicate that the treatment specified in the row got more beneficial effect than that specified in the column. For the network meta-analysis (NMA), OR of less than 1 indicate that the treatment specified in the column got more beneficial effect than that specified in the row. Bold results marked with * indicate statistical significance.

## Table S5B: League table of the primary outcome: subgroup of leukemia

| | | | | | | | | | | | | | | | | | | | | | | |
|---|---|---|---|---|---|---|---|---|---|---|---|---|---|---|---|---|---|---|---|---|---|---|
| Tirzepatide | . | . | . | . | . | . | . | . | . | . | . | . | . | 0.17 [0.01; 4.14] | . | . | . | . | . | . | . | . |
| 0.64 [0.01; 50.05] | Inject_ semaglutide_ low_dosage | . | . | . | . | . | . | . | 0.33 [0.01; 8.14] | . | . | . | . | 0.28 [0.01; 5.52] | . | . | . | . | . | . | . | . |
| 0.53 [0.01; 26.67] | 0.82 [0.02; 33.87] | Dapagliflozin_ medium_dosage | . | . | . | . | . | . | . | . | . | . | . | 0.32 [0.03; 3.09] | . | . | . | . | . | . | . | . |
| 0.51 [0.01; 26.04] | 0.80 [0.02; 33.06] | 0.98 [0.04; 24.14] | Dapagliflozin_ low_dosage | . | . | . | . | . | . | . | . | . | . | 0.33 [0.03; 3.16] | . | . | . | . | . | . | . | . |
| 0.46 [0.01; 18.54] | 0.72 [0.02; 23.23] | 0.87 [0.05; 16.26] | 0.89 [0.05; 16.66] | Canagliflozin_ high_dosage | . | 1.00 [0.06; 16.05] | . | . | . | . | . | . | . | 0.35 [0.05; 2.31] | . | . | . | . | . | . | . | . |
| 0.50 [0.01; 46.77] | 0.79 [0.01; 61.09] | 0.96 [0.02; 48.49] | 0.98 [0.02; 49.67] | 1.10 [0.03; 44.20] | Oral_ semaglutide | . | . | . | . | . | . | . | . | 0.33 [0.01; 8.18] | . | . | . | . | . | . | . | . |
| 0.31 [0.01; 11.10] | 0.49 [0.02; 13.79] | 0.60 [0.04; 9.38] | 0.61 [0.04; 9.61] | 0.68 [0.08; 6.12] | 0.62 [0.02; 21.95] | Canagliflozin_ low_dosage | . | . | . | . | . | . | . | 0.57 [0.12; 2.75] | . | . | . | . | . | . | . | . |
| 0.33 [0.01; 15.63] | 0.51 [0.01; 19.77] | 0.63 [0.03; 14.26] | 0.64 [0.03; 14.61] | 0.72 [0.04; 12.20] | 0.66 [0.01; 30.92] | 1.05 [0.07; 15.00] | Lixisenatide | 0.33 [0.01; 8.19] | . | . | . | . | . | 1.00 [0.06; 16.00] | . | . | . | . | . | . | . | . |
| 0.27 [0.01; 7.93] | 0.42 [0.02; 9.72] | 0.52 [0.04; 6.31] | 0.53 [0.04; 6.47] | 0.59 [0.07; 4.97] | 0.54 [0.02; 15.68] | 0.86 [0.13; 5.73] | 0.82 [0.09; 7.28] | Liraglutide | . | . | . | . | . | 0.56 [0.19; 1.67] | . | . | . | . | . | . | . | . |
| 0.26 [0.01; 9.56] | 0.40 [0.02; 9.24] | 0.49 [0.03; 8.19] | 0.50 [0.03; 8.39] | 0.56 [0.05; 6.75] | 0.51 [0.01; 18.91] | 0.82 [0.08; 8.09] | 0.78 [0.05; 11.82] | 0.95 [0.13; 6.87] | Inject_ semaglutide_ medium_dosage | 0.33 [0.01; 8.21] | . | . | . | 0.86 [0.13; 5.83] | . | . | . | . | . | . | . | . |
| 0.19 [0.01; 5.34] | 0.29 [0.01; 6.38] | 0.36 [0.03; 4.20] | 0.37 [0.03; 4.30] | 0.41 [0.05; 3.28] | 0.37 [0.01; 10.56] | 0.60 [0.10; 3.76] | 0.57 [0.05; 5.97] | 0.70 [0.17; 2.90] | 0.73 [0.12; 4.36] | Inject_ semaglutide_ high_dosage | . | . | . | 0.83 [0.31; 2.21] | . | . | . | . | . | . | . | . |
| 0.17 [0.00; 43.19] | 0.26 [0.00; 58.31] | 0.32 [0.00; 50.64] | 0.33 [0.00; 51.88] | 0.37 [0.00; 48.70] | 0.33 [0.00; 85.46] | 0.54 [0.00; 64.59] | 0.51 [0.00; 76.41] | 0.62 [0.01; 65.01] | 0.65 [0.01; 80.92] | 0.89 [0.01; 91.07] | Efpeglenatide_ high_dosage | . | . | . | . | . | . | . | . | 0.33 [0.01; 8.20] | . | . |
| 0.17 [0.00; 7.20] | 0.26 [0.01; 9.05] | 0.32 [0.02; 6.41] | 0.33 [0.02; 6.56] | 0.37 [0.02; 5.41] | 0.33 [0.01; 14.23] | 0.54 [0.04; 6.58] | 0.51 [0.03; 9.31] | 0.62 [0.07; 5.77] | 0.65 [0.05; 8.49] | 0.89 [0.10; 7.87] | 1.00 [0.01; 138.73] | Sotagliflozin | . | 1.00 [0.14; 7.09] | . | . | . | . | . | . | . | . |
| 0.17 [0.00; 5.92] | 0.26 [0.01; 7.35] | 0.32 [0.02; 5.01] | 0.33 [0.02; 5.13] | 0.36 [0.03; 4.10] | 0.33 [0.01; 11.71] | 0.53 [0.06; 4.88] | 0.51 [0.04; 7.21] | 0.62 [0.09; 4.10] | 0.65 [0.07; 6.35] | 0.88 [0.14; 5.54] | 0.99 [0.01; 119.55] | 0.99 [0.08; 12.23] | Empagliflozin_ high_dosage | 0.79 [0.08; 7.59] | . | . | . | . | . | . | . | 0.45 [0.07; 2.65] |
| 0.17 [0.01; 4.14] | 0.26 [0.01; 5.00] | 0.32 [0.03; 3.09] | 0.33 [0.03; 3.16] | 0.37 [0.06; 2.32] | 0.33 [0.01; 8.18] | 0.54 [0.11; 2.56] | 0.51 [0.06; 4.35] | 0.62 [0.21; 1.80] | 0.65 [0.12; 3.42] | 0.89 [0.34; 2.30] | 1.00 [0.01; 92.33] | 1.00 [0.14; 7.09] | 1.01 [0.21; 4.82] | Placebo_ or_Control | 0.93 [0.53; 1.63] | 0.80 [0.20; 3.23] | 0.72 [0.29; 1.80] | 0.50 [0.05; 5.52] | 0.60 [0.14; 2.51] | 0.33 [0.01; 8.21] | 0.33 [0.03; 3.21] | 0.35 [0.10; 1.22] |
| 0.16 [0.01; 4.04] | 0.24 [0.01; 4.90] | 0.30 [0.03; 3.07] | 0.30 [0.03; 3.15] | 0.34 [0.05; 2.34] | 0.31 [0.01; 7.99] | 0.50 [0.09; 2.63] | 0.47 [0.05; 4.34] | 0.58 [0.17; 1.92] | 0.60 [0.10; 3.49] | 0.83 [0.27; 2.50] | 0.93 [0.01; 88.86] | 0.93 [0.12; 7.14] | 0.93 [0.18; 4.95] | 0.93 [0.53; 1.63] | Dapagliflozin_ high_dosage | . | . | . | . | . | . | . |
| 0.14 [0.00; 4.44] | 0.21 [0.01; 5.49] | 0.26 [0.02; 3.67] | 0.26 [0.02; 3.76] | 0.29 [0.03; 2.97] | 0.27 [0.01; 8.79] | 0.43 [0.05; 3.49] | 0.41 [0.03; 5.27] | 0.50 [0.09; 2.87] | 0.52 [0.06; 4.56] | 0.72 [0.13; 3.85] | 0.80 [0.01; 91.49] | 0.80 [0.07; 8.89] | 0.81 [0.10; 6.57] | 0.80 [0.20; 3.23] | 0.87 [0.19; 3.88] | Albiglutide | . | . | . | . | . | . |
| 0.12 [0.00; 3.40] | 0.19 [0.01; 4.16] | 0.23 [0.02; 2.67] | 0.24 [0.02; 2.73] | 0.26 [0.03; 2.08] | 0.24 [0.01; 6.73] | 0.39 [0.06; 2.37] | 0.37 [0.04; 3.79] | 0.45 [0.11; 1.82] | 0.47 [0.07; 3.13] | 0.64 [0.17; 2.40] | 0.72 [0.01; 73.22] | 0.72 [0.08; 6.29] | 0.73 [0.12; 4.47] | 0.72 [0.29; 1.80] | 0.78 [0.27; 2.27] | 0.90 [0.17; 4.74] | Exenatide | . | . | . | . | . |
| 0.08 [0.00; 4.61] | 0.13 [0.00; 5.87] | 0.16 [0.01; 4.34] | 0.16 [0.01; 4.45] | 0.18 [0.01; 3.78] | 0.17 [0.00; 9.11] | 0.27 [0.02; 4.70] | 0.25 [0.01; 6.36] | 0.31 [0.02; 4.28] | 0.32 [0.02; 6.02] | 0.44 [0.03; 5.88] | 0.50 [0.00; 83.89] | 0.50 [0.02; 11.08] | 0.50 [0.03; 8.85] | 0.50 [0.05; 5.52] | 0.54 [0.05; 6.34] | 0.62 [0.04; 9.96] | 0.69 [0.05; 9.00] | Ertugliflozin_ low_dosage | . | . | 0.67 [0.11; 3.99] | . |
| 0.10 [0.00; 3.37] | 0.16 [0.01; 4.17] | 0.19 [0.01; 2.80] | 0.20 [0.01; 2.87] | 0.22 [0.02; 2.27] | 0.20 [0.01; 6.66] | 0.32 [0.04; 2.68] | 0.30 [0.02; 4.02] | 0.37 [0.06; 2.21] | 0.39 [0.04; 3.49] | 0.53 [0.10; 2.97] | 0.60 [0.01; 69.03] | 0.60 [0.05; 6.79] | 0.60 [0.07; 5.04] | 0.60 [0.14; 2.51] | 0.64 [0.14; 3.00] | 0.75 [0.10; 5.48] | 0.83 [0.15; 4.52] | 1.20 [0.07; 19.63] | Dulaglutide | . | . | . |
| 0.06 [0.00; 5.21] | 0.09 [0.00; 6.80] | 0.11 [0.00; 5.40] | 0.11 [0.00; 5.53] | 0.12 [0.00; 4.92] | 0.11 [0.00; 10.30] | 0.18 [0.01; 6.32] | 0.17 [0.00; 8.02] | 0.21 [0.01; 6.05] | 0.22 [0.01; 8.00] | 0.30 [0.01; 8.39] | 0.33 [0.01; 8.20] | 0.33 [0.01; 14.26] | 0.34 [0.01; 11.88] | 0.33 [0.01; 8.21] | 0.36 [0.01; 9.28] | 0.42 [0.01; 13.63] | 0.46 [0.02; 12.88] | 0.67 [0.01; 36.57] | 0.56 [0.02; 18.60] | Efpeglenatide_ medium_dosage | . | . |
| 0.06 [0.00; 2.83] | 0.09 [0.00; 3.60] | 0.11 [0.00; 2.62] | 0.11 [0.00; 2.69] | 0.12 [0.01; 2.26] | 0.11 [0.00; 5.60] | 0.18 [0.01; 2.80] | 0.17 [0.01; 3.83] | 0.21 [0.02; 2.52] | 0.22 [0.01; 3.59] | 0.30 [0.03; 3.45] | 0.33 [0.00; 52.52] | 0.33 [0.02; 6.65] | 0.34 [0.02; 5.26] | 0.33 [0.03; 3.21] | 0.36 [0.03; 3.70] | 0.41 [0.03; 5.90] | 0.46 [0.04; 5.28] | 0.67 [0.11; 3.99] | 0.56 [0.04; 8.10] | 1.00 [0.02; 50.30] | Ertugliflozin_ high_dosage | . |
| 0.06 [0.00; 1.92] | 0.10 [0.00; 2.35] | 0.12 [0.01; 1.54] | 0.12 [0.01; 1.58] | 0.14 [0.02; 1.22] | 0.13 [0.00; 3.79] | 0.20 [0.03; 1.41] | 0.19 [0.02; 2.20] | 0.24 [0.05; 1.12] | 0.25 [0.03; 1.85] | 0.34 [0.08; 1.49] | 0.38 [0.00; 40.46] | 0.38 [0.04; 3.67] | 0.38 [0.09; 1.66] | 0.38 [0.12; 1.19] | 0.41 [0.11; 1.46] | 0.47 [0.08; 2.85] | 0.53 [0.12; 2.26] | 0.76 [0.05; 10.85] | 0.63 [0.10; 3.96] | 1.14 [0.04; 34.04] | 1.14 [0.09; 14.39] | Empagliflozin_ low_dosage |

Data present as OR [95%CIs]. Pairwise (upper-right portion) and network (lower-left portion) meta-analysis results are presented as estimate

effect sizes for the outcome of events of leukemia. Interventions are reported in order of mean ranking of beneficially prophylactic effect on events of leukemia, and outcomes are expressed as odds ratio (OR) (95% confidence intervals) (95%CIs). For the pairwise meta-analyses, OR of less than 1 indicate that the treatment specified in the row got more beneficial effect than that specified in the column. For the network meta-analysis (NMA), OR of less than 1 indicate that the treatment specified in the column got more beneficial effect than that specified in the row. Bold results marked with * indicate statistical significance.

## Table S5C: League table of the primary outcome: subgroup of myeloma

| | | | | | | | | | | | | | | | | | | |
|---|---|---|---|---|---|---|---|---|---|---|---|---|---|---|---|---|---|---|
| Tirzepatide | . | . | . | . | . | . | . | . | . | . | 0.11 [0.00; 2.78] | . | . | . | . | . | . | . |
| 0.34 [0.01; 19.50] | Ertugliflozin_ low_dosage | . | . | . | . | . | . | . | . | 0.33 [0.01; 8.11] | 0.33 [0.01; 8.18] | . | . | . | . | . | . | . |
| 0.34 [0.00; 31.41] | 1.00 [0.02; 57.36] | Canagliflozin_ low_dosage | . | . | . | . | . | . | . | . | 0.33 [0.01; 8.17] | . | . | . | . | . | . | . |
| 0.34 [0.00; 31.37] | 1.00 [0.02; 57.29] | 1.00 [0.01; 92.38] | Canagliflozin_ high_dosage | . | . | . | . | . | . | . | 0.33 [0.01; 8.18] | . | . | . | . | . | . | . |
| 0.22 [0.01; 6.47] | 0.64 [0.04; 9.74] | 0.64 [0.02; 19.05] | 0.64 [0.02; 19.07] | Dapagliflozin_ high_dosage | . | . | . | . | . | . | 0.52 [0.17; 1.57] | . | . | . | . | . | . | . |
| 0.21 [0.01; 6.57] | 0.61 [0.04; 10.07] | 0.61 [0.02; 19.34] | 0.61 [0.02; 19.36] | 0.94 [0.17; 5.29] | Liraglutide | . | . | . | . | . | 0.60 [0.14; 2.51] | . | 0.33 [0.01; 8.08] | . | . | . | . | . |
| 0.17 [0.00; 15.72] | 0.50 [0.01; 28.72] | 0.50 [0.01; 46.31] | 0.50 [0.01; 46.35] | 0.78 [0.03; 23.03] | 0.83 [0.03; 26.38] | Inject_ semaglutide_ low_dosage | . | . | . | . | 0.66 [0.03; 16.34] | . | . | . | . | . | . | . |
| 0.17 [0.00; 15.65] | 0.50 [0.01; 28.58] | 0.50 [0.01; 46.08] | 0.50 [0.01; 46.13] | 0.77 [0.03; 22.92] | 0.82 [0.03; 26.25] | 1.00 [0.01; 92.11] | Inject_ semaglutide_ medium_dosage | . | . | . | 0.67 [0.03; 16.42] | . | . | . | . | . | . | . |
| 0.15 [0.00; 8.56] | 0.45 [0.01; 14.59] | 0.45 [0.01; 25.21] | 0.45 [0.01; 25.23] | 0.70 [0.05; 10.17] | 0.75 [0.05; 11.88] | 0.91 [0.02; 50.39] | 0.91 [0.02; 50.63] | Empagliflozin_ high_dosage | . | . | 1.00 [0.06; 15.94] | . | . | 0.50 [0.05; 5.52] | . | . | . | . |
| 0.14 [0.00; 4.12] | 0.40 [0.03; 6.25] | 0.40 [0.01; 12.14] | 0.40 [0.01; 12.15] | 0.62 [0.12; 3.15] | 0.66 [0.11; 3.90] | 0.80 [0.03; 24.27] | 0.80 [0.03; 24.39] | 0.88 [0.06; 13.15] | Inject_ semaglutide_ high_dosage | . | 0.83 [0.25; 2.73] | . | . | . | . | . | . | . |
| 0.11 [0.00; 4.94] | 0.33 [0.03; 3.94] | 0.33 [0.01; 14.55] | 0.33 [0.01; 14.56] | 0.51 [0.05; 5.15] | 0.54 [0.05; 6.11] | 0.66 [0.01; 29.09] | 0.66 [0.01; 29.23] | 0.73 [0.03; 17.17] | 0.82 [0.08; 8.63] | Ertugliflozin_ high_dosage | 1.01 [0.10; 9.73] | . | . | . | . | . | . | . |
| 0.11 [0.00; 2.78] | 0.33 [0.03; 3.97] | 0.33 [0.01; 8.17] | 0.33 [0.01; 8.18] | 0.52 [0.17; 1.57] | 0.55 [0.15; 2.06] | 0.66 [0.03; 16.34] | 0.67 [0.03; 16.42] | 0.73 [0.06; 8.32] | 0.83 [0.25; 2.73] | 1.01 [0.13; 7.67] | Placebo_ or_Control | 0.94 [0.11; 7.62] | 1.00 [0.29; 3.44] | 0.79 [0.21; 2.97] | 0.58 [0.06; 5.54] | 0.33 [0.01; 8.19] | 0.33 [0.01; 8.18] | 0.39 [0.09; 1.61] |
| 0.11 [0.00; 4.85] | 0.31 [0.01; 8.01] | 0.31 [0.01; 14.29] | 0.31 [0.01; 14.30] | 0.48 [0.05; 5.18] | 0.51 [0.04; 6.13] | 0.62 [0.01; 28.57] | 0.62 [0.01; 28.71] | 0.69 [0.03; 16.99] | 0.78 [0.07; 8.67] | 0.95 [0.05; 17.46] | 0.94 [0.11; 7.62] | Sotagliflozin | . | . | . | . | . | . |
| 0.11 [0.00; 3.18] | 0.31 [0.02; 4.81] | 0.31 [0.01; 9.37] | 0.31 [0.01; 9.38] | 0.48 [0.10; 2.41] | 0.51 [0.10; 2.61] | 0.62 [0.02; 18.72] | 0.62 [0.02; 18.82] | 0.68 [0.05; 10.13] | 0.78 [0.15; 4.11] | 0.94 [0.09; 9.77] | 0.93 [0.29; 3.00] | 1.00 [0.09; 10.99] | Exenatide | . | . | . | . | . |
| 0.09 [0.00; 2.86] | 0.26 [0.02; 4.39] | 0.26 [0.01; 8.43] | 0.26 [0.01; 8.44] | 0.41 [0.07; 2.30] | 0.44 [0.07; 2.82] | 0.53 [0.02; 16.85] | 0.53 [0.02; 16.94] | 0.58 [0.06; 5.85] | 0.66 [0.11; 3.90] | 0.80 [0.07; 9.01] | 0.79 [0.21; 2.97] | 0.85 [0.07; 10.12] | 0.85 [0.15; 4.97] | Empagliflozin_ low_dosage | . | . | . | . |
| 0.07 [0.00; 3.28] | 0.19 [0.01; 5.51] | 0.19 [0.00; 9.67] | 0.19 [0.00; 9.68] | 0.30 [0.02; 3.71] | 0.32 [0.02; 4.35] | 0.38 [0.01; 19.33] | 0.38 [0.01; 19.43] | 0.42 [0.02; 11.70] | 0.48 [0.04; 6.19] | 0.58 [0.03; 12.15] | 0.58 [0.06; 5.54] | 0.62 [0.03; 13.49] | 0.62 [0.05; 7.90] | 0.73 [0.05; 9.97] | Oral_ semaglutide | . | . | . |
| 0.04 [0.00; 3.48] | 0.11 [0.00; 6.36] | 0.11 [0.00; 10.26] | 0.11 [0.00; 10.27] | 0.17 [0.01; 5.10] | 0.18 [0.01; 5.84] | 0.22 [0.00; 20.51] | 0.22 [0.00; 20.61] | 0.24 [0.00; 13.60] | 0.28 [0.01; 8.44] | 0.34 [0.01; 14.89] | 0.33 [0.01; 8.19] | 0.36 [0.01; 16.36] | 0.36 [0.01; 10.80] | 0.42 [0.01; 13.38] | 0.58 [0.01; 29.18] | Albiglutide | . | . |
| 0.04 [0.00; 3.48] | 0.11 [0.00; 6.36] | 0.11 [0.00; 10.25] | 0.11 [0.00; 10.26] | 0.17 [0.01; 5.10] | 0.18 [0.01; 5.84] | 0.22 [0.00; 20.49] | 0.22 [0.00; 20.59] | 0.24 [0.00; 13.59] | 0.28 [0.01; 8.43] | 0.34 [0.01; 14.87] | 0.33 [0.01; 8.18] | 0.36 [0.01; 16.35] | 0.36 [0.01; 10.80] | 0.42 [0.01; 13.37] | 0.58 [0.01; 29.16] | 1.00 [0.01; 92.38] | Lixisenatide | . |
| 0.04 [0.00; 1.45] | 0.13 [0.01; 2.25] | 0.13 [0.00; 4.27] | 0.13 [0.00; 4.28] | 0.20 [0.03; 1.22] | 0.21 [0.03; 1.48] | 0.26 [0.01; 8.54] | 0.26 [0.01; 8.58] | 0.28 [0.02; 4.74] | 0.32 [0.05; 2.06] | 0.39 [0.03; 4.65] | 0.39 [0.09; 1.61] | 0.41 [0.03; 5.22] | 0.41 [0.07; 2.62] | 0.48 [0.07; 3.40] | 0.67 [0.05; 9.73] | 1.15 [0.03; 38.49] | 1.16 [0.03; 38.53] | Dulaglutide |

Data present as OR [95%CIs]. Pairwise (upper-right portion) and network (lower-left portion) meta-analysis results are presented as estimate effect sizes for the outcome of events of myeloma. Interventions are reported in order of mean ranking of beneficially prophylactic effect on events of myeloma, and outcomes are expressed as odds ratio (OR) (95% confidence intervals) (95%CIs). For the pairwise meta-analyses, OR of less than 1 indicate that the treatment specified in the row got more beneficial effect than that specified in the column. For the network meta-analysis (NMA), OR of less than 1 indicate that the treatment specified in the column got more beneficial effect than that specified in the row. Bold results marked with * indicate statistical significance.

# Table S5D: League table of NMA of safety profile: drop-out rate

| | | | | | | | | | | | | | | | | | | | | | | | |
|---|---|---|---|---|---|---|---|---|---|---|---|---|---|---|---|---|---|---|---|---|---|---|---|
| Liraglutide | . | . | . | . | . | . | . | . | . | . | 0.68 [0.35; 1.33] | . | 0.91 [0.35; 2.38] | . | . | . | . | . | . | . | . | *0.47 [0.34; 0.64] | . |
| 0.90 [0.60; 1.34] | Tirzepatide | . | 0.89 [0.46; 1.71] | . | . | . | . | . | . | . | . | . | . | . | . | . | . | . | . | . | . | *0.55 [0.41; 0.75] | . |
| 0.82 [0.42; 1.61] | 0.91 [0.47; 1.77] | Inject_semaglutide_low_dosage | 0.94 [0.46; 1.91] | . | . | . | . | . | . | . | . | . | . | . | . | . | . | . | . | . | . | 0.63 [0.33; 1.21] | . |
| 0.77 [0.47; 1.27] | 0.86 [0.56; 1.33] | 0.94 [0.50; 1.79] | Inject_semaglutide_medium_dosage | 1.00 [0.41; 2.48] | . | . | . | . | . | . | . | . | . | . | . | . | . | . | . | . | . | 0.63 [0.38; 1.06] | . |
| 0.71 [0.47; 1.07] | 0.79 [0.52; 1.19] | 0.86 [0.44; 1.70] | 0.91 [0.56; 1.49] | Inject_semaglutide_high_dosage | . | . | . | . | . | . | . | . | . | . | . | . | . | . | . | . | . | *0.71 [0.52; 0.97] | . |
| 0.71 [0.44; 1.15] | 0.79 [0.48; 1.29] | 0.86 [0.41; 1.80] | 0.92 [0.52; 1.62] | 1.00 [0.61; 1.66] | Canagliflozin_high_dosage | . | 0.86 [0.50; 1.49] | . | . | . | . | . | . | . | . | . | . | . | . | . | . | 0.69 [0.46; 1.05] | . |
| 0.68 [0.46; 1.00] | 0.76 [0.51; 1.12] | 0.83 [0.42; 1.62] | 0.88 [0.54; 1.44] | 0.96 [0.64; 1.45] | 0.96 [0.59; 1.55] | Empagliflozin_high_dosage | . | . | . | . | . | . | . | . | 0.87 [0.61; 1.22] | . | . | . | . | . | . | *0.72 [0.53; 0.96] | . |
| 0.64 [0.40; 1.02] | 0.71 [0.45; 1.14] | 0.78 [0.38; 1.60] | 0.83 [0.48; 1.44] | 0.91 [0.56; 1.47] | 0.91 [0.57; 1.43] | 0.95 [0.60; 1.50] | Canagliflozin_low_dosage | . | . | . | . | . | . | . | . | . | . | . | . | . | . | 0.75 [0.52; 1.10] | . |
| *0.63 [0.41; 0.98] | 0.71 [0.46; 1.09] | 0.77 [0.38; 1.55] | 0.82 [0.48; 1.39] | 0.90 [0.57; 1.41] | 0.89 [0.53; 1.50] | 0.93 [0.61; 1.43] | 0.99 [0.60; 1.62] | Dapagliflozin_high_dosage | 0.88 [0.49; 1.58] | 0.87 [0.48; 1.56] | . | . | . | . | . | . | . | . | . | . | . | 0.79 [0.57; 1.10] | . |
| 0.63 [0.35; 1.13] | 0.70 [0.39; 1.26] | 0.77 [0.34; 1.72] | 0.82 [0.42; 1.58] | 0.89 [0.49; 1.63] | 0.89 [0.46; 1.71] | 0.93 [0.52; 1.67] | 0.98 [0.52; 1.85] | 1.00 [0.59; 1.69] | Dapagliflozin_low_dosage | 0.99 [0.56; 1.74] | . | . | . | . | . | . | . | . | . | . | . | 0.72 [0.42; 1.26] | . |
| 0.62 [0.35; 1.11] | 0.69 [0.38; 1.24] | 0.75 [0.34; 1.68] | 0.80 [0.42; 1.54] | 0.88 [0.48; 1.59] | 0.87 [0.46; 1.67] | 0.91 [0.51; 1.63] | 0.96 [0.51; 1.81] | 0.98 [0.58; 1.65] | 0.98 [0.55; 1.74] | Dapagliflozin_medium_dosage | . | . | . | . | . | . | . | . | . | . | . | 0.73 [0.42; 1.27] | . |
| *0.61 [0.40; 0.95] | 0.68 [0.41; 1.12] | 0.75 [0.36; 1.56] | 0.79 [0.44; 1.41] | 0.87 [0.52; 1.44] | 0.86 [0.49; 1.53] | 0.90 [0.55; 1.47] | 0.95 [0.55; 1.65] | 0.97 [0.57; 1.63] | 0.97 [0.50; 1.87] | 0.99 [0.51; 1.90] | Exenatide | . | . | . | . | . | . | . | . | . | . | 0.86 [0.53; 1.41] | . |
| *0.60 [0.38; 0.96] | 0.67 [0.42; 1.07] | 0.74 [0.36; 1.51] | 0.78 [0.45; 1.36] | 0.85 [0.53; 1.38] | 0.85 [0.49; 1.47] | 0.89 [0.56; 1.41] | 0.94 [0.56; 1.59] | 0.95 [0.58; 1.57] | 0.96 [0.51; 1.81] | 0.97 [0.52; 1.84] | 0.99 [0.57; 1.71] | Ertugliflozin_low_dosage | . | . | . | . | . | 0.90 [0.62; 1.30] | . | . | . | 0.83 [0.57; 1.21] | . |
| 0.60 [0.36; 1.00] | 0.67 [0.38; 1.15] | 0.73 [0.34; 1.58] | 0.77 [0.41; 1.45] | 0.85 [0.48; 1.49] | 0.84 [0.46; 1.56] | 0.88 [0.51; 1.52] | 0.93 [0.51; 1.70] | 0.94 [0.53; 1.68] | 0.95 [0.47; 1.91] | 0.97 [0.48; 1.94] | 0.98 [0.53; 1.80] | 0.99 [0.54; 1.81] | Lixisenatide | . | . | . | . | . | . | . | . | 0.95 [0.56; 1.62] | . |
| 0.57 [0.27; 1.19] | 0.64 [0.30; 1.33] | 0.70 [0.28; 1.74] | 0.74 [0.33; 1.64] | 0.81 [0.38; 1.70] | 0.81 [0.37; 1.77] | 0.84 [0.40; 1.75] | 0.89 [0.41; 1.93] | 0.90 [0.42; 1.92] | 0.91 [0.39; 2.13] | 0.92 [0.39; 2.16] | 0.93 [0.42; 2.06] | 0.95 [0.44; 2.06] | 0.96 [0.42; 2.18] | Bexagliflozin | . | . | . | . | . | . | . | 0.88 [0.44; 1.73] | . |
| *0.57 [0.40; 0.81] | *0.64 [0.45; 0.91] | 0.70 [0.36; 1.34] | 0.74 [0.47; 1.18] | 0.81 [0.56; 1.18] | 0.81 [0.52; 1.27] | 0.84 [0.63; 1.13] | 0.89 [0.58; 1.36] | 0.90 [0.61; 1.34] | 0.91 [0.52; 1.58] | 0.93 [0.53; 1.61] | 0.94 [0.59; 1.48] | 0.95 [0.62; 1.46] | 0.96 [0.57; 1.60] | 1.00 [0.49; 2.04] | Empagliflozin_low_dosage | . | . | . | . | . | . | 0.86 [0.69; 1.07] | . |
| *0.56 [0.38; 0.82] | *0.62 [0.42; 0.91] | 0.68 [0.35; 1.33] | 0.72 [0.44; 1.17] | 0.79 [0.52; 1.18] | 0.78 [0.48; 1.27] | 0.82 [0.56; 1.20] | 0.87 [0.55; 1.37] | 0.88 [0.57; 1.34] | 0.88 [0.49; 1.57] | 0.90 [0.50; 1.60] | 0.91 [0.56; 1.48] | 0.92 [0.58; 1.46] | 0.93 [0.54; 1.59] | 0.97 [0.47; 2.02] | 0.97 [0.69; 1.37] | Albiglutide | . | . | . | . | . | 0.90 [0.69; 1.18] | . |
| *0.55 [0.36; 0.82] | *0.61 [0.40; 0.92] | 0.67 [0.34; 1.32] | 0.71 [0.43; 1.18] | 0.77 [0.50; 1.19] | 0.77 [0.47; 1.27] | 0.81 [0.54; 1.21] | 0.85 [0.53; 1.37] | 0.86 [0.55; 1.35] | 0.87 [0.48; 1.57] | 0.88 [0.49; 1.60] | 0.89 [0.54; 1.48] | 0.91 [0.56; 1.46] | 0.91 [0.52; 1.60] | 0.96 [0.45; 2.01] | 0.95 [0.66; 1.38] | 0.98 [0.66; 1.47] | Sotagliflozin | . | . | . | . | 0.92 [0.68; 1.24] | . |
| *0.54 [0.34; 0.86] | *0.60 [0.38; 0.96] | 0.66 [0.32; 1.35] | 0.70 [0.40; 1.22] | 0.77 [0.47; 1.24] | 0.76 [0.44; 1.32] | 0.80 [0.50; 1.26] | 0.84 [0.50; 1.42] | 0.85 [0.52; 1.40] | 0.86 [0.46; 1.62] | 0.87 [0.47; 1.64] | 0.88 [0.51; 1.53] | 0.90 [0.62; 1.31] | 0.90 [0.50; 1.64] | 0.95 [0.44; 2.05] | 0.95 [0.62; 1.45] | 0.97 [0.62; 1.54] | 0.99 [0.62; 1.60] | Ertugliflozin_high_dosage | . | . | . | 0.93 [0.64; 1.35] | . |
| *0.52 [0.29; 0.91] | 0.58 [0.33; 1.02] | 0.63 [0.29; 1.39] | 0.67 [0.35; 1.27] | 0.73 [0.41; 1.31] | 0.73 [0.39; 1.38] | 0.76 [0.43; 1.34] | 0.81 [0.44; 1.50] | 0.82 [0.45; 1.48] | 0.82 [0.40; 1.68] | 0.84 [0.41; 1.71] | 0.85 [0.45; 1.61] | 0.86 [0.46; 1.60] | 0.86 [0.44; 1.71] | 0.91 [0.39; 2.10] | 0.90 [0.53; 1.55] | 0.93 [0.53; 1.64] | 0.95 [0.53; 1.69] | 0.96 [0.52; 1.77] | Efpeglenatide_medium_dosage | 0.98 [0.60; 1.61] | . | 0.97 [0.59; 1.59] | . |
| *0.51 [0.29; 0.90] | 0.56 [0.32; 1.00] | 0.62 [0.28; 1.36] | 0.66 [0.35; 1.25] | 0.72 [0.40; 1.29] | 0.72 [0.38; 1.35] | 0.75 [0.42; 1.32] | 0.79 [0.43; 1.47] | 0.80 [0.44; 1.45] | 0.80 [0.39; 1.64] | 0.82 [0.40; 1.67] | 0.83 [0.44; 1.57] | 0.84 [0.45; 1.57] | 0.85 [0.43; 1.68] | 0.89 [0.38; 2.06] | 0.89 [0.52; 1.52] | 0.91 [0.52; 1.61] | 0.93 [0.52; 1.66] | 0.94 [0.51; 1.74] | 0.98 [0.60; 1.61] | Efpeglenatide_high_dosage | . | 0.99 [0.60; 1.62] | . |
| *0.51 [0.32; 0.82] | *0.57 [0.35; 0.92] | 0.62 [0.30; 1.29] | 0.66 [0.38; 1.16] | 0.72 [0.44; 1.18] | 0.72 [0.41; 1.26] | 0.75 [0.47; 1.21] | 0.80 [0.47; 1.36] | 0.81 [0.48; 1.34] | 0.81 [0.43; 1.54] | 0.82 [0.43; 1.57] | 0.83 [0.48; 1.46] | 0.85 [0.49; 1.45] | 0.85 [0.46; 1.57] | 0.89 [0.41; 1.95] | 0.89 [0.57; 1.39] | 0.92 [0.57; 1.47] | 0.93 [0.57; 1.53] | 0.94 [0.55; 1.61] | 0.99 [0.53; 1.85] | 1.01 [0.54; 1.89] | Dulaglutide | 0.98 [0.67; 1.44] | . |
| *0.50 [0.38; 0.66] | *0.56 [0.42; 0.74] | 0.61 [0.33; 1.13] | *0.65 [0.43; 0.98] | *0.71 [0.52; 0.96] | 0.71 [0.47; 1.05] | *0.74 [0.56; 0.97] | 0.78 [0.54; 1.13] | 0.79 [0.57; 1.10] | 0.79 [0.47; 1.33] | 0.81 [0.48; 1.35] | 0.82 [0.54; 1.23] | 0.83 [0.57; 1.21] | 0.84 [0.52; 1.34] | 0.88 [0.44; 1.73] | 0.87 [0.71; 1.08] | 0.90 [0.69; 1.18] | 0.92 [0.68; 1.24] | 0.93 [0.64; 1.34] | 0.97 [0.59; 1.59] | 0.99 [0.60; 1.62] | 0.98 [0.67; 1.44] | Placebo_or_Control | 0.63 [0.34; 1.17] |
| *0.32 [0.16; 0.62] | *0.35 [0.18; 0.69] | *0.38 [0.16; 0.92] | *0.41 [0.19; 0.86] | *0.45 [0.22; 0.89] | *0.45 [0.21; 0.93] | *0.47 [0.24; 0.92] | 0.49 [0.24; 1.01] | 0.50 [0.25; 1.00] | 0.50 [0.22; 1.12] | 0.51 [0.23; 1.14] | 0.52 [0.25; 1.08] | 0.52 [0.25; 1.08] | 0.53 [0.24; 1.15] | 0.55 [0.22; 1.38] | 0.55 [0.29; 1.06] | 0.57 [0.29; 1.12] | 0.58 [0.29; 1.15] | 0.58 [0.28; 1.20] | 0.61 [0.28; 1.35] | 0.62 [0.28; 1.38] | 0.62 [0.30; 1.28] | 0.63 [0.34; 1.17] | Oral_semaglutide |

Data present as OR [95%CIs]. Pairwise (upper-right portion) and network (lower-left portion) meta-analysis results are presented as estimate effect sizes for the outcome of safety profile (drop-out rate). Interventions are reported in order of mean ranking of safety, and outcomes are expressed as odds ratio (OR) (95% confidence intervals) (95%CIs). For the pairwise meta-analyses, OR of less than 1 indicate that the treatment specified in the row got more safety than that specified in the column. For the network meta-

analysis (NMA), OR of less than 1 indicate that the treatment specified in the column got more safety than that specified in the row. Bold results marked with * indicate statistical significance.

*Abbreviation: 95%CIs: 95% confidence intervals; GLP-1 agonist: glucagon-like peptide-1 agonist; NMA: network meta-analysis; OR: odds ratio; RCT: randomized controlled trial; SGLT2 inhibitor: sodium–glucose cotransporter 2 inhibitor*

# Table S6A: SUCRA (Surface under the cumulative ranking) of primary outcome: overall hematologic malignancy

| Treatment | Rank 1 | Rank 2 | Rank 3 | Rank 4 | Rank 5 | Rank 6 | Rank 7 | Rank 8 | Rank 9 | Rank 10 | Rank 11 | Rank 12 | Rank 13 | Rank 14 | Rank 15 | Rank 16 | Rank 17 | Rank 18 | Rank 19 | Rank 20 | Rank 21 | Rank 22 | Rank 23 | Rank 24 | SUCRA |
|---|---|---|---|---|---|---|---|---|---|---|---|---|---|---|---|---|---|---|---|---|---|---|---|---|---|
| Albiglutide | 0 | 0 | 1.25E-05 | 0.000775 | 0.0023875 | 0.0053375 | 0.012175 | 0.0178375 | 0.0239 | 0.0275125 | 0.02905 | 0.0304 | 0.0348875 | 0.0429875 | 0.056875 | 0.0700375 | 0.082925 | 0.1013375 | 0.107475 | 0.1283125 | 0.2027625 | 0.0177375 | 0.004975 | 3.00E-04 | 30.04076087 |
| Bexagliflozin | 0 | 7.50E-05 | 0.001 | 0.0055875 | 0.0030125 | 0.003375 | 0.003325 | 0.00295 | 0.002325 | 0.00265 | 0.0019 | 0.0017375 | 0.002025 | 0.0018625 | 0.0022125 | 0.00235 | 0.0029125 | 0.0037125 | 0.0045375 | 0.0064125 | 0.0103625 | 0.5614625 | 0.0304875 | 0.343725 | 7.951086957 |
| Canagliflozin_high_dosage | 0 | 2.50E-05 | 0.006775 | 0.1199125 | 0.18185 | 0.1871375 | 0.1792125 | 0.1051875 | 0.0665625 | 0.0419375 | 0.0276125 | 0.0197625 | 0.015825 | 0.0135 | 0.010625 | 0.008325 | 0.0064375 | 0.004425 | 0.00275 | 0.001625 | 0.0004625 | 3.75E-05 | 1.25E-05 | 0 | 73.2201087 |
| Canagliflozin_low_dosage | 0 | 0 | 0.0014125 | 0.0237125 | 0.05135 | 0.0945625 | 0.1258625 | 0.1367 | 0.1355125 | 0.093325 | 0.06725 | 0.0482125 | 0.0435625 | 0.040025 | 0.0372375 | 0.03315 | 0.0252875 | 0.0185125 | 0.0117375 | 0.008075 | 0.0038625 | 6.00E-04 | 5.00E-05 | 0 | 61.61858696 |
| Dapagliflozin_high_dosage | 0 | 0 | 2.50E-05 | 7.00E-04 | 0.0029 | 0.008925 | 0.0225125 | 0.043325 | 0.0707625 | 0.0956625 | 0.1148375 | 0.1075 | 0.110225 | 0.11565 | 0.1064125 | 0.081875 | 0.0621125 | 0.0325125 | 0.015825 | 0.00625 | 0.0017125 | 0.00025 | 2.50E-05 | 0 | 48.74402174 |
| Dapagliflozin_low_dosage | 0.1162375 | 0.357875 | 0.43995 | 0.0194625 | 0.0117 | 0.028 | 0.00455 | 0.0047125 | 0.002325 | 0.001875 | 0.0011125 | 0.0010875 | 0.001 | 0.00095 | 0.0008625 | 0.0009625 | 0.0013 | 0.0009875 | 9.00E-04 | 0.001875 | 0.0016125 | 4.00E-04 | 0.0002625 | 0 | 92.2238587 |
| Dapagliflozin_medium_dosage | 0.525825 | 0.2641 | 0.1909875 | 0.007375 | 0.0031375 | 0.0016875 | 0.0012875 | 0.0008625 | 0.000625 | 0.0005625 | 0.0002625 | 0.0003375 | 0.0002375 | 0.000225 | 0.0002125 | 2.00E-04 | 0.00025 | 0.0002625 | 6.00E-04 | 4.00E-04 | 0.0003875 | 0.000175 | 0 | 0 | 96.65483696 |
| Dulaglutide | 0 | 0 | 0 | 7.50E-05 | 0.000375 | 0.0010375 | 0.002175 | 0.0038 | 0.006 | 0.0073625 | 0.00975 | 0.0119375 | 0.017 | 0.0256625 | 0.0357375 | 0.051525 | 0.07575 | 0.1313875 | 0.154625 | 0.191925 | 0.2322 | 0.0331 | 0.0079375 | 0.0006375 | 23.45978261 |
| Efpeglenatide_high_dosage | 0 | 3.75E-05 | 0.001425 | 0.00255 | 0.0051375 | 0.0034375 | 0.0025125 | 0.0022 | 0.0028125 | 0.0030125 | 0.0021 | 0.00165 | 0.0013875 | 0.00215 | 0.0020875 | 0.0021375 | 0.0024375 | 0.0034625 | 0.00355 | 0.0039375 | 0.005675 | 0.245425 | 0.51885 | 0.182025 | 7.04923913 |
| Efpeglenatide_medium_dosage | 0 | 0 | 0 | 0.0002625 | 0.000225 | 0.0004375 | 9.00E-04 | 0.001125 | 0.0014 | 0.0014375 | 0.00165 | 0.001325 | 0.0012875 | 0.0012625 | 0.001725 | 0.0018875 | 0.0018375 | 0.002325 | 0.0028375 | 0.003425 | 0.0046125 | 0.0782125 | 0.4193875 | 0.4724375 | 3.612771739 |
| Empagliflozin_high_dosage | 0 | 0 | 0.005 | 0.0474 | 0.105 | 0.111725 | 0.124025 | 0.1081875 | 0.091625 | 0.0713125 | 0.0562 | 0.04335 | 0.0389625 | 0.0388125 | 0.0391 | 0.0379625 | 0.0286875 | 0.0207375 | 0.015875 | 0.01035 | 0.0047625 | 0.00075 | 0.000175 | 0 | 62.85353261 |
| Empagliflozin_low_dosage | 0 | 0 | 0 | 0 | 3.75E-05 | 3.00E-04 | 0.0006625 | 0.0017875 | 0.0040625 | 0.007575 | 0.0107375 | 0.0158625 | 0.024125 | 0.0383375 | 0.0629625 | 0.0919125 | 0.12635 | 0.1549875 | 0.168225 | 0.1742 | 0.0999125 | 0.0133875 | 0.0044875 | 8.75E-05 | 26.8561413 |
| Ertugliflozin_high_dosage | 0 | 0 | 0 | 6.25E-05 | 0.0013 | 0.0019125 | 0.004175 | 0.0081625 | 0.0163875 | 0.0189 | 0.02375 | 0.0239875 | 0.025475 | 0.0326 | 0.0515875 | 0.072925 | 0.0811875 | 0.1046875 | 0.13 | 0.1620875 | 0.208925 | 0.0257875 | 0.00575 | 0.00035 | 26.96418478 |
| Ertugliflozin_low_dosage | 0 | 2.50E-05 | 6.00E-04 | 0.0085 | 0.0179625 | 0.032225 | 0.0447 | 0.055825 | 0.064375 | 0.071225 | 0.0560375 | 0.0583625 | 0.0484125 | 0.05545 | 0.067825 | 0.073925 | 0.078225 | 0.073675 | 0.079025 | 0.0692375 | 0.0378125 | 0.0051 | 0.0014 | 7.50E-05 | 44.45565217 |
| Exenatide | 0 | 0 | 1.00E-04 | 0.001675 | 0.0053 | 0.0133 | 0.0239125 | 0.040825 | 0.056275 | 0.0702625 | 0.0725 | 0.073275 | 0.078625 | 0.091675 | 0.10925 | 0.100575 | 0.08275 | 0.0673875 | 0.0498875 | 0.0361875 | 0.023225 | 0.0023875 | 0.0005875 | 3.75E-05 | 43.94472826 |
| Inject_semaglutide_high_dosage | 0 | 0 | 3.75E-05 | 0.0002375 | 0.0019 | 0.0076625 | 0.0155875 | 0.04375 | 0.0497875 | 0.0646625 | 0.0725875 | 0.0759375 | 0.0854875 | 0.0930875 | 0.0973625 | 0.09985 | 0.1091375 | 0.072775 | 0.052925 | 0.0353125 | 0.0194125 | 0.0021 | 0.0003875 | 1.25E-05 | 42.90059783 |
| Inject_semaglutide_low_dosage | 0 | 0.00055 | 0.047125 | 0.2527125 | 0.21805 | 0.1444375 | 0.0925875 | 0.0685625 | 0.049075 | 0.0304125 | 0.02015 | 0.0154125 | 0.0124 | 0.01145 | 0.0100875 | 0.008975 | 0.0069 | 0.0045375 | 0.0035125 | 0.0019875 | 0.0009875 | 8.75E-05 | 0 | 0 | 76.50543478 |
| Inject_semaglutide_medium_dosage | 0 | 0.0011 | 0.0354625 | 0.4144 | 0.2276875 | 0.12355 | 0.070975 | 0.0423 | 0.025625 | 0.0166625 | 0.01155 | 0.0080125 | 0.006225 | 0.0047875 | 0.0038625 | 0.002775 | 0.0022 | 0.0014875 | 8.00E-04 | 0.000425 | 0.0001125 | 0 | 0 | 0 | 80.68641304 |
| Liraglutide | 0 | 0 | 6.25E-05 | 0.0013875 | 0.0060375 | 0.0198125 | 0.041675 | 0.0705125 | 0.101725 | 0.111875 | 0.1006375 | 0.1003 | 0.08335 | 0.08175 | 0.0793875 | 0.0672 | 0.0507625 | 0.0359 | 0.025625 | 0.0145625 | 0.006225 | 0.000875 | 0.0003125 | 2.50E-05 | 50.89293478 |

| | | | | | | | | | | | | | | | | | | | | | | | | | |
|---|---|---|---|---|---|---|---|---|---|---|---|---|---|---|---|---|---|---|---|---|---|---|---|---|---|
| Lixisenatide | 0 | 0.0003375 | 0.0043125 | 0.0778875 | 0.1198625 | 0.1458625 | 0.133975 | 0.1272625 | 0.0817 | 0.0566375 | 0.043975 | 0.0357125 | 0.0311125 | 0.029375 | 0.0271 | 0.0235125 | 0.019025 | 0.0151 | 0.0140375 | 0.0083625 | 0.0042375 | 0.000525 | 7.50E-05 | 1.25E-05 | 66.52309783 |
| Oral_semaglutide | 0 | 0 | 0.0008125 | 0.0117625 | 0.0254375 | 0.0471375 | 0.06285 | 0.0656 | 0.0654625 | 0.06125 | 0.0537625 | 0.0455875 | 0.0481875 | 0.052875 | 0.05655 | 0.0617875 | 0.0639375 | 0.067425 | 0.0806875 | 0.0578375 | 0.060625 | 0.0064375 | 0.00375 | 0.0002375 | 45.80440217 |
| Placebo_or_Control | 0 | 0 | 0 | 0 | 3.75E-05 | 7.50E-05 | 0.0013 | 0.006975 | 0.0295 | 0.0887375 | 0.1700125 | 0.2275375 | 0.2324875 | 0.1427375 | 0.0689 | 0.02415 | 0.0064125 | 0.000775 | 0.0002625 | 6.25E-05 | 3.75E-05 | 0 | 0 | 0 | 50.48021739 |
| Sotagliflozin | 0 | 0 | 0.0001625 | 0.0027125 | 0.0088375 | 0.01795 | 0.02905 | 0.04155 | 0.052175 | 0.05515 | 0.052575 | 0.0527125 | 0.0577125 | 0.0827875 | 0.0720375 | 0.082 | 0.083175 | 0.0816 | 0.0743 | 0.07715 | 0.070075 | 0.0051625 | 0.0010875 | 3.75E-05 | 40.51603261 |
| Tirzepatide | 0.3579375 | 0.375875 | 0.2647375 | 0.00085 | 0.000475 | 0.0001125 | 1.25E-05 | 0 | 0 | 0 | 0 | 0 | 0 | 0 | 0 | 0 | 0 | 0 | 0 | 0 | 0 | 0 | 0 | 0 | 96.04157609 |

# Table S6B: SUCRA (Surface under the cumulative ranking) of primary outcome: subgroup of lymphoma

| Treatment | Rank 1 | Rank 2 | Rank 3 | Rank 4 | Rank 5 | Rank 6 | Rank 7 | Rank 8 | Rank 9 | Rank 10 | Rank 11 | Rank 12 | Rank 13 | Rank 14 | Rank 15 | Rank 16 | Rank 17 | Rank 18 | Rank 19 | Rank 20 | Rank 21 | Rank 22 | SUCRA |
|---|---|---|---|---|---|---|---|---|---|---|---|---|---|---|---|---|---|---|---|---|---|---|---|
| Albiglutide | 0 | 0.006225 | 0.011925 | 0.01745 | 0.0231 | 0.0323375 | 0.0274125 | 0.027 | 0.0261125 | 0.0259 | 0.0289375 | 0.0313875 | 0.0378125 | 0.0427625 | 0.0501375 | 0.05715 | 0.0710125 | 0.11185 | 0.283675 | 0.0493875 | 0.0361 | 0.002325 | 32.43595238 |
| Bexagliflozin | 6.25E-05 | 0.00465 | 0.003675 | 0.0030375 | 0.0031 | 0.0027625 | 0.002625 | 0.00245 | 0.0019875 | 0.00205 | 0.0021625 | 0.0026 | 0.0026 | 0.0033 | 0.003175 | 0.0037625 | 0.0062125 | 0.0074875 | 0.016775 | 0.2868875 | 0.022925 | 0.6157125 | 6.153571429 |
| Canagliflozin_high_dosage | 0.000225 | 0.041025 | 0.0815625 | 0.105925 | 0.1146125 | 0.1089875 | 0.0931125 | 0.0723875 | 0.055725 | 0.050925 | 0.0485125 | 0.0447625 | 0.04225 | 0.038475 | 0.028925 | 0.0237125 | 0.02345 | 0.0149375 | 0.0088 | 0.0012625 | 0.0003875 | 3.75E-05 | 66.37994048 |
| Canagliflozin_low_dosage | 6.25E-05 | 0.0070125 | 0.0202 | 0.0347125 | 0.0495375 | 0.0569625 | 0.0598375 | 0.0580375 | 0.059925 | 0.055 | 0.0573625 | 0.0656375 | 0.0652125 | 0.06715 | 0.068725 | 0.0682625 | 0.0707875 | 0.0733875 | 0.0515875 | 0.0076875 | 0.002775 | 0.0001375 | 48.45410714 |
| Dapagliflozin_high_dosage | 1.25E-05 | 0.0008875 | 0.00325 | 0.007525 | 0.014 | 0.023425 | 0.030375 | 0.0387375 | 0.0498 | 0.0599875 | 0.073325 | 0.09155 | 0.1040125 | 0.112825 | 0.1108125 | 0.1074 | 0.0834375 | 0.056225 | 0.0263625 | 0.004775 | 0.00115 | 0.000125 | 42.40934524 |
| Dulaglutide | 5.00E-05 | 0.0010125 | 0.0028 | 0.0059875 | 0.009425 | 0.013975 | 0.0174625 | 0.0216375 | 0.0273 | 0.032725 | 0.0412625 | 0.0509875 | 0.0618125 | 0.079225 | 0.092225 | 0.1093875 | 0.129475 | 0.1453375 | 0.1262375 | 0.022075 | 0.0091 | 5.00E-04 | 33.30964286 |
| Efpeglenatide_high_dosage | 0.0009125 | 0.0039375 | 0.005325 | 0.00535 | 0.005125 | 0.0035875 | 0.003525 | 0.0032875 | 0.0029 | 0.003175 | 0.0033875 | 0.003525 | 0.00385 | 0.0054375 | 0.004825 | 0.0059875 | 0.0069 | 0.0090125 | 0.0182 | 0.2657125 | 0.4446 | 0.1914375 | 9.219761905 |
| Efpeglenatide_medium_dosage | 6.25E-05 | 0.004 | 0.00415 | 0.0034125 | 0.003425 | 0.00315 | 0.0033 | 0.002975 | 0.002925 | 0.0026875 | 0.0030125 | 0.003375 | 0.0039375 | 0.0041375 | 0.00465 | 0.005525 | 0.0070625 | 0.0100625 | 0.0201375 | 0.2735 | 0.4476 | 0.1869125 | 8.682559524 |
| Empagliflozin_high_dosage | 0.0068875 | 0.2075625 | 0.15505 | 0.1176125 | 0.096325 | 0.071025 | 0.055125 | 0.041025 | 0.034575 | 0.0303875 | 0.0287375 | 0.0268 | 0.0254375 | 0.0221 | 0.020025 | 0.017575 | 0.016125 | 0.0145125 | 0.0099125 | 0.0026125 | 0.000525 | 6.25E-05 | 75.03702381 |
| Empagliflozin_low_dosage | 0 | 0.0008375 | 0.003575 | 0.010675 | 0.0237125 | 0.039625 | 0.0538375 | 0.06105 | 0.0702125 | 0.0776375 | 0.086675 | 0.096825 | 0.1027625 | 0.0951125 | 0.0855 | 0.0719875 | 0.0582875 | 0.039425 | 0.018475 | 0.003025 | 0.00075 | 1.25E-05 | 47.47541667 |
| Ertugliflozin_high_dosage | 0 | 0.001175 | 0.00425 | 0.0099 | 0.01525 | 0.0247875 | 0.0315625 | 0.0392375 | 0.039475 | 0.0401 | 0.0459125 | 0.05485 | 0.0631 | 0.07025 | 0.0811875 | 0.0947625 | 0.108775 | 0.12505 | 0.11385 | 0.0244 | 0.0113625 | 0.0007625 | 36.76928571 |
| Ertugliflozin_low_dosage | 2.50E-05 | 0.014975 | 0.0281125 | 0.0404875 | 0.0508875 | 0.05815 | 0.0574 | 0.0563375 | 0.051425 | 0.0508875 | 0.058875 | 0.0606 | 0.0650125 | 0.0666625 | 0.0713875 | 0.0742 | 0.07315 | 0.0686 | 0.040675 | 0.0086625 | 0.0030375 | 0.00045 | 49.4610119 |
| Exenatide | 0.000175 | 0.025175 | 0.0537625 | 0.0837 | 0.1056 | 0.107825 | 0.1022375 | 0.085075 | 0.067225 | 0.0593875 | 0.0562125 | 0.051075 | 0.047375 | 0.0390625 | 0.0344875 | 0.0275 | 0.0235875 | 0.0174875 | 0.00985 | 0.002275 | 0.000825 | 1.00E-04 | 63.63827381 |
| Inject_semaglutide_high_dosage | 0 | 0.000375 | 0.0023625 | 0.00695 | 0.014425 | 0.0243 | 0.0311625 | 0.0364625 | 0.0421125 | 0.0515125 | 0.0633875 | 0.073475 | 0.0834125 | 0.0931 | 0.1027375 | 0.0971625 | 0.0976875 | 0.0910625 | 0.07005 | 0.0131625 | 0.0046125 | 0.0004875 | 39.42130952 |
| Inject_semaglutide_low_dosage | 0.000425 | 0.0765875 | 0.132575 | 0.152225 | 0.1205125 | 0.10225 | 0.0786875 | 0.06205 | 0.04475 | 0.0386625 | 0.03425 | 0.03215 | 0.0283875 | 0.0248 | 0.02065 | 0.018775 | 0.0139125 | 0.009875 | 0.0064625 | 0.0015 | 0.000475 | 3.75E-05 | 72.00160714 |
| Inject_semaglutide_medium_dosage | 0.0019875 | 0.3969 | 0.2205625 | 0.1234625 | 0.0773375 | 0.0496875 | 0.0336125 | 0.02315 | 0.01615 | 0.0128875 | 0.0108375 | 0.0093 | 0.00695 | 0.0047375 | 0.004525 | 0.0036625 | 0.0021875 | 0.0010625 | 0.0009125 | 3.75E-05 | 5.00E-05 | 0 | 86.0939881 |
| Liraglutide | 1.25E-05 | 0.0021875 | 0.008575 | 0.018225 | 0.030325 | 0.0428375 | 0.0574625 | 0.0569625 | 0.0642375 | 0.06825 | 0.076125 | 0.082 | 0.0862625 | 0.08475 | 0.082425 | 0.078875 | 0.0697125 | 0.0535 | 0.0299375 | 0.005475 | 0.00175 | 0.0001125 | 47.03071429 |
| Lixisenatide | 0.0019375 | 0.15985 | 0.1822125 | 0.148125 | 0.1108125 | 0.0806375 | 0.06025 | 0.045575 | 0.03615 | 0.029325 | 0.026375 | 0.0258125 | 0.0206375 | 0.017975 | 0.0152 | 0.013475 | 0.0108625 | 0.00825 | 0.004925 | 0.0011875 | 4.00E-04 | 2.50E-05 | 76.54065476 |
| Oral_semaglutide | 0.00045 | 0.0316 | 0.057775 | 0.078375 | 0.0875875 | 0.07815 | 0.071525 | 0.0623125 | 0.05545 | 0.055325 | 0.0504125 | 0.0529375 | 0.04915 | 0.0486625 | 0.0458 | 0.0442625 | 0.0426 | 0.0424125 | 0.0352625 | 0.0065625 | 0.0031375 | 0.00025 | 58.21785714 |

| | | | | | | | | | | | | | | | | | | | | | | | |
|---|---|---|---|---|---|---|---|---|---|---|---|---|---|---|---|---|---|---|---|---|---|---|---|
| Placebo_or_Control | 0 | 1.25E-05 | 0.0001375 | 0.00125 | 0.008775 | 0.034 | 0.0861375 | 0.1593 | 0.2077375 | 0.2076375 | 0.1535875 | 0.0851875 | 0.039125 | 0.0130875 | 0.003425 | 0.000475 | 0.000125 | 0 | 0 | 0 | 0 | 0 | 59.32690476 |
| Sotagliflozin | 2.50E-05 | 0.0035375 | 0.0161875 | 0.02515 | 0.035875 | 0.0414625 | 0.0433125 | 0.0449375 | 0.043825 | 0.04555 | 0.050625 | 0.0551625 | 0.0609 | 0.0663875 | 0.069175 | 0.0761 | 0.08465 | 0.1004625 | 0.1079125 | 0.0198125 | 0.0084375 | 0.0005125 | 42.02559524 |
| Tirzepatide | 0.9866875 | 0.010475 | 0.001975 | 0.0004625 | 0.00025 | 7.50E-05 | 3.75E-05 | 1.25E-05 | 0 | 0 | 2.50E-05 | 0 | 0 | 0 | 0 | 0 | 0 | 0 | 0 | 0 | 0 | 0 | 99.91547619 |

# Table S6C: SUCRA (Surface under the cumulative ranking) of primary outcome: subgroup of leukemia

| Treatment | Rank 1 | Rank 2 | Rank 3 | Rank 4 | Rank 5 | Rank 6 | Rank 7 | Rank 8 | Rank 9 | Rank 10 | Rank 11 | Rank 12 | Rank 13 | Rank 14 | Rank 15 | Rank 16 | Rank 17 | Rank 18 | Rank 19 | Rank 20 | Rank 21 | Rank 22 | Rank 23 | SUCRA |
|---|---|---|---|---|---|---|---|---|---|---|---|---|---|---|---|---|---|---|---|---|---|---|---|---|
| Albiglutide | 0 | 1.25E-05 | 1.25E-05 | 0.000175 | 0.001575 | 0.0068875 | 0.0181625 | 0.0313125 | 0.042775 | 0.05235 | 0.0593375 | 0.06085 | 0.063275 | 0.0677375 | 0.0754375 | 0.089325 | 0.09725 | 0.1020875 | 0.091375 | 0.0701 | 0.0526375 | 0.0157125 | 0.0016125 | 35.60113636 |
| Canagliflozin_high_dosage | 0 | 0.0001625 | 0.0023 | 0.017675 | 0.0768625 | 0.2068875 | 0.194025 | 0.1342125 | 0.098575 | 0.0665875 | 0.0464625 | 0.035925 | 0.026675 | 0.021175 | 0.0195125 | 0.0168625 | 0.0141625 | 0.0099875 | 0.005975 | 0.0037375 | 0.0016875 | 0.000525 | 2.50E-05 | 65.94198864 |
| Canagliflozin_low_dosage | 0 | 0 | 0.000225 | 0.0039125 | 0.0220875 | 0.0814375 | 0.132625 | 0.140425 | 0.1305125 | 0.1063875 | 0.084425 | 0.0643375 | 0.0516125 | 0.0423875 | 0.0360375 | 0.0308 | 0.0264625 | 0.0200625 | 0.0141875 | 0.008275 | 0.00315 | 0.0006125 | 3.75E-05 | 58.04244318 |
| Dapagliflozin_high_dosage | 0 | 0 | 2.50E-05 | 0.0003125 | 0.0028375 | 0.0136125 | 0.0349 | 0.067175 | 0.0957125 | 0.120725 | 0.1296625 | 0.1239625 | 0.1091875 | 0.0931375 | 0.0762375 | 0.0582625 | 0.0383 | 0.02145 | 0.0096 | 0.0038375 | 0.000875 | 0.0001875 | 0 | 50.09380682 |
| Dapagliflozin_low_dosage | 0.0561 | 0.155475 | 0.229925 | 0.221025 | 0.20305 | 0.068575 | 0.019775 | 0.0123875 | 0.00805 | 0.0049375 | 0.0041875 | 0.00285 | 0.002325 | 0.0021375 | 0.001975 | 0.0018 | 0.0016625 | 0.00135 | 0.0012125 | 0.000675 | 3.00E-04 | 0.0001875 | 3.75E-05 | 86.24454545 |
| Dapagliflozin_medium_dosage | 0.07885 | 0.163175 | 0.199125 | 0.213675 | 0.2068875 | 0.0667125 | 0.022725 | 0.012725 | 0.0086125 | 0.0063625 | 0.0039875 | 0.0033625 | 0.00245 | 0.002225 | 0.00205 | 0.001725 | 0.0017 | 0.0014875 | 0.0009625 | 7.00E-04 | 0.0003375 | 0.0001625 | 0 | 86.371875 |
| Dulaglutide | 0 | 0 | 7.50E-05 | 0.00065 | 0.003775 | 0.0137375 | 0.024725 | 0.0337375 | 0.0405 | 0.048175 | 0.0504875 | 0.051175 | 0.0508125 | 0.0557875 | 0.0630875 | 0.07695 | 0.08895 | 0.0975125 | 0.09645 | 0.09095 | 0.078875 | 0.0294125 | 0.004175 | 34.08948864 |
| Efpeglenatide_high_dosage | 0.0235625 | 0.0315 | 0.0588375 | 0.062675 | 0.05885 | 0.04375 | 0.0129875 | 0.007975 | 0.0058 | 0.004725 | 0.00395 | 0.003525 | 0.0032875 | 0.0030375 | 0.0036875 | 0.004025 | 0.0050375 | 0.005725 | 0.006925 | 0.0094875 | 0.0130375 | 0.5962625 | 0.03135 | 30.73619318 |
| Efpeglenatide_medium_dosage | 0 | 0 | 0 | 0 | 0.00015 | 0.000425 | 0.001325 | 0.00115 | 0.00145 | 0.00155 | 0.00145 | 0.0013625 | 0.0013125 | 0.0013375 | 0.001625 | 0.002075 | 0.0025875 | 0.0034875 | 0.0043625 | 0.0061125 | 0.0088375 | 0.043525 | 0.915875 | 1.381136364 |
| Empagliflozin_high_dosage | 0 | 0 | 2.50E-05 | 0.00055 | 0.0052125 | 0.0240625 | 0.0492625 | 0.0663125 | 0.067825 | 0.0694125 | 0.0674125 | 0.066025 | 0.0623125 | 0.062275 | 0.068675 | 0.0743625 | 0.0796375 | 0.0754125 | 0.07105 | 0.0557625 | 0.0269125 | 0.006775 | 0.000725 | 42.29261364 |
| Empagliflozin_low_dosage | 0 | 0 | 0 | 0 | 0 | 0 | 1.25E-05 | 0.0001875 | 0.00035 | 0.0008125 | 0.00145 | 0.0026 | 0.00475 | 0.0081125 | 0.0153125 | 0.0285125 | 0.05165 | 0.0928875 | 0.1553125 | 0.20255 | 0.3016125 | 0.1181125 | 0.015775 | 14.68931818 |
| Ertugliflozin_high_dosage | 0 | 0 | 5.00E-05 | 5.00E-04 | 0.0024375 | 0.008525 | 0.0143625 | 0.019475 | 0.024075 | 0.02745 | 0.0304625 | 0.0318625 | 0.0333 | 0.0352625 | 0.03965 | 0.0518 | 0.0665 | 0.08145 | 0.1063375 | 0.1522 | 0.1891875 | 0.071525 | 0.0135875 | 25.45193182 |
| Ertugliflozin_low_dosage | 0 | 1.25E-05 | 0.0001875 | 0.001 | 0.0055875 | 0.01785 | 0.0293375 | 0.036925 | 0.0407 | 0.04115 | 0.0407 | 0.041175 | 0.0398 | 0.042825 | 0.0466625 | 0.056725 | 0.07005 | 0.0845375 | 0.0988625 | 0.1316375 | 0.122275 | 0.044975 | 0.007025 | 31.69573864 |
| Exenatide | 0 | 0 | 5.00E-05 | 0.0006125 | 0.00405 | 0.013925 | 0.0242125 | 0.03535 | 0.04425 | 0.05345 | 0.056725 | 0.060125 | 0.0629125 | 0.06495 | 0.0792875 | 0.0905375 | 0.097675 | 0.0950875 | 0.0841125 | 0.064875 | 0.0483 | 0.0169375 | 0.002575 | 36.74625 |
| Inject_semaglutide_high_dosage | 0 | 0 | 0 | 7.50E-05 | 0.0016375 | 0.00815 | 0.021475 | 0.0428 | 0.0604125 | 0.0762625 | 0.0901125 | 0.0863 | 0.082075 | 0.0837 | 0.08575 | 0.086575 | 0.0831 | 0.07255 | 0.0551875 | 0.036575 | 0.0207875 | 0.0061375 | 0.0003375 | 41.49318182 |
| Inject_semaglutide_low_dosage | 0.32785 | 0.2709125 | 0.1779375 | 0.113925 | 0.071425 | 0.0176625 | 0.0056625 | 0.0036625 | 0.0022375 | 0.002225 | 0.0011625 | 0.0011 | 0.0011125 | 0.00085 | 0.000425 | 0.0004625 | 0.0002375 | 0.0004125 | 0.0004125 | 0.00015 | 0.00015 | 2.50E-05 | 0 | 93.06482955 |
| Inject_semaglutide_medium_dosage | 0 | 1.00E-04 | 0.00155 | 0.012025 | 0.0533125 | 0.148775 | 0.15775 | 0.1178 | 0.0985375 | 0.0766875 | 0.0588875 | 0.0463 | 0.0396625 | 0.034175 | 0.03185 | 0.0287375 | 0.028525 | 0.023125 | 0.0196875 | 0.0127 | 0.0075125 | 0.0020625 | 0.0002375 | 60.06664773 |
| Liraglutide | 0 | 0 | 0 | 5.00E-05 | 9.00E-04 | 0.0064125 | 0.017875 | 0.035025 | 0.0512375 | 0.06815 | 0.07545 | 0.0722625 | 0.0714 | 0.0692375 | 0.0785625 | 0.0840875 | 0.0887125 | 0.08355 | 0.0736875 | 0.0604625 | 0.044875 | 0.0155625 | 0.0025 | 37.84920455 |
| Lixisenatide | 0 | 5.00E-05 | 0.0008875 | 0.0077125 | 0.0452 | 0.1341875 | 0.126325 | 0.1123875 | 0.0889875 | 0.0712875 | 0.0584125 | 0.0490125 | 0.040925 | 0.038625 | 0.036875 | 0.0395375 | 0.03665 | 0.0346875 | 0.028425 | 0.0235875 | 0.019175 | 0.0063625 | 7.00E-04 | 56.04971591 |

| | | | | | | | | | | | | | | | | | | | | | | | | |
|---|---|---|---|---|---|---|---|---|---|---|---|---|---|---|---|---|---|---|---|---|---|---|---|---|
| Oral_semaglutide | 0.1734875 | 0.1751375 | 0.2046875 | 0.185575 | 0.1242375 | 0.046775 | 0.0196625 | 0.0129 | 0.008825 | 0.0066875 | 0.005175 | 0.0046375 | 0.0040125 | 0.0035 | 0.003625 | 0.00355 | 0.0033375 | 0.0036875 | 0.00305 | 0.003125 | 0.002925 | 0.001225 | 0.000175 | 87.13647727 |
| Placebo_or_Control | 0 | 0 | 0 | 0 | 0 | 2.50E-05 | 1.00E-04 | 0.001225 | 0.0058125 | 0.0230375 | 0.0657 | 0.1337625 | 0.1958 | 0.21435 | 0.1777875 | 0.109775 | 0.0497 | 0.0177625 | 0.0045125 | 0.000575 | 6.25E-05 | 1.25E-05 | 0 | 41.57204545 |
| Sotagliflozin | 0 | 2.50E-05 | 0.0002125 | 0.0020625 | 0.011475 | 0.0412625 | 0.0606375 | 0.067925 | 0.069625 | 0.068025 | 0.061625 | 0.0551625 | 0.04905 | 0.051575 | 0.0540875 | 0.0618875 | 0.066325 | 0.0699 | 0.0669875 | 0.0607 | 0.055275 | 0.0231 | 0.003075 | 42.26431818 |
| Tirzepatide | 0.34015 | 0.2034375 | 0.1238875 | 0.1558125 | 0.09845 | 0.0303625 | 0.012075 | 0.006925 | 0.0051375 | 0.0035625 | 0.002775 | 0.002325 | 0.00195 | 0.0016 | 0.0018 | 0.001625 | 0.0017875 | 0.0018 | 0.001325 | 0.001225 | 0.0012125 | 6.00E-04 | 0.000175 | 91.12511364 |

# Table S6D: SUCRA (Surface under the cumulative ranking) of primary outcome: subgroup of myeloma

| Treatment | Rank 1 | Rank 2 | Rank 3 | Rank 4 | Rank 5 | Rank 6 | Rank 7 | Rank 8 | Rank 9 | Rank 10 | Rank 11 | Rank 12 | Rank 13 | Rank 14 | Rank 15 | Rank 16 | Rank 17 | Rank 18 | Rank 19 | SUCRA |
|---|---|---|---|---|---|---|---|---|---|---|---|---|---|---|---|---|---|---|---|---|
| Albiglutide | 0 | 2.50E-05 | 3.75E-05 | 3.75E-05 | 0.0005875 | 0.0017125 | 0.0042 | 0.0046125 | 0.004125 | 0.003425 | 0.002875 | 0.0027375 | 0.0033375 | 0.0057375 | 0.0103 | 0.0173625 | 0.274175 | 0.291925 | 0.3727875 | 6.831805556 |
| Canagliflozin_high_dosage | 0.1294 | 0.1708625 | 0.187 | 0.1610875 | 0.1573125 | 0.094275 | 0.0235375 | 0.014175 | 0.009825 | 0.0081375 | 0.007175 | 0.0071125 | 0.007225 | 0.0069125 | 0.0056 | 0.007575 | 0.0018625 | 0.0009125 | 1.25E-05 | 82.86027778 |
| Canagliflozin_low_dosage | 0.11945 | 0.1967625 | 0.17125 | 0.1635375 | 0.154175 | 0.105425 | 0.0222 | 0.0124625 | 0.0085625 | 0.00715 | 0.0061 | 0.0056875 | 0.007 | 0.00615 | 0.006275 | 0.0065 | 0.000975 | 0.000325 | 1.25E-05 | 83.21555556 |
| Dapagliflozin_high_dosage | 0 | 0 | 0.000125 | 0.00065 | 0.0069375 | 0.04245 | 0.1365875 | 0.1993875 | 0.1773125 | 0.1391625 | 0.098075 | 0.0778 | 0.0581875 | 0.0380625 | 0.019175 | 0.0055625 | 0.000525 | 0 | 0 | 52.67611111 |
| Dulaglutide | 0 | 0 | 2.50E-05 | 6.25E-05 | 0.00055 | 0.001725 | 0.0082125 | 0.0130625 | 0.01755 | 0.024075 | 0.0307125 | 0.0450625 | 0.0771625 | 0.1303 | 0.220675 | 0.361225 | 0.06365 | 0.0058 | 0.00015 | 24.67201389 |
| Empagliflozin_high_dosage | 0 | 5.00E-05 | 0.0009875 | 0.0059125 | 0.0251 | 0.0881875 | 0.1546875 | 0.1124 | 0.0925875 | 0.0767125 | 0.066425 | 0.0686125 | 0.0793375 | 0.086425 | 0.077375 | 0.0578375 | 0.006575 | 0.0007875 | 0 | 48.49388889 |
| Empagliflozin_low_dosage | 0 | 0 | 0 | 0.0003875 | 0.002025 | 0.009175 | 0.0353625 | 0.0618625 | 0.0932125 | 0.1071125 | 0.11305 | 0.128375 | 0.146325 | 0.1406625 | 0.1088125 | 0.0470125 | 0.006275 | 0.0003375 | 1.25E-05 | 39.59930556 |
| Ertugliflozin_high_dosage | 0 | 0 | 5.00E-04 | 0.003525 | 0.0166375 | 0.0586875 | 0.1172 | 0.08265 | 0.0734875 | 0.0619875 | 0.0562125 | 0.0606125 | 0.0767125 | 0.095325 | 0.1299375 | 0.1426 | 0.02155 | 0.0022125 | 0.0001625 | 41.49333333 |
| Ertugliflozin_low_dosage | 0.2437125 | 0.2029875 | 0.177725 | 0.1496 | 0.118675 | 0.0723125 | 0.012025 | 0.005775 | 0.0035875 | 0.0023875 | 0.0019125 | 0.0018125 | 0.001925 | 0.0017 | 0.001775 | 0.0016 | 0.0003875 | 1.00E-04 | 0 | 88.07069444 |
| Exenatide | 0 | 0 | 7.50E-05 | 0.00025 | 0.0025125 | 0.0136375 | 0.0346625 | 0.0592375 | 0.075125 | 0.0901125 | 0.0993 | 0.1095875 | 0.1368125 | 0.15625 | 0.1358625 | 0.075925 | 0.00995 | 0.000625 | 7.50E-05 | 37.79236111 |
| Inject_semaglutide_high_dosage | 0 | 0 | 0.0002125 | 0.00135 | 0.007225 | 0.033825 | 0.0931875 | 0.1152875 | 0.124275 | 0.1158375 | 0.10185 | 0.0993 | 0.0988875 | 0.09775 | 0.0708625 | 0.0351125 | 0.0045875 | 0.0004375 | 1.25E-05 | 45.70333333 |
| Inject_semaglutide_low_dosage | 0.107875 | 0.0933625 | 0.1646 | 0.197575 | 0.18105 | 0.134725 | 0.0276875 | 0.0158875 | 0.0117875 | 0.0090625 | 0.0081625 | 0.00775 | 0.0095 | 0.01055 | 0.0090125 | 0.0089375 | 0.0021875 | 0.0002875 | 0 | 80.08631944 |
| Inject_semaglutide_medium_dosage | 0.2223375 | 0.170375 | 0.133775 | 0.1502375 | 0.145275 | 0.0965875 | 0.016925 | 0.009825 | 0.0071625 | 0.0064625 | 0.005325 | 0.005075 | 0.00545 | 0.0066375 | 0.007825 | 0.00875 | 0.00185 | 0.000125 | 0 | 84.60138889 |
| Liraglutide | 0 | 0.0001375 | 9.00E-04 | 0.0057 | 0.02415 | 0.091725 | 0.214225 | 0.183725 | 0.1420875 | 0.099575 | 0.070975 | 0.05635 | 0.0485125 | 0.0338375 | 0.019775 | 0.0075 | 8.00E-04 | 2.50E-05 | 0 | 55.92902778 |
| Lixisenatide | 0 | 3.75E-05 | 2.50E-05 | 6.25E-05 | 0.0006625 | 0.0020125 | 0.0024875 | 0.003325 | 0.0032125 | 0.0029625 | 0.0025875 | 0.003125 | 0.0036625 | 0.005375 | 0.0105625 | 0.0213875 | 0.2478875 | 0.347125 | 0.3435 | 6.685347222 |
| Oral_semaglutide | 0 | 0 | 0 | 2.50E-05 | 0.0002375 | 0.000475 | 0.001325 | 0.0027125 | 0.002175 | 0.00175 | 0.0015625 | 0.0022375 | 0.0031625 | 0.00555 | 0.0079 | 0.0163375 | 0.3252 | 0.3460875 | 0.2832625 | 6.917222222 |
| Placebo_or_Control | 0 | 0 | 0 | 0 | 1.25E-05 | 0.0005875 | 0.004125 | 0.0237 | 0.077775 | 0.171975 | 0.2586 | 0.247725 | 0.145575 | 0.05465 | 0.01365 | 0.0015125 | 6.25E-05 | 5.00E-05 | 0 | 42.51347222 |
| Sotagliflozin | 0 | 0 | 8.75E-05 | 0.0014625 | 0.005975 | 0.0257625 | 0.0702875 | 0.0706375 | 0.07 | 0.0678 | 0.06575 | 0.0678375 | 0.0882375 | 0.1152 | 0.1421125 | 0.1751875 | 0.0308125 | 0.0028375 | 1.25E-05 | 36.87402778 |
| Tirzepatide | 0.177225 | 0.1654 | 0.162675 | 0.1585375 | 0.1509 | 0.1267125 | 0.021075 | 0.009275 | 0.00615 | 0.0043125 | 0.00335 | 0.0032 | 0.0029875 | 0.002925 | 0.0025125 | 0.002075 | 0.0006875 | 0 | 0 | 84.98451389 |

# Table S6E: SUCRA (Surface under the cumulative ranking) of safety profile: drop-out rate

| Treatment | Rank 1 | Rank 2 | Rank 3 | Rank 4 | Rank 5 | Rank 6 | Rank 7 | Rank 8 | Rank 9 | Rank 10 | Rank 11 | Rank 12 | Rank 13 | Rank 14 | Rank 15 | Rank 16 | Rank 17 | Rank 18 | Rank 19 | Rank 20 | Rank 21 | Rank 22 | Rank 23 | Rank 24 | SUCRA |
|---|---|---|---|---|---|---|---|---|---|---|---|---|---|---|---|---|---|---|---|---|---|---|---|---|---|
| Albiglutide | 0.000125 | 0.0009375 | 0.002425 | 0.0054125 | 0.00955 | 0.0157 | 0.02195 | 0.029425 | 0.036975 | 0.046375 | 0.0544625 | 0.0616 | 0.0677375 | 0.075675 | 0.0775375 | 0.0779125 | 0.0749125 | 0.070525 | 0.06665 | 0.06345 | 0.0575875 | 0.0475 | 0.029375 | 0.0062 | 38.24559783 |
| Bexagliflozin | 0.04165 | 0.0378875 | 0.035375 | 0.0346875 | 0.0338125 | 0.034775 | 0.035225 | 0.0330125 | 0.032675 | 0.032375 | 0.03275 | 0.033175 | 0.0315625 | 0.031975 | 0.033825 | 0.0330125 | 0.03395 | 0.03315 | 0.036 | 0.0422 | 0.0537 | 0.0688625 | 0.1055375 | 0.078825 | 42.67788043 |
| Canagliflozin_high_dosage | 0.033175 | 0.0528375 | 0.0635375 | 0.0697125 | 0.0713125 | 0.0718875 | 0.0702 | 0.0676125 | 0.06075 | 0.05735 | 0.0524875 | 0.0474375 | 0.044 | 0.0403625 | 0.034775 | 0.0301 | 0.0270875 | 0.0233875 | 0.019975 | 0.0189625 | 0.016875 | 0.013875 | 0.00945 | 0.00285 | 63.38576087 |
| Canagliflozin_low_dosage | 0.008675 | 0.0203125 | 0.029825 | 0.0394625 | 0.04905 | 0.0566875 | 0.05995 | 0.0624625 | 0.064225 | 0.0639875 | 0.062 | 0.0597125 | 0.0569375 | 0.054675 | 0.050575 | 0.0457125 | 0.0412 | 0.0369375 | 0.032725 | 0.0312 | 0.0285125 | 0.023675 | 0.017 | 0.0045 | 53.72554348 |
| Dapagliflozin_high_dosage | 0.0025125 | 0.007625 | 0.0163875 | 0.025775 | 0.037625 | 0.0483 | 0.0562625 | 0.0617 | 0.068525 | 0.071775 | 0.0721375 | 0.070175 | 0.0698625 | 0.0654875 | 0.061 | 0.0564375 | 0.04625 | 0.04045 | 0.0347 | 0.0309 | 0.025375 | 0.0187375 | 0.010025 | 0.001975 | 51.46418478 |
| Dapagliflozin_low_dosage | 0.0236625 | 0.0335 | 0.03965 | 0.042675 | 0.046475 | 0.04825 | 0.0496 | 0.0488125 | 0.048775 | 0.0484375 | 0.04645 | 0.0477125 | 0.0462 | 0.045325 | 0.044 | 0.0414125 | 0.038325 | 0.0369875 | 0.037875 | 0.0400375 | 0.0434625 | 0.045825 | 0.0403625 | 0.0161875 | 50.95711957 |
| Dapagliflozin_medium_dosage | 0.0217 | 0.0306 | 0.0378 | 0.041425 | 0.0457375 | 0.0462875 | 0.0485125 | 0.0474625 | 0.0488625 | 0.0492125 | 0.0484 | 0.04815 | 0.046375 | 0.0449875 | 0.0438375 | 0.0413625 | 0.0394875 | 0.0384625 | 0.03915 | 0.0399625 | 0.0446375 | 0.0454125 | 0.0443875 | 0.0177875 | 50.11809783 |
| Dulaglutide | 0.0009125 | 0.0024875 | 0.0050625 | 0.007825 | 0.010325 | 0.0139875 | 0.0182625 | 0.0207 | 0.0260125 | 0.029375 | 0.0335625 | 0.0380125 | 0.04225 | 0.0468625 | 0.050425 | 0.0531375 | 0.056875 | 0.057625 | 0.064675 | 0.0771375 | 0.0912125 | 0.1028125 | 0.1081375 | 0.042325 | 29.98929348 |
| Efpeglenatide_high_dosage | 0.00865 | 0.0119875 | 0.0145375 | 0.0166375 | 0.0207125 | 0.0216625 | 0.024425 | 0.026775 | 0.0287375 | 0.0298875 | 0.032375 | 0.0342375 | 0.03485 | 0.0382375 | 0.0385875 | 0.041225 | 0.0424875 | 0.044475 | 0.0488 | 0.057575 | 0.0769625 | 0.10825 | 0.1265875 | 0.0713375 | 32.45375 |
| Efpeglenatide_medium_dosage | 0.009 | 0.0140875 | 0.016925 | 0.01935 | 0.0223625 | 0.025025 | 0.0268 | 0.0293 | 0.029875 | 0.0314125 | 0.033225 | 0.0345625 | 0.0366875 | 0.0368375 | 0.0412625 | 0.04205 | 0.042825 | 0.0441375 | 0.046925 | 0.057825 | 0.07595 | 0.104275 | 0.11555 | 0.06375 | 34.15581522 |
| Empagliflozin_high_dosage | 0.0089875 | 0.024775 | 0.0432125 | 0.061375 | 0.0765625 | 0.087025 | 0.0908 | 0.0908625 | 0.0858125 | 0.07795 | 0.0710875 | 0.0609 | 0.0535 | 0.04415 | 0.0351 | 0.027125 | 0.019475 | 0.0146375 | 0.0102875 | 0.0068375 | 0.0051 | 0.0029 | 0.0013 | 0.0002375 | 64.4648913 |
| Empagliflozin_low_dosage | 1.25E-05 | 0.0003625 | 0.0010875 | 0.0029125 | 0.0062875 | 0.0108125 | 0.0175125 | 0.0284625 | 0.0395125 | 0.052275 | 0.0666875 | 0.077375 | 0.08725 | 0.0916875 | 0.0944625 | 0.0903375 | 0.0809375 | 0.0688375 | 0.0601875 | 0.0472125 | 0.0377125 | 0.02455 | 0.011525 | 0.002 | 40.73543478 |
| Ertugliflozin_high_dosage | 0.0013875 | 0.0038875 | 0.006975 | 0.0112625 | 0.0156 | 0.0206 | 0.0247 | 0.0300625 | 0.0348625 | 0.0384 | 0.0433 | 0.047175 | 0.052625 | 0.055225 | 0.0574875 | 0.060225 | 0.0593875 | 0.0581875 | 0.0622125 | 0.068425 | 0.076525 | 0.0799625 | 0.0686875 | 0.0228375 | 35.58652174 |
| Ertugliflozin_low_dosage | 0.0068 | 0.014425 | 0.0222125 | 0.03015 | 0.0382125 | 0.0447375 | 0.0492625 | 0.0527125 | 0.056525 | 0.057925 | 0.057975 | 0.0596375 | 0.0595 | 0.057975 | 0.0542125 | 0.0532 | 0.0497375 | 0.0449125 | 0.0424 | 0.0421 | 0.041225 | 0.034325 | 0.0233 | 0.0065375 | 48.8911413 |
| Exenatide | 0.0105 | 0.0271125 | 0.0368125 | 0.0424625 | 0.0486625 | 0.05045 | 0.0523125 | 0.0539875 | 0.054175 | 0.0523125 | 0.0533875 | 0.05255 | 0.0513 | 0.048975 | 0.0480875 | 0.044825 | 0.041 | 0.0395625 | 0.0355625 | 0.038175 | 0.0392875 | 0.03705 | 0.030575 | 0.010875 | 51.47211957 |
| Inject_semaglutide_high_dosage | 0.016825 | 0.039075 | 0.061425 | 0.0788875 | 0.0892625 | 0.093875 | 0.0892625 | 0.0853375 | 0.0756875 | 0.068175 | 0.0594375 | 0.04975 | 0.0437875 | 0.0363125 | 0.0295875 | 0.0238625 | 0.018075 | 0.0135875 | 0.0096125 | 0.0069 | 0.0053125 | 0.0036375 | 0.001875 | 0.00045 | 67.1875 |
| Inject_semaglutide_low_dosage | 0.2052625 | 0.1186875 | 0.092275 | 0.07855 | 0.0657 | 0.05505 | 0.0471125 | 0.0414875 | 0.0366125 | 0.0312375 | 0.02755 | 0.0264375 | 0.0230875 | 0.0205 | 0.0194375 | 0.017075 | 0.014575 | 0.0140375 | 0.01275 | 0.0120375 | 0.013275 | 0.0125 | 0.0109625 | 0.0038 | 75.44 |
| Inject_semaglutide_medium_dosage | 0.0539 | 0.0888875 | 0.109075 | 0.109225 | 0.0995625 | 0.0844625 | 0.0737875 | 0.0630875 | 0.05355 | 0.0457625 | 0.0395375 | 0.0338125 | 0.029325 | 0.02445 | 0.0213 | 0.0170875 | 0.0132875 | 0.01065 | 0.009125 | 0.007175 | 0.005975 | 0.003875 | 0.0024875 | 0.0006125 | 73.32451087 |
| Liraglutide | 0.4097 | 0.2489625 | 0.1394 | 0.0826875 | 0.04815 | 0.0294 | 0.0160375 | 0.009675 | 0.006075 | 0.003975 | 0.002425 | 0.0013375 | 0.0009375 | 0.0005125 | 0.0002875 | 0.000275 | 0.0001125 | 2.50E-05 | 2.50E-05 | 0 | 0 | 0 | 0 | 0 | 93.77630435 |

| | | | | | | | | | | | | | | | | | | | | | | | | | |
|---|---|---|---|---|---|---|---|---|---|---|---|---|---|---|---|---|---|---|---|---|---|---|---|---|---|
| Lixisenatide | 0.0203125 | 0.0333 | 0.04025 | 0.043675 | 0.045725 | 0.0476 | 0.0489375 | 0.04805 | 0.0474375 | 0.0466625 | 0.0470875 | 0.0457 | 0.044625 | 0.0465625 | 0.0441375 | 0.0416875 | 0.039625 | 0.038625 | 0.0370875 | 0.040825 | 0.0432625 | 0.0457625 | 0.0446875 | 0.018375 | 50.35358696 |
| Oral_semaglutide | 0.0004625 | 0.0005375 | 0.0009125 | 0.00115 | 0.00155 | 0.0019375 | 0.0029 | 0.0036375 | 0.0043 | 0.004975 | 0.004975 | 0.006475 | 0.0069125 | 0.007825 | 0.009375 | 0.011125 | 0.0122875 | 0.0148625 | 0.01835 | 0.0264375 | 0.0390375 | 0.0664375 | 0.13825 | 0.6152875 | 6.963315217 |
| Placebo_or_Control | 0 | 0 | 0 | 0 | 0 | 0 | 0 | 0 | 1.25E-05 | 5.00E-05 | 0.0003375 | 0.001625 | 0.0053 | 0.0155125 | 0.0391125 | 0.079075 | 0.137275 | 0.1856875 | 0.2040625 | 0.170425 | 0.1067375 | 0.0437 | 0.0106 | 0.0004875 | 23.55690217 |
| Sotagliflozin | 0.00035 | 0.0013875 | 0.00355 | 0.006575 | 0.0101 | 0.0148375 | 0.0208625 | 0.0266625 | 0.033075 | 0.0397625 | 0.0452375 | 0.052575 | 0.0586125 | 0.0651 | 0.06805 | 0.07 | 0.0695125 | 0.0692875 | 0.070325 | 0.073875 | 0.072175 | 0.0659875 | 0.0493375 | 0.0127625 | 35.53092391 |
| Tirzepatide | 0.1154375 | 0.1863375 | 0.1812875 | 0.148125 | 0.1076625 | 0.07665 | 0.055325 | 0.0387125 | 0.02695 | 0.02035 | 0.013125 | 0.009875 | 0.006775 | 0.0047875 | 0.0035375 | 0.0017375 | 0.0013125 | 0.0009625 | 0.0005375 | 0.000325 | 1.00E-04 | 8.75E-05 | 0 | 0 | 85.54380435 |

## Table S7A: inconsistency within the network meta-analysis of primary outcome: overall hematologic malignancy

| Comparison | No.Studies | NMA | Direct | Indirect | Difference | Diff_95CI_lower | Diff_95CI_upper | *p* value |
|---|---|---|---|---|---|---|---|---|
| Albiglutide:Bexagliflozin | 0 | 0.062872591 | NA | 0.062872591 | NA | NA | NA | NA |
| Albiglutide:Canagliflozin_high_dosage | 0 | 1.269240461 | NA | 1.269240461 | NA | NA | NA | NA |
| Albiglutide:Canagliflozin_low_dosage | 0 | 0.829915896 | NA | 0.829915896 | NA | NA | NA | NA |
| Albiglutide:Dapagliflozin_high_dosage | 0 | 0.332419503 | NA | 0.332419503 | NA | NA | NA | NA |
| Albiglutide:Dapagliflozin_low_dosage | 0 | 1.588736549 | NA | 1.588736549 | NA | NA | NA | NA |
| Albiglutide:Dapagliflozin_medium_dosage | 0 | 1.612859704 | NA | 1.612859704 | NA | NA | NA | NA |
| Albiglutide:Dulaglutide | 0 | -0.308173616 | NA | -0.308173616 | NA | NA | NA | NA |
| Albiglutide:Efpeglenatide_high_dosage | 0 | -0.625711741 | NA | -0.625711741 | NA | NA | NA | NA |
| Albiglutide:Efpeglenatide_medium_dosage | 0 | -1.135800988 | NA | -1.135800988 | NA | NA | NA | NA |
| Albiglutide:Empagliflozin_high_dosage | 0 | 0.784499934 | NA | 0.784499934 | NA | NA | NA | NA |
| Albiglutide:Empagliflozin_low_dosage | 0 | -0.15604213 | NA | -0.15604213 | NA | NA | NA | NA |
| Albiglutide:Ertugliflozin_high_dosage | 0 | -0.085710886 | NA | -0.085710886 | NA | NA | NA | NA |
| Albiglutide:Ertugliflozin_low_dosage | 0 | 0.297959729 | NA | 0.297959729 | NA | NA | NA | NA |
| Albiglutide:Exenatide | 0 | 0.393868739 | NA | 0.393868739 | NA | NA | NA | NA |
| Albiglutide:Inject_semaglutide_high_dosage | 0 | 0.432847823 | NA | 0.432847823 | NA | NA | NA | NA |
| Albiglutide:Inject_semaglutide_low_dosage | 0 | 1.425818538 | NA | 1.425818538 | NA | NA | NA | NA |
| Albiglutide:Inject_semaglutide_medium_dosage | 0 | 1.327328856 | NA | 1.327328856 | NA | NA | NA | NA |
| Albiglutide:Liraglutide | 0 | 0.635347908 | NA | 0.635347908 | NA | NA | NA | NA |
| Albiglutide:Lixisenatide | 0 | 0.993452682 | NA | 0.993452682 | NA | NA | NA | NA |
| Albiglutide:Oral_semaglutide | 0 | 0.602660348 | NA | 0.602660348 | NA | NA | NA | NA |

| | | | | | | | | |
|---|---|---|---|---|---|---|---|---|
| Albiglutide:Placebo_or_Control | 4 | 0.471426164 | 0.471426164 | NA | NA | NA | NA | NA |
| Albiglutide:Sotagliflozin | 0 | 0.264705656 | NA | 0.264705656 | NA | NA | NA | NA |
| Albiglutide:Tirzepatide | 0 | 2.425174515 | NA | 2.425174515 | NA | NA | NA | NA |
| Bexagliflozin:Canagliflozin_high_dosage | 0 | 1.206367869 | NA | 1.206367869 | NA | NA | NA | NA |
| Bexagliflozin:Canagliflozin_low_dosage | 0 | 0.767043304 | NA | 0.767043304 | NA | NA | NA | NA |
| Bexagliflozin:Dapagliflozin_high_dosage | 0 | 0.269546912 | NA | 0.269546912 | NA | NA | NA | NA |
| Bexagliflozin:Dapagliflozin_low_dosage | 0 | 1.525863958 | NA | 1.525863958 | NA | NA | NA | NA |
| Bexagliflozin:Dapagliflozin_medium_dosage | 0 | 1.549987112 | NA | 1.549987112 | NA | NA | NA | NA |
| Bexagliflozin:Dulaglutide | 0 | -0.371046208 | NA | -0.371046208 | NA | NA | NA | NA |
| Bexagliflozin:Efpeglenatide_high_dosage | 0 | -0.688584333 | NA | -0.688584333 | NA | NA | NA | NA |
| Bexagliflozin:Efpeglenatide_medium_dosage | 0 | -1.19867358 | NA | -1.19867358 | NA | NA | NA | NA |
| Bexagliflozin:Empagliflozin_high_dosage | 0 | 0.721627343 | NA | 0.721627343 | NA | NA | NA | NA |
| Bexagliflozin:Empagliflozin_low_dosage | 0 | -0.218914722 | NA | -0.218914722 | NA | NA | NA | NA |
| Bexagliflozin:Ertugliflozin_high_dosage | 0 | -0.148583478 | NA | -0.148583478 | NA | NA | NA | NA |
| Bexagliflozin:Ertugliflozin_low_dosage | 0 | 0.235087138 | NA | 0.235087138 | NA | NA | NA | NA |
| Bexagliflozin:Exenatide | 0 | 0.330996147 | NA | 0.330996147 | NA | NA | NA | NA |
| Bexagliflozin:Inject_semaglutide_high_dosage | 0 | 0.369975232 | NA | 0.369975232 | NA | NA | NA | NA |
| Bexagliflozin:Inject_semaglutide_low_dosage | 0 | 1.362945947 | NA | 1.362945947 | NA | NA | NA | NA |
| Bexagliflozin:Inject_semaglutide_medium_dosage | 0 | 1.264456264 | NA | 1.264456264 | NA | NA | NA | NA |
| Bexagliflozin:Liraglutide | 0 | 0.572475317 | NA | 0.572475317 | NA | NA | NA | NA |
| Bexagliflozin:Lixisenatide | 0 | 0.930580091 | NA | 0.930580091 | NA | NA | NA | NA |
| Bexagliflozin:Oral_semaglutide | 0 | 0.539787757 | NA | 0.539787757 | NA | NA | NA | NA |

| | | | | | | | | |
|---|---|---|---|---|---|---|---|---|
| Bexagliflozin:Placebo_or_Control | 1 | 0.408553573 | 0.408553573 | NA | NA | NA | NA | NA |
| Bexagliflozin:Sotagliflozin | 0 | 0.201833065 | NA | 0.201833065 | NA | NA | NA | NA |
| Bexagliflozin:Tirzepatide | 0 | 2.362301923 | NA | 2.362301923 | NA | NA | NA | NA |
| Canagliflozin_high_dosage:Canagliflozin_low_dosage | 1 | -0.439324565 | -0.285598015 | -0.888116525 | 0.60251851 | -2.364592716 | 3.569629737 | 0.690629114 |
| Canagliflozin_high_dosage:Dapagliflozin_high_dosage | 0 | -0.936820957 | NA | -0.936820957 | NA | NA | NA | NA |
| Canagliflozin_high_dosage:Dapagliflozin_low_dosage | 0 | 0.319496088 | NA | 0.319496088 | NA | NA | NA | NA |
| Canagliflozin_high_dosage:Dapagliflozin_medium_dosage | 0 | 0.343619243 | NA | 0.343619243 | NA | NA | NA | NA |
| Canagliflozin_high_dosage:Dulaglutide | 0 | -1.577414077 | NA | -1.577414077 | NA | NA | NA | NA |
| Canagliflozin_high_dosage:Efpeglenatide_high_dosage | 0 | -1.894952202 | NA | -1.894952202 | NA | NA | NA | NA |
| Canagliflozin_high_dosage:Efpeglenatide_medium_dosage | 0 | -2.405041449 | NA | -2.405041449 | NA | NA | NA | NA |
| Canagliflozin_high_dosage:Empagliflozin_high_dosage | 0 | -0.484740526 | NA | -0.484740526 | NA | NA | NA | NA |
| Canagliflozin_high_dosage:Empagliflozin_low_dosage | 0 | -1.425282591 | NA | -1.425282591 | NA | NA | NA | NA |
| Canagliflozin_high_dosage:Ertugliflozin_high_dosage | 0 | -1.354951347 | NA | -1.354951347 | NA | NA | NA | NA |
| Canagliflozin_high_dosage:Ertugliflozin_low_dosage | 0 | -0.971280732 | NA | -0.971280732 | NA | NA | NA | NA |
| Canagliflozin_high_dosage:Exenatide | 0 | -0.875371722 | NA | -0.875371722 | NA | NA | NA | NA |
| Canagliflozin_high_dosage:Inject_semaglutide_high_dosage | 0 | -0.836392637 | NA | -0.836392637 | NA | NA | NA | NA |
| Canagliflozin_high_dosage:Inject_semaglutide_low_dosage | 0 | 0.156578078 | NA | 0.156578078 | NA | NA | NA | NA |
| Canagliflozin_high_dosage:Inject_semaglutide_medium_dosage | 0 | 0.058088395 | NA | 0.058088395 | NA | NA | NA | NA |
| Canagliflozin_high_dosage:Liraglutide | 0 | -0.633892552 | NA | -0.633892552 | NA | NA | NA | NA |
| Canagliflozin_high_dosage:Lixisenatide | 0 | -0.275787778 | NA | -0.275787778 | NA | NA | NA | NA |
| Canagliflozin_high_dosage:Oral_semaglutide | 0 | -0.666580112 | NA | -0.666580112 | NA | NA | NA | NA |
| Canagliflozin_high_dosage:Placebo_or_Control | 2 | -0.797814296 | -0.587305465 | -2.055953315 | 1.46864785 | -2.12741505 | 5.064710751 | 0.423445557 |

| | | | | | | | | |
|---|---|---|---|---|---|---|---|---|
| Canagliflozin_high_dosage:Sotagliflozin | 0 | -1.004534804 | NA | -1.004534804 | NA | NA | NA | NA |
| Canagliflozin_high_dosage:Tirzepatide | 0 | 1.155934054 | NA | 1.155934054 | NA | NA | NA | NA |
| Canagliflozin_low_dosage:Dapagliflozin_high_dosage | 0 | -0.497496392 | NA | -0.497496392 | NA | NA | NA | NA |
| Canagliflozin_low_dosage:Dapagliflozin_low_dosage | 0 | 0.758820653 | NA | 0.758820653 | NA | NA | NA | NA |
| Canagliflozin_low_dosage:Dapagliflozin_medium_dosage | 0 | 0.782943808 | NA | 0.782943808 | NA | NA | NA | NA |
| Canagliflozin_low_dosage:Dulaglutide | 0 | -1.138089512 | NA | -1.138089512 | NA | NA | NA | NA |
| Canagliflozin_low_dosage:Efpeglenatide_high_dosage | 0 | -1.455627637 | NA | -1.455627637 | NA | NA | NA | NA |
| Canagliflozin_low_dosage:Efpeglenatide_medium_dosage | 0 | -1.965716884 | NA | -1.965716884 | NA | NA | NA | NA |
| Canagliflozin_low_dosage:Empagliflozin_high_dosage | 0 | -0.045415961 | NA | -0.045415961 | NA | NA | NA | NA |
| Canagliflozin_low_dosage:Empagliflozin_low_dosage | 0 | -0.985958026 | NA | -0.985958026 | NA | NA | NA | NA |
| Canagliflozin_low_dosage:Ertugliflozin_high_dosage | 0 | -0.915626782 | NA | -0.915626782 | NA | NA | NA | NA |
| Canagliflozin_low_dosage:Ertugliflozin_low_dosage | 0 | -0.531956167 | NA | -0.531956167 | NA | NA | NA | NA |
| Canagliflozin_low_dosage:Exenatide | 0 | -0.436047157 | NA | -0.436047157 | NA | NA | NA | NA |
| Canagliflozin_low_dosage:Inject_semaglutide_high_dosage | 0 | -0.397068073 | NA | -0.397068073 | NA | NA | NA | NA |
| Canagliflozin_low_dosage:Inject_semaglutide_low_dosage | 0 | 0.595902642 | NA | 0.595902642 | NA | NA | NA | NA |
| Canagliflozin_low_dosage:Inject_semaglutide_medium_dosage | 0 | 0.49741296 | NA | 0.49741296 | NA | NA | NA | NA |
| Canagliflozin_low_dosage:Liraglutide | 0 | -0.194567987 | NA | -0.194567987 | NA | NA | NA | NA |
| Canagliflozin_low_dosage:Lixisenatide | 0 | 0.163536786 | NA | 0.163536786 | NA | NA | NA | NA |
| Canagliflozin_low_dosage:Oral_semaglutide | 0 | -0.227255548 | NA | -0.227255548 | NA | NA | NA | NA |
| Canagliflozin_low_dosage:Placebo_or_Control | 3 | -0.358489731 | -0.13765041 | -2.841598052 | 2.703947642 | -1.232231013 | 6.640126296 | 0.178176085 |
| Canagliflozin_low_dosage:Sotagliflozin | 0 | -0.565210239 | NA | -0.565210239 | NA | NA | NA | NA |
| Canagliflozin_low_dosage:Tirzepatide | 0 | 1.595258619 | NA | 1.595258619 | NA | NA | NA | NA |

| | | | | | | | | |
|---|---|---|---|---|---|---|---|---|
| Dapagliflozin_high_dosage:Dapagliflozin_low_dosage | 0 | 1.256317046 | NA | 1.256317046 | NA | NA | NA | NA |
| Dapagliflozin_high_dosage:Dapagliflozin_medium_dosage | 0 | 1.2804402 | NA | 1.2804402 | NA | NA | NA | NA |
| Dapagliflozin_high_dosage:Dulaglutide | 0 | -0.64059312 | NA | -0.64059312 | NA | NA | NA | NA |
| Dapagliflozin_high_dosage:Efpeglenatide_high_dosage | 0 | -0.958131245 | NA | -0.958131245 | NA | NA | NA | NA |
| Dapagliflozin_high_dosage:Efpeglenatide_medium_dosage | 0 | -1.468220492 | NA | -1.468220492 | NA | NA | NA | NA |
| Dapagliflozin_high_dosage:Empagliflozin_high_dosage | 0 | 0.452080431 | NA | 0.452080431 | NA | NA | NA | NA |
| Dapagliflozin_high_dosage:Empagliflozin_low_dosage | 0 | -0.488461634 | NA | -0.488461634 | NA | NA | NA | NA |
| Dapagliflozin_high_dosage:Ertugliflozin_high_dosage | 0 | -0.41813039 | NA | -0.41813039 | NA | NA | NA | NA |
| Dapagliflozin_high_dosage:Ertugliflozin_low_dosage | 0 | -0.034459775 | NA | -0.034459775 | NA | NA | NA | NA |
| Dapagliflozin_high_dosage:Exenatide | 0 | 0.061449235 | NA | 0.061449235 | NA | NA | NA | NA |
| Dapagliflozin_high_dosage:Inject_semaglutide_high_dosage | 0 | 0.10042832 | NA | 0.10042832 | NA | NA | NA | NA |
| Dapagliflozin_high_dosage:Inject_semaglutide_low_dosage | 0 | 1.093399035 | NA | 1.093399035 | NA | NA | NA | NA |
| Dapagliflozin_high_dosage:Inject_semaglutide_medium_dosage | 0 | 0.994909352 | NA | 0.994909352 | NA | NA | NA | NA |
| Dapagliflozin_high_dosage:Liraglutide | 0 | 0.302928405 | NA | 0.302928405 | NA | NA | NA | NA |
| Dapagliflozin_high_dosage:Lixisenatide | 0 | 0.661033179 | NA | 0.661033179 | NA | NA | NA | NA |
| Dapagliflozin_high_dosage:Oral_semaglutide | 0 | 0.270240845 | NA | 0.270240845 | NA | NA | NA | NA |
| Dapagliflozin_high_dosage:Placebo_or_Control | 6 | 0.139006661 | 0.139006661 | NA | NA | NA | NA | NA |
| Dapagliflozin_high_dosage:Sotagliflozin | 0 | -0.067713847 | NA | -0.067713847 | NA | NA | NA | NA |
| Dapagliflozin_high_dosage:Tirzepatide | 0 | 2.092755011 | NA | 2.092755011 | NA | NA | NA | NA |
| Dapagliflozin_low_dosage:Dapagliflozin_medium_dosage | 0 | 0.024123155 | NA | 0.024123155 | NA | NA | NA | NA |
| Dapagliflozin_low_dosage:Dulaglutide | 0 | -1.896910165 | NA | -1.896910165 | NA | NA | NA | NA |
| Dapagliflozin_low_dosage:Efpeglenatide_high_dosage | 0 | -2.21444829 | NA | -2.21444829 | NA | NA | NA | NA |

| | | | | | | | | |
|---|---|---|---|---|---|---|---|---|
| Dapagliflozin_low_dosage:Efpeglenatide_medium_dosage | 0 | -2.724537537 | NA | -2.724537537 | NA | NA | NA | NA |
| Dapagliflozin_low_dosage:Empagliflozin_high_dosage | 0 | -0.804236615 | NA | -0.804236615 | NA | NA | NA | NA |
| Dapagliflozin_low_dosage:Empagliflozin_low_dosage | 0 | -1.744778679 | NA | -1.744778679 | NA | NA | NA | NA |
| Dapagliflozin_low_dosage:Ertugliflozin_high_dosage | 0 | -1.674447435 | NA | -1.674447435 | NA | NA | NA | NA |
| Dapagliflozin_low_dosage:Ertugliflozin_low_dosage | 0 | -1.29077682 | NA | -1.29077682 | NA | NA | NA | NA |
| Dapagliflozin_low_dosage:Exenatide | 0 | -1.19486781 | NA | -1.19486781 | NA | NA | NA | NA |
| Dapagliflozin_low_dosage:Inject_semaglutide_high_dosage | 0 | -1.155888726 | NA | -1.155888726 | NA | NA | NA | NA |
| Dapagliflozin_low_dosage:Inject_semaglutide_low_dosage | 0 | -0.162918011 | NA | -0.162918011 | NA | NA | NA | NA |
| Dapagliflozin_low_dosage:Inject_semaglutide_medium_dosage | 0 | -0.261407694 | NA | -0.261407694 | NA | NA | NA | NA |
| Dapagliflozin_low_dosage:Liraglutide | 0 | -0.953388641 | NA | -0.953388641 | NA | NA | NA | NA |
| Dapagliflozin_low_dosage:Lixisenatide | 0 | -0.595283867 | NA | -0.595283867 | NA | NA | NA | NA |
| Dapagliflozin_low_dosage:Oral_semaglutide | 0 | -0.986076201 | NA | -0.986076201 | NA | NA | NA | NA |
| Dapagliflozin_low_dosage:Placebo_or_Control | 2 | -1.117310385 | -1.117310385 | NA | NA | NA | NA | NA |
| Dapagliflozin_low_dosage:Sotagliflozin | 0 | -1.324030893 | NA | -1.324030893 | NA | NA | NA | NA |
| Dapagliflozin_low_dosage:Tirzepatide | 0 | 0.836437966 | NA | 0.836437966 | NA | NA | NA | NA |
| Dapagliflozin_medium_dosage:Dulaglutide | 0 | -1.92103332 | NA | -1.92103332 | NA | NA | NA | NA |
| Dapagliflozin_medium_dosage:Efpeglenatide_high_dosage | 0 | -2.238571445 | NA | -2.238571445 | NA | NA | NA | NA |
| Dapagliflozin_medium_dosage:Efpeglenatide_medium_dosage | 0 | -2.748660692 | NA | -2.748660692 | NA | NA | NA | NA |
| Dapagliflozin_medium_dosage:Empagliflozin_high_dosage | 0 | -0.828359769 | NA | -0.828359769 | NA | NA | NA | NA |
| Dapagliflozin_medium_dosage:Empagliflozin_low_dosage | 0 | -1.768901834 | NA | -1.768901834 | NA | NA | NA | NA |
| Dapagliflozin_medium_dosage:Ertugliflozin_high_dosage | 0 | -1.69857059 | NA | -1.69857059 | NA | NA | NA | NA |
| Dapagliflozin_medium_dosage:Ertugliflozin_low_dosage | 0 | -1.314899975 | NA | -1.314899975 | NA | NA | NA | NA |

| | | | | | | | | |
|---|---|---|---|---|---|---|---|---|
| Dapagliflozin_medium_dosage:Exenatide | 0 | -1.218990965 | NA | -1.218990965 | NA | NA | NA | NA |
| Dapagliflozin_medium_dosage:Inject_semaglutide_high_dosage | 0 | -1.180011881 | NA | -1.180011881 | NA | NA | NA | NA |
| Dapagliflozin_medium_dosage:Inject_semaglutide_low_dosage | 0 | -0.187041166 | NA | -0.187041166 | NA | NA | NA | NA |
| Dapagliflozin_medium_dosage:Inject_semaglutide_medium_dosage | 0 | -0.285530848 | NA | -0.285530848 | NA | NA | NA | NA |
| Dapagliflozin_medium_dosage:Liraglutide | 0 | -0.977511795 | NA | -0.977511795 | NA | NA | NA | NA |
| Dapagliflozin_medium_dosage:Lixisenatide | 0 | -0.619407021 | NA | -0.619407021 | NA | NA | NA | NA |
| Dapagliflozin_medium_dosage:Oral_semaglutide | 0 | -1.010199356 | NA | -1.010199356 | NA | NA | NA | NA |
| Dapagliflozin_medium_dosage:Placebo_or_Control | 2 | -1.141433539 | -1.141433539 | NA | NA | NA | NA | NA |
| Dapagliflozin_medium_dosage:Sotagliflozin | 0 | -1.348154047 | NA | -1.348154047 | NA | NA | NA | NA |
| Dapagliflozin_medium_dosage:Tirzepatide | 0 | 0.812314811 | NA | 0.812314811 | NA | NA | NA | NA |
| Dulaglutide:Efpeglenatide_high_dosage | 0 | -0.317538125 | NA | -0.317538125 | NA | NA | NA | NA |
| Dulaglutide:Efpeglenatide_medium_dosage | 0 | -0.827627372 | NA | -0.827627372 | NA | NA | NA | NA |
| Dulaglutide:Empagliflozin_high_dosage | 0 | 1.092673551 | NA | 1.092673551 | NA | NA | NA | NA |
| Dulaglutide:Empagliflozin_low_dosage | 0 | 0.152131486 | NA | 0.152131486 | NA | NA | NA | NA |
| Dulaglutide:Ertugliflozin_high_dosage | 0 | 0.22246273 | NA | 0.22246273 | NA | NA | NA | NA |
| Dulaglutide:Ertugliflozin_low_dosage | 0 | 0.606133345 | NA | 0.606133345 | NA | NA | NA | NA |
| Dulaglutide:Exenatide | 0 | 0.702042355 | NA | 0.702042355 | NA | NA | NA | NA |
| Dulaglutide:Inject_semaglutide_high_dosage | 0 | 0.741021439 | NA | 0.741021439 | NA | NA | NA | NA |
| Dulaglutide:Inject_semaglutide_low_dosage | 0 | 1.733992154 | NA | 1.733992154 | NA | NA | NA | NA |
| Dulaglutide:Inject_semaglutide_medium_dosage | 0 | 1.635502472 | NA | 1.635502472 | NA | NA | NA | NA |
| Dulaglutide:Liraglutide | 0 | 0.943521525 | NA | 0.943521525 | NA | NA | NA | NA |
| Dulaglutide:Lixisenatide | 0 | 1.301626299 | NA | 1.301626299 | NA | NA | NA | NA |

| | | | | | | | | |
|---|---|---|---|---|---|---|---|---|
| Dulaglutide:Oral_semaglutide | 0 | 0.910833964 | NA | 0.910833964 | NA | NA | NA | NA |
| Dulaglutide:Placebo_or_Control | 3 | 0.779599781 | 0.779599781 | NA | NA | NA | NA | NA |
| Dulaglutide:Sotagliflozin | 0 | 0.572879273 | NA | 0.572879273 | NA | NA | NA | NA |
| Dulaglutide:Tirzepatide | 0 | 2.733348131 | NA | 2.733348131 | NA | NA | NA | NA |
| Efpeglenatide_high_dosage:Efpeglenatide_medium_dosage | 1 | -0.510089247 | -0.510089247 | NA | NA | NA | NA | NA |
| Efpeglenatide_high_dosage:Empagliflozin_high_dosage | 0 | 1.410211676 | NA | 1.410211676 | NA | NA | NA | NA |
| Efpeglenatide_high_dosage:Empagliflozin_low_dosage | 0 | 0.469669611 | NA | 0.469669611 | NA | NA | NA | NA |
| Efpeglenatide_high_dosage:Ertugliflozin_high_dosage | 0 | 0.540000855 | NA | 0.540000855 | NA | NA | NA | NA |
| Efpeglenatide_high_dosage:Ertugliflozin_low_dosage | 0 | 0.92367147 | NA | 0.92367147 | NA | NA | NA | NA |
| Efpeglenatide_high_dosage:Exenatide | 0 | 1.01958048 | NA | 1.01958048 | NA | NA | NA | NA |
| Efpeglenatide_high_dosage:Inject_semaglutide_high_dosage | 0 | 1.058559565 | NA | 1.058559565 | NA | NA | NA | NA |
| Efpeglenatide_high_dosage:Inject_semaglutide_low_dosage | 0 | 2.05153028 | NA | 2.05153028 | NA | NA | NA | NA |
| Efpeglenatide_high_dosage:Inject_semaglutide_medium_dosage | 0 | 1.953040597 | NA | 1.953040597 | NA | NA | NA | NA |
| Efpeglenatide_high_dosage:Liraglutide | 0 | 1.26105965 | NA | 1.26105965 | NA | NA | NA | NA |
| Efpeglenatide_high_dosage:Lixisenatide | 0 | 1.619164424 | NA | 1.619164424 | NA | NA | NA | NA |
| Efpeglenatide_high_dosage:Oral_semaglutide | 0 | 1.22837209 | NA | 1.22837209 | NA | NA | NA | NA |
| Efpeglenatide_high_dosage:Placebo_or_Control | 1 | 1.097137906 | 1.097137906 | NA | NA | NA | NA | NA |
| Efpeglenatide_high_dosage:Sotagliflozin | 0 | 0.890417398 | NA | 0.890417398 | NA | NA | NA | NA |
| Efpeglenatide_high_dosage:Tirzepatide | 0 | 3.050886256 | NA | 3.050886256 | NA | NA | NA | NA |
| Efpeglenatide_medium_dosage:Empagliflozin_high_dosage | 0 | 1.920300922 | NA | 1.920300922 | NA | NA | NA | NA |
| Efpeglenatide_medium_dosage:Empagliflozin_low_dosage | 0 | 0.979758858 | NA | 0.979758858 | NA | NA | NA | NA |
| Efpeglenatide_medium_dosage:Ertugliflozin_high_dosage | 0 | 1.050090102 | NA | 1.050090102 | NA | NA | NA | NA |

| | | | | | | | | |
|---|---|---|---|---|---|---|---|---|
| Efpeglenatide_medium_dosage:Ertugliflozin_low_dosage | 0 | 1.433760717 | NA | 1.433760717 | NA | NA | NA | NA |
| Efpeglenatide_medium_dosage:Exenatide | 0 | 1.529669727 | NA | 1.529669727 | NA | NA | NA | NA |
| Efpeglenatide_medium_dosage:Inject_semaglutide_high_dosage | 0 | 1.568648811 | NA | 1.568648811 | NA | NA | NA | NA |
| Efpeglenatide_medium_dosage:Inject_semaglutide_low_dosage | 0 | 2.561619526 | NA | 2.561619526 | NA | NA | NA | NA |
| Efpeglenatide_medium_dosage:Inject_semaglutide_medium_dosage | 0 | 2.463129844 | NA | 2.463129844 | NA | NA | NA | NA |
| Efpeglenatide_medium_dosage:Liraglutide | 0 | 1.771148897 | NA | 1.771148897 | NA | NA | NA | NA |
| Efpeglenatide_medium_dosage:Lixisenatide | 0 | 2.12925367 | NA | 2.12925367 | NA | NA | NA | NA |
| Efpeglenatide_medium_dosage:Oral_semaglutide | 0 | 1.738461336 | NA | 1.738461336 | NA | NA | NA | NA |
| Efpeglenatide_medium_dosage:Placebo_or_Control | 1 | 1.607227153 | 1.607227153 | NA | NA | NA | NA | NA |
| Efpeglenatide_medium_dosage:Sotagliflozin | 0 | 1.400506645 | NA | 1.400506645 | NA | NA | NA | NA |
| Efpeglenatide_medium_dosage:Tirzepatide | 0 | 3.560975503 | NA | 3.560975503 | NA | NA | NA | NA |
| Empagliflozin_high_dosage:Empagliflozin_low_dosage | 3 | -0.940542064 | -0.981454702 | -0.798921716 | -0.182532986 | -2.864964464 | 2.499898491 | 0.893900123 |
| Empagliflozin_high_dosage:Ertugliflozin_high_dosage | 0 | -0.87021082 | NA | -0.87021082 | NA | NA | NA | NA |
| Empagliflozin_high_dosage:Ertugliflozin_low_dosage | 0 | -0.486540205 | NA | -0.486540205 | NA | NA | NA | NA |
| Empagliflozin_high_dosage:Exenatide | 0 | -0.390631196 | NA | -0.390631196 | NA | NA | NA | NA |
| Empagliflozin_high_dosage:Inject_semaglutide_high_dosage | 0 | -0.351652111 | NA | -0.351652111 | NA | NA | NA | NA |
| Empagliflozin_high_dosage:Inject_semaglutide_low_dosage | 0 | 0.641318604 | NA | 0.641318604 | NA | NA | NA | NA |
| Empagliflozin_high_dosage:Inject_semaglutide_medium_dosage | 0 | 0.542828921 | NA | 0.542828921 | NA | NA | NA | NA |
| Empagliflozin_high_dosage:Liraglutide | 0 | -0.149152026 | NA | -0.149152026 | NA | NA | NA | NA |
| Empagliflozin_high_dosage:Lixisenatide | 0 | 0.208952748 | NA | 0.208952748 | NA | NA | NA | NA |
| Empagliflozin_high_dosage:Oral_semaglutide | 0 | -0.181839586 | NA | -0.181839586 | NA | NA | NA | NA |
| Empagliflozin_high_dosage:Placebo_or_Control | 3 | -0.31307377 | -0.532377685 | 0.253625151 | -0.786002836 | -3.358312295 | 1.786306623 | 0.549244467 |

| | | | | | | | | |
|---|---|---|---|---|---|---|---|---|
| Empagliflozin_high_dosage:Sotagliflozin | 0 | -0.519794278 | NA | -0.519794278 | NA | NA | NA | NA |
| Empagliflozin_high_dosage:Tirzepatide | 0 | 1.64067458 | NA | 1.64067458 | NA | NA | NA | NA |
| Empagliflozin_low_dosage:Ertugliflozin_high_dosage | 0 | 0.070331244 | NA | 0.070331244 | NA | NA | NA | NA |
| Empagliflozin_low_dosage:Ertugliflozin_low_dosage | 0 | 0.454001859 | NA | 0.454001859 | NA | NA | NA | NA |
| Empagliflozin_low_dosage:Exenatide | 0 | 0.549910869 | NA | 0.549910869 | NA | NA | NA | NA |
| Empagliflozin_low_dosage:Inject_semaglutide_high_dosage | 0 | 0.588889953 | NA | 0.588889953 | NA | NA | NA | NA |
| Empagliflozin_low_dosage:Inject_semaglutide_low_dosage | 0 | 1.581860668 | NA | 1.581860668 | NA | NA | NA | NA |
| Empagliflozin_low_dosage:Inject_semaglutide_medium_dosage | 0 | 1.483370986 | NA | 1.483370986 | NA | NA | NA | NA |
| Empagliflozin_low_dosage:Liraglutide | 0 | 0.791390039 | NA | 0.791390039 | NA | NA | NA | NA |
| Empagliflozin_low_dosage:Lixisenatide | 0 | 1.149494812 | NA | 1.149494812 | NA | NA | NA | NA |
| Empagliflozin_low_dosage:Oral_semaglutide | 0 | 0.758702478 | NA | 0.758702478 | NA | NA | NA | NA |
| Empagliflozin_low_dosage:Placebo_or_Control | 5 | 0.627468295 | 0.650204734 | 0.093247386 | 0.556957348 | -2.592098009 | 3.706012704 | 0.72885516 |
| Empagliflozin_low_dosage:Sotagliflozin | 0 | 0.420747787 | NA | 0.420747787 | NA | NA | NA | NA |
| Empagliflozin_low_dosage:Tirzepatide | 0 | 2.581216645 | NA | 2.581216645 | NA | NA | NA | NA |
| Ertugliflozin_high_dosage:Ertugliflozin_low_dosage | 3 | 0.383670615 | 0.335598871 | 1.831310087 | -1.495711216 | -6.982657788 | 3.991235357 | 0.593151067 |
| Ertugliflozin_high_dosage:Exenatide | 0 | 0.479579625 | NA | 0.479579625 | NA | NA | NA | NA |
| Ertugliflozin_high_dosage:Inject_semaglutide_high_dosage | 0 | 0.518558709 | NA | 0.518558709 | NA | NA | NA | NA |
| Ertugliflozin_high_dosage:Inject_semaglutide_low_dosage | 0 | 1.511529424 | NA | 1.511529424 | NA | NA | NA | NA |
| Ertugliflozin_high_dosage:Inject_semaglutide_medium_dosage | 0 | 1.413039742 | NA | 1.413039742 | NA | NA | NA | NA |
| Ertugliflozin_high_dosage:Liraglutide | 0 | 0.721058795 | NA | 0.721058795 | NA | NA | NA | NA |
| Ertugliflozin_high_dosage:Lixisenatide | 0 | 1.079163568 | NA | 1.079163568 | NA | NA | NA | NA |
| Ertugliflozin_high_dosage:Oral_semaglutide | 0 | 0.688371234 | NA | 0.688371234 | NA | NA | NA | NA |

| | | | | | | | | |
|---|---|---|---|---|---|---|---|---|
| Ertugliflozin_high_dosage:Placebo_or_Control | 3 | 0.557137051 | 0.494571754 | 2.650338031 | -2.155766278 | -8.171679925 | 3.86014737 | 0.482466379 |
| Ertugliflozin_high_dosage:Sotagliflozin | 0 | 0.350416543 | NA | 0.350416543 | NA | NA | NA | NA |
| Ertugliflozin_high_dosage:Tirzepatide | 0 | 2.510885401 | NA | 2.510885401 | NA | NA | NA | NA |
| Ertugliflozin_low_dosage:Exenatide | 0 | 0.09590901 | NA | 0.09590901 | NA | NA | NA | NA |
| Ertugliflozin_low_dosage:Inject_semaglutide_high_dosage | 0 | 0.134888094 | NA | 0.134888094 | NA | NA | NA | NA |
| Ertugliflozin_low_dosage:Inject_semaglutide_low_dosage | 0 | 1.127858809 | NA | 1.127858809 | NA | NA | NA | NA |
| Ertugliflozin_low_dosage:Inject_semaglutide_medium_dosage | 0 | 1.029369127 | NA | 1.029369127 | NA | NA | NA | NA |
| Ertugliflozin_low_dosage:Liraglutide | 0 | 0.337388179 | NA | 0.337388179 | NA | NA | NA | NA |
| Ertugliflozin_low_dosage:Lixisenatide | 0 | 0.695492953 | NA | 0.695492953 | NA | NA | NA | NA |
| Ertugliflozin_low_dosage:Oral_semaglutide | 0 | 0.304700619 | NA | 0.304700619 | NA | NA | NA | NA |
| Ertugliflozin_low_dosage:Placebo_or_Control | 1 | 0.173466435 | 0.182321557 | 0.109312861 | 0.073008696 | -3.338179572 | 3.484196964 | 0.96653971 |
| Ertugliflozin_low_dosage:Sotagliflozin | 0 | -0.033254073 | NA | -0.033254073 | NA | NA | NA | NA |
| Ertugliflozin_low_dosage:Tirzepatide | 0 | 2.127214786 | NA | 2.127214786 | NA | NA | NA | NA |
| Exenatide:Inject_semaglutide_high_dosage | 0 | 0.038979084 | NA | 0.038979084 | NA | NA | NA | NA |
| Exenatide:Inject_semaglutide_low_dosage | 0 | 1.031949799 | NA | 1.031949799 | NA | NA | NA | NA |
| Exenatide:Inject_semaglutide_medium_dosage | 0 | 0.933460117 | NA | 0.933460117 | NA | NA | NA | NA |
| Exenatide:Liraglutide | 1 | 0.24147917 | 1.115743329 | 0.182215554 | 0.933527774 | -2.379088644 | 4.246144192 | 0.580717444 |
| Exenatide:Lixisenatide | 0 | 0.599583943 | NA | 0.599583943 | NA | NA | NA | NA |
| Exenatide:Oral_semaglutide | 0 | 0.208791609 | NA | 0.208791609 | NA | NA | NA | NA |
| Exenatide:Placebo_or_Control | 1 | 0.077557426 | 0.048405145 | 0.981932919 | -0.933527774 | -4.246144192 | 2.379088644 | 0.580717444 |
| Exenatide:Sotagliflozin | 0 | -0.129163082 | NA | -0.129163082 | NA | NA | NA | NA |
| Exenatide:Tirzepatide | 0 | 2.031305776 | NA | 2.031305776 | NA | NA | NA | NA |

| | | | | | | | | |
|---|---|---|---|---|---|---|---|---|
| Inject_semaglutide_high_dosage:Inject_semaglutide_low_dosage | 0 | 0.992970715 | NA | 0.992970715 | NA | NA | NA | NA |
| Inject_semaglutide_high_dosage:Inject_semaglutide_medium_dosage | 1 | 0.894481032 | 1.611925476 | 0.737494445 | 0.874431031 | -2.481183207 | 4.230045269 | 0.609531685 |
| Inject_semaglutide_high_dosage:Liraglutide | 0 | 0.202500085 | NA | 0.202500085 | NA | NA | NA | NA |
| Inject_semaglutide_high_dosage:Lixisenatide | 0 | 0.560604859 | NA | 0.560604859 | NA | NA | NA | NA |
| Inject_semaglutide_high_dosage:Oral_semaglutide | 0 | 0.169812525 | NA | 0.169812525 | NA | NA | NA | NA |
| Inject_semaglutide_high_dosage:Placebo_or_Control | 4 | 0.038578341 | 0.012948973 | 0.887380004 | -0.874431031 | -4.230045269 | 2.481183207 | 0.609531685 |
| Inject_semaglutide_high_dosage:Sotagliflozin | 0 | -0.168142167 | NA | -0.168142167 | NA | NA | NA | NA |
| Inject_semaglutide_high_dosage:Tirzepatide | 0 | 1.992326692 | NA | 1.992326692 | NA | NA | NA | NA |
| Inject_semaglutide_low_dosage:Inject_semaglutide_medium_dosage | 2 | -0.098489683 | -0.048130021 | -0.335218098 | 0.287088077 | -4.30297713 | 4.877153284 | 0.902434155 |
| Inject_semaglutide_low_dosage:Liraglutide | 0 | -0.79047063 | NA | -0.79047063 | NA | NA | NA | NA |
| Inject_semaglutide_low_dosage:Lixisenatide | 0 | -0.432365856 | NA | -0.432365856 | NA | NA | NA | NA |
| Inject_semaglutide_low_dosage:Oral_semaglutide | 0 | -0.82315819 | NA | -0.82315819 | NA | NA | NA | NA |
| Inject_semaglutide_low_dosage:Placebo_or_Control | 2 | -0.954392374 | -1.072448502 | 1.759823751 | -2.832272253 | -10.35053098 | 4.685986476 | 0.460298131 |
| Inject_semaglutide_low_dosage:Sotagliflozin | 0 | -1.161112882 | NA | -1.161112882 | NA | NA | NA | NA |
| Inject_semaglutide_low_dosage:Tirzepatide | 0 | 0.999355977 | NA | 0.999355977 | NA | NA | NA | NA |
| Inject_semaglutide_medium_dosage:Liraglutide | 0 | -0.691980947 | NA | -0.691980947 | NA | NA | NA | NA |
| Inject_semaglutide_medium_dosage:Lixisenatide | 0 | -0.333876173 | NA | -0.333876173 | NA | NA | NA | NA |
| Inject_semaglutide_medium_dosage:Oral_semaglutide | 0 | -0.724668507 | NA | -0.724668507 | NA | NA | NA | NA |
| Inject_semaglutide_medium_dosage:Placebo_or_Control | 2 | -0.855902691 | -0.902926539 | -0.724395344 | -0.178531195 | -2.900414105 | 2.543351715 | 0.897708887 |
| Inject_semaglutide_medium_dosage:Sotagliflozin | 0 | -1.062623199 | NA | -1.062623199 | NA | NA | NA | NA |
| Inject_semaglutide_medium_dosage:Tirzepatide | 1 | 1.097845659 | 2.200066498 | 0.655506111 | 1.544560387 | -2.246249932 | 5.335370705 | 0.424531322 |
| Liraglutide:Lixisenatide | 1 | 0.358104774 | 1.103562794 | 0.216302341 | 0.887260453 | -2.610992225 | 4.385513132 | 0.619115082 |

| | | | | | | | | |
|---|---|---|---|---|---|---|---|---|
| Liraglutide:Oral_semaglutide | 0 | -0.03268756 | NA | -0.03268756 | NA | NA | NA | NA |
| Liraglutide:Placebo_or_Control | 3 | -0.163921744 | -0.159336038 | -0.234417885 | 0.075081847 | -2.368923135 | 2.519086829 | 0.951987021 |
| Liraglutide:Sotagliflozin | 0 | -0.370642252 | NA | -0.370642252 | NA | NA | NA | NA |
| Liraglutide:Tirzepatide | 0 | 1.789826606 | NA | 1.789826606 | NA | NA | NA | NA |
| Lixisenatide:Oral_semaglutide | 0 | -0.390792334 | NA | -0.390792334 | NA | NA | NA | NA |
| Lixisenatide:Placebo_or_Control | 1 | -0.522026518 | -0.405795523 | -1.293055976 | 0.887260453 | -2.610992225 | 4.385513132 | 0.619115082 |
| Lixisenatide:Sotagliflozin | 0 | -0.728747026 | NA | -0.728747026 | NA | NA | NA | NA |
| Lixisenatide:Tirzepatide | 0 | 1.431721833 | NA | 1.431721833 | NA | NA | NA | NA |
| Oral_semaglutide:Placebo_or_Control | 2 | -0.131234184 | -0.131234184 | NA | NA | NA | NA | NA |
| Oral_semaglutide:Sotagliflozin | 0 | -0.337954692 | NA | -0.337954692 | NA | NA | NA | NA |
| Oral_semaglutide:Tirzepatide | 0 | 1.822514167 | NA | 1.822514167 | NA | NA | NA | NA |
| Sotagliflozin:Placebo_or_Control | 4 | 0.206720508 | 0.206720508 | NA | NA | NA | NA | NA |
| Tirzepatide:Placebo_or_Control | 4 | -1.95374835 | -1.685536016 | -3.230096402 | 1.544560387 | -2.246249932 | 5.335370705 | 0.424531322 |
| Sotagliflozin:Tirzepatide | 0 | 2.160468858 | NA | 2.160468858 | NA | NA | NA | NA |

## Table S7B: inconsistency within the network meta-analysis of primary outcome: subgroup of lymphoma

| Comparison | No.Studies | NMA | Direct | Indirect | Difference | Diff_95CI_lower | Diff_95CI_upper | *p* value |
|---|---|---|---|---|---|---|---|---|
| Albiglutide:Bexagliflozin | 0 | 0.187481401 | NA | 0.187481401 | NA | NA | NA | NA |
| Albiglutide:Canagliflozin_high_dosage | 0 | 1.210733311 | NA | 1.210733311 | NA | NA | NA | NA |
| Albiglutide:Canagliflozin_low_dosage | 0 | 0.734828593 | NA | 0.734828593 | NA | NA | NA | NA |
| Albiglutide:Dapagliflozin_high_dosage | 0 | -0.01856673 | NA | -0.01856673 | NA | NA | NA | NA |
| Albiglutide:Dulaglutide | 0 | -0.203152141 | NA | -0.203152141 | NA | NA | NA | NA |
| Albiglutide:Efpeglenatide_high_dosage | 0 | -0.501102932 | NA | -0.501102932 | NA | NA | NA | NA |
| Albiglutide:Efpeglenatide_medium_dosage | 0 | -0.49963072 | NA | -0.49963072 | NA | NA | NA | NA |
| Albiglutide:Empagliflozin_high_dosage | 0 | 1.387604525 | NA | 1.387604525 | NA | NA | NA | NA |
| Albiglutide:Empagliflozin_low_dosage | 0 | 0.227884993 | NA | 0.227884993 | NA | NA | NA | NA |
| Albiglutide:Ertugliflozin_high_dosage | 0 | 0.094447335 | NA | 0.094447335 | NA | NA | NA | NA |
| Albiglutide:Ertugliflozin_low_dosage | 0 | 0.318143618 | NA | 0.318143618 | NA | NA | NA | NA |
| Albiglutide:Exenatide | 0 | 0.843811983 | NA | 0.843811983 | NA | NA | NA | NA |
| Albiglutide:Inject_semaglutide_high_dosage | 0 | 0.289712541 | NA | 0.289712541 | NA | NA | NA | NA |
| Albiglutide:Inject_semaglutide_low_dosage | 0 | 1.150511412 | NA | 1.150511412 | NA | NA | NA | NA |
| Albiglutide:Inject_semaglutide_medium_dosage | 0 | 1.297736098 | NA | 1.297736098 | NA | NA | NA | NA |
| Albiglutide:Liraglutide | 0 | 0.359754786 | NA | 0.359754786 | NA | NA | NA | NA |
| Albiglutide:Lixisenatide | 0 | 1.512986207 | NA | 1.512986207 | NA | NA | NA | NA |
| Albiglutide:Oral_semaglutide | 0 | 0.904726442 | NA | 0.904726442 | NA | NA | NA | NA |
| Albiglutide:Placebo_or_Control | 2 | 0.596034973 | 0.596034973 | NA | NA | NA | NA | NA |
| Albiglutide:Sotagliflozin | 0 | 0.435480895 | NA | 0.435480895 | NA | NA | NA | NA |

| | | | | | | | | |
|---|---|---|---|---|---|---|---|---|
| Albiglutide:Tirzepatide | 0 | 2.316391428 | NA | 2.316391428 | NA | NA | NA | NA |
| Bexagliflozin:Canagliflozin_high_dosage | 0 | 1.02325191 | NA | 1.02325191 | NA | NA | NA | NA |
| Bexagliflozin:Canagliflozin_low_dosage | 0 | 0.547347192 | NA | 0.547347192 | NA | NA | NA | NA |
| Bexagliflozin:Dapagliflozin_high_dosage | 0 | -0.20604813 | NA | -0.20604813 | NA | NA | NA | NA |
| Bexagliflozin:Dulaglutide | 0 | -0.390633542 | NA | -0.390633542 | NA | NA | NA | NA |
| Bexagliflozin:Efpeglenatide_high_dosage | 0 | -0.688584333 | NA | -0.688584333 | NA | NA | NA | NA |
| Bexagliflozin:Efpeglenatide_medium_dosage | 0 | -0.687112121 | NA | -0.687112121 | NA | NA | NA | NA |
| Bexagliflozin:Empagliflozin_high_dosage | 0 | 1.200123124 | NA | 1.200123124 | NA | NA | NA | NA |
| Bexagliflozin:Empagliflozin_low_dosage | 0 | 0.040403592 | NA | 0.040403592 | NA | NA | NA | NA |
| Bexagliflozin:Ertugliflozin_high_dosage | 0 | -0.093034066 | NA | -0.093034066 | NA | NA | NA | NA |
| Bexagliflozin:Ertugliflozin_low_dosage | 0 | 0.130662218 | NA | 0.130662218 | NA | NA | NA | NA |
| Bexagliflozin:Exenatide | 0 | 0.656330583 | NA | 0.656330583 | NA | NA | NA | NA |
| Bexagliflozin:Inject_semaglutide_high_dosage | 0 | 0.10223114 | NA | 0.10223114 | NA | NA | NA | NA |
| Bexagliflozin:Inject_semaglutide_low_dosage | 0 | 0.963030011 | NA | 0.963030011 | NA | NA | NA | NA |
| Bexagliflozin:Inject_semaglutide_medium_dosage | 0 | 1.110254697 | NA | 1.110254697 | NA | NA | NA | NA |
| Bexagliflozin:Liraglutide | 0 | 0.172273385 | NA | 0.172273385 | NA | NA | NA | NA |
| Bexagliflozin:Lixisenatide | 0 | 1.325504807 | NA | 1.325504807 | NA | NA | NA | NA |
| Bexagliflozin:Oral_semaglutide | 0 | 0.717245041 | NA | 0.717245041 | NA | NA | NA | NA |
| Bexagliflozin:Placebo_or_Control | 1 | 0.408553573 | 0.408553573 | NA | NA | NA | NA | NA |
| Bexagliflozin:Sotagliflozin | 0 | 0.247999494 | NA | 0.247999494 | NA | NA | NA | NA |
| Bexagliflozin:Tirzepatide | 0 | 2.128910027 | NA | 2.128910027 | NA | NA | NA | NA |
| Canagliflozin_high_dosage:Canagliflozin_low_dosage | 1 | -0.475904718 | -0.334390348 | -1.033707986 | 0.699317638 | -2.912249851 | 4.310885127 | 0.704306835 |

| | | | | | | | | |
|---|---|---|---|---|---|---|---|---|
| Canagliflozin_high_dosage:Dapagliflozin_high_dosage | 0 | -1.22930004 | NA | -1.22930004 | NA | NA | NA | NA |
| Canagliflozin_high_dosage:Dulaglutide | 0 | -1.413885452 | NA | -1.413885452 | NA | NA | NA | NA |
| Canagliflozin_high_dosage:Efpeglenatide_high_dosage | 0 | -1.711836243 | NA | -1.711836243 | NA | NA | NA | NA |
| Canagliflozin_high_dosage:Efpeglenatide_medium_dosage | 0 | -1.710364031 | NA | -1.710364031 | NA | NA | NA | NA |
| Canagliflozin_high_dosage:Empagliflozin_high_dosage | 0 | 0.176871214 | NA | 0.176871214 | NA | NA | NA | NA |
| Canagliflozin_high_dosage:Empagliflozin_low_dosage | 0 | -0.982848318 | NA | -0.982848318 | NA | NA | NA | NA |
| Canagliflozin_high_dosage:Ertugliflozin_high_dosage | 0 | -1.116285976 | NA | -1.116285976 | NA | NA | NA | NA |
| Canagliflozin_high_dosage:Ertugliflozin_low_dosage | 0 | -0.892589692 | NA | -0.892589692 | NA | NA | NA | NA |
| Canagliflozin_high_dosage:Exenatide | 0 | -0.366921328 | NA | -0.366921328 | NA | NA | NA | NA |
| Canagliflozin_high_dosage:Inject_semaglutide_high_dosage | 0 | -0.92102077 | NA | -0.92102077 | NA | NA | NA | NA |
| Canagliflozin_high_dosage:Inject_semaglutide_low_dosage | 0 | -0.060221899 | NA | -0.060221899 | NA | NA | NA | NA |
| Canagliflozin_high_dosage:Inject_semaglutide_medium_dosage | 0 | 0.087002787 | NA | 0.087002787 | NA | NA | NA | NA |
| Canagliflozin_high_dosage:Liraglutide | 0 | -0.850978525 | NA | -0.850978525 | NA | NA | NA | NA |
| Canagliflozin_high_dosage:Lixisenatide | 0 | 0.302252897 | NA | 0.302252897 | NA | NA | NA | NA |
| Canagliflozin_high_dosage:Oral_semaglutide | 0 | -0.306006869 | NA | -0.306006869 | NA | NA | NA | NA |
| Canagliflozin_high_dosage:Placebo_or_Control | 2 | -0.614698337 | -0.360808 | -1.558128736 | 1.197320736 | -2.662491578 | 5.05713305 | 0.543197847 |
| Canagliflozin_high_dosage:Sotagliflozin | 0 | -0.775252416 | NA | -0.775252416 | NA | NA | NA | NA |
| Canagliflozin_high_dosage:Tirzepatide | 0 | 1.105658117 | NA | 1.105658117 | NA | NA | NA | NA |
| Canagliflozin_low_dosage:Dapagliflozin_high_dosage | 0 | -0.753395322 | NA | -0.753395322 | NA | NA | NA | NA |
| Canagliflozin_low_dosage:Dulaglutide | 0 | -0.937980734 | NA | -0.937980734 | NA | NA | NA | NA |
| Canagliflozin_low_dosage:Efpeglenatide_high_dosage | 0 | -1.235931525 | NA | -1.235931525 | NA | NA | NA | NA |
| Canagliflozin_low_dosage:Efpeglenatide_medium_dosage | 0 | -1.234459313 | NA | -1.234459313 | NA | NA | NA | NA |

| | | | | | | | | |
|---|---|---|---|---|---|---|---|---|
| Canagliflozin_low_dosage:Empagliflozin_high_dosage | 0 | 0.652775932 | NA | 0.652775932 | NA | NA | NA | NA |
| Canagliflozin_low_dosage:Empagliflozin_low_dosage | 0 | -0.5069436 | NA | -0.5069436 | NA | NA | NA | NA |
| Canagliflozin_low_dosage:Ertugliflozin_high_dosage | 0 | -0.640381258 | NA | -0.640381258 | NA | NA | NA | NA |
| Canagliflozin_low_dosage:Ertugliflozin_low_dosage | 0 | -0.416684974 | NA | -0.416684974 | NA | NA | NA | NA |
| Canagliflozin_low_dosage:Exenatide | 0 | 0.10898339 | NA | 0.10898339 | NA | NA | NA | NA |
| Canagliflozin_low_dosage:Inject_semaglutide_high_dosage | 0 | -0.445116052 | NA | -0.445116052 | NA | NA | NA | NA |
| Canagliflozin_low_dosage:Inject_semaglutide_low_dosage | 0 | 0.415682819 | NA | 0.415682819 | NA | NA | NA | NA |
| Canagliflozin_low_dosage:Inject_semaglutide_medium_dosage | 0 | 0.562907505 | NA | 0.562907505 | NA | NA | NA | NA |
| Canagliflozin_low_dosage:Liraglutide | 0 | -0.375073807 | NA | -0.375073807 | NA | NA | NA | NA |
| Canagliflozin_low_dosage:Lixisenatide | 0 | 0.778157615 | NA | 0.778157615 | NA | NA | NA | NA |
| Canagliflozin_low_dosage:Oral_semaglutide | 0 | 0.169897849 | NA | 0.169897849 | NA | NA | NA | NA |
| Canagliflozin_low_dosage:Placebo_or_Control | 2 | -0.138793619 | 0.351143058 | -1.482530374 | 1.833673431 | -1.775880468 | 5.443227331 | 0.319409252 |
| Canagliflozin_low_dosage:Sotagliflozin | 0 | -0.299347698 | NA | -0.299347698 | NA | NA | NA | NA |
| Canagliflozin_low_dosage:Tirzepatide | 0 | 1.581562835 | NA | 1.581562835 | NA | NA | NA | NA |
| Dapagliflozin_high_dosage:Dulaglutide | 0 | -0.184585412 | NA | -0.184585412 | NA | NA | NA | NA |
| Dapagliflozin_high_dosage:Efpeglenatide_high_dosage | 0 | -0.482536203 | NA | -0.482536203 | NA | NA | NA | NA |
| Dapagliflozin_high_dosage:Efpeglenatide_medium_dosage | 0 | -0.48106399 | NA | -0.48106399 | NA | NA | NA | NA |
| Dapagliflozin_high_dosage:Empagliflozin_high_dosage | 0 | 1.406171254 | NA | 1.406171254 | NA | NA | NA | NA |
| Dapagliflozin_high_dosage:Empagliflozin_low_dosage | 0 | 0.246451722 | NA | 0.246451722 | NA | NA | NA | NA |
| Dapagliflozin_high_dosage:Ertugliflozin_high_dosage | 0 | 0.113014064 | NA | 0.113014064 | NA | NA | NA | NA |
| Dapagliflozin_high_dosage:Ertugliflozin_low_dosage | 0 | 0.336710348 | NA | 0.336710348 | NA | NA | NA | NA |
| Dapagliflozin_high_dosage:Exenatide | 0 | 0.862378713 | NA | 0.862378713 | NA | NA | NA | NA |

| | | | | | | | | |
|---|---|---|---|---|---|---|---|---|
| Dapagliflozin_high_dosage:Inject_semaglutide_high_dosage | 0 | 0.30827927 | NA | 0.30827927 | NA | NA | NA | NA |
| Dapagliflozin_high_dosage:Inject_semaglutide_low_dosage | 0 | 1.169078141 | NA | 1.169078141 | NA | NA | NA | NA |
| Dapagliflozin_high_dosage:Inject_semaglutide_medium_dosage | 0 | 1.316302827 | NA | 1.316302827 | NA | NA | NA | NA |
| Dapagliflozin_high_dosage:Liraglutide | 0 | 0.378321516 | NA | 0.378321516 | NA | NA | NA | NA |
| Dapagliflozin_high_dosage:Lixisenatide | 0 | 1.531552937 | NA | 1.531552937 | NA | NA | NA | NA |
| Dapagliflozin_high_dosage:Oral_semaglutide | 0 | 0.923293172 | NA | 0.923293172 | NA | NA | NA | NA |
| Dapagliflozin_high_dosage:Placebo_or_Control | 3 | 0.614601703 | 0.614601703 | NA | NA | NA | NA | NA |
| Dapagliflozin_high_dosage:Sotagliflozin | 0 | 0.454047624 | NA | 0.454047624 | NA | NA | NA | NA |
| Dapagliflozin_high_dosage:Tirzepatide | 0 | 2.334958158 | NA | 2.334958158 | NA | NA | NA | NA |
| Dulaglutide:Efpeglenatide_high_dosage | 0 | -0.297950791 | NA | -0.297950791 | NA | NA | NA | NA |
| Dulaglutide:Efpeglenatide_medium_dosage | 0 | -0.296478579 | NA | -0.296478579 | NA | NA | NA | NA |
| Dulaglutide:Empagliflozin_high_dosage | 0 | 1.590756666 | NA | 1.590756666 | NA | NA | NA | NA |
| Dulaglutide:Empagliflozin_low_dosage | 0 | 0.431037134 | NA | 0.431037134 | NA | NA | NA | NA |
| Dulaglutide:Ertugliflozin_high_dosage | 0 | 0.297599476 | NA | 0.297599476 | NA | NA | NA | NA |
| Dulaglutide:Ertugliflozin_low_dosage | 0 | 0.52129576 | NA | 0.52129576 | NA | NA | NA | NA |
| Dulaglutide:Exenatide | 0 | 1.046964125 | NA | 1.046964125 | NA | NA | NA | NA |
| Dulaglutide:Inject_semaglutide_high_dosage | 0 | 0.492864682 | NA | 0.492864682 | NA | NA | NA | NA |
| Dulaglutide:Inject_semaglutide_low_dosage | 0 | 1.353663553 | NA | 1.353663553 | NA | NA | NA | NA |
| Dulaglutide:Inject_semaglutide_medium_dosage | 0 | 1.500888239 | NA | 1.500888239 | NA | NA | NA | NA |
| Dulaglutide:Liraglutide | 0 | 0.562906927 | NA | 0.562906927 | NA | NA | NA | NA |
| Dulaglutide:Lixisenatide | 0 | 1.716138349 | NA | 1.716138349 | NA | NA | NA | NA |
| Dulaglutide:Oral_semaglutide | 0 | 1.107878583 | NA | 1.107878583 | NA | NA | NA | NA |

| | | | | | | | | |
|---|---|---|---|---|---|---|---|---|
| Dulaglutide:Placebo_or_Control | 2 | 0.799187115 | 0.799187115 | NA | NA | NA | NA | NA |
| Dulaglutide:Sotagliflozin | 0 | 0.638633036 | NA | 0.638633036 | NA | NA | NA | NA |
| Dulaglutide:Tirzepatide | 0 | 2.519543569 | NA | 2.519543569 | NA | NA | NA | NA |
| Efpeglenatide_high_dosage:Efpeglenatide_medium_dosage | 1 | 0.001472212 | 0.001472212 | NA | NA | NA | NA | NA |
| Efpeglenatide_high_dosage:Empagliflozin_high_dosage | 0 | 1.888707457 | NA | 1.888707457 | NA | NA | NA | NA |
| Efpeglenatide_high_dosage:Empagliflozin_low_dosage | 0 | 0.728987925 | NA | 0.728987925 | NA | NA | NA | NA |
| Efpeglenatide_high_dosage:Ertugliflozin_high_dosage | 0 | 0.595550267 | NA | 0.595550267 | NA | NA | NA | NA |
| Efpeglenatide_high_dosage:Ertugliflozin_low_dosage | 0 | 0.819246551 | NA | 0.819246551 | NA | NA | NA | NA |
| Efpeglenatide_high_dosage:Exenatide | 0 | 1.344914915 | NA | 1.344914915 | NA | NA | NA | NA |
| Efpeglenatide_high_dosage:Inject_semaglutide_high_dosage | 0 | 0.790815473 | NA | 0.790815473 | NA | NA | NA | NA |
| Efpeglenatide_high_dosage:Inject_semaglutide_low_dosage | 0 | 1.651614344 | NA | 1.651614344 | NA | NA | NA | NA |
| Efpeglenatide_high_dosage:Inject_semaglutide_medium_dosage | 0 | 1.79883903 | NA | 1.79883903 | NA | NA | NA | NA |
| Efpeglenatide_high_dosage:Liraglutide | 0 | 0.860857718 | NA | 0.860857718 | NA | NA | NA | NA |
| Efpeglenatide_high_dosage:Lixisenatide | 0 | 2.01408914 | NA | 2.01408914 | NA | NA | NA | NA |
| Efpeglenatide_high_dosage:Oral_semaglutide | 0 | 1.405829374 | NA | 1.405829374 | NA | NA | NA | NA |
| Efpeglenatide_high_dosage:Placebo_or_Control | 1 | 1.097137906 | 1.097137906 | NA | NA | NA | NA | NA |
| Efpeglenatide_high_dosage:Sotagliflozin | 0 | 0.936583827 | NA | 0.936583827 | NA | NA | NA | NA |
| Efpeglenatide_high_dosage:Tirzepatide | 0 | 2.81749436 | NA | 2.81749436 | NA | NA | NA | NA |
| Efpeglenatide_medium_dosage:Empagliflozin_high_dosage | 0 | 1.887235245 | NA | 1.887235245 | NA | NA | NA | NA |
| Efpeglenatide_medium_dosage:Empagliflozin_low_dosage | 0 | 0.727515713 | NA | 0.727515713 | NA | NA | NA | NA |
| Efpeglenatide_medium_dosage:Ertugliflozin_high_dosage | 0 | 0.594078055 | NA | 0.594078055 | NA | NA | NA | NA |
| Efpeglenatide_medium_dosage:Ertugliflozin_low_dosage | 0 | 0.817774339 | NA | 0.817774339 | NA | NA | NA | NA |

| | | | | | | | | |
|---|---|---|---|---|---|---|---|---|
| Efpeglenatide_medium_dosage:Exenatide | 0 | 1.343442703 | NA | 1.343442703 | NA | NA | NA | NA |
| Efpeglenatide_medium_dosage:Inject_semaglutide_high_dosage | 0 | 0.789343261 | NA | 0.789343261 | NA | NA | NA | NA |
| Efpeglenatide_medium_dosage:Inject_semaglutide_low_dosage | 0 | 1.650142132 | NA | 1.650142132 | NA | NA | NA | NA |
| Efpeglenatide_medium_dosage:Inject_semaglutide_medium_dosage | 0 | 1.797366818 | NA | 1.797366818 | NA | NA | NA | NA |
| Efpeglenatide_medium_dosage:Liraglutide | 0 | 0.859385506 | NA | 0.859385506 | NA | NA | NA | NA |
| Efpeglenatide_medium_dosage:Lixisenatide | 0 | 2.012616927 | NA | 2.012616927 | NA | NA | NA | NA |
| Efpeglenatide_medium_dosage:Oral_semaglutide | 0 | 1.404357162 | NA | 1.404357162 | NA | NA | NA | NA |
| Efpeglenatide_medium_dosage:Placebo_or_Control | 1 | 1.095665694 | 1.095665694 | NA | NA | NA | NA | NA |
| Efpeglenatide_medium_dosage:Sotagliflozin | 0 | 0.935111615 | NA | 0.935111615 | NA | NA | NA | NA |
| Efpeglenatide_medium_dosage:Tirzepatide | 0 | 2.816022148 | NA | 2.816022148 | NA | NA | NA | NA |
| Empagliflozin_high_dosage:Empagliflozin_low_dosage | 1 | -1.159719532 | -0.69229321 | -3.990419392 | 3.298126182 | -3.080018422 | 9.676270787 | 0.310824467 |
| Empagliflozin_high_dosage:Ertugliflozin_high_dosage | 0 | -1.29315719 | NA | -1.29315719 | NA | NA | NA | NA |
| Empagliflozin_high_dosage:Ertugliflozin_low_dosage | 0 | -1.069460906 | NA | -1.069460906 | NA | NA | NA | NA |
| Empagliflozin_high_dosage:Exenatide | 0 | -0.543792541 | NA | -0.543792541 | NA | NA | NA | NA |
| Empagliflozin_high_dosage:Inject_semaglutide_high_dosage | 0 | -1.097891984 | NA | -1.097891984 | NA | NA | NA | NA |
| Empagliflozin_high_dosage:Inject_semaglutide_low_dosage | 0 | -0.237093113 | NA | -0.237093113 | NA | NA | NA | NA |
| Empagliflozin_high_dosage:Inject_semaglutide_medium_dosage | 0 | -0.089868427 | NA | -0.089868427 | NA | NA | NA | NA |
| Empagliflozin_high_dosage:Liraglutide | 0 | -1.027849739 | NA | -1.027849739 | NA | NA | NA | NA |
| Empagliflozin_high_dosage:Lixisenatide | 0 | 0.125381683 | NA | 0.125381683 | NA | NA | NA | NA |
| Empagliflozin_high_dosage:Oral_semaglutide | 0 | -0.482878083 | NA | -0.482878083 | NA | NA | NA | NA |
| Empagliflozin_high_dosage:Placebo_or_Control | 1 | -0.791569551 | -1.10332221 | 3.292594015 | -4.395916224 | -12.89704317 | 4.105210718 | 0.310824467 |
| Empagliflozin_high_dosage:Sotagliflozin | 0 | -0.95212363 | NA | -0.95212363 | NA | NA | NA | NA |

| | | | | | | | | |
|---|---|---|---|---|---|---|---|---|
| Empagliflozin_high_dosage:Tirzepatide | 0 | 0.928786903 | NA | 0.928786903 | NA | NA | NA | NA |
| Empagliflozin_low_dosage:Ertugliflozin_high_dosage | 0 | -0.133437658 | NA | -0.133437658 | NA | NA | NA | NA |
| Empagliflozin_low_dosage:Ertugliflozin_low_dosage | 0 | 0.090258626 | NA | 0.090258626 | NA | NA | NA | NA |
| Empagliflozin_low_dosage:Exenatide | 0 | 0.61592699 | NA | 0.61592699 | NA | NA | NA | NA |
| Empagliflozin_low_dosage:Inject_semaglutide_high_dosage | 0 | 0.061827548 | NA | 0.061827548 | NA | NA | NA | NA |
| Empagliflozin_low_dosage:Inject_semaglutide_low_dosage | 0 | 0.922626419 | NA | 0.922626419 | NA | NA | NA | NA |
| Empagliflozin_low_dosage:Inject_semaglutide_medium_dosage | 0 | 1.069851105 | NA | 1.069851105 | NA | NA | NA | NA |
| Empagliflozin_low_dosage:Liraglutide | 0 | 0.131869793 | NA | 0.131869793 | NA | NA | NA | NA |
| Empagliflozin_low_dosage:Lixisenatide | 0 | 1.285101215 | NA | 1.285101215 | NA | NA | NA | NA |
| Empagliflozin_low_dosage:Oral_semaglutide | 0 | 0.676841449 | NA | 0.676841449 | NA | NA | NA | NA |
| Empagliflozin_low_dosage:Placebo_or_Control | 4 | 0.368149981 | 0.368149981 | NA | NA | NA | NA | NA |
| Empagliflozin_low_dosage:Sotagliflozin | 0 | 0.207595902 | NA | 0.207595902 | NA | NA | NA | NA |
| Empagliflozin_low_dosage:Tirzepatide | 0 | 2.088506435 | NA | 2.088506435 | NA | NA | NA | NA |
| Ertugliflozin_high_dosage:Ertugliflozin_low_dosage | 2 | 0.223696284 | 0.176697429 | 1.622225494 | -1.445528065 | -8.505715127 | 5.614658996 | 0.688206575 |
| Ertugliflozin_high_dosage:Exenatide | 0 | 0.749364649 | NA | 0.749364649 | NA | NA | NA | NA |
| Ertugliflozin_high_dosage:Inject_semaglutide_high_dosage | 0 | 0.195265206 | NA | 0.195265206 | NA | NA | NA | NA |
| Ertugliflozin_high_dosage:Inject_semaglutide_low_dosage | 0 | 1.056064077 | NA | 1.056064077 | NA | NA | NA | NA |
| Ertugliflozin_high_dosage:Inject_semaglutide_medium_dosage | 0 | 1.203288763 | NA | 1.203288763 | NA | NA | NA | NA |
| Ertugliflozin_high_dosage:Liraglutide | 0 | 0.265307451 | NA | 0.265307451 | NA | NA | NA | NA |
| Ertugliflozin_high_dosage:Lixisenatide | 0 | 1.418538873 | NA | 1.418538873 | NA | NA | NA | NA |
| Ertugliflozin_high_dosage:Oral_semaglutide | 0 | 0.810279107 | NA | 0.810279107 | NA | NA | NA | NA |
| Ertugliflozin_high_dosage:Placebo_or_Control | 2 | 0.501587639 | 0.432505637 | 2.910692852 | -2.478187215 | -10.60733302 | 5.650958586 | 0.550174176 |

| | | | | | | | | |
|---|---|---|---|---|---|---|---|---|
| Ertugliflozin_high_dosage:Sotagliflozin | 0 | 0.34103356 | NA | 0.34103356 | NA | NA | NA | NA |
| Ertugliflozin_high_dosage:Tirzepatide | 0 | 2.221944093 | NA | 2.221944093 | NA | NA | NA | NA |
| Ertugliflozin_low_dosage:Exenatide | 0 | 0.525668365 | NA | 0.525668365 | NA | NA | NA | NA |
| Ertugliflozin_low_dosage:Inject_semaglutide_high_dosage | 0 | -0.028431078 | NA | -0.028431078 | NA | NA | NA | NA |
| Ertugliflozin_low_dosage:Inject_semaglutide_low_dosage | 0 | 0.832367793 | NA | 0.832367793 | NA | NA | NA | NA |
| Ertugliflozin_low_dosage:Inject_semaglutide_medium_dosage | 0 | 0.979592479 | NA | 0.979592479 | NA | NA | NA | NA |
| Ertugliflozin_low_dosage:Liraglutide | 0 | 0.041611167 | NA | 0.041611167 | NA | NA | NA | NA |
| Ertugliflozin_low_dosage:Lixisenatide | 0 | 1.194842589 | NA | 1.194842589 | NA | NA | NA | NA |
| Ertugliflozin_low_dosage:Oral_semaglutide | 0 | 0.586582823 | NA | 0.586582823 | NA | NA | NA | NA |
| Ertugliflozin_low_dosage:Placebo_or_Control | 1 | 0.277891355 | 0.287682072 | 0.18944794 | 0.098234132 | -4.646389683 | 4.842857947 | 0.967630976 |
| Ertugliflozin_low_dosage:Sotagliflozin | 0 | 0.117337276 | NA | 0.117337276 | NA | NA | NA | NA |
| Ertugliflozin_low_dosage:Tirzepatide | 0 | 1.998247809 | NA | 1.998247809 | NA | NA | NA | NA |
| Exenatide:Inject_semaglutide_high_dosage | 0 | -0.554099442 | NA | -0.554099442 | NA | NA | NA | NA |
| Exenatide:Inject_semaglutide_low_dosage | 0 | 0.306699429 | NA | 0.306699429 | NA | NA | NA | NA |
| Exenatide:Inject_semaglutide_medium_dosage | 0 | 0.453924114 | NA | 0.453924114 | NA | NA | NA | NA |
| Exenatide:Liraglutide | 0 | -0.484057197 | NA | -0.484057197 | NA | NA | NA | NA |
| Exenatide:Lixisenatide | 0 | 0.669174224 | NA | 0.669174224 | NA | NA | NA | NA |
| Exenatide:Oral_semaglutide | 0 | 0.060914459 | NA | 0.060914459 | NA | NA | NA | NA |
| Exenatide:Placebo_or_Control | 1 | -0.24777701 | -0.24777701 | NA | NA | NA | NA | NA |
| Exenatide:Sotagliflozin | 0 | -0.408331089 | NA | -0.408331089 | NA | NA | NA | NA |
| Exenatide:Tirzepatide | 0 | 1.472579445 | NA | 1.472579445 | NA | NA | NA | NA |
| Inject_semaglutide_high_dosage:Inject_semaglutide_low_dosage | 0 | 0.860798871 | NA | 0.860798871 | NA | NA | NA | NA |

| | | | | | | | | |
|---|---|---|---|---|---|---|---|---|
| Inject_semaglutide_high_dosage:Inject_semaglutide_medium_dosage | 1 | 1.008023557 | 1.098612289 | 0.97817013 | 0.120442159 | -3.573489684 | 3.814374001 | 0.949045531 |
| Inject_semaglutide_high_dosage:Liraglutide | 0 | 0.070042245 | NA | 0.070042245 | NA | NA | NA | NA |
| Inject_semaglutide_high_dosage:Lixisenatide | 0 | 1.223273667 | NA | 1.223273667 | NA | NA | NA | NA |
| Inject_semaglutide_high_dosage:Oral_semaglutide | 0 | 0.615013901 | NA | 0.615013901 | NA | NA | NA | NA |
| Inject_semaglutide_high_dosage:Placebo_or_Control | 3 | 0.306322433 | 0.299787256 | 0.420229414 | -0.120442159 | -3.814374001 | 3.573489684 | 0.949045531 |
| Inject_semaglutide_high_dosage:Sotagliflozin | 0 | 0.145768354 | NA | 0.145768354 | NA | NA | NA | NA |
| Inject_semaglutide_high_dosage:Tirzepatide | 0 | 2.026678887 | NA | 2.026678887 | NA | NA | NA | NA |
| Inject_semaglutide_low_dosage:Inject_semaglutide_medium_dosage | 2 | 0.147224686 | 0.472892936 | -1.230319618 | 1.703212554 | -3.093367999 | 6.499793107 | 0.486452958 |
| Inject_semaglutide_low_dosage:Liraglutide | 0 | -0.790756626 | NA | -0.790756626 | NA | NA | NA | NA |
| Inject_semaglutide_low_dosage:Lixisenatide | 0 | 0.362474796 | NA | 0.362474796 | NA | NA | NA | NA |
| Inject_semaglutide_low_dosage:Oral_semaglutide | 0 | -0.24578497 | NA | -0.24578497 | NA | NA | NA | NA |
| Inject_semaglutide_low_dosage:Placebo_or_Control | 2 | -0.554476438 | -0.693710386 | 2.797394006 | -3.491104392 | -11.39493599 | 4.412727205 | 0.386648325 |
| Inject_semaglutide_low_dosage:Sotagliflozin | 0 | -0.715030517 | NA | -0.715030517 | NA | NA | NA | NA |
| Inject_semaglutide_low_dosage:Tirzepatide | 0 | 1.165880016 | NA | 1.165880016 | NA | NA | NA | NA |
| Inject_semaglutide_medium_dosage:Liraglutide | 0 | -0.937981312 | NA | -0.937981312 | NA | NA | NA | NA |
| Inject_semaglutide_medium_dosage:Lixisenatide | 0 | 0.21525011 | NA | 0.21525011 | NA | NA | NA | NA |
| Inject_semaglutide_medium_dosage:Oral_semaglutide | 0 | -0.393009656 | NA | -0.393009656 | NA | NA | NA | NA |
| Inject_semaglutide_medium_dosage:Placebo_or_Control | 2 | -0.701701124 | -1.045069515 | -0.06405328 | -0.981016236 | -4.04105542 | 2.079022948 | 0.529778778 |
| Inject_semaglutide_medium_dosage:Sotagliflozin | 0 | -0.862255203 | NA | -0.862255203 | NA | NA | NA | NA |
| Inject_semaglutide_medium_dosage:Tirzepatide | 1 | 1.01865533 | 2.200066498 | 0.333932731 | 1.866133767 | -2.158577843 | 5.890845376 | 0.36346921 |
| Liraglutide:Lixisenatide | 0 | 1.153231421 | NA | 1.153231421 | NA | NA | NA | NA |
| Liraglutide:Oral_semaglutide | 0 | 0.544971656 | NA | 0.544971656 | NA | NA | NA | NA |

| | | | | | | | | |
|---|---|---|---|---|---|---|---|---|
| Liraglutide:Placebo_or_Control | 3 | 0.236280188 | 0.236280188 | NA | NA | NA | NA | NA |
| Liraglutide:Sotagliflozin | 0 | 0.075726109 | NA | 0.075726109 | NA | NA | NA | NA |
| Liraglutide:Tirzepatide | 0 | 1.956636642 | NA | 1.956636642 | NA | NA | NA | NA |
| Lixisenatide:Oral_semaglutide | 0 | -0.608259765 | NA | -0.608259765 | NA | NA | NA | NA |
| Lixisenatide:Placebo_or_Control | 1 | -0.916951234 | -0.916951234 | NA | NA | NA | NA | NA |
| Lixisenatide:Sotagliflozin | 0 | -1.077505313 | NA | -1.077505313 | NA | NA | NA | NA |
| Lixisenatide:Tirzepatide | 0 | 0.80340522 | NA | 0.80340522 | NA | NA | NA | NA |
| Oral_semaglutide:Placebo_or_Control | 2 | -0.308691468 | -0.308691468 | NA | NA | NA | NA | NA |
| Oral_semaglutide:Sotagliflozin | 0 | -0.469245547 | NA | -0.469245547 | NA | NA | NA | NA |
| Oral_semaglutide:Tirzepatide | 0 | 1.411664986 | NA | 1.411664986 | NA | NA | NA | NA |
| Sotagliflozin:Placebo_or_Control | 4 | 0.160554079 | 0.160554079 | NA | NA | NA | NA | NA |
| Tirzepatide:Placebo_or_Control | 3 | -1.720356454 | -1.326345037 | -3.192478804 | 1.866133767 | -2.158577843 | 5.890845376 | 0.36346921 |
| Sotagliflozin:Tirzepatide | 0 | 1.880910533 | NA | 1.880910533 | NA | NA | NA | NA |

## Table S7C: inconsistency within the network meta-analysis of primary outcome: subgroup of leukemia

| Comparison | No.Studies | NMA | Direct | Indirect | Difference | Diff_95CI_lower | Diff_95CI_upper | *p* value |
|---|---|---|---|---|---|---|---|---|
| Albiglutide:Canagliflozin_high_dosage | 0 | 1.223513314 | NA | 1.223513314 | NA | NA | NA | NA |
| Albiglutide:Canagliflozin_low_dosage | 0 | 0.842125983 | NA | 0.842125983 | NA | NA | NA | NA |
| Albiglutide:Dapagliflozin_high_dosage | 0 | 0.144456124 | NA | 0.144456124 | NA | NA | NA | NA |
| Albiglutide:Dapagliflozin_low_dosage | 0 | 1.334968749 | NA | 1.334968749 | NA | NA | NA | NA |
| Albiglutide:Dapagliflozin_medium_dosage | 0 | 1.359091903 | NA | 1.359091903 | NA | NA | NA | NA |
| Albiglutide:Dulaglutide | 0 | -0.294178075 | NA | -0.294178075 | NA | NA | NA | NA |
| Albiglutide:Efpeglenatide_high_dosage | 0 | 0.219869124 | NA | 0.219869124 | NA | NA | NA | NA |
| Albiglutide:Efpeglenatide_medium_dosage | 0 | -0.878007329 | NA | -0.878007329 | NA | NA | NA | NA |
| Albiglutide:Empagliflozin_high_dosage | 0 | 0.212145468 | NA | 0.212145468 | NA | NA | NA | NA |
| Albiglutide:Empagliflozin_low_dosage | 0 | -0.748590905 | NA | -0.748590905 | NA | NA | NA | NA |
| Albiglutide:Ertugliflozin_high_dosage | 0 | -0.880953925 | NA | -0.880953925 | NA | NA | NA | NA |
| Albiglutide:Ertugliflozin_low_dosage | 0 | -0.475488816 | NA | -0.475488816 | NA | NA | NA | NA |
| Albiglutide:Exenatide | 0 | -0.105013921 | NA | -0.105013921 | NA | NA | NA | NA |
| Albiglutide:Inject_semaglutide_high_dosage | 0 | 0.334534808 | NA | 0.334534808 | NA | NA | NA | NA |
| Albiglutide:Inject_semaglutide_low_dosage | 0 | 1.558340002 | NA | 1.558340002 | NA | NA | NA | NA |
| Albiglutide:Inject_semaglutide_medium_dosage | 0 | 0.649551352 | NA | 0.649551352 | NA | NA | NA | NA |
| Albiglutide:Liraglutide | 0 | 0.696148179 | NA | 0.696148179 | NA | NA | NA | NA |
| Albiglutide:Lixisenatide | 0 | 0.894184356 | NA | 0.894184356 | NA | NA | NA | NA |
| Albiglutide:Oral_semaglutide | 0 | 1.316899188 | NA | 1.316899188 | NA | NA | NA | NA |
| Albiglutide:Placebo_or_Control | 3 | 0.217658364 | 0.217658364 | NA | NA | NA | NA | NA |

| | | | | | | | | |
|---|---|---|---|---|---|---|---|---|
| Albiglutide:Sotagliflozin | 0 | 0.21860417 | NA | 0.21860417 | NA | NA | NA | NA |
| Albiglutide:Tirzepatide | 0 | 2.000635342 | NA | 2.000635342 | NA | NA | NA | NA |
| Canagliflozin_high_dosage:Canagliflozin_low_dosage | 1 | -0.381387331 | 0.002773927 | -1.022025807 | 1.024799734 | -3.503929382 | 5.553528851 | 0.657391471 |
| Canagliflozin_high_dosage:Dapagliflozin_high_dosage | 0 | -1.07905719 | NA | -1.07905719 | NA | NA | NA | NA |
| Canagliflozin_high_dosage:Dapagliflozin_low_dosage | 0 | 0.111455435 | NA | 0.111455435 | NA | NA | NA | NA |
| Canagliflozin_high_dosage:Dapagliflozin_medium_dosage | 0 | 0.13557859 | NA | 0.13557859 | NA | NA | NA | NA |
| Canagliflozin_high_dosage:Dulaglutide | 0 | -1.517691389 | NA | -1.517691389 | NA | NA | NA | NA |
| Canagliflozin_high_dosage:Efpeglenatide_high_dosage | 0 | -1.00364419 | NA | -1.00364419 | NA | NA | NA | NA |
| Canagliflozin_high_dosage:Efpeglenatide_medium_dosage | 0 | -2.101520643 | NA | -2.101520643 | NA | NA | NA | NA |
| Canagliflozin_high_dosage:Empagliflozin_high_dosage | 0 | -1.011367845 | NA | -1.011367845 | NA | NA | NA | NA |
| Canagliflozin_high_dosage:Empagliflozin_low_dosage | 0 | -1.972104219 | NA | -1.972104219 | NA | NA | NA | NA |
| Canagliflozin_high_dosage:Ertugliflozin_high_dosage | 0 | -2.104467238 | NA | -2.104467238 | NA | NA | NA | NA |
| Canagliflozin_high_dosage:Ertugliflozin_low_dosage | 0 | -1.69900213 | NA | -1.69900213 | NA | NA | NA | NA |
| Canagliflozin_high_dosage:Exenatide | 0 | -1.328527235 | NA | -1.328527235 | NA | NA | NA | NA |
| Canagliflozin_high_dosage:Inject_semaglutide_high_dosage | 0 | -0.888978506 | NA | -0.888978506 | NA | NA | NA | NA |
| Canagliflozin_high_dosage:Inject_semaglutide_low_dosage | 0 | 0.334826688 | NA | 0.334826688 | NA | NA | NA | NA |
| Canagliflozin_high_dosage:Inject_semaglutide_medium_dosage | 0 | -0.573961962 | NA | -0.573961962 | NA | NA | NA | NA |
| Canagliflozin_high_dosage:Liraglutide | 0 | -0.527365135 | NA | -0.527365135 | NA | NA | NA | NA |
| Canagliflozin_high_dosage:Lixisenatide | 0 | -0.329328957 | NA | -0.329328957 | NA | NA | NA | NA |
| Canagliflozin_high_dosage:Oral_semaglutide | 0 | 0.093385875 | NA | 0.093385875 | NA | NA | NA | NA |
| Canagliflozin_high_dosage:Placebo_or_Control | 2 | -1.00585495 | -1.046530259 | -0.023925797 | -1.022604462 | -10.46762973 | 8.422420804 | 0.831948235 |
| Canagliflozin_high_dosage:Sotagliflozin | 0 | -1.004909144 | NA | -1.004909144 | NA | NA | NA | NA |

| | | | | | | | | |
|---|---|---|---|---|---|---|---|---|
| Canagliflozin_high_dosage:Tirzepatide | 0 | 0.777122028 | NA | 0.777122028 | NA | NA | NA | NA |
| Canagliflozin_low_dosage:Dapagliflozin_high_dosage | 0 | -0.697669859 | NA | -0.697669859 | NA | NA | NA | NA |
| Canagliflozin_low_dosage:Dapagliflozin_low_dosage | 0 | 0.492842766 | NA | 0.492842766 | NA | NA | NA | NA |
| Canagliflozin_low_dosage:Dapagliflozin_medium_dosage | 0 | 0.51696592 | NA | 0.51696592 | NA | NA | NA | NA |
| Canagliflozin_low_dosage:Dulaglutide | 0 | -1.136304058 | NA | -1.136304058 | NA | NA | NA | NA |
| Canagliflozin_low_dosage:Efpeglenatide_high_dosage | 0 | -0.622256859 | NA | -0.622256859 | NA | NA | NA | NA |
| Canagliflozin_low_dosage:Efpeglenatide_medium_dosage | 0 | -1.720133312 | NA | -1.720133312 | NA | NA | NA | NA |
| Canagliflozin_low_dosage:Empagliflozin_high_dosage | 0 | -0.629980515 | NA | -0.629980515 | NA | NA | NA | NA |
| Canagliflozin_low_dosage:Empagliflozin_low_dosage | 0 | -1.590716888 | NA | -1.590716888 | NA | NA | NA | NA |
| Canagliflozin_low_dosage:Ertugliflozin_high_dosage | 0 | -1.723079908 | NA | -1.723079908 | NA | NA | NA | NA |
| Canagliflozin_low_dosage:Ertugliflozin_low_dosage | 0 | -1.317614799 | NA | -1.317614799 | NA | NA | NA | NA |
| Canagliflozin_low_dosage:Exenatide | 0 | -0.947139904 | NA | -0.947139904 | NA | NA | NA | NA |
| Canagliflozin_low_dosage:Inject_semaglutide_high_dosage | 0 | -0.507591176 | NA | -0.507591176 | NA | NA | NA | NA |
| Canagliflozin_low_dosage:Inject_semaglutide_low_dosage | 0 | 0.716214019 | NA | 0.716214019 | NA | NA | NA | NA |
| Canagliflozin_low_dosage:Inject_semaglutide_medium_dosage | 0 | -0.192574631 | NA | -0.192574631 | NA | NA | NA | NA |
| Canagliflozin_low_dosage:Liraglutide | 0 | -0.145977804 | NA | -0.145977804 | NA | NA | NA | NA |
| Canagliflozin_low_dosage:Lixisenatide | 0 | 0.052058373 | NA | 0.052058373 | NA | NA | NA | NA |
| Canagliflozin_low_dosage:Oral_semaglutide | 0 | 0.474773205 | NA | 0.474773205 | NA | NA | NA | NA |
| Canagliflozin_low_dosage:Placebo_or_Control | 3 | -0.624467619 | -0.569893819 | -3.445227555 | 2.875333736 | -8.592463582 | 14.34313105 | 0.623126609 |
| Canagliflozin_low_dosage:Sotagliflozin | 0 | -0.623521813 | NA | -0.623521813 | NA | NA | NA | NA |
| Canagliflozin_low_dosage:Tirzepatide | 0 | 1.158509359 | NA | 1.158509359 | NA | NA | NA | NA |
| Dapagliflozin_high_dosage:Dapagliflozin_low_dosage | 0 | 1.190512625 | NA | 1.190512625 | NA | NA | NA | NA |

| | | | | | | | | |
|---|---|---|---|---|---|---|---|---|
| Dapagliflozin_high_dosage:Dapagliflozin_medium_dosage | 0 | 1.21463578 | NA | 1.21463578 | NA | NA | NA | NA |
| Dapagliflozin_high_dosage:Dulaglutide | 0 | -0.438634199 | NA | -0.438634199 | NA | NA | NA | NA |
| Dapagliflozin_high_dosage:Efpeglenatide_high_dosage | 0 | 0.075413 | NA | 0.075413 | NA | NA | NA | NA |
| Dapagliflozin_high_dosage:Efpeglenatide_medium_dosage | 0 | -1.022463453 | NA | -1.022463453 | NA | NA | NA | NA |
| Dapagliflozin_high_dosage:Empagliflozin_high_dosage | 0 | 0.067689345 | NA | 0.067689345 | NA | NA | NA | NA |
| Dapagliflozin_high_dosage:Empagliflozin_low_dosage | 0 | -0.893047029 | NA | -0.893047029 | NA | NA | NA | NA |
| Dapagliflozin_high_dosage:Ertugliflozin_high_dosage | 0 | -1.025410048 | NA | -1.025410048 | NA | NA | NA | NA |
| Dapagliflozin_high_dosage:Ertugliflozin_low_dosage | 0 | -0.61994494 | NA | -0.61994494 | NA | NA | NA | NA |
| Dapagliflozin_high_dosage:Exenatide | 0 | -0.249470045 | NA | -0.249470045 | NA | NA | NA | NA |
| Dapagliflozin_high_dosage:Inject_semaglutide_high_dosage | 0 | 0.190078684 | NA | 0.190078684 | NA | NA | NA | NA |
| Dapagliflozin_high_dosage:Inject_semaglutide_low_dosage | 0 | 1.413883878 | NA | 1.413883878 | NA | NA | NA | NA |
| Dapagliflozin_high_dosage:Inject_semaglutide_medium_dosage | 0 | 0.505095228 | NA | 0.505095228 | NA | NA | NA | NA |
| Dapagliflozin_high_dosage:Liraglutide | 0 | 0.551692055 | NA | 0.551692055 | NA | NA | NA | NA |
| Dapagliflozin_high_dosage:Lixisenatide | 0 | 0.749728233 | NA | 0.749728233 | NA | NA | NA | NA |
| Dapagliflozin_high_dosage:Oral_semaglutide | 0 | 1.172443065 | NA | 1.172443065 | NA | NA | NA | NA |
| Dapagliflozin_high_dosage:Placebo_or_Control | 6 | 0.07320224 | 0.07320224 | NA | NA | NA | NA | NA |
| Dapagliflozin_high_dosage:Sotagliflozin | 0 | 0.074148046 | NA | 0.074148046 | NA | NA | NA | NA |
| Dapagliflozin_high_dosage:Tirzepatide | 0 | 1.856179218 | NA | 1.856179218 | NA | NA | NA | NA |
| Dapagliflozin_low_dosage:Dapagliflozin_medium_dosage | 0 | 0.024123155 | NA | 0.024123155 | NA | NA | NA | NA |
| Dapagliflozin_low_dosage:Dulaglutide | 0 | -1.629146824 | NA | -1.629146824 | NA | NA | NA | NA |
| Dapagliflozin_low_dosage:Efpeglenatide_high_dosage | 0 | -1.115099625 | NA | -1.115099625 | NA | NA | NA | NA |
| Dapagliflozin_low_dosage:Efpeglenatide_medium_dosage | 0 | -2.212976078 | NA | -2.212976078 | NA | NA | NA | NA |

| | | | | | | | | |
|---|---|---|---|---|---|---|---|---|
| Dapagliflozin_low_dosage:Empagliflozin_high_dosage | 0 | -1.12282328 | NA | -1.12282328 | NA | NA | NA | NA |
| Dapagliflozin_low_dosage:Empagliflozin_low_dosage | 0 | -2.083559654 | NA | -2.083559654 | NA | NA | NA | NA |
| Dapagliflozin_low_dosage:Ertugliflozin_high_dosage | 0 | -2.215922673 | NA | -2.215922673 | NA | NA | NA | NA |
| Dapagliflozin_low_dosage:Ertugliflozin_low_dosage | 0 | -1.810457565 | NA | -1.810457565 | NA | NA | NA | NA |
| Dapagliflozin_low_dosage:Exenatide | 0 | -1.43998267 | NA | -1.43998267 | NA | NA | NA | NA |
| Dapagliflozin_low_dosage:Inject_semaglutide_high_dosage | 0 | -1.000433941 | NA | -1.000433941 | NA | NA | NA | NA |
| Dapagliflozin_low_dosage:Inject_semaglutide_low_dosage | 0 | 0.223371253 | NA | 0.223371253 | NA | NA | NA | NA |
| Dapagliflozin_low_dosage:Inject_semaglutide_medium_dosage | 0 | -0.685417397 | NA | -0.685417397 | NA | NA | NA | NA |
| Dapagliflozin_low_dosage:Liraglutide | 0 | -0.63882057 | NA | -0.63882057 | NA | NA | NA | NA |
| Dapagliflozin_low_dosage:Lixisenatide | 0 | -0.440784392 | NA | -0.440784392 | NA | NA | NA | NA |
| Dapagliflozin_low_dosage:Oral_semaglutide | 0 | -0.01806956 | NA | -0.01806956 | NA | NA | NA | NA |
| Dapagliflozin_low_dosage:Placebo_or_Control | 2 | -1.117310385 | -1.117310385 | NA | NA | NA | NA | NA |
| Dapagliflozin_low_dosage:Sotagliflozin | 0 | -1.116364579 | NA | -1.116364579 | NA | NA | NA | NA |
| Dapagliflozin_low_dosage:Tirzepatide | 0 | 0.665666593 | NA | 0.665666593 | NA | NA | NA | NA |
| Dapagliflozin_medium_dosage:Dulaglutide | 0 | -1.653269979 | NA | -1.653269979 | NA | NA | NA | NA |
| Dapagliflozin_medium_dosage:Efpeglenatide_high_dosage | 0 | -1.139222779 | NA | -1.139222779 | NA | NA | NA | NA |
| Dapagliflozin_medium_dosage:Efpeglenatide_medium_dosage | 0 | -2.237099233 | NA | -2.237099233 | NA | NA | NA | NA |
| Dapagliflozin_medium_dosage:Empagliflozin_high_dosage | 0 | -1.146946435 | NA | -1.146946435 | NA | NA | NA | NA |
| Dapagliflozin_medium_dosage:Empagliflozin_low_dosage | 0 | -2.107682808 | NA | -2.107682808 | NA | NA | NA | NA |
| Dapagliflozin_medium_dosage:Ertugliflozin_high_dosage | 0 | -2.240045828 | NA | -2.240045828 | NA | NA | NA | NA |
| Dapagliflozin_medium_dosage:Ertugliflozin_low_dosage | 0 | -1.83458072 | NA | -1.83458072 | NA | NA | NA | NA |
| Dapagliflozin_medium_dosage:Exenatide | 0 | -1.464105825 | NA | -1.464105825 | NA | NA | NA | NA |

| | | | | | | | | |
|---|---|---|---|---|---|---|---|---|
| Dapagliflozin_medium_dosage:Inject_semaglutide_high_dosage | 0 | -1.024557096 | NA | -1.024557096 | NA | NA | NA | NA |
| Dapagliflozin_medium_dosage:Inject_semaglutide_low_dosage | 0 | 0.199248099 | NA | 0.199248099 | NA | NA | NA | NA |
| Dapagliflozin_medium_dosage:Inject_semaglutide_medium_dosage | 0 | -0.709540552 | NA | -0.709540552 | NA | NA | NA | NA |
| Dapagliflozin_medium_dosage:Liraglutide | 0 | -0.662943725 | NA | -0.662943725 | NA | NA | NA | NA |
| Dapagliflozin_medium_dosage:Lixisenatide | 0 | -0.464907547 | NA | -0.464907547 | NA | NA | NA | NA |
| Dapagliflozin_medium_dosage:Oral_semaglutide | 0 | -0.042192715 | NA | -0.042192715 | NA | NA | NA | NA |
| Dapagliflozin_medium_dosage:Placebo_or_Control | 2 | -1.141433539 | -1.141433539 | NA | NA | NA | NA | NA |
| Dapagliflozin_medium_dosage:Sotagliflozin | 0 | -1.140487734 | NA | -1.140487734 | NA | NA | NA | NA |
| Dapagliflozin_medium_dosage:Tirzepatide | 0 | 0.641543438 | NA | 0.641543438 | NA | NA | NA | NA |
| Dulaglutide:Efpeglenatide_high_dosage | 0 | 0.5140472 | NA | 0.5140472 | NA | NA | NA | NA |
| Dulaglutide:Efpeglenatide_medium_dosage | 0 | -0.583829254 | NA | -0.583829254 | NA | NA | NA | NA |
| Dulaglutide:Empagliflozin_high_dosage | 0 | 0.506323544 | NA | 0.506323544 | NA | NA | NA | NA |
| Dulaglutide:Empagliflozin_low_dosage | 0 | -0.45441283 | NA | -0.45441283 | NA | NA | NA | NA |
| Dulaglutide:Ertugliflozin_high_dosage | 0 | -0.586775849 | NA | -0.586775849 | NA | NA | NA | NA |
| Dulaglutide:Ertugliflozin_low_dosage | 0 | -0.181310741 | NA | -0.181310741 | NA | NA | NA | NA |
| Dulaglutide:Exenatide | 0 | 0.189164154 | NA | 0.189164154 | NA | NA | NA | NA |
| Dulaglutide:Inject_semaglutide_high_dosage | 0 | 0.628712883 | NA | 0.628712883 | NA | NA | NA | NA |
| Dulaglutide:Inject_semaglutide_low_dosage | 0 | 1.852518078 | NA | 1.852518078 | NA | NA | NA | NA |
| Dulaglutide:Inject_semaglutide_medium_dosage | 0 | 0.943729427 | NA | 0.943729427 | NA | NA | NA | NA |
| Dulaglutide:Liraglutide | 0 | 0.990326254 | NA | 0.990326254 | NA | NA | NA | NA |
| Dulaglutide:Lixisenatide | 0 | 1.188362432 | NA | 1.188362432 | NA | NA | NA | NA |
| Dulaglutide:Oral_semaglutide | 0 | 1.611077264 | NA | 1.611077264 | NA | NA | NA | NA |

| | | | | | | | | |
|---|---|---|---|---|---|---|---|---|
| Dulaglutide:Placebo_or_Control | 1 | 0.51183644 | 0.51183644 | NA | NA | NA | NA | NA |
| Dulaglutide:Sotagliflozin | 0 | 0.512782245 | NA | 0.512782245 | NA | NA | NA | NA |
| Dulaglutide:Tirzepatide | 0 | 2.294813417 | NA | 2.294813417 | NA | NA | NA | NA |
| Efpeglenatide_high_dosage:Efpeglenatide_medium_dosage | 1 | -1.097876453 | -1.097876453 | NA | NA | NA | NA | NA |
| Efpeglenatide_high_dosage:Empagliflozin_high_dosage | 0 | -0.007723656 | NA | -0.007723656 | NA | NA | NA | NA |
| Efpeglenatide_high_dosage:Empagliflozin_low_dosage | 0 | -0.968460029 | NA | -0.968460029 | NA | NA | NA | NA |
| Efpeglenatide_high_dosage:Ertugliflozin_high_dosage | 0 | -1.100823049 | NA | -1.100823049 | NA | NA | NA | NA |
| Efpeglenatide_high_dosage:Ertugliflozin_low_dosage | 0 | -0.69535794 | NA | -0.69535794 | NA | NA | NA | NA |
| Efpeglenatide_high_dosage:Exenatide | 0 | -0.324883045 | NA | -0.324883045 | NA | NA | NA | NA |
| Efpeglenatide_high_dosage:Inject_semaglutide_high_dosage | 0 | 0.114665683 | NA | 0.114665683 | NA | NA | NA | NA |
| Efpeglenatide_high_dosage:Inject_semaglutide_low_dosage | 0 | 1.338470878 | NA | 1.338470878 | NA | NA | NA | NA |
| Efpeglenatide_high_dosage:Inject_semaglutide_medium_dosage | 0 | 0.429682228 | NA | 0.429682228 | NA | NA | NA | NA |
| Efpeglenatide_high_dosage:Liraglutide | 0 | 0.476279055 | NA | 0.476279055 | NA | NA | NA | NA |
| Efpeglenatide_high_dosage:Lixisenatide | 0 | 0.674315232 | NA | 0.674315232 | NA | NA | NA | NA |
| Efpeglenatide_high_dosage:Oral_semaglutide | 0 | 1.097030064 | NA | 1.097030064 | NA | NA | NA | NA |
| Efpeglenatide_high_dosage:Placebo_or_Control | 0 | -0.00221076 | NA | -0.00221076 | NA | NA | NA | NA |
| Efpeglenatide_high_dosage:Sotagliflozin | 0 | -0.001264955 | NA | -0.001264955 | NA | NA | NA | NA |
| Efpeglenatide_high_dosage:Tirzepatide | 0 | 1.780766218 | NA | 1.780766218 | NA | NA | NA | NA |
| Efpeglenatide_medium_dosage:Empagliflozin_high_dosage | 0 | 1.090152798 | NA | 1.090152798 | NA | NA | NA | NA |
| Efpeglenatide_medium_dosage:Empagliflozin_low_dosage | 0 | 0.129416424 | NA | 0.129416424 | NA | NA | NA | NA |
| Efpeglenatide_medium_dosage:Ertugliflozin_high_dosage | 0 | -0.002946595 | NA | -0.002946595 | NA | NA | NA | NA |
| Efpeglenatide_medium_dosage:Ertugliflozin_low_dosage | 0 | 0.402518513 | NA | 0.402518513 | NA | NA | NA | NA |

| | | | | | | | | |
|---|---|---|---|---|---|---|---|---|
| Efpeglenatide_medium_dosage:Exenatide | 0 | 0.772993408 | NA | 0.772993408 | NA | NA | NA | NA |
| Efpeglenatide_medium_dosage:Inject_semaglutide_high_dosage | 0 | 1.212542137 | NA | 1.212542137 | NA | NA | NA | NA |
| Efpeglenatide_medium_dosage:Inject_semaglutide_low_dosage | 0 | 2.436347331 | NA | 2.436347331 | NA | NA | NA | NA |
| Efpeglenatide_medium_dosage:Inject_semaglutide_medium_dosage | 0 | 1.527558681 | NA | 1.527558681 | NA | NA | NA | NA |
| Efpeglenatide_medium_dosage:Liraglutide | 0 | 1.574155508 | NA | 1.574155508 | NA | NA | NA | NA |
| Efpeglenatide_medium_dosage:Lixisenatide | 0 | 1.772191686 | NA | 1.772191686 | NA | NA | NA | NA |
| Efpeglenatide_medium_dosage:Oral_semaglutide | 0 | 2.194906518 | NA | 2.194906518 | NA | NA | NA | NA |
| Efpeglenatide_medium_dosage:Placebo_or_Control | 1 | 1.095665694 | 1.095665694 | NA | NA | NA | NA | NA |
| Efpeglenatide_medium_dosage:Sotagliflozin | 0 | 1.096611499 | NA | 1.096611499 | NA | NA | NA | NA |
| Efpeglenatide_medium_dosage:Tirzepatide | 0 | 2.878642671 | NA | 2.878642671 | NA | NA | NA | NA |
| Empagliflozin_high_dosage:Empagliflozin_low_dosage | 3 | -0.960736373 | -0.809426225 | -1.277464162 | 0.468037937 | -2.670921325 | 3.6069972 | 0.770101171 |
| Empagliflozin_high_dosage:Ertugliflozin_high_dosage | 0 | -1.093099393 | NA | -1.093099393 | NA | NA | NA | NA |
| Empagliflozin_high_dosage:Ertugliflozin_low_dosage | 0 | -0.687634285 | NA | -0.687634285 | NA | NA | NA | NA |
| Empagliflozin_high_dosage:Exenatide | 0 | -0.31715939 | NA | -0.31715939 | NA | NA | NA | NA |
| Empagliflozin_high_dosage:Inject_semaglutide_high_dosage | 0 | 0.122389339 | NA | 0.122389339 | NA | NA | NA | NA |
| Empagliflozin_high_dosage:Inject_semaglutide_low_dosage | 0 | 1.346194534 | NA | 1.346194534 | NA | NA | NA | NA |
| Empagliflozin_high_dosage:Inject_semaglutide_medium_dosage | 0 | 0.437405883 | NA | 0.437405883 | NA | NA | NA | NA |
| Empagliflozin_high_dosage:Liraglutide | 0 | 0.48400271 | NA | 0.48400271 | NA | NA | NA | NA |
| Empagliflozin_high_dosage:Lixisenatide | 0 | 0.682038888 | NA | 0.682038888 | NA | NA | NA | NA |
| Empagliflozin_high_dosage:Oral_semaglutide | 0 | 1.10475372 | NA | 1.10475372 | NA | NA | NA | NA |
| Empagliflozin_high_dosage:Placebo_or_Control | 2 | 0.005512896 | -0.238121707 | 0.22991623 | -0.468037937 | -3.6069972 | 2.670921325 | 0.770101171 |
| Empagliflozin_high_dosage:Sotagliflozin | 0 | 0.006458701 | NA | 0.006458701 | NA | NA | NA | NA |

| | | | | | | | | |
|---|---|---|---|---|---|---|---|---|
| Empagliflozin_high_dosage:Tirzepatide | 0 | 1.788489873 | NA | 1.788489873 | NA | NA | NA | NA |
| Empagliflozin_low_dosage:Ertugliflozin_high_dosage | 0 | -0.13236302 | NA | -0.13236302 | NA | NA | NA | NA |
| Empagliflozin_low_dosage:Ertugliflozin_low_dosage | 0 | 0.273102089 | NA | 0.273102089 | NA | NA | NA | NA |
| Empagliflozin_low_dosage:Exenatide | 0 | 0.643576984 | NA | 0.643576984 | NA | NA | NA | NA |
| Empagliflozin_low_dosage:Inject_semaglutide_high_dosage | 0 | 1.083125712 | NA | 1.083125712 | NA | NA | NA | NA |
| Empagliflozin_low_dosage:Inject_semaglutide_low_dosage | 0 | 2.306930907 | NA | 2.306930907 | NA | NA | NA | NA |
| Empagliflozin_low_dosage:Inject_semaglutide_medium_dosage | 0 | 1.398142257 | NA | 1.398142257 | NA | NA | NA | NA |
| Empagliflozin_low_dosage:Liraglutide | 0 | 1.444739084 | NA | 1.444739084 | NA | NA | NA | NA |
| Empagliflozin_low_dosage:Lixisenatide | 0 | 1.642775261 | NA | 1.642775261 | NA | NA | NA | NA |
| Empagliflozin_low_dosage:Oral_semaglutide | 0 | 2.065490093 | NA | 2.065490093 | NA | NA | NA | NA |
| Empagliflozin_low_dosage:Placebo_or_Control | 5 | 0.966249269 | 1.039342455 | 0.571304518 | 0.468037937 | -2.670921325 | 3.6069972 | 0.770101171 |
| Empagliflozin_low_dosage:Sotagliflozin | 0 | 0.967195075 | NA | 0.967195075 | NA | NA | NA | NA |
| Empagliflozin_low_dosage:Tirzepatide | 0 | 2.749226247 | NA | 2.749226247 | NA | NA | NA | NA |
| Ertugliflozin_high_dosage:Ertugliflozin_low_dosage | 1 | 0.405465108 | 0.405465108 | NA | NA | NA | NA | NA |
| Ertugliflozin_high_dosage:Exenatide | 0 | 0.775940003 | NA | 0.775940003 | NA | NA | NA | NA |
| Ertugliflozin_high_dosage:Inject_semaglutide_high_dosage | 0 | 1.215488732 | NA | 1.215488732 | NA | NA | NA | NA |
| Ertugliflozin_high_dosage:Inject_semaglutide_low_dosage | 0 | 2.439293927 | NA | 2.439293927 | NA | NA | NA | NA |
| Ertugliflozin_high_dosage:Inject_semaglutide_medium_dosage | 0 | 1.530505276 | NA | 1.530505276 | NA | NA | NA | NA |
| Ertugliflozin_high_dosage:Liraglutide | 0 | 1.577102103 | NA | 1.577102103 | NA | NA | NA | NA |
| Ertugliflozin_high_dosage:Lixisenatide | 0 | 1.775138281 | NA | 1.775138281 | NA | NA | NA | NA |
| Ertugliflozin_high_dosage:Oral_semaglutide | 0 | 2.197853113 | NA | 2.197853113 | NA | NA | NA | NA |
| Ertugliflozin_high_dosage:Placebo_or_Control | 1 | 1.098612289 | 1.098612289 | NA | NA | NA | NA | NA |

| | | | | | | | | |
|---|---|---|---|---|---|---|---|---|
| Ertugliflozin_high_dosage:Sotagliflozin | 0 | 1.099558094 | NA | 1.099558094 | NA | NA | NA | NA |
| Ertugliflozin_high_dosage:Tirzepatide | 0 | 2.881589266 | NA | 2.881589266 | NA | NA | NA | NA |
| Ertugliflozin_low_dosage:Exenatide | 0 | 0.370474895 | NA | 0.370474895 | NA | NA | NA | NA |
| Ertugliflozin_low_dosage:Inject_semaglutide_high_dosage | 0 | 0.810023624 | NA | 0.810023624 | NA | NA | NA | NA |
| Ertugliflozin_low_dosage:Inject_semaglutide_low_dosage | 0 | 2.033828818 | NA | 2.033828818 | NA | NA | NA | NA |
| Ertugliflozin_low_dosage:Inject_semaglutide_medium_dosage | 0 | 1.125040168 | NA | 1.125040168 | NA | NA | NA | NA |
| Ertugliflozin_low_dosage:Liraglutide | 0 | 1.171636995 | NA | 1.171636995 | NA | NA | NA | NA |
| Ertugliflozin_low_dosage:Lixisenatide | 0 | 1.369673173 | NA | 1.369673173 | NA | NA | NA | NA |
| Ertugliflozin_low_dosage:Oral_semaglutide | 0 | 1.792388005 | NA | 1.792388005 | NA | NA | NA | NA |
| Ertugliflozin_low_dosage:Placebo_or_Control | 1 | 0.693147181 | 0.693147181 | NA | NA | NA | NA | NA |
| Ertugliflozin_low_dosage:Sotagliflozin | 0 | 0.694092986 | NA | 0.694092986 | NA | NA | NA | NA |
| Ertugliflozin_low_dosage:Tirzepatide | 0 | 2.476124158 | NA | 2.476124158 | NA | NA | NA | NA |
| Exenatide:Inject_semaglutide_high_dosage | 0 | 0.439548729 | NA | 0.439548729 | NA | NA | NA | NA |
| Exenatide:Inject_semaglutide_low_dosage | 0 | 1.663353923 | NA | 1.663353923 | NA | NA | NA | NA |
| Exenatide:Inject_semaglutide_medium_dosage | 0 | 0.754565273 | NA | 0.754565273 | NA | NA | NA | NA |
| Exenatide:Liraglutide | 0 | 0.8011621 | NA | 0.8011621 | NA | NA | NA | NA |
| Exenatide:Lixisenatide | 0 | 0.999198278 | NA | 0.999198278 | NA | NA | NA | NA |
| Exenatide:Oral_semaglutide | 0 | 1.42191311 | NA | 1.42191311 | NA | NA | NA | NA |
| Exenatide:Placebo_or_Control | 1 | 0.322672285 | 0.322672285 | NA | NA | NA | NA | NA |
| Exenatide:Sotagliflozin | 0 | 0.323618091 | NA | 0.323618091 | NA | NA | NA | NA |
| Exenatide:Tirzepatide | 0 | 2.105649263 | NA | 2.105649263 | NA | NA | NA | NA |
| Inject_semaglutide_high_dosage:Inject_semaglutide_low_dosage | 0 | 1.223805195 | NA | 1.223805195 | NA | NA | NA | NA |

| | | | | | | | | |
|---|---|---|---|---|---|---|---|---|
| Inject_semaglutide_high_dosage:Inject_semaglutide_medium_dosage | 1 | 0.315016544 | 1.098612289 | -0.038596271 | 1.137208559 | -2.722110892 | 4.996528011 | 0.563578844 |
| Inject_semaglutide_high_dosage:Liraglutide | 0 | 0.361613371 | NA | 0.361613371 | NA | NA | NA | NA |
| Inject_semaglutide_high_dosage:Lixisenatide | 0 | 0.559649549 | NA | 0.559649549 | NA | NA | NA | NA |
| Inject_semaglutide_high_dosage:Oral_semaglutide | 0 | 0.982364381 | NA | 0.982364381 | NA | NA | NA | NA |
| Inject_semaglutide_high_dosage:Placebo_or_Control | 3 | -0.116876443 | -0.190618967 | 0.946589593 | -1.137208559 | -4.996528011 | 2.722110892 | 0.563578844 |
| Inject_semaglutide_high_dosage:Sotagliflozin | 0 | -0.115930638 | NA | -0.115930638 | NA | NA | NA | NA |
| Inject_semaglutide_high_dosage:Tirzepatide | 0 | 1.666100534 | NA | 1.666100534 | NA | NA | NA | NA |
| Inject_semaglutide_low_dosage:Inject_semaglutide_medium_dosage | 1 | -0.90878865 | -1.104680268 | 3.439996077 | -4.544676346 | -19.96784361 | 10.87849091 | 0.563578844 |
| Inject_semaglutide_low_dosage:Liraglutide | 0 | -0.862191823 | NA | -0.862191823 | NA | NA | NA | NA |
| Inject_semaglutide_low_dosage:Lixisenatide | 0 | -0.664155646 | NA | -0.664155646 | NA | NA | NA | NA |
| Inject_semaglutide_low_dosage:Oral_semaglutide | 0 | -0.241440814 | NA | -0.241440814 | NA | NA | NA | NA |
| Inject_semaglutide_low_dosage:Placebo_or_Control | 1 | -1.340681638 | -1.256702964 | -10.34229117 | 9.085588202 | -21.74797085 | 39.91914726 | 0.563578844 |
| Inject_semaglutide_low_dosage:Sotagliflozin | 0 | -1.339735832 | NA | -1.339735832 | NA | NA | NA | NA |
| Inject_semaglutide_low_dosage:Tirzepatide | 0 | 0.44229534 | NA | 0.44229534 | NA | NA | NA | NA |
| Inject_semaglutide_medium_dosage:Liraglutide | 0 | 0.046596827 | NA | 0.046596827 | NA | NA | NA | NA |
| Inject_semaglutide_medium_dosage:Lixisenatide | 0 | 0.244633005 | NA | 0.244633005 | NA | NA | NA | NA |
| Inject_semaglutide_medium_dosage:Oral_semaglutide | 0 | 0.667347837 | NA | 0.667347837 | NA | NA | NA | NA |
| Inject_semaglutide_medium_dosage:Placebo_or_Control | 1 | -0.431892988 | -0.152022696 | -1.289231256 | 1.137208559 | -2.722110892 | 4.996528011 | 0.563578844 |
| Inject_semaglutide_medium_dosage:Sotagliflozin | 0 | -0.430947182 | NA | -0.430947182 | NA | NA | NA | NA |
| Inject_semaglutide_medium_dosage:Tirzepatide | 0 | 1.35108399 | NA | 1.35108399 | NA | NA | NA | NA |
| Liraglutide:Lixisenatide | 1 | 0.198036178 | 1.103562794 | -0.585128252 | 1.688691046 | -2.690175395 | 6.067557487 | 0.449738117 |
| Liraglutide:Oral_semaglutide | 0 | 0.62075101 | NA | 0.62075101 | NA | NA | NA | NA |

| | | | | | | | | |
|---|---|---|---|---|---|---|---|---|
| Liraglutide:Placebo_or_Control | 2 | -0.478489814 | -0.584798273 | 1.103892772 | -1.688691046 | -6.067557487 | 2.690175395 | 0.449738117 |
| Liraglutide:Sotagliflozin | 0 | -0.477544009 | NA | -0.477544009 | NA | NA | NA | NA |
| Liraglutide:Tirzepatide | 0 | 1.304487163 | NA | 1.304487163 | NA | NA | NA | NA |
| Lixisenatide:Oral_semaglutide | 0 | 0.422714832 | NA | 0.422714832 | NA | NA | NA | NA |
| Lixisenatide:Placebo_or_Control | 1 | -0.676525992 | 0.000329979 | -1.688361067 | 1.688691046 | -2.690175395 | 6.067557487 | 0.449738117 |
| Lixisenatide:Sotagliflozin | 0 | -0.675580187 | NA | -0.675580187 | NA | NA | NA | NA |
| Lixisenatide:Tirzepatide | 0 | 1.106450985 | NA | 1.106450985 | NA | NA | NA | NA |
| Oral_semaglutide:Placebo_or_Control | 1 | -1.099240824 | -1.099240824 | NA | NA | NA | NA | NA |
| Oral_semaglutide:Sotagliflozin | 0 | -1.098295019 | NA | -1.098295019 | NA | NA | NA | NA |
| Oral_semaglutide:Tirzepatide | 0 | 0.683736153 | NA | 0.683736153 | NA | NA | NA | NA |
| Sotagliflozin:Placebo_or_Control | 1 | -0.000945805 | -0.000945805 | NA | NA | NA | NA | NA |
| Tirzepatide:Placebo_or_Control | 1 | -1.782976978 | -1.782976978 | NA | NA | NA | NA | NA |
| Sotagliflozin:Tirzepatide | 0 | 1.782031172 | NA | 1.782031172 | NA | NA | NA | NA |

## Table S7D: inconsistency within the network meta-analysis of primary outcome: subgroup of myeloma

| Comparison | No.Studies | NMA | Direct | Indirect | Difference | Diff_95CI_lower | Diff_95CI_upper | *p* value |
|---|---|---|---|---|---|---|---|---|
| Albiglutide:Canagliflozin_high_dosage | 0 | 2.197701357 | NA | 2.197701357 | NA | NA | NA | NA |
| Albiglutide:Canagliflozin_low_dosage | 0 | 2.198831956 | NA | 2.198831956 | NA | NA | NA | NA |
| Albiglutide:Dapagliflozin_high_dosage | 0 | 1.758089416 | NA | 1.758089416 | NA | NA | NA | NA |
| Albiglutide:Dulaglutide | 0 | 0.143916078 | NA | 0.143916078 | NA | NA | NA | NA |
| Albiglutide:Empagliflozin_high_dosage | 0 | 1.407795517 | NA | 1.407795517 | NA | NA | NA | NA |
| Albiglutide:Empagliflozin_low_dosage | 0 | 0.868338851 | NA | 0.868338851 | NA | NA | NA | NA |
| Albiglutide:Ertugliflozin_high_dosage | 0 | 1.08789439 | NA | 1.08789439 | NA | NA | NA | NA |
| Albiglutide:Ertugliflozin_low_dosage | 0 | 2.199402723 | NA | 2.199402723 | NA | NA | NA | NA |
| Albiglutide:Exenatide | 0 | 1.027880943 | NA | 1.027880943 | NA | NA | NA | NA |
| Albiglutide:Inject_semaglutide_high_dosage | 0 | 1.281062846 | NA | 1.281062846 | NA | NA | NA | NA |
| Albiglutide:Inject_semaglutide_low_dosage | 0 | 1.506591388 | NA | 1.506591388 | NA | NA | NA | NA |
| Albiglutide:Inject_semaglutide_medium_dosage | 0 | 1.501739953 | NA | 1.501739953 | NA | NA | NA | NA |
| Albiglutide:Liraglutide | 0 | 1.697756666 | NA | 1.697756666 | NA | NA | NA | NA |
| Albiglutide:Lixisenatide | 0 | -0.000871784 | NA | -0.000871784 | NA | NA | NA | NA |
| Albiglutide:Oral_semaglutide | 0 | 0.547184052 | NA | 0.547184052 | NA | NA | NA | NA |
| Albiglutide:Placebo_or_Control | 1 | 1.098400245 | 1.098400245 | NA | NA | NA | NA | NA |
| Albiglutide:Sotagliflozin | 0 | 1.031522199 | NA | 1.031522199 | NA | NA | NA | NA |
| Albiglutide:Tirzepatide | 0 | 3.279411078 | NA | 3.279411078 | NA | NA | NA | NA |
| Canagliflozin_high_dosage:Canagliflozin_low_dosage | 0 | 0.001130599 | NA | 0.001130599 | NA | NA | NA | NA |
| Canagliflozin_high_dosage:Dapagliflozin_high_dosage | 0 | -0.439611942 | NA | -0.439611942 | NA | NA | NA | NA |

| | | | | | | | | |
|---|---|---|---|---|---|---|---|---|
| Canagliflozin_high_dosage:Dulaglutide | 0 | -2.053785279 | NA | -2.053785279 | NA | NA | NA | NA |
| Canagliflozin_high_dosage:Empagliflozin_high_dosage | 0 | -0.78990584 | NA | -0.78990584 | NA | NA | NA | NA |
| Canagliflozin_high_dosage:Empagliflozin_low_dosage | 0 | -1.329362506 | NA | -1.329362506 | NA | NA | NA | NA |
| Canagliflozin_high_dosage:Ertugliflozin_high_dosage | 0 | -1.109806967 | NA | -1.109806967 | NA | NA | NA | NA |
| Canagliflozin_high_dosage:Ertugliflozin_low_dosage | 0 | 0.001701366 | NA | 0.001701366 | NA | NA | NA | NA |
| Canagliflozin_high_dosage:Exenatide | 0 | -1.169820414 | NA | -1.169820414 | NA | NA | NA | NA |
| Canagliflozin_high_dosage:Inject_semaglutide_high_dosage | 0 | -0.916638511 | NA | -0.916638511 | NA | NA | NA | NA |
| Canagliflozin_high_dosage:Inject_semaglutide_low_dosage | 0 | -0.691109969 | NA | -0.691109969 | NA | NA | NA | NA |
| Canagliflozin_high_dosage:Inject_semaglutide_medium_dosage | 0 | -0.695961404 | NA | -0.695961404 | NA | NA | NA | NA |
| Canagliflozin_high_dosage:Liraglutide | 0 | -0.499944691 | NA | -0.499944691 | NA | NA | NA | NA |
| Canagliflozin_high_dosage:Lixisenatide | 0 | -2.198573141 | NA | -2.198573141 | NA | NA | NA | NA |
| Canagliflozin_high_dosage:Oral_semaglutide | 0 | -1.650517305 | NA | -1.650517305 | NA | NA | NA | NA |
| Canagliflozin_high_dosage:Placebo_or_Control | 1 | -1.099301113 | -1.099301113 | NA | NA | NA | NA | NA |
| Canagliflozin_high_dosage:Sotagliflozin | 0 | -1.166179158 | NA | -1.166179158 | NA | NA | NA | NA |
| Canagliflozin_high_dosage:Tirzepatide | 0 | 1.08170972 | NA | 1.08170972 | NA | NA | NA | NA |
| Canagliflozin_low_dosage:Dapagliflozin_high_dosage | 0 | -0.440742541 | NA | -0.440742541 | NA | NA | NA | NA |
| Canagliflozin_low_dosage:Dulaglutide | 0 | -2.054915878 | NA | -2.054915878 | NA | NA | NA | NA |
| Canagliflozin_low_dosage:Empagliflozin_high_dosage | 0 | -0.791036439 | NA | -0.791036439 | NA | NA | NA | NA |
| Canagliflozin_low_dosage:Empagliflozin_low_dosage | 0 | -1.330493105 | NA | -1.330493105 | NA | NA | NA | NA |
| Canagliflozin_low_dosage:Ertugliflozin_high_dosage | 0 | -1.110937566 | NA | -1.110937566 | NA | NA | NA | NA |
| Canagliflozin_low_dosage:Ertugliflozin_low_dosage | 0 | 0.000570767 | NA | 0.000570767 | NA | NA | NA | NA |
| Canagliflozin_low_dosage:Exenatide | 0 | -1.170951013 | NA | -1.170951013 | NA | NA | NA | NA |

| | | | | | | | | |
|---|---|---|---|---|---|---|---|---|
| Canagliflozin_low_dosage:Inject_semaglutide_high_dosage | 0 | -0.91776911 | NA | -0.91776911 | NA | NA | NA | NA |
| Canagliflozin_low_dosage:Inject_semaglutide_low_dosage | 0 | -0.692240568 | NA | -0.692240568 | NA | NA | NA | NA |
| Canagliflozin_low_dosage:Inject_semaglutide_medium_dosage | 0 | -0.697092003 | NA | -0.697092003 | NA | NA | NA | NA |
| Canagliflozin_low_dosage:Liraglutide | 0 | -0.50107529 | NA | -0.50107529 | NA | NA | NA | NA |
| Canagliflozin_low_dosage:Lixisenatide | 0 | -2.19970374 | NA | -2.19970374 | NA | NA | NA | NA |
| Canagliflozin_low_dosage:Oral_semaglutide | 0 | -1.651647904 | NA | -1.651647904 | NA | NA | NA | NA |
| Canagliflozin_low_dosage:Placebo_or_Control | 1 | -1.100431712 | -1.100431712 | NA | NA | NA | NA | NA |
| Canagliflozin_low_dosage:Sotagliflozin | 0 | -1.167309757 | NA | -1.167309757 | NA | NA | NA | NA |
| Canagliflozin_low_dosage:Tirzepatide | 0 | 1.080579122 | NA | 1.080579122 | NA | NA | NA | NA |
| Dapagliflozin_high_dosage:Dulaglutide | 0 | -1.614173337 | NA | -1.614173337 | NA | NA | NA | NA |
| Dapagliflozin_high_dosage:Empagliflozin_high_dosage | 0 | -0.350293899 | NA | -0.350293899 | NA | NA | NA | NA |
| Dapagliflozin_high_dosage:Empagliflozin_low_dosage | 0 | -0.889750564 | NA | -0.889750564 | NA | NA | NA | NA |
| Dapagliflozin_high_dosage:Ertugliflozin_high_dosage | 0 | -0.670195026 | NA | -0.670195026 | NA | NA | NA | NA |
| Dapagliflozin_high_dosage:Ertugliflozin_low_dosage | 0 | 0.441313307 | NA | 0.441313307 | NA | NA | NA | NA |
| Dapagliflozin_high_dosage:Exenatide | 0 | -0.730208472 | NA | -0.730208472 | NA | NA | NA | NA |
| Dapagliflozin_high_dosage:Inject_semaglutide_high_dosage | 0 | -0.477026569 | NA | -0.477026569 | NA | NA | NA | NA |
| Dapagliflozin_high_dosage:Inject_semaglutide_low_dosage | 0 | -0.251498028 | NA | -0.251498028 | NA | NA | NA | NA |
| Dapagliflozin_high_dosage:Inject_semaglutide_medium_dosage | 0 | -0.256349462 | NA | -0.256349462 | NA | NA | NA | NA |
| Dapagliflozin_high_dosage:Liraglutide | 0 | -0.060332749 | NA | -0.060332749 | NA | NA | NA | NA |
| Dapagliflozin_high_dosage:Lixisenatide | 0 | -1.758961199 | NA | -1.758961199 | NA | NA | NA | NA |
| Dapagliflozin_high_dosage:Oral_semaglutide | 0 | -1.210905364 | NA | -1.210905364 | NA | NA | NA | NA |
| Dapagliflozin_high_dosage:Placebo_or_Control | 4 | -0.659689171 | -0.659689171 | NA | NA | NA | NA | NA |

| | | | | | | | | |
|---|---|---|---|---|---|---|---|---|
| Dapagliflozin_high_dosage:Sotagliflozin | 0 | -0.726567217 | NA | -0.726567217 | NA | NA | NA | NA |
| Dapagliflozin_high_dosage:Tirzepatide | 0 | 1.521321662 | NA | 1.521321662 | NA | NA | NA | NA |
| Dulaglutide:Empagliflozin_high_dosage | 0 | 1.263879439 | NA | 1.263879439 | NA | NA | NA | NA |
| Dulaglutide:Empagliflozin_low_dosage | 0 | 0.724422773 | NA | 0.724422773 | NA | NA | NA | NA |
| Dulaglutide:Ertugliflozin_high_dosage | 0 | 0.943978312 | NA | 0.943978312 | NA | NA | NA | NA |
| Dulaglutide:Ertugliflozin_low_dosage | 0 | 2.055486645 | NA | 2.055486645 | NA | NA | NA | NA |
| Dulaglutide:Exenatide | 0 | 0.883964865 | NA | 0.883964865 | NA | NA | NA | NA |
| Dulaglutide:Inject_semaglutide_high_dosage | 0 | 1.137146768 | NA | 1.137146768 | NA | NA | NA | NA |
| Dulaglutide:Inject_semaglutide_low_dosage | 0 | 1.36267531 | NA | 1.36267531 | NA | NA | NA | NA |
| Dulaglutide:Inject_semaglutide_medium_dosage | 0 | 1.357823875 | NA | 1.357823875 | NA | NA | NA | NA |
| Dulaglutide:Liraglutide | 0 | 1.553840588 | NA | 1.553840588 | NA | NA | NA | NA |
| Dulaglutide:Lixisenatide | 0 | -0.144787862 | NA | -0.144787862 | NA | NA | NA | NA |
| Dulaglutide:Oral_semaglutide | 0 | 0.403267974 | NA | 0.403267974 | NA | NA | NA | NA |
| Dulaglutide:Placebo_or_Control | 2 | 0.954484166 | 0.954484166 | NA | NA | NA | NA | NA |
| Dulaglutide:Sotagliflozin | 0 | 0.887606121 | NA | 0.887606121 | NA | NA | NA | NA |
| Dulaglutide:Tirzepatide | 0 | 3.135494999 | NA | 3.135494999 | NA | NA | NA | NA |
| Empagliflozin_high_dosage:Empagliflozin_low_dosage | 1 | -0.539456665 | -0.69229321 | 1.276829362 | -1.969122571 | -10.58778917 | 6.649544027 | 0.654300021 |
| Empagliflozin_high_dosage:Ertugliflozin_high_dosage | 0 | -0.319901127 | NA | -0.319901127 | NA | NA | NA | NA |
| Empagliflozin_high_dosage:Ertugliflozin_low_dosage | 0 | 0.791607206 | NA | 0.791607206 | NA | NA | NA | NA |
| Empagliflozin_high_dosage:Exenatide | 0 | -0.379914574 | NA | -0.379914574 | NA | NA | NA | NA |
| Empagliflozin_high_dosage:Inject_semaglutide_high_dosage | 0 | -0.126732671 | NA | -0.126732671 | NA | NA | NA | NA |
| Empagliflozin_high_dosage:Inject_semaglutide_low_dosage | 0 | 0.098795871 | NA | 0.098795871 | NA | NA | NA | NA |

| | | | | | | | | |
|---|---|---|---|---|---|---|---|---|
| Empagliflozin_high_dosage:Inject_semaglutide_medium_dosage | 0 | 0.093944437 | NA | 0.093944437 | NA | NA | NA | NA |
| Empagliflozin_high_dosage:Liraglutide | 0 | 0.28996115 | NA | 0.28996115 | NA | NA | NA | NA |
| Empagliflozin_high_dosage:Lixisenatide | 0 | -1.4086673 | NA | -1.4086673 | NA | NA | NA | NA |
| Empagliflozin_high_dosage:Oral_semaglutide | 0 | -0.860611465 | NA | -0.860611465 | NA | NA | NA | NA |
| Empagliflozin_high_dosage:Placebo_or_Control | 1 | -0.309395272 | -0.00385192 | -1.316972211 | 1.313120291 | -4.434285257 | 7.060525839 | 0.654300021 |
| Empagliflozin_high_dosage:Sotagliflozin | 0 | -0.376273318 | NA | -0.376273318 | NA | NA | NA | NA |
| Empagliflozin_high_dosage:Tirzepatide | 0 | 1.871615561 | NA | 1.871615561 | NA | NA | NA | NA |
| Empagliflozin_low_dosage:Ertugliflozin_high_dosage | 0 | 0.219555539 | NA | 0.219555539 | NA | NA | NA | NA |
| Empagliflozin_low_dosage:Ertugliflozin_low_dosage | 0 | 1.331063872 | NA | 1.331063872 | NA | NA | NA | NA |
| Empagliflozin_low_dosage:Exenatide | 0 | 0.159542092 | NA | 0.159542092 | NA | NA | NA | NA |
| Empagliflozin_low_dosage:Inject_semaglutide_high_dosage | 0 | 0.412723995 | NA | 0.412723995 | NA | NA | NA | NA |
| Empagliflozin_low_dosage:Inject_semaglutide_low_dosage | 0 | 0.638252537 | NA | 0.638252537 | NA | NA | NA | NA |
| Empagliflozin_low_dosage:Inject_semaglutide_medium_dosage | 0 | 0.633401102 | NA | 0.633401102 | NA | NA | NA | NA |
| Empagliflozin_low_dosage:Liraglutide | 0 | 0.829417815 | NA | 0.829417815 | NA | NA | NA | NA |
| Empagliflozin_low_dosage:Lixisenatide | 0 | -0.869210635 | NA | -0.869210635 | NA | NA | NA | NA |
| Empagliflozin_low_dosage:Oral_semaglutide | 0 | -0.321154799 | NA | -0.321154799 | NA | NA | NA | NA |
| Empagliflozin_low_dosage:Placebo_or_Control | 4 | 0.230061393 | 0.230061393 | NA | NA | NA | NA | NA |
| Empagliflozin_low_dosage:Sotagliflozin | 0 | 0.163183347 | NA | 0.163183347 | NA | NA | NA | NA |
| Empagliflozin_low_dosage:Tirzepatide | 0 | 2.411072226 | NA | 2.411072226 | NA | NA | NA | NA |
| Ertugliflozin_high_dosage:Ertugliflozin_low_dosage | 1 | 1.111508333 | 1.113175653 | 1.109014356 | 0.004161296 | -5.061387725 | 5.069710318 | 0.998715334 |
| Ertugliflozin_high_dosage:Exenatide | 0 | -0.060013447 | NA | -0.060013447 | NA | NA | NA | NA |
| Ertugliflozin_high_dosage:Inject_semaglutide_high_dosage | 0 | 0.193168456 | NA | 0.193168456 | NA | NA | NA | NA |

| | | | | | | | | |
|---|---|---|---|---|---|---|---|---|
| Ertugliflozin_high_dosage:Inject_semaglutide_low_dosage | 0 | 0.418696998 | NA | 0.418696998 | NA | NA | NA | NA |
| Ertugliflozin_high_dosage:Inject_semaglutide_medium_dosage | 0 | 0.413845563 | NA | 0.413845563 | NA | NA | NA | NA |
| Ertugliflozin_high_dosage:Liraglutide | 0 | 0.609862276 | NA | 0.609862276 | NA | NA | NA | NA |
| Ertugliflozin_high_dosage:Lixisenatide | 0 | -1.088766174 | NA | -1.088766174 | NA | NA | NA | NA |
| Ertugliflozin_high_dosage:Oral_semaglutide | 0 | -0.540710338 | NA | -0.540710338 | NA | NA | NA | NA |
| Ertugliflozin_high_dosage:Placebo_or_Control | 2 | 0.010505855 | 0.009673603 | 0.013834899 | -0.004161296 | -5.069710318 | 5.061387725 | 0.998715334 |
| Ertugliflozin_high_dosage:Sotagliflozin | 0 | -0.056372191 | NA | -0.056372191 | NA | NA | NA | NA |
| Ertugliflozin_high_dosage:Tirzepatide | 0 | 2.191516688 | NA | 2.191516688 | NA | NA | NA | NA |
| Ertugliflozin_low_dosage:Exenatide | 0 | -1.17152178 | NA | -1.17152178 | NA | NA | NA | NA |
| Ertugliflozin_low_dosage:Inject_semaglutide_high_dosage | 0 | -0.918339877 | NA | -0.918339877 | NA | NA | NA | NA |
| Ertugliflozin_low_dosage:Inject_semaglutide_low_dosage | 0 | -0.692811335 | NA | -0.692811335 | NA | NA | NA | NA |
| Ertugliflozin_low_dosage:Inject_semaglutide_medium_dosage | 0 | -0.69766277 | NA | -0.69766277 | NA | NA | NA | NA |
| Ertugliflozin_low_dosage:Liraglutide | 0 | -0.501646057 | NA | -0.501646057 | NA | NA | NA | NA |
| Ertugliflozin_low_dosage:Lixisenatide | 0 | -2.200274507 | NA | -2.200274507 | NA | NA | NA | NA |
| Ertugliflozin_low_dosage:Oral_semaglutide | 0 | -1.652218671 | NA | -1.652218671 | NA | NA | NA | NA |
| Ertugliflozin_low_dosage:Placebo_or_Control | 1 | -1.101002478 | -1.099340753 | -1.10350205 | 0.004161296 | -5.061387725 | 5.069710318 | 0.998715334 |
| Ertugliflozin_low_dosage:Sotagliflozin | 0 | -1.167880524 | NA | -1.167880524 | NA | NA | NA | NA |
| Ertugliflozin_low_dosage:Tirzepatide | 0 | 1.080008355 | NA | 1.080008355 | NA | NA | NA | NA |
| Exenatide:Inject_semaglutide_high_dosage | 0 | 0.253181903 | NA | 0.253181903 | NA | NA | NA | NA |
| Exenatide:Inject_semaglutide_low_dosage | 0 | 0.478710445 | NA | 0.478710445 | NA | NA | NA | NA |
| Exenatide:Inject_semaglutide_medium_dosage | 0 | 0.47385901 | NA | 0.47385901 | NA | NA | NA | NA |
| Exenatide:Liraglutide | 1 | 0.669875723 | 1.115743329 | 0.514204965 | 0.601538363 | -3.1220149 | 4.325091626 | 0.751523379 |

| | | | | | | | | |
|---|---|---|---|---|---|---|---|---|
| Exenatide:Lixisenatide | 0 | -1.028752727 | NA | -1.028752727 | NA | NA | NA | NA |
| Exenatide:Oral_semaglutide | 0 | -0.480696891 | NA | -0.480696891 | NA | NA | NA | NA |
| Exenatide:Placebo_or_Control | 1 | 0.070519301 | 0.003807974 | 0.605346338 | -0.601538363 | -4.325091626 | 3.1220149 | 0.751523379 |
| Exenatide:Sotagliflozin | 0 | 0.003641256 | NA | 0.003641256 | NA | NA | NA | NA |
| Exenatide:Tirzepatide | 0 | 2.251530134 | NA | 2.251530134 | NA | NA | NA | NA |
| Inject_semaglutide_high_dosage:Inject_semaglutide_low_dosage | 0 | 0.225528542 | NA | 0.225528542 | NA | NA | NA | NA |
| Inject_semaglutide_high_dosage:Inject_semaglutide_medium_dosage | 0 | 0.220677107 | NA | 0.220677107 | NA | NA | NA | NA |
| Inject_semaglutide_high_dosage:Liraglutide | 0 | 0.41669382 | NA | 0.41669382 | NA | NA | NA | NA |
| Inject_semaglutide_high_dosage:Lixisenatide | 0 | -1.28193463 | NA | -1.28193463 | NA | NA | NA | NA |
| Inject_semaglutide_high_dosage:Oral_semaglutide | 0 | -0.733878794 | NA | -0.733878794 | NA | NA | NA | NA |
| Inject_semaglutide_high_dosage:Placebo_or_Control | 1 | -0.182662602 | -0.182662602 | NA | NA | NA | NA | NA |
| Inject_semaglutide_high_dosage:Sotagliflozin | 0 | -0.249540647 | NA | -0.249540647 | NA | NA | NA | NA |
| Inject_semaglutide_high_dosage:Tirzepatide | 0 | 1.998348231 | NA | 1.998348231 | NA | NA | NA | NA |
| Inject_semaglutide_low_dosage:Inject_semaglutide_medium_dosage | 0 | -0.004851435 | NA | -0.004851435 | NA | NA | NA | NA |
| Inject_semaglutide_low_dosage:Liraglutide | 0 | 0.191165278 | NA | 0.191165278 | NA | NA | NA | NA |
| Inject_semaglutide_low_dosage:Lixisenatide | 0 | -1.507463172 | NA | -1.507463172 | NA | NA | NA | NA |
| Inject_semaglutide_low_dosage:Oral_semaglutide | 0 | -0.959407336 | NA | -0.959407336 | NA | NA | NA | NA |
| Inject_semaglutide_low_dosage:Placebo_or_Control | 1 | -0.408191143 | -0.408191143 | NA | NA | NA | NA | NA |
| Inject_semaglutide_low_dosage:Sotagliflozin | 0 | -0.475069189 | NA | -0.475069189 | NA | NA | NA | NA |
| Inject_semaglutide_low_dosage:Tirzepatide | 0 | 1.77281969 | NA | 1.77281969 | NA | NA | NA | NA |
| Inject_semaglutide_medium_dosage:Liraglutide | 0 | 0.196016713 | NA | 0.196016713 | NA | NA | NA | NA |
| Inject_semaglutide_medium_dosage:Lixisenatide | 0 | -1.502611737 | NA | -1.502611737 | NA | NA | NA | NA |

| | | | | | | | | |
|---|---|---|---|---|---|---|---|---|
| Inject_semaglutide_medium_dosage:Oral_semaglutide | 0 | -0.954555901 | NA | -0.954555901 | NA | NA | NA | NA |
| Inject_semaglutide_medium_dosage:Placebo_or_Control | 1 | -0.403339709 | -0.403339709 | NA | NA | NA | NA | NA |
| Inject_semaglutide_medium_dosage:Sotagliflozin | 0 | -0.470217755 | NA | -0.470217755 | NA | NA | NA | NA |
| Inject_semaglutide_medium_dosage:Tirzepatide | 0 | 1.777671124 | NA | 1.777671124 | NA | NA | NA | NA |
| Liraglutide:Lixisenatide | 0 | -1.69862845 | NA | -1.69862845 | NA | NA | NA | NA |
| Liraglutide:Oral_semaglutide | 0 | -1.150572614 | NA | -1.150572614 | NA | NA | NA | NA |
| Liraglutide:Placebo_or_Control | 1 | -0.599356422 | -0.510396991 | -1.111935354 | 0.601538363 | -3.1220149 | 4.325091626 | 0.751523379 |
| Liraglutide:Sotagliflozin | 0 | -0.666234468 | NA | -0.666234468 | NA | NA | NA | NA |
| Liraglutide:Tirzepatide | 0 | 1.581654411 | NA | 1.581654411 | NA | NA | NA | NA |
| Lixisenatide:Oral_semaglutide | 0 | 0.548055836 | NA | 0.548055836 | NA | NA | NA | NA |
| Lixisenatide:Placebo_or_Control | 1 | 1.099272028 | 1.099272028 | NA | NA | NA | NA | NA |
| Lixisenatide:Sotagliflozin | 0 | 1.032393982 | NA | 1.032393982 | NA | NA | NA | NA |
| Lixisenatide:Tirzepatide | 0 | 3.280282861 | NA | 3.280282861 | NA | NA | NA | NA |
| Oral_semaglutide:Placebo_or_Control | 2 | 0.551216193 | 0.551216193 | NA | NA | NA | NA | NA |
| Oral_semaglutide:Sotagliflozin | 0 | 0.484338147 | NA | 0.484338147 | NA | NA | NA | NA |
| Oral_semaglutide:Tirzepatide | 0 | 2.732227026 | NA | 2.732227026 | NA | NA | NA | NA |
| Sotagliflozin:Placebo_or_Control | 2 | 0.066878046 | 0.066878046 | NA | NA | NA | NA | NA |
| Tirzepatide:Placebo_or_Control | 1 | -2.181010833 | -2.181010833 | NA | NA | NA | NA | NA |
| Sotagliflozin:Tirzepatide | 0 | 2.247888879 | NA | 2.247888879 | NA | NA | NA | NA |

## Table S7E: inconsistency within the network meta-analysis of safety profile: drop-out rate

| Comparison | No.Studies | NMA | Direct | Indirect | Difference | Diff_95CI_lower | Diff_95CI_upper | *p* value |
|---|---|---|---|---|---|---|---|---|
| Albiglutide:Bexagliflozin | 0 | 0.029218958 | NA | 0.029218958 | NA | NA | NA | NA |
| Albiglutide:Canagliflozin_high_dosage | 0 | 0.243731565 | NA | 0.243731565 | NA | NA | NA | NA |
| Albiglutide:Canagliflozin_low_dosage | 0 | 0.144417243 | NA | 0.144417243 | NA | NA | NA | NA |
| Albiglutide:Dapagliflozin_high_dosage | 0 | 0.132491881 | NA | 0.132491881 | NA | NA | NA | NA |
| Albiglutide:Dapagliflozin_low_dosage | 0 | 0.127568939 | NA | 0.127568939 | NA | NA | NA | NA |
| Albiglutide:Dapagliflozin_medium_dosage | 0 | 0.108768998 | NA | 0.108768998 | NA | NA | NA | NA |
| Albiglutide:Dulaglutide | 0 | -0.083654418 | NA | -0.083654418 | NA | NA | NA | NA |
| Albiglutide:Efpeglenatide_high_dosage | 0 | -0.090086972 | NA | -0.090086972 | NA | NA | NA | NA |
| Albiglutide:Efpeglenatide_medium_dosage | 0 | -0.070259529 | NA | -0.070259529 | NA | NA | NA | NA |
| Albiglutide:Empagliflozin_high_dosage | 0 | 0.200613003 | NA | 0.200613003 | NA | NA | NA | NA |
| Albiglutide:Empagliflozin_low_dosage | 0 | 0.030884639 | NA | 0.030884639 | NA | NA | NA | NA |
| Albiglutide:Ertugliflozin_high_dosage | 0 | -0.025495097 | NA | -0.025495097 | NA | NA | NA | NA |
| Albiglutide:Ertugliflozin_low_dosage | 0 | 0.082537251 | NA | 0.082537251 | NA | NA | NA | NA |
| Albiglutide:Exenatide | 0 | 0.097246873 | NA | 0.097246873 | NA | NA | NA | NA |
| Albiglutide:Inject_semaglutide_high_dosage | 0 | 0.240902346 | NA | 0.240902346 | NA | NA | NA | NA |
| Albiglutide:Inject_semaglutide_low_dosage | 0 | 0.389899155 | NA | 0.389899155 | NA | NA | NA | NA |
| Albiglutide:Inject_semaglutide_medium_dosage | 0 | 0.33062504 | NA | 0.33062504 | NA | NA | NA | NA |
| Albiglutide:Liraglutide | 0 | 0.588612086 | NA | 0.588612086 | NA | NA | NA | NA |
| Albiglutide:Lixisenatide | 0 | 0.075071443 | NA | 0.075071443 | NA | NA | NA | NA |
| Albiglutide:Oral_semaglutide | 0 | -0.564978106 | NA | -0.564978106 | NA | NA | NA | NA |

| | | | | | | | | |
|---|---|---|---|---|---|---|---|---|
| Albiglutide:Placebo_or_Control | 5 | -0.103216354 | -0.103216354 | NA | NA | NA | NA | NA |
| Albiglutide:Sotagliflozin | 0 | -0.016120889 | NA | -0.016120889 | NA | NA | NA | NA |
| Albiglutide:Tirzepatide | 0 | 0.481489458 | NA | 0.481489458 | NA | NA | NA | NA |
| Bexagliflozin:Canagliflozin_high_dosage | 0 | 0.214512607 | NA | 0.214512607 | NA | NA | NA | NA |
| Bexagliflozin:Canagliflozin_low_dosage | 0 | 0.115198285 | NA | 0.115198285 | NA | NA | NA | NA |
| Bexagliflozin:Dapagliflozin_high_dosage | 0 | 0.103272923 | NA | 0.103272923 | NA | NA | NA | NA |
| Bexagliflozin:Dapagliflozin_low_dosage | 0 | 0.098349981 | NA | 0.098349981 | NA | NA | NA | NA |
| Bexagliflozin:Dapagliflozin_medium_dosage | 0 | 0.07955004 | NA | 0.07955004 | NA | NA | NA | NA |
| Bexagliflozin:Dulaglutide | 0 | -0.112873377 | NA | -0.112873377 | NA | NA | NA | NA |
| Bexagliflozin:Efpeglenatide_high_dosage | 0 | -0.119305931 | NA | -0.119305931 | NA | NA | NA | NA |
| Bexagliflozin:Efpeglenatide_medium_dosage | 0 | -0.099478487 | NA | -0.099478487 | NA | NA | NA | NA |
| Bexagliflozin:Empagliflozin_high_dosage | 0 | 0.171394044 | NA | 0.171394044 | NA | NA | NA | NA |
| Bexagliflozin:Empagliflozin_low_dosage | 0 | 0.001665681 | NA | 0.001665681 | NA | NA | NA | NA |
| Bexagliflozin:Ertugliflozin_high_dosage | 0 | -0.054714055 | NA | -0.054714055 | NA | NA | NA | NA |
| Bexagliflozin:Ertugliflozin_low_dosage | 0 | 0.053318293 | NA | 0.053318293 | NA | NA | NA | NA |
| Bexagliflozin:Exenatide | 0 | 0.068027915 | NA | 0.068027915 | NA | NA | NA | NA |
| Bexagliflozin:Inject_semaglutide_high_dosage | 0 | 0.211683388 | NA | 0.211683388 | NA | NA | NA | NA |
| Bexagliflozin:Inject_semaglutide_low_dosage | 0 | 0.360680197 | NA | 0.360680197 | NA | NA | NA | NA |
| Bexagliflozin:Inject_semaglutide_medium_dosage | 0 | 0.301406082 | NA | 0.301406082 | NA | NA | NA | NA |
| Bexagliflozin:Liraglutide | 0 | 0.559393127 | NA | 0.559393127 | NA | NA | NA | NA |
| Bexagliflozin:Lixisenatide | 0 | 0.045852485 | NA | 0.045852485 | NA | NA | NA | NA |
| Bexagliflozin:Oral_semaglutide | 0 | -0.594197065 | NA | -0.594197065 | NA | NA | NA | NA |

| | | | | | | | | |
|---|---|---|---|---|---|---|---|---|
| Bexagliflozin:Placebo_or_Control | 1 | -0.132435312 | -0.132435312 | NA | NA | NA | NA | NA |
| Bexagliflozin:Sotagliflozin | 0 | -0.045339847 | NA | -0.045339847 | NA | NA | NA | NA |
| Bexagliflozin:Tirzepatide | 0 | 0.4522705 | NA | 0.4522705 | NA | NA | NA | NA |
| Canagliflozin_high_dosage:Canagliflozin_low_dosage | 1 | -0.099314322 | -0.145934915 | 0.010735285 | -0.156670199 | -1.155845755 | 0.842505356 | 0.758598811 |
| Canagliflozin_high_dosage:Dapagliflozin_high_dosage | 0 | -0.111239684 | NA | -0.111239684 | NA | NA | NA | NA |
| Canagliflozin_high_dosage:Dapagliflozin_low_dosage | 0 | -0.116162626 | NA | -0.116162626 | NA | NA | NA | NA |
| Canagliflozin_high_dosage:Dapagliflozin_medium_dosage | 0 | -0.134962567 | NA | -0.134962567 | NA | NA | NA | NA |
| Canagliflozin_high_dosage:Dulaglutide | 0 | -0.327385984 | NA | -0.327385984 | NA | NA | NA | NA |
| Canagliflozin_high_dosage:Efpeglenatide_high_dosage | 0 | -0.333818538 | NA | -0.333818538 | NA | NA | NA | NA |
| Canagliflozin_high_dosage:Efpeglenatide_medium_dosage | 0 | -0.313991094 | NA | -0.313991094 | NA | NA | NA | NA |
| Canagliflozin_high_dosage:Empagliflozin_high_dosage | 0 | -0.043118563 | NA | -0.043118563 | NA | NA | NA | NA |
| Canagliflozin_high_dosage:Empagliflozin_low_dosage | 0 | -0.212846926 | NA | -0.212846926 | NA | NA | NA | NA |
| Canagliflozin_high_dosage:Ertugliflozin_high_dosage | 0 | -0.269226663 | NA | -0.269226663 | NA | NA | NA | NA |
| Canagliflozin_high_dosage:Ertugliflozin_low_dosage | 0 | -0.161194314 | NA | -0.161194314 | NA | NA | NA | NA |
| Canagliflozin_high_dosage:Exenatide | 0 | -0.146484692 | NA | -0.146484692 | NA | NA | NA | NA |
| Canagliflozin_high_dosage:Inject_semaglutide_high_dosage | 0 | -0.002829219 | NA | -0.002829219 | NA | NA | NA | NA |
| Canagliflozin_high_dosage:Inject_semaglutide_low_dosage | 0 | 0.14616759 | NA | 0.14616759 | NA | NA | NA | NA |
| Canagliflozin_high_dosage:Inject_semaglutide_medium_dosage | 0 | 0.086893475 | NA | 0.086893475 | NA | NA | NA | NA |
| Canagliflozin_high_dosage:Liraglutide | 0 | 0.34488052 | NA | 0.34488052 | NA | NA | NA | NA |
| Canagliflozin_high_dosage:Lixisenatide | 0 | -0.168660122 | NA | -0.168660122 | NA | NA | NA | NA |
| Canagliflozin_high_dosage:Oral_semaglutide | 0 | -0.808709672 | NA | -0.808709672 | NA | NA | NA | NA |
| Canagliflozin_high_dosage:Placebo_or_Control | 2 | -0.34694792 | -0.368088873 | -0.062216635 | -0.305872239 | -1.874732205 | 1.262987728 | 0.702369615 |

| | | | | | | | | |
|---|---|---|---|---|---|---|---|---|
| Canagliflozin_high_dosage:Sotagliflozin | 0 | -0.259852454 | NA | -0.259852454 | NA | NA | NA | NA |
| Canagliflozin_high_dosage:Tirzepatide | 0 | 0.237757893 | NA | 0.237757893 | NA | NA | NA | NA |
| Canagliflozin_low_dosage:Dapagliflozin_high_dosage | 0 | -0.011925362 | NA | -0.011925362 | NA | NA | NA | NA |
| Canagliflozin_low_dosage:Dapagliflozin_low_dosage | 0 | -0.016848304 | NA | -0.016848304 | NA | NA | NA | NA |
| Canagliflozin_low_dosage:Dapagliflozin_medium_dosage | 0 | -0.035648245 | NA | -0.035648245 | NA | NA | NA | NA |
| Canagliflozin_low_dosage:Dulaglutide | 0 | -0.228071661 | NA | -0.228071661 | NA | NA | NA | NA |
| Canagliflozin_low_dosage:Efpeglenatide_high_dosage | 0 | -0.234504215 | NA | -0.234504215 | NA | NA | NA | NA |
| Canagliflozin_low_dosage:Efpeglenatide_medium_dosage | 0 | -0.214676772 | NA | -0.214676772 | NA | NA | NA | NA |
| Canagliflozin_low_dosage:Empagliflozin_high_dosage | 0 | 0.05619576 | NA | 0.05619576 | NA | NA | NA | NA |
| Canagliflozin_low_dosage:Empagliflozin_low_dosage | 0 | -0.113532604 | NA | -0.113532604 | NA | NA | NA | NA |
| Canagliflozin_low_dosage:Ertugliflozin_high_dosage | 0 | -0.16991234 | NA | -0.16991234 | NA | NA | NA | NA |
| Canagliflozin_low_dosage:Ertugliflozin_low_dosage | 0 | -0.061879992 | NA | -0.061879992 | NA | NA | NA | NA |
| Canagliflozin_low_dosage:Exenatide | 0 | -0.04717037 | NA | -0.04717037 | NA | NA | NA | NA |
| Canagliflozin_low_dosage:Inject_semaglutide_high_dosage | 0 | 0.096485103 | NA | 0.096485103 | NA | NA | NA | NA |
| Canagliflozin_low_dosage:Inject_semaglutide_low_dosage | 0 | 0.245481913 | NA | 0.245481913 | NA | NA | NA | NA |
| Canagliflozin_low_dosage:Inject_semaglutide_medium_dosage | 0 | 0.186207798 | NA | 0.186207798 | NA | NA | NA | NA |
| Canagliflozin_low_dosage:Liraglutide | 0 | 0.444194843 | NA | 0.444194843 | NA | NA | NA | NA |
| Canagliflozin_low_dosage:Lixisenatide | 0 | -0.0693458 | NA | -0.0693458 | NA | NA | NA | NA |
| Canagliflozin_low_dosage:Oral_semaglutide | 0 | -0.709395349 | NA | -0.709395349 | NA | NA | NA | NA |
| Canagliflozin_low_dosage:Placebo_or_Control | 3 | -0.247633597 | -0.286047782 | 0.505331623 | -0.791379405 | -2.504024884 | 0.921266074 | 0.365115646 |
| Canagliflozin_low_dosage:Sotagliflozin | 0 | -0.160538132 | NA | -0.160538132 | NA | NA | NA | NA |
| Canagliflozin_low_dosage:Tirzepatide | 0 | 0.337072215 | NA | 0.337072215 | NA | NA | NA | NA |

| | | | | | | | | |
|---|---|---|---|---|---|---|---|---|
| Dapagliflozin_high_dosage:Dapagliflozin_low_dosage | 2 | -0.004922942 | -0.125817225 | 0.503435556 | -0.629252782 | -1.966160169 | 0.707654605 | 0.356261698 |
| Dapagliflozin_high_dosage:Dapagliflozin_medium_dosage | 2 | -0.023722883 | -0.140554571 | 0.456903263 | -0.597457833 | -1.918492629 | 0.723576963 | 0.375389545 |
| Dapagliflozin_high_dosage:Dulaglutide | 0 | -0.216146299 | NA | -0.216146299 | NA | NA | NA | NA |
| Dapagliflozin_high_dosage:Efpeglenatide_high_dosage | 0 | -0.222578853 | NA | -0.222578853 | NA | NA | NA | NA |
| Dapagliflozin_high_dosage:Efpeglenatide_medium_dosage | 0 | -0.20275141 | NA | -0.20275141 | NA | NA | NA | NA |
| Dapagliflozin_high_dosage:Empagliflozin_high_dosage | 0 | 0.068121122 | NA | 0.068121122 | NA | NA | NA | NA |
| Dapagliflozin_high_dosage:Empagliflozin_low_dosage | 0 | -0.101607242 | NA | -0.101607242 | NA | NA | NA | NA |
| Dapagliflozin_high_dosage:Ertugliflozin_high_dosage | 0 | -0.157986978 | NA | -0.157986978 | NA | NA | NA | NA |
| Dapagliflozin_high_dosage:Ertugliflozin_low_dosage | 0 | -0.04995463 | NA | -0.04995463 | NA | NA | NA | NA |
| Dapagliflozin_high_dosage:Exenatide | 0 | -0.035245008 | NA | -0.035245008 | NA | NA | NA | NA |
| Dapagliflozin_high_dosage:Inject_semaglutide_high_dosage | 0 | 0.108410465 | NA | 0.108410465 | NA | NA | NA | NA |
| Dapagliflozin_high_dosage:Inject_semaglutide_low_dosage | 0 | 0.257407275 | NA | 0.257407275 | NA | NA | NA | NA |
| Dapagliflozin_high_dosage:Inject_semaglutide_medium_dosage | 0 | 0.19813316 | NA | 0.19813316 | NA | NA | NA | NA |
| Dapagliflozin_high_dosage:Liraglutide | 0 | 0.456120205 | NA | 0.456120205 | NA | NA | NA | NA |
| Dapagliflozin_high_dosage:Lixisenatide | 0 | -0.057420437 | NA | -0.057420437 | NA | NA | NA | NA |
| Dapagliflozin_high_dosage:Oral_semaglutide | 0 | -0.697469987 | NA | -0.697469987 | NA | NA | NA | NA |
| Dapagliflozin_high_dosage:Placebo_or_Control | 6 | -0.235708235 | -0.236268149 | NA | NA | NA | NA | NA |
| Dapagliflozin_high_dosage:Sotagliflozin | 0 | -0.14861277 | NA | -0.14861277 | NA | NA | NA | NA |
| Dapagliflozin_high_dosage:Tirzepatide | 0 | 0.348997577 | NA | 0.348997577 | NA | NA | NA | NA |
| Dapagliflozin_low_dosage:Dapagliflozin_medium_dosage | 2 | -0.018799941 | -0.015094838 | -3.904646189 | 3.889551352 | -14.61426794 | 22.39337064 | 0.680347068 |
| Dapagliflozin_low_dosage:Dulaglutide | 0 | -0.211223358 | NA | -0.211223358 | NA | NA | NA | NA |
| Dapagliflozin_low_dosage:Efpeglenatide_high_dosage | 0 | -0.217655912 | NA | -0.217655912 | NA | NA | NA | NA |

| | | | | | | | | |
|---|---|---|---|---|---|---|---|---|
| Dapagliflozin_low_dosage:Efpeglenatide_medium_dosage | 0 | -0.197828468 | NA | -0.197828468 | NA | NA | NA | NA |
| Dapagliflozin_low_dosage:Empagliflozin_high_dosage | 0 | 0.073044063 | NA | 0.073044063 | NA | NA | NA | NA |
| Dapagliflozin_low_dosage:Empagliflozin_low_dosage | 0 | -0.0966843 | NA | -0.0966843 | NA | NA | NA | NA |
| Dapagliflozin_low_dosage:Ertugliflozin_high_dosage | 0 | -0.153064037 | NA | -0.153064037 | NA | NA | NA | NA |
| Dapagliflozin_low_dosage:Ertugliflozin_low_dosage | 0 | -0.045031688 | NA | -0.045031688 | NA | NA | NA | NA |
| Dapagliflozin_low_dosage:Exenatide | 0 | -0.030322066 | NA | -0.030322066 | NA | NA | NA | NA |
| Dapagliflozin_low_dosage:Inject_semaglutide_high_dosage | 0 | 0.113333407 | NA | 0.113333407 | NA | NA | NA | NA |
| Dapagliflozin_low_dosage:Inject_semaglutide_low_dosage | 0 | 0.262330216 | NA | 0.262330216 | NA | NA | NA | NA |
| Dapagliflozin_low_dosage:Inject_semaglutide_medium_dosage | 0 | 0.203056101 | NA | 0.203056101 | NA | NA | NA | NA |
| Dapagliflozin_low_dosage:Liraglutide | 0 | 0.461043146 | NA | 0.461043146 | NA | NA | NA | NA |
| Dapagliflozin_low_dosage:Lixisenatide | 0 | -0.052497496 | NA | -0.052497496 | NA | NA | NA | NA |
| Dapagliflozin_low_dosage:Oral_semaglutide | 0 | -0.692547046 | NA | -0.692547046 | NA | NA | NA | NA |
| Dapagliflozin_low_dosage:Placebo_or_Control | 2 | -0.230785294 | -0.32489795 | 0.369058973 | -0.693956923 | -2.197322277 | 0.809408431 | 0.365611673 |
| Dapagliflozin_low_dosage:Sotagliflozin | 0 | -0.143689828 | NA | -0.143689828 | NA | NA | NA | NA |
| Dapagliflozin_low_dosage:Tirzepatide | 0 | 0.353920519 | NA | 0.353920519 | NA | NA | NA | NA |
| Dapagliflozin_medium_dosage:Dulaglutide | 0 | -0.192423417 | NA | -0.192423417 | NA | NA | NA | NA |
| Dapagliflozin_medium_dosage:Efpeglenatide_high_dosage | 0 | -0.198855971 | NA | -0.198855971 | NA | NA | NA | NA |
| Dapagliflozin_medium_dosage:Efpeglenatide_medium_dosage | 0 | -0.179028527 | NA | -0.179028527 | NA | NA | NA | NA |
| Dapagliflozin_medium_dosage:Empagliflozin_high_dosage | 0 | 0.091844004 | NA | 0.091844004 | NA | NA | NA | NA |
| Dapagliflozin_medium_dosage:Empagliflozin_low_dosage | 0 | -0.077884359 | NA | -0.077884359 | NA | NA | NA | NA |
| Dapagliflozin_medium_dosage:Ertugliflozin_high_dosage | 0 | -0.134264095 | NA | -0.134264095 | NA | NA | NA | NA |
| Dapagliflozin_medium_dosage:Ertugliflozin_low_dosage | 0 | -0.026231747 | NA | -0.026231747 | NA | NA | NA | NA |

| | | | | | | | | |
|---|---|---|---|---|---|---|---|---|
| Dapagliflozin_medium_dosage:Exenatide | 0 | -0.011522125 | NA | -0.011522125 | NA | NA | NA | NA |
| Dapagliflozin_medium_dosage:Inject_semaglutide_high_dosage | 0 | 0.132133348 | NA | 0.132133348 | NA | NA | NA | NA |
| Dapagliflozin_medium_dosage:Inject_semaglutide_low_dosage | 0 | 0.281130157 | NA | 0.281130157 | NA | NA | NA | NA |
| Dapagliflozin_medium_dosage:Inject_semaglutide_medium_dosage | 0 | 0.221856042 | NA | 0.221856042 | NA | NA | NA | NA |
| Dapagliflozin_medium_dosage:Liraglutide | 0 | 0.479843087 | NA | 0.479843087 | NA | NA | NA | NA |
| Dapagliflozin_medium_dosage:Lixisenatide | 0 | -0.033697555 | NA | -0.033697555 | NA | NA | NA | NA |
| Dapagliflozin_medium_dosage:Oral_semaglutide | 0 | -0.673747105 | NA | -0.673747105 | NA | NA | NA | NA |
| Dapagliflozin_medium_dosage:Placebo_or_Control | 2 | -0.211985352 | -0.310499554 | 0.405953157 | -0.716452711 | -2.202669868 | 0.769764447 | 0.344746033 |
| Dapagliflozin_medium_dosage:Sotagliflozin | 0 | -0.124889887 | NA | -0.124889887 | NA | NA | NA | NA |
| Dapagliflozin_medium_dosage:Tirzepatide | 0 | 0.37272046 | NA | 0.37272046 | NA | NA | NA | NA |
| Dulaglutide:Efpeglenatide_high_dosage | 0 | -0.006432554 | NA | -0.006432554 | NA | NA | NA | NA |
| Dulaglutide:Efpeglenatide_medium_dosage | 0 | 0.01339489 | NA | 0.01339489 | NA | NA | NA | NA |
| Dulaglutide:Empagliflozin_high_dosage | 0 | 0.284267421 | NA | 0.284267421 | NA | NA | NA | NA |
| Dulaglutide:Empagliflozin_low_dosage | 0 | 0.114539058 | NA | 0.114539058 | NA | NA | NA | NA |
| Dulaglutide:Ertugliflozin_high_dosage | 0 | 0.058159321 | NA | 0.058159321 | NA | NA | NA | NA |
| Dulaglutide:Ertugliflozin_low_dosage | 0 | 0.16619167 | NA | 0.16619167 | NA | NA | NA | NA |
| Dulaglutide:Exenatide | 0 | 0.180901291 | NA | 0.180901291 | NA | NA | NA | NA |
| Dulaglutide:Inject_semaglutide_high_dosage | 0 | 0.324556765 | NA | 0.324556765 | NA | NA | NA | NA |
| Dulaglutide:Inject_semaglutide_low_dosage | 0 | 0.473553574 | NA | 0.473553574 | NA | NA | NA | NA |
| Dulaglutide:Inject_semaglutide_medium_dosage | 0 | 0.414279459 | NA | 0.414279459 | NA | NA | NA | NA |
| Dulaglutide:Liraglutide | 0 | 0.672266504 | NA | 0.672266504 | NA | NA | NA | NA |
| Dulaglutide:Lixisenatide | 0 | 0.158725862 | NA | 0.158725862 | NA | NA | NA | NA |

| | | | | | | | | |
|---|---|---|---|---|---|---|---|---|
| Dulaglutide:Oral_semaglutide | 0 | -0.481323688 | NA | -0.481323688 | NA | NA | NA | NA |
| Dulaglutide:Placebo_or_Control | 3 | -0.019561936 | -0.019561936 | NA | NA | NA | NA | NA |
| Dulaglutide:Sotagliflozin | 0 | 0.06753353 | NA | 0.06753353 | NA | NA | NA | NA |
| Dulaglutide:Tirzepatide | 0 | 0.565143876 | NA | 0.565143876 | NA | NA | NA | NA |
| Efpeglenatide_high_dosage:Efpeglenatide_medium_dosage | 1 | 0.019827444 | 0.019827444 | NA | NA | NA | NA | NA |
| Efpeglenatide_high_dosage:Empagliflozin_high_dosage | 0 | 0.290699975 | NA | 0.290699975 | NA | NA | NA | NA |
| Efpeglenatide_high_dosage:Empagliflozin_low_dosage | 0 | 0.120971612 | NA | 0.120971612 | NA | NA | NA | NA |
| Efpeglenatide_high_dosage:Ertugliflozin_high_dosage | 0 | 0.064591875 | NA | 0.064591875 | NA | NA | NA | NA |
| Efpeglenatide_high_dosage:Ertugliflozin_low_dosage | 0 | 0.172624224 | NA | 0.172624224 | NA | NA | NA | NA |
| Efpeglenatide_high_dosage:Exenatide | 0 | 0.187333845 | NA | 0.187333845 | NA | NA | NA | NA |
| Efpeglenatide_high_dosage:Inject_semaglutide_high_dosage | 0 | 0.330989319 | NA | 0.330989319 | NA | NA | NA | NA |
| Efpeglenatide_high_dosage:Inject_semaglutide_low_dosage | 0 | 0.479986128 | NA | 0.479986128 | NA | NA | NA | NA |
| Efpeglenatide_high_dosage:Inject_semaglutide_medium_dosage | 0 | 0.420712013 | NA | 0.420712013 | NA | NA | NA | NA |
| Efpeglenatide_high_dosage:Liraglutide | 0 | 0.678699058 | NA | 0.678699058 | NA | NA | NA | NA |
| Efpeglenatide_high_dosage:Lixisenatide | 0 | 0.165158416 | NA | 0.165158416 | NA | NA | NA | NA |
| Efpeglenatide_high_dosage:Oral_semaglutide | 0 | -0.474891134 | NA | -0.474891134 | NA | NA | NA | NA |
| Efpeglenatide_high_dosage:Placebo_or_Control | 1 | -0.013129382 | -0.013129382 | NA | NA | NA | NA | NA |
| Efpeglenatide_high_dosage:Sotagliflozin | 0 | 0.073966083 | NA | 0.073966083 | NA | NA | NA | NA |
| Efpeglenatide_high_dosage:Tirzepatide | 0 | 0.57157643 | NA | 0.57157643 | NA | NA | NA | NA |
| Efpeglenatide_medium_dosage:Empagliflozin_high_dosage | 0 | 0.270872531 | NA | 0.270872531 | NA | NA | NA | NA |
| Efpeglenatide_medium_dosage:Empagliflozin_low_dosage | 0 | 0.101144168 | NA | 0.101144168 | NA | NA | NA | NA |
| Efpeglenatide_medium_dosage:Ertugliflozin_high_dosage | 0 | 0.044764432 | NA | 0.044764432 | NA | NA | NA | NA |

| | | | | | | | | |
|---|---|---|---|---|---|---|---|---|
| Efpeglenatide_medium_dosage:Ertugliflozin_low_dosage | 0 | 0.15279678 | NA | 0.15279678 | NA | NA | NA | NA |
| Efpeglenatide_medium_dosage:Exenatide | 0 | 0.167506402 | NA | 0.167506402 | NA | NA | NA | NA |
| Efpeglenatide_medium_dosage:Inject_semaglutide_high_dosage | 0 | 0.311161875 | NA | 0.311161875 | NA | NA | NA | NA |
| Efpeglenatide_medium_dosage:Inject_semaglutide_low_dosage | 0 | 0.460158684 | NA | 0.460158684 | NA | NA | NA | NA |
| Efpeglenatide_medium_dosage:Inject_semaglutide_medium_dosage | 0 | 0.400884569 | NA | 0.400884569 | NA | NA | NA | NA |
| Efpeglenatide_medium_dosage:Liraglutide | 0 | 0.658871614 | NA | 0.658871614 | NA | NA | NA | NA |
| Efpeglenatide_medium_dosage:Lixisenatide | 0 | 0.145330972 | NA | 0.145330972 | NA | NA | NA | NA |
| Efpeglenatide_medium_dosage:Oral_semaglutide | 0 | -0.494718578 | NA | -0.494718578 | NA | NA | NA | NA |
| Efpeglenatide_medium_dosage:Placebo_or_Control | 1 | -0.032956825 | -0.032956825 | NA | NA | NA | NA | NA |
| Efpeglenatide_medium_dosage:Sotagliflozin | 0 | 0.05413864 | NA | 0.05413864 | NA | NA | NA | NA |
| Efpeglenatide_medium_dosage:Tirzepatide | 0 | 0.551748987 | NA | 0.551748987 | NA | NA | NA | NA |
| Empagliflozin_high_dosage:Empagliflozin_low_dosage | 4 | -0.169728363 | -0.144660927 | -0.231308844 | 0.086647918 | -0.555795035 | 0.72909087 | 0.791513829 |
| Empagliflozin_high_dosage:Ertugliflozin_high_dosage | 0 | -0.2261081 | NA | -0.2261081 | NA | NA | NA | NA |
| Empagliflozin_high_dosage:Ertugliflozin_low_dosage | 0 | -0.118075751 | NA | -0.118075751 | NA | NA | NA | NA |
| Empagliflozin_high_dosage:Exenatide | 0 | -0.10336613 | NA | -0.10336613 | NA | NA | NA | NA |
| Empagliflozin_high_dosage:Inject_semaglutide_high_dosage | 0 | 0.040289344 | NA | 0.040289344 | NA | NA | NA | NA |
| Empagliflozin_high_dosage:Inject_semaglutide_low_dosage | 0 | 0.189286153 | NA | 0.189286153 | NA | NA | NA | NA |
| Empagliflozin_high_dosage:Inject_semaglutide_medium_dosage | 0 | 0.130012038 | NA | 0.130012038 | NA | NA | NA | NA |
| Empagliflozin_high_dosage:Liraglutide | 0 | 0.387999083 | NA | 0.387999083 | NA | NA | NA | NA |
| Empagliflozin_high_dosage:Lixisenatide | 0 | -0.125541559 | NA | -0.125541559 | NA | NA | NA | NA |
| Empagliflozin_high_dosage:Oral_semaglutide | 0 | -0.765591109 | NA | -0.765591109 | NA | NA | NA | NA |
| Empagliflozin_high_dosage:Placebo_or_Control | 5 | -0.303829357 | -0.334989889 | -0.072958996 | -0.262030893 | -1.110046623 | 0.585984837 | 0.544770387 |

| | | | | | | | | |
|---|---|---|---|---|---|---|---|---|
| Empagliflozin_high_dosage:Sotagliflozin | 0 | -0.216733892 | NA | -0.216733892 | NA | NA | NA | NA |
| Empagliflozin_high_dosage:Tirzepatide | 0 | 0.280876455 | NA | 0.280876455 | NA | NA | NA | NA |
| Empagliflozin_low_dosage:Ertugliflozin_high_dosage | 0 | -0.056379737 | NA | -0.056379737 | NA | NA | NA | NA |
| Empagliflozin_low_dosage:Ertugliflozin_low_dosage | 0 | 0.051652612 | NA | 0.051652612 | NA | NA | NA | NA |
| Empagliflozin_low_dosage:Exenatide | 0 | 0.066362234 | NA | 0.066362234 | NA | NA | NA | NA |
| Empagliflozin_low_dosage:Inject_semaglutide_high_dosage | 0 | 0.210017707 | NA | 0.210017707 | NA | NA | NA | NA |
| Empagliflozin_low_dosage:Inject_semaglutide_low_dosage | 0 | 0.359014516 | NA | 0.359014516 | NA | NA | NA | NA |
| Empagliflozin_low_dosage:Inject_semaglutide_medium_dosage | 0 | 0.299740401 | NA | 0.299740401 | NA | NA | NA | NA |
| Empagliflozin_low_dosage:Liraglutide | 0 | 0.557727446 | NA | 0.557727446 | NA | NA | NA | NA |
| Empagliflozin_low_dosage:Lixisenatide | 0 | 0.044186804 | NA | 0.044186804 | NA | NA | NA | NA |
| Empagliflozin_low_dosage:Oral_semaglutide | 0 | -0.595862746 | NA | -0.595862746 | NA | NA | NA | NA |
| Empagliflozin_low_dosage:Placebo_or_Control | 7 | -0.134100994 | -0.149181969 | 0.26090661 | -0.410088578 | -1.532385362 | 0.712208205 | 0.473884428 |
| Empagliflozin_low_dosage:Sotagliflozin | 0 | -0.047005528 | NA | -0.047005528 | NA | NA | NA | NA |
| Empagliflozin_low_dosage:Tirzepatide | 0 | 0.450604819 | NA | 0.450604819 | NA | NA | NA | NA |
| Ertugliflozin_high_dosage:Ertugliflozin_low_dosage | 3 | 0.108032348 | 0.110893931 | NA | NA | NA | NA | NA |
| Ertugliflozin_high_dosage:Exenatide | 0 | 0.12274197 | NA | 0.12274197 | NA | NA | NA | NA |
| Ertugliflozin_high_dosage:Inject_semaglutide_high_dosage | 0 | 0.266397444 | NA | 0.266397444 | NA | NA | NA | NA |
| Ertugliflozin_high_dosage:Inject_semaglutide_low_dosage | 0 | 0.415394253 | NA | 0.415394253 | NA | NA | NA | NA |
| Ertugliflozin_high_dosage:Inject_semaglutide_medium_dosage | 0 | 0.356120138 | NA | 0.356120138 | NA | NA | NA | NA |
| Ertugliflozin_high_dosage:Liraglutide | 0 | 0.614107183 | NA | 0.614107183 | NA | NA | NA | NA |
| Ertugliflozin_high_dosage:Lixisenatide | 0 | 0.100566541 | NA | 0.100566541 | NA | NA | NA | NA |
| Ertugliflozin_high_dosage:Oral_semaglutide | 0 | -0.539483009 | NA | -0.539483009 | NA | NA | NA | NA |

| | | | | | | | | |
|---|---|---|---|---|---|---|---|---|
| Ertugliflozin_high_dosage:Placebo_or_Control | 3 | -0.077721257 | -0.072871969 | -5.99113997 | 5.918268 | -7.001479365 | 18.83801537 | 0.369282158 |
| Ertugliflozin_high_dosage:Sotagliflozin | 0 | 0.009374208 | NA | 0.009374208 | NA | NA | NA | NA |
| Ertugliflozin_high_dosage:Tirzepatide | 0 | 0.506984555 | NA | 0.506984555 | NA | NA | NA | NA |
| Ertugliflozin_low_dosage:Exenatide | 0 | 0.014709622 | NA | 0.014709622 | NA | NA | NA | NA |
| Ertugliflozin_low_dosage:Inject_semaglutide_high_dosage | 0 | 0.158365095 | NA | 0.158365095 | NA | NA | NA | NA |
| Ertugliflozin_low_dosage:Inject_semaglutide_low_dosage | 0 | 0.307361904 | NA | 0.307361904 | NA | NA | NA | NA |
| Ertugliflozin_low_dosage:Inject_semaglutide_medium_dosage | 0 | 0.248087789 | NA | 0.248087789 | NA | NA | NA | NA |
| Ertugliflozin_low_dosage:Liraglutide | 0 | 0.506074834 | NA | 0.506074834 | NA | NA | NA | NA |
| Ertugliflozin_low_dosage:Lixisenatide | 0 | -0.007465808 | NA | -0.007465808 | NA | NA | NA | NA |
| Ertugliflozin_low_dosage:Oral_semaglutide | 0 | -0.647515358 | NA | -0.647515358 | NA | NA | NA | NA |
| Ertugliflozin_low_dosage:Placebo_or_Control | 3 | -0.185753605 | -0.18288228 | -1.999705135 | 1.816822854 | -7.592634423 | 11.22628013 | 0.705104362 |
| Ertugliflozin_low_dosage:Sotagliflozin | 0 | -0.09865814 | NA | -0.09865814 | NA | NA | NA | NA |
| Ertugliflozin_low_dosage:Tirzepatide | 0 | 0.398952207 | NA | 0.398952207 | NA | NA | NA | NA |
| Exenatide:Inject_semaglutide_high_dosage | 0 | 0.143655473 | NA | 0.143655473 | NA | NA | NA | NA |
| Exenatide:Inject_semaglutide_low_dosage | 0 | 0.292652283 | NA | 0.292652283 | NA | NA | NA | NA |
| Exenatide:Inject_semaglutide_medium_dosage | 0 | 0.233378167 | NA | 0.233378167 | NA | NA | NA | NA |
| Exenatide:Liraglutide | 1 | 0.491365213 | 0.387314009 | 0.566854883 | -0.179540874 | -1.061732058 | 0.70265031 | 0.68997726 |
| Exenatide:Lixisenatide | 0 | -0.02217543 | NA | -0.02217543 | NA | NA | NA | NA |
| Exenatide:Oral_semaglutide | 0 | -0.662224979 | NA | -0.662224979 | NA | NA | NA | NA |
| Exenatide:Placebo_or_Control | 1 | -0.200463227 | -0.145529276 | -0.32507015 | 0.179540874 | -0.70265031 | 1.061732058 | 0.68997726 |
| Exenatide:Sotagliflozin | 0 | -0.113367762 | NA | -0.113367762 | NA | NA | NA | NA |
| Exenatide:Tirzepatide | 0 | 0.384242585 | NA | 0.384242585 | NA | NA | NA | NA |

| | | | | | | | | |
|---|---|---|---|---|---|---|---|---|
| Inject_semaglutide_high_dosage:Inject_semaglutide_low_dosage | 0 | 0.148996809 | NA | 0.148996809 | NA | NA | NA | NA |
| Inject_semaglutide_high_dosage:Inject_semaglutide_medium_dosage | 1 | 0.089722694 | -0.002560821 | 0.127735235 | -0.130296056 | -1.206533668 | 0.945941556 | 0.812435312 |
| Inject_semaglutide_high_dosage:Liraglutide | 0 | 0.347709739 | NA | 0.347709739 | NA | NA | NA | NA |
| Inject_semaglutide_high_dosage:Lixisenatide | 0 | -0.165830903 | NA | -0.165830903 | NA | NA | NA | NA |
| Inject_semaglutide_high_dosage:Oral_semaglutide | 0 | -0.805880453 | NA | -0.805880453 | NA | NA | NA | NA |
| Inject_semaglutide_high_dosage:Placebo_or_Control | 4 | -0.344118701 | -0.341954133 | -0.445058393 | 0.103104259 | -2.034115557 | 2.240324076 | 0.92466985 |
| Inject_semaglutide_high_dosage:Sotagliflozin | 0 | -0.257023235 | NA | -0.257023235 | NA | NA | NA | NA |
| Inject_semaglutide_high_dosage:Tirzepatide | 0 | 0.240587112 | NA | 0.240587112 | NA | NA | NA | NA |
| Inject_semaglutide_low_dosage:Inject_semaglutide_medium_dosage | 2 | -0.059274115 | -0.063383938 | -0.041430685 | -0.021953253 | -1.666245889 | 1.622339383 | 0.979123468 |
| Inject_semaglutide_low_dosage:Liraglutide | 0 | 0.19871293 | NA | 0.19871293 | NA | NA | NA | NA |
| Inject_semaglutide_low_dosage:Lixisenatide | 0 | -0.314827712 | NA | -0.314827712 | NA | NA | NA | NA |
| Inject_semaglutide_low_dosage:Oral_semaglutide | 0 | -0.954877262 | NA | -0.954877262 | NA | NA | NA | NA |
| Inject_semaglutide_low_dosage:Placebo_or_Control | 2 | -0.49311551 | -0.462250586 | -0.750429108 | 0.288178522 | -1.704209858 | 2.280566903 | 0.776802268 |
| Inject_semaglutide_low_dosage:Sotagliflozin | 0 | -0.406020044 | NA | -0.406020044 | NA | NA | NA | NA |
| Inject_semaglutide_low_dosage:Tirzepatide | 0 | 0.091590303 | NA | 0.091590303 | NA | NA | NA | NA |
| Inject_semaglutide_medium_dosage:Liraglutide | 0 | 0.257987045 | NA | 0.257987045 | NA | NA | NA | NA |
| Inject_semaglutide_medium_dosage:Lixisenatide | 0 | -0.255553597 | NA | -0.255553597 | NA | NA | NA | NA |
| Inject_semaglutide_medium_dosage:Oral_semaglutide | 0 | -0.895603147 | NA | -0.895603147 | NA | NA | NA | NA |
| Inject_semaglutide_medium_dosage:Placebo_or_Control | 3 | -0.433841395 | -0.459685667 | -0.39165066 | -0.068035006 | -0.913435386 | 0.777365373 | 0.874668478 |
| Inject_semaglutide_medium_dosage:Sotagliflozin | 0 | -0.346745929 | NA | -0.346745929 | NA | NA | NA | NA |
| Inject_semaglutide_medium_dosage:Tirzepatide | 1 | 0.150864418 | 0.116456419 | 0.178646213 | -0.062189794 | -0.937463265 | 0.813083677 | 0.889245444 |
| Liraglutide:Lixisenatide | 1 | -0.513540642 | -0.092260733 | -0.682291934 | 0.590031201 | -0.544715071 | 1.724777474 | 0.308147137 |

| | | | | | | | | |
|---|---|---|---|---|---|---|---|---|
| Liraglutide:Oral_semaglutide | 0 | -1.153590192 | NA | -1.153590192 | NA | NA | NA | NA |
| Liraglutide:Placebo_or_Control | 3 | -0.69182844 | -0.75812291 | -0.389956771 | -0.368166139 | -1.099200875 | 0.362868597 | 0.323601674 |
| Liraglutide:Sotagliflozin | 0 | -0.604732974 | NA | -0.604732974 | NA | NA | NA | NA |
| Liraglutide:Tirzepatide | 0 | -0.107122628 | NA | -0.107122628 | NA | NA | NA | NA |
| Lixisenatide:Oral_semaglutide | 0 | -0.64004955 | NA | -0.64004955 | NA | NA | NA | NA |
| Lixisenatide:Placebo_or_Control | 1 | -0.178287798 | -0.048228542 | -0.638259743 | 0.590031201 | -0.544715071 | 1.724777474 | 0.308147137 |
| Lixisenatide:Sotagliflozin | 0 | -0.091192332 | NA | -0.091192332 | NA | NA | NA | NA |
| Lixisenatide:Tirzepatide | 0 | 0.406418015 | NA | 0.406418015 | NA | NA | NA | NA |
| Oral_semaglutide:Placebo_or_Control | 2 | 0.461761752 | 0.461761752 | NA | NA | NA | NA | NA |
| Oral_semaglutide:Sotagliflozin | 0 | 0.548857217 | NA | 0.548857217 | NA | NA | NA | NA |
| Oral_semaglutide:Tirzepatide | 0 | 1.046467564 | NA | 1.046467564 | NA | NA | NA | NA |
| Sotagliflozin:Placebo_or_Control | 4 | -0.087095465 | -0.087095465 | NA | NA | NA | NA | NA |
| Tirzepatide:Placebo_or_Control | 4 | -0.584705812 | -0.592206827 | -0.530017033 | -0.062189794 | -0.937463265 | 0.813083677 | 0.889245444 |
| Sotagliflozin:Tirzepatide | 0 | 0.497610347 | NA | 0.497610347 | NA | NA | NA | NA |

*Abbreviation: 95%CIs: 95% confidence intervals; GLP-1 agonist: glucagon-like peptide-1 agonist; NA: not applicable; NMA: network meta-analysis; OR: odds ratio; RCT: randomized controlled trial; SGLT2 inhibitor: sodium–glucose cotransporter 2 inhibitor*

## Table S8A: GRADE of primary outcome: overall hematologic malignancy

| | Comparison | No.Studies | Direct | | Indirect | | NMA | |
|---|---|---|---|---|---|---|---|---|
| | | | Estimate | Rate | Estimate | Rate | Estimate | Rate |
| 1 | Albiglutide:Bexagliflozin | 0 | | | 0.062872591 | ⊕⊕○○ Low | 0.062872591 | ⊕⊕○○ Low |
| 2 | Albiglutide:Canagliflozin_high_dosage | 0 | | | 1.269240461 | ⊕⊕○○ Low | 1.269240461 | ⊕⊕○○ Low |
| 3 | Albiglutide:Canagliflozin_low_dosage | 0 | | | 0.829915896 | ⊕⊕○○ Low | 0.829915896 | ⊕⊕○○ Low |
| 4 | Albiglutide:Dapagliflozin_high_dosage | 0 | | | 0.332419503 | ⊕⊕○○ Low | 0.332419503 | ⊕⊕○○ Low |
| 5 | Albiglutide:Dapagliflozin_low_dosage | 0 | | | 1.588736549 | ⊕⊕○○ Low | 1.588736549 | ⊕⊕○○ Low |
| 6 | Albiglutide:Dapagliflozin_medium_dosage | 0 | | | 1.612859704 | ⊕⊕○○ Low | 1.612859704 | ⊕⊕○○ Low |
| 7 | Albiglutide:Dulaglutide | 0 | | | -0.308173616 | ⊕⊕○○ Low | -0.308173616 | ⊕⊕○○ Low |
| 8 | Albiglutide:Efpeglenatide_high_dosage | 0 | | | -0.625711741 | ⊕⊕○○ Low | -0.625711741 | ⊕⊕○○ Low |
| 9 | Albiglutide:Efpeglenatide_medium_dosage | 0 | | | -1.135800988 | ⊕⊕○○ Low | -1.135800988 | ⊕⊕○○ Low |
| 10 | Albiglutide:Empagliflozin_high_dosage | 0 | | | 0.784499934 | ⊕⊕○○ Low | 0.784499934 | ⊕⊕○○ Low |
| 11 | Albiglutide:Empagliflozin_low_dosage | 0 | | | -0.15604213 | ⊕⊕○○ Low | -0.15604213 | ⊕⊕○○ Low |
| 12 | Albiglutide:Ertugliflozin_high_dosage | 0 | | | -0.085710886 | ⊕⊕○○ Low | -0.085710886 | ⊕⊕○○ Low |
| 13 | Albiglutide:Ertugliflozin_low_dosage | 0 | | | 0.297959729 | ⊕⊕○○ Low | 0.297959729 | ⊕⊕○○ Low |
| 14 | Albiglutide:Exenatide | 0 | | | 0.393868739 | ⊕⊕○○ Low | 0.393868739 | ⊕⊕○○ Low |
| 15 | Albiglutide:Inject_semaglutide_high_dosage | 0 | | | 0.432847823 | ⊕⊕○○ Low | 0.432847823 | ⊕⊕○○ Low |
| 16 | Albiglutide:Inject_semaglutide_low_dosage | 0 | | | 1.425818538 | ⊕⊕○○ Low | 1.425818538 | ⊕⊕○○ Low |
| 17 | Albiglutide:Inject_semaglutide_medium_dosage | 0 | | | 1.327328856 | ⊕⊕○○ Low | 1.327328856 | ⊕⊕○○ Low |
| 18 | Albiglutide:Liraglutide | 0 | | | 0.635347908 | ⊕⊕○○ Low | 0.635347908 | ⊕⊕○○ Low |
| 19 | Albiglutide:Lixisenatide | 0 | | | 0.993452682 | ⊕⊕○○ Low | 0.993452682 | ⊕⊕○○ Low |

| | | | | | | | | |
|---|---|---|---|---|---|---|---|---|
| 20 | Albiglutide:Oral_semaglutide | 0 | | | 0.602660348 | ⊕⊕○○ Low | 0.602660348 | ⊕⊕○○ Low |
| 21 | Albiglutide:Placebo_or_Control | 4 | 0.471426164 | ⊕⊕⊕⊕ High | | | 0.471426164 | ⊕⊕⊕○ Medium |
| 22 | Albiglutide:Sotagliflozin | 0 | | | 0.264705656 | ⊕⊕○○ Low | 0.264705656 | ⊕⊕○○ Low |
| 23 | Albiglutide:Tirzepatide | 0 | | | 2.425174515 | ⊕⊕○○ Low | 2.425174515 | ⊕⊕○○ Low |
| 24 | Bexagliflozin:Canagliflozin_high_dosage | 0 | | | 1.206367869 | ⊕⊕○○ Low | 1.206367869 | ⊕⊕○○ Low |
| 25 | Bexagliflozin:Canagliflozin_low_dosage | 0 | | | 0.767043304 | ⊕⊕○○ Low | 0.767043304 | ⊕⊕○○ Low |
| 26 | Bexagliflozin:Dapagliflozin_high_dosage | 0 | | | 0.269546912 | ⊕⊕○○ Low | 0.269546912 | ⊕⊕○○ Low |
| 27 | Bexagliflozin:Dapagliflozin_low_dosage | 0 | | | 1.525863958 | ⊕⊕○○ Low | 1.525863958 | ⊕⊕○○ Low |
| 28 | Bexagliflozin:Dapagliflozin_medium_dosage | 0 | | | 1.549987112 | ⊕⊕○○ Low | 1.549987112 | ⊕⊕○○ Low |
| 29 | Bexagliflozin:Dulaglutide | 0 | | | -0.371046208 | ⊕⊕○○ Low | -0.371046208 | ⊕⊕○○ Low |
| 30 | Bexagliflozin:Efpeglenatide_high_dosage | 0 | | | -0.688584333 | ⊕⊕○○ Low | -0.688584333 | ⊕⊕○○ Low |
| 31 | Bexagliflozin:Efpeglenatide_medium_dosage | 0 | | | -1.19867358 | ⊕⊕○○ Low | -1.19867358 | ⊕⊕○○ Low |
| 32 | Bexagliflozin:Empagliflozin_high_dosage | 0 | | | 0.721627343 | ⊕⊕○○ Low | 0.721627343 | ⊕⊕○○ Low |
| 33 | Bexagliflozin:Empagliflozin_low_dosage | 0 | | | -0.218914722 | ⊕⊕○○ Low | -0.218914722 | ⊕⊕○○ Low |
| 34 | Bexagliflozin:Ertugliflozin_high_dosage | 0 | | | -0.148583478 | ⊕⊕○○ Low | -0.148583478 | ⊕⊕○○ Low |
| 35 | Bexagliflozin:Ertugliflozin_low_dosage | 0 | | | 0.235087138 | ⊕⊕○○ Low | 0.235087138 | ⊕⊕○○ Low |
| 36 | Bexagliflozin:Exenatide | 0 | | | 0.330996147 | ⊕⊕○○ Low | 0.330996147 | ⊕⊕○○ Low |
| 37 | Bexagliflozin:Inject_semaglutide_high_dosage | 0 | | | 0.369975232 | ⊕⊕○○ Low | 0.369975232 | ⊕⊕○○ Low |
| 38 | Bexagliflozin:Inject_semaglutide_low_dosage | 0 | | | 1.362945947 | ⊕⊕○○ Low | 1.362945947 | ⊕⊕○○ Low |
| 39 | Bexagliflozin:Inject_semaglutide_medium_dosage | 0 | | | 1.264456264 | ⊕⊕○○ Low | 1.264456264 | ⊕⊕○○ Low |
| 40 | Bexagliflozin:Liraglutide | 0 | | | 0.572475317 | ⊕⊕○○ Low | 0.572475317 | ⊕⊕○○ Low |
| 41 | Bexagliflozin:Lixisenatide | 0 | | | 0.930580091 | ⊕⊕○○ Low | 0.930580091 | ⊕⊕○○ Low |

| | | | | | | | | |
|---|---|---|---|---|---|---|---|---|
| 42 | Bexagliflozin:Oral_semaglutide | 0 | | | 0.539787757 | ⊕⊕○○ Low | 0.539787757 | ⊕⊕○○ Low |
| 43 | Bexagliflozin:Placebo_or_Control | 1 | 0.408553573 | ⊕⊕⊕⊕ High | | | 0.408553573 | ⊕⊕⊕○ Medium |
| 44 | Bexagliflozin:Sotagliflozin | 0 | | | 0.201833065 | ⊕⊕○○ Low | 0.201833065 | ⊕⊕○○ Low |
| 45 | Bexagliflozin:Tirzepatide | 0 | | | 2.362301923 | ⊕⊕○○ Low | 2.362301923 | ⊕⊕○○ Low |
| 46 | Canagliflozin_high_dosage:Canagliflozin_low_dosage | 1 | -0.285598015 | ⊕⊕⊕⊕ High | -0.888116525 | ⊕⊕⊕⊕ High | -0.439324565 | ⊕⊕⊕⊕ High |
| 47 | Canagliflozin_high_dosage:Dapagliflozin_high_dosage | 0 | | | -0.936820957 | ⊕⊕○○ Low | -0.936820957 | ⊕⊕○○ Low |
| 48 | Canagliflozin_high_dosage:Dapagliflozin_low_dosage | 0 | | | 0.319496088 | ⊕⊕○○ Low | 0.319496088 | ⊕⊕○○ Low |
| 49 | Canagliflozin_high_dosage:Dapagliflozin_medium_dosage | 0 | | | 0.343619243 | ⊕⊕○○ Low | 0.343619243 | ⊕⊕○○ Low |
| 50 | Canagliflozin_high_dosage:Dulaglutide | 0 | | | -1.577414077 | ⊕⊕○○ Low | -1.577414077 | ⊕⊕○○ Low |
| 51 | Canagliflozin_high_dosage:Efpeglenatide_high_dosage | 0 | | | -1.894952202 | ⊕⊕○○ Low | -1.894952202 | ⊕⊕○○ Low |
| 52 | Canagliflozin_high_dosage:Efpeglenatide_medium_dosage | 0 | | | -2.405041449 | ⊕⊕○○ Low | -2.405041449 | ⊕⊕○○ Low |
| 53 | Canagliflozin_high_dosage:Empagliflozin_high_dosage | 0 | | | -0.484740526 | ⊕⊕○○ Low | -0.484740526 | ⊕⊕○○ Low |
| 54 | Canagliflozin_high_dosage:Empagliflozin_low_dosage | 0 | | | -1.425282591 | ⊕⊕○○ Low | -1.425282591 | ⊕⊕○○ Low |
| 55 | Canagliflozin_high_dosage:Ertugliflozin_high_dosage | 0 | | | -1.354951347 | ⊕⊕○○ Low | -1.354951347 | ⊕⊕○○ Low |
| 56 | Canagliflozin_high_dosage:Ertugliflozin_low_dosage | 0 | | | -0.971280732 | ⊕⊕○○ Low | -0.971280732 | ⊕⊕○○ Low |
| 57 | Canagliflozin_high_dosage:Exenatide | 0 | | | -0.875371722 | ⊕⊕○○ Low | -0.875371722 | ⊕⊕○○ Low |
| 58 | Canagliflozin_high_dosage:Inject_semaglutide_high_dosage | 0 | | | -0.836392637 | ⊕⊕○○ Low | -0.836392637 | ⊕⊕○○ Low |
| 59 | Canagliflozin_high_dosage:Inject_semaglutide_low_dosage | 0 | | | 0.156578078 | ⊕⊕○○ Low | 0.156578078 | ⊕⊕○○ Low |
| 60 | Canagliflozin_high_dosage:Inject_semaglutide_medium_dosage | 0 | | | 0.058088395 | ⊕⊕○○ Low | 0.058088395 | ⊕⊕○○ Low |
| 61 | Canagliflozin_high_dosage:Liraglutide | 0 | | | -0.633892552 | ⊕⊕○○ Low | -0.633892552 | ⊕⊕○○ Low |
| 62 | Canagliflozin_high_dosage:Lixisenatide | 0 | | | -0.275787778 | ⊕⊕○○ Low | -0.275787778 | ⊕⊕○○ Low |
| 63 | Canagliflozin_high_dosage:Oral_semaglutide | 0 | | | -0.666580112 | ⊕⊕○○ Low | -0.666580112 | ⊕⊕○○ Low |

| | | | | | | | | |
|---|---|---|---|---|---|---|---|---|
| 64 | Canagliflozin_high_dosage:Placebo_or_Control | 2 | -0.587305465 | ⊕⊕⊕⊕ High | -2.055953315 | ⊕⊕⊕⊕ High | -0.797814296 | ⊕⊕⊕⊕ High |
| 65 | Canagliflozin_high_dosage:Sotagliflozin | 0 | | | -1.004534804 | ⊕⊕○○ Low | -1.004534804 | ⊕⊕○○ Low |
| 66 | Canagliflozin_high_dosage:Tirzepatide | 0 | | | 1.155934054 | ⊕⊕○○ Low | 1.155934054 | ⊕⊕○○ Low |
| 67 | Canagliflozin_low_dosage:Dapagliflozin_high_dosage | 0 | | | -0.497496392 | ⊕⊕○○ Low | -0.497496392 | ⊕⊕○○ Low |
| 68 | Canagliflozin_low_dosage:Dapagliflozin_low_dosage | 0 | | | 0.758820653 | ⊕⊕○○ Low | 0.758820653 | ⊕⊕○○ Low |
| 69 | Canagliflozin_low_dosage:Dapagliflozin_medium_dosage | 0 | | | 0.782943808 | ⊕⊕○○ Low | 0.782943808 | ⊕⊕○○ Low |
| 70 | Canagliflozin_low_dosage:Dulaglutide | 0 | | | -1.138089512 | ⊕⊕○○ Low | -1.138089512 | ⊕⊕○○ Low |
| 71 | Canagliflozin_low_dosage:Efpeglenatide_high_dosage | 0 | | | -1.455627637 | ⊕⊕○○ Low | -1.455627637 | ⊕⊕○○ Low |
| 72 | Canagliflozin_low_dosage:Efpeglenatide_medium_dosage | 0 | | | -1.965716884 | ⊕⊕○○ Low | -1.965716884 | ⊕⊕○○ Low |
| 73 | Canagliflozin_low_dosage:Empagliflozin_high_dosage | 0 | | | -0.045415961 | ⊕⊕○○ Low | -0.045415961 | ⊕⊕○○ Low |
| 74 | Canagliflozin_low_dosage:Empagliflozin_low_dosage | 0 | | | -0.985958026 | ⊕⊕○○ Low | -0.985958026 | ⊕⊕○○ Low |
| 75 | Canagliflozin_low_dosage:Ertugliflozin_high_dosage | 0 | | | -0.915626782 | ⊕⊕○○ Low | -0.915626782 | ⊕⊕○○ Low |
| 76 | Canagliflozin_low_dosage:Ertugliflozin_low_dosage | 0 | | | -0.531956167 | ⊕⊕○○ Low | -0.531956167 | ⊕⊕○○ Low |
| 77 | Canagliflozin_low_dosage:Exenatide | 0 | | | -0.436047157 | ⊕⊕○○ Low | -0.436047157 | ⊕⊕○○ Low |
| 78 | Canagliflozin_low_dosage:Inject_semaglutide_high_dosage | 0 | | | -0.397068073 | ⊕⊕○○ Low | -0.397068073 | ⊕⊕○○ Low |
| 79 | Canagliflozin_low_dosage:Inject_semaglutide_low_dosage | 0 | | | 0.595902642 | ⊕⊕○○ Low | 0.595902642 | ⊕⊕○○ Low |
| 80 | Canagliflozin_low_dosage:Inject_semaglutide_medium_dosage | 0 | | | 0.49741296 | ⊕⊕○○ Low | 0.49741296 | ⊕⊕○○ Low |
| 81 | Canagliflozin_low_dosage:Liraglutide | 0 | | | -0.194567987 | ⊕⊕○○ Low | -0.194567987 | ⊕⊕○○ Low |
| 82 | Canagliflozin_low_dosage:Lixisenatide | 0 | | | 0.163536786 | ⊕⊕○○ Low | 0.163536786 | ⊕⊕○○ Low |
| 83 | Canagliflozin_low_dosage:Oral_semaglutide | 0 | | | -0.227255548 | ⊕⊕○○ Low | -0.227255548 | ⊕⊕○○ Low |
| 84 | Canagliflozin_low_dosage:Placebo_or_Control | 3 | -0.13765041 | ⊕⊕⊕⊕ High | -2.841598052 | ⊕⊕⊕⊕ High | -0.358489731 | ⊕⊕⊕⊕ High |
| 85 | Canagliflozin_low_dosage:Sotagliflozin | 0 | | | -0.565210239 | ⊕⊕○○ Low | -0.565210239 | ⊕⊕○○ Low |

| | | | | | | | | |
|---|---|---|---|---|---|---|---|---|
| 86 | Canagliflozin_low_dosage:Tirzepatide | 0 | | | 1.595258619 | ⊕⊕○○ Low | 1.595258619 | ⊕⊕○○ Low |
| 87 | Dapagliflozin_high_dosage:Dapagliflozin_low_dosage | 0 | | | 1.256317046 | ⊕⊕○○ Low | 1.256317046 | ⊕⊕○○ Low |
| 88 | Dapagliflozin_high_dosage:Dapagliflozin_medium_dosage | 0 | | | 1.2804402 | ⊕⊕○○ Low | 1.2804402 | ⊕⊕○○ Low |
| 89 | Dapagliflozin_high_dosage:Dulaglutide | 0 | | | -0.64059312 | ⊕⊕○○ Low | -0.64059312 | ⊕⊕○○ Low |
| 90 | Dapagliflozin_high_dosage:Efpeglenatide_high_dosage | 0 | | | -0.958131245 | ⊕⊕○○ Low | -0.958131245 | ⊕⊕○○ Low |
| 91 | Dapagliflozin_high_dosage:Efpeglenatide_medium_dosage | 0 | | | -1.468220492 | ⊕⊕○○ Low | -1.468220492 | ⊕⊕○○ Low |
| 92 | Dapagliflozin_high_dosage:Empagliflozin_high_dosage | 0 | | | 0.452080431 | ⊕⊕○○ Low | 0.452080431 | ⊕⊕○○ Low |
| 93 | Dapagliflozin_high_dosage:Empagliflozin_low_dosage | 0 | | | -0.488461634 | ⊕⊕○○ Low | -0.488461634 | ⊕⊕○○ Low |
| 94 | Dapagliflozin_high_dosage:Ertugliflozin_high_dosage | 0 | | | -0.41813039 | ⊕⊕○○ Low | -0.41813039 | ⊕⊕○○ Low |
| 95 | Dapagliflozin_high_dosage:Ertugliflozin_low_dosage | 0 | | | -0.034459775 | ⊕⊕○○ Low | -0.034459775 | ⊕⊕○○ Low |
| 96 | Dapagliflozin_high_dosage:Exenatide | 0 | | | 0.061449235 | ⊕⊕○○ Low | 0.061449235 | ⊕⊕○○ Low |
| 97 | Dapagliflozin_high_dosage:Inject_semaglutide_high_dosage | 0 | | | 0.10042832 | ⊕⊕○○ Low | 0.10042832 | ⊕⊕○○ Low |
| 98 | Dapagliflozin_high_dosage:Inject_semaglutide_low_dosage | 0 | | | 1.093399035 | ⊕⊕○○ Low | 1.093399035 | ⊕⊕○○ Low |
| 99 | Dapagliflozin_high_dosage:Inject_semaglutide_medium_dosage | 0 | | | 0.994909352 | ⊕⊕○○ Low | 0.994909352 | ⊕⊕○○ Low |
| 100 | Dapagliflozin_high_dosage:Liraglutide | 0 | | | 0.302928405 | ⊕⊕○○ Low | 0.302928405 | ⊕⊕○○ Low |
| 101 | Dapagliflozin_high_dosage:Lixisenatide | 0 | | | 0.661033179 | ⊕⊕○○ Low | 0.661033179 | ⊕⊕○○ Low |
| 102 | Dapagliflozin_high_dosage:Oral_semaglutide | 0 | | | 0.270240845 | ⊕⊕○○ Low | 0.270240845 | ⊕⊕○○ Low |
| 103 | Dapagliflozin_high_dosage:Placebo_or_Control | 6 | 0.139006661 | ⊕⊕⊕⊕ High | | | 0.139006661 | ⊕⊕⊕○ Medium |
| 104 | Dapagliflozin_high_dosage:Sotagliflozin | 0 | | | -0.067713847 | ⊕⊕○○ Low | -0.067713847 | ⊕⊕○○ Low |
| 105 | Dapagliflozin_high_dosage:Tirzepatide | 0 | | | 2.092755011 | ⊕⊕○○ Low | 2.092755011 | ⊕⊕○○ Low |
| 106 | Dapagliflozin_low_dosage:Dapagliflozin_medium_dosage | 0 | | | 0.024123155 | ⊕⊕○○ Low | 0.024123155 | ⊕⊕○○ Low |
| 107 | Dapagliflozin_low_dosage:Dulaglutide | 0 | | | -1.896910165 | ⊕⊕○○ Low | -1.896910165 | ⊕⊕○○ Low |

| | | | | | | | | |
|---|---|---|---|---|---|---|---|---|
| 108 | Dapagliflozin_low_dosage:Efpeglenatide_high_dosage | 0 | | | -2.21444829 | ⊕⊕○○ Low | -2.21444829 | ⊕⊕○○ Low |
| 109 | Dapagliflozin_low_dosage:Efpeglenatide_medium_dosage | 0 | | | -2.724537537 | ⊕⊕○○ Low | -2.724537537 | ⊕⊕○○ Low |
| 110 | Dapagliflozin_low_dosage:Empagliflozin_high_dosage | 0 | | | -0.804236615 | ⊕⊕○○ Low | -0.804236615 | ⊕⊕○○ Low |
| 111 | Dapagliflozin_low_dosage:Empagliflozin_low_dosage | 0 | | | -1.744778679 | ⊕⊕○○ Low | -1.744778679 | ⊕⊕○○ Low |
| 112 | Dapagliflozin_low_dosage:Ertugliflozin_high_dosage | 0 | | | -1.674447435 | ⊕⊕○○ Low | -1.674447435 | ⊕⊕○○ Low |
| 113 | Dapagliflozin_low_dosage:Ertugliflozin_low_dosage | 0 | | | -1.29077682 | ⊕⊕○○ Low | -1.29077682 | ⊕⊕○○ Low |
| 114 | Dapagliflozin_low_dosage:Exenatide | 0 | | | -1.19486781 | ⊕⊕○○ Low | -1.19486781 | ⊕⊕○○ Low |
| 115 | Dapagliflozin_low_dosage:Inject_semaglutide_high_dosage | 0 | | | -1.155888726 | ⊕⊕○○ Low | -1.155888726 | ⊕⊕○○ Low |
| 116 | Dapagliflozin_low_dosage:Inject_semaglutide_low_dosage | 0 | | | -0.162918011 | ⊕⊕○○ Low | -0.162918011 | ⊕⊕○○ Low |
| 117 | Dapagliflozin_low_dosage:Inject_semaglutide_medium_dosage | 0 | | | -0.261407694 | ⊕⊕○○ Low | -0.261407694 | ⊕⊕○○ Low |
| 118 | Dapagliflozin_low_dosage:Liraglutide | 0 | | | -0.953388641 | ⊕⊕○○ Low | -0.953388641 | ⊕⊕○○ Low |
| 119 | Dapagliflozin_low_dosage:Lixisenatide | 0 | | | -0.595283867 | ⊕⊕○○ Low | -0.595283867 | ⊕⊕○○ Low |
| 120 | Dapagliflozin_low_dosage:Oral_semaglutide | 0 | | | -0.986076201 | ⊕⊕○○ Low | -0.986076201 | ⊕⊕○○ Low |
| 121 | Dapagliflozin_low_dosage:Placebo_or_Control | 2 | -1.117310385 | ⊕⊕⊕⊕ High | | | -1.117310385 | ⊕⊕⊕○ Medium |
| 122 | Dapagliflozin_low_dosage:Sotagliflozin | 0 | | | -1.324030893 | ⊕⊕○○ Low | -1.324030893 | ⊕⊕○○ Low |
| 123 | Dapagliflozin_low_dosage:Tirzepatide | 0 | | | 0.836437966 | ⊕⊕○○ Low | 0.836437966 | ⊕⊕○○ Low |
| 124 | Dapagliflozin_medium_dosage:Dulaglutide | 0 | | | -1.92103332 | ⊕⊕○○ Low | -1.92103332 | ⊕⊕○○ Low |
| 125 | Dapagliflozin_medium_dosage:Efpeglenatide_high_dosage | 0 | | | -2.238571445 | ⊕⊕○○ Low | -2.238571445 | ⊕⊕○○ Low |
| 126 | Dapagliflozin_medium_dosage:Efpeglenatide_medium_dosage | 0 | | | -2.748660692 | ⊕⊕○○ Low | -2.748660692 | ⊕⊕○○ Low |
| 127 | Dapagliflozin_medium_dosage:Empagliflozin_high_dosage | 0 | | | -0.828359769 | ⊕⊕○○ Low | -0.828359769 | ⊕⊕○○ Low |
| 128 | Dapagliflozin_medium_dosage:Empagliflozin_low_dosage | 0 | | | -1.768901834 | ⊕⊕○○ Low | -1.768901834 | ⊕⊕○○ Low |
| 129 | Dapagliflozin_medium_dosage:Ertugliflozin_high_dosage | 0 | | | -1.69857059 | ⊕⊕○○ Low | -1.69857059 | ⊕⊕○○ Low |

| | | | | | | | | |
|---|---|---|---|---|---|---|---|---|
| 130 | Dapagliflozin_medium_dosage:Ertugliflozin_low_dosage | 0 | | | -1.314899975 | ⊕⊕○○ Low | -1.314899975 | ⊕⊕○○ Low |
| 131 | Dapagliflozin_medium_dosage:Exenatide | 0 | | | -1.218990965 | ⊕⊕○○ Low | -1.218990965 | ⊕⊕○○ Low |
| 132 | Dapagliflozin_medium_dosage:Inject_semaglutide_high_dosage | 0 | | | -1.180011881 | ⊕⊕○○ Low | -1.180011881 | ⊕⊕○○ Low |
| 133 | Dapagliflozin_medium_dosage:Inject_semaglutide_low_dosage | 0 | | | -0.187041166 | ⊕⊕○○ Low | -0.187041166 | ⊕⊕○○ Low |
| 134 | Dapagliflozin_medium_dosage:Inject_semaglutide_medium_dosage | 0 | | | -0.285530848 | ⊕⊕○○ Low | -0.285530848 | ⊕⊕○○ Low |
| 135 | Dapagliflozin_medium_dosage:Liraglutide | 0 | | | -0.977511795 | ⊕⊕○○ Low | -0.977511795 | ⊕⊕○○ Low |
| 136 | Dapagliflozin_medium_dosage:Lixisenatide | 0 | | | -0.619407021 | ⊕⊕○○ Low | -0.619407021 | ⊕⊕○○ Low |
| 137 | Dapagliflozin_medium_dosage:Oral_semaglutide | 0 | | | -1.010199356 | ⊕⊕○○ Low | -1.010199356 | ⊕⊕○○ Low |
| 138 | Dapagliflozin_medium_dosage:Placebo_or_Control | 2 | -1.141433539 | ⊕⊕⊕⊕ High | | | -1.141433539 | ⊕⊕⊕○ Medium |
| 139 | Dapagliflozin_medium_dosage:Sotagliflozin | 0 | | | -1.348154047 | ⊕⊕○○ Low | -1.348154047 | ⊕⊕○○ Low |
| 140 | Dapagliflozin_medium_dosage:Tirzepatide | 0 | | | 0.812314811 | ⊕⊕○○ Low | 0.812314811 | ⊕⊕○○ Low |
| 141 | Dulaglutide:Efpeglenatide_high_dosage | 0 | | | -0.317538125 | ⊕⊕○○ Low | -0.317538125 | ⊕⊕○○ Low |
| 142 | Dulaglutide:Efpeglenatide_medium_dosage | 0 | | | -0.827627372 | ⊕⊕○○ Low | -0.827627372 | ⊕⊕○○ Low |
| 143 | Dulaglutide:Empagliflozin_high_dosage | 0 | | | 1.092673551 | ⊕⊕○○ Low | 1.092673551 | ⊕⊕○○ Low |
| 144 | Dulaglutide:Empagliflozin_low_dosage | 0 | | | 0.152131486 | ⊕⊕○○ Low | 0.152131486 | ⊕⊕○○ Low |
| 145 | Dulaglutide:Ertugliflozin_high_dosage | 0 | | | 0.22246273 | ⊕⊕○○ Low | 0.22246273 | ⊕⊕○○ Low |
| 146 | Dulaglutide:Ertugliflozin_low_dosage | 0 | | | 0.606133345 | ⊕⊕○○ Low | 0.606133345 | ⊕⊕○○ Low |
| 147 | Dulaglutide:Exenatide | 0 | | | 0.702042355 | ⊕⊕○○ Low | 0.702042355 | ⊕⊕○○ Low |
| 148 | Dulaglutide:Inject_semaglutide_high_dosage | 0 | | | 0.741021439 | ⊕⊕○○ Low | 0.741021439 | ⊕⊕○○ Low |
| 149 | Dulaglutide:Inject_semaglutide_low_dosage | 0 | | | 1.733992154 | ⊕⊕○○ Low | 1.733992154 | ⊕⊕○○ Low |
| 150 | Dulaglutide:Inject_semaglutide_medium_dosage | 0 | | | 1.635502472 | ⊕⊕○○ Low | 1.635502472 | ⊕⊕○○ Low |
| 151 | Dulaglutide:Liraglutide | 0 | | | 0.943521525 | ⊕⊕○○ Low | 0.943521525 | ⊕⊕○○ Low |

| | | | | | | | | |
|---|---|---|---|---|---|---|---|---|
| 152 | Dulaglutide:Lixisenatide | 0 | | | 1.301626299 | ⊕⊕○○ Low | 1.301626299 | ⊕⊕○○ Low |
| 153 | Dulaglutide:Oral_semaglutide | 0 | | | 0.910833964 | ⊕⊕○○ Low | 0.910833964 | ⊕⊕○○ Low |
| 154 | Dulaglutide:Placebo_or_Control | 3 | 0.779599781 | ⊕⊕⊕⊕ High | | | 0.779599781 | ⊕⊕⊕○ Medium |
| 155 | Dulaglutide:Sotagliflozin | 0 | | | 0.572879273 | ⊕⊕○○ Low | 0.572879273 | ⊕⊕○○ Low |
| 156 | Dulaglutide:Tirzepatide | 0 | | | 2.733348131 | ⊕⊕○○ Low | 2.733348131 | ⊕⊕○○ Low |
| 157 | Efpeglenatide_high_dosage:Efpeglenatide_medium_dosage | 1 | -0.510089247 | ⊕⊕⊕⊕ High | | | -0.510089247 | ⊕⊕⊕○ Medium |
| 158 | Efpeglenatide_high_dosage:Empagliflozin_high_dosage | 0 | | | 1.410211676 | ⊕⊕○○ Low | 1.410211676 | ⊕⊕○○ Low |
| 159 | Efpeglenatide_high_dosage:Empagliflozin_low_dosage | 0 | | | 0.469669611 | ⊕⊕○○ Low | 0.469669611 | ⊕⊕○○ Low |
| 160 | Efpeglenatide_high_dosage:Ertugliflozin_high_dosage | 0 | | | 0.540000855 | ⊕⊕○○ Low | 0.540000855 | ⊕⊕○○ Low |
| 161 | Efpeglenatide_high_dosage:Ertugliflozin_low_dosage | 0 | | | 0.92367147 | ⊕⊕○○ Low | 0.92367147 | ⊕⊕○○ Low |
| 162 | Efpeglenatide_high_dosage:Exenatide | 0 | | | 1.01958048 | ⊕⊕○○ Low | 1.01958048 | ⊕⊕○○ Low |
| 163 | Efpeglenatide_high_dosage:Inject_semaglutide_high_dosage | 0 | | | 1.058559565 | ⊕⊕○○ Low | 1.058559565 | ⊕⊕○○ Low |
| 164 | Efpeglenatide_high_dosage:Inject_semaglutide_low_dosage | 0 | | | 2.05153028 | ⊕⊕○○ Low | 2.05153028 | ⊕⊕○○ Low |
| 165 | Efpeglenatide_high_dosage:Inject_semaglutide_medium_dosage | 0 | | | 1.953040597 | ⊕⊕○○ Low | 1.953040597 | ⊕⊕○○ Low |
| 166 | Efpeglenatide_high_dosage:Liraglutide | 0 | | | 1.26105965 | ⊕⊕○○ Low | 1.26105965 | ⊕⊕○○ Low |
| 167 | Efpeglenatide_high_dosage:Lixisenatide | 0 | | | 1.619164424 | ⊕⊕○○ Low | 1.619164424 | ⊕⊕○○ Low |
| 168 | Efpeglenatide_high_dosage:Oral_semaglutide | 0 | | | 1.22837209 | ⊕⊕○○ Low | 1.22837209 | ⊕⊕○○ Low |
| 169 | Efpeglenatide_high_dosage:Placebo_or_Control | 1 | 1.097137906 | ⊕⊕⊕⊕ High | | | 1.097137906 | ⊕⊕⊕○ Medium |
| 170 | Efpeglenatide_high_dosage:Sotagliflozin | 0 | | | 0.890417398 | ⊕⊕○○ Low | 0.890417398 | ⊕⊕○○ Low |
| 171 | Efpeglenatide_high_dosage:Tirzepatide | 0 | | | 3.050886256 | ⊕⊕○○ Low | 3.050886256 | ⊕⊕○○ Low |
| 172 | Efpeglenatide_medium_dosage:Empagliflozin_high_dosage | 0 | | | 1.920300922 | ⊕⊕○○ Low | 1.920300922 | ⊕⊕○○ Low |
| 173 | Efpeglenatide_medium_dosage:Empagliflozin_low_dosage | 0 | | | 0.979758858 | ⊕⊕○○ Low | 0.979758858 | ⊕⊕○○ Low |

| | | | | | | | | |
|---|---|---|---|---|---|---|---|---|
| 174 | Efpeglenatide_medium_dosage:Ertugliflozin_high_dosage | 0 | | | 1.050090102 | ⊕⊕○○ Low | 1.050090102 | ⊕⊕○○ Low |
| 175 | Efpeglenatide_medium_dosage:Ertugliflozin_low_dosage | 0 | | | 1.433760717 | ⊕⊕○○ Low | 1.433760717 | ⊕⊕○○ Low |
| 176 | Efpeglenatide_medium_dosage:Exenatide | 0 | | | 1.529669727 | ⊕⊕○○ Low | 1.529669727 | ⊕⊕○○ Low |
| 177 | Efpeglenatide_medium_dosage:Inject_semaglutide_high_dosage | 0 | | | 1.568648811 | ⊕⊕○○ Low | 1.568648811 | ⊕⊕○○ Low |
| 178 | Efpeglenatide_medium_dosage:Inject_semaglutide_low_dosage | 0 | | | 2.561619526 | ⊕⊕○○ Low | 2.561619526 | ⊕⊕○○ Low |
| 179 | Efpeglenatide_medium_dosage:Inject_semaglutide_medium_dosage | 0 | | | 2.463129844 | ⊕⊕○○ Low | 2.463129844 | ⊕⊕○○ Low |
| 180 | Efpeglenatide_medium_dosage:Liraglutide | 0 | | | 1.771148897 | ⊕⊕○○ Low | 1.771148897 | ⊕⊕○○ Low |
| 181 | Efpeglenatide_medium_dosage:Lixisenatide | 0 | | | 2.12925367 | ⊕⊕○○ Low | 2.12925367 | ⊕⊕○○ Low |
| 182 | Efpeglenatide_medium_dosage:Oral_semaglutide | 0 | | | 1.738461336 | ⊕⊕○○ Low | 1.738461336 | ⊕⊕○○ Low |
| 183 | Efpeglenatide_medium_dosage:Placebo_or_Control | 1 | 1.607227153 | ⊕⊕⊕⊕ High | | | 1.607227153 | ⊕⊕⊕○ Medium |
| 184 | Efpeglenatide_medium_dosage:Sotagliflozin | 0 | | | 1.400506645 | ⊕⊕○○ Low | 1.400506645 | ⊕⊕○○ Low |
| 185 | Efpeglenatide_medium_dosage:Tirzepatide | 0 | | | 3.560975503 | ⊕⊕○○ Low | 3.560975503 | ⊕⊕○○ Low |
| 186 | Empagliflozin_high_dosage:Empagliflozin_low_dosage | 3 | -0.981454702 | ⊕⊕⊕⊕ High | -0.798921716 | ⊕⊕⊕⊕ High | -0.940542064 | ⊕⊕⊕⊕ High |
| 187 | Empagliflozin_high_dosage:Ertugliflozin_high_dosage | 0 | | | -0.87021082 | ⊕⊕○○ Low | -0.87021082 | ⊕⊕○○ Low |
| 188 | Empagliflozin_high_dosage:Ertugliflozin_low_dosage | 0 | | | -0.486540205 | ⊕⊕○○ Low | -0.486540205 | ⊕⊕○○ Low |
| 189 | Empagliflozin_high_dosage:Exenatide | 0 | | | -0.390631196 | ⊕⊕○○ Low | -0.390631196 | ⊕⊕○○ Low |
| 190 | Empagliflozin_high_dosage:Inject_semaglutide_high_dosage | 0 | | | -0.351652111 | ⊕⊕○○ Low | -0.351652111 | ⊕⊕○○ Low |
| 191 | Empagliflozin_high_dosage:Inject_semaglutide_low_dosage | 0 | | | 0.641318604 | ⊕⊕○○ Low | 0.641318604 | ⊕⊕○○ Low |
| 192 | Empagliflozin_high_dosage:Inject_semaglutide_medium_dosage | 0 | | | 0.542828921 | ⊕⊕○○ Low | 0.542828921 | ⊕⊕○○ Low |
| 193 | Empagliflozin_high_dosage:Liraglutide | 0 | | | -0.149152026 | ⊕⊕○○ Low | -0.149152026 | ⊕⊕○○ Low |
| 194 | Empagliflozin_high_dosage:Lixisenatide | 0 | | | 0.208952748 | ⊕⊕○○ Low | 0.208952748 | ⊕⊕○○ Low |
| 195 | Empagliflozin_high_dosage:Oral_semaglutide | 0 | | | -0.181839586 | ⊕⊕○○ Low | -0.181839586 | ⊕⊕○○ Low |

| | | | | | | | | |
|---|---|---|---|---|---|---|---|---|
| 196 | Empagliflozin_high_dosage:Placebo_or_Control | 3 | -0.532377685 | ⊕⊕⊕⊕ High | 0.253625151 | ⊕⊕⊕○ Medium | -0.31307377 | ⊕⊕⊕⊕ High |
| 197 | Empagliflozin_high_dosage:Sotagliflozin | 0 | | | -0.519794278 | ⊕⊕○○ Low | -0.519794278 | ⊕⊕○○ Low |
| 198 | Empagliflozin_high_dosage:Tirzepatide | 0 | | | 1.64067458 | ⊕⊕○○ Low | 1.64067458 | ⊕⊕○○ Low |
| 199 | Empagliflozin_low_dosage:Ertugliflozin_high_dosage | 0 | | | 0.070331244 | ⊕⊕○○ Low | 0.070331244 | ⊕⊕○○ Low |
| 200 | Empagliflozin_low_dosage:Ertugliflozin_low_dosage | 0 | | | 0.454001859 | ⊕⊕○○ Low | 0.454001859 | ⊕⊕○○ Low |
| 201 | Empagliflozin_low_dosage:Exenatide | 0 | | | 0.549910869 | ⊕⊕○○ Low | 0.549910869 | ⊕⊕○○ Low |
| 202 | Empagliflozin_low_dosage:Inject_semaglutide_high_dosage | 0 | | | 0.588889953 | ⊕⊕○○ Low | 0.588889953 | ⊕⊕○○ Low |
| 203 | Empagliflozin_low_dosage:Inject_semaglutide_low_dosage | 0 | | | 1.581860668 | ⊕⊕○○ Low | 1.581860668 | ⊕⊕○○ Low |
| 204 | Empagliflozin_low_dosage:Inject_semaglutide_medium_dosage | 0 | | | 1.483370986 | ⊕⊕○○ Low | 1.483370986 | ⊕⊕○○ Low |
| 205 | Empagliflozin_low_dosage:Liraglutide | 0 | | | 0.791390039 | ⊕⊕○○ Low | 0.791390039 | ⊕⊕○○ Low |
| 206 | Empagliflozin_low_dosage:Lixisenatide | 0 | | | 1.149494812 | ⊕⊕○○ Low | 1.149494812 | ⊕⊕○○ Low |
| 207 | Empagliflozin_low_dosage:Oral_semaglutide | 0 | | | 0.758702478 | ⊕⊕○○ Low | 0.758702478 | ⊕⊕○○ Low |
| 208 | Empagliflozin_low_dosage:Placebo_or_Control | 5 | 0.650204734 | ⊕⊕⊕⊕ High | 0.093247386 | ⊕⊕⊕⊕ High | 0.627468295 | ⊕⊕⊕⊕ High |
| 209 | Empagliflozin_low_dosage:Sotagliflozin | 0 | | | 0.420747787 | ⊕⊕○○ Low | 0.420747787 | ⊕⊕○○ Low |
| 210 | Empagliflozin_low_dosage:Tirzepatide | 0 | | | 2.581216645 | ⊕⊕○○ Low | 2.581216645 | ⊕⊕○○ Low |
| 211 | Ertugliflozin_high_dosage:Ertugliflozin_low_dosage | 3 | 0.335598871 | ⊕⊕⊕⊕ High | 1.831310087 | ⊕⊕⊕⊕ High | 0.383670615 | ⊕⊕⊕⊕ High |
| 212 | Ertugliflozin_high_dosage:Exenatide | 0 | | | 0.479579625 | ⊕⊕○○ Low | 0.479579625 | ⊕⊕○○ Low |
| 213 | Ertugliflozin_high_dosage:Inject_semaglutide_high_dosage | 0 | | | 0.518558709 | ⊕⊕○○ Low | 0.518558709 | ⊕⊕○○ Low |
| 214 | Ertugliflozin_high_dosage:Inject_semaglutide_low_dosage | 0 | | | 1.511529424 | ⊕⊕○○ Low | 1.511529424 | ⊕⊕○○ Low |
| 215 | Ertugliflozin_high_dosage:Inject_semaglutide_medium_dosage | 0 | | | 1.413039742 | ⊕⊕○○ Low | 1.413039742 | ⊕⊕○○ Low |
| 216 | Ertugliflozin_high_dosage:Liraglutide | 0 | | | 0.721058795 | ⊕⊕○○ Low | 0.721058795 | ⊕⊕○○ Low |
| 217 | Ertugliflozin_high_dosage:Lixisenatide | 0 | | | 1.079163568 | ⊕⊕○○ Low | 1.079163568 | ⊕⊕○○ Low |

| | | | | | | | | |
|---|---|---|---|---|---|---|---|---|
| 218 | Ertugliflozin_high_dosage:Oral_semaglutide | 0 | | | 0.688371234 | ⊕⊕○○ Low | 0.688371234 | ⊕⊕○○ Low |
| 219 | Ertugliflozin_high_dosage:Placebo_or_Control | 3 | 0.494571754 | ⊕⊕⊕⊕ High | 2.650338031 | ⊕⊕⊕⊕ High | 0.557137051 | ⊕⊕⊕⊕ High |
| 220 | Ertugliflozin_high_dosage:Sotagliflozin | 0 | | | 0.350416543 | ⊕⊕○○ Low | 0.350416543 | ⊕⊕○○ Low |
| 221 | Ertugliflozin_high_dosage:Tirzepatide | 0 | | | 2.510885401 | ⊕⊕○○ Low | 2.510885401 | ⊕⊕○○ Low |
| 222 | Ertugliflozin_low_dosage:Exenatide | 0 | | | 0.09590901 | ⊕⊕○○ Low | 0.09590901 | ⊕⊕○○ Low |
| 223 | Ertugliflozin_low_dosage:Inject_semaglutide_high_dosage | 0 | | | 0.134888094 | ⊕⊕○○ Low | 0.134888094 | ⊕⊕○○ Low |
| 224 | Ertugliflozin_low_dosage:Inject_semaglutide_low_dosage | 0 | | | 1.127858809 | ⊕⊕○○ Low | 1.127858809 | ⊕⊕○○ Low |
| 225 | Ertugliflozin_low_dosage:Inject_semaglutide_medium_dosage | 0 | | | 1.029369127 | ⊕⊕○○ Low | 1.029369127 | ⊕⊕○○ Low |
| 226 | Ertugliflozin_low_dosage:Liraglutide | 0 | | | 0.337388179 | ⊕⊕○○ Low | 0.337388179 | ⊕⊕○○ Low |
| 227 | Ertugliflozin_low_dosage:Lixisenatide | 0 | | | 0.695492953 | ⊕⊕○○ Low | 0.695492953 | ⊕⊕○○ Low |
| 228 | Ertugliflozin_low_dosage:Oral_semaglutide | 0 | | | 0.304700619 | ⊕⊕○○ Low | 0.304700619 | ⊕⊕○○ Low |
| 229 | Ertugliflozin_low_dosage:Placebo_or_Control | 1 | 0.182321557 | ⊕⊕⊕⊕ High | 0.109312861 | ⊕⊕⊕⊕ High | 0.173466435 | ⊕⊕⊕⊕ High |
| 230 | Ertugliflozin_low_dosage:Sotagliflozin | 0 | | | -0.033254073 | ⊕⊕○○ Low | -0.033254073 | ⊕⊕○○ Low |
| 231 | Ertugliflozin_low_dosage:Tirzepatide | 0 | | | 2.127214786 | ⊕⊕○○ Low | 2.127214786 | ⊕⊕○○ Low |
| 232 | Exenatide:Inject_semaglutide_high_dosage | 0 | | | 0.038979084 | ⊕⊕○○ Low | 0.038979084 | ⊕⊕○○ Low |
| 233 | Exenatide:Inject_semaglutide_low_dosage | 0 | | | 1.031949799 | ⊕⊕○○ Low | 1.031949799 | ⊕⊕○○ Low |
| 234 | Exenatide:Inject_semaglutide_medium_dosage | 0 | | | 0.933460117 | ⊕⊕○○ Low | 0.933460117 | ⊕⊕○○ Low |
| 235 | Exenatide:Liraglutide | 1 | 1.115743329 | ⊕⊕⊕⊕ High | 0.182215554 | ⊕⊕⊕⊕ High | 0.24147917 | ⊕⊕⊕⊕ High |
| 236 | Exenatide:Lixisenatide | 0 | | | 0.599583943 | ⊕⊕○○ Low | 0.599583943 | ⊕⊕○○ Low |
| 237 | Exenatide:Oral_semaglutide | 0 | | | 0.208791609 | ⊕⊕○○ Low | 0.208791609 | ⊕⊕○○ Low |
| 238 | Exenatide:Placebo_or_Control | 1 | 0.048405145 | ⊕⊕⊕⊕ High | 0.981932919 | ⊕⊕⊕⊕ High | 0.077557426 | ⊕⊕⊕⊕ High |
| 239 | Exenatide:Sotagliflozin | 0 | | | -0.129163082 | ⊕⊕○○ Low | -0.129163082 | ⊕⊕○○ Low |

| | | | | | | | | |
|---|---|---|---|---|---|---|---|---|
| 240 | Exenatide:Tirzepatide | 0 | | | 2.031305776 | ⊕⊕○○ Low | 2.031305776 | ⊕⊕○○ Low |
| 241 | Inject_semaglutide_high_dosage:Inject_semaglutide_low_dosage | 0 | | | 0.992970715 | ⊕⊕○○ Low | 0.992970715 | ⊕⊕○○ Low |
| 242 | Inject_semaglutide_high_dosage:Inject_semaglutide_medium_dosage | 1 | 1.611925476 | ⊕⊕⊕⊕ High | 0.737494445 | ⊕⊕⊕⊕ High | 0.894481032 | ⊕⊕⊕⊕ High |
| 243 | Inject_semaglutide_high_dosage:Liraglutide | 0 | | | 0.202500085 | ⊕⊕○○ Low | 0.202500085 | ⊕⊕○○ Low |
| 244 | Inject_semaglutide_high_dosage:Lixisenatide | 0 | | | 0.560604859 | ⊕⊕○○ Low | 0.560604859 | ⊕⊕○○ Low |
| 245 | Inject_semaglutide_high_dosage:Oral_semaglutide | 0 | | | 0.169812525 | ⊕⊕○○ Low | 0.169812525 | ⊕⊕○○ Low |
| 246 | Inject_semaglutide_high_dosage:Placebo_or_Control | 4 | 0.012948973 | ⊕⊕⊕⊕ High | 0.887380004 | ⊕⊕⊕⊕ High | 0.038578341 | ⊕⊕⊕⊕ High |
| 247 | Inject_semaglutide_high_dosage:Sotagliflozin | 0 | | | -0.168142167 | ⊕⊕○○ Low | -0.168142167 | ⊕⊕○○ Low |
| 248 | Inject_semaglutide_high_dosage:Tirzepatide | 0 | | | 1.992326692 | ⊕⊕○○ Low | 1.992326692 | ⊕⊕○○ Low |
| 249 | Inject_semaglutide_low_dosage:Inject_semaglutide_medium_dosage | 2 | -0.048130021 | ⊕⊕⊕⊕ High | -0.335218098 | ⊕⊕⊕⊕ High | -0.098489683 | ⊕⊕⊕⊕ High |
| 250 | Inject_semaglutide_low_dosage:Liraglutide | 0 | | | -0.79047063 | ⊕⊕○○ Low | -0.79047063 | ⊕⊕○○ Low |
| 251 | Inject_semaglutide_low_dosage:Lixisenatide | 0 | | | -0.432365856 | ⊕⊕○○ Low | -0.432365856 | ⊕⊕○○ Low |
| 252 | Inject_semaglutide_low_dosage:Oral_semaglutide | 0 | | | -0.82315819 | ⊕⊕○○ Low | -0.82315819 | ⊕⊕○○ Low |
| 253 | Inject_semaglutide_low_dosage:Placebo_or_Control | 2 | -1.072448502 | ⊕⊕⊕⊕ High | 1.759823751 | ⊕⊕⊕○ Medium | -0.954392374 | ⊕⊕⊕⊕ High |
| 254 | Inject_semaglutide_low_dosage:Sotagliflozin | 0 | | | -1.161112882 | ⊕⊕○○ Low | -1.161112882 | ⊕⊕○○ Low |
| 255 | Inject_semaglutide_low_dosage:Tirzepatide | 0 | | | 0.999355977 | ⊕⊕○○ Low | 0.999355977 | ⊕⊕○○ Low |
| 256 | Inject_semaglutide_medium_dosage:Liraglutide | 0 | | | -0.691980947 | ⊕⊕○○ Low | -0.691980947 | ⊕⊕○○ Low |
| 257 | Inject_semaglutide_medium_dosage:Lixisenatide | 0 | | | -0.333876173 | ⊕⊕○○ Low | -0.333876173 | ⊕⊕○○ Low |
| 258 | Inject_semaglutide_medium_dosage:Oral_semaglutide | 0 | | | -0.724668507 | ⊕⊕○○ Low | -0.724668507 | ⊕⊕○○ Low |
| 259 | Inject_semaglutide_medium_dosage:Placebo_or_Control | 2 | -0.902926539 | ⊕⊕⊕⊕ High | -0.724395344 | ⊕⊕⊕⊕ High | -0.855902691 | ⊕⊕⊕⊕ High |
| 260 | Inject_semaglutide_medium_dosage:Sotagliflozin | 0 | | | -1.062623199 | ⊕⊕○○ Low | -1.062623199 | ⊕⊕○○ Low |
| 261 | Inject_semaglutide_medium_dosage:Tirzepatide | 1 | 2.200066498 | ⊕⊕⊕⊕ High | 0.655506111 | ⊕⊕⊕⊕ High | 1.097845659 | ⊕⊕⊕⊕ High |

| | | | | | | | | |
|---|---|---|---|---|---|---|---|---|
| 262 | Liraglutide:Lixisenatide | 1 | 1.103562794 | ⊕⊕⊕⊕ High | 0.216302341 | ⊕⊕⊕⊕ High | 0.358104774 | ⊕⊕⊕⊕ High |
| 263 | Liraglutide:Oral_semaglutide | 0 | | | -0.03268756 | ⊕⊕○○ Low | -0.03268756 | ⊕⊕○○ Low |
| 264 | Liraglutide:Placebo_or_Control | 3 | -0.159336038 | ⊕⊕⊕⊕ High | -0.234417885 | ⊕⊕⊕⊕ High | -0.163921744 | ⊕⊕⊕⊕ High |
| 265 | Liraglutide:Sotagliflozin | 0 | | | -0.370642252 | ⊕⊕○○ Low | -0.370642252 | ⊕⊕○○ Low |
| 266 | Liraglutide:Tirzepatide | 0 | | | 1.789826606 | ⊕⊕○○ Low | 1.789826606 | ⊕⊕○○ Low |
| 267 | Lixisenatide:Oral_semaglutide | 0 | | | -0.390792334 | ⊕⊕○○ Low | -0.390792334 | ⊕⊕○○ Low |
| 268 | Lixisenatide:Placebo_or_Control | 1 | -0.405795523 | ⊕⊕⊕⊕ High | -1.293055976 | ⊕⊕⊕⊕ High | -0.522026518 | ⊕⊕⊕⊕ High |
| 269 | Lixisenatide:Sotagliflozin | 0 | | | -0.728747026 | ⊕⊕○○ Low | -0.728747026 | ⊕⊕○○ Low |
| 270 | Lixisenatide:Tirzepatide | 0 | | | 1.431721833 | ⊕⊕○○ Low | 1.431721833 | ⊕⊕○○ Low |
| 271 | Oral_semaglutide:Placebo_or_Control | 2 | -0.131234184 | ⊕⊕⊕⊕ High | | | -0.131234184 | ⊕⊕⊕○ Medium |
| 272 | Oral_semaglutide:Sotagliflozin | 0 | | | -0.337954692 | ⊕⊕○○ Low | -0.337954692 | ⊕⊕○○ Low |
| 273 | Oral_semaglutide:Tirzepatide | 0 | | | 1.822514167 | ⊕⊕○○ Low | 1.822514167 | ⊕⊕○○ Low |
| 274 | Sotagliflozin:Placebo_or_Control | 4 | 0.206720508 | ⊕⊕⊕⊕ High | | | 0.206720508 | ⊕⊕⊕○ Medium |
| 275 | Tirzepatide:Placebo_or_Control | 4 | -1.685536016 | ⊕⊕⊕⊕ High | -3.230096402 | ⊕⊕⊕⊕ High | -1.95374835 | ⊕⊕⊕⊕ High |
| 276 | Sotagliflozin:Tirzepatide | 0 | | | 2.160468858 | ⊕⊕○○ Low | 2.160468858 | ⊕⊕○○ Low |

## Table S8B: GRADE of primary outcome: subgroup of lymphoma

| | Comparison | No.Studies | Direct | | Indirect | | NMA | |
|---|---|---|---|---|---|---|---|---|
| | | | Estimate | Rate | Estimate | Rate | Estimate | Rate |
| 1 | Albiglutide:Bexagliflozin | 0 | | | 0.187481401 | ⊕⊕○○ Low | 0.187481401 | ⊕⊕○○ Low |
| 2 | Albiglutide:Canagliflozin_high_dosage | 0 | | | 1.210733311 | ⊕⊕○○ Low | 1.210733311 | ⊕⊕○○ Low |
| 3 | Albiglutide:Canagliflozin_low_dosage | 0 | | | 0.734828593 | ⊕⊕○○ Low | 0.734828593 | ⊕⊕○○ Low |
| 4 | Albiglutide:Dapagliflozin_high_dosage | 0 | | | -0.01856673 | ⊕⊕○○ Low | -0.01856673 | ⊕⊕○○ Low |
| 5 | Albiglutide:Dulaglutide | 0 | | | -0.203152141 | ⊕⊕○○ Low | -0.203152141 | ⊕⊕○○ Low |
| 6 | Albiglutide:Efpeglenatide_high_dosage | 0 | | | -0.501102932 | ⊕⊕○○ Low | -0.501102932 | ⊕⊕○○ Low |
| 7 | Albiglutide:Efpeglenatide_medium_dosage | 0 | | | -0.49963072 | ⊕⊕○○ Low | -0.49963072 | ⊕⊕○○ Low |
| 8 | Albiglutide:Empagliflozin_high_dosage | 0 | | | 1.387604525 | ⊕⊕○○ Low | 1.387604525 | ⊕⊕○○ Low |
| 9 | Albiglutide:Empagliflozin_low_dosage | 0 | | | 0.227884993 | ⊕⊕○○ Low | 0.227884993 | ⊕⊕○○ Low |
| 10 | Albiglutide:Ertugliflozin_high_dosage | 0 | | | 0.094447335 | ⊕⊕○○ Low | 0.094447335 | ⊕⊕○○ Low |
| 11 | Albiglutide:Ertugliflozin_low_dosage | 0 | | | 0.318143618 | ⊕⊕○○ Low | 0.318143618 | ⊕⊕○○ Low |
| 12 | Albiglutide:Exenatide | 0 | | | 0.843811983 | ⊕⊕○○ Low | 0.843811983 | ⊕⊕○○ Low |
| 13 | Albiglutide:Inject_semaglutide_high_dosage | 0 | | | 0.289712541 | ⊕⊕○○ Low | 0.289712541 | ⊕⊕○○ Low |
| 14 | Albiglutide:Inject_semaglutide_low_dosage | 0 | | | 1.150511412 | ⊕⊕○○ Low | 1.150511412 | ⊕⊕○○ Low |
| 15 | Albiglutide:Inject_semaglutide_medium_dosage | 0 | | | 1.297736098 | ⊕⊕○○ Low | 1.297736098 | ⊕⊕○○ Low |
| 16 | Albiglutide:Liraglutide | 0 | | | 0.359754786 | ⊕⊕○○ Low | 0.359754786 | ⊕⊕○○ Low |
| 17 | Albiglutide:Lixisenatide | 0 | | | 1.512986207 | ⊕⊕○○ Low | 1.512986207 | ⊕⊕○○ Low |
| 18 | Albiglutide:Oral_semaglutide | 0 | | | 0.904726442 | ⊕⊕○○ Low | 0.904726442 | ⊕⊕○○ Low |
| 19 | Albiglutide:Placebo_or_Control | 2 | 0.596034973 | ⊕⊕⊕⊕ High | | | 0.596034973 | ⊕⊕⊕○ Medium |

| | | | | | | | | |
|---|---|---|---|---|---|---|---|---|
| 20 | Albiglutide:Sotagliflozin | 0 | | | 0.435480895 | ⊕⊕○○ Low | 0.435480895 | ⊕⊕○○ Low |
| 21 | Albiglutide:Tirzepatide | 0 | | | 2.316391428 | ⊕⊕○○ Low | 2.316391428 | ⊕⊕○○ Low |
| 22 | Bexagliflozin:Canagliflozin_high_dosage | 0 | | | 1.02325191 | ⊕⊕○○ Low | 1.02325191 | ⊕⊕○○ Low |
| 23 | Bexagliflozin:Canagliflozin_low_dosage | 0 | | | 0.547347192 | ⊕⊕○○ Low | 0.547347192 | ⊕⊕○○ Low |
| 24 | Bexagliflozin:Dapagliflozin_high_dosage | 0 | | | -0.20604813 | ⊕⊕○○ Low | -0.20604813 | ⊕⊕○○ Low |
| 25 | Bexagliflozin:Dulaglutide | 0 | | | -0.390633542 | ⊕⊕○○ Low | -0.390633542 | ⊕⊕○○ Low |
| 26 | Bexagliflozin:Efpeglenatide_high_dosage | 0 | | | -0.688584333 | ⊕⊕○○ Low | -0.688584333 | ⊕⊕○○ Low |
| 27 | Bexagliflozin:Efpeglenatide_medium_dosage | 0 | | | -0.687112121 | ⊕⊕○○ Low | -0.687112121 | ⊕⊕○○ Low |
| 28 | Bexagliflozin:Empagliflozin_high_dosage | 0 | | | 1.200123124 | ⊕⊕○○ Low | 1.200123124 | ⊕⊕○○ Low |
| 29 | Bexagliflozin:Empagliflozin_low_dosage | 0 | | | 0.040403592 | ⊕⊕○○ Low | 0.040403592 | ⊕⊕○○ Low |
| 30 | Bexagliflozin:Ertugliflozin_high_dosage | 0 | | | -0.093034066 | ⊕⊕○○ Low | -0.093034066 | ⊕⊕○○ Low |
| 31 | Bexagliflozin:Ertugliflozin_low_dosage | 0 | | | 0.130662218 | ⊕⊕○○ Low | 0.130662218 | ⊕⊕○○ Low |
| 32 | Bexagliflozin:Exenatide | 0 | | | 0.656330583 | ⊕⊕○○ Low | 0.656330583 | ⊕⊕○○ Low |
| 33 | Bexagliflozin:Inject_semaglutide_high_dosage | 0 | | | 0.10223114 | ⊕⊕○○ Low | 0.10223114 | ⊕⊕○○ Low |
| 34 | Bexagliflozin:Inject_semaglutide_low_dosage | 0 | | | 0.963030011 | ⊕⊕○○ Low | 0.963030011 | ⊕⊕○○ Low |
| 35 | Bexagliflozin:Inject_semaglutide_medium_dosage | 0 | | | 1.110254697 | ⊕⊕○○ Low | 1.110254697 | ⊕⊕○○ Low |
| 36 | Bexagliflozin:Liraglutide | 0 | | | 0.172273385 | ⊕⊕○○ Low | 0.172273385 | ⊕⊕○○ Low |
| 37 | Bexagliflozin:Lixisenatide | 0 | | | 1.325504807 | ⊕⊕○○ Low | 1.325504807 | ⊕⊕○○ Low |
| 38 | Bexagliflozin:Oral_semaglutide | 0 | | | 0.717245041 | ⊕⊕○○ Low | 0.717245041 | ⊕⊕○○ Low |
| 39 | Bexagliflozin:Placebo_or_Control | 1 | 0.408553573 | ⊕⊕⊕⊕ High | | | 0.408553573 | ⊕⊕⊕○ Medium |
| 40 | Bexagliflozin:Sotagliflozin | 0 | | | 0.247999494 | ⊕⊕○○ Low | 0.247999494 | ⊕⊕○○ Low |
| 41 | Bexagliflozin:Tirzepatide | 0 | | | 2.128910027 | ⊕⊕○○ Low | 2.128910027 | ⊕⊕○○ Low |

| | | | | | | | | |
|---|---|---|---|---|---|---|---|---|
| 42 | Canagliflozin_high_dosage:Canagliflozin_low_dosage | 1 | -0.334390348 | ⊕⊕⊕⊕ High | -1.033707986 | ⊕⊕⊕⊕ High | -0.475904718 | ⊕⊕⊕⊕ High |
| 43 | Canagliflozin_high_dosage:Dapagliflozin_high_dosage | 0 | | | -1.22930004 | ⊕⊕○○ Low | -1.22930004 | ⊕⊕○○ Low |
| 44 | Canagliflozin_high_dosage:Dulaglutide | 0 | | | -1.413885452 | ⊕⊕○○ Low | -1.413885452 | ⊕⊕○○ Low |
| 45 | Canagliflozin_high_dosage:Efpeglenatide_high_dosage | 0 | | | -1.711836243 | ⊕⊕○○ Low | -1.711836243 | ⊕⊕○○ Low |
| 46 | Canagliflozin_high_dosage:Efpeglenatide_medium_dosage | 0 | | | -1.710364031 | ⊕⊕○○ Low | -1.710364031 | ⊕⊕○○ Low |
| 47 | Canagliflozin_high_dosage:Empagliflozin_high_dosage | 0 | | | 0.176871214 | ⊕⊕○○ Low | 0.176871214 | ⊕⊕○○ Low |
| 48 | Canagliflozin_high_dosage:Empagliflozin_low_dosage | 0 | | | -0.982848318 | ⊕⊕○○ Low | -0.982848318 | ⊕⊕○○ Low |
| 49 | Canagliflozin_high_dosage:Ertugliflozin_high_dosage | 0 | | | -1.116285976 | ⊕⊕○○ Low | -1.116285976 | ⊕⊕○○ Low |
| 50 | Canagliflozin_high_dosage:Ertugliflozin_low_dosage | 0 | | | -0.892589692 | ⊕⊕○○ Low | -0.892589692 | ⊕⊕○○ Low |
| 51 | Canagliflozin_high_dosage:Exenatide | 0 | | | -0.366921328 | ⊕⊕○○ Low | -0.366921328 | ⊕⊕○○ Low |
| 52 | Canagliflozin_high_dosage:Inject_semaglutide_high_dosage | 0 | | | -0.92102077 | ⊕⊕○○ Low | -0.92102077 | ⊕⊕○○ Low |
| 53 | Canagliflozin_high_dosage:Inject_semaglutide_low_dosage | 0 | | | -0.060221899 | ⊕⊕○○ Low | -0.060221899 | ⊕⊕○○ Low |
| 54 | Canagliflozin_high_dosage:Inject_semaglutide_medium_dosage | 0 | | | 0.087002787 | ⊕⊕○○ Low | 0.087002787 | ⊕⊕○○ Low |
| 55 | Canagliflozin_high_dosage:Liraglutide | 0 | | | -0.850978525 | ⊕⊕○○ Low | -0.850978525 | ⊕⊕○○ Low |
| 56 | Canagliflozin_high_dosage:Lixisenatide | 0 | | | 0.302252897 | ⊕⊕○○ Low | 0.302252897 | ⊕⊕○○ Low |
| 57 | Canagliflozin_high_dosage:Oral_semaglutide | 0 | | | -0.306006869 | ⊕⊕○○ Low | -0.306006869 | ⊕⊕○○ Low |
| 58 | Canagliflozin_high_dosage:Placebo_or_Control | 2 | -0.360808 | ⊕⊕⊕⊕ High | -1.558128736 | ⊕⊕⊕⊕ High | -0.614698337 | ⊕⊕⊕⊕ High |
| 59 | Canagliflozin_high_dosage:Sotagliflozin | 0 | | | -0.775252416 | ⊕⊕○○ Low | -0.775252416 | ⊕⊕○○ Low |
| 60 | Canagliflozin_high_dosage:Tirzepatide | 0 | | | 1.105658117 | ⊕⊕○○ Low | 1.105658117 | ⊕⊕○○ Low |
| 61 | Canagliflozin_low_dosage:Dapagliflozin_high_dosage | 0 | | | -0.753395322 | ⊕⊕○○ Low | -0.753395322 | ⊕⊕○○ Low |
| 62 | Canagliflozin_low_dosage:Dulaglutide | 0 | | | -0.937980734 | ⊕⊕○○ Low | -0.937980734 | ⊕⊕○○ Low |
| 63 | Canagliflozin_low_dosage:Efpeglenatide_high_dosage | 0 | | | -1.235931525 | ⊕⊕○○ Low | -1.235931525 | ⊕⊕○○ Low |

| | | | | | | | | |
|---|---|---|---|---|---|---|---|---|
| 64 | Canagliflozin_low_dosage:Efpeglenatide_medium_dosage | 0 | | | -1.234459313 | ⊕⊕○○ Low | -1.234459313 | ⊕⊕○○ Low |
| 65 | Canagliflozin_low_dosage:Empagliflozin_high_dosage | 0 | | | 0.652775932 | ⊕⊕○○ Low | 0.652775932 | ⊕⊕○○ Low |
| 66 | Canagliflozin_low_dosage:Empagliflozin_low_dosage | 0 | | | -0.5069436 | ⊕⊕○○ Low | -0.5069436 | ⊕⊕○○ Low |
| 67 | Canagliflozin_low_dosage:Ertugliflozin_high_dosage | 0 | | | -0.640381258 | ⊕⊕○○ Low | -0.640381258 | ⊕⊕○○ Low |
| 68 | Canagliflozin_low_dosage:Ertugliflozin_low_dosage | 0 | | | -0.416684974 | ⊕⊕○○ Low | -0.416684974 | ⊕⊕○○ Low |
| 69 | Canagliflozin_low_dosage:Exenatide | 0 | | | 0.10898339 | ⊕⊕○○ Low | 0.10898339 | ⊕⊕○○ Low |
| 70 | Canagliflozin_low_dosage:Inject_semaglutide_high_dosage | 0 | | | -0.445116052 | ⊕⊕○○ Low | -0.445116052 | ⊕⊕○○ Low |
| 71 | Canagliflozin_low_dosage:Inject_semaglutide_low_dosage | 0 | | | 0.415682819 | ⊕⊕○○ Low | 0.415682819 | ⊕⊕○○ Low |
| 72 | Canagliflozin_low_dosage:Inject_semaglutide_medium_dosage | 0 | | | 0.562907505 | ⊕⊕○○ Low | 0.562907505 | ⊕⊕○○ Low |
| 73 | Canagliflozin_low_dosage:Liraglutide | 0 | | | -0.375073807 | ⊕⊕○○ Low | -0.375073807 | ⊕⊕○○ Low |
| 74 | Canagliflozin_low_dosage:Lixisenatide | 0 | | | 0.778157615 | ⊕⊕○○ Low | 0.778157615 | ⊕⊕○○ Low |
| 75 | Canagliflozin_low_dosage:Oral_semaglutide | 0 | | | 0.169897849 | ⊕⊕○○ Low | 0.169897849 | ⊕⊕○○ Low |
| 76 | Canagliflozin_low_dosage:Placebo_or_Control | 2 | 0.351143058 | ⊕⊕⊕⊕ High | -1.482530374 | ⊕⊕⊕○ Medium | -0.138793619 | ⊕⊕⊕⊕ High |
| 77 | Canagliflozin_low_dosage:Sotagliflozin | 0 | | | -0.299347698 | ⊕⊕○○ Low | -0.299347698 | ⊕⊕○○ Low |
| 78 | Canagliflozin_low_dosage:Tirzepatide | 0 | | | 1.581562835 | ⊕⊕○○ Low | 1.581562835 | ⊕⊕○○ Low |
| 79 | Dapagliflozin_high_dosage:Dulaglutide | 0 | | | -0.184585412 | ⊕⊕○○ Low | -0.184585412 | ⊕⊕○○ Low |
| 80 | Dapagliflozin_high_dosage:Efpeglenatide_high_dosage | 0 | | | -0.482536203 | ⊕⊕○○ Low | -0.482536203 | ⊕⊕○○ Low |
| 81 | Dapagliflozin_high_dosage:Efpeglenatide_medium_dosage | 0 | | | -0.48106399 | ⊕⊕○○ Low | -0.48106399 | ⊕⊕○○ Low |
| 82 | Dapagliflozin_high_dosage:Empagliflozin_high_dosage | 0 | | | 1.406171254 | ⊕⊕○○ Low | 1.406171254 | ⊕⊕○○ Low |
| 83 | Dapagliflozin_high_dosage:Empagliflozin_low_dosage | 0 | | | 0.246451722 | ⊕⊕○○ Low | 0.246451722 | ⊕⊕○○ Low |
| 84 | Dapagliflozin_high_dosage:Ertugliflozin_high_dosage | 0 | | | 0.113014064 | ⊕⊕○○ Low | 0.113014064 | ⊕⊕○○ Low |
| 85 | Dapagliflozin_high_dosage:Ertugliflozin_low_dosage | 0 | | | 0.336710348 | ⊕⊕○○ Low | 0.336710348 | ⊕⊕○○ Low |

| | | | | | | | | |
|---|---|---|---|---|---|---|---|---|
| 86 | Dapagliflozin_high_dosage:Exenatide | 0 | | | 0.862378713 | ⊕⊕○○ Low | 0.862378713 | ⊕⊕○○ Low |
| 87 | Dapagliflozin_high_dosage:Inject_semaglutide_high_dosage | 0 | | | 0.30827927 | ⊕⊕○○ Low | 0.30827927 | ⊕⊕○○ Low |
| 88 | Dapagliflozin_high_dosage:Inject_semaglutide_low_dosage | 0 | | | 1.169078141 | ⊕⊕○○ Low | 1.169078141 | ⊕⊕○○ Low |
| 89 | Dapagliflozin_high_dosage:Inject_semaglutide_medium_dosage | 0 | | | 1.316302827 | ⊕⊕○○ Low | 1.316302827 | ⊕⊕○○ Low |
| 90 | Dapagliflozin_high_dosage:Liraglutide | 0 | | | 0.378321516 | ⊕⊕○○ Low | 0.378321516 | ⊕⊕○○ Low |
| 91 | Dapagliflozin_high_dosage:Lixisenatide | 0 | | | 1.531552937 | ⊕⊕○○ Low | 1.531552937 | ⊕⊕○○ Low |
| 92 | Dapagliflozin_high_dosage:Oral_semaglutide | 0 | | | 0.923293172 | ⊕⊕○○ Low | 0.923293172 | ⊕⊕○○ Low |
| 93 | Dapagliflozin_high_dosage:Placebo_or_Control | 3 | 0.614601703 | ⊕⊕⊕⊕ High | | | 0.614601703 | ⊕⊕⊕○ Medium |
| 94 | Dapagliflozin_high_dosage:Sotagliflozin | 0 | | | 0.454047624 | ⊕⊕○○ Low | 0.454047624 | ⊕⊕○○ Low |
| 95 | Dapagliflozin_high_dosage:Tirzepatide | 0 | | | 2.334958158 | ⊕⊕○○ Low | 2.334958158 | ⊕⊕○○ Low |
| 96 | Dulaglutide:Efpeglenatide_high_dosage | 0 | | | -0.297950791 | ⊕⊕○○ Low | -0.297950791 | ⊕⊕○○ Low |
| 97 | Dulaglutide:Efpeglenatide_medium_dosage | 0 | | | -0.296478579 | ⊕⊕○○ Low | -0.296478579 | ⊕⊕○○ Low |
| 98 | Dulaglutide:Empagliflozin_high_dosage | 0 | | | 1.590756666 | ⊕⊕○○ Low | 1.590756666 | ⊕⊕○○ Low |
| 99 | Dulaglutide:Empagliflozin_low_dosage | 0 | | | 0.431037134 | ⊕⊕○○ Low | 0.431037134 | ⊕⊕○○ Low |
| 100 | Dulaglutide:Ertugliflozin_high_dosage | 0 | | | 0.297599476 | ⊕⊕○○ Low | 0.297599476 | ⊕⊕○○ Low |
| 101 | Dulaglutide:Ertugliflozin_low_dosage | 0 | | | 0.52129576 | ⊕⊕○○ Low | 0.52129576 | ⊕⊕○○ Low |
| 102 | Dulaglutide:Exenatide | 0 | | | 1.046964125 | ⊕⊕○○ Low | 1.046964125 | ⊕⊕○○ Low |
| 103 | Dulaglutide:Inject_semaglutide_high_dosage | 0 | | | 0.492864682 | ⊕⊕○○ Low | 0.492864682 | ⊕⊕○○ Low |
| 104 | Dulaglutide:Inject_semaglutide_low_dosage | 0 | | | 1.353663553 | ⊕⊕○○ Low | 1.353663553 | ⊕⊕○○ Low |
| 105 | Dulaglutide:Inject_semaglutide_medium_dosage | 0 | | | 1.500888239 | ⊕⊕○○ Low | 1.500888239 | ⊕⊕○○ Low |
| 106 | Dulaglutide:Liraglutide | 0 | | | 0.562906927 | ⊕⊕○○ Low | 0.562906927 | ⊕⊕○○ Low |
| 107 | Dulaglutide:Lixisenatide | 0 | | | 1.716138349 | ⊕⊕○○ Low | 1.716138349 | ⊕⊕○○ Low |

| | | | | | | | | |
|---|---|---|---|---|---|---|---|---|
| 108 | Dulaglutide:Oral_semaglutide | 0 | | | 1.107878583 | ⊕⊕○○ Low | 1.107878583 | ⊕⊕○○ Low |
| 109 | Dulaglutide:Placebo_or_Control | 2 | 0.799187115 | ⊕⊕⊕⊕ High | | | 0.799187115 | ⊕⊕⊕○ Medium |
| 110 | Dulaglutide:Sotagliflozin | 0 | | | 0.638633036 | ⊕⊕○○ Low | 0.638633036 | ⊕⊕○○ Low |
| 111 | Dulaglutide:Tirzepatide | 0 | | | 2.519543569 | ⊕⊕○○ Low | 2.519543569 | ⊕⊕○○ Low |
| 112 | Efpeglenatide_high_dosage:Efpeglenatide_medium_dosage | 1 | 0.001472212 | ⊕⊕⊕⊕ High | | | 0.001472212 | ⊕⊕⊕○ Medium |
| 113 | Efpeglenatide_high_dosage:Empagliflozin_high_dosage | 0 | | | 1.888707457 | ⊕⊕○○ Low | 1.888707457 | ⊕⊕○○ Low |
| 114 | Efpeglenatide_high_dosage:Empagliflozin_low_dosage | 0 | | | 0.728987925 | ⊕⊕○○ Low | 0.728987925 | ⊕⊕○○ Low |
| 115 | Efpeglenatide_high_dosage:Ertugliflozin_high_dosage | 0 | | | 0.595550267 | ⊕⊕○○ Low | 0.595550267 | ⊕⊕○○ Low |
| 116 | Efpeglenatide_high_dosage:Ertugliflozin_low_dosage | 0 | | | 0.819246551 | ⊕⊕○○ Low | 0.819246551 | ⊕⊕○○ Low |
| 117 | Efpeglenatide_high_dosage:Exenatide | 0 | | | 1.344914915 | ⊕⊕○○ Low | 1.344914915 | ⊕⊕○○ Low |
| 118 | Efpeglenatide_high_dosage:Inject_semaglutide_high_dosage | 0 | | | 0.790815473 | ⊕⊕○○ Low | 0.790815473 | ⊕⊕○○ Low |
| 119 | Efpeglenatide_high_dosage:Inject_semaglutide_low_dosage | 0 | | | 1.651614344 | ⊕⊕○○ Low | 1.651614344 | ⊕⊕○○ Low |
| 120 | Efpeglenatide_high_dosage:Inject_semaglutide_medium_dosage | 0 | | | 1.79883903 | ⊕⊕○○ Low | 1.79883903 | ⊕⊕○○ Low |
| 121 | Efpeglenatide_high_dosage:Liraglutide | 0 | | | 0.860857718 | ⊕⊕○○ Low | 0.860857718 | ⊕⊕○○ Low |
| 122 | Efpeglenatide_high_dosage:Lixisenatide | 0 | | | 2.01408914 | ⊕⊕○○ Low | 2.01408914 | ⊕⊕○○ Low |
| 123 | Efpeglenatide_high_dosage:Oral_semaglutide | 0 | | | 1.405829374 | ⊕⊕○○ Low | 1.405829374 | ⊕⊕○○ Low |
| 124 | Efpeglenatide_high_dosage:Placebo_or_Control | 1 | 1.097137906 | ⊕⊕⊕⊕ High | | | 1.097137906 | ⊕⊕⊕○ Medium |
| 125 | Efpeglenatide_high_dosage:Sotagliflozin | 0 | | | 0.936583827 | ⊕⊕○○ Low | 0.936583827 | ⊕⊕○○ Low |
| 126 | Efpeglenatide_high_dosage:Tirzepatide | 0 | | | 2.81749436 | ⊕⊕○○ Low | 2.81749436 | ⊕⊕○○ Low |
| 127 | Efpeglenatide_medium_dosage:Empagliflozin_high_dosage | 0 | | | 1.887235245 | ⊕⊕○○ Low | 1.887235245 | ⊕⊕○○ Low |
| 128 | Efpeglenatide_medium_dosage:Empagliflozin_low_dosage | 0 | | | 0.727515713 | ⊕⊕○○ Low | 0.727515713 | ⊕⊕○○ Low |
| 129 | Efpeglenatide_medium_dosage:Ertugliflozin_high_dosage | 0 | | | 0.594078055 | ⊕⊕○○ Low | 0.594078055 | ⊕⊕○○ Low |

| | | | | | | | | |
|---|---|---|---|---|---|---|---|---|
| 130 | Efpeglenatide_medium_dosage:Ertugliflozin_low_dosage | 0 | | | 0.817774339 | ⊕⊕○○ Low | 0.817774339 | ⊕⊕○○ Low |
| 131 | Efpeglenatide_medium_dosage:Exenatide | 0 | | | 1.343442703 | ⊕⊕○○ Low | 1.343442703 | ⊕⊕○○ Low |
| 132 | Efpeglenatide_medium_dosage:Inject_semaglutide_high_dosage | 0 | | | 0.789343261 | ⊕⊕○○ Low | 0.789343261 | ⊕⊕○○ Low |
| 133 | Efpeglenatide_medium_dosage:Inject_semaglutide_low_dosage | 0 | | | 1.650142132 | ⊕⊕○○ Low | 1.650142132 | ⊕⊕○○ Low |
| 134 | Efpeglenatide_medium_dosage:Inject_semaglutide_medium_dosage | 0 | | | 1.797366818 | ⊕⊕○○ Low | 1.797366818 | ⊕⊕○○ Low |
| 135 | Efpeglenatide_medium_dosage:Liraglutide | 0 | | | 0.859385506 | ⊕⊕○○ Low | 0.859385506 | ⊕⊕○○ Low |
| 136 | Efpeglenatide_medium_dosage:Lixisenatide | 0 | | | 2.012616927 | ⊕⊕○○ Low | 2.012616927 | ⊕⊕○○ Low |
| 137 | Efpeglenatide_medium_dosage:Oral_semaglutide | 0 | | | 1.404357162 | ⊕⊕○○ Low | 1.404357162 | ⊕⊕○○ Low |
| 138 | Efpeglenatide_medium_dosage:Placebo_or_Control | 1 | 1.095665694 | ⊕⊕⊕⊕ High | | | 1.095665694 | ⊕⊕⊕○ Medium |
| 139 | Efpeglenatide_medium_dosage:Sotagliflozin | 0 | | | 0.935111615 | ⊕⊕○○ Low | 0.935111615 | ⊕⊕○○ Low |
| 140 | Efpeglenatide_medium_dosage:Tirzepatide | 0 | | | 2.816022148 | ⊕⊕○○ Low | 2.816022148 | ⊕⊕○○ Low |
| 141 | Empagliflozin_high_dosage:Empagliflozin_low_dosage | 1 | -0.69229321 | ⊕⊕⊕⊕ High | -3.990419392 | ⊕⊕⊕⊕ High | -1.159719532 | ⊕⊕⊕⊕ High |
| 142 | Empagliflozin_high_dosage:Ertugliflozin_high_dosage | 0 | | | -1.29315719 | ⊕⊕○○ Low | -1.29315719 | ⊕⊕○○ Low |
| 143 | Empagliflozin_high_dosage:Ertugliflozin_low_dosage | 0 | | | -1.069460906 | ⊕⊕○○ Low | -1.069460906 | ⊕⊕○○ Low |
| 144 | Empagliflozin_high_dosage:Exenatide | 0 | | | -0.543792541 | ⊕⊕○○ Low | -0.543792541 | ⊕⊕○○ Low |
| 145 | Empagliflozin_high_dosage:Inject_semaglutide_high_dosage | 0 | | | -1.097891984 | ⊕⊕○○ Low | -1.097891984 | ⊕⊕○○ Low |
| 146 | Empagliflozin_high_dosage:Inject_semaglutide_low_dosage | 0 | | | -0.237093113 | ⊕⊕○○ Low | -0.237093113 | ⊕⊕○○ Low |
| 147 | Empagliflozin_high_dosage:Inject_semaglutide_medium_dosage | 0 | | | -0.089868427 | ⊕⊕○○ Low | -0.089868427 | ⊕⊕○○ Low |
| 148 | Empagliflozin_high_dosage:Liraglutide | 0 | | | -1.027849739 | ⊕⊕○○ Low | -1.027849739 | ⊕⊕○○ Low |
| 149 | Empagliflozin_high_dosage:Lixisenatide | 0 | | | 0.125381683 | ⊕⊕○○ Low | 0.125381683 | ⊕⊕○○ Low |
| 150 | Empagliflozin_high_dosage:Oral_semaglutide | 0 | | | -0.482878083 | ⊕⊕○○ Low | -0.482878083 | ⊕⊕○○ Low |
| 151 | Empagliflozin_high_dosage:Placebo_or_Control | 1 | -1.10332221 | ⊕⊕⊕⊕ High | 3.292594015 | ⊕⊕⊕○ Medium | -0.791569551 | ⊕⊕⊕⊕ High |

| | | | | | | | | |
|---|---|---|---|---|---|---|---|---|
| 152 | Empagliflozin_high_dosage:Sotagliflozin | 0 | | | -0.95212363 | ⊕⊕○○ Low | -0.95212363 | ⊕⊕○○ Low |
| 153 | Empagliflozin_high_dosage:Tirzepatide | 0 | | | 0.928786903 | ⊕⊕○○ Low | 0.928786903 | ⊕⊕○○ Low |
| 154 | Empagliflozin_low_dosage:Ertugliflozin_high_dosage | 0 | | | -0.133437658 | ⊕⊕○○ Low | -0.133437658 | ⊕⊕○○ Low |
| 155 | Empagliflozin_low_dosage:Ertugliflozin_low_dosage | 0 | | | 0.090258626 | ⊕⊕○○ Low | 0.090258626 | ⊕⊕○○ Low |
| 156 | Empagliflozin_low_dosage:Exenatide | 0 | | | 0.61592699 | ⊕⊕○○ Low | 0.61592699 | ⊕⊕○○ Low |
| 157 | Empagliflozin_low_dosage:Inject_semaglutide_high_dosage | 0 | | | 0.061827548 | ⊕⊕○○ Low | 0.061827548 | ⊕⊕○○ Low |
| 158 | Empagliflozin_low_dosage:Inject_semaglutide_low_dosage | 0 | | | 0.922626419 | ⊕⊕○○ Low | 0.922626419 | ⊕⊕○○ Low |
| 159 | Empagliflozin_low_dosage:Inject_semaglutide_medium_dosage | 0 | | | 1.069851105 | ⊕⊕○○ Low | 1.069851105 | ⊕⊕○○ Low |
| 160 | Empagliflozin_low_dosage:Liraglutide | 0 | | | 0.131869793 | ⊕⊕○○ Low | 0.131869793 | ⊕⊕○○ Low |
| 161 | Empagliflozin_low_dosage:Lixisenatide | 0 | | | 1.285101215 | ⊕⊕○○ Low | 1.285101215 | ⊕⊕○○ Low |
| 162 | Empagliflozin_low_dosage:Oral_semaglutide | 0 | | | 0.676841449 | ⊕⊕○○ Low | 0.676841449 | ⊕⊕○○ Low |
| 163 | Empagliflozin_low_dosage:Placebo_or_Control | 4 | 0.368149981 | ⊕⊕⊕⊕ High | | | 0.368149981 | ⊕⊕⊕○ Medium |
| 164 | Empagliflozin_low_dosage:Sotagliflozin | 0 | | | 0.207595902 | ⊕⊕○○ Low | 0.207595902 | ⊕⊕○○ Low |
| 165 | Empagliflozin_low_dosage:Tirzepatide | 0 | | | 2.088506435 | ⊕⊕○○ Low | 2.088506435 | ⊕⊕○○ Low |
| 166 | Ertugliflozin_high_dosage:Ertugliflozin_low_dosage | 2 | 0.176697429 | ⊕⊕⊕⊕ High | 1.622225494 | ⊕⊕⊕⊕ High | 0.223696284 | ⊕⊕⊕⊕ High |
| 167 | Ertugliflozin_high_dosage:Exenatide | 0 | | | 0.749364649 | ⊕⊕○○ Low | 0.749364649 | ⊕⊕○○ Low |
| 168 | Ertugliflozin_high_dosage:Inject_semaglutide_high_dosage | 0 | | | 0.195265206 | ⊕⊕○○ Low | 0.195265206 | ⊕⊕○○ Low |
| 169 | Ertugliflozin_high_dosage:Inject_semaglutide_low_dosage | 0 | | | 1.056064077 | ⊕⊕○○ Low | 1.056064077 | ⊕⊕○○ Low |
| 170 | Ertugliflozin_high_dosage:Inject_semaglutide_medium_dosage | 0 | | | 1.203288763 | ⊕⊕○○ Low | 1.203288763 | ⊕⊕○○ Low |
| 171 | Ertugliflozin_high_dosage:Liraglutide | 0 | | | 0.265307451 | ⊕⊕○○ Low | 0.265307451 | ⊕⊕○○ Low |
| 172 | Ertugliflozin_high_dosage:Lixisenatide | 0 | | | 1.418538873 | ⊕⊕○○ Low | 1.418538873 | ⊕⊕○○ Low |
| 173 | Ertugliflozin_high_dosage:Oral_semaglutide | 0 | | | 0.810279107 | ⊕⊕○○ Low | 0.810279107 | ⊕⊕○○ Low |

| | | | | | | | | |
|---|---|---|---|---|---|---|---|---|
| 174 | Ertugliflozin_high_dosage:Placebo_or_Control | 2 | 0.432505637 | ⊕⊕⊕⊕ High | 2.910692852 | ⊕⊕⊕⊕ High | 0.501587639 | ⊕⊕⊕⊕ High |
| 175 | Ertugliflozin_high_dosage:Sotagliflozin | 0 | | | 0.34103356 | ⊕⊕○○ Low | 0.34103356 | ⊕⊕○○ Low |
| 176 | Ertugliflozin_high_dosage:Tirzepatide | 0 | | | 2.221944093 | ⊕⊕○○ Low | 2.221944093 | ⊕⊕○○ Low |
| 177 | Ertugliflozin_low_dosage:Exenatide | 0 | | | 0.525668365 | ⊕⊕○○ Low | 0.525668365 | ⊕⊕○○ Low |
| 178 | Ertugliflozin_low_dosage:Inject_semaglutide_high_dosage | 0 | | | -0.028431078 | ⊕⊕○○ Low | -0.028431078 | ⊕⊕○○ Low |
| 179 | Ertugliflozin_low_dosage:Inject_semaglutide_low_dosage | 0 | | | 0.832367793 | ⊕⊕○○ Low | 0.832367793 | ⊕⊕○○ Low |
| 180 | Ertugliflozin_low_dosage:Inject_semaglutide_medium_dosage | 0 | | | 0.979592479 | ⊕⊕○○ Low | 0.979592479 | ⊕⊕○○ Low |
| 181 | Ertugliflozin_low_dosage:Liraglutide | 0 | | | 0.041611167 | ⊕⊕○○ Low | 0.041611167 | ⊕⊕○○ Low |
| 182 | Ertugliflozin_low_dosage:Lixisenatide | 0 | | | 1.194842589 | ⊕⊕○○ Low | 1.194842589 | ⊕⊕○○ Low |
| 183 | Ertugliflozin_low_dosage:Oral_semaglutide | 0 | | | 0.586582823 | ⊕⊕○○ Low | 0.586582823 | ⊕⊕○○ Low |
| 184 | Ertugliflozin_low_dosage:Placebo_or_Control | 1 | 0.287682072 | ⊕⊕⊕⊕ High | 0.18944794 | ⊕⊕⊕⊕ High | 0.277891355 | ⊕⊕⊕⊕ High |
| 185 | Ertugliflozin_low_dosage:Sotagliflozin | 0 | | | 0.117337276 | ⊕⊕○○ Low | 0.117337276 | ⊕⊕○○ Low |
| 186 | Ertugliflozin_low_dosage:Tirzepatide | 0 | | | 1.998247809 | ⊕⊕○○ Low | 1.998247809 | ⊕⊕○○ Low |
| 187 | Exenatide:Inject_semaglutide_high_dosage | 0 | | | -0.554099442 | ⊕⊕○○ Low | -0.554099442 | ⊕⊕○○ Low |
| 188 | Exenatide:Inject_semaglutide_low_dosage | 0 | | | 0.306699429 | ⊕⊕○○ Low | 0.306699429 | ⊕⊕○○ Low |
| 189 | Exenatide:Inject_semaglutide_medium_dosage | 0 | | | 0.453924114 | ⊕⊕○○ Low | 0.453924114 | ⊕⊕○○ Low |
| 190 | Exenatide:Liraglutide | 0 | | | -0.484057197 | ⊕⊕○○ Low | -0.484057197 | ⊕⊕○○ Low |
| 191 | Exenatide:Lixisenatide | 0 | | | 0.669174224 | ⊕⊕○○ Low | 0.669174224 | ⊕⊕○○ Low |
| 192 | Exenatide:Oral_semaglutide | 0 | | | 0.060914459 | ⊕⊕○○ Low | 0.060914459 | ⊕⊕○○ Low |
| 193 | Exenatide:Placebo_or_Control | 1 | -0.24777701 | ⊕⊕⊕⊕ High | | | -0.24777701 | ⊕⊕⊕○ Medium |
| 194 | Exenatide:Sotagliflozin | 0 | | | -0.408331089 | ⊕⊕○○ Low | -0.408331089 | ⊕⊕○○ Low |
| 195 | Exenatide:Tirzepatide | 0 | | | 1.472579445 | ⊕⊕○○ Low | 1.472579445 | ⊕⊕○○ Low |

| | | | | | | | | |
|---|---|---|---|---|---|---|---|---|
| 196 | Inject_semaglutide_high_dosage:Inject_semaglutide_low_dosage | 0 | | | 0.860798871 | ⨁⨁○○ Low | 0.860798871 | ⨁⨁○○ Low |
| 197 | Inject_semaglutide_high_dosage:Inject_semaglutide_medium_dosage | 1 | 1.098612289 | ⨁⨁⨁⨁ High | 0.97817013 | ⨁⨁⨁⨁ High | 1.008023557 | ⨁⨁⨁⨁ High |
| 198 | Inject_semaglutide_high_dosage:Liraglutide | 0 | | | 0.070042245 | ⨁⨁○○ Low | 0.070042245 | ⨁⨁○○ Low |
| 199 | Inject_semaglutide_high_dosage:Lixisenatide | 0 | | | 1.223273667 | ⨁⨁○○ Low | 1.223273667 | ⨁⨁○○ Low |
| 200 | Inject_semaglutide_high_dosage:Oral_semaglutide | 0 | | | 0.615013901 | ⨁⨁○○ Low | 0.615013901 | ⨁⨁○○ Low |
| 201 | Inject_semaglutide_high_dosage:Placebo_or_Control | 3 | 0.299787256 | ⨁⨁⨁⨁ High | 0.420229414 | ⨁⨁⨁⨁ High | 0.306322433 | ⨁⨁⨁⨁ High |
| 202 | Inject_semaglutide_high_dosage:Sotagliflozin | 0 | | | 0.145768354 | ⨁⨁○○ Low | 0.145768354 | ⨁⨁○○ Low |
| 203 | Inject_semaglutide_high_dosage:Tirzepatide | 0 | | | 2.026678887 | ⨁⨁○○ Low | 2.026678887 | ⨁⨁○○ Low |
| 204 | Inject_semaglutide_low_dosage:Inject_semaglutide_medium_dosage | 2 | 0.472892936 | ⨁⨁⨁⨁ High | -1.230319618 | ⨁⨁⨁○ Medium | 0.147224686 | ⨁⨁⨁⨁ High |
| 205 | Inject_semaglutide_low_dosage:Liraglutide | 0 | | | -0.790756626 | ⨁⨁○○ Low | -0.790756626 | ⨁⨁○○ Low |
| 206 | Inject_semaglutide_low_dosage:Lixisenatide | 0 | | | 0.362474796 | ⨁⨁○○ Low | 0.362474796 | ⨁⨁○○ Low |
| 207 | Inject_semaglutide_low_dosage:Oral_semaglutide | 0 | | | -0.24578497 | ⨁⨁○○ Low | -0.24578497 | ⨁⨁○○ Low |
| 208 | Inject_semaglutide_low_dosage:Placebo_or_Control | 2 | -0.693710386 | ⨁⨁⨁⨁ High | 2.797394006 | ⨁⨁⨁○ Medium | -0.554476438 | ⨁⨁⨁⨁ High |
| 209 | Inject_semaglutide_low_dosage:Sotagliflozin | 0 | | | -0.715030517 | ⨁⨁○○ Low | -0.715030517 | ⨁⨁○○ Low |
| 210 | Inject_semaglutide_low_dosage:Tirzepatide | 0 | | | 1.165880016 | ⨁⨁○○ Low | 1.165880016 | ⨁⨁○○ Low |
| 211 | Inject_semaglutide_medium_dosage:Liraglutide | 0 | | | -0.937981312 | ⨁⨁○○ Low | -0.937981312 | ⨁⨁○○ Low |
| 212 | Inject_semaglutide_medium_dosage:Lixisenatide | 0 | | | 0.21525011 | ⨁⨁○○ Low | 0.21525011 | ⨁⨁○○ Low |
| 213 | Inject_semaglutide_medium_dosage:Oral_semaglutide | 0 | | | -0.393009656 | ⨁⨁○○ Low | -0.393009656 | ⨁⨁○○ Low |
| 214 | Inject_semaglutide_medium_dosage:Placebo_or_Control | 2 | -1.045069515 | ⨁⨁⨁⨁ High | -0.06405328 | ⨁⨁⨁⨁ High | -0.701701124 | ⨁⨁⨁⨁ High |
| 215 | Inject_semaglutide_medium_dosage:Sotagliflozin | 0 | | | -0.862255203 | ⨁⨁○○ Low | -0.862255203 | ⨁⨁○○ Low |
| 216 | Inject_semaglutide_medium_dosage:Tirzepatide | 1 | 2.200066498 | ⨁⨁⨁⨁ High | 0.333932731 | ⨁⨁⨁⨁ High | 1.01865533 | ⨁⨁⨁⨁ High |
| 217 | Liraglutide:Lixisenatide | 0 | | | 1.153231421 | ⨁⨁○○ Low | 1.153231421 | ⨁⨁○○ Low |

| | | | | | | | | |
|---|---|---|---|---|---|---|---|---|
| 218 | Liraglutide:Oral_semaglutide | 0 | | | 0.544971656 | ⊕⊕○○ Low | 0.544971656 | ⊕⊕○○ Low |
| 219 | Liraglutide:Placebo_or_Control | 3 | 0.236280188 | ⊕⊕⊕⊕ High | | | 0.236280188 | ⊕⊕⊕○ Medium |
| 220 | Liraglutide:Sotagliflozin | 0 | | | 0.075726109 | ⊕⊕○○ Low | 0.075726109 | ⊕⊕○○ Low |
| 221 | Liraglutide:Tirzepatide | 0 | | | 1.956636642 | ⊕⊕○○ Low | 1.956636642 | ⊕⊕○○ Low |
| 222 | Lixisenatide:Oral_semaglutide | 0 | | | -0.608259765 | ⊕⊕○○ Low | -0.608259765 | ⊕⊕○○ Low |
| 223 | Lixisenatide:Placebo_or_Control | 1 | -0.916951234 | ⊕⊕⊕⊕ High | | | -0.916951234 | ⊕⊕⊕○ Medium |
| 224 | Lixisenatide:Sotagliflozin | 0 | | | -1.077505313 | ⊕⊕○○ Low | -1.077505313 | ⊕⊕○○ Low |
| 225 | Lixisenatide:Tirzepatide | 0 | | | 0.80340522 | ⊕⊕○○ Low | 0.80340522 | ⊕⊕○○ Low |
| 226 | Oral_semaglutide:Placebo_or_Control | 2 | -0.308691468 | ⊕⊕⊕⊕ High | | | -0.308691468 | ⊕⊕⊕○ Medium |
| 227 | Oral_semaglutide:Sotagliflozin | 0 | | | -0.469245547 | ⊕⊕○○ Low | -0.469245547 | ⊕⊕○○ Low |
| 228 | Oral_semaglutide:Tirzepatide | 0 | | | 1.411664986 | ⊕⊕○○ Low | 1.411664986 | ⊕⊕○○ Low |
| 229 | Sotagliflozin:Placebo_or_Control | 4 | 0.160554079 | ⊕⊕⊕⊕ High | | | 0.160554079 | ⊕⊕⊕○ Medium |
| 230 | Tirzepatide:Placebo_or_Control | 3 | -1.326345037 | ⊕⊕⊕⊕ High | -3.192478804 | ⊕⊕⊕⊕ High | -1.720356454 | ⊕⊕⊕⊕ High |
| 231 | Sotagliflozin:Tirzepatide | 0 | | | 1.880910533 | ⊕⊕○○ Low | 1.880910533 | ⊕⊕○○ Low |

## Table S8C: GRADE of primary outcome: subgroup of leukemia

| | Comparison | No.Studies | Direct | | Indirect | | NMA | |
|---|---|---|---|---|---|---|---|---|
| | | | Estimate | Rate | Estimate | Rate | Estimate | Rate |
| 1 | Albiglutide:Canagliflozin_high_dosage | 0 | | | 1.223513314 | ⊕⊕○○ Low | 1.223513314 | ⊕⊕○○ Low |
| 2 | Albiglutide:Canagliflozin_low_dosage | 0 | | | 0.842125983 | ⊕⊕○○ Low | 0.842125983 | ⊕⊕○○ Low |
| 3 | Albiglutide:Dapagliflozin_high_dosage | 0 | | | 0.144456124 | ⊕⊕○○ Low | 0.144456124 | ⊕⊕○○ Low |
| 4 | Albiglutide:Dapagliflozin_low_dosage | 0 | | | 1.334968749 | ⊕⊕○○ Low | 1.334968749 | ⊕⊕○○ Low |
| 5 | Albiglutide:Dapagliflozin_medium_dosage | 0 | | | 1.359091903 | ⊕⊕○○ Low | 1.359091903 | ⊕⊕○○ Low |
| 6 | Albiglutide:Dulaglutide | 0 | | | -0.294178075 | ⊕⊕○○ Low | -0.294178075 | ⊕⊕○○ Low |
| 7 | Albiglutide:Efpeglenatide_high_dosage | 0 | | | 0.219869124 | ⊕⊕○○ Low | 0.219869124 | ⊕⊕○○ Low |
| 8 | Albiglutide:Efpeglenatide_medium_dosage | 0 | | | -0.878007329 | ⊕⊕○○ Low | -0.878007329 | ⊕⊕○○ Low |
| 9 | Albiglutide:Empagliflozin_high_dosage | 0 | | | 0.212145468 | ⊕⊕○○ Low | 0.212145468 | ⊕⊕○○ Low |
| 10 | Albiglutide:Empagliflozin_low_dosage | 0 | | | -0.748590905 | ⊕⊕○○ Low | -0.748590905 | ⊕⊕○○ Low |
| 11 | Albiglutide:Ertugliflozin_high_dosage | 0 | | | -0.880953925 | ⊕⊕○○ Low | -0.880953925 | ⊕⊕○○ Low |
| 12 | Albiglutide:Ertugliflozin_low_dosage | 0 | | | -0.475488816 | ⊕⊕○○ Low | -0.475488816 | ⊕⊕○○ Low |
| 13 | Albiglutide:Exenatide | 0 | | | -0.105013921 | ⊕⊕○○ Low | -0.105013921 | ⊕⊕○○ Low |
| 14 | Albiglutide:Inject_semaglutide_high_dosage | 0 | | | 0.334534808 | ⊕⊕○○ Low | 0.334534808 | ⊕⊕○○ Low |
| 15 | Albiglutide:Inject_semaglutide_low_dosage | 0 | | | 1.558340002 | ⊕⊕○○ Low | 1.558340002 | ⊕⊕○○ Low |
| 16 | Albiglutide:Inject_semaglutide_medium_dosage | 0 | | | 0.649551352 | ⊕⊕○○ Low | 0.649551352 | ⊕⊕○○ Low |
| 17 | Albiglutide:Liraglutide | 0 | | | 0.696148179 | ⊕⊕○○ Low | 0.696148179 | ⊕⊕○○ Low |
| 18 | Albiglutide:Lixisenatide | 0 | | | 0.894184356 | ⊕⊕○○ Low | 0.894184356 | ⊕⊕○○ Low |
| 19 | Albiglutide:Oral_semaglutide | 0 | | | 1.316899188 | ⊕⊕○○ Low | 1.316899188 | ⊕⊕○○ Low |

| | | | | | | | | |
|---|---|---|---|---|---|---|---|---|
| 20 | Albiglutide:Placebo_or_Control | 3 | 0.217658364 | ⊕⊕⊕⊕ High | | | 0.217658364 | ⊕⊕⊕○ Medium |
| 21 | Albiglutide:Sotagliflozin | 0 | | | 0.21860417 | ⊕⊕○○ Low | 0.21860417 | ⊕⊕○○ Low |
| 22 | Albiglutide:Tirzepatide | 0 | | | 2.000635342 | ⊕⊕○○ Low | 2.000635342 | ⊕⊕○○ Low |
| 23 | Canagliflozin_high_dosage:Canagliflozin_low_dosage | 1 | 0.002773927 | ⊕⊕⊕⊕ High | -1.022025807 | ⊕⊕⊕○ Medium | -0.381387331 | ⊕⊕⊕⊕ High |
| 24 | Canagliflozin_high_dosage:Dapagliflozin_high_dosage | 0 | | | -1.07905719 | ⊕⊕○○ Low | -1.07905719 | ⊕⊕○○ Low |
| 25 | Canagliflozin_high_dosage:Dapagliflozin_low_dosage | 0 | | | 0.111455435 | ⊕⊕○○ Low | 0.111455435 | ⊕⊕○○ Low |
| 26 | Canagliflozin_high_dosage:Dapagliflozin_medium_dosage | 0 | | | 0.13557859 | ⊕⊕○○ Low | 0.13557859 | ⊕⊕○○ Low |
| 27 | Canagliflozin_high_dosage:Dulaglutide | 0 | | | -1.517691389 | ⊕⊕○○ Low | -1.517691389 | ⊕⊕○○ Low |
| 28 | Canagliflozin_high_dosage:Efpeglenatide_high_dosage | 0 | | | -1.00364419 | ⊕⊕○○ Low | -1.00364419 | ⊕⊕○○ Low |
| 29 | Canagliflozin_high_dosage:Efpeglenatide_medium_dosage | 0 | | | -2.101520643 | ⊕⊕○○ Low | -2.101520643 | ⊕⊕○○ Low |
| 30 | Canagliflozin_high_dosage:Empagliflozin_high_dosage | 0 | | | -1.011367845 | ⊕⊕○○ Low | -1.011367845 | ⊕⊕○○ Low |
| 31 | Canagliflozin_high_dosage:Empagliflozin_low_dosage | 0 | | | -1.972104219 | ⊕⊕○○ Low | -1.972104219 | ⊕⊕○○ Low |
| 32 | Canagliflozin_high_dosage:Ertugliflozin_high_dosage | 0 | | | -2.104467238 | ⊕⊕○○ Low | -2.104467238 | ⊕⊕○○ Low |
| 33 | Canagliflozin_high_dosage:Ertugliflozin_low_dosage | 0 | | | -1.69900213 | ⊕⊕○○ Low | -1.69900213 | ⊕⊕○○ Low |
| 34 | Canagliflozin_high_dosage:Exenatide | 0 | | | -1.328527235 | ⊕⊕○○ Low | -1.328527235 | ⊕⊕○○ Low |
| 35 | Canagliflozin_high_dosage:Inject_semaglutide_high_dosage | 0 | | | -0.888978506 | ⊕⊕○○ Low | -0.888978506 | ⊕⊕○○ Low |
| 36 | Canagliflozin_high_dosage:Inject_semaglutide_low_dosage | 0 | | | 0.334826688 | ⊕⊕○○ Low | 0.334826688 | ⊕⊕○○ Low |
| 37 | Canagliflozin_high_dosage:Inject_semaglutide_medium_dosage | 0 | | | -0.573961962 | ⊕⊕○○ Low | -0.573961962 | ⊕⊕○○ Low |
| 38 | Canagliflozin_high_dosage:Liraglutide | 0 | | | -0.527365135 | ⊕⊕○○ Low | -0.527365135 | ⊕⊕○○ Low |
| 39 | Canagliflozin_high_dosage:Lixisenatide | 0 | | | -0.329328957 | ⊕⊕○○ Low | -0.329328957 | ⊕⊕○○ Low |
| 40 | Canagliflozin_high_dosage:Oral_semaglutide | 0 | | | 0.093385875 | ⊕⊕○○ Low | 0.093385875 | ⊕⊕○○ Low |
| 41 | Canagliflozin_high_dosage:Placebo_or_Control | 2 | -1.046530259 | ⊕⊕⊕⊕ High | -0.023925797 | ⊕⊕⊕⊕ High | -1.00585495 | ⊕⊕⊕⊕ High |

| | | | | | | | | |
|---|---|---|---|---|---|---|---|---|
| 42 | Canagliflozin_high_dosage:Sotagliflozin | 0 | | | -1.004909144 | ⊕⊕○○ Low | -1.004909144 | ⊕⊕○○ Low |
| 43 | Canagliflozin_high_dosage:Tirzepatide | 0 | | | 0.777122028 | ⊕⊕○○ Low | 0.777122028 | ⊕⊕○○ Low |
| 44 | Canagliflozin_low_dosage:Dapagliflozin_high_dosage | 0 | | | -0.697669859 | ⊕⊕○○ Low | -0.697669859 | ⊕⊕○○ Low |
| 45 | Canagliflozin_low_dosage:Dapagliflozin_low_dosage | 0 | | | 0.492842766 | ⊕⊕○○ Low | 0.492842766 | ⊕⊕○○ Low |
| 46 | Canagliflozin_low_dosage:Dapagliflozin_medium_dosage | 0 | | | 0.51696592 | ⊕⊕○○ Low | 0.51696592 | ⊕⊕○○ Low |
| 47 | Canagliflozin_low_dosage:Dulaglutide | 0 | | | -1.136304058 | ⊕⊕○○ Low | -1.136304058 | ⊕⊕○○ Low |
| 48 | Canagliflozin_low_dosage:Efpeglenatide_high_dosage | 0 | | | -0.622256859 | ⊕⊕○○ Low | -0.622256859 | ⊕⊕○○ Low |
| 49 | Canagliflozin_low_dosage:Efpeglenatide_medium_dosage | 0 | | | -1.720133312 | ⊕⊕○○ Low | -1.720133312 | ⊕⊕○○ Low |
| 50 | Canagliflozin_low_dosage:Empagliflozin_high_dosage | 0 | | | -0.629980515 | ⊕⊕○○ Low | -0.629980515 | ⊕⊕○○ Low |
| 51 | Canagliflozin_low_dosage:Empagliflozin_low_dosage | 0 | | | -1.590716888 | ⊕⊕○○ Low | -1.590716888 | ⊕⊕○○ Low |
| 52 | Canagliflozin_low_dosage:Ertugliflozin_high_dosage | 0 | | | -1.723079908 | ⊕⊕○○ Low | -1.723079908 | ⊕⊕○○ Low |
| 53 | Canagliflozin_low_dosage:Ertugliflozin_low_dosage | 0 | | | -1.317614799 | ⊕⊕○○ Low | -1.317614799 | ⊕⊕○○ Low |
| 54 | Canagliflozin_low_dosage:Exenatide | 0 | | | -0.947139904 | ⊕⊕○○ Low | -0.947139904 | ⊕⊕○○ Low |
| 55 | Canagliflozin_low_dosage:Inject_semaglutide_high_dosage | 0 | | | -0.507591176 | ⊕⊕○○ Low | -0.507591176 | ⊕⊕○○ Low |
| 56 | Canagliflozin_low_dosage:Inject_semaglutide_low_dosage | 0 | | | 0.716214019 | ⊕⊕○○ Low | 0.716214019 | ⊕⊕○○ Low |
| 57 | Canagliflozin_low_dosage:Inject_semaglutide_medium_dosage | 0 | | | -0.192574631 | ⊕⊕○○ Low | -0.192574631 | ⊕⊕○○ Low |
| 58 | Canagliflozin_low_dosage:Liraglutide | 0 | | | -0.145977804 | ⊕⊕○○ Low | -0.145977804 | ⊕⊕○○ Low |
| 59 | Canagliflozin_low_dosage:Lixisenatide | 0 | | | 0.052058373 | ⊕⊕○○ Low | 0.052058373 | ⊕⊕○○ Low |
| 60 | Canagliflozin_low_dosage:Oral_semaglutide | 0 | | | 0.474773205 | ⊕⊕○○ Low | 0.474773205 | ⊕⊕○○ Low |
| 61 | Canagliflozin_low_dosage:Placebo_or_Control | 3 | -0.569893819 | ⊕⊕⊕⊕ High | -3.445227555 | ⊕⊕⊕⊕ High | -0.624467619 | ⊕⊕⊕⊕ High |
| 62 | Canagliflozin_low_dosage:Sotagliflozin | 0 | | | -0.623521813 | ⊕⊕○○ Low | -0.623521813 | ⊕⊕○○ Low |
| 63 | Canagliflozin_low_dosage:Tirzepatide | 0 | | | 1.158509359 | ⊕⊕○○ Low | 1.158509359 | ⊕⊕○○ Low |

| | | | | | | | | |
|---|---|---|---|---|---|---|---|---|
| 64 | Dapagliflozin_high_dosage:Dapagliflozin_low_dosage | 0 | | | 1.190512625 | ⊕⊕○○ Low | 1.190512625 | ⊕⊕○○ Low |
| 65 | Dapagliflozin_high_dosage:Dapagliflozin_medium_dosage | 0 | | | 1.21463578 | ⊕⊕○○ Low | 1.21463578 | ⊕⊕○○ Low |
| 66 | Dapagliflozin_high_dosage:Dulaglutide | 0 | | | -0.438634199 | ⊕⊕○○ Low | -0.438634199 | ⊕⊕○○ Low |
| 67 | Dapagliflozin_high_dosage:Efpeglenatide_high_dosage | 0 | | | 0.075413 | ⊕⊕○○ Low | 0.075413 | ⊕⊕○○ Low |
| 68 | Dapagliflozin_high_dosage:Efpeglenatide_medium_dosage | 0 | | | -1.022463453 | ⊕⊕○○ Low | -1.022463453 | ⊕⊕○○ Low |
| 69 | Dapagliflozin_high_dosage:Empagliflozin_high_dosage | 0 | | | 0.067689345 | ⊕⊕○○ Low | 0.067689345 | ⊕⊕○○ Low |
| 70 | Dapagliflozin_high_dosage:Empagliflozin_low_dosage | 0 | | | -0.893047029 | ⊕⊕○○ Low | -0.893047029 | ⊕⊕○○ Low |
| 71 | Dapagliflozin_high_dosage:Ertugliflozin_high_dosage | 0 | | | -1.025410048 | ⊕⊕○○ Low | -1.025410048 | ⊕⊕○○ Low |
| 72 | Dapagliflozin_high_dosage:Ertugliflozin_low_dosage | 0 | | | -0.61994494 | ⊕⊕○○ Low | -0.61994494 | ⊕⊕○○ Low |
| 73 | Dapagliflozin_high_dosage:Exenatide | 0 | | | -0.249470045 | ⊕⊕○○ Low | -0.249470045 | ⊕⊕○○ Low |
| 74 | Dapagliflozin_high_dosage:Inject_semaglutide_high_dosage | 0 | | | 0.190078684 | ⊕⊕○○ Low | 0.190078684 | ⊕⊕○○ Low |
| 75 | Dapagliflozin_high_dosage:Inject_semaglutide_low_dosage | 0 | | | 1.413883878 | ⊕⊕○○ Low | 1.413883878 | ⊕⊕○○ Low |
| 76 | Dapagliflozin_high_dosage:Inject_semaglutide_medium_dosage | 0 | | | 0.505095228 | ⊕⊕○○ Low | 0.505095228 | ⊕⊕○○ Low |
| 77 | Dapagliflozin_high_dosage:Liraglutide | 0 | | | 0.551692055 | ⊕⊕○○ Low | 0.551692055 | ⊕⊕○○ Low |
| 78 | Dapagliflozin_high_dosage:Lixisenatide | 0 | | | 0.749728233 | ⊕⊕○○ Low | 0.749728233 | ⊕⊕○○ Low |
| 79 | Dapagliflozin_high_dosage:Oral_semaglutide | 0 | | | 1.172443065 | ⊕⊕○○ Low | 1.172443065 | ⊕⊕○○ Low |
| 80 | Dapagliflozin_high_dosage:Placebo_or_Control | 6 | 0.07320224 | ⊕⊕⊕⊕ High | | | 0.07320224 | ⊕⊕⊕○ Medium |
| 81 | Dapagliflozin_high_dosage:Sotagliflozin | 0 | | | 0.074148046 | ⊕⊕○○ Low | 0.074148046 | ⊕⊕○○ Low |
| 82 | Dapagliflozin_high_dosage:Tirzepatide | 0 | | | 1.856179218 | ⊕⊕○○ Low | 1.856179218 | ⊕⊕○○ Low |
| 83 | Dapagliflozin_low_dosage:Dapagliflozin_medium_dosage | 0 | | | 0.024123155 | ⊕⊕○○ Low | 0.024123155 | ⊕⊕○○ Low |
| 84 | Dapagliflozin_low_dosage:Dulaglutide | 0 | | | -1.629146824 | ⊕⊕○○ Low | -1.629146824 | ⊕⊕○○ Low |
| 85 | Dapagliflozin_low_dosage:Efpeglenatide_high_dosage | 0 | | | -1.115099625 | ⊕⊕○○ Low | -1.115099625 | ⊕⊕○○ Low |

| | | | | | | | | |
|---|---|---|---|---|---|---|---|---|
| 86 | Dapagliflozin_low_dosage:Efpeglenatide_medium_dosage | 0 | | | -2.212976078 | ⊕⊕○○ Low | -2.212976078 | ⊕⊕○○ Low |
| 87 | Dapagliflozin_low_dosage:Empagliflozin_high_dosage | 0 | | | -1.12282328 | ⊕⊕○○ Low | -1.12282328 | ⊕⊕○○ Low |
| 88 | Dapagliflozin_low_dosage:Empagliflozin_low_dosage | 0 | | | -2.083559654 | ⊕⊕○○ Low | -2.083559654 | ⊕⊕○○ Low |
| 89 | Dapagliflozin_low_dosage:Ertugliflozin_high_dosage | 0 | | | -2.215922673 | ⊕⊕○○ Low | -2.215922673 | ⊕⊕○○ Low |
| 90 | Dapagliflozin_low_dosage:Ertugliflozin_low_dosage | 0 | | | -1.810457565 | ⊕⊕○○ Low | -1.810457565 | ⊕⊕○○ Low |
| 91 | Dapagliflozin_low_dosage:Exenatide | 0 | | | -1.43998267 | ⊕⊕○○ Low | -1.43998267 | ⊕⊕○○ Low |
| 92 | Dapagliflozin_low_dosage:Inject_semaglutide_high_dosage | 0 | | | -1.000433941 | ⊕⊕○○ Low | -1.000433941 | ⊕⊕○○ Low |
| 93 | Dapagliflozin_low_dosage:Inject_semaglutide_low_dosage | 0 | | | 0.223371253 | ⊕⊕○○ Low | 0.223371253 | ⊕⊕○○ Low |
| 94 | Dapagliflozin_low_dosage:Inject_semaglutide_medium_dosage | 0 | | | -0.685417397 | ⊕⊕○○ Low | -0.685417397 | ⊕⊕○○ Low |
| 95 | Dapagliflozin_low_dosage:Liraglutide | 0 | | | -0.63882057 | ⊕⊕○○ Low | -0.63882057 | ⊕⊕○○ Low |
| 96 | Dapagliflozin_low_dosage:Lixisenatide | 0 | | | -0.440784392 | ⊕⊕○○ Low | -0.440784392 | ⊕⊕○○ Low |
| 97 | Dapagliflozin_low_dosage:Oral_semaglutide | 0 | | | -0.01806956 | ⊕⊕○○ Low | -0.01806956 | ⊕⊕○○ Low |
| 98 | Dapagliflozin_low_dosage:Placebo_or_Control | 2 | -1.117310385 | ⊕⊕⊕⊕ High | | | -1.117310385 | ⊕⊕⊕○ Medium |
| 99 | Dapagliflozin_low_dosage:Sotagliflozin | 0 | | | -1.116364579 | ⊕⊕○○ Low | -1.116364579 | ⊕⊕○○ Low |
| 100 | Dapagliflozin_low_dosage:Tirzepatide | 0 | | | 0.665666593 | ⊕⊕○○ Low | 0.665666593 | ⊕⊕○○ Low |
| 101 | Dapagliflozin_medium_dosage:Dulaglutide | 0 | | | -1.653269979 | ⊕⊕○○ Low | -1.653269979 | ⊕⊕○○ Low |
| 102 | Dapagliflozin_medium_dosage:Efpeglenatide_high_dosage | 0 | | | -1.139222779 | ⊕⊕○○ Low | -1.139222779 | ⊕⊕○○ Low |
| 103 | Dapagliflozin_medium_dosage:Efpeglenatide_medium_dosage | 0 | | | -2.237099233 | ⊕⊕○○ Low | -2.237099233 | ⊕⊕○○ Low |
| 104 | Dapagliflozin_medium_dosage:Empagliflozin_high_dosage | 0 | | | -1.146946435 | ⊕⊕○○ Low | -1.146946435 | ⊕⊕○○ Low |
| 105 | Dapagliflozin_medium_dosage:Empagliflozin_low_dosage | 0 | | | -2.107682808 | ⊕⊕○○ Low | -2.107682808 | ⊕⊕○○ Low |
| 106 | Dapagliflozin_medium_dosage:Ertugliflozin_high_dosage | 0 | | | -2.240045828 | ⊕⊕○○ Low | -2.240045828 | ⊕⊕○○ Low |
| 107 | Dapagliflozin_medium_dosage:Ertugliflozin_low_dosage | 0 | | | -1.83458072 | ⊕⊕○○ Low | -1.83458072 | ⊕⊕○○ Low |

| | | | | | | | | |
|---|---|---|---|---|---|---|---|---|
| 108 | Dapagliflozin_medium_dosage:Exenatide | 0 | | | -1.464105825 | ⊕⊕○○ Low | -1.464105825 | ⊕⊕○○ Low |
| 109 | Dapagliflozin_medium_dosage:Inject_semaglutide_high_dosage | 0 | | | -1.024557096 | ⊕⊕○○ Low | -1.024557096 | ⊕⊕○○ Low |
| 110 | Dapagliflozin_medium_dosage:Inject_semaglutide_low_dosage | 0 | | | 0.199248099 | ⊕⊕○○ Low | 0.199248099 | ⊕⊕○○ Low |
| 111 | Dapagliflozin_medium_dosage:Inject_semaglutide_medium_dosage | 0 | | | -0.709540552 | ⊕⊕○○ Low | -0.709540552 | ⊕⊕○○ Low |
| 112 | Dapagliflozin_medium_dosage:Liraglutide | 0 | | | -0.662943725 | ⊕⊕○○ Low | -0.662943725 | ⊕⊕○○ Low |
| 113 | Dapagliflozin_medium_dosage:Lixisenatide | 0 | | | -0.464907547 | ⊕⊕○○ Low | -0.464907547 | ⊕⊕○○ Low |
| 114 | Dapagliflozin_medium_dosage:Oral_semaglutide | 0 | | | -0.042192715 | ⊕⊕○○ Low | -0.042192715 | ⊕⊕○○ Low |
| 115 | Dapagliflozin_medium_dosage:Placebo_or_Control | 2 | -1.141433539 | ⊕⊕⊕⊕ High | | | -1.141433539 | ⊕⊕⊕○ Medium |
| 116 | Dapagliflozin_medium_dosage:Sotagliflozin | 0 | | | -1.140487734 | ⊕⊕○○ Low | -1.140487734 | ⊕⊕○○ Low |
| 117 | Dapagliflozin_medium_dosage:Tirzepatide | 0 | | | 0.641543438 | ⊕⊕○○ Low | 0.641543438 | ⊕⊕○○ Low |
| 118 | Dulaglutide:Efpeglenatide_high_dosage | 0 | | | 0.5140472 | ⊕⊕○○ Low | 0.5140472 | ⊕⊕○○ Low |
| 119 | Dulaglutide:Efpeglenatide_medium_dosage | 0 | | | -0.583829254 | ⊕⊕○○ Low | -0.583829254 | ⊕⊕○○ Low |
| 120 | Dulaglutide:Empagliflozin_high_dosage | 0 | | | 0.506323544 | ⊕⊕○○ Low | 0.506323544 | ⊕⊕○○ Low |
| 121 | Dulaglutide:Empagliflozin_low_dosage | 0 | | | -0.45441283 | ⊕⊕○○ Low | -0.45441283 | ⊕⊕○○ Low |
| 122 | Dulaglutide:Ertugliflozin_high_dosage | 0 | | | -0.586775849 | ⊕⊕○○ Low | -0.586775849 | ⊕⊕○○ Low |
| 123 | Dulaglutide:Ertugliflozin_low_dosage | 0 | | | -0.181310741 | ⊕⊕○○ Low | -0.181310741 | ⊕⊕○○ Low |
| 124 | Dulaglutide:Exenatide | 0 | | | 0.189164154 | ⊕⊕○○ Low | 0.189164154 | ⊕⊕○○ Low |
| 125 | Dulaglutide:Inject_semaglutide_high_dosage | 0 | | | 0.628712883 | ⊕⊕○○ Low | 0.628712883 | ⊕⊕○○ Low |
| 126 | Dulaglutide:Inject_semaglutide_low_dosage | 0 | | | 1.852518078 | ⊕⊕○○ Low | 1.852518078 | ⊕⊕○○ Low |
| 127 | Dulaglutide:Inject_semaglutide_medium_dosage | 0 | | | 0.943729427 | ⊕⊕○○ Low | 0.943729427 | ⊕⊕○○ Low |
| 128 | Dulaglutide:Liraglutide | 0 | | | 0.990326254 | ⊕⊕○○ Low | 0.990326254 | ⊕⊕○○ Low |
| 129 | Dulaglutide:Lixisenatide | 0 | | | 1.188362432 | ⊕⊕○○ Low | 1.188362432 | ⊕⊕○○ Low |

| | | | | | | | | |
|---|---|---|---|---|---|---|---|---|
| 130 | Dulaglutide:Oral_semaglutide | 0 | | | 1.611077264 | ⊕⊕○○ Low | 1.611077264 | ⊕⊕○○ Low |
| 131 | Dulaglutide:Placebo_or_Control | 1 | 0.51183644 | ⊕⊕⊕⊕ High | | | 0.51183644 | ⊕⊕⊕○ Medium |
| 132 | Dulaglutide:Sotagliflozin | 0 | | | 0.512782245 | ⊕⊕○○ Low | 0.512782245 | ⊕⊕○○ Low |
| 133 | Dulaglutide:Tirzepatide | 0 | | | 2.294813417 | ⊕⊕○○ Low | 2.294813417 | ⊕⊕○○ Low |
| 134 | Efpeglenatide_high_dosage:Efpeglenatide_medium_dosage | 1 | -1.097876453 | ⊕⊕⊕⊕ High | | | -1.097876453 | ⊕⊕⊕○ Medium |
| 135 | Efpeglenatide_high_dosage:Empagliflozin_high_dosage | 0 | | | -0.007723656 | ⊕⊕○○ Low | -0.007723656 | ⊕⊕○○ Low |
| 136 | Efpeglenatide_high_dosage:Empagliflozin_low_dosage | 0 | | | -0.968460029 | ⊕⊕○○ Low | -0.968460029 | ⊕⊕○○ Low |
| 137 | Efpeglenatide_high_dosage:Ertugliflozin_high_dosage | 0 | | | -1.100823049 | ⊕⊕○○ Low | -1.100823049 | ⊕⊕○○ Low |
| 138 | Efpeglenatide_high_dosage:Ertugliflozin_low_dosage | 0 | | | -0.69535794 | ⊕⊕○○ Low | -0.69535794 | ⊕⊕○○ Low |
| 139 | Efpeglenatide_high_dosage:Exenatide | 0 | | | -0.324883045 | ⊕⊕○○ Low | -0.324883045 | ⊕⊕○○ Low |
| 140 | Efpeglenatide_high_dosage:Inject_semaglutide_high_dosage | 0 | | | 0.114665683 | ⊕⊕○○ Low | 0.114665683 | ⊕⊕○○ Low |
| 141 | Efpeglenatide_high_dosage:Inject_semaglutide_low_dosage | 0 | | | 1.338470878 | ⊕⊕○○ Low | 1.338470878 | ⊕⊕○○ Low |
| 142 | Efpeglenatide_high_dosage:Inject_semaglutide_medium_dosage | 0 | | | 0.429682228 | ⊕⊕○○ Low | 0.429682228 | ⊕⊕○○ Low |
| 143 | Efpeglenatide_high_dosage:Liraglutide | 0 | | | 0.476279055 | ⊕⊕○○ Low | 0.476279055 | ⊕⊕○○ Low |
| 144 | Efpeglenatide_high_dosage:Lixisenatide | 0 | | | 0.674315232 | ⊕⊕○○ Low | 0.674315232 | ⊕⊕○○ Low |
| 145 | Efpeglenatide_high_dosage:Oral_semaglutide | 0 | | | 1.097030064 | ⊕⊕○○ Low | 1.097030064 | ⊕⊕○○ Low |
| 146 | Efpeglenatide_high_dosage:Placebo_or_Control | 0 | | | -0.00221076 | ⊕⊕○○ Low | -0.00221076 | ⊕⊕○○ Low |
| 147 | Efpeglenatide_high_dosage:Sotagliflozin | 0 | | | -0.001264955 | ⊕⊕○○ Low | -0.001264955 | ⊕⊕○○ Low |
| 148 | Efpeglenatide_high_dosage:Tirzepatide | 0 | | | 1.780766218 | ⊕⊕○○ Low | 1.780766218 | ⊕⊕○○ Low |
| 149 | Efpeglenatide_medium_dosage:Empagliflozin_high_dosage | 0 | | | 1.090152798 | ⊕⊕○○ Low | 1.090152798 | ⊕⊕○○ Low |
| 150 | Efpeglenatide_medium_dosage:Empagliflozin_low_dosage | 0 | | | 0.129416424 | ⊕⊕○○ Low | 0.129416424 | ⊕⊕○○ Low |
| 151 | Efpeglenatide_medium_dosage:Ertugliflozin_high_dosage | 0 | | | -0.002946595 | ⊕⊕○○ Low | -0.002946595 | ⊕⊕○○ Low |

| | | | | | | | | |
|---|---|---|---|---|---|---|---|---|
| 152 | Efpeglenatide_medium_dosage:Ertugliflozin_low_dosage | 0 | | | 0.402518513 | ⊕⊕○○ Low | 0.402518513 | ⊕⊕○○ Low |
| 153 | Efpeglenatide_medium_dosage:Exenatide | 0 | | | 0.772993408 | ⊕⊕○○ Low | 0.772993408 | ⊕⊕○○ Low |
| 154 | Efpeglenatide_medium_dosage:Inject_semaglutide_high_dosage | 0 | | | 1.212542137 | ⊕⊕○○ Low | 1.212542137 | ⊕⊕○○ Low |
| 155 | Efpeglenatide_medium_dosage:Inject_semaglutide_low_dosage | 0 | | | 2.436347331 | ⊕⊕○○ Low | 2.436347331 | ⊕⊕○○ Low |
| 156 | Efpeglenatide_medium_dosage:Inject_semaglutide_medium_dosage | 0 | | | 1.527558681 | ⊕⊕○○ Low | 1.527558681 | ⊕⊕○○ Low |
| 157 | Efpeglenatide_medium_dosage:Liraglutide | 0 | | | 1.574155508 | ⊕⊕○○ Low | 1.574155508 | ⊕⊕○○ Low |
| 158 | Efpeglenatide_medium_dosage:Lixisenatide | 0 | | | 1.772191686 | ⊕⊕○○ Low | 1.772191686 | ⊕⊕○○ Low |
| 159 | Efpeglenatide_medium_dosage:Oral_semaglutide | 0 | | | 2.194906518 | ⊕⊕○○ Low | 2.194906518 | ⊕⊕○○ Low |
| 160 | Efpeglenatide_medium_dosage:Placebo_or_Control | 1 | 1.095665694 | ⊕⊕⊕⊕ High | | | 1.095665694 | ⊕⊕⊕○ Medium |
| 161 | Efpeglenatide_medium_dosage:Sotagliflozin | 0 | | | 1.096611499 | ⊕⊕○○ Low | 1.096611499 | ⊕⊕○○ Low |
| 162 | Efpeglenatide_medium_dosage:Tirzepatide | 0 | | | 2.878642671 | ⊕⊕○○ Low | 2.878642671 | ⊕⊕○○ Low |
| 163 | Empagliflozin_high_dosage:Empagliflozin_low_dosage | 3 | -0.809426225 | ⊕⊕⊕⊕ High | -1.277464162 | ⊕⊕⊕⊕ High | -0.960736373 | ⊕⊕⊕⊕ High |
| 164 | Empagliflozin_high_dosage:Ertugliflozin_high_dosage | 0 | | | -1.093099393 | ⊕⊕○○ Low | -1.093099393 | ⊕⊕○○ Low |
| 165 | Empagliflozin_high_dosage:Ertugliflozin_low_dosage | 0 | | | -0.687634285 | ⊕⊕○○ Low | -0.687634285 | ⊕⊕○○ Low |
| 166 | Empagliflozin_high_dosage:Exenatide | 0 | | | -0.31715939 | ⊕⊕○○ Low | -0.31715939 | ⊕⊕○○ Low |
| 167 | Empagliflozin_high_dosage:Inject_semaglutide_high_dosage | 0 | | | 0.122389339 | ⊕⊕○○ Low | 0.122389339 | ⊕⊕○○ Low |
| 168 | Empagliflozin_high_dosage:Inject_semaglutide_low_dosage | 0 | | | 1.346194534 | ⊕⊕○○ Low | 1.346194534 | ⊕⊕○○ Low |
| 169 | Empagliflozin_high_dosage:Inject_semaglutide_medium_dosage | 0 | | | 0.437405883 | ⊕⊕○○ Low | 0.437405883 | ⊕⊕○○ Low |
| 170 | Empagliflozin_high_dosage:Liraglutide | 0 | | | 0.48400271 | ⊕⊕○○ Low | 0.48400271 | ⊕⊕○○ Low |
| 171 | Empagliflozin_high_dosage:Lixisenatide | 0 | | | 0.682038888 | ⊕⊕○○ Low | 0.682038888 | ⊕⊕○○ Low |
| 172 | Empagliflozin_high_dosage:Oral_semaglutide | 0 | | | 1.10475372 | ⊕⊕○○ Low | 1.10475372 | ⊕⊕○○ Low |
| 173 | Empagliflozin_high_dosage:Placebo_or_Control | 2 | -0.238121707 | ⊕⊕⊕⊕ High | 0.22991623 | ⊕⊕⊕○ Medium | 0.005512896 | ⊕⊕⊕⊕ High |

| | | | | | | | | |
|---|---|---|---|---|---|---|---|---|
| 174 | Empagliflozin_high_dosage:Sotagliflozin | 0 | | | 0.006458701 | ⊕⊕○○ Low | 0.006458701 | ⊕⊕○○ Low |
| 175 | Empagliflozin_high_dosage:Tirzepatide | 0 | | | 1.788489873 | ⊕⊕○○ Low | 1.788489873 | ⊕⊕○○ Low |
| 176 | Empagliflozin_low_dosage:Ertugliflozin_high_dosage | 0 | | | -0.13236302 | ⊕⊕○○ Low | -0.13236302 | ⊕⊕○○ Low |
| 177 | Empagliflozin_low_dosage:Ertugliflozin_low_dosage | 0 | | | 0.273102089 | ⊕⊕○○ Low | 0.273102089 | ⊕⊕○○ Low |
| 178 | Empagliflozin_low_dosage:Exenatide | 0 | | | 0.643576984 | ⊕⊕○○ Low | 0.643576984 | ⊕⊕○○ Low |
| 179 | Empagliflozin_low_dosage:Inject_semaglutide_high_dosage | 0 | | | 1.083125712 | ⊕⊕○○ Low | 1.083125712 | ⊕⊕○○ Low |
| 180 | Empagliflozin_low_dosage:Inject_semaglutide_low_dosage | 0 | | | 2.306930907 | ⊕⊕○○ Low | 2.306930907 | ⊕⊕○○ Low |
| 181 | Empagliflozin_low_dosage:Inject_semaglutide_medium_dosage | 0 | | | 1.398142257 | ⊕⊕○○ Low | 1.398142257 | ⊕⊕○○ Low |
| 182 | Empagliflozin_low_dosage:Liraglutide | 0 | | | 1.444739084 | ⊕⊕○○ Low | 1.444739084 | ⊕⊕○○ Low |
| 183 | Empagliflozin_low_dosage:Lixisenatide | 0 | | | 1.642775261 | ⊕⊕○○ Low | 1.642775261 | ⊕⊕○○ Low |
| 184 | Empagliflozin_low_dosage:Oral_semaglutide | 0 | | | 2.065490093 | ⊕⊕○○ Low | 2.065490093 | ⊕⊕○○ Low |
| 185 | Empagliflozin_low_dosage:Placebo_or_Control | 5 | 1.039342455 | ⊕⊕⊕⊕ High | 0.571304518 | ⊕⊕⊕⊕ High | 0.966249269 | ⊕⊕⊕⊕ High |
| 186 | Empagliflozin_low_dosage:Sotagliflozin | 0 | | | 0.967195075 | ⊕⊕○○ Low | 0.967195075 | ⊕⊕○○ Low |
| 187 | Empagliflozin_low_dosage:Tirzepatide | 0 | | | 2.749226247 | ⊕⊕○○ Low | 2.749226247 | ⊕⊕○○ Low |
| 188 | Ertugliflozin_high_dosage:Ertugliflozin_low_dosage | 1 | 0.405465108 | ⊕⊕⊕⊕ High | | | 0.405465108 | ⊕⊕⊕○ Medium |
| 189 | Ertugliflozin_high_dosage:Exenatide | 0 | | | 0.775940003 | ⊕⊕○○ Low | 0.775940003 | ⊕⊕○○ Low |
| 190 | Ertugliflozin_high_dosage:Inject_semaglutide_high_dosage | 0 | | | 1.215488732 | ⊕⊕○○ Low | 1.215488732 | ⊕⊕○○ Low |
| 191 | Ertugliflozin_high_dosage:Inject_semaglutide_low_dosage | 0 | | | 2.439293927 | ⊕⊕○○ Low | 2.439293927 | ⊕⊕○○ Low |
| 192 | Ertugliflozin_high_dosage:Inject_semaglutide_medium_dosage | 0 | | | 1.530505276 | ⊕⊕○○ Low | 1.530505276 | ⊕⊕○○ Low |
| 193 | Ertugliflozin_high_dosage:Liraglutide | 0 | | | 1.577102103 | ⊕⊕○○ Low | 1.577102103 | ⊕⊕○○ Low |
| 194 | Ertugliflozin_high_dosage:Lixisenatide | 0 | | | 1.775138281 | ⊕⊕○○ Low | 1.775138281 | ⊕⊕○○ Low |
| 195 | Ertugliflozin_high_dosage:Oral_semaglutide | 0 | | | 2.197853113 | ⊕⊕○○ Low | 2.197853113 | ⊕⊕○○ Low |

| | | | | | | | | |
|---|---|---|---|---|---|---|---|---|
| 196 | Ertugliflozin_high_dosage:Placebo_or_Control | 1 | 1.098612289 | ⊕⊕⊕⊕ High | | | 1.098612289 | ⊕⊕⊕○ Medium |
| 197 | Ertugliflozin_high_dosage:Sotagliflozin | 0 | | | 1.099558094 | ⊕⊕○○ Low | 1.099558094 | ⊕⊕○○ Low |
| 198 | Ertugliflozin_high_dosage:Tirzepatide | 0 | | | 2.881589266 | ⊕⊕○○ Low | 2.881589266 | ⊕⊕○○ Low |
| 199 | Ertugliflozin_low_dosage:Exenatide | 0 | | | 0.370474895 | ⊕⊕○○ Low | 0.370474895 | ⊕⊕○○ Low |
| 200 | Ertugliflozin_low_dosage:Inject_semaglutide_high_dosage | 0 | | | 0.810023624 | ⊕⊕○○ Low | 0.810023624 | ⊕⊕○○ Low |
| 201 | Ertugliflozin_low_dosage:Inject_semaglutide_low_dosage | 0 | | | 2.033828818 | ⊕⊕○○ Low | 2.033828818 | ⊕⊕○○ Low |
| 202 | Ertugliflozin_low_dosage:Inject_semaglutide_medium_dosage | 0 | | | 1.125040168 | ⊕⊕○○ Low | 1.125040168 | ⊕⊕○○ Low |
| 203 | Ertugliflozin_low_dosage:Liraglutide | 0 | | | 1.171636995 | ⊕⊕○○ Low | 1.171636995 | ⊕⊕○○ Low |
| 204 | Ertugliflozin_low_dosage:Lixisenatide | 0 | | | 1.369673173 | ⊕⊕○○ Low | 1.369673173 | ⊕⊕○○ Low |
| 205 | Ertugliflozin_low_dosage:Oral_semaglutide | 0 | | | 1.792388005 | ⊕⊕○○ Low | 1.792388005 | ⊕⊕○○ Low |
| 206 | Ertugliflozin_low_dosage:Placebo_or_Control | 1 | 0.693147181 | ⊕⊕⊕⊕ High | | | 0.693147181 | ⊕⊕⊕○ Medium |
| 207 | Ertugliflozin_low_dosage:Sotagliflozin | 0 | | | 0.694092986 | ⊕⊕○○ Low | 0.694092986 | ⊕⊕○○ Low |
| 208 | Ertugliflozin_low_dosage:Tirzepatide | 0 | | | 2.476124158 | ⊕⊕○○ Low | 2.476124158 | ⊕⊕○○ Low |
| 209 | Exenatide:Inject_semaglutide_high_dosage | 0 | | | 0.439548729 | ⊕⊕○○ Low | 0.439548729 | ⊕⊕○○ Low |
| 210 | Exenatide:Inject_semaglutide_low_dosage | 0 | | | 1.663353923 | ⊕⊕○○ Low | 1.663353923 | ⊕⊕○○ Low |
| 211 | Exenatide:Inject_semaglutide_medium_dosage | 0 | | | 0.754565273 | ⊕⊕○○ Low | 0.754565273 | ⊕⊕○○ Low |
| 212 | Exenatide:Liraglutide | 0 | | | 0.8011621 | ⊕⊕○○ Low | 0.8011621 | ⊕⊕○○ Low |
| 213 | Exenatide:Lixisenatide | 0 | | | 0.999198278 | ⊕⊕○○ Low | 0.999198278 | ⊕⊕○○ Low |
| 214 | Exenatide:Oral_semaglutide | 0 | | | 1.42191311 | ⊕⊕○○ Low | 1.42191311 | ⊕⊕○○ Low |
| 215 | Exenatide:Placebo_or_Control | 1 | 0.322672285 | ⊕⊕⊕⊕ High | | | 0.322672285 | ⊕⊕⊕○ Medium |
| 216 | Exenatide:Sotagliflozin | 0 | | | 0.323618091 | ⊕⊕○○ Low | 0.323618091 | ⊕⊕○○ Low |
| 217 | Exenatide:Tirzepatide | 0 | | | 2.105649263 | ⊕⊕○○ Low | 2.105649263 | ⊕⊕○○ Low |

| | | | | | | | | |
|---|---|---|---|---|---|---|---|---|
| 218 | Inject_semaglutide_high_dosage:Inject_semaglutide_low_dosage | 0 | | | 1.223805195 | ⊕⊕○○ Low | 1.223805195 | ⊕⊕○○ Low |
| 219 | Inject_semaglutide_high_dosage:Inject_semaglutide_medium_dosage | 1 | 1.098612289 | ⊕⊕⊕⊕ High | -0.038596271 | ⊕⊕⊕○ Medium | 0.315016544 | ⊕⊕⊕⊕ High |
| 220 | Inject_semaglutide_high_dosage:Liraglutide | 0 | | | 0.361613371 | ⊕⊕○○ Low | 0.361613371 | ⊕⊕○○ Low |
| 221 | Inject_semaglutide_high_dosage:Lixisenatide | 0 | | | 0.559649549 | ⊕⊕○○ Low | 0.559649549 | ⊕⊕○○ Low |
| 222 | Inject_semaglutide_high_dosage:Oral_semaglutide | 0 | | | 0.982364381 | ⊕⊕○○ Low | 0.982364381 | ⊕⊕○○ Low |
| 223 | Inject_semaglutide_high_dosage:Placebo_or_Control | 3 | -0.190618967 | ⊕⊕⊕⊕ High | 0.946589593 | ⊕⊕⊕○ Medium | -0.116876443 | ⊕⊕⊕⊕ High |
| 224 | Inject_semaglutide_high_dosage:Sotagliflozin | 0 | | | -0.115930638 | ⊕⊕○○ Low | -0.115930638 | ⊕⊕○○ Low |
| 225 | Inject_semaglutide_high_dosage:Tirzepatide | 0 | | | 1.666100534 | ⊕⊕○○ Low | 1.666100534 | ⊕⊕○○ Low |
| 226 | Inject_semaglutide_low_dosage:Inject_semaglutide_medium_dosage | 1 | -1.104680268 | ⊕⊕⊕⊕ High | 3.439996077 | ⊕⊕⊕○ Medium | -0.90878865 | ⊕⊕⊕⊕ High |
| 227 | Inject_semaglutide_low_dosage:Liraglutide | 0 | | | -0.862191823 | ⊕⊕○○ Low | -0.862191823 | ⊕⊕○○ Low |
| 228 | Inject_semaglutide_low_dosage:Lixisenatide | 0 | | | -0.664155646 | ⊕⊕○○ Low | -0.664155646 | ⊕⊕○○ Low |
| 229 | Inject_semaglutide_low_dosage:Oral_semaglutide | 0 | | | -0.241440814 | ⊕⊕○○ Low | -0.241440814 | ⊕⊕○○ Low |
| 230 | Inject_semaglutide_low_dosage:Placebo_or_Control | 1 | -1.256702964 | ⊕⊕⊕⊕ High | -10.34229117 | ⊕⊕⊕⊕ High | -1.340681638 | ⊕⊕⊕⊕ High |
| 231 | Inject_semaglutide_low_dosage:Sotagliflozin | 0 | | | -1.339735832 | ⊕⊕○○ Low | -1.339735832 | ⊕⊕○○ Low |
| 232 | Inject_semaglutide_low_dosage:Tirzepatide | 0 | | | 0.44229534 | ⊕⊕○○ Low | 0.44229534 | ⊕⊕○○ Low |
| 233 | Inject_semaglutide_medium_dosage:Liraglutide | 0 | | | 0.046596827 | ⊕⊕○○ Low | 0.046596827 | ⊕⊕○○ Low |
| 234 | Inject_semaglutide_medium_dosage:Lixisenatide | 0 | | | 0.244633005 | ⊕⊕○○ Low | 0.244633005 | ⊕⊕○○ Low |
| 235 | Inject_semaglutide_medium_dosage:Oral_semaglutide | 0 | | | 0.667347837 | ⊕⊕○○ Low | 0.667347837 | ⊕⊕○○ Low |
| 236 | Inject_semaglutide_medium_dosage:Placebo_or_Control | 1 | -0.152022696 | ⊕⊕⊕⊕ High | -1.289231256 | ⊕⊕⊕⊕ High | -0.431892988 | ⊕⊕⊕⊕ High |
| 237 | Inject_semaglutide_medium_dosage:Sotagliflozin | 0 | | | -0.430947182 | ⊕⊕○○ Low | -0.430947182 | ⊕⊕○○ Low |
| 238 | Inject_semaglutide_medium_dosage:Tirzepatide | 0 | | | 1.35108399 | ⊕⊕○○ Low | 1.35108399 | ⊕⊕○○ Low |
| 239 | Liraglutide:Lixisenatide | 1 | 1.103562794 | ⊕⊕⊕⊕ High | -0.585128252 | ⊕⊕⊕○ Medium | 0.198036178 | ⊕⊕⊕⊕ High |

| | | | | | | | | |
|---|---|---|---|---|---|---|---|---|
| 240 | Liraglutide:Oral_semaglutide | 0 | | | 0.62075101 | ⊕⊕○○ Low | 0.62075101 | ⊕⊕○○ Low |
| 241 | Liraglutide:Placebo_or_Control | 2 | -0.584798273 | ⊕⊕⊕⊕ High | 1.103892772 | ⊕⊕⊕○ Medium | -0.478489814 | ⊕⊕⊕⊕ High |
| 242 | Liraglutide:Sotagliflozin | 0 | | | -0.477544009 | ⊕⊕○○ Low | -0.477544009 | ⊕⊕○○ Low |
| 243 | Liraglutide:Tirzepatide | 0 | | | 1.304487163 | ⊕⊕○○ Low | 1.304487163 | ⊕⊕○○ Low |
| 244 | Lixisenatide:Oral_semaglutide | 0 | | | 0.422714832 | ⊕⊕○○ Low | 0.422714832 | ⊕⊕○○ Low |
| 245 | Lixisenatide:Placebo_or_Control | 1 | 0.000329979 | ⊕⊕⊕⊕ High | -1.688361067 | ⊕⊕⊕○ Medium | -0.676525992 | ⊕⊕⊕⊕ High |
| 246 | Lixisenatide:Sotagliflozin | 0 | | | -0.675580187 | ⊕⊕○○ Low | -0.675580187 | ⊕⊕○○ Low |
| 247 | Lixisenatide:Tirzepatide | 0 | | | 1.106450985 | ⊕⊕○○ Low | 1.106450985 | ⊕⊕○○ Low |
| 248 | Oral_semaglutide:Placebo_or_Control | 1 | -1.099240824 | ⊕⊕⊕⊕ High | | | -1.099240824 | ⊕⊕⊕○ Medium |
| 249 | Oral_semaglutide:Sotagliflozin | 0 | | | -1.098295019 | ⊕⊕○○ Low | -1.098295019 | ⊕⊕○○ Low |
| 250 | Oral_semaglutide:Tirzepatide | 0 | | | 0.683736153 | ⊕⊕○○ Low | 0.683736153 | ⊕⊕○○ Low |
| 251 | Sotagliflozin:Placebo_or_Control | 1 | -0.000945805 | ⊕⊕⊕⊕ High | | | -0.000945805 | ⊕⊕⊕○ Medium |
| 252 | Tirzepatide:Placebo_or_Control | 1 | -1.782976978 | ⊕⊕⊕⊕ High | | | -1.782976978 | |
| 253 | Sotagliflozin:Tirzepatide | 0 | | | 1.782031172 | ⊕⊕○○ Low | 1.782031172 | ⊕⊕○○ Low |

## Table S8D: GRADE of primary outcome: subgroup of myeloma

| | Comparison | No.Studies | Direct | | Indirect | | NMA | |
|---|---|---|---|---|---|---|---|---|
| | | | Estimate | Rate | Estimate | Rate | Estimate | Rate |
| 1 | Albiglutide:Canagliflozin_high_dosage | 0 | | | 2.197701357 | ⊕⊕○○ Low | 2.197701357 | ⊕⊕○○ Low |
| 2 | Albiglutide:Canagliflozin_low_dosage | 0 | | | 2.198831956 | ⊕⊕○○ Low | 2.198831956 | ⊕⊕○○ Low |
| 3 | Albiglutide:Dapagliflozin_high_dosage | 0 | | | 1.758089416 | ⊕⊕○○ Low | 1.758089416 | ⊕⊕○○ Low |
| 4 | Albiglutide:Dulaglutide | 0 | | | 0.143916078 | ⊕⊕○○ Low | 0.143916078 | ⊕⊕○○ Low |
| 5 | Albiglutide:Empagliflozin_high_dosage | 0 | | | 1.407795517 | ⊕⊕○○ Low | 1.407795517 | ⊕⊕○○ Low |
| 6 | Albiglutide:Empagliflozin_low_dosage | 0 | | | 0.868338851 | ⊕⊕○○ Low | 0.868338851 | ⊕⊕○○ Low |
| 7 | Albiglutide:Ertugliflozin_high_dosage | 0 | | | 1.08789439 | ⊕⊕○○ Low | 1.08789439 | ⊕⊕○○ Low |
| 8 | Albiglutide:Ertugliflozin_low_dosage | 0 | | | 2.199402723 | ⊕⊕○○ Low | 2.199402723 | ⊕⊕○○ Low |
| 9 | Albiglutide:Exenatide | 0 | | | 1.027880943 | ⊕⊕○○ Low | 1.027880943 | ⊕⊕○○ Low |
| 10 | Albiglutide:Inject_semaglutide_high_dosage | 0 | | | 1.281062846 | ⊕⊕○○ Low | 1.281062846 | ⊕⊕○○ Low |
| 11 | Albiglutide:Inject_semaglutide_low_dosage | 0 | | | 1.506591388 | ⊕⊕○○ Low | 1.506591388 | ⊕⊕○○ Low |
| 12 | Albiglutide:Inject_semaglutide_medium_dosage | 0 | | | 1.501739953 | ⊕⊕○○ Low | 1.501739953 | ⊕⊕○○ Low |
| 13 | Albiglutide:Liraglutide | 0 | | | 1.697756666 | ⊕⊕○○ Low | 1.697756666 | ⊕⊕○○ Low |
| 14 | Albiglutide:Lixisenatide | 0 | | | -0.000871784 | ⊕⊕○○ Low | -0.000871784 | ⊕⊕○○ Low |
| 15 | Albiglutide:Oral_semaglutide | 0 | | | 0.547184052 | ⊕⊕○○ Low | 0.547184052 | ⊕⊕○○ Low |
| 16 | Albiglutide:Placebo_or_Control | 1 | 1.098400245 | ⊕⊕⊕⊕ High | | | 1.098400245 | ⊕⊕⊕○ Medium |
| 17 | Albiglutide:Sotagliflozin | 0 | | | 1.031522199 | ⊕⊕○○ Low | 1.031522199 | ⊕⊕○○ Low |
| 18 | Albiglutide:Tirzepatide | 0 | | | 3.279411078 | ⊕⊕○○ Low | 3.279411078 | ⊕⊕○○ Low |
| 19 | Canagliflozin_high_dosage:Canagliflozin_low_dosage | 0 | | | 0.001130599 | ⊕⊕○○ Low | 0.001130599 | ⊕⊕○○ Low |

| | | | | | | | | |
|---|---|---|---|---|---|---|---|---|
| 20 | Canagliflozin_high_dosage:Dapagliflozin_high_dosage | 0 | | | -0.439611942 | ⊕⊕○○ Low | -0.439611942 | ⊕⊕○○ Low |
| 21 | Canagliflozin_high_dosage:Dulaglutide | 0 | | | -2.053785279 | ⊕⊕○○ Low | -2.053785279 | ⊕⊕○○ Low |
| 22 | Canagliflozin_high_dosage:Empagliflozin_high_dosage | 0 | | | -0.78990584 | ⊕⊕○○ Low | -0.78990584 | ⊕⊕○○ Low |
| 23 | Canagliflozin_high_dosage:Empagliflozin_low_dosage | 0 | | | -1.329362506 | ⊕⊕○○ Low | -1.329362506 | ⊕⊕○○ Low |
| 24 | Canagliflozin_high_dosage:Ertugliflozin_high_dosage | 0 | | | -1.109806967 | ⊕⊕○○ Low | -1.109806967 | ⊕⊕○○ Low |
| 25 | Canagliflozin_high_dosage:Ertugliflozin_low_dosage | 0 | | | 0.001701366 | ⊕⊕○○ Low | 0.001701366 | ⊕⊕○○ Low |
| 26 | Canagliflozin_high_dosage:Exenatide | 0 | | | -1.169820414 | ⊕⊕○○ Low | -1.169820414 | ⊕⊕○○ Low |
| 27 | Canagliflozin_high_dosage:Inject_semaglutide_high_dosage | 0 | | | -0.916638511 | ⊕⊕○○ Low | -0.916638511 | ⊕⊕○○ Low |
| 28 | Canagliflozin_high_dosage:Inject_semaglutide_low_dosage | 0 | | | -0.691109969 | ⊕⊕○○ Low | -0.691109969 | ⊕⊕○○ Low |
| 29 | Canagliflozin_high_dosage:Inject_semaglutide_medium_dosage | 0 | | | -0.695961404 | ⊕⊕○○ Low | -0.695961404 | ⊕⊕○○ Low |
| 30 | Canagliflozin_high_dosage:Liraglutide | 0 | | | -0.499944691 | ⊕⊕○○ Low | -0.499944691 | ⊕⊕○○ Low |
| 31 | Canagliflozin_high_dosage:Lixisenatide | 0 | | | -2.198573141 | ⊕⊕○○ Low | -2.198573141 | ⊕⊕○○ Low |
| 32 | Canagliflozin_high_dosage:Oral_semaglutide | 0 | | | -1.650517305 | ⊕⊕○○ Low | -1.650517305 | ⊕⊕○○ Low |
| 33 | Canagliflozin_high_dosage:Placebo_or_Control | 1 | -1.099301113 | ⊕⊕⊕⊕ High | | | -1.099301113 | ⊕⊕⊕○ Medium |
| 34 | Canagliflozin_high_dosage:Sotagliflozin | 0 | | | -1.166179158 | ⊕⊕○○ Low | -1.166179158 | ⊕⊕○○ Low |
| 35 | Canagliflozin_high_dosage:Tirzepatide | 0 | | | 1.08170972 | ⊕⊕○○ Low | 1.08170972 | ⊕⊕○○ Low |
| 36 | Canagliflozin_low_dosage:Dapagliflozin_high_dosage | 0 | | | -0.440742541 | ⊕⊕○○ Low | -0.440742541 | ⊕⊕○○ Low |
| 37 | Canagliflozin_low_dosage:Dulaglutide | 0 | | | -2.054915878 | ⊕⊕○○ Low | -2.054915878 | ⊕⊕○○ Low |
| 38 | Canagliflozin_low_dosage:Empagliflozin_high_dosage | 0 | | | -0.791036439 | ⊕⊕○○ Low | -0.791036439 | ⊕⊕○○ Low |
| 39 | Canagliflozin_low_dosage:Empagliflozin_low_dosage | 0 | | | -1.330493105 | ⊕⊕○○ Low | -1.330493105 | ⊕⊕○○ Low |
| 40 | Canagliflozin_low_dosage:Ertugliflozin_high_dosage | 0 | | | -1.110937566 | ⊕⊕○○ Low | -1.110937566 | ⊕⊕○○ Low |
| 41 | Canagliflozin_low_dosage:Ertugliflozin_low_dosage | 0 | | | 0.000570767 | ⊕⊕○○ Low | 0.000570767 | ⊕⊕○○ Low |

| | | | | | | | | |
|---|---|---|---|---|---|---|---|---|
| 42 | Canagliflozin_low_dosage:Exenatide | 0 | | | -1.170951013 | ⊕⊕○○ Low | -1.170951013 | ⊕⊕○○ Low |
| 43 | Canagliflozin_low_dosage:Inject_semaglutide_high_dosage | 0 | | | -0.91776911 | ⊕⊕○○ Low | -0.91776911 | ⊕⊕○○ Low |
| 44 | Canagliflozin_low_dosage:Inject_semaglutide_low_dosage | 0 | | | -0.692240568 | ⊕⊕○○ Low | -0.692240568 | ⊕⊕○○ Low |
| 45 | Canagliflozin_low_dosage:Inject_semaglutide_medium_dosage | 0 | | | -0.697092003 | ⊕⊕○○ Low | -0.697092003 | ⊕⊕○○ Low |
| 46 | Canagliflozin_low_dosage:Liraglutide | 0 | | | -0.50107529 | ⊕⊕○○ Low | -0.50107529 | ⊕⊕○○ Low |
| 47 | Canagliflozin_low_dosage:Lixisenatide | 0 | | | -2.19970374 | ⊕⊕○○ Low | -2.19970374 | ⊕⊕○○ Low |
| 48 | Canagliflozin_low_dosage:Oral_semaglutide | 0 | | | -1.651647904 | ⊕⊕○○ Low | -1.651647904 | ⊕⊕○○ Low |
| 49 | Canagliflozin_low_dosage:Placebo_or_Control | 1 | -1.100431712 | ⊕⊕⊕⊕ High | | | -1.100431712 | ⊕⊕⊕○ Medium |
| 50 | Canagliflozin_low_dosage:Sotagliflozin | 0 | | | -1.167309757 | ⊕⊕○○ Low | -1.167309757 | ⊕⊕○○ Low |
| 51 | Canagliflozin_low_dosage:Tirzepatide | 0 | | | 1.080579122 | ⊕⊕○○ Low | 1.080579122 | ⊕⊕○○ Low |
| 52 | Dapagliflozin_high_dosage:Dulaglutide | 0 | | | -1.614173337 | ⊕⊕○○ Low | -1.614173337 | ⊕⊕○○ Low |
| 53 | Dapagliflozin_high_dosage:Empagliflozin_high_dosage | 0 | | | -0.350293899 | ⊕⊕○○ Low | -0.350293899 | ⊕⊕○○ Low |
| 54 | Dapagliflozin_high_dosage:Empagliflozin_low_dosage | 0 | | | -0.889750564 | ⊕⊕○○ Low | -0.889750564 | ⊕⊕○○ Low |
| 55 | Dapagliflozin_high_dosage:Ertugliflozin_high_dosage | 0 | | | -0.670195026 | ⊕⊕○○ Low | -0.670195026 | ⊕⊕○○ Low |
| 56 | Dapagliflozin_high_dosage:Ertugliflozin_low_dosage | 0 | | | 0.441313307 | ⊕⊕○○ Low | 0.441313307 | ⊕⊕○○ Low |
| 57 | Dapagliflozin_high_dosage:Exenatide | 0 | | | -0.730208472 | ⊕⊕○○ Low | -0.730208472 | ⊕⊕○○ Low |
| 58 | Dapagliflozin_high_dosage:Inject_semaglutide_high_dosage | 0 | | | -0.477026569 | ⊕⊕○○ Low | -0.477026569 | ⊕⊕○○ Low |
| 59 | Dapagliflozin_high_dosage:Inject_semaglutide_low_dosage | 0 | | | -0.251498028 | ⊕⊕○○ Low | -0.251498028 | ⊕⊕○○ Low |
| 60 | Dapagliflozin_high_dosage:Inject_semaglutide_medium_dosage | 0 | | | -0.256349462 | ⊕⊕○○ Low | -0.256349462 | ⊕⊕○○ Low |
| 61 | Dapagliflozin_high_dosage:Liraglutide | 0 | | | -0.060332749 | ⊕⊕○○ Low | -0.060332749 | ⊕⊕○○ Low |
| 62 | Dapagliflozin_high_dosage:Lixisenatide | 0 | | | -1.758961199 | ⊕⊕○○ Low | -1.758961199 | ⊕⊕○○ Low |
| 63 | Dapagliflozin_high_dosage:Oral_semaglutide | 0 | | | -1.210905364 | ⊕⊕○○ Low | -1.210905364 | ⊕⊕○○ Low |

| | | | | | | | | |
|---|---|---|---|---|---|---|---|---|
| 64 | Dapagliflozin_high_dosage:Placebo_or_Control | 4 | -0.659689171 | ⊕⊕⊕⊕ High | | | -0.659689171 | ⊕⊕⊕○ Medium |
| 65 | Dapagliflozin_high_dosage:Sotagliflozin | 0 | | | -0.726567217 | ⊕⊕○○ Low | -0.726567217 | ⊕⊕○○ Low |
| 66 | Dapagliflozin_high_dosage:Tirzepatide | 0 | | | 1.521321662 | ⊕⊕○○ Low | 1.521321662 | ⊕⊕○○ Low |
| 67 | Dulaglutide:Empagliflozin_high_dosage | 0 | | | 1.263879439 | ⊕⊕○○ Low | 1.263879439 | ⊕⊕○○ Low |
| 68 | Dulaglutide:Empagliflozin_low_dosage | 0 | | | 0.724422773 | ⊕⊕○○ Low | 0.724422773 | ⊕⊕○○ Low |
| 69 | Dulaglutide:Ertugliflozin_high_dosage | 0 | | | 0.943978312 | ⊕⊕○○ Low | 0.943978312 | ⊕⊕○○ Low |
| 70 | Dulaglutide:Ertugliflozin_low_dosage | 0 | | | 2.055486645 | ⊕⊕○○ Low | 2.055486645 | ⊕⊕○○ Low |
| 71 | Dulaglutide:Exenatide | 0 | | | 0.883964865 | ⊕⊕○○ Low | 0.883964865 | ⊕⊕○○ Low |
| 72 | Dulaglutide:Inject_semaglutide_high_dosage | 0 | | | 1.137146768 | ⊕⊕○○ Low | 1.137146768 | ⊕⊕○○ Low |
| 73 | Dulaglutide:Inject_semaglutide_low_dosage | 0 | | | 1.36267531 | ⊕⊕○○ Low | 1.36267531 | ⊕⊕○○ Low |
| 74 | Dulaglutide:Inject_semaglutide_medium_dosage | 0 | | | 1.357823875 | ⊕⊕○○ Low | 1.357823875 | ⊕⊕○○ Low |
| 75 | Dulaglutide:Liraglutide | 0 | | | 1.553840588 | ⊕⊕○○ Low | 1.553840588 | ⊕⊕○○ Low |
| 76 | Dulaglutide:Lixisenatide | 0 | | | -0.144787862 | ⊕⊕○○ Low | -0.144787862 | ⊕⊕○○ Low |
| 77 | Dulaglutide:Oral_semaglutide | 0 | | | 0.403267974 | ⊕⊕○○ Low | 0.403267974 | ⊕⊕○○ Low |
| 78 | Dulaglutide:Placebo_or_Control | 2 | 0.954484166 | ⊕⊕⊕⊕ High | | | 0.954484166 | ⊕⊕⊕○ Medium |
| 79 | Dulaglutide:Sotagliflozin | 0 | | | 0.887606121 | ⊕⊕○○ Low | 0.887606121 | ⊕⊕○○ Low |
| 80 | Dulaglutide:Tirzepatide | 0 | | | 3.135494999 | ⊕⊕○○ Low | 3.135494999 | ⊕⊕○○ Low |
| 81 | Empagliflozin_high_dosage:Empagliflozin_low_dosage | 1 | -0.69229321 | ⊕⊕⊕⊕ High | 1.276829362 | ⊕⊕⊕○ Medium | -0.539456665 | ⊕⊕⊕⊕ High |
| 82 | Empagliflozin_high_dosage:Ertugliflozin_high_dosage | 0 | | | -0.319901127 | ⊕⊕○○ Low | -0.319901127 | ⊕⊕○○ Low |
| 83 | Empagliflozin_high_dosage:Ertugliflozin_low_dosage | 0 | | | 0.791607206 | ⊕⊕○○ Low | 0.791607206 | ⊕⊕○○ Low |
| 84 | Empagliflozin_high_dosage:Exenatide | 0 | | | -0.379914574 | ⊕⊕○○ Low | -0.379914574 | ⊕⊕○○ Low |
| 85 | Empagliflozin_high_dosage:Inject_semaglutide_high_dosage | 0 | | | -0.126732671 | ⊕⊕○○ Low | -0.126732671 | ⊕⊕○○ Low |

| | | | | | | | | |
|---|---|---|---|---|---|---|---|---|
| 86 | Empagliflozin_high_dosage:Inject_semaglutide_low_dosage | 0 | | | 0.098795871 | ⊕⊕○○ Low | 0.098795871 | ⊕⊕○○ Low |
| 87 | Empagliflozin_high_dosage:Inject_semaglutide_medium_dosage | 0 | | | 0.093944437 | ⊕⊕○○ Low | 0.093944437 | ⊕⊕○○ Low |
| 88 | Empagliflozin_high_dosage:Liraglutide | 0 | | | 0.28996115 | ⊕⊕○○ Low | 0.28996115 | ⊕⊕○○ Low |
| 89 | Empagliflozin_high_dosage:Lixisenatide | 0 | | | -1.4086673 | ⊕⊕○○ Low | -1.4086673 | ⊕⊕○○ Low |
| 90 | Empagliflozin_high_dosage:Oral_semaglutide | 0 | | | -0.860611465 | ⊕⊕○○ Low | -0.860611465 | ⊕⊕○○ Low |
| 91 | Empagliflozin_high_dosage:Placebo_or_Control | 1 | -0.00385192 | ⊕⊕⊕⊕ High | -1.316972211 | ⊕⊕⊕⊕ High | -0.309395272 | ⊕⊕⊕⊕ High |
| 92 | Empagliflozin_high_dosage:Sotagliflozin | 0 | | | -0.376273318 | ⊕⊕○○ Low | -0.376273318 | ⊕⊕○○ Low |
| 93 | Empagliflozin_high_dosage:Tirzepatide | 0 | | | 1.871615561 | ⊕⊕○○ Low | 1.871615561 | ⊕⊕○○ Low |
| 94 | Empagliflozin_low_dosage:Ertugliflozin_high_dosage | 0 | | | 0.219555539 | ⊕⊕○○ Low | 0.219555539 | ⊕⊕○○ Low |
| 95 | Empagliflozin_low_dosage:Ertugliflozin_low_dosage | 0 | | | 1.331063872 | ⊕⊕○○ Low | 1.331063872 | ⊕⊕○○ Low |
| 96 | Empagliflozin_low_dosage:Exenatide | 0 | | | 0.159542092 | ⊕⊕○○ Low | 0.159542092 | ⊕⊕○○ Low |
| 97 | Empagliflozin_low_dosage:Inject_semaglutide_high_dosage | 0 | | | 0.412723995 | ⊕⊕○○ Low | 0.412723995 | ⊕⊕○○ Low |
| 98 | Empagliflozin_low_dosage:Inject_semaglutide_low_dosage | 0 | | | 0.638252537 | ⊕⊕○○ Low | 0.638252537 | ⊕⊕○○ Low |
| 99 | Empagliflozin_low_dosage:Inject_semaglutide_medium_dosage | 0 | | | 0.633401102 | ⊕⊕○○ Low | 0.633401102 | ⊕⊕○○ Low |
| 100 | Empagliflozin_low_dosage:Liraglutide | 0 | | | 0.829417815 | ⊕⊕○○ Low | 0.829417815 | ⊕⊕○○ Low |
| 101 | Empagliflozin_low_dosage:Lixisenatide | 0 | | | -0.869210635 | ⊕⊕○○ Low | -0.869210635 | ⊕⊕○○ Low |
| 102 | Empagliflozin_low_dosage:Oral_semaglutide | 0 | | | -0.321154799 | ⊕⊕○○ Low | -0.321154799 | ⊕⊕○○ Low |
| 103 | Empagliflozin_low_dosage:Placebo_or_Control | 4 | 0.230061393 | ⊕⊕⊕⊕ High | | | 0.230061393 | ⊕⊕⊕○ Medium |
| 104 | Empagliflozin_low_dosage:Sotagliflozin | 0 | | | 0.163183347 | ⊕⊕○○ Low | 0.163183347 | ⊕⊕○○ Low |
| 105 | Empagliflozin_low_dosage:Tirzepatide | 0 | | | 2.411072226 | ⊕⊕○○ Low | 2.411072226 | ⊕⊕○○ Low |
| 106 | Ertugliflozin_high_dosage:Ertugliflozin_low_dosage | 1 | 1.113175653 | ⊕⊕⊕⊕ High | 1.109014356 | ⊕⊕⊕⊕ High | 1.111508333 | ⊕⊕⊕⊕ High |
| 107 | Ertugliflozin_high_dosage:Exenatide | 0 | | | -0.060013447 | ⊕⊕○○ Low | -0.060013447 | ⊕⊕○○ Low |

| | | | | | | | | |
|---|---|---|---|---|---|---|---|---|
| 108 | Ertugliflozin_high_dosage:Inject_semaglutide_high_dosage | 0 | | | 0.193168456 | ⊕⊕○○ Low | 0.193168456 | ⊕⊕○○ Low |
| 109 | Ertugliflozin_high_dosage:Inject_semaglutide_low_dosage | 0 | | | 0.418696998 | ⊕⊕○○ Low | 0.418696998 | ⊕⊕○○ Low |
| 110 | Ertugliflozin_high_dosage:Inject_semaglutide_medium_dosage | 0 | | | 0.413845563 | ⊕⊕○○ Low | 0.413845563 | ⊕⊕○○ Low |
| 111 | Ertugliflozin_high_dosage:Liraglutide | 0 | | | 0.609862276 | ⊕⊕○○ Low | 0.609862276 | ⊕⊕○○ Low |
| 112 | Ertugliflozin_high_dosage:Lixisenatide | 0 | | | -1.088766174 | ⊕⊕○○ Low | -1.088766174 | ⊕⊕○○ Low |
| 113 | Ertugliflozin_high_dosage:Oral_semaglutide | 0 | | | -0.540710338 | ⊕⊕○○ Low | -0.540710338 | ⊕⊕○○ Low |
| 114 | Ertugliflozin_high_dosage:Placebo_or_Control | 2 | 0.009673603 | ⊕⊕⊕⊕ High | 0.013834899 | ⊕⊕⊕⊕ High | 0.010505855 | ⊕⊕⊕⊕ High |
| 115 | Ertugliflozin_high_dosage:Sotagliflozin | 0 | | | -0.056372191 | ⊕⊕○○ Low | -0.056372191 | ⊕⊕○○ Low |
| 116 | Ertugliflozin_high_dosage:Tirzepatide | 0 | | | 2.191516688 | ⊕⊕○○ Low | 2.191516688 | ⊕⊕○○ Low |
| 117 | Ertugliflozin_low_dosage:Exenatide | 0 | | | -1.17152178 | ⊕⊕○○ Low | -1.17152178 | ⊕⊕○○ Low |
| 118 | Ertugliflozin_low_dosage:Inject_semaglutide_high_dosage | 0 | | | -0.918339877 | ⊕⊕○○ Low | -0.918339877 | ⊕⊕○○ Low |
| 119 | Ertugliflozin_low_dosage:Inject_semaglutide_low_dosage | 0 | | | -0.692811335 | ⊕⊕○○ Low | -0.692811335 | ⊕⊕○○ Low |
| 120 | Ertugliflozin_low_dosage:Inject_semaglutide_medium_dosage | 0 | | | -0.69766277 | ⊕⊕○○ Low | -0.69766277 | ⊕⊕○○ Low |
| 121 | Ertugliflozin_low_dosage:Liraglutide | 0 | | | -0.501646057 | ⊕⊕○○ Low | -0.501646057 | ⊕⊕○○ Low |
| 122 | Ertugliflozin_low_dosage:Lixisenatide | 0 | | | -2.200274507 | ⊕⊕○○ Low | -2.200274507 | ⊕⊕○○ Low |
| 123 | Ertugliflozin_low_dosage:Oral_semaglutide | 0 | | | -1.652218671 | ⊕⊕○○ Low | -1.652218671 | ⊕⊕○○ Low |
| 124 | Ertugliflozin_low_dosage:Placebo_or_Control | 1 | -1.099340753 | ⊕⊕⊕⊕ High | -1.10350205 | ⊕⊕⊕⊕ High | -1.101002478 | ⊕⊕⊕⊕ High |
| 125 | Ertugliflozin_low_dosage:Sotagliflozin | 0 | | | -1.167880524 | ⊕⊕○○ Low | -1.167880524 | ⊕⊕○○ Low |
| 126 | Ertugliflozin_low_dosage:Tirzepatide | 0 | | | 1.080008355 | ⊕⊕○○ Low | 1.080008355 | ⊕⊕○○ Low |
| 127 | Exenatide:Inject_semaglutide_high_dosage | 0 | | | 0.253181903 | ⊕⊕○○ Low | 0.253181903 | ⊕⊕○○ Low |
| 128 | Exenatide:Inject_semaglutide_low_dosage | 0 | | | 0.478710445 | ⊕⊕○○ Low | 0.478710445 | ⊕⊕○○ Low |
| 129 | Exenatide:Inject_semaglutide_medium_dosage | 0 | | | 0.47385901 | ⊕⊕○○ Low | 0.47385901 | ⊕⊕○○ Low |

| | | | | | | | | |
|---|---|---|---|---|---|---|---|---|
| 130 | Exenatide:Liraglutide | 1 | 1.115743329 | ⊕⊕⊕⊕ High | 0.514204965 | ⊕⊕⊕⊕ High | 0.669875723 | ⊕⊕⊕⊕ High |
| 131 | Exenatide:Lixisenatide | 0 | | | -1.028752727 | ⊕⊕○○ Low | -1.028752727 | ⊕⊕○○ Low |
| 132 | Exenatide:Oral_semaglutide | 0 | | | -0.480696891 | ⊕⊕○○ Low | -0.480696891 | ⊕⊕○○ Low |
| 133 | Exenatide:Placebo_or_Control | 1 | 0.003807974 | ⊕⊕⊕⊕ High | 0.605346338 | ⊕⊕⊕⊕ High | 0.070519301 | ⊕⊕⊕⊕ High |
| 134 | Exenatide:Sotagliflozin | 0 | | | 0.003641256 | ⊕⊕○○ Low | 0.003641256 | ⊕⊕○○ Low |
| 135 | Exenatide:Tirzepatide | 0 | | | 2.251530134 | ⊕⊕○○ Low | 2.251530134 | ⊕⊕○○ Low |
| 136 | Inject_semaglutide_high_dosage:Inject_semaglutide_low_dosage | 0 | | | 0.225528542 | ⊕⊕○○ Low | 0.225528542 | ⊕⊕○○ Low |
| 137 | Inject_semaglutide_high_dosage:Inject_semaglutide_medium_dosage | 0 | | | 0.220677107 | ⊕⊕○○ Low | 0.220677107 | ⊕⊕○○ Low |
| 138 | Inject_semaglutide_high_dosage:Liraglutide | 0 | | | 0.41669382 | ⊕⊕○○ Low | 0.41669382 | ⊕⊕○○ Low |
| 139 | Inject_semaglutide_high_dosage:Lixisenatide | 0 | | | -1.28193463 | ⊕⊕○○ Low | -1.28193463 | ⊕⊕○○ Low |
| 140 | Inject_semaglutide_high_dosage:Oral_semaglutide | 0 | | | -0.733878794 | ⊕⊕○○ Low | -0.733878794 | ⊕⊕○○ Low |
| 141 | Inject_semaglutide_high_dosage:Placebo_or_Control | 1 | -0.182662602 | ⊕⊕⊕⊕ High | | | -0.182662602 | ⊕⊕⊕○ Medium |
| 142 | Inject_semaglutide_high_dosage:Sotagliflozin | 0 | | | -0.249540647 | ⊕⊕○○ Low | -0.249540647 | ⊕⊕○○ Low |
| 143 | Inject_semaglutide_high_dosage:Tirzepatide | 0 | | | 1.998348231 | ⊕⊕○○ Low | 1.998348231 | ⊕⊕○○ Low |
| 144 | Inject_semaglutide_low_dosage:Inject_semaglutide_medium_dosage | 0 | | | -0.004851435 | ⊕⊕○○ Low | -0.004851435 | ⊕⊕○○ Low |
| 145 | Inject_semaglutide_low_dosage:Liraglutide | 0 | | | 0.191165278 | ⊕⊕○○ Low | 0.191165278 | ⊕⊕○○ Low |
| 146 | Inject_semaglutide_low_dosage:Lixisenatide | 0 | | | -1.507463172 | ⊕⊕○○ Low | -1.507463172 | ⊕⊕○○ Low |
| 147 | Inject_semaglutide_low_dosage:Oral_semaglutide | 0 | | | -0.959407336 | ⊕⊕○○ Low | -0.959407336 | ⊕⊕○○ Low |
| 148 | Inject_semaglutide_low_dosage:Placebo_or_Control | 1 | -0.408191143 | ⊕⊕⊕⊕ High | | | -0.408191143 | ⊕⊕⊕○ Medium |
| 149 | Inject_semaglutide_low_dosage:Sotagliflozin | 0 | | | -0.475069189 | ⊕⊕○○ Low | -0.475069189 | ⊕⊕○○ Low |
| 150 | Inject_semaglutide_low_dosage:Tirzepatide | 0 | | | 1.77281969 | ⊕⊕○○ Low | 1.77281969 | ⊕⊕○○ Low |
| 151 | Inject_semaglutide_medium_dosage:Liraglutide | 0 | | | 0.196016713 | ⊕⊕○○ Low | 0.196016713 | ⊕⊕○○ Low |

| | | | | | | | | |
|---|---|---|---|---|---|---|---|---|
| 152 | Inject_semaglutide_medium_dosage:Lixisenatide | 0 | | | -1.502611737 | ⊕⊕○○ Low | -1.502611737 | ⊕⊕○○ Low |
| 153 | Inject_semaglutide_medium_dosage:Oral_semaglutide | 0 | | | -0.954555901 | ⊕⊕○○ Low | -0.954555901 | ⊕⊕○○ Low |
| 154 | Inject_semaglutide_medium_dosage:Placebo_or_Control | 1 | -0.403339709 | ⊕⊕⊕⊕ High | | | -0.403339709 | ⊕⊕⊕○ Medium |
| 155 | Inject_semaglutide_medium_dosage:Sotagliflozin | 0 | | | -0.470217755 | ⊕⊕○○ Low | -0.470217755 | ⊕⊕○○ Low |
| 156 | Inject_semaglutide_medium_dosage:Tirzepatide | 0 | | | 1.777671124 | ⊕⊕○○ Low | 1.777671124 | ⊕⊕○○ Low |
| 157 | Liraglutide:Lixisenatide | 0 | | | -1.69862845 | ⊕⊕○○ Low | -1.69862845 | ⊕⊕○○ Low |
| 158 | Liraglutide:Oral_semaglutide | 0 | | | -1.150572614 | ⊕⊕○○ Low | -1.150572614 | ⊕⊕○○ Low |
| 159 | Liraglutide:Placebo_or_Control | 1 | -0.510396991 | ⊕⊕⊕⊕ High | -1.111935354 | ⊕⊕⊕⊕ High | -0.599356422 | ⊕⊕⊕⊕ High |
| 160 | Liraglutide:Sotagliflozin | 0 | | | -0.666234468 | ⊕⊕○○ Low | -0.666234468 | ⊕⊕○○ Low |
| 161 | Liraglutide:Tirzepatide | 0 | | | 1.581654411 | ⊕⊕○○ Low | 1.581654411 | ⊕⊕○○ Low |
| 162 | Lixisenatide:Oral_semaglutide | 0 | | | 0.548055836 | ⊕⊕○○ Low | 0.548055836 | ⊕⊕○○ Low |
| 163 | Lixisenatide:Placebo_or_Control | 1 | 1.099272028 | ⊕⊕⊕⊕ High | | | 1.099272028 | ⊕⊕⊕○ Medium |
| 164 | Lixisenatide:Sotagliflozin | 0 | | | 1.032393982 | ⊕⊕○○ Low | 1.032393982 | ⊕⊕○○ Low |
| 165 | Lixisenatide:Tirzepatide | 0 | | | 3.280282861 | ⊕⊕○○ Low | 3.280282861 | ⊕⊕○○ Low |
| 166 | Oral_semaglutide:Placebo_or_Control | 2 | 0.551216193 | ⊕⊕⊕⊕ High | | | 0.551216193 | ⊕⊕⊕○ Medium |
| 167 | Oral_semaglutide:Sotagliflozin | 0 | | | 0.484338147 | ⊕⊕○○ Low | 0.484338147 | ⊕⊕○○ Low |
| 168 | Oral_semaglutide:Tirzepatide | 0 | | | 2.732227026 | ⊕⊕○○ Low | 2.732227026 | ⊕⊕○○ Low |
| 169 | Sotagliflozin:Placebo_or_Control | 2 | 0.066878046 | ⊕⊕⊕⊕ High | | | 0.066878046 | ⊕⊕⊕○ Medium |
| 170 | Tirzepatide:Placebo_or_Control | 1 | -2.181010833 | ⊕⊕⊕⊕ High | | | -2.181010833 | |
| 171 | Sotagliflozin:Tirzepatide | 0 | | | 2.247888879 | ⊕⊕○○ Low | 2.247888879 | ⊕⊕○○ Low |

## Table S8E: GRADE of safety profile: drop-out rate

| | Comparison | No.Studies | Direct | | Indirect | | NMA | |
|---|---|---|---|---|---|---|---|---|
| | | | Estimate | Rate | Estimate | Rate | Estimate | Rate |
| 1 | Albiglutide:Bexagliflozin | 0 | | | 0.029218958 | ⊕⊕○○ Low | 0.029218958 | ⊕⊕○○ Low |
| 2 | Albiglutide:Canagliflozin_high_dosage | 0 | | | 0.243731565 | ⊕⊕○○ Low | 0.243731565 | ⊕⊕○○ Low |
| 3 | Albiglutide:Canagliflozin_low_dosage | 0 | | | 0.144417243 | ⊕⊕○○ Low | 0.144417243 | ⊕⊕○○ Low |
| 4 | Albiglutide:Dapagliflozin_high_dosage | 0 | | | 0.132491881 | ⊕⊕○○ Low | 0.132491881 | ⊕⊕○○ Low |
| 5 | Albiglutide:Dapagliflozin_low_dosage | 0 | | | 0.127568939 | ⊕⊕○○ Low | 0.127568939 | ⊕⊕○○ Low |
| 6 | Albiglutide:Dapagliflozin_medium_dosage | 0 | | | 0.108768998 | ⊕⊕○○ Low | 0.108768998 | ⊕⊕○○ Low |
| 7 | Albiglutide:Dulaglutide | 0 | | | -0.083654418 | ⊕⊕○○ Low | -0.083654418 | ⊕⊕○○ Low |
| 8 | Albiglutide:Efpeglenatide_high_dosage | 0 | | | -0.090086972 | ⊕⊕○○ Low | -0.090086972 | ⊕⊕○○ Low |
| 9 | Albiglutide:Efpeglenatide_medium_dosage | 0 | | | -0.070259529 | ⊕⊕○○ Low | -0.070259529 | ⊕⊕○○ Low |
| 10 | Albiglutide:Empagliflozin_high_dosage | 0 | | | 0.200613003 | ⊕⊕○○ Low | 0.200613003 | ⊕⊕○○ Low |
| 11 | Albiglutide:Empagliflozin_low_dosage | 0 | | | 0.030884639 | ⊕⊕○○ Low | 0.030884639 | ⊕⊕○○ Low |
| 12 | Albiglutide:Ertugliflozin_high_dosage | 0 | | | -0.025495097 | ⊕⊕○○ Low | -0.025495097 | ⊕⊕○○ Low |
| 13 | Albiglutide:Ertugliflozin_low_dosage | 0 | | | 0.082537251 | ⊕⊕○○ Low | 0.082537251 | ⊕⊕○○ Low |
| 14 | Albiglutide:Exenatide | 0 | | | 0.097246873 | ⊕⊕○○ Low | 0.097246873 | ⊕⊕○○ Low |
| 15 | Albiglutide:Inject_semaglutide_high_dosage | 0 | | | 0.240902346 | ⊕⊕○○ Low | 0.240902346 | ⊕⊕○○ Low |
| 16 | Albiglutide:Inject_semaglutide_low_dosage | 0 | | | 0.389899155 | ⊕⊕○○ Low | 0.389899155 | ⊕⊕○○ Low |
| 17 | Albiglutide:Inject_semaglutide_medium_dosage | 0 | | | 0.33062504 | ⊕⊕○○ Low | 0.33062504 | ⊕⊕○○ Low |
| 18 | Albiglutide:Liraglutide | 0 | | | 0.588612086 | ⊕⊕○○ Low | 0.588612086 | ⊕⊕○○ Low |
| 19 | Albiglutide:Lixisenatide | 0 | | | 0.075071443 | ⊕⊕○○ Low | 0.075071443 | ⊕⊕○○ Low |

| | | | | | | | | |
|---|---|---|---|---|---|---|---|---|
| 20 | Albiglutide:Oral_semaglutide | 0 | | | -0.564978106 | ⊕⊕○○ Low | -0.564978106 | ⊕⊕○○ Low |
| 21 | Albiglutide:Placebo_or_Control | 5 | -0.103216354 | ⊕⊕⊕⊕ High | | | -0.103216354 | ⊕⊕⊕○ Medium |
| 22 | Albiglutide:Sotagliflozin | 0 | | | -0.016120889 | ⊕⊕○○ Low | -0.016120889 | ⊕⊕○○ Low |
| 23 | Albiglutide:Tirzepatide | 0 | | | 0.481489458 | ⊕⊕○○ Low | 0.481489458 | ⊕⊕○○ Low |
| 24 | Bexagliflozin:Canagliflozin_high_dosage | 0 | | | 0.214512607 | ⊕⊕○○ Low | 0.214512607 | ⊕⊕○○ Low |
| 25 | Bexagliflozin:Canagliflozin_low_dosage | 0 | | | 0.115198285 | ⊕⊕○○ Low | 0.115198285 | ⊕⊕○○ Low |
| 26 | Bexagliflozin:Dapagliflozin_high_dosage | 0 | | | 0.103272923 | ⊕⊕○○ Low | 0.103272923 | ⊕⊕○○ Low |
| 27 | Bexagliflozin:Dapagliflozin_low_dosage | 0 | | | 0.098349981 | ⊕⊕○○ Low | 0.098349981 | ⊕⊕○○ Low |
| 28 | Bexagliflozin:Dapagliflozin_medium_dosage | 0 | | | 0.07955004 | ⊕⊕○○ Low | 0.07955004 | ⊕⊕○○ Low |
| 29 | Bexagliflozin:Dulaglutide | 0 | | | -0.112873377 | ⊕⊕○○ Low | -0.112873377 | ⊕⊕○○ Low |
| 30 | Bexagliflozin:Efpeglenatide_high_dosage | 0 | | | -0.119305931 | ⊕⊕○○ Low | -0.119305931 | ⊕⊕○○ Low |
| 31 | Bexagliflozin:Efpeglenatide_medium_dosage | 0 | | | -0.099478487 | ⊕⊕○○ Low | -0.099478487 | ⊕⊕○○ Low |
| 32 | Bexagliflozin:Empagliflozin_high_dosage | 0 | | | 0.171394044 | ⊕⊕○○ Low | 0.171394044 | ⊕⊕○○ Low |
| 33 | Bexagliflozin:Empagliflozin_low_dosage | 0 | | | 0.001665681 | ⊕⊕○○ Low | 0.001665681 | ⊕⊕○○ Low |
| 34 | Bexagliflozin:Ertugliflozin_high_dosage | 0 | | | -0.054714055 | ⊕⊕○○ Low | -0.054714055 | ⊕⊕○○ Low |
| 35 | Bexagliflozin:Ertugliflozin_low_dosage | 0 | | | 0.053318293 | ⊕⊕○○ Low | 0.053318293 | ⊕⊕○○ Low |
| 36 | Bexagliflozin:Exenatide | 0 | | | 0.068027915 | ⊕⊕○○ Low | 0.068027915 | ⊕⊕○○ Low |
| 37 | Bexagliflozin:Inject_semaglutide_high_dosage | 0 | | | 0.211683388 | ⊕⊕○○ Low | 0.211683388 | ⊕⊕○○ Low |
| 38 | Bexagliflozin:Inject_semaglutide_low_dosage | 0 | | | 0.360680197 | ⊕⊕○○ Low | 0.360680197 | ⊕⊕○○ Low |
| 39 | Bexagliflozin:Inject_semaglutide_medium_dosage | 0 | | | 0.301406082 | ⊕⊕○○ Low | 0.301406082 | ⊕⊕○○ Low |
| 40 | Bexagliflozin:Liraglutide | 0 | | | 0.559393127 | ⊕⊕○○ Low | 0.559393127 | ⊕⊕○○ Low |
| 41 | Bexagliflozin:Lixisenatide | 0 | | | 0.045852485 | ⊕⊕○○ Low | 0.045852485 | ⊕⊕○○ Low |

| | | | | | | | | |
|---|---|---|---|---|---|---|---|---|
| 42 | Bexagliflozin:Oral_semaglutide | 0 | | | -0.594197065 | ⊕⊕○○ Low | -0.594197065 | ⊕⊕○○ Low |
| 43 | Bexagliflozin:Placebo_or_Control | 1 | -0.132435312 | ⊕⊕⊕⊕ High | | | -0.132435312 | ⊕⊕⊕○ Medium |
| 44 | Bexagliflozin:Sotagliflozin | 0 | | | -0.045339847 | ⊕⊕○○ Low | -0.045339847 | ⊕⊕○○ Low |
| 45 | Bexagliflozin:Tirzepatide | 0 | | | 0.4522705 | ⊕⊕○○ Low | 0.4522705 | ⊕⊕○○ Low |
| 46 | Canagliflozin_high_dosage:Canagliflozin_low_dosage | 1 | -0.145934915 | ⊕⊕⊕⊕ High | 0.010735285 | ⊕⊕⊕○ Medium | -0.099314322 | ⊕⊕⊕⊕ High |
| 47 | Canagliflozin_high_dosage:Dapagliflozin_high_dosage | 0 | | | -0.111239684 | ⊕⊕○○ Low | -0.111239684 | ⊕⊕○○ Low |
| 48 | Canagliflozin_high_dosage:Dapagliflozin_low_dosage | 0 | | | -0.116162626 | ⊕⊕○○ Low | -0.116162626 | ⊕⊕○○ Low |
| 49 | Canagliflozin_high_dosage:Dapagliflozin_medium_dosage | 0 | | | -0.134962567 | ⊕⊕○○ Low | -0.134962567 | ⊕⊕○○ Low |
| 50 | Canagliflozin_high_dosage:Dulaglutide | 0 | | | -0.327385984 | ⊕⊕○○ Low | -0.327385984 | ⊕⊕○○ Low |
| 51 | Canagliflozin_high_dosage:Efpeglenatide_high_dosage | 0 | | | -0.333818538 | ⊕⊕○○ Low | -0.333818538 | ⊕⊕○○ Low |
| 52 | Canagliflozin_high_dosage:Efpeglenatide_medium_dosage | 0 | | | -0.313991094 | ⊕⊕○○ Low | -0.313991094 | ⊕⊕○○ Low |
| 53 | Canagliflozin_high_dosage:Empagliflozin_high_dosage | 0 | | | -0.043118563 | ⊕⊕○○ Low | -0.043118563 | ⊕⊕○○ Low |
| 54 | Canagliflozin_high_dosage:Empagliflozin_low_dosage | 0 | | | -0.212846926 | ⊕⊕○○ Low | -0.212846926 | ⊕⊕○○ Low |
| 55 | Canagliflozin_high_dosage:Ertugliflozin_high_dosage | 0 | | | -0.269226663 | ⊕⊕○○ Low | -0.269226663 | ⊕⊕○○ Low |
| 56 | Canagliflozin_high_dosage:Ertugliflozin_low_dosage | 0 | | | -0.161194314 | ⊕⊕○○ Low | -0.161194314 | ⊕⊕○○ Low |
| 57 | Canagliflozin_high_dosage:Exenatide | 0 | | | -0.146484692 | ⊕⊕○○ Low | -0.146484692 | ⊕⊕○○ Low |
| 58 | Canagliflozin_high_dosage:Inject_semaglutide_high_dosage | 0 | | | -0.002829219 | ⊕⊕○○ Low | -0.002829219 | ⊕⊕○○ Low |
| 59 | Canagliflozin_high_dosage:Inject_semaglutide_low_dosage | 0 | | | 0.14616759 | ⊕⊕○○ Low | 0.14616759 | ⊕⊕○○ Low |
| 60 | Canagliflozin_high_dosage:Inject_semaglutide_medium_dosage | 0 | | | 0.086893475 | ⊕⊕○○ Low | 0.086893475 | ⊕⊕○○ Low |
| 61 | Canagliflozin_high_dosage:Liraglutide | 0 | | | 0.34488052 | ⊕⊕○○ Low | 0.34488052 | ⊕⊕○○ Low |
| 62 | Canagliflozin_high_dosage:Lixisenatide | 0 | | | -0.168660122 | ⊕⊕○○ Low | -0.168660122 | ⊕⊕○○ Low |
| 63 | Canagliflozin_high_dosage:Oral_semaglutide | 0 | | | -0.808709672 | ⊕⊕○○ Low | -0.808709672 | ⊕⊕○○ Low |

| | | | | | | | | |
|---|---|---|---|---|---|---|---|---|
| 64 | Canagliflozin_high_dosage:Placebo_or_Control | 2 | -0.368088873 | ⊕⊕⊕⊕ High | -0.062216635 | ⊕⊕⊕⊕ High | -0.34694792 | ⊕⊕⊕⊕ High |
| 65 | Canagliflozin_high_dosage:Sotagliflozin | 0 | | | -0.259852454 | ⊕⊕○○ Low | -0.259852454 | ⊕⊕○○ Low |
| 66 | Canagliflozin_high_dosage:Tirzepatide | 0 | | | 0.237757893 | ⊕⊕○○ Low | 0.237757893 | ⊕⊕○○ Low |
| 67 | Canagliflozin_low_dosage:Dapagliflozin_high_dosage | 0 | | | -0.011925362 | ⊕⊕○○ Low | -0.011925362 | ⊕⊕○○ Low |
| 68 | Canagliflozin_low_dosage:Dapagliflozin_low_dosage | 0 | | | -0.016848304 | ⊕⊕○○ Low | -0.016848304 | ⊕⊕○○ Low |
| 69 | Canagliflozin_low_dosage:Dapagliflozin_medium_dosage | 0 | | | -0.035648245 | ⊕⊕○○ Low | -0.035648245 | ⊕⊕○○ Low |
| 70 | Canagliflozin_low_dosage:Dulaglutide | 0 | | | -0.228071661 | ⊕⊕○○ Low | -0.228071661 | ⊕⊕○○ Low |
| 71 | Canagliflozin_low_dosage:Efpeglenatide_high_dosage | 0 | | | -0.234504215 | ⊕⊕○○ Low | -0.234504215 | ⊕⊕○○ Low |
| 72 | Canagliflozin_low_dosage:Efpeglenatide_medium_dosage | 0 | | | -0.214676772 | ⊕⊕○○ Low | -0.214676772 | ⊕⊕○○ Low |
| 73 | Canagliflozin_low_dosage:Empagliflozin_high_dosage | 0 | | | 0.05619576 | ⊕⊕○○ Low | 0.05619576 | ⊕⊕○○ Low |
| 74 | Canagliflozin_low_dosage:Empagliflozin_low_dosage | 0 | | | -0.113532604 | ⊕⊕○○ Low | -0.113532604 | ⊕⊕○○ Low |
| 75 | Canagliflozin_low_dosage:Ertugliflozin_high_dosage | 0 | | | -0.16991234 | ⊕⊕○○ Low | -0.16991234 | ⊕⊕○○ Low |
| 76 | Canagliflozin_low_dosage:Ertugliflozin_low_dosage | 0 | | | -0.061879992 | ⊕⊕○○ Low | -0.061879992 | ⊕⊕○○ Low |
| 77 | Canagliflozin_low_dosage:Exenatide | 0 | | | -0.04717037 | ⊕⊕○○ Low | -0.04717037 | ⊕⊕○○ Low |
| 78 | Canagliflozin_low_dosage:Inject_semaglutide_high_dosage | 0 | | | 0.096485103 | ⊕⊕○○ Low | 0.096485103 | ⊕⊕○○ Low |
| 79 | Canagliflozin_low_dosage:Inject_semaglutide_low_dosage | 0 | | | 0.245481913 | ⊕⊕○○ Low | 0.245481913 | ⊕⊕○○ Low |
| 80 | Canagliflozin_low_dosage:Inject_semaglutide_medium_dosage | 0 | | | 0.186207798 | ⊕⊕○○ Low | 0.186207798 | ⊕⊕○○ Low |
| 81 | Canagliflozin_low_dosage:Liraglutide | 0 | | | 0.444194843 | ⊕⊕○○ Low | 0.444194843 | ⊕⊕○○ Low |
| 82 | Canagliflozin_low_dosage:Lixisenatide | 0 | | | -0.0693458 | ⊕⊕○○ Low | -0.0693458 | ⊕⊕○○ Low |
| 83 | Canagliflozin_low_dosage:Oral_semaglutide | 0 | | | -0.709395349 | ⊕⊕○○ Low | -0.709395349 | ⊕⊕○○ Low |
| 84 | Canagliflozin_low_dosage:Placebo_or_Control | 3 | -0.286047782 | ⊕⊕⊕⊕ High | 0.505331623 | ⊕⊕⊕○ Medium | -0.247633597 | ⊕⊕⊕⊕ High |
| 85 | Canagliflozin_low_dosage:Sotagliflozin | 0 | | | -0.160538132 | ⊕⊕○○ Low | -0.160538132 | ⊕⊕○○ Low |

| | | | | | | | | |
|---|---|---|---|---|---|---|---|---|
| 86 | Canagliflozin_low_dosage:Tirzepatide | 0 | | | 0.337072215 | ⊕⊕○○ Low | 0.337072215 | ⊕⊕○○ Low |
| 87 | Dapagliflozin_high_dosage:Dapagliflozin_low_dosage | 2 | -0.125817225 | ⊕⊕⊕⊕ High | 0.503435556 | ⊕⊕⊕○ Medium | -0.004922942 | ⊕⊕⊕⊕ High |
| 88 | Dapagliflozin_high_dosage:Dapagliflozin_medium_dosage | 2 | -0.140554571 | ⊕⊕⊕⊕ High | 0.456903263 | ⊕⊕⊕○ Medium | -0.023722883 | ⊕⊕⊕⊕ High |
| 89 | Dapagliflozin_high_dosage:Dulaglutide | 0 | | | -0.216146299 | ⊕⊕○○ Low | -0.216146299 | ⊕⊕○○ Low |
| 90 | Dapagliflozin_high_dosage:Efpeglenatide_high_dosage | 0 | | | -0.222578853 | ⊕⊕○○ Low | -0.222578853 | ⊕⊕○○ Low |
| 91 | Dapagliflozin_high_dosage:Efpeglenatide_medium_dosage | 0 | | | -0.20275141 | ⊕⊕○○ Low | -0.20275141 | ⊕⊕○○ Low |
| 92 | Dapagliflozin_high_dosage:Empagliflozin_high_dosage | 0 | | | 0.068121122 | ⊕⊕○○ Low | 0.068121122 | ⊕⊕○○ Low |
| 93 | Dapagliflozin_high_dosage:Empagliflozin_low_dosage | 0 | | | -0.101607242 | ⊕⊕○○ Low | -0.101607242 | ⊕⊕○○ Low |
| 94 | Dapagliflozin_high_dosage:Ertugliflozin_high_dosage | 0 | | | -0.157986978 | ⊕⊕○○ Low | -0.157986978 | ⊕⊕○○ Low |
| 95 | Dapagliflozin_high_dosage:Ertugliflozin_low_dosage | 0 | | | -0.04995463 | ⊕⊕○○ Low | -0.04995463 | ⊕⊕○○ Low |
| 96 | Dapagliflozin_high_dosage:Exenatide | 0 | | | -0.035245008 | ⊕⊕○○ Low | -0.035245008 | ⊕⊕○○ Low |
| 97 | Dapagliflozin_high_dosage:Inject_semaglutide_high_dosage | 0 | | | 0.108410465 | ⊕⊕○○ Low | 0.108410465 | ⊕⊕○○ Low |
| 98 | Dapagliflozin_high_dosage:Inject_semaglutide_low_dosage | 0 | | | 0.257407275 | ⊕⊕○○ Low | 0.257407275 | ⊕⊕○○ Low |
| 99 | Dapagliflozin_high_dosage:Inject_semaglutide_medium_dosage | 0 | | | 0.19813316 | ⊕⊕○○ Low | 0.19813316 | ⊕⊕○○ Low |
| 100 | Dapagliflozin_high_dosage:Liraglutide | 0 | | | 0.456120205 | ⊕⊕○○ Low | 0.456120205 | ⊕⊕○○ Low |
| 101 | Dapagliflozin_high_dosage:Lixisenatide | 0 | | | -0.057420437 | ⊕⊕○○ Low | -0.057420437 | ⊕⊕○○ Low |
| 102 | Dapagliflozin_high_dosage:Oral_semaglutide | 0 | | | -0.697469987 | ⊕⊕○○ Low | -0.697469987 | ⊕⊕○○ Low |
| 103 | Dapagliflozin_high_dosage:Placebo_or_Control | 6 | -0.236268149 | ⊕⊕⊕⊕ High | | | -0.235708235 | ⊕⊕⊕○ Medium |
| 104 | Dapagliflozin_high_dosage:Sotagliflozin | 0 | | | -0.14861277 | ⊕⊕○○ Low | -0.14861277 | ⊕⊕○○ Low |
| 105 | Dapagliflozin_high_dosage:Tirzepatide | 0 | | | 0.348997577 | ⊕⊕○○ Low | 0.348997577 | ⊕⊕○○ Low |
| 106 | Dapagliflozin_low_dosage:Dapagliflozin_medium_dosage | 2 | -0.015094838 | ⊕⊕⊕⊕ High | -3.904646189 | ⊕⊕⊕⊕ High | -0.018799941 | ⊕⊕⊕⊕ High |
| 107 | Dapagliflozin_low_dosage:Dulaglutide | 0 | | | -0.211223358 | ⊕⊕○○ Low | -0.211223358 | ⊕⊕○○ Low |

| | | | | | | | | |
|---|---|---|---|---|---|---|---|---|
| 108 | Dapagliflozin_low_dosage:Efpeglenatide_high_dosage | 0 | | | -0.217655912 | ⊕⊕○○ Low | -0.217655912 | ⊕⊕○○ Low |
| 109 | Dapagliflozin_low_dosage:Efpeglenatide_medium_dosage | 0 | | | -0.197828468 | ⊕⊕○○ Low | -0.197828468 | ⊕⊕○○ Low |
| 110 | Dapagliflozin_low_dosage:Empagliflozin_high_dosage | 0 | | | 0.073044063 | ⊕⊕○○ Low | 0.073044063 | ⊕⊕○○ Low |
| 111 | Dapagliflozin_low_dosage:Empagliflozin_low_dosage | 0 | | | -0.0966843 | ⊕⊕○○ Low | -0.0966843 | ⊕⊕○○ Low |
| 112 | Dapagliflozin_low_dosage:Ertugliflozin_high_dosage | 0 | | | -0.153064037 | ⊕⊕○○ Low | -0.153064037 | ⊕⊕○○ Low |
| 113 | Dapagliflozin_low_dosage:Ertugliflozin_low_dosage | 0 | | | -0.045031688 | ⊕⊕○○ Low | -0.045031688 | ⊕⊕○○ Low |
| 114 | Dapagliflozin_low_dosage:Exenatide | 0 | | | -0.030322066 | ⊕⊕○○ Low | -0.030322066 | ⊕⊕○○ Low |
| 115 | Dapagliflozin_low_dosage:Inject_semaglutide_high_dosage | 0 | | | 0.113333407 | ⊕⊕○○ Low | 0.113333407 | ⊕⊕○○ Low |
| 116 | Dapagliflozin_low_dosage:Inject_semaglutide_low_dosage | 0 | | | 0.262330216 | ⊕⊕○○ Low | 0.262330216 | ⊕⊕○○ Low |
| 117 | Dapagliflozin_low_dosage:Inject_semaglutide_medium_dosage | 0 | | | 0.203056101 | ⊕⊕○○ Low | 0.203056101 | ⊕⊕○○ Low |
| 118 | Dapagliflozin_low_dosage:Liraglutide | 0 | | | 0.461043146 | ⊕⊕○○ Low | 0.461043146 | ⊕⊕○○ Low |
| 119 | Dapagliflozin_low_dosage:Lixisenatide | 0 | | | -0.052497496 | ⊕⊕○○ Low | -0.052497496 | ⊕⊕○○ Low |
| 120 | Dapagliflozin_low_dosage:Oral_semaglutide | 0 | | | -0.692547046 | ⊕⊕○○ Low | -0.692547046 | ⊕⊕○○ Low |
| 121 | Dapagliflozin_low_dosage:Placebo_or_Control | 2 | -0.32489795 | ⊕⊕⊕⊕ High | 0.369058973 | ⊕⊕⊕○ Medium | -0.230785294 | ⊕⊕⊕⊕ High |
| 122 | Dapagliflozin_low_dosage:Sotagliflozin | 0 | | | -0.143689828 | ⊕⊕○○ Low | -0.143689828 | ⊕⊕○○ Low |
| 123 | Dapagliflozin_low_dosage:Tirzepatide | 0 | | | 0.353920519 | ⊕⊕○○ Low | 0.353920519 | ⊕⊕○○ Low |
| 124 | Dapagliflozin_medium_dosage:Dulaglutide | 0 | | | -0.192423417 | ⊕⊕○○ Low | -0.192423417 | ⊕⊕○○ Low |
| 125 | Dapagliflozin_medium_dosage:Efpeglenatide_high_dosage | 0 | | | -0.198855971 | ⊕⊕○○ Low | -0.198855971 | ⊕⊕○○ Low |
| 126 | Dapagliflozin_medium_dosage:Efpeglenatide_medium_dosage | 0 | | | -0.179028527 | ⊕⊕○○ Low | -0.179028527 | ⊕⊕○○ Low |
| 127 | Dapagliflozin_medium_dosage:Empagliflozin_high_dosage | 0 | | | 0.091844004 | ⊕⊕○○ Low | 0.091844004 | ⊕⊕○○ Low |
| 128 | Dapagliflozin_medium_dosage:Empagliflozin_low_dosage | 0 | | | -0.077884359 | ⊕⊕○○ Low | -0.077884359 | ⊕⊕○○ Low |
| 129 | Dapagliflozin_medium_dosage:Ertugliflozin_high_dosage | 0 | | | -0.134264095 | ⊕⊕○○ Low | -0.134264095 | ⊕⊕○○ Low |

| | | | | | | | | |
|---|---|---|---|---|---|---|---|---|
| 130 | Dapagliflozin_medium_dosage:Ertugliflozin_low_dosage | 0 | | | -0.026231747 | ⊕⊕○○ Low | -0.026231747 | ⊕⊕○○ Low |
| 131 | Dapagliflozin_medium_dosage:Exenatide | 0 | | | -0.011522125 | ⊕⊕○○ Low | -0.011522125 | ⊕⊕○○ Low |
| 132 | Dapagliflozin_medium_dosage:Inject_semaglutide_high_dosage | 0 | | | 0.132133348 | ⊕⊕○○ Low | 0.132133348 | ⊕⊕○○ Low |
| 133 | Dapagliflozin_medium_dosage:Inject_semaglutide_low_dosage | 0 | | | 0.281130157 | ⊕⊕○○ Low | 0.281130157 | ⊕⊕○○ Low |
| 134 | Dapagliflozin_medium_dosage:Inject_semaglutide_medium_dosage | 0 | | | 0.221856042 | ⊕⊕○○ Low | 0.221856042 | ⊕⊕○○ Low |
| 135 | Dapagliflozin_medium_dosage:Liraglutide | 0 | | | 0.479843087 | ⊕⊕○○ Low | 0.479843087 | ⊕⊕○○ Low |
| 136 | Dapagliflozin_medium_dosage:Lixisenatide | 0 | | | -0.033697555 | ⊕⊕○○ Low | -0.033697555 | ⊕⊕○○ Low |
| 137 | Dapagliflozin_medium_dosage:Oral_semaglutide | 0 | | | -0.673747105 | ⊕⊕○○ Low | -0.673747105 | ⊕⊕○○ Low |
| 138 | Dapagliflozin_medium_dosage:Placebo_or_Control | 2 | -0.310499554 | ⊕⊕⊕⊕ High | 0.405953157 | ⊕⊕⊕○ Medium | -0.211985352 | ⊕⊕⊕⊕ High |
| 139 | Dapagliflozin_medium_dosage:Sotagliflozin | 0 | | | -0.124889887 | ⊕⊕○○ Low | -0.124889887 | ⊕⊕○○ Low |
| 140 | Dapagliflozin_medium_dosage:Tirzepatide | 0 | | | 0.37272046 | ⊕⊕○○ Low | 0.37272046 | ⊕⊕○○ Low |
| 141 | Dulaglutide:Efpeglenatide_high_dosage | 0 | | | -0.006432554 | ⊕⊕○○ Low | -0.006432554 | ⊕⊕○○ Low |
| 142 | Dulaglutide:Efpeglenatide_medium_dosage | 0 | | | 0.01339489 | ⊕⊕○○ Low | 0.01339489 | ⊕⊕○○ Low |
| 143 | Dulaglutide:Empagliflozin_high_dosage | 0 | | | 0.284267421 | ⊕⊕○○ Low | 0.284267421 | ⊕⊕○○ Low |
| 144 | Dulaglutide:Empagliflozin_low_dosage | 0 | | | 0.114539058 | ⊕⊕○○ Low | 0.114539058 | ⊕⊕○○ Low |
| 145 | Dulaglutide:Ertugliflozin_high_dosage | 0 | | | 0.058159321 | ⊕⊕○○ Low | 0.058159321 | ⊕⊕○○ Low |
| 146 | Dulaglutide:Ertugliflozin_low_dosage | 0 | | | 0.16619167 | ⊕⊕○○ Low | 0.16619167 | ⊕⊕○○ Low |
| 147 | Dulaglutide:Exenatide | 0 | | | 0.180901291 | ⊕⊕○○ Low | 0.180901291 | ⊕⊕○○ Low |
| 148 | Dulaglutide:Inject_semaglutide_high_dosage | 0 | | | 0.324556765 | ⊕⊕○○ Low | 0.324556765 | ⊕⊕○○ Low |
| 149 | Dulaglutide:Inject_semaglutide_low_dosage | 0 | | | 0.473553574 | ⊕⊕○○ Low | 0.473553574 | ⊕⊕○○ Low |
| 150 | Dulaglutide:Inject_semaglutide_medium_dosage | 0 | | | 0.414279459 | ⊕⊕○○ Low | 0.414279459 | ⊕⊕○○ Low |
| 151 | Dulaglutide:Liraglutide | 0 | | | 0.672266504 | ⊕⊕○○ Low | 0.672266504 | ⊕⊕○○ Low |

| | | | | | | | | |
|---|---|---|---|---|---|---|---|---|
| 152 | Dulaglutide:Lixisenatide | 0 | | | 0.158725862 | ⊕⊕○○ Low | 0.158725862 | ⊕⊕○○ Low |
| 153 | Dulaglutide:Oral_semaglutide | 0 | | | -0.481323688 | ⊕⊕○○ Low | -0.481323688 | ⊕⊕○○ Low |
| 154 | Dulaglutide:Placebo_or_Control | 3 | -0.019561936 | ⊕⊕⊕⊕ High | | | -0.019561936 | ⊕⊕⊕○ Medium |
| 155 | Dulaglutide:Sotagliflozin | 0 | | | 0.06753353 | ⊕⊕○○ Low | 0.06753353 | ⊕⊕○○ Low |
| 156 | Dulaglutide:Tirzepatide | 0 | | | 0.565143876 | ⊕⊕○○ Low | 0.565143876 | ⊕⊕○○ Low |
| 157 | Efpeglenatide_high_dosage:Efpeglenatide_medium_dosage | 1 | 0.019827444 | ⊕⊕⊕⊕ High | | | 0.019827444 | ⊕⊕⊕○ Medium |
| 158 | Efpeglenatide_high_dosage:Empagliflozin_high_dosage | 0 | | | 0.290699975 | ⊕⊕○○ Low | 0.290699975 | ⊕⊕○○ Low |
| 159 | Efpeglenatide_high_dosage:Empagliflozin_low_dosage | 0 | | | 0.120971612 | ⊕⊕○○ Low | 0.120971612 | ⊕⊕○○ Low |
| 160 | Efpeglenatide_high_dosage:Ertugliflozin_high_dosage | 0 | | | 0.064591875 | ⊕⊕○○ Low | 0.064591875 | ⊕⊕○○ Low |
| 161 | Efpeglenatide_high_dosage:Ertugliflozin_low_dosage | 0 | | | 0.172624224 | ⊕⊕○○ Low | 0.172624224 | ⊕⊕○○ Low |
| 162 | Efpeglenatide_high_dosage:Exenatide | 0 | | | 0.187333845 | ⊕⊕○○ Low | 0.187333845 | ⊕⊕○○ Low |
| 163 | Efpeglenatide_high_dosage:Inject_semaglutide_high_dosage | 0 | | | 0.330989319 | ⊕⊕○○ Low | 0.330989319 | ⊕⊕○○ Low |
| 164 | Efpeglenatide_high_dosage:Inject_semaglutide_low_dosage | 0 | | | 0.479986128 | ⊕⊕○○ Low | 0.479986128 | ⊕⊕○○ Low |
| 165 | Efpeglenatide_high_dosage:Inject_semaglutide_medium_dosage | 0 | | | 0.420712013 | ⊕⊕○○ Low | 0.420712013 | ⊕⊕○○ Low |
| 166 | Efpeglenatide_high_dosage:Liraglutide | 0 | | | 0.678699058 | ⊕⊕○○ Low | 0.678699058 | ⊕⊕○○ Low |
| 167 | Efpeglenatide_high_dosage:Lixisenatide | 0 | | | 0.165158416 | ⊕⊕○○ Low | 0.165158416 | ⊕⊕○○ Low |
| 168 | Efpeglenatide_high_dosage:Oral_semaglutide | 0 | | | -0.474891134 | ⊕⊕○○ Low | -0.474891134 | ⊕⊕○○ Low |
| 169 | Efpeglenatide_high_dosage:Placebo_or_Control | 1 | -0.013129382 | ⊕⊕⊕⊕ High | | | -0.013129382 | ⊕⊕⊕○ Medium |
| 170 | Efpeglenatide_high_dosage:Sotagliflozin | 0 | | | 0.073966083 | ⊕⊕○○ Low | 0.073966083 | ⊕⊕○○ Low |
| 171 | Efpeglenatide_high_dosage:Tirzepatide | 0 | | | 0.57157643 | ⊕⊕○○ Low | 0.57157643 | ⊕⊕○○ Low |
| 172 | Efpeglenatide_medium_dosage:Empagliflozin_high_dosage | 0 | | | 0.270872531 | ⊕⊕○○ Low | 0.270872531 | ⊕⊕○○ Low |
| 173 | Efpeglenatide_medium_dosage:Empagliflozin_low_dosage | 0 | | | 0.101144168 | ⊕⊕○○ Low | 0.101144168 | ⊕⊕○○ Low |

| | | | | | | | | |
|---|---|---|---|---|---|---|---|---|
| 174 | Efpeglenatide_medium_dosage:Ertugliflozin_high_dosage | 0 | | | 0.044764432 | ⊕⊕○○ Low | 0.044764432 | ⊕⊕○○ Low |
| 175 | Efpeglenatide_medium_dosage:Ertugliflozin_low_dosage | 0 | | | 0.15279678 | ⊕⊕○○ Low | 0.15279678 | ⊕⊕○○ Low |
| 176 | Efpeglenatide_medium_dosage:Exenatide | 0 | | | 0.167506402 | ⊕⊕○○ Low | 0.167506402 | ⊕⊕○○ Low |
| 177 | Efpeglenatide_medium_dosage:Inject_semaglutide_high_dosage | 0 | | | 0.311161875 | ⊕⊕○○ Low | 0.311161875 | ⊕⊕○○ Low |
| 178 | Efpeglenatide_medium_dosage:Inject_semaglutide_low_dosage | 0 | | | 0.460158684 | ⊕⊕○○ Low | 0.460158684 | ⊕⊕○○ Low |
| 179 | Efpeglenatide_medium_dosage:Inject_semaglutide_medium_dosage | 0 | | | 0.400884569 | ⊕⊕○○ Low | 0.400884569 | ⊕⊕○○ Low |
| 180 | Efpeglenatide_medium_dosage:Liraglutide | 0 | | | 0.658871614 | ⊕⊕○○ Low | 0.658871614 | ⊕⊕○○ Low |
| 181 | Efpeglenatide_medium_dosage:Lixisenatide | 0 | | | 0.145330972 | ⊕⊕○○ Low | 0.145330972 | ⊕⊕○○ Low |
| 182 | Efpeglenatide_medium_dosage:Oral_semaglutide | 0 | | | -0.494718578 | ⊕⊕○○ Low | -0.494718578 | ⊕⊕○○ Low |
| 183 | Efpeglenatide_medium_dosage:Placebo_or_Control | 1 | -0.032956825 | ⊕⊕⊕⊕ High | | | -0.032956825 | ⊕⊕⊕○ Medium |
| 184 | Efpeglenatide_medium_dosage:Sotagliflozin | 0 | | | 0.05413864 | ⊕⊕○○ Low | 0.05413864 | ⊕⊕○○ Low |
| 185 | Efpeglenatide_medium_dosage:Tirzepatide | 0 | | | 0.551748987 | ⊕⊕○○ Low | 0.551748987 | ⊕⊕○○ Low |
| 186 | Empagliflozin_high_dosage:Empagliflozin_low_dosage | 4 | -0.144660927 | ⊕⊕⊕⊕ High | -0.231308844 | ⊕⊕⊕⊕ High | -0.169728363 | ⊕⊕⊕⊕ High |
| 187 | Empagliflozin_high_dosage:Ertugliflozin_high_dosage | 0 | | | -0.2261081 | ⊕⊕○○ Low | -0.2261081 | ⊕⊕○○ Low |
| 188 | Empagliflozin_high_dosage:Ertugliflozin_low_dosage | 0 | | | -0.118075751 | ⊕⊕○○ Low | -0.118075751 | ⊕⊕○○ Low |
| 189 | Empagliflozin_high_dosage:Exenatide | 0 | | | -0.10336613 | ⊕⊕○○ Low | -0.10336613 | ⊕⊕○○ Low |
| 190 | Empagliflozin_high_dosage:Inject_semaglutide_high_dosage | 0 | | | 0.040289344 | ⊕⊕○○ Low | 0.040289344 | ⊕⊕○○ Low |
| 191 | Empagliflozin_high_dosage:Inject_semaglutide_low_dosage | 0 | | | 0.189286153 | ⊕⊕○○ Low | 0.189286153 | ⊕⊕○○ Low |
| 192 | Empagliflozin_high_dosage:Inject_semaglutide_medium_dosage | 0 | | | 0.130012038 | ⊕⊕○○ Low | 0.130012038 | ⊕⊕○○ Low |
| 193 | Empagliflozin_high_dosage:Liraglutide | 0 | | | 0.387999083 | ⊕⊕○○ Low | 0.387999083 | ⊕⊕○○ Low |
| 194 | Empagliflozin_high_dosage:Lixisenatide | 0 | | | -0.125541559 | ⊕⊕○○ Low | -0.125541559 | ⊕⊕○○ Low |
| 195 | Empagliflozin_high_dosage:Oral_semaglutide | 0 | | | -0.765591109 | ⊕⊕○○ Low | -0.765591109 | ⊕⊕○○ Low |

| | | | | | | | | |
|---|---|---|---|---|---|---|---|---|
| 196 | Empagliflozin_high_dosage:Placebo_or_Control | 5 | -0.334989889 | ⊕⊕⊕⊕ High | -0.072958996 | ⊕⊕⊕⊕ High | -0.303829357 | ⊕⊕⊕⊕ High |
| 197 | Empagliflozin_high_dosage:Sotagliflozin | 0 | | | -0.216733892 | ⊕⊕○○ Low | -0.216733892 | ⊕⊕○○ Low |
| 198 | Empagliflozin_high_dosage:Tirzepatide | 0 | | | 0.280876455 | ⊕⊕○○ Low | 0.280876455 | ⊕⊕○○ Low |
| 199 | Empagliflozin_low_dosage:Ertugliflozin_high_dosage | 0 | | | -0.056379737 | ⊕⊕○○ Low | -0.056379737 | ⊕⊕○○ Low |
| 200 | Empagliflozin_low_dosage:Ertugliflozin_low_dosage | 0 | | | 0.051652612 | ⊕⊕○○ Low | 0.051652612 | ⊕⊕○○ Low |
| 201 | Empagliflozin_low_dosage:Exenatide | 0 | | | 0.066362234 | ⊕⊕○○ Low | 0.066362234 | ⊕⊕○○ Low |
| 202 | Empagliflozin_low_dosage:Inject_semaglutide_high_dosage | 0 | | | 0.210017707 | ⊕⊕○○ Low | 0.210017707 | ⊕⊕○○ Low |
| 203 | Empagliflozin_low_dosage:Inject_semaglutide_low_dosage | 0 | | | 0.359014516 | ⊕⊕○○ Low | 0.359014516 | ⊕⊕○○ Low |
| 204 | Empagliflozin_low_dosage:Inject_semaglutide_medium_dosage | 0 | | | 0.299740401 | ⊕⊕○○ Low | 0.299740401 | ⊕⊕○○ Low |
| 205 | Empagliflozin_low_dosage:Liraglutide | 0 | | | 0.557727446 | ⊕⊕○○ Low | 0.557727446 | ⊕⊕○○ Low |
| 206 | Empagliflozin_low_dosage:Lixisenatide | 0 | | | 0.044186804 | ⊕⊕○○ Low | 0.044186804 | ⊕⊕○○ Low |
| 207 | Empagliflozin_low_dosage:Oral_semaglutide | 0 | | | -0.595862746 | ⊕⊕○○ Low | -0.595862746 | ⊕⊕○○ Low |
| 208 | Empagliflozin_low_dosage:Placebo_or_Control | 7 | -0.149181969 | ⊕⊕⊕⊕ High | 0.26090661 | ⊕⊕⊕○ Medium | -0.134100994 | ⊕⊕⊕⊕ High |
| 209 | Empagliflozin_low_dosage:Sotagliflozin | 0 | | | -0.047005528 | ⊕⊕○○ Low | -0.047005528 | ⊕⊕○○ Low |
| 210 | Empagliflozin_low_dosage:Tirzepatide | 0 | | | 0.450604819 | ⊕⊕○○ Low | 0.450604819 | ⊕⊕○○ Low |
| 211 | Ertugliflozin_high_dosage:Ertugliflozin_low_dosage | 3 | 0.110893931 | ⊕⊕⊕⊕ High | | | 0.108032348 | ⊕⊕⊕○ Medium |
| 212 | Ertugliflozin_high_dosage:Exenatide | 0 | | | 0.12274197 | ⊕⊕○○ Low | 0.12274197 | ⊕⊕○○ Low |
| 213 | Ertugliflozin_high_dosage:Inject_semaglutide_high_dosage | 0 | | | 0.266397444 | ⊕⊕○○ Low | 0.266397444 | ⊕⊕○○ Low |
| 214 | Ertugliflozin_high_dosage:Inject_semaglutide_low_dosage | 0 | | | 0.415394253 | ⊕⊕○○ Low | 0.415394253 | ⊕⊕○○ Low |
| 215 | Ertugliflozin_high_dosage:Inject_semaglutide_medium_dosage | 0 | | | 0.356120138 | ⊕⊕○○ Low | 0.356120138 | ⊕⊕○○ Low |
| 216 | Ertugliflozin_high_dosage:Liraglutide | 0 | | | 0.614107183 | ⊕⊕○○ Low | 0.614107183 | ⊕⊕○○ Low |
| 217 | Ertugliflozin_high_dosage:Lixisenatide | 0 | | | 0.100566541 | ⊕⊕○○ Low | 0.100566541 | ⊕⊕○○ Low |

| | | | | | | | | |
|---|---|---|---|---|---|---|---|---|
| 218 | Ertugliflozin_high_dosage:Oral_semaglutide | 0 | | | -0.539483009 | ⊕⊕○○ Low | -0.539483009 | ⊕⊕○○ Low |
| 219 | Ertugliflozin_high_dosage:Placebo_or_Control | 3 | -0.072871969 | ⊕⊕⊕⊕ High | -5.99113997 | ⊕⊕⊕⊕ High | -0.077721257 | ⊕⊕⊕⊕ High |
| 220 | Ertugliflozin_high_dosage:Sotagliflozin | 0 | | | 0.009374208 | ⊕⊕○○ Low | 0.009374208 | ⊕⊕○○ Low |
| 221 | Ertugliflozin_high_dosage:Tirzepatide | 0 | | | 0.506984555 | ⊕⊕○○ Low | 0.506984555 | ⊕⊕○○ Low |
| 222 | Ertugliflozin_low_dosage:Exenatide | 0 | | | 0.014709622 | ⊕⊕○○ Low | 0.014709622 | ⊕⊕○○ Low |
| 223 | Ertugliflozin_low_dosage:Inject_semaglutide_high_dosage | 0 | | | 0.158365095 | ⊕⊕○○ Low | 0.158365095 | ⊕⊕○○ Low |
| 224 | Ertugliflozin_low_dosage:Inject_semaglutide_low_dosage | 0 | | | 0.307361904 | ⊕⊕○○ Low | 0.307361904 | ⊕⊕○○ Low |
| 225 | Ertugliflozin_low_dosage:Inject_semaglutide_medium_dosage | 0 | | | 0.248087789 | ⊕⊕○○ Low | 0.248087789 | ⊕⊕○○ Low |
| 226 | Ertugliflozin_low_dosage:Liraglutide | 0 | | | 0.506074834 | ⊕⊕○○ Low | 0.506074834 | ⊕⊕○○ Low |
| 227 | Ertugliflozin_low_dosage:Lixisenatide | 0 | | | -0.007465808 | ⊕⊕○○ Low | -0.007465808 | ⊕⊕○○ Low |
| 228 | Ertugliflozin_low_dosage:Oral_semaglutide | 0 | | | -0.647515358 | ⊕⊕○○ Low | -0.647515358 | ⊕⊕○○ Low |
| 229 | Ertugliflozin_low_dosage:Placebo_or_Control | 3 | -0.18288228 | ⊕⊕⊕⊕ High | -1.999705135 | ⊕⊕⊕⊕ High | -0.185753605 | ⊕⊕⊕⊕ High |
| 230 | Ertugliflozin_low_dosage:Sotagliflozin | 0 | | | -0.09865814 | ⊕⊕○○ Low | -0.09865814 | ⊕⊕○○ Low |
| 231 | Ertugliflozin_low_dosage:Tirzepatide | 0 | | | 0.398952207 | ⊕⊕○○ Low | 0.398952207 | ⊕⊕○○ Low |
| 232 | Exenatide:Inject_semaglutide_high_dosage | 0 | | | 0.143655473 | ⊕⊕○○ Low | 0.143655473 | ⊕⊕○○ Low |
| 233 | Exenatide:Inject_semaglutide_low_dosage | 0 | | | 0.292652283 | ⊕⊕○○ Low | 0.292652283 | ⊕⊕○○ Low |
| 234 | Exenatide:Inject_semaglutide_medium_dosage | 0 | | | 0.233378167 | ⊕⊕○○ Low | 0.233378167 | ⊕⊕○○ Low |
| 235 | Exenatide:Liraglutide | 1 | 0.387314009 | ⊕⊕⊕⊕ High | 0.566854883 | ⊕⊕⊕⊕ High | 0.491365213 | ⊕⊕⊕⊕ High |
| 236 | Exenatide:Lixisenatide | 0 | | | -0.02217543 | ⊕⊕○○ Low | -0.02217543 | ⊕⊕○○ Low |
| 237 | Exenatide:Oral_semaglutide | 0 | | | -0.662224979 | ⊕⊕○○ Low | -0.662224979 | ⊕⊕○○ Low |
| 238 | Exenatide:Placebo_or_Control | 1 | -0.145529276 | ⊕⊕⊕⊕ High | -0.32507015 | ⊕⊕⊕⊕ High | -0.200463227 | ⊕⊕⊕⊕ High |
| 239 | Exenatide:Sotagliflozin | 0 | | | -0.113367762 | ⊕⊕○○ Low | -0.113367762 | ⊕⊕○○ Low |

| | | | | | | | | |
|---|---|---|---|---|---|---|---|---|
| 240 | Exenatide:Tirzepatide | 0 | | | 0.384242585 | ⊕⊕○○ Low | 0.384242585 | ⊕⊕○○ Low |
| 241 | Inject_semaglutide_high_dosage:Inject_semaglutide_low_dosage | 0 | | | 0.148996809 | ⊕⊕○○ Low | 0.148996809 | ⊕⊕○○ Low |
| 242 | Inject_semaglutide_high_dosage:Inject_semaglutide_medium_dosage | 1 | -0.002560821 | ⊕⊕⊕⊕ High | 0.127735235 | ⊕⊕⊕○ Medium | 0.089722694 | ⊕⊕⊕⊕ High |
| 243 | Inject_semaglutide_high_dosage:Liraglutide | 0 | | | 0.347709739 | ⊕⊕○○ Low | 0.347709739 | ⊕⊕○○ Low |
| 244 | Inject_semaglutide_high_dosage:Lixisenatide | 0 | | | -0.165830903 | ⊕⊕○○ Low | -0.165830903 | ⊕⊕○○ Low |
| 245 | Inject_semaglutide_high_dosage:Oral_semaglutide | 0 | | | -0.805880453 | ⊕⊕○○ Low | -0.805880453 | ⊕⊕○○ Low |
| 246 | Inject_semaglutide_high_dosage:Placebo_or_Control | 4 | -0.341954133 | ⊕⊕⊕⊕ High | -0.445058393 | ⊕⊕⊕⊕ High | -0.344118701 | ⊕⊕⊕⊕ High |
| 247 | Inject_semaglutide_high_dosage:Sotagliflozin | 0 | | | -0.257023235 | ⊕⊕○○ Low | -0.257023235 | ⊕⊕○○ Low |
| 248 | Inject_semaglutide_high_dosage:Tirzepatide | 0 | | | 0.240587112 | ⊕⊕○○ Low | 0.240587112 | ⊕⊕○○ Low |
| 249 | Inject_semaglutide_low_dosage:Inject_semaglutide_medium_dosage | 2 | -0.063383938 | ⊕⊕⊕⊕ High | -0.041430685 | ⊕⊕⊕⊕ High | -0.059274115 | ⊕⊕⊕⊕ High |
| 250 | Inject_semaglutide_low_dosage:Liraglutide | 0 | | | 0.19871293 | ⊕⊕○○ Low | 0.19871293 | ⊕⊕○○ Low |
| 251 | Inject_semaglutide_low_dosage:Lixisenatide | 0 | | | -0.314827712 | ⊕⊕○○ Low | -0.314827712 | ⊕⊕○○ Low |
| 252 | Inject_semaglutide_low_dosage:Oral_semaglutide | 0 | | | -0.954877262 | ⊕⊕○○ Low | -0.954877262 | ⊕⊕○○ Low |
| 253 | Inject_semaglutide_low_dosage:Placebo_or_Control | 2 | -0.462250586 | ⊕⊕⊕⊕ High | -0.750429108 | ⊕⊕⊕⊕ High | -0.49311551 | ⊕⊕⊕⊕ High |
| 254 | Inject_semaglutide_low_dosage:Sotagliflozin | 0 | | | -0.406020044 | ⊕⊕○○ Low | -0.406020044 | ⊕⊕○○ Low |
| 255 | Inject_semaglutide_low_dosage:Tirzepatide | 0 | | | 0.091590303 | ⊕⊕○○ Low | 0.091590303 | ⊕⊕○○ Low |
| 256 | Inject_semaglutide_medium_dosage:Liraglutide | 0 | | | 0.257987045 | ⊕⊕○○ Low | 0.257987045 | ⊕⊕○○ Low |
| 257 | Inject_semaglutide_medium_dosage:Lixisenatide | 0 | | | -0.255553597 | ⊕⊕○○ Low | -0.255553597 | ⊕⊕○○ Low |
| 258 | Inject_semaglutide_medium_dosage:Oral_semaglutide | 0 | | | -0.895603147 | ⊕⊕○○ Low | -0.895603147 | ⊕⊕○○ Low |
| 259 | Inject_semaglutide_medium_dosage:Placebo_or_Control | 3 | -0.459685667 | ⊕⊕⊕⊕ High | -0.39165066 | ⊕⊕⊕⊕ High | -0.433841395 | ⊕⊕⊕⊕ High |
| 260 | Inject_semaglutide_medium_dosage:Sotagliflozin | 0 | | | -0.346745929 | ⊕⊕○○ Low | -0.346745929 | ⊕⊕○○ Low |
| 261 | Inject_semaglutide_medium_dosage:Tirzepatide | 1 | 0.116456419 | ⊕⊕⊕⊕ High | 0.178646213 | ⊕⊕⊕⊕ High | 0.150864418 | ⊕⊕⊕⊕ High |

| | | | | | | | | |
|---|---|---|---|---|---|---|---|---|
| 262 | Liraglutide:Lixisenatide | 1 | -0.092260733 | ⊕⊕⊕⊕ High | -0.682291934 | ⊕⊕⊕⊕ High | -0.513540642 | ⊕⊕⊕⊕ High |
| 263 | Liraglutide:Oral_semaglutide | 0 | | | -1.153590192 | ⊕⊕○○ Low | -1.153590192 | ⊕⊕○○ Low |
| 264 | Liraglutide:Placebo_or_Control | 3 | -0.75812291 | ⊕⊕⊕⊕ High | -0.389956771 | ⊕⊕⊕⊕ High | -0.69182844 | ⊕⊕⊕⊕ High |
| 265 | Liraglutide:Sotagliflozin | 0 | | | -0.604732974 | ⊕⊕○○ Low | -0.604732974 | ⊕⊕○○ Low |
| 266 | Liraglutide:Tirzepatide | 0 | | | -0.107122628 | ⊕⊕○○ Low | -0.107122628 | ⊕⊕○○ Low |
| 267 | Lixisenatide:Oral_semaglutide | 0 | | | -0.64004955 | ⊕⊕○○ Low | -0.64004955 | ⊕⊕○○ Low |
| 268 | Lixisenatide:Placebo_or_Control | 1 | -0.048228542 | ⊕⊕⊕⊕ High | -0.638259743 | ⊕⊕⊕⊕ High | -0.178287798 | ⊕⊕⊕⊕ High |
| 269 | Lixisenatide:Sotagliflozin | 0 | | | -0.091192332 | ⊕⊕○○ Low | -0.091192332 | ⊕⊕○○ Low |
| 270 | Lixisenatide:Tirzepatide | 0 | | | 0.406418015 | ⊕⊕○○ Low | 0.406418015 | ⊕⊕○○ Low |
| 271 | Oral_semaglutide:Placebo_or_Control | 2 | 0.461761752 | ⊕⊕⊕⊕ High | | | 0.461761752 | ⊕⊕⊕○ Medium |
| 272 | Oral_semaglutide:Sotagliflozin | 0 | | | 0.548857217 | ⊕⊕○○ Low | 0.548857217 | ⊕⊕○○ Low |
| 273 | Oral_semaglutide:Tirzepatide | 0 | | | 1.046467564 | ⊕⊕○○ Low | 1.046467564 | ⊕⊕○○ Low |
| 274 | Sotagliflozin:Placebo_or_Control | 4 | -0.087095465 | ⊕⊕⊕⊕ High | | | -0.087095465 | ⊕⊕⊕○ Medium |
| 275 | Tirzepatide:Placebo_or_Control | 4 | -0.592206827 | ⊕⊕⊕⊕ High | -0.530017033 | ⊕⊕⊕⊕ High | -0.584705812 | ⊕⊕⊕⊕ High |
| 276 | Sotagliflozin:Tirzepatide | 0 | | | 0.497610347 | ⊕⊕○○ Low | 0.497610347 | ⊕⊕○○ Low |

*Abbreviation: 95%CIs: 95% confidence intervals; GLP-1 agonist: glucagon-like peptide-1 agonist; NA: not applicable; NMA: network meta-analysis; OR: odds ratio; RCT: randomized controlled trial; SGLT2 inhibitor: sodium–glucose cotransporter 2 inhibitor*

## Reference list of supplement tables:


1. Page, M.J.; McKenzie, J.E.; Bossuyt, P.M.; Boutron, I.; Hoffmann, T.C.; Mulrow, C.D.; Shamseer, L.; Tetzlaff, J.M.; Akl, E.A.; Brennan, S.E.; et al. The PRISMA 2020 statement: an updated guideline for reporting systematic reviews. *Bmj* **2021**, *372*, n71, doi:10.1136/bmj.n71.
2. Mattii, L.; Moscato, S.; Ippolito, C.; Polizzi, E.; Novo, G.; Zucchi, R.; De Caterina, R.; Ghelardoni, S.; Madonna, R. Empagliflozin mitigates ponatinib-induced cardiotoxicity by restoring the connexin 43-autophagy pathway. *Biomed Pharmacother* **2024**, *178*, 117278, doi:10.1016/j.biopha.2024.117278.
3. Leiter, L.A.; Cefalu, W.T.; de Bruin, T.W.; Xu, J.; Parikh, S.; Johnsson, E.; Gause-Nilsson, I. Long-term maintenance of efficacy of dapagliflozin in patients with type 2 diabetes mellitus and cardiovascular disease. *Diabetes Obes Metab* **2016**, *18*, 766-774, doi:10.1111/dom.12666.
4. Nagendra, L.; Bg, H.; Sharma, M.; Dutta, D. Semaglutide and cancer: A systematic review and meta-analysis. *Diabetes Metab Syndr* **2023**, *17*, 102834, doi:10.1016/j.dsx.2023.102834.
5. Zhang, L.; Xue, B.; Yu, F.; Yin, Y.; Jin, S. Deciphering the Causal Relationship between Sodium-glucose Cotransporter 2 Inhibition and Cancer Risks: A Comprehensive Mendelian Randomization Study. *J Cancer* **2024**, *15*, 3903-3912, doi:10.7150/jca.96435.
6. Guo, W.; Zhao, L.; Huang, W.; Chen, J.; Zhong, T.; Yan, S.; Hu, W.; Zeng, F.; Peng, C.; Yan, H. Sodium-glucose cotransporter 2 inhibitors, inflammation, and heart failure: a two-sample Mendelian randomization study. *Cardiovasc Diabetol* **2024**, *23*, 118, doi:10.1186/s12933-024-02210-5.
7. Roden, M.; Weng, J.; Eilbracht, J.; Delafont, B.; Kim, G.; Woerle, H.J.; Broedl, U.C.; investigators, E.-R.M.t. Empagliflozin monotherapy with sitagliptin as an active comparator in patients with type 2 diabetes: a randomised, double-blind, placebo-controlled, phase 3 trial. *Lancet Diabetes Endocrinol* **2013**, *1*, 208-219, doi:10.1016/S2213-8587(13)70084-6.
8. Polidori, D.; Mari, A.; Ferrannini, E. Canagliflozin, a sodium glucose co-transporter 2 inhibitor, improves model-based indices of beta cell

function in patients with type 2 diabetes. *Diabetologia* **2014**, *57*, 891-901, doi:10.1007/s00125-014-3196-x.

9. Stenlof, K.; Cefalu, W.T.; Kim, K.A.; Jodar, E.; Alba, M.; Edwards, R.; Tong, C.; Canovatchel, W.; Meininger, G. Long-term efficacy and safety of canagliflozin monotherapy in patients with type 2 diabetes inadequately controlled with diet and exercise: findings from the 52-week CANTATA-M study. *Curr Med Res Opin* **2014**, *30*, 163-175, doi:10.1185/03007995.2013.850066.
10. Lavalle-Gonzalez, F.J.; Januszewicz, A.; Davidson, J.; Tong, C.; Qiu, R.; Canovatchel, W.; Meininger, G. Efficacy and safety of canagliflozin compared with placebo and sitagliptin in patients with type 2 diabetes on background metformin monotherapy: a randomised trial. *Diabetologia* **2013**, *56*, 2582-2592, doi:10.1007/s00125-013-3039-1.
11. Rosenstock, J.; Raccah, D.; Koranyi, L.; Maffei, L.; Boka, G.; Miossec, P.; Gerich, J.E. Efficacy and safety of lixisenatide once daily versus exenatide twice daily in type 2 diabetes inadequately controlled on metformin: a 24-week, randomized, open-label, active-controlled study (GetGoal-X). *Diabetes Care* **2013**, *36*, 2945-2951, doi:10.2337/dc12-2709.
12. Charbonnel, B.; Steinberg, H.; Eymard, E.; Xu, L.; Thakkar, P.; Prabhu, V.; Davies, M.J.; Engel, S.S. Efficacy and safety over 26 weeks of an oral treatment strategy including sitagliptin compared with an injectable treatment strategy with liraglutide in patients with type 2 diabetes mellitus inadequately controlled on metformin: a randomised clinical trial. *Diabetologia* **2013**, *56*, 1503-1511, doi:10.1007/s00125-013-2905-1.
13. Schernthaner, G.; Gross, J.L.; Rosenstock, J.; Guarisco, M.; Fu, M.; Yee, J.; Kawaguchi, M.; Canovatchel, W.; Meininger, G. Canagliflozin compared with sitagliptin for patients with type 2 diabetes who do not have adequate glycemic control with metformin plus sulfonylurea: a 52-week randomized trial. *Diabetes Care* **2013**, *36*, 2508-2515, doi:10.2337/dc12-2491.
14. Gallwitz, B.; Guzman, J.; Dotta, F.; Guerci, B.; Simo, R.; Basson, B.R.; Festa, A.; Kiljanski, J.; Sapin, H.; Trautmann, M.; et al. Exenatide twice daily versus glimepiride for prevention of glycaemic deterioration in patients with type 2 diabetes with metformin failure (EUREXA): an open-label, randomised controlled trial. *Lancet* **2012**, *379*, 2270-2278, doi:10.1016/S0140-6736(12)60479-6.
15. Gallwitz, B.; Bohmer, M.; Segiet, T.; Molle, A.; Milek, K.; Becker, B.; Helsberg, K.; Petto, H.; Peters, N.; Bachmann, O. Exenatide twice daily versus premixed insulin aspart 70/30 in metformin-treated patients with type 2 diabetes: a randomized 26-week study on glycemic control and hypoglycemia. *Diabetes Care* **2011**, *34*, 604-606, doi:10.2337/dc10-1900.

16. Garber, A.; Henry, R.; Ratner, R.; Garcia-Hernandez, P.A.; Rodriguez-Pattzi, H.; Olvera-Alvarez, I.; Hale, P.M.; Zdravkovic, M.; Bode, B.; Group, L.-S. Liraglutide versus glimepiride monotherapy for type 2 diabetes (LEAD-3 Mono): a randomised, 52-week, phase III, double-blind, parallel-treatment trial. *Lancet* **2009**, *373*, 473-481, doi:10.1016/S0140-6736(08)61246-5.
17. Davies, M.J.; Bergenstal, R.; Bode, B.; Kushner, R.F.; Lewin, A.; Skjoth, T.V.; Andreasen, A.H.; Jensen, C.B.; DeFronzo, R.A.; Group, N.N.S. Efficacy of Liraglutide for Weight Loss Among Patients With Type 2 Diabetes: The SCALE Diabetes Randomized Clinical Trial. *Jama* **2015**, *314*, 687-699, doi:10.1001/jama.2015.9676.
18. Mathieu, C.; Ranetti, A.E.; Li, D.; Ekholm, E.; Cook, W.; Hirshberg, B.; Chen, H.; Hansen, L.; Iqbal, N. Randomized, Double-Blind, Phase 3 Trial of Triple Therapy With Dapagliflozin Add-on to Saxagliptin Plus Metformin in Type 2 Diabetes. *Diabetes Care* **2015**, *38*, 2009-2017, doi:10.2337/dc15-0779.
19. Kovacs, C.S.; Seshiah, V.; Merker, L.; Christiansen, A.V.; Roux, F.; Salsali, A.; Kim, G.; Stella, P.; Woerle, H.J.; Broedl, U.C.; et al. Empagliflozin as Add-on Therapy to Pioglitazone With or Without Metformin in Patients With Type 2 Diabetes Mellitus. *Clin Ther* **2015**, *37*, 1773-1788 e1771, doi:10.1016/j.clinthera.2015.05.511.
20. Blonde, L.; Jendle, J.; Gross, J.; Woo, V.; Jiang, H.; Fahrbach, J.L.; Milicevic, Z. Once-weekly dulaglutide versus bedtime insulin glargine, both in combination with prandial insulin lispro, in patients with type 2 diabetes (AWARD-4): a randomised, open-label, phase 3, non-inferiority study. *Lancet* **2015**, *385*, 2057-2066, doi:10.1016/S0140-6736(15)60936-9.
21. Weinstock, R.S.; Guerci, B.; Umpierrez, G.; Nauck, M.A.; Skrivanek, Z.; Milicevic, Z. Safety and efficacy of once-weekly dulaglutide versus sitagliptin after 2 years in metformin-treated patients with type 2 diabetes (AWARD-5): a randomized, phase III study. *Diabetes Obes Metab* **2015**, *17*, 849-858, doi:10.1111/dom.12479.
22. Cefalu, W.T.; Leiter, L.A.; de Bruin, T.W.; Gause-Nilsson, I.; Sugg, J.; Parikh, S.J. Dapagliflozin's Effects on Glycemia and Cardiovascular Risk Factors in High-Risk Patients With Type 2 Diabetes: A 24-Week, Multicenter, Randomized, Double-Blind, Placebo-Controlled Study With a 28-Week Extension. *Diabetes Care* **2015**, *38*, 1218-1227, doi:10.2337/dc14-0315.
23. Dungan, K.M.; Povedano, S.T.; Forst, T.; Gonzalez, J.G.; Atisso, C.; Sealls, W.; Fahrbach, J.L. Once-weekly dulaglutide versus once-daily liraglutide in metformin-treated patients with type 2 diabetes (AWARD-6): a randomised, open-label, phase 3, non-inferiority trial.

*Lancet* **2014**, *384*, 1349-1357, doi:10.1016/S0140-6736(14)60976-4.

24. Wysham, C.; Blevins, T.; Arakaki, R.; Colon, G.; Garcia, P.; Atisso, C.; Kuhstoss, D.; Lakshmanan, M. Efficacy and safety of dulaglutide added onto pioglitazone and metformin versus exenatide in type 2 diabetes in a randomized controlled trial (AWARD-1). *Diabetes Care* **2014**, *37*, 2159-2167, doi:10.2337/dc13-2760.
25. Barnett, A.H.; Mithal, A.; Manassie, J.; Jones, R.; Rattunde, H.; Woerle, H.J.; Broedl, U.C.; investigators, E.-R.R.t. Efficacy and safety of empagliflozin added to existing antidiabetes treatment in patients with type 2 diabetes and chronic kidney disease: a randomised, double-blind, placebo-controlled trial. *Lancet Diabetes Endocrinol* **2014**, *2*, 369-384, doi:10.1016/S2213-8587(13)70208-0.
26. Pratley, R.E.; Nauck, M.A.; Barnett, A.H.; Feinglos, M.N.; Ovalle, F.; Harman-Boehm, I.; Ye, J.; Scott, R.; Johnson, S.; Stewart, M.; et al. Once-weekly albiglutide versus once-daily liraglutide in patients with type 2 diabetes inadequately controlled on oral drugs (HARMONY 7): a randomised, open-label, multicentre, non-inferiority phase 3 study. *Lancet Diabetes Endocrinol* **2014**, *2*, 289-297, doi:10.1016/S2213-8587(13)70214-6.
27. Home, P.D.; Ahren, B.; Reusch, J.E.B.; Rendell, M.; Weissman, P.N.; Cirkel, D.T.; Miller, D.; Ambery, P.; Carr, M.C.; Nauck, M.A. Three-year data from 5 HARMONY phase 3 clinical trials of albiglutide in type 2 diabetes mellitus: Long-term efficacy with or without rescue therapy. *Diabetes Res Clin Pract* **2017**, *131*, 49-60, doi:10.1016/j.diabres.2017.06.013.
28. Januzzi, J.L., Jr.; Butler, J.; Jarolim, P.; Sattar, N.; Vijapurkar, U.; Desai, M.; Davies, M.J. Effects of Canagliflozin on Cardiovascular Biomarkers in Older Adults With Type 2 Diabetes. *J Am Coll Cardiol* **2017**, *70*, 704-712, doi:10.1016/j.jacc.2017.06.016.
29. Ahren, B.; Masmiquel, L.; Kumar, H.; Sargin, M.; Karsbol, J.D.; Jacobsen, S.H.; Chow, F. Efficacy and safety of once-weekly semaglutide versus once-daily sitagliptin as an add-on to metformin, thiazolidinediones, or both, in patients with type 2 diabetes (SUSTAIN 2): a 56-week, double-blind, phase 3a, randomised trial. *Lancet Diabetes Endocrinol* **2017**, *5*, 341-354, doi:10.1016/S2213-8587(17)30092-X.
30. Aroda, V.R.; Bain, S.C.; Cariou, B.; Piletic, M.; Rose, L.; Axelsen, M.; Rowe, E.; DeVries, J.H. Efficacy and safety of once-weekly semaglutide versus once-daily insulin glargine as add-on to metformin (with or without sulfonylureas) in insulin-naive patients with type 2 diabetes (SUSTAIN 4): a randomised, open-label, parallel-group, multicentre, multinational, phase 3a trial. *Lancet Diabetes Endocrinol* **2017**, *5*, 355-366, doi:10.1016/S2213-8587(17)30085-2.

31. Meneilly, G.S.; Roy-Duval, C.; Alawi, H.; Dailey, G.; Bellido, D.; Trescoli, C.; Manrique Hurtado, H.; Guo, H.; Pilorget, V.; Perfetti, R.; et al. Lixisenatide Therapy in Older Patients With Type 2 Diabetes Inadequately Controlled on Their Current Antidiabetic Treatment: The GetGoal-O Randomized Trial. *Diabetes Care* **2017**, *40*, 485-493, doi:10.2337/dc16-2143.
32. Mellander, A.; Billger, M.; Johnsson, E.; Traff, A.K.; Yoshida, S.; Johnsson, K. Hypersensitivity Events, Including Potentially Hypersensitivity-Related Skin Events, with Dapagliflozin in Patients with Type 2 Diabetes Mellitus: A Pooled Analysis. *Clinical drug investigation* **2016**, *36*, 925-933, doi:10.1007/s40261-016-0438-3.
33. Investigators, F.-S.T. Glucose Variability in a 26-Week Randomized Comparison of Mealtime Treatment With Rapid-Acting Insulin Versus GLP-1 Agonist in Participants With Type 2 Diabetes at High Cardiovascular Risk. *Diabetes Care* **2016**, *39*, 973-981, doi:10.2337/dc15-2782.
34. Dungan, K.M.; Weitgasser, R.; Perez Manghi, F.; Pintilei, E.; Fahrbach, J.L.; Jiang, H.H.; Shell, J.; Robertson, K.E. A 24-week study to evaluate the efficacy and safety of once-weekly dulaglutide added on to glimepiride in type 2 diabetes (AWARD-8). *Diabetes Obes Metab* **2016**, *18*, 475-482, doi:10.1111/dom.12634.
35. Davies, M.J.; Bain, S.C.; Atkin, S.L.; Rossing, P.; Scott, D.; Shamkhalova, M.S.; Bosch-Traberg, H.; Syren, A.; Umpierrez, G.E. Efficacy and Safety of Liraglutide Versus Placebo as Add-on to Glucose-Lowering Therapy in Patients With Type 2 Diabetes and Moderate Renal Impairment (LIRA-RENAL): A Randomized Clinical Trial. *Diabetes Care* **2016**, *39*, 222-230, doi:10.2337/dc14-2883.
36. Coskun, T.; Sloop, K.W.; Loghin, C.; Alsina-Fernandez, J.; Urva, S.; Bokvist, K.B.; Cui, X.; Briere, D.A.; Cabrera, O.; Roell, W.C.; et al. LY3298176, a novel dual GIP and GLP-1 receptor agonist for the treatment of type 2 diabetes mellitus: From discovery to clinical proof of concept. *Mol Metab* **2018**, *18*, 3-14, doi:10.1016/j.molmet.2018.09.009.
37. Frias, J.P.; Nauck, M.A.; Van, J.; Kutner, M.E.; Cui, X.; Benson, C.; Urva, S.; Gimeno, R.E.; Milicevic, Z.; Robins, D.; et al. Efficacy and safety of LY3298176, a novel dual GIP and GLP-1 receptor agonist, in patients with type 2 diabetes: a randomised, placebo-controlled and active comparator-controlled phase 2 trial. *Lancet* **2018**, *392*, 2180-2193, doi:10.1016/S0140-6736(18)32260-8.
38. O'Neil, P.M.; Birkenfeld, A.L.; McGowan, B.; Mosenzon, O.; Pedersen, S.D.; Wharton, S.; Carson, C.G.; Jepsen, C.H.; Kabisch, M.; Wilding, J.P.H. Efficacy and safety of semaglutide compared with liraglutide and placebo for weight loss in patients with obesity: a randomised,

double-blind, placebo and active controlled, dose-ranging, phase 2 trial. *Lancet* **2018**, *392*, 637-649, doi:10.1016/S0140-6736(18)31773-2.
39. Buse, J.B.; Garg, S.K.; Rosenstock, J.; Bailey, T.S.; Banks, P.; Bode, B.W.; Danne, T.; Kushner, J.A.; Lane, W.S.; Lapuerta, P.; et al. Sotagliflozin in Combination With Optimized Insulin Therapy in Adults With Type 1 Diabetes: The North American inTandem1 Study. *Diabetes Care* **2018**, *41*, 1970-1980, doi:10.2337/dc18-0343.
40. Tuttle, K.R.; Lakshmanan, M.C.; Rayner, B.; Busch, R.S.; Zimmermann, A.G.; Woodward, D.B.; Botros, F.T. Dulaglutide versus insulin glargine in patients with type 2 diabetes and moderate-to-severe chronic kidney disease (AWARD-7): a multicentre, open-label, randomised trial. *Lancet Diabetes Endocrinol* **2018**, *6*, 605-617, doi:10.1016/S2213-8587(18)30104-9.
41. Ludvik, B.; Frias, J.P.; Tinahones, F.J.; Wainstein, J.; Jiang, H.; Robertson, K.E.; Garcia-Perez, L.E.; Woodward, D.B.; Milicevic, Z. Dulaglutide as add-on therapy to SGLT2 inhibitors in patients with inadequately controlled type 2 diabetes (AWARD-10): a 24-week, randomised, double-blind, placebo-controlled trial. *Lancet Diabetes Endocrinol* **2018**, *6*, 370-381, doi:10.1016/S2213-8587(18)30023-8.
42. Aronson, R.; Frias, J.; Goldman, A.; Darekar, A.; Lauring, B.; Terra, S.G. Long-term efficacy and safety of ertugliflozin monotherapy in patients with inadequately controlled T2DM despite diet and exercise: VERTIS MONO extension study. *Diabetes Obes Metab* **2018**, *20*, 1453-1460, doi:10.1111/dom.13251.
43. Pratley, R.E.; Aroda, V.R.; Lingvay, I.; Ludemann, J.; Andreassen, C.; Navarria, A.; Viljoen, A.; investigators, S. Semaglutide versus dulaglutide once weekly in patients with type 2 diabetes (SUSTAIN 7): a randomised, open-label, phase 3b trial. *Lancet Diabetes Endocrinol* **2018**, *6*, 275-286, doi:10.1016/S2213-8587(18)30024-X.
44. Ahmann, A.J.; Capehorn, M.; Charpentier, G.; Dotta, F.; Henkel, E.; Lingvay, I.; Holst, A.G.; Annett, M.P.; Aroda, V.R. Efficacy and Safety of Once-Weekly Semaglutide Versus Exenatide ER in Subjects With Type 2 Diabetes (SUSTAIN 3): A 56-Week, Open-Label, Randomized Clinical Trial. *Diabetes Care* **2018**, *41*, 258-266, doi:10.2337/dc17-0417.
45. Yu, M.; Brunt, K.V.; Milicevic, Z.; Varnado, O.; Boye, K.S. Patient-reported Outcomes in Patients with Type 2 Diabetes Treated with Dulaglutide Added to Titrated Insulin Glargine (AWARD-9). *Clin Ther* **2017**, *39*, 2284-2295, doi:10.1016/j.clinthera.2017.10.002.
46. Bhatt, D.L.; Szarek, M.; Steg, P.G.; Cannon, C.P.; Leiter, L.A.; McGuire, D.K.; Lewis, J.B.; Riddle, M.C.; Voors, A.A.; Metra, M.; et al.

Sotagliflozin in Patients with Diabetes and Recent Worsening Heart Failure. *N Engl J Med* **2021**, *384*, 117-128, doi:10.1056/NEJMoa2030183.
47. Stack, A.G.; Han, D.; Goldwater, R.; Johansson, S.; Dronamraju, N.; Oscarsson, J.; Johnsson, E.; Parkinson, J.; Erlandsson, F. Dapagliflozin Added to Verinurad Plus Febuxostat Further Reduces Serum Uric Acid in Hyperuricemia: The QUARTZ Study. *J Clin Endocrinol Metab* **2021**, *106*, e2347-e2356, doi:10.1210/clinem/dgaa748.
48. Wang, J.; Li, H.Q.; Xu, X.H.; Kong, X.C.; Sun, R.; Jing, T.; Ye, L.; Su, X.F.; Ma, J.H. The Effects of Once-Weekly Dulaglutide and Insulin Glargine on Glucose Fluctuation in Poorly Oral-Antidiabetic Controlled Patients with Type 2 Diabetes Mellitus. *Biomed Res Int* **2019**, *2019*, 2682657, doi:10.1155/2019/2682657.
49. Lingvay, I.; Catarig, A.M.; Frias, J.P.; Kumar, H.; Lausvig, N.L.; le Roux, C.W.; Thielke, D.; Viljoen, A.; McCrimmon, R.J. Efficacy and safety of once-weekly semaglutide versus daily canagliflozin as add-on to metformin in patients with type 2 diabetes (SUSTAIN 8): a double-blind, phase 3b, randomised controlled trial. *Lancet Diabetes Endocrinol* **2019**, *7*, 834-844, doi:10.1016/S2213-8587(19)30311-0.
50. Rodbard, H.W.; Rosenstock, J.; Canani, L.H.; Deerochanawong, C.; Gumprecht, J.; Lindberg, S.O.; Lingvay, I.; Sondergaard, A.L.; Treppendahl, M.B.; Montanya, E.; et al. Oral Semaglutide Versus Empagliflozin in Patients With Type 2 Diabetes Uncontrolled on Metformin: The PIONEER 2 Trial. *Diabetes Care* **2019**, *42*, 2272-2281, doi:10.2337/dc19-0883.
51. Mullins, R.J.; Mustapic, M.; Chia, C.W.; Carlson, O.; Gulyani, S.; Tran, J.; Li, Y.; Mattson, M.P.; Resnick, S.; Egan, J.M.; et al. A Pilot Study of Exenatide Actions in Alzheimer's Disease. *Curr Alzheimer Res* **2019**, *16*, 741-752, doi:10.2174/1567205016666190913155950.
52. Pieber, T.R.; Bode, B.; Mertens, A.; Cho, Y.M.; Christiansen, E.; Hertz, C.L.; Wallenstein, S.O.R.; Buse, J.B.; investigators, P. Efficacy and safety of oral semaglutide with flexible dose adjustment versus sitagliptin in type 2 diabetes (PIONEER 7): a multicentre, open-label, randomised, phase 3a trial. *Lancet Diabetes Endocrinol* **2019**, *7*, 528-539, doi:10.1016/S2213-8587(19)30194-9.
53. Mosenzon, O.; Blicher, T.M.; Rosenlund, S.; Eriksson, J.W.; Heller, S.; Hels, O.H.; Pratley, R.; Sathyapalan, T.; Desouza, C.; Investigators, P. Efficacy and safety of oral semaglutide in patients with type 2 diabetes and moderate renal impairment (PIONEER 5): a placebo-controlled, randomised, phase 3a trial. *Lancet Diabetes Endocrinol* **2019**, *7*, 515-527, doi:10.1016/S2213-8587(19)30192-5.
54. Aroda, V.R.; Rosenstock, J.; Terauchi, Y.; Altuntas, Y.; Lalic, N.M.; Morales Villegas, E.C.; Jeppesen, O.K.; Christiansen, E.; Hertz, C.L.;

Haluzik, M.; et al. PIONEER 1: Randomized Clinical Trial of the Efficacy and Safety of Oral Semaglutide Monotherapy in Comparison With Placebo in Patients With Type 2 Diabetes. *Diabetes Care* **2019**, *42*, 1724-1732, doi:10.2337/dc19-0749.
55. Pratley, R.; Amod, A.; Hoff, S.T.; Kadowaki, T.; Lingvay, I.; Nauck, M.; Pedersen, K.B.; Saugstrup, T.; Meier, J.J.; investigators, P. Oral semaglutide versus subcutaneous liraglutide and placebo in type 2 diabetes (PIONEER 4): a randomised, double-blind, phase 3a trial. *Lancet* **2019**, *394*, 39-50, doi:10.1016/S0140-6736(19)31271-1.
56. Voors, A.A.; Angermann, C.E.; Teerlink, J.R.; Collins, S.P.; Kosiborod, M.; Biegus, J.; Ferreira, J.P.; Nassif, M.E.; Psotka, M.A.; Tromp, J.; et al. The SGLT2 inhibitor empagliflozin in patients hospitalized for acute heart failure: a multinational randomized trial. *Nat Med* **2022**, *28*, 568-574, doi:10.1038/s41591-021-01659-1.
57. Spertus, J.A.; Birmingham, M.C.; Nassif, M.; Damaraju, C.V.; Abbate, A.; Butler, J.; Lanfear, D.E.; Lingvay, I.; Kosiborod, M.N.; Januzzi, J.L. The SGLT2 inhibitor canagliflozin in heart failure: the CHIEF-HF remote, patient-centered randomized trial. *Nat Med* **2022**, *28*, 809-813, doi:10.1038/s41591-022-01703-8.
58. Dahl, D.; Onishi, Y.; Norwood, P.; Huh, R.; Bray, R.; Patel, H.; Rodriguez, A. Effect of Subcutaneous Tirzepatide vs Placebo Added to Titrated Insulin Glargine on Glycemic Control in Patients With Type 2 Diabetes: The SURPASS-5 Randomized Clinical Trial. *Jama* **2022**, *327*, 534-545, doi:10.1001/jama.2022.0078.
59. Kadowaki, T.; Isendahl, J.; Khalid, U.; Lee, S.Y.; Nishida, T.; Ogawa, W.; Tobe, K.; Yamauchi, T.; Lim, S.; investigators, S. Semaglutide once a week in adults with overweight or obesity, with or without type 2 diabetes in an east Asian population (STEP 6): a randomised, double-blind, double-dummy, placebo-controlled, phase 3a trial. *Lancet Diabetes Endocrinol* **2022**, *10*, 193-206, doi:10.1016/S2213-8587(22)00008-0.
60. Rubino, D.M.; Greenway, F.L.; Khalid, U.; O'Neil, P.M.; Rosenstock, J.; Sorrig, R.; Wadden, T.A.; Wizert, A.; Garvey, W.T.; Investigators, S. Effect of Weekly Subcutaneous Semaglutide vs Daily Liraglutide on Body Weight in Adults With Overweight or Obesity Without Diabetes: The STEP 8 Randomized Clinical Trial. *Jama* **2022**, *327*, 138-150, doi:10.1001/jama.2021.23619.
61. Rodgers, M.; Migdal, A.L.; Rodriguez, T.G.; Chen, Z.Z.; Nath, A.K.; Gerszten, R.E.; Kasid, N.; Toschi, E.; Tripaldi, J.; Heineman, B.; et al. Weight Loss Outcomes Among Early High Responders to Exenatide Treatment: A Randomized, Placebo Controlled Study in Overweight

and Obese Women. *Front Endocrinol (Lausanne)* **2021**, *12*, 742873, doi:10.3389/fendo.2021.742873.
62. Ludvik, B.; Giorgino, F.; Jodar, E.; Frias, J.P.; Fernandez Lando, L.; Brown, K.; Bray, R.; Rodriguez, A. Once-weekly tirzepatide versus once-daily insulin degludec as add-on to metformin with or without SGLT2 inhibitors in patients with type 2 diabetes (SURPASS-3): a randomised, open-label, parallel-group, phase 3 trial. *Lancet* **2021**, *398*, 583-598, doi:10.1016/S0140-6736(21)01443-4.
63. Kosiborod, M.N.; Esterline, R.; Furtado, R.H.M.; Oscarsson, J.; Gasparyan, S.B.; Koch, G.G.; Martinez, F.; Mukhtar, O.; Verma, S.; Chopra, V.; et al. Dapagliflozin in patients with cardiometabolic risk factors hospitalised with COVID-19 (DARE-19): a randomised, double-blind, placebo-controlled, phase 3 trial. *Lancet Diabetes Endocrinol* **2021**, *9*, 586-594, doi:10.1016/S2213-8587(21)00180-7.
64. Rosenstock, J.; Wysham, C.; Frias, J.P.; Kaneko, S.; Lee, C.J.; Fernandez Lando, L.; Mao, H.; Cui, X.; Karanikas, C.A.; Thieu, V.T. Efficacy and safety of a novel dual GIP and GLP-1 receptor agonist tirzepatide in patients with type 2 diabetes (SURPASS-1): a double-blind, randomised, phase 3 trial. *Lancet* **2021**, *398*, 143-155, doi:10.1016/S0140-6736(21)01324-6.
65. Wadden, T.A.; Bailey, T.S.; Billings, L.K.; Davies, M.; Frias, J.P.; Koroleva, A.; Lingvay, I.; O'Neil, P.M.; Rubino, D.M.; Skovgaard, D.; et al. Effect of Subcutaneous Semaglutide vs Placebo as an Adjunct to Intensive Behavioral Therapy on Body Weight in Adults With Overweight or Obesity: The STEP 3 Randomized Clinical Trial. *Jama* **2021**, *325*, 1403-1413, doi:10.1001/jama.2021.1831.
66. Aroda, V.R.; Frias, J.P.; Ji, L.; Niemoeller, E.; Nguyen-Pascal, M.L.; Denkel, K.; Espinasse, M.; Guo, H.; Baek, S.; Choi, J.; et al. Efficacy and safety of once-weekly efpeglenatide in people with suboptimally controlled type 2 diabetes: The AMPLITUDE-D, AMPLITUDE-L and AMPLITUDE-S randomized controlled trials. *Diabetes Obes Metab* **2023**, *25*, 2084-2095, doi:10.1111/dom.15079.
67. Ji, L.; Lu, Y.; Li, Q.; Fu, L.; Luo, Y.; Lei, T.; Li, L.; Ye, S.; Shi, B.; Li, X.; et al. Efficacy and safety of empagliflozin in combination with insulin in Chinese patients with type 2 diabetes and insufficient glycaemic control: A phase III, randomized, double-blind, placebo-controlled, parallel study. *Diabetes Obes Metab* **2023**, *25*, 1839-1848, doi:10.1111/dom.15041.
68. Garvey, W.T.; Batterham, R.L.; Bhatta, M.; Buscemi, S.; Christensen, L.N.; Frias, J.P.; Jodar, E.; Kandler, K.; Rigas, G.; Wadden, T.A.; et al. Two-year effects of semaglutide in adults with overweight or obesity: the STEP 5 trial. *Nat Med* **2022**, *28*, 2083-2091, doi:10.1038/s41591-022-02026-4.
69. Inagaki, N.; Takeuchi, M.; Oura, T.; Imaoka, T.; Seino, Y. Efficacy and safety of tirzepatide monotherapy compared with dulaglutide in

Japanese patients with type 2 diabetes (SURPASS J-mono): a double-blind, multicentre, randomised, phase 3 trial. *Lancet Diabetes Endocrinol* **2022**, *10*, 623-633, doi:10.1016/S2213-8587(22)00188-7.
70. Kadowaki, T.; Chin, R.; Ozeki, A.; Imaoka, T.; Ogawa, Y. Safety and efficacy of tirzepatide as an add-on to single oral antihyperglycaemic medication in patients with type 2 diabetes in Japan (SURPASS J-combo): a multicentre, randomised, open-label, parallel-group, phase 3 trial. *Lancet Diabetes Endocrinol* **2022**, *10*, 634-644, doi:10.1016/S2213-8587(22)00187-5.
71. Frias, J.P.; Choi, J.; Rosenstock, J.; Popescu, L.; Niemoeller, E.; Muehlen-Bartmer, I.; Baek, S. Efficacy and Safety of Once-Weekly Efpeglenatide Monotherapy Versus Placebo in Type 2 Diabetes: The AMPLITUDE-M Randomized Controlled Trial. *Diabetes Care* **2022**, *45*, 1592-1600, doi:10.2337/dc21-2656.
72. Kellerer, M.; Kaltoft, M.S.; Lawson, J.; Nielsen, L.L.; Strojek, K.; Tabak, O.; Jacob, S. Effect of once-weekly semaglutide versus thrice-daily insulin aspart, both as add-on to metformin and optimized insulin glargine treatment in participants with type 2 diabetes (SUSTAIN 11): A randomized, open-label, multinational, phase 3b trial. *Diabetes Obes Metab* **2022**, *24*, 1788-1799, doi:10.1111/dom.14765.
73. Tuttle, K.R.; Levin, A.; Nangaku, M.; Kadowaki, T.; Agarwal, R.; Hauske, S.J.; Elsasser, A.; Ritter, I.; Steubl, D.; Wanner, C.; et al. Safety of Empagliflozin in Patients With Type 2 Diabetes and Chronic Kidney Disease: Pooled Analysis of Placebo-Controlled Clinical Trials. *Diabetes Care* **2022**, *45*, 1445-1452, doi:10.2337/dc21-2034.
74. Heise, T.; Mari, A.; DeVries, J.H.; Urva, S.; Li, J.; Pratt, E.J.; Coskun, T.; Thomas, M.K.; Mather, K.J.; Haupt, A.; et al. Effects of subcutaneous tirzepatide versus placebo or semaglutide on pancreatic islet function and insulin sensitivity in adults with type 2 diabetes: a multicentre, randomised, double-blind, parallel-arm, phase 1 clinical trial. *Lancet Diabetes Endocrinol* **2022**, *10*, 418-429, doi:10.1016/S2213-8587(22)00085-7.
75. Fox, C.K.; Clark, J.M.; Rudser, K.D.; Ryder, J.R.; Gross, A.C.; Nathan, B.M.; Sunni, M.; Dengel, D.R.; Billington, C.J.; Bensignor, M.O.; et al. Exenatide for weight-loss maintenance in adolescents with severe obesity: A randomized, placebo-controlled trial. *Obesity (Silver Spring)* **2022**, *30*, 1105-1115, doi:10.1002/oby.23395.
76. Dei Cas, A.; Micheli, M.M.; Aldigeri, R.; Gardini, S.; Ferrari-Pellegrini, F.; Perini, M.; Messa, G.; Antonini, M.; Spigoni, V.; Cinquegrani, G.; et al. Long-acting exenatide does not prevent cognitive decline in mild cognitive impairment: a proof-of-concept clinical trial. *Journal of*

*endocrinological investigation* **2024**, *47*, 2339-2349, doi:10.1007/s40618-024-02320-7.
77. Mu, Y.; Bao, X.; Eliaschewitz, F.G.; Hansen, M.R.; Kim, B.T.; Koroleva, A.; Ma, R.C.W.; Yang, T.; Zu, N.; Liu, M.; et al. Efficacy and safety of once weekly semaglutide 2.4 mg for weight management in a predominantly east Asian population with overweight or obesity (STEP 7): a double-blind, multicentre, randomised controlled trial. *Lancet Diabetes Endocrinol* **2024**, *12*, 184-195, doi:10.1016/S2213-8587(23)00388-1.
78. Lee, B.W.; Cho, Y.M.; Kim, S.G.; Ko, S.H.; Lim, S.; Dahaoui, A.; Jeong, J.S.; Lim, H.J.; Yu, J.M. Efficacy and Safety of Once-Weekly Semaglutide Versus Once-Daily Sitagliptin as Metformin Add-on in a Korean Population with Type 2 Diabetes. *Diabetes Ther* **2024**, *15*, 547-563, doi:10.1007/s13300-023-01515-0.
79. Tuttle, K.R.; Hauske, S.J.; Canziani, M.E.; Caramori, M.L.; Cherney, D.; Cronin, L.; Heerspink, H.J.L.; Hugo, C.; Nangaku, M.; Rotter, R.C.; et al. Efficacy and safety of aldosterone synthase inhibition with and without empagliflozin for chronic kidney disease: a randomised, controlled, phase 2 trial. *Lancet* **2024**, *403*, 379-390, doi:10.1016/S0140-6736(23)02408-X.
80. Wadden, T.A.; Chao, A.M.; Machineni, S.; Kushner, R.; Ard, J.; Srivastava, G.; Halpern, B.; Zhang, S.; Chen, J.; Bunck, M.C.; et al. Tirzepatide after intensive lifestyle intervention in adults with overweight or obesity: the SURMOUNT-3 phase 3 trial. *Nat Med* **2023**, *29*, 2909-2918, doi:10.1038/s41591-023-02597-w.
81. Rosenstock, J.; Frias, J.P.; Rodbard, H.W.; Tofe, S.; Sears, E.; Huh, R.; Fernandez Lando, L.; Patel, H. Tirzepatide vs Insulin Lispro Added to Basal Insulin in Type 2 Diabetes: The SURPASS-6 Randomized Clinical Trial. *Jama* **2023**, *330*, 1631-1640, doi:10.1001/jama.2023.20294.
82. Frias, J.P.; Hsia, S.; Eyde, S.; Liu, R.; Ma, X.; Konig, M.; Kazda, C.; Mather, K.J.; Haupt, A.; Pratt, E.; et al. Efficacy and safety of oral orforglipron in patients with type 2 diabetes: a multicentre, randomised, dose-response, phase 2 study. *Lancet* **2023**, *402*, 472-483, doi:10.1016/S0140-6736(23)01302-8.
83. Feng, P.; Sheng, X.; Ji, Y.; Urva, S.; Wang, F.; Miller, S.; Qian, C.; An, Z.; Cui, Y. A Phase 1 Multiple Dose Study of Tirzepatide in Chinese Patients with Type 2 Diabetes. *Adv Ther* **2023**, *40*, 3434-3445, doi:10.1007/s12325-023-02536-8.
84. Gao, L.; Lee, B.W.; Chawla, M.; Kim, J.; Huo, L.; Du, L.; Huang, Y.; Ji, L. Tirzepatide versus insulin glargine as second-line or third-line therapy in type 2 diabetes in the Asia-Pacific region: the SURPASS-AP-Combo trial. *Nat Med* **2023**, *29*, 1500-1510, doi:10.1038/s41591-

023-02344-1.
85. Buse, J.B.; Nordahl Christensen, H.; Harty, B.J.; Mitchell, J.; Soule, B.P.; Zacherle, E.; Cziraky, M.; Willey, V.J. Study design and baseline profile for adults with type 2 diabetes in the once-weekly subcutaneous SEmaglutide randomized PRAgmatic (SEPRA) trial. *BMJ Open Diabetes Res Care* **2023**, *11*, doi:10.1136/bmjdrc-2022-003206.
86. Bliddal, H.; Bays, H.; Czernichow, S.; Udden Hemmingsson, J.; Hjelmesaeth, J.; Hoffmann Morville, T.; Koroleva, A.; Skov Neergaard, J.; Velez Sanchez, P.; Wharton, S.; et al. Once-Weekly Semaglutide in Persons with Obesity and Knee Osteoarthritis. *N Engl J Med* **2024**, *391*, 1573-1583, doi:10.1056/NEJMoa2403664.
87. Zhao, L.; Cheng, Z.; Lu, Y.; Liu, M.; Chen, H.; Zhang, M.; Wang, R.; Yuan, Y.; Li, X. Tirzepatide for Weight Reduction in Chinese Adults With Obesity: The SURMOUNT-CN Randomized Clinical Trial. *Jama* **2024**, *332*, 551-560, doi:10.1001/jama.2024.9217.
88. Natale, P.; Tunnicliffe, D.J.; Toyama, T.; Palmer, S.C.; Saglimbene, V.M.; Ruospo, M.; Gargano, L.; Stallone, G.; Gesualdo, L.; Strippoli, G.F. Sodium-glucose co-transporter protein 2 (SGLT2) inhibitors for people with chronic kidney disease and diabetes. *The Cochrane database of systematic reviews* **2024**, *5*, CD015588, doi:10.1002/14651858.CD015588.pub2.
89. Wason, S. Efficacy and Bone Safety of Sotagliflozin 400 and 200 mg Versus Placebo in Participants With Type 2 Diabetes Mellitus Who Have Inadequate Glycemic Control (SOTA-BONE). Available online: https://clinicaltrials.gov/study/NCT03386344?cond=NCT03386344&rank=1 (accessed on 2024/10/28).
90. SURMOUNT-J. A Study of Tirzepatide (LY3298176) in Participants With Obesity Disease (SURMOUNT-J). Available online: https://clinicaltrials.gov/study/NCT04844918?cond=NCT04844918&rank=1 (accessed on 2024/10/28).
91. Sorum, M.E.; Gang, A.O.; Tholstrup, D.M.; Gudbrandsdottir, S.; Kissow, H.; Kornblit, B.; Muller, K.; Knop, F.K. Semaglutide treatment for PRevention Of Toxicity in high-dosE Chemotherapy with autologous haematopoietic stem-cell Transplantation (PROTECT): study protocol for a randomised, double-blind, placebo-controlled, investigator-initiated study. *BMJ Open* **2024**, *14*, e089862, doi:10.1136/bmjopen-2024-089862.
92. Aronne, L.J.; Sattar, N.; Horn, D.B.; Bays, H.E.; Wharton, S.; Lin, W.Y.; Ahmad, N.N.; Zhang, S.; Liao, R.; Bunck, M.C.; et al. Continued Treatment With Tirzepatide for Maintenance of Weight Reduction in Adults With Obesity: The SURMOUNT-4 Randomized Clinical Trial.

*Jama* **2024**, *331*, 38-48, doi:10.1001/jama.2023.24945.
93. Cherney, D.Z.I.; Ferrannini, E.; Umpierrez, G.E.; Peters, A.L.; Rosenstock, J.; Powell, D.R.; Davies, M.J.; Banks, P.; Agarwal, R. Efficacy and safety of sotagliflozin in patients with type 2 diabetes and stage 3 chronic kidney disease. *Diabetes Obes Metab* **2023**, *25*, 1646-1657, doi:10.1111/dom.15019.
94. Garvey, W.T.; Frias, J.P.; Jastreboff, A.M.; le Roux, C.W.; Sattar, N.; Aizenberg, D.; Mao, H.; Zhang, S.; Ahmad, N.N.; Bunck, M.C.; et al. Tirzepatide once weekly for the treatment of obesity in people with type 2 diabetes (SURMOUNT-2): a double-blind, randomised, multicentre, placebo-controlled, phase 3 trial. *Lancet* **2023**, *402*, 613-626, doi:10.1016/S0140-6736(23)01200-X.
95. The, E.-K.C.G.; Herrington, W.G.; Staplin, N.; Wanner, C.; Green, J.B.; Hauske, S.J.; Emberson, J.R.; Preiss, D.; Judge, P.; Mayne, K.J.; et al. Empagliflozin in Patients with Chronic Kidney Disease. *N Engl J Med* **2023**, *388*, 117-127, doi:10.1056/NEJMoa2204233.
96. Lincoff, A.M.; Brown-Frandsen, K.; Colhoun, H.M.; Deanfield, J.; Emerson, S.S.; Esbjerg, S.; Hardt-Lindberg, S.; Hovingh, G.K.; Kahn, S.E.; Kushner, R.F.; et al. Semaglutide and Cardiovascular Outcomes in Obesity without Diabetes. *N Engl J Med* **2023**, *389*, 2221-2232, doi:10.1056/NEJMoa2307563.
97. Jastreboff, A.M.; Aronne, L.J.; Ahmad, N.N.; Wharton, S.; Connery, L.; Alves, B.; Kiyosue, A.; Zhang, S.; Liu, B.; Bunck, M.C.; et al. Tirzepatide Once Weekly for the Treatment of Obesity. *N Engl J Med* **2022**, *387*, 205-216, doi:10.1056/NEJMoa2206038.
98. Solomon, S.D.; McMurray, J.J.V.; Claggett, B.; de Boer, R.A.; DeMets, D.; Hernandez, A.F.; Inzucchi, S.E.; Kosiborod, M.N.; Lam, C.S.P.; Martinez, F.; et al. Dapagliflozin in Heart Failure with Mildly Reduced or Preserved Ejection Fraction. *N Engl J Med* **2022**, *387*, 1089-1098, doi:10.1056/NEJMoa2206286.
99. Wada, T.; Mori-Anai, K.; Takahashi, A.; Matsui, T.; Inagaki, M.; Iida, M.; Maruyama, K.; Tsuda, H. Effect of canagliflozin on the decline of estimated glomerular filtration rate in chronic kidney disease patients with type 2 diabetes mellitus: A multicenter, randomized, double-blind, placebo-controlled, parallel-group, phase III study in Japan. *J Diabetes Investig* **2022**, *13*, 1981-1989, doi:10.1111/jdi.13888.
100. Anker, S.D.; Butler, J.; Filippatos, G.; Ferreira, J.P.; Bocchi, E.; Bohm, M.; Brunner-La Rocca, H.P.; Choi, D.J.; Chopra, V.; Chuquiure-Valenzuela, E.; et al. Empagliflozin in Heart Failure with a Preserved Ejection Fraction. *N Engl J Med* **2021**, *385*, 1451-1461, doi:10.1056/NEJMoa2107038.

101. Bhatt, D.L.; Szarek, M.; Pitt, B.; Cannon, C.P.; Leiter, L.A.; McGuire, D.K.; Lewis, J.B.; Riddle, M.C.; Inzucchi, S.E.; Kosiborod, M.N.; et al. Sotagliflozin in Patients with Diabetes and Chronic Kidney Disease. *N Engl J Med* **2021**, *384*, 129-139, doi:10.1056/NEJMoa2030186.
102. Davies, M.; Faerch, L.; Jeppesen, O.K.; Pakseresht, A.; Pedersen, S.D.; Perreault, L.; Rosenstock, J.; Shimomura, I.; Viljoen, A.; Wadden, T.A.; et al. Semaglutide 2.4 mg once a week in adults with overweight or obesity, and type 2 diabetes (STEP 2): a randomised, double-blind, double-dummy, placebo-controlled, phase 3 trial. *Lancet* **2021**, *397*, 971-984, doi:10.1016/S0140-6736(21)00213-0.
103. Del Prato, S.; Kahn, S.E.; Pavo, I.; Weerakkody, G.J.; Yang, Z.; Doupis, J.; Aizenberg, D.; Wynne, A.G.; Riesmeyer, J.S.; Heine, R.J.; et al. Tirzepatide versus insulin glargine in type 2 diabetes and increased cardiovascular risk (SURPASS-4): a randomised, open-label, parallel-group, multicentre, phase 3 trial. *Lancet* **2021**, *398*, 1811-1824, doi:10.1016/S0140-6736(21)02188-7.
104. Frias, J.P.; Davies, M.J.; Rosenstock, J.; Perez Manghi, F.C.; Fernandez Lando, L.; Bergman, B.K.; Liu, B.; Cui, X.; Brown, K.; Investigators, S.-. Tirzepatide versus Semaglutide Once Weekly in Patients with Type 2 Diabetes. *N Engl J Med* **2021**, *385*, 503-515, doi:10.1056/NEJMoa2107519.
105. Gerstein, H.C.; Sattar, N.; Rosenstock, J.; Ramasundarahettige, C.; Pratley, R.; Lopes, R.D.; Lam, C.S.P.; Khurmi, N.S.; Heenan, L.; Del Prato, S.; et al. Cardiovascular and Renal Outcomes with Efpeglenatide in Type 2 Diabetes. *N Engl J Med* **2021**, *385*, 896-907, doi:10.1056/NEJMoa2108269.
106. Lock, J.P. Bexagliflozin Efficacy and Safety Trial (BEST). Available online: https://clinicaltrials.gov/study/NCT02558296?cond=NCT02558296&rank=1 (accessed on 2024/10/28).
107. Rubino, D.; Abrahamsson, N.; Davies, M.; Hesse, D.; Greenway, F.L.; Jensen, C.; Lingvay, I.; Mosenzon, O.; Rosenstock, J.; Rubio, M.A.; et al. Effect of Continued Weekly Subcutaneous Semaglutide vs Placebo on Weight Loss Maintenance in Adults With Overweight or Obesity: The STEP 4 Randomized Clinical Trial. *Jama* **2021**, *325*, 1414-1425, doi:10.1001/jama.2021.3224.
108. Wason, S. Efficacy and Safety of Sotagliflozin Versus Placebo in Participants With Type 2 Diabetes Mellitus Who Have Inadequate Glycemic Control While Taking Insulin Alone or With Other Oral Antidiabetic Agents (SOTA-INS). Available online: https://clinicaltrials.gov/study/NCT03285594?cond=NCT03285594&rank=1 (accessed on 2024/10/28).
109. Wilding, J.P.H.; Batterham, R.L.; Calanna, S.; Davies, M.; Van Gaal, L.F.; Lingvay, I.; McGowan, B.M.; Rosenstock, J.; Tran, M.T.D.; Wadden,

T.A.; et al. Once-Weekly Semaglutide in Adults with Overweight or Obesity. *N Engl J Med* **2021**, *384*, 989-1002, doi:10.1056/NEJMoa2032183.
110. Cannon, C.P.; Pratley, R.; Dagogo-Jack, S.; Mancuso, J.; Huyck, S.; Masiukiewicz, U.; Charbonnel, B.; Frederich, R.; Gallo, S.; Cosentino, F.; et al. Cardiovascular Outcomes with Ertugliflozin in Type 2 Diabetes. *N Engl J Med* **2020**, *383*, 1425-1435, doi:10.1056/NEJMoa2004967.
111. Heerspink, H.J.L.; Stefansson, B.V.; Correa-Rotter, R.; Chertow, G.M.; Greene, T.; Hou, F.F.; Mann, J.F.E.; McMurray, J.J.V.; Lindberg, M.; Rossing, P.; et al. Dapagliflozin in Patients with Chronic Kidney Disease. *N Engl J Med* **2020**, *383*, 1436-1446, doi:10.1056/NEJMoa2024816.
112. Packer, M.; Anker, S.D.; Butler, J.; Filippatos, G.; Pocock, S.J.; Carson, P.; Januzzi, J.; Verma, S.; Tsutsui, H.; Brueckmann, M.; et al. Cardiovascular and Renal Outcomes with Empagliflozin in Heart Failure. *N Engl J Med* **2020**, *383*, 1413-1424, doi:10.1056/NEJMoa2022190.
113. Gallo, S.; Charbonnel, B.; Goldman, A.; Shi, H.; Huyck, S.; Darekar, A.; Lauring, B.; Terra, S.G. Long-term efficacy and safety of ertugliflozin in patients with type 2 diabetes mellitus inadequately controlled with metformin monotherapy: 104-week VERTIS MET trial. *Diabetes Obes Metab* **2019**, *21*, 1027-1036, doi:10.1111/dom.13631.
114. Gerstein, H.C.; Colhoun, H.M.; Dagenais, G.R.; Diaz, R.; Lakshmanan, M.; Pais, P.; Probstfield, J.; Riesmeyer, J.S.; Riddle, M.C.; Ryden, L.; et al. Dulaglutide and cardiovascular outcomes in type 2 diabetes (REWIND): a double-blind, randomised placebo-controlled trial. *Lancet* **2019**, *394*, 121-130, doi:10.1016/S0140-6736(19)31149-3.
115. Husain, M.; Birkenfeld, A.L.; Donsmark, M.; Dungan, K.; Eliaschewitz, F.G.; Franco, D.R.; Jeppesen, O.K.; Lingvay, I.; Mosenzon, O.; Pedersen, S.D.; et al. Oral Semaglutide and Cardiovascular Outcomes in Patients with Type 2 Diabetes. *N Engl J Med* **2019**, *381*, 841-851, doi:10.1056/NEJMoa1901118.
116. McMurray, J.J.V.; Solomon, S.D.; Inzucchi, S.E.; Kober, L.; Kosiborod, M.N.; Martinez, F.A.; Ponikowski, P.; Sabatine, M.S.; Anand, I.S.; Belohlavek, J.; et al. Dapagliflozin in Patients with Heart Failure and Reduced Ejection Fraction. *N Engl J Med* **2019**, *381*, 1995-2008, doi:10.1056/NEJMoa1911303.
117. Perkovic, V.; Jardine, M.J.; Neal, B.; Bompoint, S.; Heerspink, H.J.L.; Charytan, D.M.; Edwards, R.; Agarwal, R.; Bakris, G.; Bull, S.; et al.

Canagliflozin and Renal Outcomes in Type 2 Diabetes and Nephropathy. *N Engl J Med* **2019**, *380*, 2295-2306, doi:10.1056/NEJMoa1811744.
118. Rosenstock, J.; Allison, D.; Birkenfeld, A.L.; Blicher, T.M.; Deenadayalan, S.; Jacobsen, J.B.; Serusclat, P.; Violante, R.; Watada, H.; Davies, M.; et al. Effect of Additional Oral Semaglutide vs Sitagliptin on Glycated Hemoglobin in Adults With Type 2 Diabetes Uncontrolled With Metformin Alone or With Sulfonylurea: The PIONEER 3 Randomized Clinical Trial. *Jama* **2019**, *321*, 1466-1480, doi:10.1001/jama.2019.2942.
119. Wiviott, S.D.; Raz, I.; Bonaca, M.P.; Mosenzon, O.; Kato, E.T.; Cahn, A.; Silverman, M.G.; Zelniker, T.A.; Kuder, J.F.; Murphy, S.A.; et al. Dapagliflozin and Cardiovascular Outcomes in Type 2 Diabetes. *N Engl J Med* **2019**, *380*, 347-357, doi:10.1056/NEJMoa1812389.
120. Danne, T.; Cariou, B.; Banks, P.; Brandle, M.; Brath, H.; Franek, E.; Kushner, J.A.; Lapuerta, P.; McGuire, D.K.; Peters, A.L.; et al. HbA(1c) and Hypoglycemia Reductions at 24 and 52 Weeks With Sotagliflozin in Combination With Insulin in Adults With Type 1 Diabetes: The European inTandem2 Study. *Diabetes Care* **2018**, *41*, 1981-1990, doi:10.2337/dc18-0342.
121. Grunberger, G.; Camp, S.; Johnson, J.; Huyck, S.; Terra, S.G.; Mancuso, J.P.; Jiang, Z.W.; Golm, G.; Engel, S.S.; Lauring, B. Ertugliflozin in Patients with Stage 3 Chronic Kidney Disease and Type 2 Diabetes Mellitus: The VERTIS RENAL Randomized Study. *Diabetes Ther* **2018**, *9*, 49-66, doi:10.1007/s13300-017-0337-5.
122. Hernandez, A.F.; Green, J.B.; Janmohamed, S.; D'Agostino, R.B., Sr.; Granger, C.B.; Jones, N.P.; Leiter, L.A.; Rosenberg, A.E.; Sigmon, K.N.; Somerville, M.C.; et al. Albiglutide and cardiovascular outcomes in patients with type 2 diabetes and cardiovascular disease (Harmony Outcomes): a double-blind, randomised placebo-controlled trial. *Lancet* **2018**, *392*, 1519-1529, doi:10.1016/S0140-6736(18)32261-X.
123. Kaku, K.; Yamada, Y.; Watada, H.; Abiko, A.; Nishida, T.; Zacho, J.; Kiyosue, A. Safety and efficacy of once-weekly semaglutide vs additional oral antidiabetic drugs in Japanese people with inadequately controlled type 2 diabetes: A randomized trial. *Diabetes Obes Metab* **2018**, *20*, 1202-1212, doi:10.1111/dom.13218.
124. Holman, R.R.; Bethel, M.A.; Mentz, R.J.; Thompson, V.P.; Lokhnygina, Y.; Buse, J.B.; Chan, J.C.; Choi, J.; Gustavson, S.M.; Iqbal, N.; et al. Effects of Once-Weekly Exenatide on Cardiovascular Outcomes in Type 2 Diabetes. *N Engl J Med* **2017**, *377*, 1228-1239, doi:10.1056/NEJMoa1612917.

125. Neal, B.; Perkovic, V.; Mahaffey, K.W.; de Zeeuw, D.; Fulcher, G.; Erondu, N.; Shaw, W.; Law, G.; Desai, M.; Matthews, D.R.; et al. Canagliflozin and Cardiovascular and Renal Events in Type 2 Diabetes. *N Engl J Med* **2017**, *377*, 644-657, doi:10.1056/NEJMoa1611925.
126. Hadjadj, S.; Rosenstock, J.; Meinicke, T.; Woerle, H.J.; Broedl, U.C. Initial Combination of Empagliflozin and Metformin in Patients With Type 2 Diabetes. *Diabetes Care* **2016**, *39*, 1718-1728, doi:10.2337/dc16-0522.
127. Marso, S.P.; Daniels, G.H.; Brown-Frandsen, K.; Kristensen, P.; Mann, J.F.; Nauck, M.A.; Nissen, S.E.; Pocock, S.; Poulter, N.R.; Ravn, L.S.; et al. Liraglutide and Cardiovascular Outcomes in Type 2 Diabetes. *N Engl J Med* **2016**, *375*, 311-322, doi:10.1056/NEJMoa1603827.
128. Marso, S.P.; Bain, S.C.; Consoli, A.; Eliaschewitz, F.G.; Jodar, E.; Leiter, L.A.; Lingvay, I.; Rosenstock, J.; Seufert, J.; Warren, M.L.; et al. Semaglutide and Cardiovascular Outcomes in Patients with Type 2 Diabetes. *N Engl J Med* **2016**, *375*, 1834-1844, doi:10.1056/NEJMoa1607141.
129. Nauck, M.; Rizzo, M.; Johnson, A.; Bosch-Traberg, H.; Madsen, J.; Cariou, B. Once-Daily Liraglutide Versus Lixisenatide as Add-on to Metformin in Type 2 Diabetes: A 26-Week Randomized Controlled Clinical Trial. *Diabetes Care* **2016**, *39*, 1501-1509, doi:10.2337/dc15-2479.
130. Nauck, M.A.; Stewart, M.W.; Perkins, C.; Jones-Leone, A.; Yang, F.; Perry, C.; Reinhardt, R.R.; Rendell, M. Efficacy and safety of once-weekly GLP-1 receptor agonist albiglutide (HARMONY 2): 52 week primary endpoint results from a randomised, placebo-controlled trial in patients with type 2 diabetes mellitus inadequately controlled with diet and exercise. *Diabetologia* **2016**, *59*, 266-274, doi:10.1007/s00125-015-3795-1.
131. Giorgino, F.; Benroubi, M.; Sun, J.H.; Zimmermann, A.G.; Pechtner, V. Efficacy and Safety of Once-Weekly Dulaglutide Versus Insulin Glargine in Patients With Type 2 Diabetes on Metformin and Glimepiride (AWARD-2). *Diabetes Care* **2015**, *38*, 2241-2249, doi:10.2337/dc14-1625.
132. Pfeffer, M.A.; Claggett, B.; Diaz, R.; Dickstein, K.; Gerstein, H.C.; Kober, L.V.; Lawson, F.C.; Ping, L.; Wei, X.; Lewis, E.F.; et al. Lixisenatide in Patients with Type 2 Diabetes and Acute Coronary Syndrome. *N Engl J Med* **2015**, *373*, 2247-2257, doi:10.1056/NEJMoa1509225.
133. Pi-Sunyer, X.; Astrup, A.; Fujioka, K.; Greenway, F.; Halpern, A.; Krempf, M.; Lau, D.C.; le Roux, C.W.; Violante Ortiz, R.; Jensen, C.B.; et al. A Randomized, Controlled Trial of 3.0 mg of Liraglutide in Weight Management. *N Engl J Med* **2015**, *373*, 11-22,

doi:10.1056/NEJMoa1411892.
134. Zinman, B.; Wanner, C.; Lachin, J.M.; Fitchett, D.; Bluhmki, E.; Hantel, S.; Mattheus, M.; Devins, T.; Johansen, O.E.; Woerle, H.J.; et al. Empagliflozin, Cardiovascular Outcomes, and Mortality in Type 2 Diabetes. *N Engl J Med* **2015**, *373*, 2117-2128, doi:10.1056/NEJMoa1504720.
135. Ridderstrale, M.; Andersen, K.R.; Zeller, C.; Kim, G.; Woerle, H.J.; Broedl, U.C.; investigators, E.-R.H.H.S.t. Comparison of empagliflozin and glimepiride as add-on to metformin in patients with type 2 diabetes: a 104-week randomised, active-controlled, double-blind, phase 3 trial. *Lancet Diabetes Endocrinol* **2014**, *2*, 691-700, doi:10.1016/S2213-8587(14)70120-2.
136. Rosenstock, J.; Fonseca, V.A.; Gross, J.L.; Ratner, R.E.; Ahren, B.; Chow, F.C.; Yang, F.; Miller, D.; Johnson, S.L.; Stewart, M.W.; et al. Advancing basal insulin replacement in type 2 diabetes inadequately controlled with insulin glargine plus oral agents: a comparison of adding albiglutide, a weekly GLP-1 receptor agonist, versus thrice-daily prandial insulin lispro. *Diabetes Care* **2014**, *37*, 2317-2325, doi:10.2337/dc14-0001.
137. Umpierrez, G.; Tofe Povedano, S.; Perez Manghi, F.; Shurzinske, L.; Pechtner, V. Efficacy and safety of dulaglutide monotherapy versus metformin in type 2 diabetes in a randomized controlled trial (AWARD-3). *Diabetes Care* **2014**, *37*, 2168-2176, doi:10.2337/dc13-2759.
138. Weissman, P.N.; Carr, M.C.; Ye, J.; Cirkel, D.T.; Stewart, M.; Perry, C.; Pratley, R. HARMONY 4: randomised clinical trial comparing once-weekly albiglutide and insulin glargine in patients with type 2 diabetes inadequately controlled with metformin with or without sulfonylurea. *Diabetologia* **2014**, *57*, 2475-2484, doi:10.1007/s00125-014-3360-3.
139. Ferrannini, E.; Berk, A.; Hantel, S.; Pinnetti, S.; Hach, T.; Woerle, H.J.; Broedl, U.C. Long-term safety and efficacy of empagliflozin, sitagliptin, and metformin: an active-controlled, parallel-group, randomized, 78-week open-label extension study in patients with type 2 diabetes. *Diabetes Care* **2013**, *36*, 4015-4021, doi:10.2337/dc13-0663.
140. Wilding, J.P.; Woo, V.; Soler, N.G.; Pahor, A.; Sugg, J.; Rohwedder, K.; Parikh, S.; Dapagliflozin 006 Study, G. Long-term efficacy of dapagliflozin in patients with type 2 diabetes mellitus receiving high doses of insulin: a randomized trial. *Ann Intern Med* **2012**, *156*, 405-415, doi:10.7326/0003-4819-156-6-201203200-00003.
141. Bailey, C.J.; Gross, J.L.; Pieters, A.; Bastien, A.; List, J.F. Effect of dapagliflozin in patients with type 2 diabetes who have inadequate

glycaemic control with metformin: a randomised, double-blind, placebo-controlled trial. *Lancet* **2010**, *375*, 2223-2233, doi:10.1016/S0140-6736(10)60407-2.

142. Buse, J.B.; Rosenstock, J.; Sesti, G.; Schmidt, W.E.; Montanya, E.; Brett, J.H.; Zychma, M.; Blonde, L.; Group, L.-S. Liraglutide once a day versus exenatide twice a day for type 2 diabetes: a 26-week randomised, parallel-group, multinational, open-label trial (LEAD-6). *Lancet* **2009**, *374*, 39-47, doi:10.1016/S0140-6736(09)60659-0.

143. Nauck, M.; Frid, A.; Hermansen, K.; Shah, N.S.; Tankova, T.; Mitha, I.H.; Zdravkovic, M.; During, M.; Matthews, D.R.; Group, L.-S. Efficacy and safety comparison of liraglutide, glimepiride, and placebo, all in combination with metformin, in type 2 diabetes: the LEAD (liraglutide effect and action in diabetes)-2 study. *Diabetes Care* **2009**, *32*, 84-90, doi:10.2337/dc08-1355.
